# Tibetan tea reduces obesity brought on by a high‐fat diet and modulates gut flora in mice

**DOI:** 10.1002/fsn3.3607

**Published:** 2023-08-07

**Authors:** Gang He, Tangcong Chen, Lifen Huang, Yiyuan Zhang, Yanjiao Feng, Qijun Liu, Xiaojing Yin, Shaokui Qu, Chen Yang, Jianghong Wan, Li Liang, Jun Yan, Wei Liu

**Affiliations:** ^1^ Key Laboratory of Medicinal and Edible Plants Resources Development of Sichuan Education Department Sichuan Industrial Institute of Antibiotics, School of Pharmacy, Chengdu University Chengdu China; ^2^ Sichuan Jiang's Tibetan Tea Co., LTD Ya'an China

**Keywords:** gut flora, high‐fat diet, obesity, short‐chain fatty acids, Tibetan tea

## Abstract

It has been shown that Tibetan tea (TT) inhibits obesity and controls lipid metabolism. The fundamental processes by which TT prevents obesity are yet entirely unknown. Consequently, this research aimed to ascertain if TT may prevent obesity by modifying the gut flora. Our research demonstrated that TT prevented mice from gaining weight and accumulating fat due to the high‐fat diet (HFD), decreased levels of blood total cholesterol (TC), triglycerides (TG), and low‐density lipoprotein cholesterol (LDL‐C), and raised levels of high‐density lipoprotein cholesterol (HDL‐C). Adipogenesis‐related genes such as acetyl‐Coenzyme A carboxylase 1 (ACC1, LOC107476), fatty acid synthase (Fas, LOC14104), sterol regulatory element‐binding protein‐1c (SREBP‐1c, LOC20787), CCAAT/enhancer‐binding protein α (C/EBPα, LOC12606), stearoyl‐CoA desaturase 1 (SCD1, LOC20249), and peroxisome proliferator‐activated receptor γ (PPARγ, LOC19016) had their expression downregulated by lowering the *Firmicutes/Bacteroidetes* (*F/B*) ratio and controlling the number of certain gut bacteria. TT also alleviated HFD‐induced abnormalities of the gut microbiota. The *Muribaculaceae*, *Lachnospiraceae NK4A136_group*, *Alistipes*, and *Odoribacter* families were identified as the major beneficial gut microorganisms using Spearman's correlation analysis. Fecal microbiota transplantation (FMT) demonstrated that TT's anti‐obesity and gut microbiota‐modulating benefits might be transmitted to mice on an HFD, demonstrating that one of TT's targets for preventing obesity is the gut microbiota. TT also increased the amount of short‐chain fatty acids (SCFAs) in the feces, including acetic, propionic, and butyric acids. These results indicate the possible development of TT as a prebiotic to combat obesity and associated disorders. These results suggest that TT may act as a prebiotic against obesity and its associated diseases.

## Graphical abstract

We were able to demonstrate that Tibetan tea (TT) dramatically inhibited high‐fat diet‐induced weight gain, fat accumulation, hyperglycemia, and hyperlipidemia in mice, as well as control the expression of genes involved in lipid metabolism. The way that TT controls the gut microbiota produces short‐chain fatty acids and controls the expression of genes involved in lipid synthesis may be responsible for its positive effects on obesity. Our research gave TT's potential as a functional beverage for the prevention and treatment of obesity a new direction.

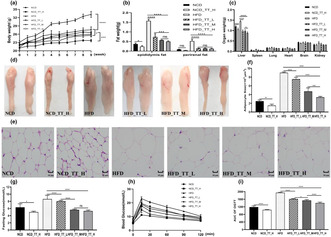

## 1 INTRODUCTION

Obesity is a chronic condition that may cause a variety of health issues and shorten life expectancy (Blüher, 2013). Weight increase is merely one sign of obesity. However, it is also linked to problems with lipid and blood glucose metabolism, chronic inflammation, and a higher risk of many illnesses, including type 2 diabetes, hypertension, and cancer (Saltiel & Olefsky, 2017). Obesity has a complicated etiology influenced by hereditary and non‐genetic variables. In recent years, mounting research has revealed that obesity may be caused by an imbalance in the gut flora (Liu, Liu, et al., 2021).

The gut microbiome consists of thousands of bacterial species, mainly *Bacteroidota*, *Firmicutes*, *Proteobacteria*, and *Actinobacteria* (Benahmed et al., 2021; Pung et al., 2022). The development of obesity is strongly correlated with gut microbiota, according to several researches (He et al., 2022; Kang et al., 2022; Su et al., 2022). The development of obesity is impacted by how a high‐fat diet (HFD) changes the gut microbiota's composition by drastically increasing the ratio of *Firmicutes* to *Bacteroidota* (*F/B*) (Houtman et al., 2022; Indiani et al., 2018).

Numerous natural bioactive substances, including dietary polysaccharides, have been shown to suppress obesity and control lipid metabolism via controlling the gut microbiota in recent years (Li, Ma, et al., 2022; Rehman et al., 2022; Zhang et al., 2021). These findings imply that dietary interventions for obesity may affect the gut microbiome (Asadi et al., 2022).

Depending on processing techniques and production regions, tea is classified as green, yellow, white, oolong, dark, and black (Xu et al., 2018). *Tibetan* tea (TT) is a type of dark tea, mainly produced in Ya'an, Sichuan Province, China, and has a history of about 1000 years (Xie et al., 2018). TT is a geographically iconic ethnic product made from several processes, which give it its unique flavor. The most important of these is pile fermentation (Zheng et al., 2020), in which microorganisms perform a variety of reactions on the tea leaves, including degradation, oxidation, and condensation, changing the tea's chemical compounds (Li et al., 2018). The Tibetan's extreme diet (red meat and zanba as the primary food) is high in cholesterol, fat, and sugar. The *Tibetan* ancestors found that drinking TT could keep them healthy and avoid the harm of hyperlipidemia, hyperglycemia, hypertension, and other diseases caused by obesity (Li, Zhang, et al., 2022). Many studies have shown that TT has a variety of pharmacological effects, such as weight loss (Yuan et al., 2016), antioxidants (Xie et al., 2018), anti‐radiation (Yuan et al., 2016), and protection against ulcerative colitis (Wang et al., 2021).

Although studies have reported many benefits of TT, its effect on obesity is unclear. The lipid and glucose metabolism, as well as weight, in TT‐gavaged HFD‐induced obese mice, were the main subjects of this work. By transplanting fecal bacteria, we confirmed the gut microbiota's involvement in preventing obesity and further our investigation into the gut microbiota's structure. Our study suggests that TT can treat obesity and its complications through dietary intervention.

## 2 MATERIALS AND METHODS

### 2.1 Materials

The experimental animals were given both a low‐fat diet and a typical control diet. The standard control diet from Keao Xieli Feed Co., Ltd., had 3.40 kcal/g of fat, 11.85% of protein, and 65.08% of carbs. The high‐fat diet, which had 5.13 kcal/g of calories and included 27.20% carbs, 34.5% fat, and 23.25% protein, was purchased from Jiangsu Pharmaceutical & Bioengineering Co., Ltd. Caffeine, gallic acid (GA), catechin, epigallocatechin gallate (EGCG), (−)‐epigallocatechin (EGC), and epicatechin (EC) were purchased from Yuanye Biotechnology Co., Ltd. Methanol, anhydrous ethanol, acetonitrile, and phenol reagents (all HPLC grade) were purchased from Sigma‐Aldrich. Other reagents such as acetic acid, propionic acid, butyric acid, and isobutyric acid are of analytical grade (Kelong Chemical Co., Ltd.).

### 2.2 Preparation of TT water extracts and compositional analysis

TT was extracted as described by Gong et al. (2020) with minor modifications. 500 g TT (Sichuan Jiang's Tibetan Tea Co., Ltd.) was crushed and extracted in 5 L purified water for 30 min, and the water temperature was maintained at 90°C. Ultrasonic extraction was performed for 10 min, and filter residue and filtrate were collected by filtration solution. Add 1.5 L pure water to the filter residue, extract at 90°C for 20 min, and collect the filtrate. The filtrate was combined twice, concentrated to 1/10 volume, and vacuum freeze‐dried to obtain the TT extract.

The quantification of tea polyphenols, total flavonoids, and soluble sugar in TT water extracts was carried out by the Folin–Ciocalteu colorimetric method (Pérez‐Burillo et al., 2018), the aluminum trichloride‐sodium nitrite colorimetric assay (Liu et al., 2018), and the phenol‐sulfuric acid method (Liu, Li, et al., 2021), respectively. Non‐targeted metabolomic analysis using liquid chromatography with tandem mass spectrometry (LC–MS/MS) (Liu et al., 2022). The content of several chemicals, such as catechin, caffeine, GA, EGCG, EGC, and EC, was then determined by high‐performance liquid chromatography (Liu et al., 2022).

### 2.3 Animals and experimental design

At eight weeks old and 20 ± 2 g each, 60 male C57BL/6J mice were purchased from SiPeiFu Biotechnology Co., Ltd. in Beijing, China. They were given access to food, water, and a 12‐h light/dark cycle while being housed in a controlled environment with a temperature of 22 ± 2°C and a humidity level of 55% ± 5%. The Sichuan Industrial Institute of Antibiotics' Ethical Committee at Chengdu University gave its approval for the animal trials (Approval Number: SIIA 20210706). After a week of acclimatization, the mice were randomly divided into six groups, each with ten mice: (1) *NCD*, which received standard chow and was treated with 0.9% saline; (2) *NCD_TT_H*, which received standard chow and was treated with 400 mg/kg of TT extract; (3) *HFD*, which received high‐fat chow and was treated with 0.9% saline; (4) *HFD_TT_L*, which received high‐fat feed and was treated with 100 mg/kg of TT extract; (5) *HFD_TT_M* group, which received high‐fat feed and was treated with 200 mg/kg of TT extract; (6) *HFD_TT_H* group, which received high‐fat feed and was treated with 400 mg/kg of TT extract. The weight of the mice was noted weekly throughout the 9‐week trial. After a 12‐h fast, mice were slaughtered at the end of the ninth week, and blood and fat samples were taken. The other tissue was snap‐frozen in liquid nitrogen, and the epididymal fat was preserved in 4% formaldehyde for further study.

### 2.4 Fecal microbiota transplantation

Fecal microbiota transplantation (FMT) was performed according to the method described by Chang et al. (2015). Briefly, mice in the *NCD*, *NCD_TT_H*, *HFD*, and *HFD_TT_H* groups were fed for 2 months and then used as donor mice. The feces of each of the four groups of mice were collected in sterile cages; 200 mg of feces were resuspended in 2 mL of sterile saline, vortexed for 10 s, and then centrifuged at 800 *g* for 3 min. Eight‐week‐old male recipient mice were fed HFD and gavaged with fresh graft samples (100 μL per mouse) daily for two months.

### 2.5 Biochemical analysis

According to the protocol of the kit (Jiancheng, Inc.), the serum concentrations of high‐density lipoprotein cholesterol (HDL‐C), low‐density lipoprotein cholesterol (LDL‐C), total cholesterol (TC), and triglyceride (TG) were measured.

### 2.6 Oral glucose tolerance test

The mice fasted for 12 h at week 8. 1g of glucose per kilogram of body weight was gavaged, and blood glucose levels were checked at 0, 15, 30, 60, 90, and 120 min later.

### 2.7 RNA extraction and analysis of gene expression

Through the use of the Trizol reagent procedure, total RNA was extracted from the tissue. Using Beijing Labgic Technology Co., Ltd.'s Reverse Transcription kit (which includes a dsDNase), cDNA was produced from an equal volume of total RNA. A StepOnePlusTM Real‐Time PCR Detection System from Applied Biosystems was used to evaluate the generated cDNA.

The Table S2 included the PCR primer sequences for the associated genes. Forty cycles of 95°C for 120 s, 95°C for 5 s, and 60°C for 10 s were amplified during the PCR's 3 min at 95°C. Glyceraldehyde‐3‐phosphate dehydrogenase (GAPDH, LOC14433) was used as an internal reference to compute the relative amount using the 2−^ΔΔ*C*t^ technique.

### 2.8 Histopathological analysis

A 4% formaldehyde solution was used to fix freshly removed mouse epididymal fat overnight. Later, it underwent dehydration, embedding, sectioning, and morphological analysis using hematoxylin and eosin staining.

### 2.9 Gas chromatography–mass spectrometry analysis

Short‐chain fatty acids were detected in feces using the technique previously described (Zhang et al., 2020). The fecal sample was thawed, suspended in 1 mL of 25% methanol solution, and vortexed rapidly for 5 min. After centrifuging at 9568 *g* at 4°C for 10 min and performing a static extraction at 4°C for 30 min, the supernatant was recovered. 0.04 M HCl (10:1, V/V) and the supernatant were combined before standing overnight. Before analysis, the supernatant was put into a clean micro gas‐phase vial after being filtered via a 0.22 μm membrane. Gas chromatography and mass spectrometry (GC–MS) equipment from Perkin Elmer was used to analyze the samples (Perkin Elmer Technologies).

### 2.10 DNA extraction and sequencing of 16S rRNA

According to the manufacturer's recommendations, DNA was extracted from feces samples using the MagPure Soil DNA LQ Kit (Magen). Thermo Fisher Scientific, microspectrophotometer was used to quantify the DNA content and integrity, and agarose gel electrophoresis was used to corroborate the results further. Using two universal primer pairs (343F: 5′‐TACGGRAGGCAGCAG‐3′; 798R: 5′‐ AGGGTATCTAATCCT‐3′), the V3‐V4 hypervariable portions of the bacterial 16S rRNA gene were amplified. Over a 25‐L response. AMPure XP beads (Beckman Coulter Co.) were used to purify the PCR products, and a Qubit dsDNA assay kit was used to quantify them. The concentrations were then modified for sequencing. OE Biotechnology Ltd. carried out sequencing and analysis of the 16S rRNA gene amplicon in Shanghai, China.

### 2.11 Analytical statistics

The difference was shown to be significant using one‐way ANOVA and the Tukey test. A statistically significant value of *p* < .05 was used to represent the data as mean ± SD (standard deviation). The studies utilized GraphPad Prism 9.0 (San Diego).

## 3 RESULTS

### 3.1 Chemical composition of TT water extracts

The chemical composition and content of TT water extracts are shown in Table 1. The most dominant chemicals in TT water extracts are tea polyphenols, flavonoids, and soluble sugars. We used non‐targeted metabolomic analysis to validate the non‐volatile metabolism of Tibetan tea water extracts, and the results are shown in Table S1. These include a wide range of amino acids and their derivatives, phenolic acids, flavonoids, terpenoids, alkaloids, lipids, and other metabolites. The contents of caffeine, GA, EGCG, EGC, and EC were analyzed by high‐performance liquid chromatography, and the results are shown in Table 1. The caffeine content is higher than several other ingredients.

**TABLE 1:** Profiles of chemical components of Tibetan tea water extracts.

Components	Content (mg/g)
Tea polyphenols	596.91 ± 4.24
Total flavonoids	83.60 ± 1.90
Soluble sugar	179.43 ± 3.35
GA	79.69 ± 2.91
EGC	11.20 ± 2.00
Catechin	10.40 ± 1.10
Caffeine	95.50 ± 0.40
EC	4.45 ± 0.05
EGCG	No detected

*Note*: Data are expressed as mean ± SD (*n* = 6).

Abbreviations: EC, (−)‐epicatechin; EGC, (−)‐epigallocatechin; EGCG, epigallocatechin gallate; GA, gallic acid.

### 3.2 TT inhibits HFD‐induced obesity in mice

**FIGURE 1:**
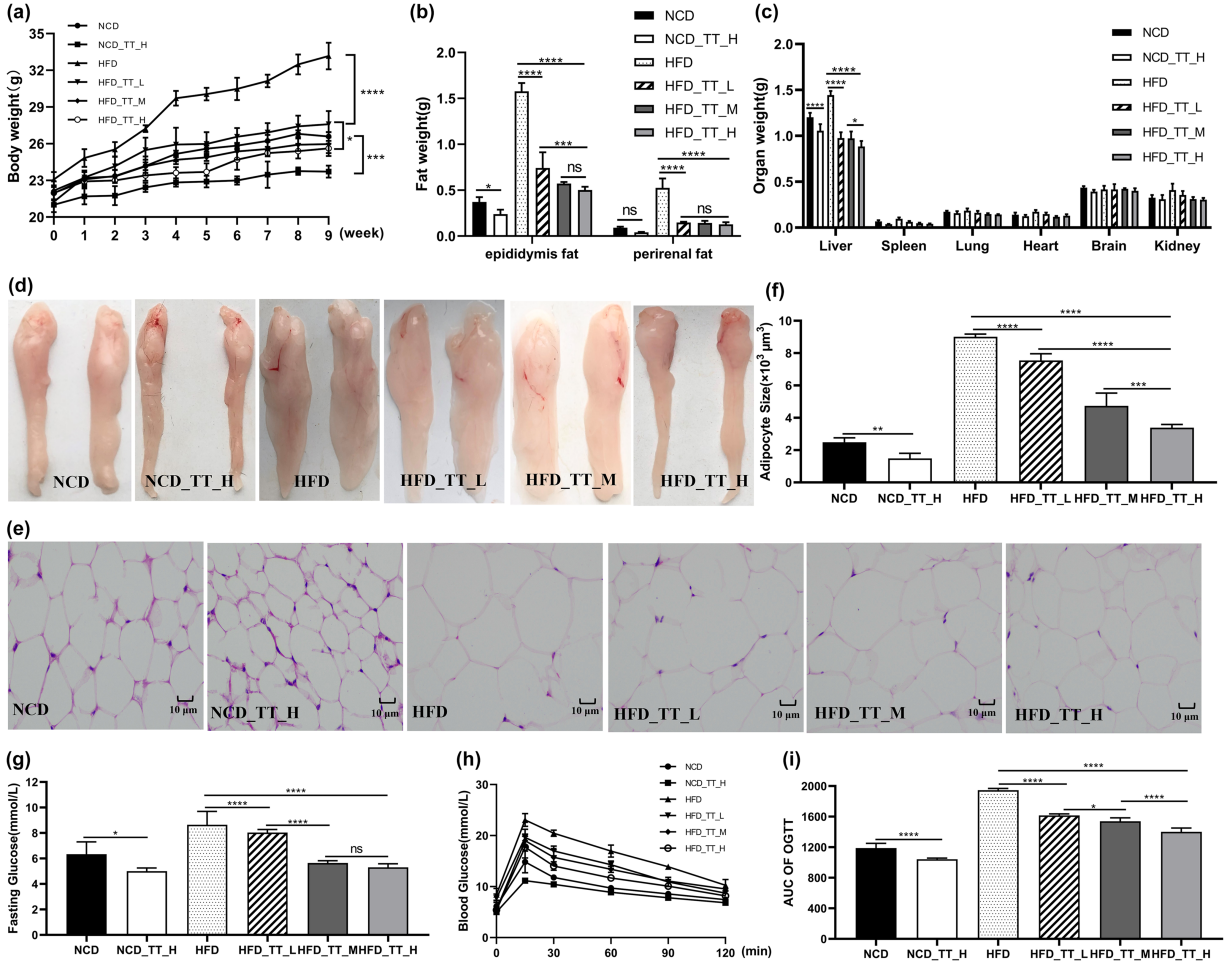
Tibetan tea attenuated high‐fat diet‐induced obesity in mice. (a) Body weight. (b) Weight of epididymal fat and perirenal fat. (c) Weight of different organs. (d) Morphology of the epididymis fat. (e) Morphology of adipocytes. (f) Epididymal adipocyte size. (g) Fasting glucose. (h) Oral glucose tolerance test. (i) Area under the curve of oral glucose tolerance test. Differences between groups were assessed using a one‐way ANOVA (ns for *p* > .05, *p < .05, **p < .01, ***p < .001 and ****p < .0001).

The HFD mice were noticeably heavier than the other five groups after 9 weeks, as shown in Figure 1. After TT intervention, the weight of mice was significantly reduced. Interestingly, TT inhibited body weight in mice in the form of dose dependence (Figure 1a). The weights of various organs (Figure 1c) and the weights of mice's perirenal and epididymal fat (Figure 1b) show the beneficial effects of TT in the treatment of obesity. According to morphological analysis, TT effectively prevented HFD‐induced fat formation (Figure 1d) and adipocyte growth (Figure 1e,f) (*p* < .05).

### 3.3 TT helps obese mice with their blood lipid and glucose metabolic disorders

Blood lipids and glucose levels are likely to change as obesity progresses. We performed a glucose tolerance test and determined the AUC values in the trial's eighth week (Figure 1h,i). According to the findings, between 15 and 120 min, the blood glucose levels of the mice given the HFD were greater than those of the animals fed the NCD. Additionally, compared to the *HFD* group, the mice's blood glucose levels were lower after TT intervention. The impact of TT on the serum biochemical parameters in mice is shown in Figure 1g and Table 2. We found that the HFD considerably reduced HDL‐C and significantly raised fasting hyperglycemia, TC, TG, and LDL‐C in mice. Feeding TT significantly reversed these trends (*p* < .05).

**TABLE 2:** Effects of TT on metabolic syndrome in HFD‐fed mice.

Parameters	NCD	NCD_TT_H	HFD	HFD_TT_L	HFD_TT_M	HFD_TT_H
TC (mmol/L)	3.12 ± 0.14^e^	2.37 ± 0.16^f^	5.02 ± 0.07^a^	4.68 ± 0.01^b^	4.29 ± 0.22^c^	3.79 ± 0.11^d^
TG (mmol/L)	0.73 ± 0.04^c^	0.54 ± 0.022^f^	1.20 ± 0.02^a^	0.99 ± 0.04^b^	0.65 ± 0.03^d^	0.58 ± 0.02^e^
LDL‐C (mmol/L)	1.45 ± 0.18^c^	0.57 ± 0.03^d^	2.56 ± 0.20^a^	1.87 ± 0.24^b^	0.69 ± 0.10^d^	0.52 ± 0.03^d^
HDL‐C (mmol/L)	3.66 ± 0.05^c^	5.28 ± 0.54^a^	2.72 ± 0.05^e^	3.12 ± 0.05^d^	4.20 ± 0.05^b^	5.51 ± 0.14^a^

*Note*: Significant differences were evaluated by one‐way analysis of variance (ANOVA). Comparison of each parameter between the six groups, with different superscript letters indicating significant differences (*p* < .05).

Abbreviations: HFD, high‐fat diet; HFD_TT_H, high‐fat diet with 400 mg/kg of TT extract; HFD_TT_L, high‐fat diet with 100 mg/kg of TT extract; HFD_TT_M, high‐fat diet with 200 mg/kg of TT extract; NCD, normal chow diet; NCD_TT_H, normal chow diet with 400 mg/kg of TT extract.

### 3.4 TT ameliorates HFD‐induced gut microbial disturbance in mice

**FIGURE 2:**
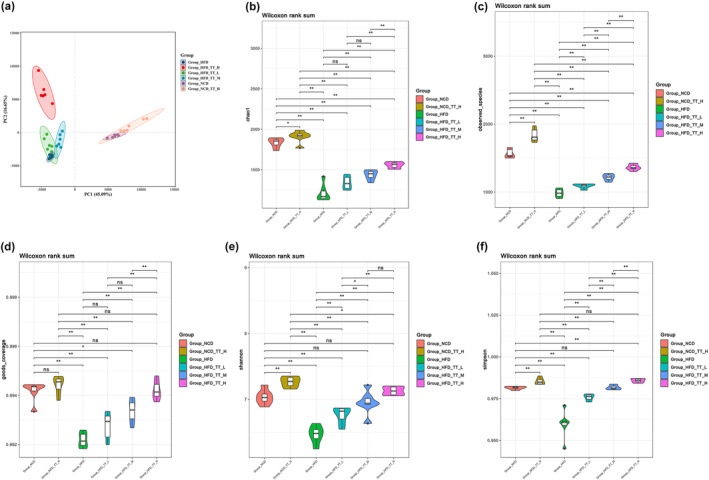
Tibetan tea modulated the structure and diversity of the fecal microbiota. (a) principal components analysis plot. Alpha Diversity‐related boxplot analysis, including (b) chao1, (c) observed_species, (d) goods_coverage, (e) Shannon index, (f) Simpson index. Differences between groups were assessed using a one‐way ANOVA (ns for *p* > .05, *p < .05, **p < .01, ***p < .001 and ****p < .0001).

We amplified the bacterial 16S rRNA gene's V3‐V4 region and sequenced it using the Illumina MiSeq technology in order to evaluate the cumulative TT‐induced alterations in the gut microbiota. According to principal components analysis (PCoA) (Figure 2a), the microbial community composition across the groups seemed to be clustered across the groups. The difference in PCoA1 was 45.09%, principally reflecting the impact of normal and high‐fat diets on the gut microbiota structure; in contrast, the difference in PCoA2's vertical coordinate was 16.65%, primarily highlighting the impact of TT on the gut microbiota. The *HFD* group had the lowest chao1 values, observed‐species values, and goods coverage values compared to the other five groups, according to the alpha diversity analysis (Figure 2b–d), and these three values increased considerably after feeding TT. Figure 2e,f demonstrates this. The Shannon and Simpson index grew considerably after TT's involvement. These findings imply that HFD reduces the variety of the gut microbiota in mice. By boosting variety and abundance, feeding TT, on the other hand, greatly improved the gut microbiota structure.

**FIGURE 3:**
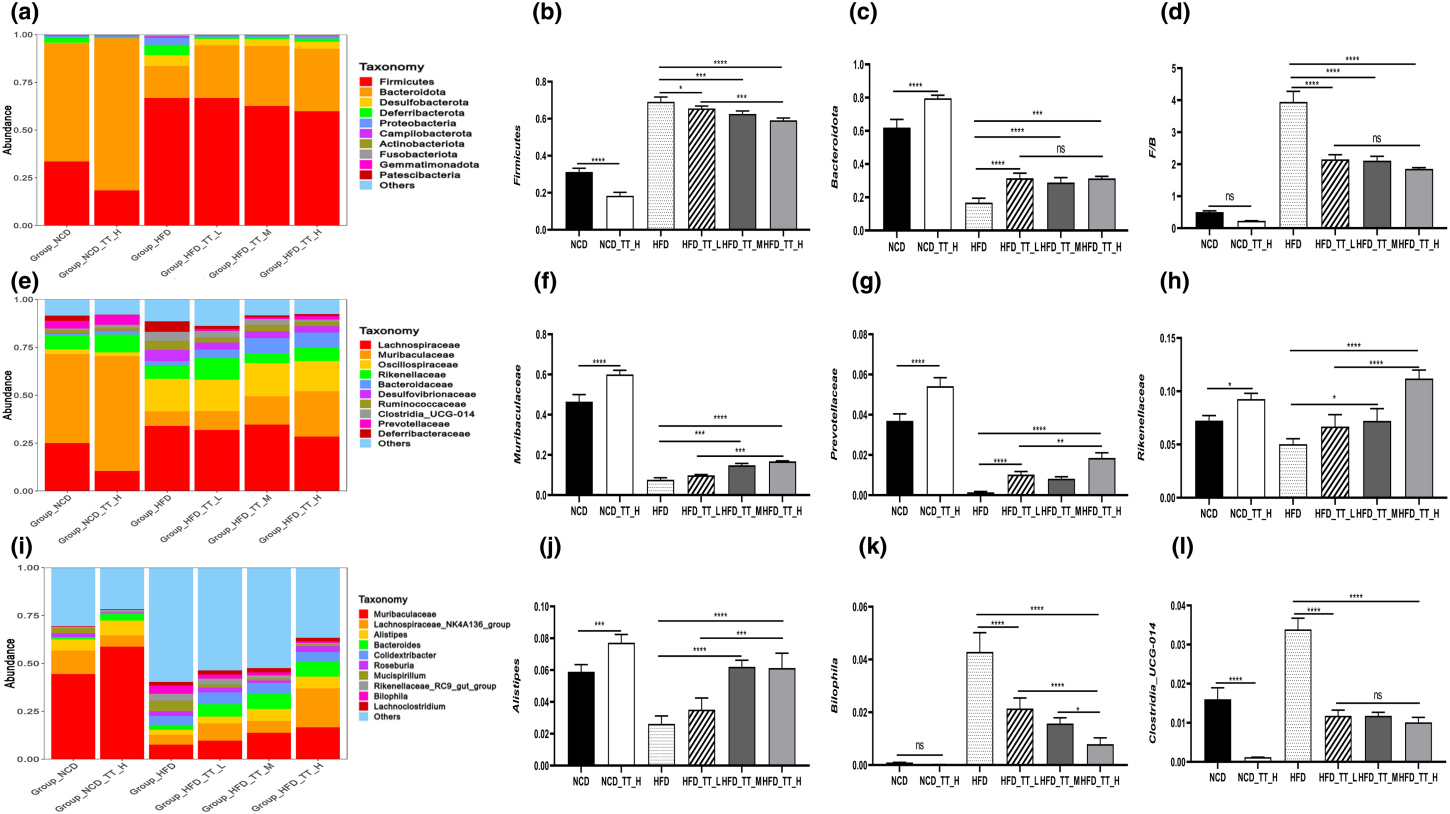
Tibetan tea modulated the structure of the fecal microbiota in mice. Phylum‐(a), family‐(e), and genus‐level (i) distribution of fecal microbiota. Relative abundance of the phyla *Firmicutes* (b) and *Bacteroidota* (c); (d) Relative population abundance ratio of *Firmicutes* and *Bacteroidota*; (f–h, j–l) relative abundance of some microorganisms at the family and genus levels. Differences between groups were assessed using a one‐way ANOVA (ns for *p* > .05, *p < .05, **p < .01, ***p < .001, and ****p < .0001).

Comparing the relative prevalence of the key microbial groups in the gut microbiota of six food groups allowed researchers to understand better how TT affects the regulation of the gut microbiota. *Bacteroidota*, *Firmicutes*, *Deferribacterota*, *Desulfobacterota*, and *Proteobacteria* were among the prominent species identified in histograms showing the relative abundance of gut microbiomes at the phylum level (Figure 3a). With more than 90% of the total, the two main phyla are Bacteroidota and Firmicutes. By increasing the number of *Firmicutes* (Figure 3b) and reducing the quantity of *Bacteroidota* (Figure 3c), the *HFD* group outperformed the other five groups in terms of *F/B* (Figure 3d). The gut microbiota's final makeup was changed.

The most numerous bacteria were those belonging to the families *Muribaculaceae*, *Lachnospiraceae*, *Rikenellaceae*, *Prevotellaceae*, *Deferribacteraceae*, and *Oscillospiraceae* (Figure 3e). The TT intervention substantially reduced the population abundance of *Clostridia UCG‐014* and *Desulfovibrionaceae* (Figure 3e) and increased the population abundance of *Muribaculaceae* (Figure 3f), *Prevotellaceae* (Figure 3g), and *Rikenellaceae* (Figure 3h) as compared to the *HFD* group. After TT therapy, we examined the genus‐level alterations in the gut microbiota, and the findings revealed variations across all experimental groups. The *HFD* group had lower relative population abundances of the *Muribaculaceae* (Figure 3i), *Lachnospiraceae_NK4A136_group* (Figure 3i), *Alistipes* (Figure 3j), *Bacteroides* (Figure 3i), and *Colidextribacter* (Figure 3i) than the *NCD* and TT intervention groups. While *Bilophila* (Figure 3k), *Clostridia_UCG‐014* (Figure 3l), *Mucispirillum* (Figure S1A), and *Blautia* (Figure S1B) had greater relative population abundances. These findings imply that animals on a high‐fat diet had changed gut microbiotas and that TT intervention corrected these abnormalities.

### 3.5 TT's impact on the SCFAs

The impact of TT on the level of SCFAs in mouse feces is shown in Table 3. The amount of fecal SCFAs in the *HFD* group was much lower than that of the other five groups. The contents of butyric acid, propionic acid, and acetic acid in the mouse feces of the *NCD_TT_H* group were substantially greater than those of the *NCD* group (*p* < .05). After TT intervention, animals had substantially increased amounts of butyric acid, propionic acid, and acetic acid in their feces compared to the *HFD* group. Similar to this, TT significantly altered branched‐chain fatty acids (BCFAs), mostly by increasing the quantity of isobutyric acid; however, isovaleric acid did not change.

**TABLE 3:** Fecal SCFAs contents of mice.

SCFAs (μg/mg)	NCD	NCD_TT_H	HFD	HFD_TT_L	HFD_TT_M	HFD_TT_H
Acetic acid	0.87 ± 0.06^b^	1.07 ± 0.12^a^	0.53 ± 0.01^e^	0.57 ± 0.01^de^	0.63 ± 0.07^cd^	0.70 ± 0.04^c^
Propionic acid	0.36 ± 0.07^b^	0.66 ± 0.11^a^	0.08 ± 0.00^d^	0.11 ± 0.01^d^	0.14 ± 0.02^cd^	0.20 ± 0.03^c^
Isobutyric acid	0.18 ± 0.02^b^	0.22 ± 0.01^a^	0.05 ± 0.00^f^	0.08 ± 0.01^e^	0.11 ± 0.02^d^	0.16 ± 0.02^c^
Butyric acid	0.29 ± 0.02^b^	0.43 ± 0.07^a^	0.06 ± 0.00^d^	0.06 ± 0.00^d^	0.08 ± 0.00^cd^	0.10 ± 0.01^c^
Isovaleric acid	0.05 ± 0.01^a^	0.04 ± 0.01^a^	0.04 ± 0.01^a^	0.05 ± 0.01^a^	0.06 ± 0.00^a^	0.05 ± 0.01^a^
Valeric acid	0.02 ± 0.01^a^	0.02 ± 0.01^a^	0.02 ± 0.01^a^	0.02 ± 0.00^a^	0.02 ± 0.01^a^	0.03 ± 0.01^a^

*Note*: Data are expressed as mean ± SD (*n* = 6). Significant differences were evaluated by one‐way analysis of variance (ANOVA). Comparison of each parameter between the six groups, with different superscript letters indicating significant differences (*p* < .05).

### 3.6 TT regulates the expression of genes associated with obesity

**FIGURE 4:**
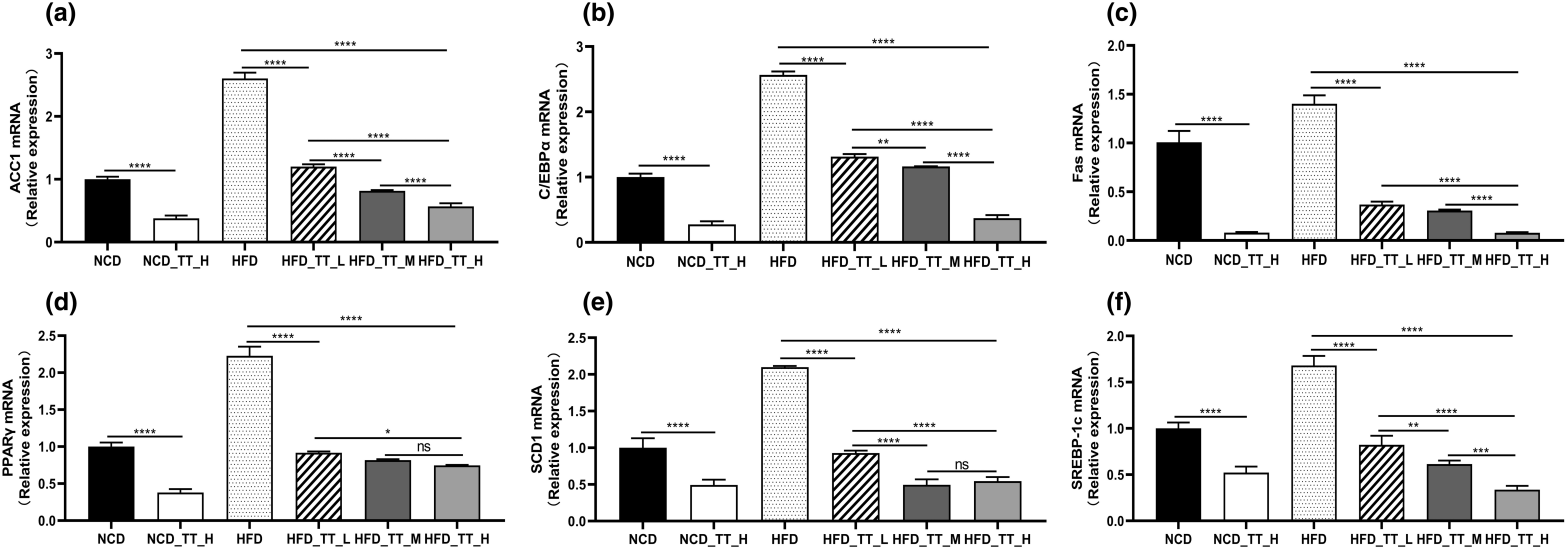
Tibetan tea regulated the expression of genes related to lipid synthesis. Relative expression of (a) ACC1, (b) C/EBPα, (c) Fas, (d) PPARγ, (e) SCD1, and (f) SREBP‐1c in the epididymal fat compared to the *NCD* group. Differences between groups were assessed using a one‐way ANOVA (ns for *p* > .05, *p < .05, **p < .01, ***p < .001, and ****p < .0001).

Figure 4 displays the lipid synthesis‐related genes' levels of gene expression. When compared to the *NCD* group, the *NCD_TT_H* group's expression of ACC1, C/EBPα, Fas, PPARγ, SCD1, and SREBP‐1c was considerably lower (*p* < .05). The expression of six genes was also significantly lower in the three TT intervention groups than in the *HFD* group (*p* < .05). Interestingly, in comparison to the *HFD* group, there was a dose‐dependent reduction in the expression of ACC1, C/EBPα, Fas, and SREBP‐1c (Figure 4a–c,f).

### 3.7 Transplantation of feces microbiota prevents obesity and improves lipid metabolism in mice

**FIGURE 5:**
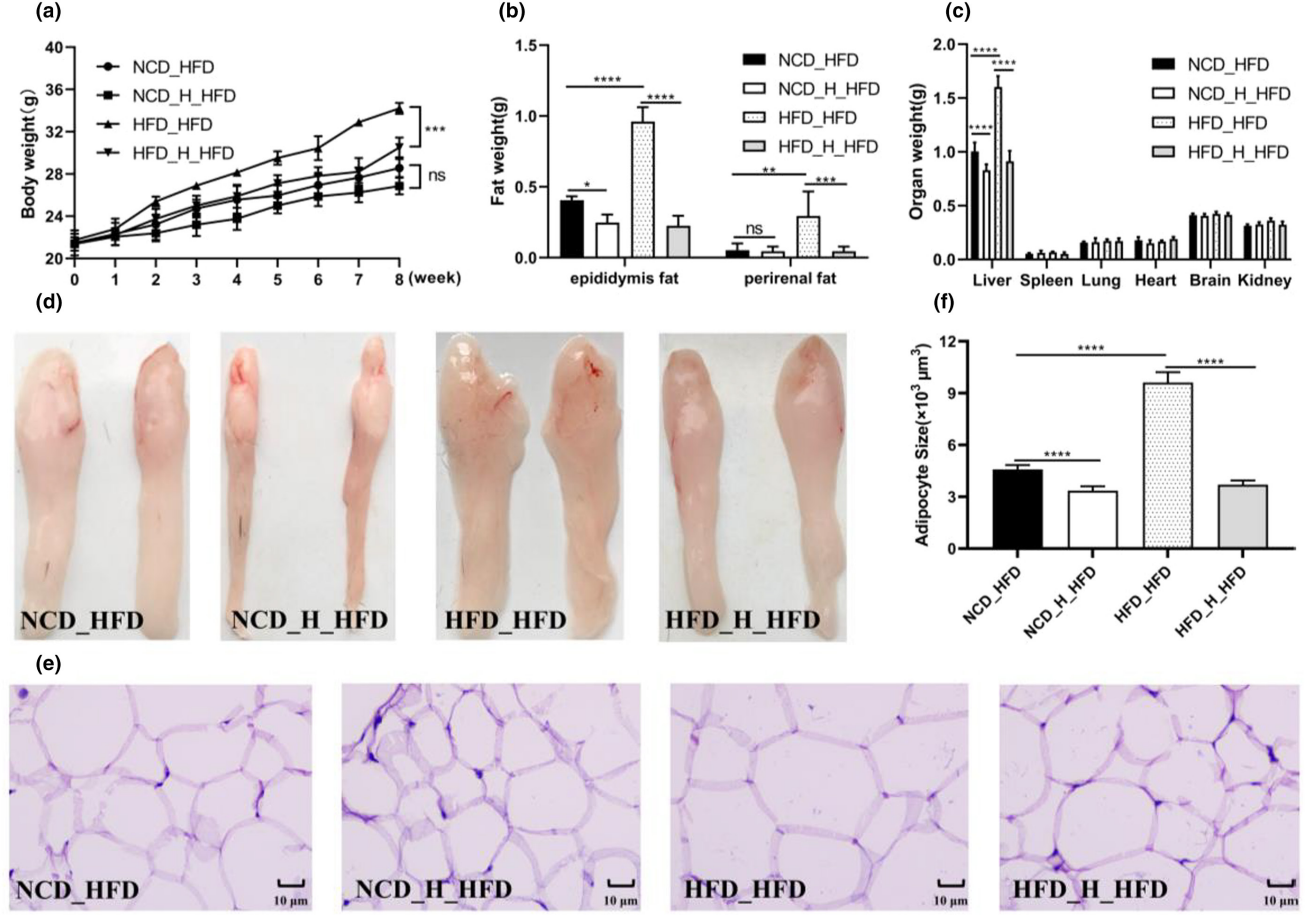
FMT attenuated fat accumulation in obese mice. (a) Body weight. (b) Epididymal fat and perirenal fat weight. (c) Weight of different organs. (d) Morphological observations of the epididymis fat. (e) Hematoxylin and eosin staining of epididymis fat. (f) Epididymal adipocyte size. Differences between groups were assessed using a one‐way ANOVA (ns for *p* > .05, *p < .05, **p < .01, ***p < .001 and ****p < .0001).

To study the impact of TT on the gut microorganisms of obese mice, we transplanted feces from four donor mouse groups (*NCD*, *NCD_TT_H*, *HFD*, *HFD_TT_ H*) into HFD‐fed animals. Then, we examined obesity‐related characteristics and metabolic alterations in glucose and lipids levels. The findings demonstrated that fecal transplantation from the *NCD* group, *NCD_TT_H* group, and *HFD_TT_H* group decreased the body weight (Figure 5a), liver weight (Figure 5c), fat accumulation (Figure 5b,d), and adipocyte growth (Figure 5e,f) of recipient mice in comparison to the *HFD* group. Similar to Table 2, Table 4 depicts the variations in blood glucose and cholesterol levels in mice. The *HFD_HFD* group showed substantially greater fasting glucose, TC, TG, and LDL‐C levels and higher HDL‐C levels than the other three groups.

**TABLE 4:** The effect of fecal transplantation on metabolic syndrome in HFD‐fed mice.

Parameters	NCD_HFD	NCD_H_HFD	HFD_HFD	HFD_H_HFD
Fasting glucose (mmol/L)	7.87 ± 0.47^b^	6.86 ± 0.50^c^	9.10 ± 0.07^a^	6.78 ± 0.60^c^
TC (mmol/L)	4.50 ± 0.14^b^	1.56 ± 0.10^c^	5.63 ± 1.20^a^	2.01 ± 0.13^c^
TG (mmol/L)	1.21 ± 0.10^b^	0.39 ± 0.02^d^	2.07 ± 0.12^a^	0.85 ± 0.09^c^
LDL‐C (mmol/L)	1.33 ± 0.20^b^	0.59 ± 0.06^d^	2.31 ± 0.06^a^	0.93 ± 0.05^c^
HDL‐C (mmol/L)	3.84 ± 0.07^b^	5.45 ± 0.07^a^	2.07 ± 0.06^c^	3.94 ± 0.10^b^

*Note*: Data are expressed as mean ± SD (*n* = 6). Significant differences were evaluated by one‐way analysis of variance (ANOVA). Comparison of each parameter between the four groups, with different superscript letters indicating significant differences (*p* < .05).

### 3.8 Fecal microbiota transplantation alters the makeup of the gut microbiota in obese mice

**FIGURE 6:**
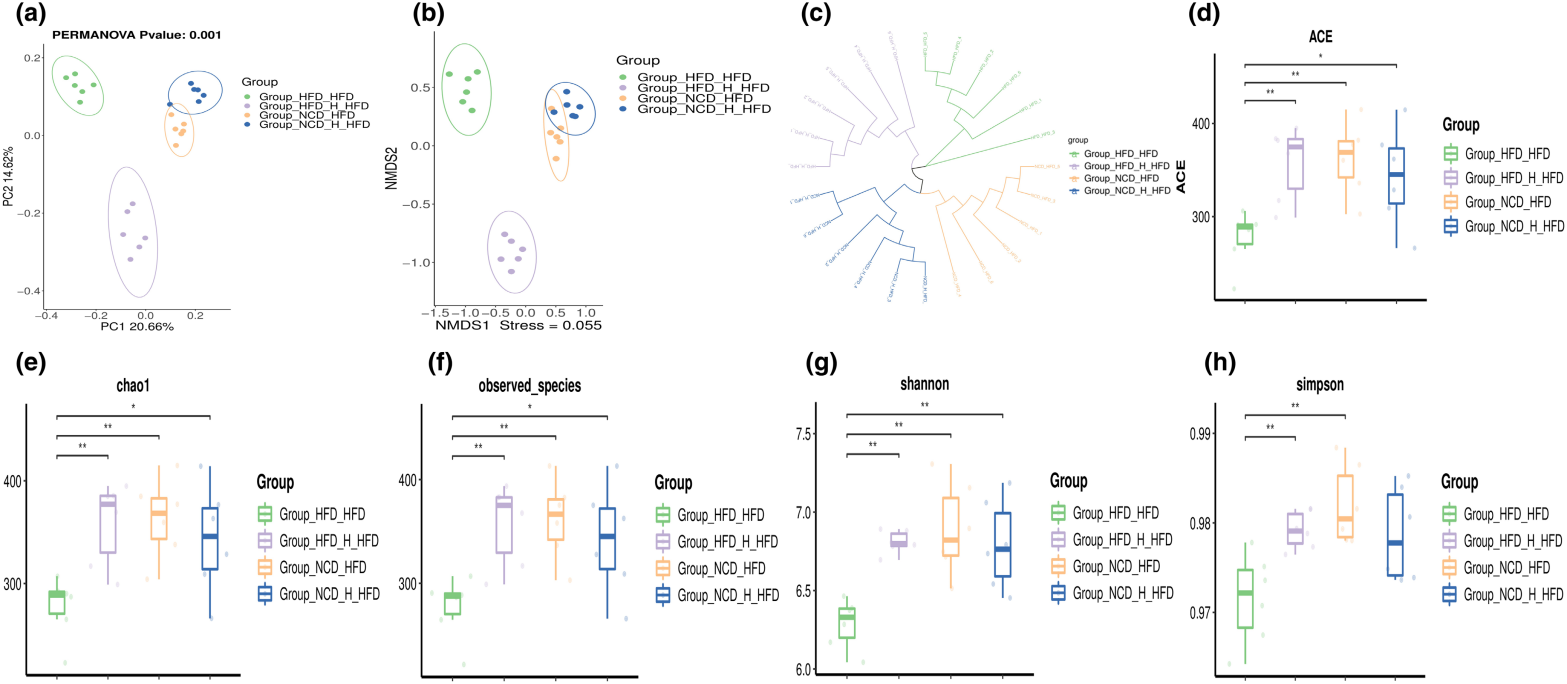
FMT modulated the diversity and structure of the fecal microbiota in mice. Beta diversity analysis includes (a) PCoA, (b) non‐metric multidimensional scaling (NMDS) analysis, and (c) hierarchical clustering. Alpha diversity‐related boxplot analysis includes(d) ACE, (e) Chao 1, (f) Observed‐species, (g) Shannon index, (h) Simpson index. Differences between groups were assessed using a one‐way ANOVA (ns for *p* > .05, *p < .05, **p < .01, ***p < .001, and ****p < .0001).

We examined the microbial composition of the guts of four groups of mice that had their fecal microbiota transplanted in order to investigate the regulatory effects of TT on gut microorganisms. PCoA, NMDS, and the Unweighted Pair Group Method with Arithmetic Mean (UPGMA) were used in the investigation of the diversity. According to PCoA, there were significant differences between the intestinal microbiotas of the four mouse groups (Figure 6a). For PC1 and PC2, these differences were 20.66% and 14.62%, respectively, with the *NCD_ H_HFD* and *NCD_ HFD* groups being distributed in the right quadrant and the *HFD_ H_HFD* and *HFD_ HFD* groups in the left quadrant. Similar findings from the NMDS analysis were obtained (Figure 6b), and the UPGMA sample hierarchical cluster analysis revealed that each of the four groups was grouped into a single cluster (Figure 6c). The alpha diversity among the four groups also varied significantly in a noticeable way. In comparison to the other three groups, *HFD_HFD* had lower values for ACE, Chao 1, species richness, Shannon, and Simpson indices (Figure 6d–h). After transplanting mouse feces fed with TT, however, all of these values rose.

**FIGURE 7:**
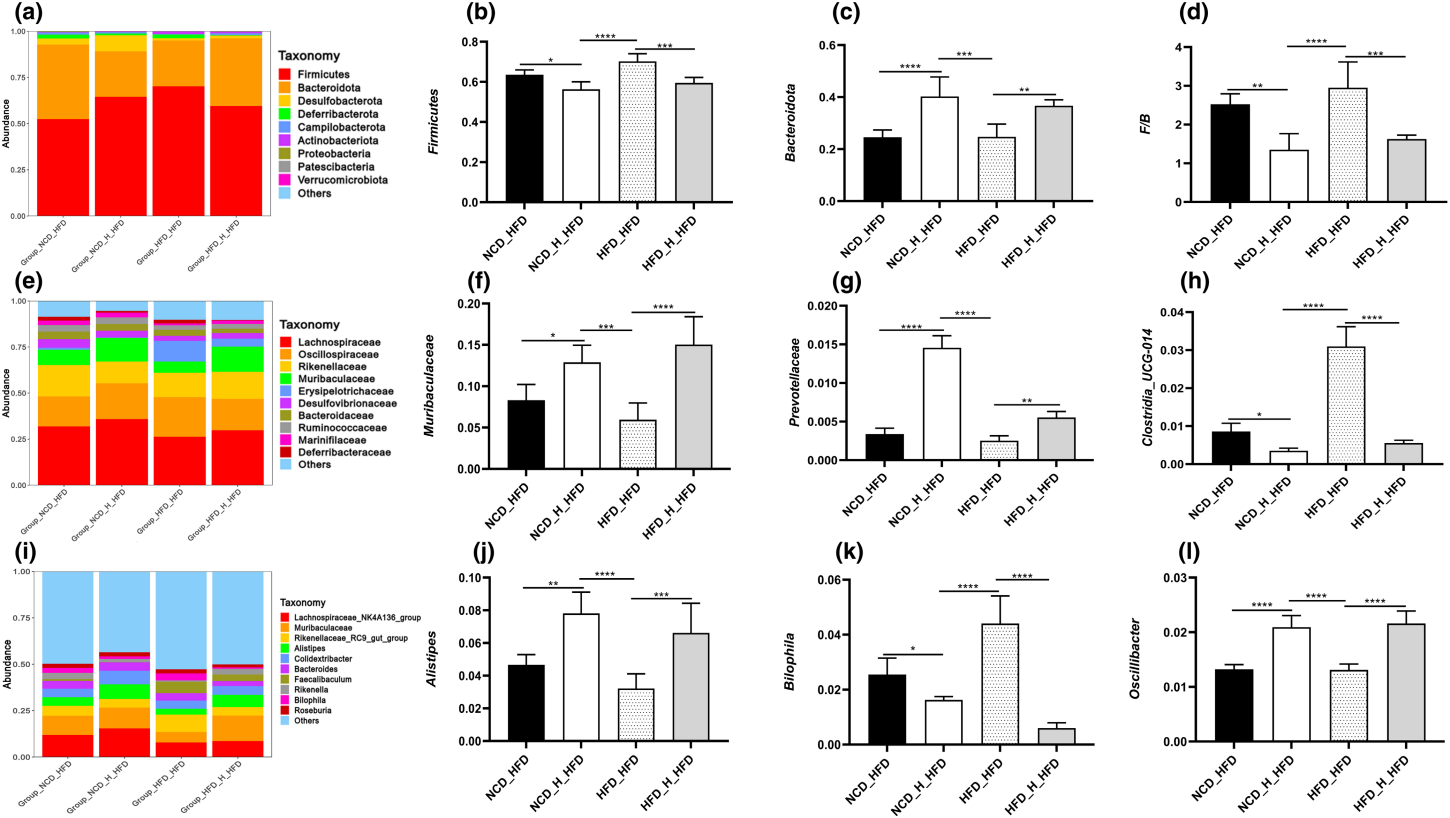
Fecal microbiota transplantation regulated the structure of the fecal microbiota. Phylum‐(a), family‐(e), and genus‐level (i) distribution of fecal microbiota. Relative abundance of the phyla *Firmicutes* (b) and *Bacteroidota* (c); (d) Relative population abundance ratio of *Firmicutes* and *Bacteroidota*; (f–h, j–l) relative abundance of some microorganisms at the family and genus levels. Differences between groups were assessed using a one‐way ANOVA (ns for *p* > .05, *p < .05, **p < .01, ***p < .001, and ****p < .0001).

We analyzed the microbial makeup of four groups to further look into the particular gut microbiota alterations at various taxonomic levels. Similar to the earlier findings, the *HFD_HFD* group changed the makeup of the gut microbiota at the phylum level by raising the *F/B* ratio, which fell following the transplantation of mouse feces from TT‐fed animals (Figure 7a–d). Additionally, there were notable changes between the four groups' microbial compositions at the family level (Figure 7e). Transplantation of TT‐fed mouse feces substantially enhanced the relative population abundance of *Lachnospiraceae* (Figure 7e), *Muribaculaceae* (Figure 7f), *Ruminococcaceae* (Figure 7e), and *Prevotellaceae* (Figure 7g) compared to the *HFD_HFD* group, whereas *Clostridia_UCG‐014* (Figure 7h) and *Deferribacteraceae* decreased in relative population abundance. In all experimental groups, changes in gut microbiota were seen at the genus level. In comparison to the *HFD_HFD* group, the relative population abundance of the *Lachnospiraceae_NK4A136_group* (Figure S1C), *Colidextribacter* (Figure S1D), *Alistipes* (Figure 7j), and *Oscillibacter* (Figure 7l) was greater in both the *NCD_H_HFD* group and the *HFD_H_HFD* group, whereas the *Bilophila* (Figure 7k) was lower. These findings revealed that an *HFD* changed the microbial composition of mice's guts. However, the gut microbial diversity and disordered intestinal microorganisms were enhanced when mice fed TT's feces were transplanted.

### 3.9 Association of gut microbiota with obesity‐related parameters, SCFAs, and genes related to lipid synthesis

**FIGURE 8:**
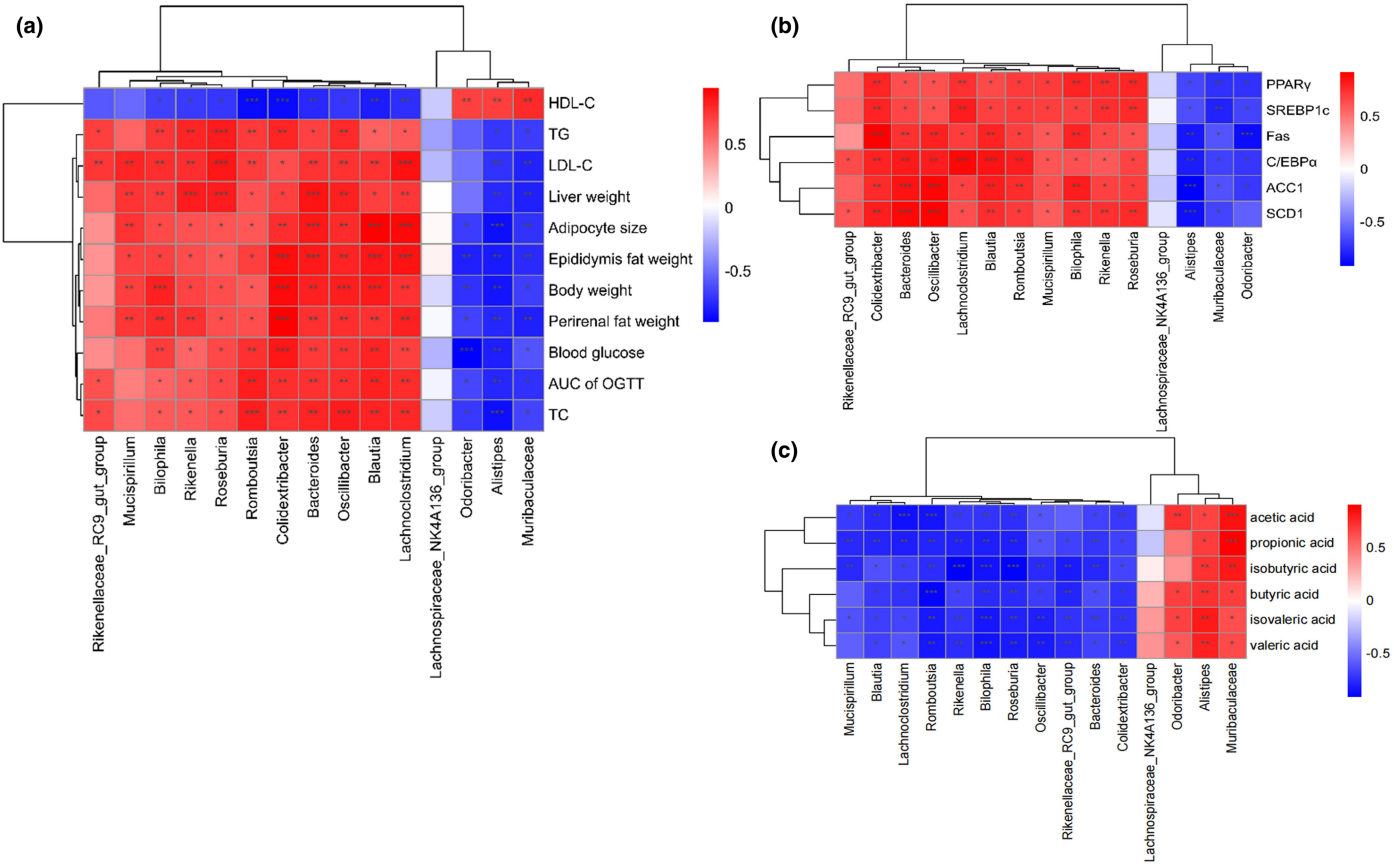
Heatmap of Spearman's correlation between gut microbiota relative abundances and obesity‐related indexes, SCFAs, and genes related to lipid synthesis in mice altered by HFD or TT intervention. (a), (b), and (c) represent the correlation analysis between obesity‐related indexes, SCFAs and genes related to lipid synthesis, and the top 15 most abundant genera of gut microbes, respectively. The red and blue blocks represent positive and negative correlations, respectively. Significant correlations are presented as *p < .05, **p < .01, and ***p < .001 (*n* = 6).

We used Spearman's correlation analysis to determine the gut microbiota that predominated and may have influenced how well TT prevented obesity caused by the HFD. The heatmap in Figure 8 demonstrated a positive or negative correlation between the top 15 most prevalent genera and factors associated with obesity, the presence of SCFAs, and genes involved in lipid synthesis. As shown in Figure 8a, *Lachnospiraceae_NK4A136_group*, *Odoribacter*, *Alistipes*, and *Muribaculaceae* exhibited a positive correlation with HDL‐C and a negative correlation with other obesity‐related parameters, like blood glucose, body weight TC, and TG; *Colidextribacter*, *Blautia*, and *Bilophila* showed the opposite trend compared to the gut mentioned above microbes. Interestingly, *Lachnospiraceae_NK4A136_group*, *Odoribacter*, *Alistipes*, and *Muribaculaceae* also showed a negative correlation with the PPARγ, SREBP1c, Fas, C/EBPα, ACC1, and SCD1 expressions, while the remaining 11 gut microbes showed a positive correlation (Figure 8b). *Lachnospiraceae_NK4A136_group*, *Odoribacter*, *Alistipes*, and *Muribaculaceae* showed a clear positive link exists between the concentrations of SCFAs and BCFAs in feces (Figure 8c).

## 4 DISCUSSION

As an epidemic, obesity is related to many cardiovascular and metabolic diseases, even cancer (Bagnall et al., 2019). Statistics show that each year, almost 2.8 million individuals pass away due to obesity‐related illnesses (Wiciński et al., 2020), making the need for obesity treatment and prevention urgent. According to several researches, drinking dark tea may help prevent and cure obesity (Lim et al., 2022; Wang, Hu, et al., 2022). There have been claims that the dark tea variety TT offers anti‐obesity properties (Yuan et al., 2016). Its anti‐obesity properties and influence on gut microorganisms have an unknown underlying mechanism. The present study explored how TT inhibited HFD‐induced obesity in mice.

In mice, a high‐fat diet was shown to increase weight growth and fat storage, whereas TT therapy was found to reduce these tendencies. Additionally, we discovered that, in line with several researches, feeding TT to obese mice decreased fasting hyperglycemia, TC, TG, and LDL‐C and elevated HDL‐C. In mice with obesity brought on by the HFD, liupao tea extract was utilized to reduce body weight via altering oxidative stress and lipid metabolism (Wu et al., 2021). Pu‐erh tea decreased obesity in mice via modifying the gut microbiome (Ye et al., 2021). Our findings amply supported the notion that TT suppresses HFD‐induced obesity in mice, controls glucose and lipid metabolism, and is helpful in the management of obesity.

Numerous researches in recent years have proven that the gut microbiota is essential for controlling the host's lipid and glucose metabolism (Chen et al., 2018; Liu, Liu, et al., 2021; Wang & Jia, 2016). As a result, one possible target for the treatment of obesity is the gut flora. In the current research, the gut bacteria in the HFD group of mice saw a considerable alteration in structure and composition. Obese mice's disturbed gut microbes were improved after receiving TT therapy, mostly in the form of enhanced gut microbiota diversity and abundance. Furthermore, the *F/B* ratio is one trait of the gut microbiota of obese mice (Abenavoli et al., 2019; Stojanov et al., 2020). The *HFD* group in the current research had the highest *F/B* values, which were dramatically decreased by the TT therapy. In compared to the *HFD* group, the TT treatment enhanced the relative population abundance of the families *Muribaculaceae*, *Prevotellaceae*, *Rikenellaceae*, and *Bacteroidaceae* while decreasing the relative population abundance of the families *Clostridia_UCG‐014* and *Desulfovibrionaceae*. Less of the genus related to controlling obesity, such as *Muribaculaceae*, *Prevotellaceae*, and *Rikenellaceae*, were present in the fat lab animals (Shen et al., 2017; Zhao et al., 2022; Ziętak et al., 2016). According to studies, *Clostridia_UCG‐014* is more prevalent and favorably linked with blood glucose in obesity models (Koontanatechanon et al., 2022).

Similarly, *Desulfovibrionaceae* was found to be increased in abundance due to a high‐fat diet (Zhang et al., 2010). At the genus level, the relative population abundances of *Bilophila*, *Blautia*, *Clostridia_UCG‐014*, and *Mucispirillum* were greater in the *HFD* group than those of *Alistipes*, *Muribaculaceae*, *Lachnospiraceae_NK4A136_group*, *Bacteroides*, and *Colidextribacter*. According to reports, Colidextribacter may release inosine, possibly reducing acute liver damage and inflammation brought on by LPS (Guo et al., 2021). On the other hand, the lipopolysaccharide‐producing bacteria *Bilophila* have been linked to worsening inflammation, and metabolic abnormalities brought on by HFD in mice (Lu et al., 2021). With Spearman correlation analysis, we found that Lachnospiraceae_NK4A136_group, Odoribacter, Alistipes, and Muribaculaceae were inversely linked with the majority of obesity‐related variables, revealing that these gut microbes may play a role as beneficial microbes (Figure 8a).

The anti‐obesity properties of TT were transmitted to mice fed an HFD via fecal microbiota transplantation studies. We found that recipient mice gained less weight after receiving transplants of TT‐fed mouse feces and that fat storage and adipocyte growth were suppressed. The blood concentrations of TC, TG, LDL‐C, and HDL‐C in the other three mouse groups were also lower than those in the *HFD_HFD* group. It is interesting to note that FMT had a significant impact on obese mice's gut flora as well. The key symptoms were the gut microbiota's altered diversity, composition, and structure. By changing *F/B* values, FMT modified the phylum‐level composition of the gut microbiota in obese mice. At the family and genus levels, the *NCD_H_HFD* and *HFD_H_HFD* groups demonstrated a more substantial prevalence of *Lachnospiraceae*, *Muribaculaceae*, *Ruminococcaceae*, *Prevotellaceae*, *Lachnospiraceae_NK4A136_group*, *Colidextribacter*, and *Alistipes*. However, there were fewer instances of *Clostridia_UCG‐014*, *Deferribacteraceae*, *Bilophila*, and *Blautia*. These findings showed that TT alters the gut microbiota of obese mice and that this alteration helps with weight reduction.

It has been reported that the fungi *Alistipes*, *Prevotellaceae*, and *Lachnospiraceae_NK4A136_group* generate SCFAs (David et al., 2014; Koh et al., 2016; Li, Zhao, et al., 2022; Wu et al., 2020). SCFAs, which are mostly composed of acetic, propionic, and butyric acids and are often formed by the fermentation of dietary polysaccharides by gut bacteria, play an important role in the prevention and treatment of obesity (Coppola et al., 2021; Zaky et al., 2021). The G protein‐coupled receptor GPR43, which is connected to calorie expenditure and lipid metabolism, is activated by SCFAs as endogenous signaling molecules (Schoeler & Caesar, 2019). Additionally, SCFAs enhance glucagon‐like peptide‐1 and gut hormone secretion, which decreases appetite and increases fullness in the body (Kimura et al., 2020). SCFAs may also maintain gut homeostasis by modulating the gut‐brain axis and preserving the integrity of the gut barrier (Silva et al., 2020; Zheng et al., 2017). Through the use of GC–MS, we were able to ascertain the amount of SCFAs present in mouse feces and discovered that the TT treatment dramatically boosted the synthesis of SCFAs, namely acetic, propionic, and butyric acids. It was shown that TT could also regulate lipid metabolism and maintain gut homeostasis by increasing the production of SCFAs, thus effectively preventing HFD‐induced obesity.

Obesity is characterized by an increase in adipose tissue weight brought on by an increase in the quantity and size of adipocytes (Yang & Kim, 2015). Therefore, preventing the development of obesity and the problems is connected with lowering the creation and storage of fat. Our findings demonstrated that TT decreased the weight increase and adipocyte growth brought on by the HFD. We also discovered that TT therapy decreased the expression of the genes ACC1, C/EBPα, Fas, PPARγ, SCD1, and SREBP‐1c in the mice's adipose tissue. The fatty acid production pathway's rate‐limiting enzyme is known as ACC1 (Wang, Yu, et al., 2022). PPARγ has been shown to control the expression of genes associated with adipose tissue and to encourage lipogenesis (Lee et al., 2018). C/EBPα is a crucial control point for adipocyte differentiation and PPARγ‐induced lipogenesis (Lee et al., 2019). SREBP‐1c is an essential transcription factor that controls the expression of the genes involved in the fatty acid synthesis and activates the adipogenic transcription factors ACC‐1, Fas, and SCD1, which control the formation of adipose tissue and the accumulation of lipids (Fang et al., 2019; Linden et al., 2018; Zhu et al., 2019).

## 5 CONCLUSIONS

As a result of our research, we were able to demonstrate that TT dramatically inhibited HFD‐induced weight gain, fat accumulation, hyperglycemia, and hyperlipidemia in mice, as well as control the expression of genes involved in lipid metabolism. The way that TT controls the gut microbiota produces SCFAs and controls the expression of genes involved in lipid synthesis may be responsible for its positive effects on obesity. Our research gave TT's potential as a functional beverage for preventing and treating obesity a new direction.

## AUTHOR CONTRIBUTIONS

**Gang He:** Conceptualization (lead); data curation (supporting); formal analysis (lead); funding acquisition (lead); investigation (supporting); methodology (lead); project administration (lead); resources (lead); supervision (lead); validation (equal); visualization (equal); writing – original draft (equal); writing – review and editing (lead). **Tangcong Chen:** Conceptualization (lead); data curation (lead); formal analysis (lead); investigation (lead); methodology (lead); supervision (lead); validation (lead); visualization (lead); writing – original draft (lead); writing – review and editing (lead). **Lifen Huang:** Conceptualization (equal); data curation (equal); formal analysis (equal); investigation (equal); supervision (equal); validation (equal); visualization (equal); writing – original draft (supporting); writing – review and editing (supporting). **Yiyuan Zhang:** Conceptualization (supporting); data curation (equal); formal analysis (supporting); investigation (supporting); methodology (supporting); validation (supporting); visualization (supporting). **Yanjiao Feng:** Data curation (supporting); formal analysis (supporting); investigation (supporting); methodology (supporting); validation (supporting); visualization (supporting); writing – original draft (supporting). **Qijun Liu:** Formal analysis (supporting); investigation (lead); methodology (supporting); visualization (supporting); writing – original draft (supporting). **Xiaojing Yin:** Data curation (supporting); formal analysis (supporting); investigation (supporting); validation (supporting); visualization (supporting); writing – original draft (supporting). **Shaokui Qu:** Conceptualization (supporting); data curation (supporting); formal analysis (supporting); investigation (supporting); validation (supporting); visualization (supporting); writing – original draft (supporting). **Chen Yang:** Conceptualization (supporting); data curation (supporting); formal analysis (supporting); investigation (supporting); methodology (supporting); resources (supporting). **Jianghong Wan:** Formal analysis (equal); investigation (equal); methodology (equal). **Li liang:** Conceptualization (supporting); funding acquisition (equal); project administration (supporting); supervision (supporting). **Jun Yan:** Conceptualization (equal); formal analysis (equal); funding acquisition (lead); methodology (equal); project administration (lead); resources (equal); supervision (equal). **Wei Liu:** Conceptualization (equal); formal analysis (equal); funding acquisition (lead); project administration (lead); resources (equal); supervision (equal).

## CONFLICT OF INTEREST STATEMENT

The authors declare no conflicts of interest.

## ETHICS STATEMENTS

The Sichuan Industrial Institute of Antibiotics at Chengdu University's Protection of Laboratory Animals committee examined and approved the animal research (Chengdu, China; Approval Number: SIIA 20210706).

## Supporting information


Figure S1.

Table S1.

## Data Availability

The NCBI public database may be accessed at the following site. The raw readings were submitted there. https://www.ncbi.nlm.nih.gov/sra/PRJNA901348.
