# Supplementary material for: Tibetan tea reduces obesity brought on by a high‐fat diet and modulates gut flora in mice
Source: Food Sci Nutr. 2023 Aug 7;11(10):6582–95. doi: 10.1002/fsn3.3607 (PMC10563754; doi:10.1002/fsn3.3607)
Supplement: Supplementary file 1 — Figure S1. [file FSN3-11-6582-s002.docx]

**Supplementary Figure 1**. A, B, C and D are the relative population abundances of *Mucispirillum*, *Blautia*, *Lachnospiraceae_NK4A136_grou*p and *Colidextribacter* respectively. Differences between groups were assessed using a one-way ANOVA (ns for *P* >0.05, **P* <0.05, ***P* <0.01, ****P* <0.001 and *****P* <0.0001).
