# Supplementary material for: Tibetan tea reduces obesity brought on by a high‐fat diet and modulates gut flora in mice
Source: Food Sci Nutr. 2023 Aug 7;11(10):6582–95. doi: 10.1002/fsn3.3607 (PMC10563754; doi:10.1002/fsn3.3607)
Supplement: Supplementary file 2 — Table S1. [file FSN3-11-6582-s001.pdf]

Food Science & Nutrition
Open Access

Tibetan tea reduces obesity brought on by a high-fat diet and modulates gut flora in mice

Journal:	*Food Science & Nutrition*
Manuscript ID	FSN3-2023-01-0095.R2
Wiley - Manuscript type:	Original Article
Date Submitted by the Author:	15-Jul-2023
Complete List of Authors:	he, gang; Cheng Du University<br>Chen, Tangcong; Cheng Du University<br>Huang, Lifen; Cheng Du University<br>Zhang, Yiyuan; Cheng Du University<br>Fen, Yanjiao; Cheng Du University<br>Liu, Qijun; Cheng Du University<br>Yin, Xiaojing; Cheng Du University<br>Qu, Shaokui; Cheng Du University<br>Yang, Chen; Cheng Du University<br>Wan, Jianghong; Cheng Du University<br>liang, li; Cheng Du University<br>Yan, Jun; Cheng Du University<br>Liu, Wei; Cheng Du University
Search Terms:	food, nutrition, gut microbiota, obesity
Abstract:	It has been shown that Tibetan tea (TT) inhibits obesity and controls lipid metabolism. The fundamental processes by which TT prevents obesity are yet completely unknown. Consequently, the purpose of this research was to ascertain if TT may prevent obesity by modifying the gut flora. Our research demonstrated that TT prevented mice from gaining weight and accumulating fat due to the high-fat diet (HFD), decreased levels of blood total cholesterol (TC), triglycerides (TG), and low-density lipoprotein cholesterol (LDL-C), and raised levels of high-density lipoprotein cholesterol (HDL-C). Adipogenesis-related genes such as acetyl-CoA carboxylase 1 (ACC1), fatty acid synthase (Fas), sterol regulatory element-binding protein-1c (SREBP-1c), CCAAT/enhancer-binding protein α (C/EBPα), stearoyl-CoA desaturase 1 (SCD1), and peroxisome proliferator-activated receptor γ (PPARγ) had their expression downregulated by By lowering the Firmicutes/Bacteroidetes (F/B) ratio and controlling the number of certain gut bacteria. TT also alleviated HFD-induced abnormalities of the gut microbiota. The Muribaculaceae, Lachnospiraceae NK4A136_group, Alistipes, and Odoribacter families were identified as the major beneficial gut microorganisms using Spearman's correlation analysis. Fecal microbiota transplantation (FMT) demonstrated that TT's anti-obesity and gut microbiota-modulating benefits might be transmitted to mice on an HFD, demonstrating that one of TT's targets for preventing obesity is the gut microbiota. TT also increased the amount of short-chain fatty acids

	(SCFAs) in the feces, including acetic, propionic, and butyric acids. These results point to the possible development of TT as a prebiotic to combat obesity and the disorders it is connected with.

SCHOLARONE™
Manuscripts

Tibetan tea reduces obesity brought on by a high-fat diet and modulates gut flora in mice

Gang He[1,†] | Tangcong Chen[1,†] | Lifen Huang[1] | Yiyuan Zhang[1] | Yanjiao Feng[1] | Qijun Liu[1] | Xiaojing Yin[1] | Shaokui Qu[1] | Chen Yang[1] | Jianghong Wan[1, 2] | Li Liang[1] | Jun Yan[1] | Wei Liu[1]

[1]Key Laboratory of Medicinal and Edible Plants Resources Development of Sichuan Education Department, Sichuan Industrial Institute of Antibiotics, School of Pharmacy, Chengdu University, Chengdu, China

[2]Sichuan Jiang's Tibetan Tea Co., LTD, Ya'an, China

[†]These authors contributed equally to this work.

Correspondence

Gang He, Key Laboratory of Medicinal and Edible Plants Resources Development of Sichuan Education Department, Sichuan Industrial Institute of Antibiotics, School of Pharmacy, Chengdu University, Chengdu,610106, China

Email: hegang@cdu.edu.cn

Wei Liu, Key Laboratory of Medicinal and Edible Plants Resources Development of Sichuan Education Department, Sichuan Industrial Institute of Antibiotics, School of Pharmacy, Chengdu University, Chengdu,610106, China

Email: jmee@cdu.edu.cn

Funding information

This project was supported by the National Natural Science Foundation of China, Grant Number: 31870655 and the Sichuan Province Science and Technology Support Program, Grant Number: 2019YFH0054 and 2020YFH0205.

For Review Only

Abstract

It has been shown that Tibetan tea (TT) inhibits obesity and controls lipid metabolism. The fundamental processes by which TT prevents obesity are yet entirely unknown. Consequently, this research aimed to ascertain if TT may prevent obesity by modifying the gut flora. Our research demonstrated that TT prevented mice from gaining weight and accumulating fat due to the high-fat diet (HFD), decreased levels of blood total cholesterol (TC), triglycerides (TG), and low-density lipoprotein cholesterol (LDL-C), and raised levels of high-density lipoprotein cholesterol (HDL-C). Adipogenesis-related genes such as acetyl-Coenzyme A carboxylase 1 (ACC1, LOC107476), fatty acid synthase (Fas, LOC14104), sterol regulatory element-binding protein-1c (SREBP-1c, LOC20787), CCAAT/enhancer-binding protein α (C/EBPα, LOC12606), stearoyl-CoA desaturase 1 (SCD1, LOC20249), and peroxisome proliferator-activated receptor γ (PPARγ, LOC19016) had their expression downregulated by lowering the *Firmicutes/Bacteroidetes* (*F/B*) ratio and controlling the number of certain gut bacteria. TT also alleviated HFD-induced abnormalities of the gut microbiota. The *Muribaculaceae*, *Lachnospiraceae NK4A136_group*, *Alistipes*, and *Odoribacter* families were identified as the major beneficial gut microorganisms using Spearman's correlation analysis. Fecal microbiota transplantation (FMT) demonstrated that

TT's anti-obesity and gut microbiota-modulating benefits might be transmitted to mice on an HFD, demonstrating that one of TT's targets for preventing obesity is the gut microbiota. TT also increased the amount of short-chain fatty acids (SCFAs) in the feces, including acetic, propionic, and butyric acids. These results indicate the possible development of TT as a prebiotic to combat obesity and associated disorders.These results suggest that TT may act as a prebiotic against obesity and its associated diseases.

KEYWORDS

Tibetan tea, obesity, high-fat diet, gut flora, short-chain fatty acids

1 | INTRODUCTION

Obesity is a chronic condition that may cause a variety of health issues and shorten life expectancy (Blüher, 2013). Weight increase is merely one sign of obesity. However, it is also linked to problems with lipid and blood glucose metabolism, chronic inflammation, and a higher risk of many illnesses, including type 2 diabetes, hypertension, and cancer (Saltiel & Olefsky, 2017). Obesity has a complicated etiology influenced by hereditary and non-genetic variables. In recent years, mounting research has revealed that obesity may be caused by an imbalance in the gut flora (B. N. Liu, Liu, Liang, & Wang, 2021).

The gut microbiome consists of thousands of bacterial species, mainly *Bacteroidota*, *Firmicutes*, *Proteobacteria,* and *Actinobacteria* (Gasmi Benahmed et al., 2021; Pung et al., 2022). The development of obesity is strongly correlated with gut microbiota, according to several research (He et al., 2022; Kang et al., 2022; Su et al., 2022). The development of obesity is impacted by how a high-fat diet (HFD) changes the gut microbiota's composition by drastically increasing the ratio of *Firmicutes* to *Bacteroidota* (*F/B*) (Houtman, Eckermann, Smidt, & de Weerth, 2022; Indiani et al., 2018).

Numerous natural bioactive substances, including dietary polysaccharides, have been shown to suppress obesity and control lipid metabolism via controlling the gut microbiota in recent years (L. Li et al., 2022; Rehman, Khan, Xin, & Liang, 2022; Y. Zhang et al., 2021). These findings imply that dietary interventions for obesity may affect the gut microbiome (Asadi et al., 2022).

Depending on processing techniques and production regions, tea is classified as green, yellow, white, oolong, dark, and black (Xu et al., 2018). *Tibetan* tea (TT) is a type of dark tea, mainly produced in Ya'an, Sichuan Province, China, and has a history of about 1000 years (Xie et al., 2018). TT is a geographically iconic ethnic product made from several processes, which give it its unique flavour. The most important of these is pile fermentation (Q. Zheng, Li, Zhang, Gao, & Tan, 2020), in which microorganisms perform a variety of reactions on the tea leaves, including degradation, oxidation and condensation, changing the tea's chemical compounds (Q. Li et al., 2018). The Tibetan's extreme diet (red meat and zanba as the primary food) is high in cholesterol, fat, and sugar. The *Tibetan* ancestors found that drinking TT could keep them healthy and avoid the harm of hyperlipidemia, hyperglycemia, hypertension, and other diseases caused by obesity (K. Li et al., 2022). Many studies have shown that TT has a variety of pharmacological effects, such as weight loss(Yuan et al., 2016), antioxidants (Xie et al., 2018), anti-radiation (Yuan et al., 2016), and protection against ulcerative colitis (N. Wang et al., 2021).

For Review Only

Although studies have reported many benefits of TT, its effect on obesity is unclear. The lipid and glucose metabolism, as well as weight, in TT-gavaged HFD-induced obese mice, were the main subjects of this work. By transplanting fecal bacteria, we confirmed the gut microbiota's involvement in preventing obesity and further our investigation into the gut microbiota's structure. Our study suggests that TT can treat obesity and its complications through dietary intervention.

2 | MATERIALS AND METHODS

2.1 | Materials

The experimental animals were given both a low-fat diet and a typical control diet. The standard control diet from Keao Xieli Feed Co., Ltd. in Beijing, China, had 3.40 kcal/g of fat, 11.85% of protein, and 65.08% of carbs. The high-fat diet, which had 5.13 kcal/g of calories and included 27.20% carbs, 34.5% fat, and 23.25% protein, was purchased from Jiangsu Pharmaceutical & Bioengineering Co., Ltd. in Jiangsu, China. Caffeine, gallic acid (GA), catechin, epigallocatechin gallate (EGCG),(-)-epigallocatechin (EGC), and epicatechin (EC) were purchased from Yuanye Biotechnology Co., Ltd (Shanghai, China). Methanol, anhydrous ethanol, acetonitrile and phenol reagents (all HPLC grade) were purchased from Sigma-Aldrich (Saint Louis, USA). Other reagents such as acetic acid, propionic acid, butyric acid and isobutyric acid are of analytical grade (Kelong Chemical Co., Ltd, Chengdu, China).

2.2 | Preparation of TT water extracts and compositional analysis

TT was extracted as described by Gong (Gong et al., 2020) with minor modifications. 500 g TT (Sichuan Jiang's Tibetan Tea Co., LTD) was crushed and extracted in 5 L purified water for 30 min, and the water temperature was maintained at 90 °C. Ultrasonic extraction was performed for 10 min, and filter residue and filtrate were collected by filtration solution. Add 1.5 L pure water to the filter residue, extract at 90 °C for 20 min, and collect the filtrate. The filtrate was combined twice, concentrated to 1/10 volume, and vacuum freeze-dried to obtain the TT extract.

The quantification of tea polyphenols, total flavonoids and soluble sugar in TT water extracts was carried out by the Folin-Ciocalteu colorimetric method(Pérez-Burillo, Giménez, Rufián-Henares, & Pastoriza, 2018), the aluminum trichloride-sodium nitrite colorimetric assay(Y. Liu et al., 2018) and the phenol-sulfuric acid method(Y. Liu et al., 2021), respectively. Non-targeted metabolomic analysis using liquid chromatography with tandem mass spectrometry (LC-MS/MS)(Y. Liu et al., 2022). The content of several chemicals, such as catechin, caffeine, GA, EGCG, EGC and EC, was then determined by high performance liquid chromatography(Y. Liu et al., 2022).

2.3 | Animals and experimental design

At 8 weeks old and 20 ± 2 g each, 60 male C57BL/6J mice were purchased from SiPeiFu Biotechnology Co., Ltd. in Beijing, China. They were given access to food, water, and a 12-hour light/dark cycle while being housed in a controlled environment with a temperature of 22 ± 2 °C and a humidity level of 55 ± 5 %. The Sichuan Industrial Institute of Antibiotics' Ethical Committee at Chengdu University gave its approval for the animal trials (Approval Number: SIIA 20210706). The study was. After a week of acclimatization, the mice were randomly

For Review Only

divided into six groups, each with 10 mice: (1) *NCD*, which received standard chow and was treated with 0.9% saline; (2) *NCD_TT_H*, which received standard chow and was treated with 400 mg/kg of TT extract; (3) *HFD*, which received high-fat chow and was treated with 0.9% saline; (4) *HFD_TT_L*, which received high-fat feed and was treated with 100 mg/kg of TT extract; (5) *HFD_TT_M* group, which received high-fat feed and was treated with 200 mg/kg of TT extract; (6) *HFD_TT_H* group, which received high-fat feed and was treated with 400 mg/kg of TT extract. The weight of the mice was noted weekly throughout the nine-week trial. After a 12-hour fast, mice were slaughtered at the end of the ninth week, and blood and fat samples were taken. The other tissue was snap-frozen in liquid nitrogen, and the epididymal fat was preserved in 4% formaldehyde for further study.

2.4 | Fecal microbiota transplantation

FMT was performed according to the method described by Chang et al (Chang et al., 2015). Briefly, mice in the *NCD*, *NCD_TT_H*, *HFD*, and *HFD_TT_H* groups were fed for two months and then used as donor mice. The faeces of each of the four groups of mice were collected in sterile cages; 200 mg of faeces were resuspended in 2 mL of sterile saline, vortexed for ten seconds, and then centrifuged at 800 g for 3 min. 8-week-old male recipient mice were fed HFD and gavaged with fresh graft samples (100 μL per mouse) daily for 2 months.

For Review Only

2.5 | Biochemical analysis

According to the protocol of the kit (Jiancheng, Inc., Nanjing, China), the serum concentrations of high-density lipoprotein cholesterol (HDL-C), low-density lipoprotein cholesterol (LDL-C), total cholesterol (TC), and triglyceride (TG) were measured.

2.6 | Oral glucose tolerance test

The mice fasted for 12 hours at week 8. One gram of glucose per kilogram of body weight was gavaged, and blood glucose levels were checked at 0, 15, 30, 60, 90, and 120 min later.

2.7 | RNA extraction and analysis of gene expression

Through the use of the Trizol reagent procedure, total RNA was extracted from the tissue. Using Beijing Labgic Technology Co., Ltd.'s Reverse Transcription kit (which includes a dsDNase), cDNA was produced from an equal volume of total RNA. A StepOnePlusTM Real-Time PCR Detection System from Applied Biosystems was used to evaluate the generated cDNA.

The Supplementary Table 2 included the PCR primer sequences for the associated genes. 40 cycles of 95 °C for 120 s, 95 °C for 5 s, and 60 °C for 10 s were amplified during the PCR's three minutes at 95 °C. Glyceraldehyde-3-phosphate dehydrogenase (GAPDH, LOC14433) was used as an internal reference to compute the relative amount using the $2^{-\Delta\Delta Ct}$ technique.

2.8 | Histopathological analysis

A 4% formaldehyde solution was used to fix freshly removed mouse epididymal fat overnight. Later, it underwent dehydration, embedding, sectioning, and morphological analysis using hematoxylin and eosin staining.

2.9 | Gas chromatography-mass spectrometry analysis

Short-chain fatty acids were detected in feces using the technique previously described (X. Y. Zhang et al., 2020). The fecal sample was thawed, suspended in 1 mL of 25% methanol solution, and vortexed rapidly for 5 min. After centrifuging at 10,000 rpm at 4 °C for 10 min and performing a static extraction at 4 °C for 30 min, the supernatant was recovered. 0.04 M HCl (10:1,V/V) and the supernatant were combined before standing overnight. Before analysis, the supernatant was put into a clean micro gas-phase vial after being filtered via a 0.22 μm membrane. Gas chromatography and mass spectrometry (GC-MS) equipment from Perkin Elmer was used to analyze the samples (Perkin Elmer Technologies, USA).

2.10 | DNA extraction and sequencing of 16S rRNA

According to the manufacturer's recommendations, DNA was extracted from feces samples using the MagPure Soil DNA LQ Kit (Magen, Guangdong, China). Thermo Fisher Scientific, Waltham, Massachusetts, USA, microspectrophotometer was used to quantify the DNA content and integrity, and agarose gel electrophoresis was used to corroborate the results further. Using two universal primer pairs (343F: 5′-TACGGRAGGCAGCAG-3; 798R: 5′-AGGGTATCTAATCCT-3), the V3-V4 hypervariable portions of the bacterial 16S rRNA gene were amplified. Over a 25-L response. AMPure XP beads (Beckman Coulter Co., USA) were used to purify the PCR products, and a Qubit dsDNA assay kit was used to quantify them. The concentrations were then modified for sequencing. OE Biotechnology LTD. carried out sequencing and analysis of the 16S rRNA gene amplicon in Shanghai, China.

2.11 | Analytical statistics

The difference was shown to be significant using one-way ANOVA and the Tukey test. A statistically significant value of $P < 0.05$ was used to represent the data as mean ± SD (standard deviation). The studies utilized GraphPad Prism 9.0 (San Diego, California, USA).

3 | RESULTS

3.1 | Chemical composition of TT water extracts

The chemical composition and content of TT water extracts are shown in Table 1. The most dominant chemicals in TT water extracts are tea polyphenols, flavonoids and soluble sugars. We used non-targeted metabolomic analysis to validate the non-volatile metabolism of Tibetan tea water extracts and the results are shown in Supplementary Table 1. These include a wide range of amino acids and their derivatives, phenolic acids, flavonoids, terpenoids, alkaloids, lipids and other metabolites. The content of caffeine, GA, EGCG, EGC and EC were analysed by high performance liquid chromatography and the results are shown in Table 1. The caffeine content is higher than several other ingredients.

3.2 | TT inhibits HFD-induced obesity in mice

The HFD mice were noticeably heavier than the other five groups after nine weeks, as seen in Figure 1. After TT intervention, the weight of mice was significantly reduced. Interestingly, TT inhibited body weight in mice in the form of dose dependence (Figure 1A). The weights of various organs (Figure 1C) and the weights of mice's perirenal and epididymal fat (Figure 1B) show the beneficial effects of TT in the treatment of obesity. According to morphological analysis, TT effectively prevented HFD-induced fat formation (Figure 1D) and adipocyte

For Review Only

growth (Figures 1E and F) (P < 0.05).

3.3 | TT helps obese mice with their blood lipid and glucose metabolic disorders

Blood lipids and glucose levels are likely to change as obesity progresses. We performed a glucose tolerance test and determined the AUC values in the trial's eighth week (Figures 1H and I). According to the findings, between 15 and 120 min, the blood glucose levels of the mice given the HFD were greater than those of the animals fed the NCD. Additionally, compared to the *HFD* group, the mice's blood glucose levels were lower after TT intervention. The impact of TT on the serum biochemical parameters in mice is shown in Figure 1G and Table 2. We found that the HFD considerably reduced HDL-C and significantly raised fasting hyperglycemia, TC, TG, and LDL-C in mice. Feeding TT significantly reversed these trends (P < 0.05).

3.4 | TT ameliorates HFD-induced gut microbial disturbance in mice

For Review Only

We amplified the bacterial 16S rRNA gene's V3-V4 region and sequenced it using the Illumina MiSeq technology in order to evaluate the cumulative TT-induced alterations in the gut microbiota. According to principal components analysis (PCoA) (Figure 2A), the microbial community composition across the groups seemed to be clustered across the groups. The difference in PCoA1 was 45.09%, principally reflecting the impact of normal and high-fat diets on the gut microbiota structure; in contrast, the difference in PCoA2's vertical coordinate was 16.65%, primarily highlighting the impact of TT on the gut microbiota. The *HFD* group had the lowest chao1 values, observed species values, and goods coverage values compared to the other five groups, according to the alpha diversity analysis (Figures 2B-D), and these three values increased considerably after feeding TT. Figures 2E and F demonstrate this. The Shannon and Simpson index grew considerably after TT's involvement. These findings imply that HFD reduces the variety of the gut microbiota in mice. By boosting variety and abundance, feeding TT, on the other hand, greatly improved the gut microbiota structure.

Comparing the relative prevalence of the key microbial groups in the gut microbiota of six food groups allowed researchers to understand better how TT affects the regulation of the gut microbiota. *Bacteroidota*, *Firmicutes*, *Deferribacterota*, *Desulfobacterota*, and *Proteobacteria* were among the prominent species identified in histograms showing the relative abundance of gut microbiomes at the phylum level (Figure 3A). With more than 90% of the total, the two main phyla are Bacteroidota and Firmicutes. By increasing the number of *Firmicutes* (Figure 3B) and reducing the quantity of *Bacteroidota* (Figure 3C), the *HFD* group outperformed the other five groups in terms of *F/B* (Figure 3D). The gut microbiota's final makeup was changed. The most numerous bacteria were those belonging to the families *Muribaculaceae*, *Lachnospiraceae*, *Rikenellaceae*, *Prevotellaceae*, *Deferribacteraceae*, and *Oscillospiraceae* (Figure 3E). The TT intervention substantially reduced the population abundance of *Clostridia UCG-014* and *Desulfovibrionaceae* (Figure 3E) and increased the population abundance of *Muribaculaceae* (Figure 3F), *Prevotellaceae* (Figure 3G), *Rikenellaceae* (Figure 3H) as compared to the *HFD* group. After TT therapy, we examined the genus-level alterations in the gut microbiota, and the findings revealed variations across all experimental groups. The *HFD* group had lower relative population abundances of the *Muribaculaceae* (Figure 3I), *Lachnospiraceae_NK4A136_group* (Figure 3I), *Alistipes* (Figure 3J), *Bacteroides* (Figure 3I),

and *Colidextribacter* (Figure 3I) than the *NCD* and TT intervention groups. While *Bilophila* (Figure 3K), *Clostridia_UCG-014* (Figure 3L), *Mucispirillum* (Supplementary Figure 1A), and *Blautia* (Supplementary Figure 1B) had greater relative population abundances. These findings imply that animals on a high-fat diet had changed gut microbiotas and that TT intervention corrected these abnormalities.

3.5 | TT's impact on the SCFAs

The impact of TT on the level of SCFAs in mouse feces is seen in Table 3. The amount of fecal SCFAs in the *HFD* group was much lower than that of the other five groups. The contents of butyric acid, propionic acid, and acetic acid in the mouse feces of the *NCD_TT_H* group were substantially greater than those of the *NCD* group (P < 0.05). After TT intervention, animals had substantially increased amounts of butyric acid, propionic acid, and acetic acid in their feces compared to the *HFD* group. Similar to this, TT significantly altered branched-chain fatty acids (BCFAs), mostly by increasing the quantity of isobutyric acid; however, isovaleric acid did not change.

3.6 | TT regulates the expression of genes associated with obesity

Figure 4 displays the lipid synthesis-related genes' levels of gene expression. When compared to the *NCD* group, the *NCD_TT_H* group's expression of ACC1, C/EBPα, Fas, PPARγ, SCD1, and SREBP-1c was considerably lower (P < 0.05). The expression of six genes was also significantly lower in the three TT intervention groups than in the *HFD* group (P < 0.05). Interestingly, in comparison to the *HFD* group, there was a dose-dependent reduction in the expression of ACC1, C/EBPα, Fas, and SREBP-1c (Figures 4A, B, C, and F).

3.7 | Transplantation of feces microbiota prevents obesity and improves lipid metabolism in mice

To study the impact of TT on the gut microorganisms of obese mice, we transplanted feces from four donor mouse groups (*NCD*, *NCD_TT_H*, *HFD*, *HFD_TT_ H*) into HFD-fed animals. Then, we examined obesity-related characteristics and metabolic alterations in glucose and lipids levels. The findings demonstrated that fecal transplantation from the *NCD* group, *NCD_TT_H* group, and *HFD_TT_H* group decreased the body weight (Figure 5A), liver weight (Figure 5C), fat accumulation (Figures 5B and D), and adipocyte growth (Figures 5E, F) of recipient mice in comparison to the *HFD* group. Similar to Table 2, Table 4 depicts the variations in blood glucose and cholesterol levels in mice. The *HFD_HFD* group showed substantially greater fasting glucose, TC, TG, and LDL-C levels and higher HDL-C levels than the other three groups.

3.8 | Fecal microbiota transplantation alters the makeup of the gut microbiota in obese mice

We examined the microbial composition of the guts of four groups of mice that had their faecal microbiota transplanted in order to investigate the regulatory effects of TT on gut microorganisms. PCoA, NMDS, and the Unweighted Pair Group Method with Arithmetic Mean (UPGMA) were used in the investigation of the diversity. According to PCoA, there were significant differences between the intestinal microbiotas of the four mouse groups (Figure 6A). For PC1 and PC2, these differences were 20.66% and 14.62%, respectively, with the

For Review Only

NCD_ H_HFD and *NCD_ HFD* groups being distributed in the right quadrant and the *HFD_ H_HFD* and *HFD_ HFD* groups in the left quadrant. Similar findings from the NMDS analysis were obtained (Figure 6B), and the UPGMA sample hierarchical cluster analysis revealed that each of the four groups was grouped into a single cluster (Figure 6C). The alpha diversity amongst the four groups also varied significantly in a noticeable way. In comparison to the other three groups, *HFD_HFD* had lower values for ACE, Chao 1, species richness, Shannon, and Simpson indices (Figures 6D-H). After transplanting mouse feces fed with TT, however, all of these values rose.

We analyzed the microbial makeup of four groups to further look into the particular gut microbiota alterations at various taxonomic levels. Similar to the earlier findings, the *HFD_HFD* group changed the makeup of the gut microbiota at the phylum level by raising the *F/B* ratio, which fell following the transplantation of mouse feces from TT-fed animals (Figures 7A-D). Additionally, there were notable changes between the four groups' microbial compositions at the family level (Figure 7E). Transplantation of TT-fed mouse feces substantially enhanced the relative population abundance of *Lachnospiraceae* (Figure 7E), *Muribaculaceae* (Figure 7F), *Ruminococcaceae* (Figure 7E), and *Prevotellaceae* (Figure 7G) compared to the *HFD_HFD* group, whereas *Clostridia_UCG-014* (Figure 7H) and *Deferribacteraceae* decreased in relative population abundance. In all experimental groups, changes in gut microbiota were seen at the genus level. In comparison to the *HFD_HFD* group, the relative population abundance of the *Lachnospiraceae_NK4A136_group* (Supplementary Figure 1C), *Colidextribacter* (Supplementary Figure 1D), *Alistipes* (Figure 7J), and *Oscillibacter* (Figure 7L) was greater in both the *NCD_H_HFD* group and the *HFD_H_HFD* group, whereas the *Bilophila* (Figure 7K) was lower. These findings revealed that an *HFD* changed the microbial composition of mice's guts. However, the gut microbial diversity and disordered intestinal microorganisms were enhanced when mice fed TT's feces were transplanted.

3.9 | Association of gut microbiota with obesity-related parameters, SCFAs, and genes related to lipid synthesis

We used Spearman's correlation analysis to determine the gut microbiota that predominated and may have influenced how well TT prevented obesity caused by the HFD. The heatmap in Figure 8 demonstrated a positive or negative correlation between the top 15 most prevalent genera and factors associated with obesity, the presence of SCFAs, and genes involved in lipid synthesis. As seen in Figure 8A, *Lachnospiraceae_NK4A136_group*, *Odoribacter*, *Alistipes,* and *Muribaculaceae* exhibited a positive correlation with HDL-C and a negative correlation with other obesity-related parameters, like blood glucose, body weight TC and TG; *Colidextribacter*, *Blautia,* and *Bilophila* showed the opposite trend compared to the gut mentioned above microbes. Interestingly, *Lachnospiraceae_NK4A136_group*, *Odoribacter*, *Alistipes,* and *Muribaculaceae* also showed a negative correlation with the PPARγ, SREBP1c, Fas, C/EBPα, ACC1, and SCD1 expressions, while the remaining 11 gut microbes showed a positive correlation (Figure 8B). *Lachnospiraceae_NK4A136_group*, *Odoribacter*, *Alistipes,* and *Muribaculaceae* showed a clear positive link exists between the concentrations of SCFAs and BCFAs in feces (Figure 8C).

4 | DISCUSSION

For Review Only

As an epidemic, obesity is related to many cardiovascular and metabolic diseases, even cancer (Bagnall et al., 2019). Statistics show that each year, almost 2.8 million individuals pass away due to obesity-related illnesses (Wiciński, Gębalski, Gołębiewski, & Malinowski, 2020), making the need for obesity treatment and prevention urgent. According to several research, drinking dark tea may help prevent and cure obesity (Lim et al., 2022; C. Wang et al., 2022). There have been claims that the dark tea variety TT offers anti-obesity properties (Yuan et al., 2016). Its anti-obesity properties and influence on gut microorganisms have an unknown underlying mechanism. The present study explored how TT inhibited HFD-induced obesity in mice.

In mice, a high-fat diet was shown to increase weight growth and fat storage, whereas TT therapy was found to reduce these tendencies. Additionally, we discovered that, in line with several research, feeding TT to obese mice decreased fasting hyperglycemia, TC, TG, and LDL-C and elevated HDL-C. In mice with obesity brought on by the HFD, liupao tea extract was utilized to reduce body weight via altering oxidative stress and lipid metabolism (Y. Wu, Sun, Yi, Tan, & Zhao, 2021). Pu-erh tea decreased obesity in mice via modifying the gut microbiome(Ye et al., 2021). Our findings amply supported the notion that TT suppresses HFD-induced obesity in mice, controls glucose and lipid metabolism, and is helpful in the management of obesity.

For Review Only

Numerous research in recent years has proven that the gut microbiota is essential for controlling the host's lipid and glucose metabolism (Chen et al., 2018; B. N. Liu et al., 2021; J. Wang & Jia, 2016). As a result, one possible target for the treatment of obesity is the gut flora. In the current research, the gut bacteria in the HFD group of mice saw a considerable alteration in structure and composition. Obese mice's disturbed gut microbes were improved after receiving TT therapy, mostly in the form of enhanced gut microbiota diversity and abundance. Furthermore, the *F/B* ratio is one trait of the gut microbiota of obese mice (Abenavoli et al., 2019; Stojanov, Berlec, & Štrukelj, 2020). The *HFD* group in the current research had the highest *F/B* values, which were dramatically decreased by the TT therapy. In compared to the *HFD* group, the TT treatment enhanced the relative population abundance of the families *Muribaculaceae*, *Prevotellaceae*, *Rikenellaceae*, and *Bacteroidaceae* while decreasing the relative population abundance of the families *Clostridia_UCG-014* and *Desulfovibrionaceae*. Less of the genus related to controlling obesity, such as *Muribaculaceae*, *Prevotellaceae,* and *Rikenellaceae*, were present in the fat lab animals (Shen et al., 2017; Zhao, Lyu, Zhai, Sun, & Ding, 2022; Ziętak et al., 2016). According to studies, *Clostridia_UCG-014* is more prevalent and favorably linked with blood glucose in obesity models (Koontanatechanon et al., 2022).

Similarly, *Desulfovibrionaceae* was found to be increased in abundance due to a high-fat diet (C. Zhang et al., 2010). At the genus level, the relative population abundances of *Bilophila*, *Blautia*, *Clostridia_UCG-014,* and *Mucispirillum* were greater in the *HFD* group than those of *Alistipes*, *Muribaculaceae*, *Lachnospiraceae_NK4A136_group*, *Bacteroides* and *Colidextribacter*. According to reports, Colidextribacter may release inosine, possibly reducing acute liver damage and inflammation brought on by LPS (Guo et al., 2021). On the other hand, the lipopolysaccharide-producing bacteria *Bilophila* has been linked to worsening inflammation, and metabolic abnormalities brought on by HFD in mice (Lu et al., 2021). With Spearman correlation analysis, we found that Lachnospiraceae_NK4A136_group,

Odoribacter, Alistipes, and Muribaculaceae were inversely linked with the majority of obesity-related variables, revealing that these gut microbes may play a role as beneficial microbes(Figure 8A).

The anti-obesity properties of TT were transmitted to mice fed an HFD via faecal microbiota transplantation studies. We found that recipient mice gained less weight after receiving transplants of TT-fed mouse feces and that fat storage and adipocyte growth were suppressed. The blood concentrations of TC, TG, LDL-C, and HDL-C in the other three mouse groups were also lower than those in the *HFD_HFD* group. It's interesting to note that FMT had a significant impact on obese mice's gut flora as well. The key symptoms were the gut microbiota's altered diversity, composition, and structure. By changing *F/B* values, FMT modified the phylum-level composition of the gut microbiota in obese mice. At the family and genus levels, the *NCD_H_HFD* and *HFD_H_HFD* groups demonstrated a more substantial prevalence of *Lachnospiraceae*, *Muribaculaceae*, *Ruminococcaceae*, *Prevotellaceae*, *Lachnospiraceae_NK4A136_group*, *Colidextribacter,* and *Alistipes*. However, there were fewer instances of *Clostridia_UCG-014*, *Deferribacteraceae*, *Bilophila,* and *Blautia*. These findings showed that TT alters the gut microbiota of obese mice and that this alteration helps with weight reduction.

It has been reported that the fungi *Alistipes, Prevotellaceae,* and *Lachnospiraceae_NK4A136_group* generate SCFAs (David et al., 2014; Koh, De Vadder, Kovatcheva-Datchary, & Bäckhed, 2016; J. Li, Zhao, Li, Wang, & Zhao, 2022; M. Wu et al., 2020). SCFAs, which are mostly composed of acetic, propionic, and butyric acids and are often formed by the fermentation of dietary polysaccharides by gut bacteria, play an important role in the prevention and treatment of obesity (Coppola, Avagliano, Calignano, & Berni Canani, 2021; Zaky, Glastras, Wong, Pollock, & Saad, 2021). The G protein-coupled receptor GPR43, which is connected to calorie expenditure and lipid metabolism, is activated by SCFAs as endogenous signaling molecules (Schoeler & Caesar, 2019). Additionally, SCFAs enhance glucagon-like peptide-1 and gut hormone secretion, which decreases appetite and increases fullness in the body (Kimura, Ichimura, Ohue-Kitano, & Igarashi, 2020). SCFAs may also maintain gut homeostasis by modulating the gut-brain axis and preserving the integrity of the gut barrier (Silva, Bernardi, & Frozza, 2020; L. Zheng et al., 2017). Through the use of GC-MS, we were able to ascertain the amount of SCFAs present in mouse feces and discovered that the TT treatment dramatically boosted the synthesis of SCFAs, namely acetic, propionic, and butyric acids. It was shown that TT could also regulate lipid metabolism and maintain gut homeostasis by increasing the production of SCFAs, thus effectively preventing HFD-induced obesity.

Obesity is characterized by an increase in adipose tissue weight brought on by an increase in the quantity and size of adipocytes (Yang & Kim, 2015). Therefore, preventing the development of obesity and the problems is connected with lowering the creation and storage of fat. Our findings demonstrated that TT decreased the weight increase and adipocyte growth brought on by the HFD. We also discovered that TT therapy decreased the expression of the genes ACC1, C/EBPα, Fas, PPARγ, SCD1, and SREBP-1c in the mice's adipose tissue. The fatty acid production pathway's rate-limiting enzyme is known as ACC1 (Y. Wang et al., 2022). PPARγ has been shown to control the expression of genes associated with adipose tissue and to encourage lipogenesis (H. J. Lee, Le, Lee, Choi, & Yang, 2018).

For Review Only

C/EBPα is a crucial control point for adipocyte differentiation and PPARγ-induced lipogenesis (J. E. Lee, Schmidt, Lai, & Ge, 2019). SREBP-1c is an essential transcription factor that controls the expression of the genes involved in the fatty acid synthesis and activates the adipogenic transcription factors ACC-1, Fas, and SCD1, which control the formation of adipose tissue and the accumulation of lipids (Fang et al., 2019; Linden et al., 2018; Zhu et al., 2019).

5 | CONCLUSIONS

As a result of our research, we were able to demonstrate that TT dramatically inhibited HFD-induced weight gain, fat accumulation, hyperglycemia, and hyperlipidemia in mice, as well as control the expression of genes involved in lipid metabolism. The way that TT controls the gut microbiota produces SCFAs and controls the expression of genes involved in lipid synthesis may be responsible for its positive effects on obesity. Our research gave TT's potential as a functional beverage for preventing and treating obesity a new direction.

CREDIT AUTHOR STATEMENT

Gang He: Supervision(equal);data curation (equal); software(equal); investigation(equal); methodology(equal);formal analysis(equal); writing- review & editing(equal);funding acquisition(equal); provided the concept(equal).**Tangcong Chen:** Data curation(equal); software(equal); investigation(equal); methodology(equal); formal analysis(equal); writing-original draft(equal);writing - review & editing(equal).**Lifen Huang:** Formal analysis(equal); writing - review & editing(equal); investigation; methodology(equal).**Yiyuan Zhang:** Investigation(equal); methodology(equal); writing - review & editing(equal).**Yanjiao Feng:** Formal analysis and methodology(equal); writing - review & editing(equal).**Qijun Liu:** Formal analysis and methodology(equal); writing - review & editing(equal). **Xiaojing Yin:** Formal analysis and methodology(equal); writing - review & editing(equal).**Shaokui Qu:** Investigation; methodology(equal); writing - review & editing(equal).**Chen Yang:** Formal analysis and methodology(equal).**Jianghong Wan:** Formal analysis(equal); supervision and provided the concept(equal).**Li Liang:** Formal analysis(equal); writing - review & editing(equal).**Jun Yan:** Formal analysis(equal); writing - review & editing(equal).**Wei Liu:** Supervision(equal); funding acquisition(equal); provided the concept(equal).

ACKNOWLEDGMENTS

This project was supported by the National Natural Science Foundation of China (Grant Number: 31870655) and the Sichuan Province Science and Technology Support Program (Grant Number: 2019YFH0054 and 2020YFH0205).

ETHICS STATEMENTS

The Sichuan Industrial Institute of Antibiotics at Chengdu University's Protection of Laboratory Animals committee examined and approved the animal research (Chengdu, China; Approval Number: SIIA 20210706)

CONFLICTS OF INTEREST

The authors declare no conflicts of interest.

DATA AVAILABILITY STATEMENT

The NCBI public database may be accessed at the following site. The raw readings were submitted there. https://www.ncbi.nlm.nih.gov/sra/PRJNA901348

For Review Only

ORCID

Gang He https://orcid.org/0000-0001-5026-1493

REFERENCES

Abenavoli, L., Scarpellini, E., Colica, C., Boccuto, L., Salehi, B., Sharifi-Rad, J., . . . Capasso, R. (2019). Gut Microbiota and Obesity: A Role for Probiotics. *Nutrients,* 11(11).

Asadi, A., Shadab Mehr, N., Mohamadi, M. H., Shokri, F., Heidary, M., Sadeghifard, N., & Khoshnood, S. (2022). Obesity and gut-microbiota-brain axis: A narrative review. *J Clin Lab Anal,* 36(5), e24420.

Bagnall, A. M., Radley, D., Jones, R., Gately, P., Nobles, J., Van Dijk, M., . . . Sahota, P. (2019). Whole systems approaches to obesity and other complex public health challenges: a systematic review. *BMC Public Health,* 19(1), 8.

Blüher, M. (2013). Adipose tissue dysfunction contributes to obesity related metabolic diseases. *Best Pract Res Clin Endocrinol Metab,* 27(2), 163-177.

Chang, C. J., Lin, C. S., Lu, C. C., Martel, J., Ko, Y. F., Ojcius, D. M., . . . Young, D. E. (2015). Ganoderma lucidum reduces obesity in mice by modulating the composition of the gut microbiota. *Nature Publishing Group*(1).

Chen, G., Xie, M., Dai, Z., Wan, P., Ye, H., Zeng, X., & Sun, Y. (2018). Kudingcha and Fuzhuan Brick Tea Prevent Obesity and Modulate Gut Microbiota in High-Fat Diet Fed Mice. *Mol Nutr Food Res,* 62(6), e1700485.

Coppola, S., Avagliano, C., Calignano, A., & Berni Canani, R. (2021). The Protective Role of Butyrate against Obesity and Obesity-Related Diseases. *Molecules,* 26(3).

David, L. A., Maurice, C. F., Carmody, R. N., Gootenberg, D. B., Button, J. E., Wolfe, B. E., . . . Turnbaugh, P. J. (2014). Diet rapidly and reproducibly alters the human gut microbiome. *Nature,* 505(7484), 559-563.

Fang, K., Wu, F., Chen, G., Dong, H., Li, J., Zhao, Y., . . . Lu, F. (2019). Diosgenin ameliorates palmitic acid-induced lipid accumulation via AMPK/ACC/CPT-1A and SREBP-1c/FAS signaling pathways in LO2 cells. *BMC Complement Altern Med,* 19(1), 255.

Gasmi Benahmed, A., Gasmi, A., Doşa, A., Chirumbolo, S., Mujawdiya, P. K., Aaseth, J., . . . Bjørklund, G. (2021). Association between the gut and oral microbiome with obesity. *Anaerobe,* 70, 102248.

Gong, Z. P., Ouyang, J., Wu, X. L., Zhou, F., Lu, D. M., Zhao, C. J., . . . Zhu, M. Z. (2020). Dark tea extracts: Chemical constituents and modulatory effect on gastrointestinal function. *Biomed Pharmacother,* 130, 110514.

Guo, W., Xiang, Q., Mao, B., Tang, X., Cui, S., Li, X., . . . Chen, W. (2021). Protective Effects of Microbiome-Derived Inosine on Lipopolysaccharide-Induced Acute Liver Damage and Inflammation in Mice via Mediating the TLR4/NF-κB Pathway. *J Agric Food Chem,* 69(27), 7619-7628.

He, G., Chen, T., Huang, L., Zhang, Y., Feng, Y., Qu, S., . . . Liu, W. (2022). Tremella fuciformis polysaccharide reduces obesity in high-fat diet-fed mice by modulation of gut microbiota. *Frontiers in Microbiology,* 13.

Houtman, T. A., Eckermann, H. A., Smidt, H., & de Weerth, C. (2022). Gut microbiota and BMI throughout childhood: the role of firmicutes, bacteroidetes, and short-chain fatty acid producers. *Sci Rep,* 12(1), 3140.

For Review Only

Indiani, C., Rizzardi, K. F., Castelo, P. M., Ferraz, L. F. C., Darrieux, M., & Parisotto, T. M. (2018). Childhood Obesity and Firmicutes/Bacteroidetes Ratio in the Gut Microbiota: A Systematic Review. *Child Obes,* 14(8), 501-509.

Kang, Y., Kang, X., Yang, H., Liu, H., Yang, X., Liu, Q., . . . Fan, W. (2022). Lactobacillus acidophilus ameliorates obesity in mice through modulation of gut microbiota dysbiosis and intestinal permeability. *Pharmacol Res,* 175, 106020.

Kimura, I., Ichimura, A., Ohue-Kitano, R., & Igarashi, M. (2020). Free Fatty Acid Receptors in Health and Disease. *Physiol Rev,* 100(1), 171-210.

Koh, A., De Vadder, F., Kovatcheva-Datchary, P., & Bäckhed, F. (2016). From Dietary Fiber to Host Physiology: Short-Chain Fatty Acids as Key Bacterial Metabolites. *Cell,* 165(6), 1332-1345.

Koontanatechanon, A., Wongphatcharachai, M., Nonthabenjawan, N., Jariyahatthakij, P., Leksrisompong, P., Srichana, P., . . . Pawa, K. K. (2022). The Effects of Increasing Dietary Fat on Serum Lipid Profile and Modification of Gut Microbiome in C57BL/6N Mice. *J Oleo Sci,* 71(7), 1039-1049.

Lee, H. J., Le, B., Lee, D. R., Choi, B. K., & Yang, S. H. (2018). Cissus quadrangularis extract (CQR-300) inhibits lipid accumulation by downregulating adipogenesis and lipogenesis in 3T3-L1 cells. *Toxicol Rep,* 5, 608-614.

Lee, J. E., Schmidt, H., Lai, B., & Ge, K. (2019). Transcriptional and Epigenomic Regulation of Adipogenesis. *Mol Cell Biol,* 39(11).

Li, J., Zhao, M., Li, J., Wang, M., & Zhao, C. (2022). Combining fecal microbiome and metabolomics to reveal the disturbance of gut microbiota in liver injury and the therapeutic mechanism of shaoyao gancao decoction. *Front Pharmacol,* 13, 911356.

Li, K., Zhang, Q., Cai, H., He, R., Nima, Q., Li, Y., . . . Liu, Q. (2022). Association of Tibetan Habitual Food and Metabolic Syndrome Among Tibetan People in China: A Cross-Sectional Study. *Front Nutr,* 9, 888317.

Li, L., Ma, L., Wen, Y., Xie, J., Yan, L., Ji, A., . . . Sheng, J. (2022). Crude Polysaccharide Extracted From Moringa oleifera Leaves Prevents Obesity in Association With Modulating Gut Microbiota in High-Fat Diet-Fed Mice. *Front Nutr,* 9, 861588.

Li, Q., Chai, S., Li, Y., Huang, J., Luo, Y., Xiao, L., & Liu, Z. (2018). Biochemical Components Associated With Microbial Community Shift During the Pile-Fermentation of Primary Dark Tea. *Front Microbiol,* 9, 1509.

Lim, H. J., Lim, T. J., Lee, J. H., Lee, J. H., Kim, M. O., Park, J. Y., . . . Choi, S. H. (2022). Anti-obesity Effects of Dark Tea Extracts by Down-regulation of C/EBPα and PPARγ. *In Vivo,* 36(4), 1753-1760.

Linden, A. G., Li, S., Choi, H. Y., Fang, F., Fukasawa, M., Uyeda, K., . . . Liang, G. (2018). Interplay between ChREBP and SREBP-1c coordinates postprandial glycolysis and lipogenesis in livers of mice. *J Lipid Res,* 59(3), 475-487.

Liu, B. N., Liu, X. T., Liang, Z. H., & Wang, J. H. (2021). Gut microbiota in obesity. *World J Gastroenterol,* 27(25), 3837-3850.

Liu, Y., Huang, W., Zhang, C., Li, C., Fang, Z., Zeng, Z., . . . Lan, X. (2022). Targeted and untargeted metabolomic analyses and biological activity of Tibetan tea. *Food Chem,* 384, 132517.

Liu, Y., Li, Y., Ke, Y., Li, C., Zhang, Z., Wu, Y., . . . Wu, W. (2021). In vitro saliva-

For Review Only

gastrointestinal digestion and fecal fermentation of Oudemansiella radicata polysaccharides reveal its digestion profile and effect on the modulation of the gut microbiota. *Carbohydr Polym,* 251, 117041.

Liu, Y., Luo, L., Liao, C., Chen, L., Wang, J., & Zeng, L. (2018). Effects of brewing conditions on the phytochemical composition, sensory qualities and antioxidant activity of green tea infusion: A study using response surface methodology. *Food Chem,* 269, 24-34.

Lu, H., You, Y., Zhou, X., He, Q., Wang, M., Chen, L., . . . Lou, L. (2021). Citrus reticulatae pericarpium Extract Decreases the Susceptibility to HFD-Induced Glycolipid Metabolism Disorder in Mice Exposed to Azithromycin in Early Life. *Front Immunol,* 12, 774433.

Pérez-Burillo, S., Giménez, R., Rufián-Henares, J. A., & Pastoriza, S. (2018). Effect of brewing time and temperature on antioxidant capacity and phenols of white tea: Relationship with sensory properties. *Food Chem,* 248, 111-118.

Pung, H. C., Lin, W. S., Lo, Y. C., Hsu, C. C., Ho, C. T., & Pan, M. H. (2022). Ulva prolifera polysaccharide exerts anti-obesity effects via upregulation of adiponectin expression and gut microbiota modulation in high-fat diet-fed C57BL/6 mice. *J Food Drug Anal,* 30(1), 46-61.

Rehman, A. U., Khan, A. I., Xin, Y., & Liang, W. (2022). Morchella esculenta polysaccharide attenuate obesity, inflammation and modulate gut microbiota. *AMB Express,* 12(1), 114.

Saltiel, A. R., & Olefsky, J. M. (2017). Inflammatory mechanisms linking obesity and metabolic disease. *J Clin Invest,* 127(1), 1-4.

Schoeler, M., & Caesar, R. (2019). Dietary lipids, gut microbiota and lipid metabolism. *Rev Endocr Metab Disord,* 20(4), 461-472.

Shen, F., Zheng, R. D., Sun, X. Q., Ding, W. J., Wang, X. Y., & Fan, J. G. (2017). Gut microbiota dysbiosis in patients with non-alcoholic fatty liver disease. *Hepatobiliary Pancreat Dis Int,* 16(4), 375-381.

Silva, Y. P., Bernardi, A., & Frozza, R. L. (2020). The Role of Short-Chain Fatty Acids From Gut Microbiota in Gut-Brain Communication. *Front Endocrinol (Lausanne),* 11, 25.

Stojanov, S., Berlec, A., & Štrukelj, B. (2020). The Influence of Probiotics on the Firmicutes/Bacteroidetes Ratio in the Treatment of Obesity and Inflammatory Bowel disease. *Microorganisms,* 8(11).

Su, H., Wang, W. J., Zheng, G. D., Yin, Z. P., Li, J. E., Chen, L. L., & Zhang, Q. F. (2022). The anti-obesity and gut microbiota modulating effects of taxifolin in C57BL/6J mice fed with a high-fat diet. *J Sci Food Agric,* 102(4), 1598-1608.

Wang, C., Hu, M., Yi, Y., Wen, X., Lv, C., Shi, M., & Zeng, C. (2022). Multiomic analysis of dark tea extract on glycolipid metabolic disorders in db/db mice. *Front Nutr,* 9, 1006517.

Wang, J., & Jia, H. (2016). Metagenome-wide association studies: fine-mining the microbiome. *Nat Rev Microbiol,* 14(8), 508-522.

Wang, N., Wu, T., Du, D., Mei, J., Luo, H., Liu, Z., . . . Zhu, H. (2021). Transcriptome and Gut Microbiota Profiling Revealed the Protective Effect of Tibetan Tea on Ulcerative Colitis in Mice. *Front Microbiol,* 12, 748594.

Wang, Y., Yu, W., Li, S., Guo, D., He, J., & Wang, Y. (2022). Acetyl-CoA Carboxylases and

For Review Only

Diseases. *Front Oncol,* 12, 836058.

Wiciński, M., Gębalski, J., Gołębiewski, J., & Malinowski, B. (2020). Probiotics for the Treatment of Overweight and Obesity in Humans-A Review of Clinical Trials. *Microorganisms,* 8(8).

Wu, M., Yang, S., Wang, S., Cao, Y., Zhao, R., Li, X., . . . Liu, L. (2020). Effect of Berberine on Atherosclerosis and Gut Microbiota Modulation and Their Correlation in High-Fat Diet-Fed ApoE-/- Mice. *Front Pharmacol,* 11, 223.

Wu, Y., Sun, H., Yi, R., Tan, F., & Zhao, X. (2021). Anti-obesity effect of Liupao tea extract by modulating lipid metabolism and oxidative stress in high-fat-diet-induced obese mice. *J Food Sci,* 86(1), 215-227.

Xie, H., Li, X., Ren, Z., Qiu, W., Chen, J., Jiang, Q., . . . Chen, D. (2018). Antioxidant and Cytoprotective Effects of Tibetan Tea and Its Phenolic Components. *Molecules,* 23(2).

Xu, J., Wang, M., Zhao, J., Wang, Y. H., Tang, Q., & Khan, I. A. (2018). Yellow tea (Camellia sinensis L.), a promising Chinese tea: Processing, chemical constituents and health benefits. *Food Res Int,* 107, 567-577.

Yang, J. W., & Kim, S. S. (2015). Ginsenoside Rc promotes anti-adipogenic activity on 3T3-L1 adipocytes by down-regulating C/EBPα and PPARγ. *Molecules,* 20(1), 1293-1303.

Ye, J., Zhao, Y., Chen, X., Zhou, H., Yang, Y., Zhang, X., . . . Xiao, M. (2021). Pu-erh tea ameliorates obesity and modulates gut microbiota in high fat diet fed mice. *Food Res Int,* 144, 110360.

Yuan, Y., Zhang, B., Lu, X., Chen, D., Li, F., Yao, Y., & Peng, Y. (2016). Study on anti-obese and hypolipidemic effects of Ya'an Tibetan Tea in rats with dyslipidemias. *Pharmacology and Clinics of Chinese Materia Medica*.

Zaky, A., Glastras, S. J., Wong, M. Y. W., Pollock, C. A., & Saad, S. (2021). The Role of the Gut Microbiome in Diabetes and Obesity-Related Kidney Disease. *Int J Mol Sci,* 22(17).

Zhang, C., Zhang, M., Wang, S., Han, R., Cao, Y., Hua, W., . . . Zhao, L. (2010). Interactions between gut microbiota, host genetics and diet relevant to development of metabolic syndromes in mice. *Isme j,* 4(2), 232-241.

Zhang, X. Y., Chen, J., Yi, K., Peng, L., Xie, J., Gou, X., . . . Tang, L. (2020). Phlorizin ameliorates obesity-associated endotoxemia and insulin resistance in high-fat diet-fed mice by targeting the gut microbiota and intestinal barrier integrity. *Gut Microbes,* 12(1), 1-18.

Zhang, Y., Yang, L., Zhao, N., Hong, Z., Cai, B., Le, Q., . . . He, J. (2021). Soluble Polysaccharide Derived from Laminaria japonica Attenuates Obesity-Related Nonalcoholic Fatty Liver Disease Associated with Gut Microbiota Regulation. *Mar Drugs,* 19(12).

Zhao, H., Lyu, Y., Zhai, R., Sun, G., & Ding, X. (2022). Metformin Mitigates Sepsis-Related Neuroinflammation via Modulating Gut Microbiota and Metabolites. *Front Immunol,* 13, 797312.

Zheng, L., Kelly, C. J., Battista, K. D., Schaefer, R., Lanis, J. M., Alexeev, E. E., . . . Colgan, S. P. (2017). Microbial-Derived Butyrate Promotes Epithelial Barrier Function through IL-10 Receptor-Dependent Repression of Claudin-2. *J Immunol,* 199(8), 2976-2984.

For Review Only

Zheng, Q., Li, W., Zhang, H., Gao, X., & Tan, S. (2020). Optimizing synchronous extraction and antioxidant activity evaluation of polyphenols and polysaccharides from Ya'an Tibetan tea (Camellia sinensis). *Food Sci Nutr,* 8(1), 489-499.

Zhu, X., Bian, H., Wang, L., Sun, X., Xu, X., Yan, H., . . . Gao, X. (2019). Berberine attenuates nonalcoholic hepatic steatosis through the AMPK-SREBP-1c-SCD1 pathway. *Free Radic Biol Med,* 141, 192-204.

Ziętak, M., Kovatcheva-Datchary, P., Markiewicz, L. H., Ståhlman, M., Kozak, L. P., & Bäckhed, F. (2016). Altered Microbiota Contributes to Reduced Diet-Induced Obesity upon Cold Exposure. *Cell Metab,* 23(6), 1216-1223.

For Review Only

Table 1. Profiles of chemical components of TT water extracts.

Components	Content (mg/g)
Tea polyphenols	596.91±4.24
Total flavonoids	83.60±1.90
Soluble sugar	179.43±3.35
GA	79.69±2.91
EGC	11.20±2.00
Catechin	10.40±1.10
Caffeine	95.50±0.40
EC	4.45±0.05
EGCG	no detected

Data are expressed as mean ± SD(n = 6). GA, gallic acid; EGC, (-)-epigallocatechin; EC,(-)-epicatechin; EGCG, epigallocatechin gallate.

For Review Only

Table 2. Effects of TT on metabolic syndrome in HFD-fed mice.

Parameters	NCD	NCD_TT_H	HFD	HFD_TT_L	HFD_TT_M	HFD_TT_H
TC (mmol/L)	3.12±0.14[e]	2.37±0.16[f]	5.02±0.07[a]	4.68±0.01[b]	4.29±0.22[c]	3.79±0.11[d]
TG (mmol/L)	0.73±0.04[c]	0.54±0.022[f]	1.20±0.02[a]	0.99±0.04[b]	0.65±0.03[d]	0.58±0.02[e]
LDL-C (mmol/L)	1.45±0.18[c]	0.57±0.03[d]	2.56±0.20[a]	1.87±0.24[b]	0.69±0.10[d]	0.52±0.03[d]
HDL-C (mmol/L)	3.66±0.05[c]	5.28±0.54[a]	2.72±0.05[e]	3.12±0.05[d]	4.20±0.05[b]	5.51±0.14[a]

NCD: normal chow diet; NCD_TT_H: normal chow diet with 400mg/kg of TT extract; HFD: high-fat diet; HFD_TT_L: high-fat diet with 100mg/kg of TT extract; HFD_TT_M: high-fat diet with 200mg/kg of TT extract; HFD_TT_H: high-fat diet with 400mg/kg of TT extract. Significant differences were evaluated by one-way analysis of variance (ANOVA). Comparison of each parameter between the six groups, with different superscript letters indicating significant differences ($P < 0.05$).

For Review Only

Table 3. Fecal SCFAs contents of mice.

SCFAs (μg/mg)	NCD	NCD_TT_H	HFD	HFD_TT_L	HFD_TT_M	HFD_TT_H
Acetic acid	0.87±0.06[b]	1.07±0.12[a]	0.53±0.01[e]	0.57±0.01[de]	0.63±0.07[cd]	0. 70±0. 04[c]
Propionic acid	0.36±0.07[b]	0.66±0.11[a]	0.08±0.00[d]	0.11±0.01[d]	0.14±0.02[cd]	0. 20±0. 03[c]
Isobutyric acid	0.18±0.02[b]	0.22±0.01[a]	0.05±0.00[f]	0.08±0.01[e]	0.11±0.02[d]	0. 16±0. 02[c]
Butyric acid	0.29±0.02[b]	0.43±0.07[a]	0.06±0.00[d]	0.06±0.00[d]	0.08±0.00[cd]	0. 10±0. 01[c]
Isovaleric acid	0.05±0.01[a]	0.04±0.01[a]	0.04±0.01[a]	0.05±0.01[a]	0.06±0.00[a]	0. 05±0. 01[a]
Valeric acid	0.02±0.01[a]	0.02±0.01[a]	0.02±0.01[a]	0.02±0.00[a]	0.02±0.01[a]	0.03±0.01[a]

Data are expressed as mean ± SD (n = 6). Significant differences were evaluated by one-way analysis of variance (ANOVA). Comparison of each parameter between the six groups, with different superscript letters indicating significant differences ($P < 0.05$).

For Review Only

Table 4. The effect of faecal transplantation on metabolic syndrome in HFD-fed mice.

Parameters	NCD_HFD	NCD_H_HFD	HFD_HFD	HFD_H_HFD
Fasting Glucose（mmol/L)	7.87±0.47[b]	6.86±0.50[c]	9.10±0.07[a]	6.78±0.60[c]
TC (mmol/L)	4.50±0.14[b]	1.56±0.10[c]	5.63±1.20[a]	2.01±0.13[c]
TG (mmol/L)	1.21±0.10[b]	0.39±0.02[d]	2.07±0.12[a]	0.85±0.09[c]
LDL-C (mmol/L)	1.33±0.20[b]	0.59±0.06[d]	2.31±0.06[a]	0.93±0.05[c]
HDL-C (mmol/L)	3.84±0.07[b]	5.45±0.07[a]	2.07±0.06[c]	3.94±0.10[b]

Data are expressed as mean ± SD (n = 6). Significant differences were evaluated by one-way analysis of variance (ANOVA). Comparison of each parameter between the four groups, with different superscript letters indicating significant differences ($P < 0.05$).

For Review Only

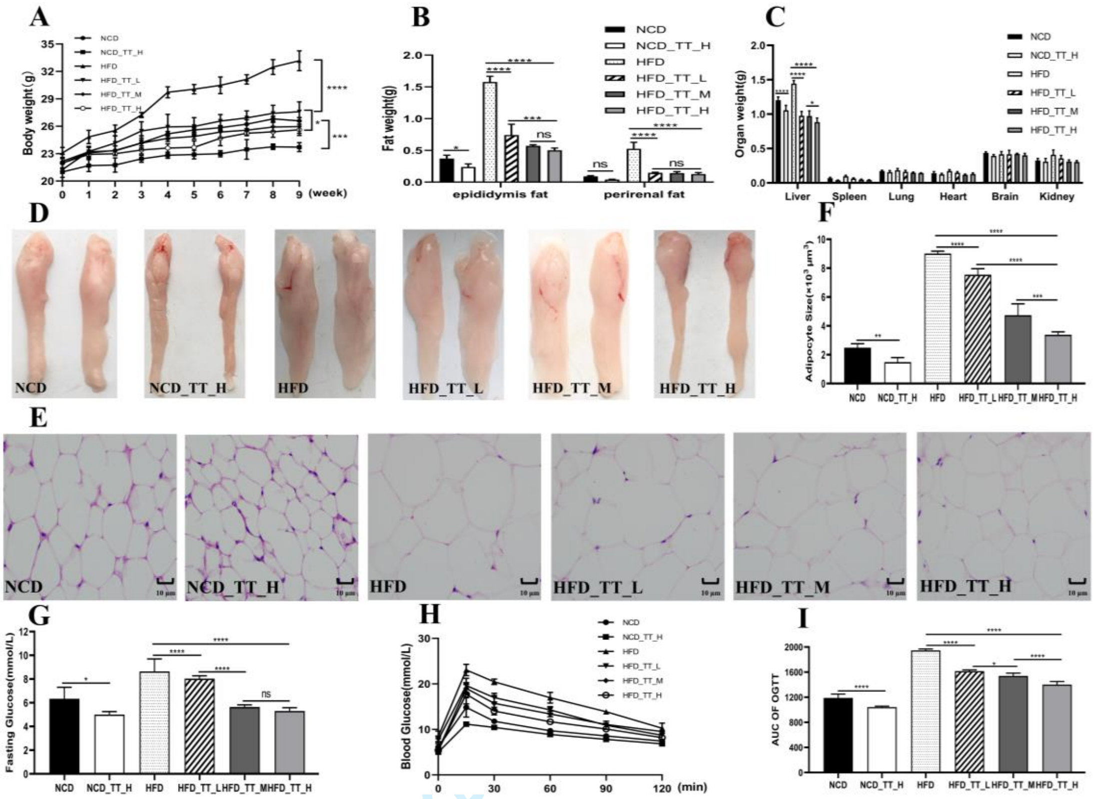

Figure 1. **TT attenuated HFD-induced obesity in mice.** (A) Body weight. (B) Weight of epididymal fat and perirenal fat. (C)Weight of different organs. (D)Morphology of the epididymis fat. (E) Morphology of adipocytes. (F) Epididymal adipocyte size. (G)Fasting glucose. (H)Oral glucose tolerance test. (I)Area under the curve (AUC) of oral glucose tolerance test. Differences between groups were assessed using a one-way ANOVA (ns for $P > 0.05$, $*P < 0.05$, $**P < 0.01$, $***P < 0.001$ and $****P < 0.0001$).

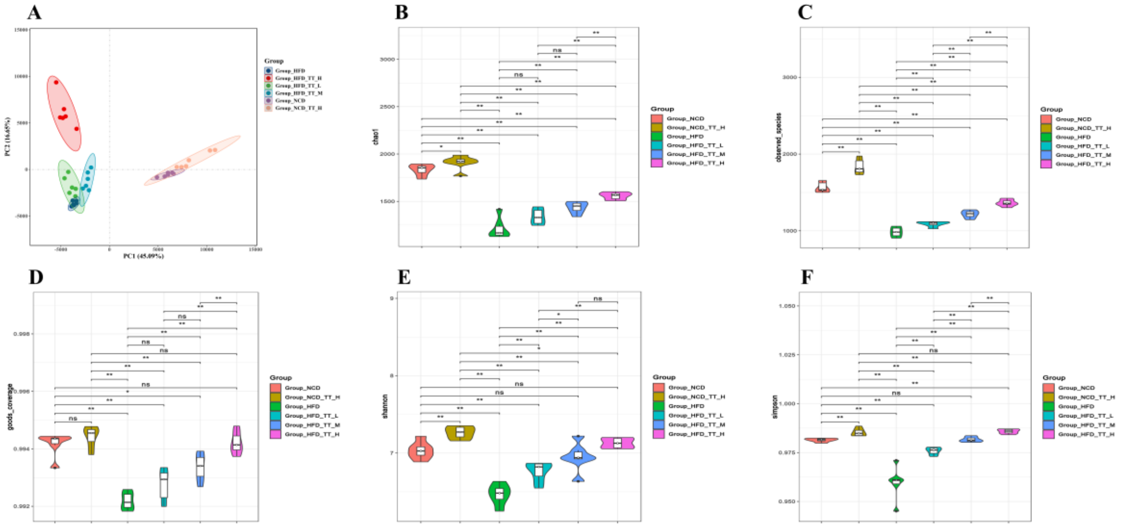

Figure 2. TT modulated the structure and diversity of the faecal microbiota. (A) principal components analysis (PCoA) plot. Alpha Diversity-related boxplot analysis, including (B) chao1, (C) observed_species, (D) goods_coverage, (E) Shannon index, (F) Simpson index. Differences between groups were assessed using a one-way ANOVA (ns for $P > 0.05$, $*P < 0.05$, $**P < 0.01$, $***P < 0.001$ and $****P < 0.0001$).

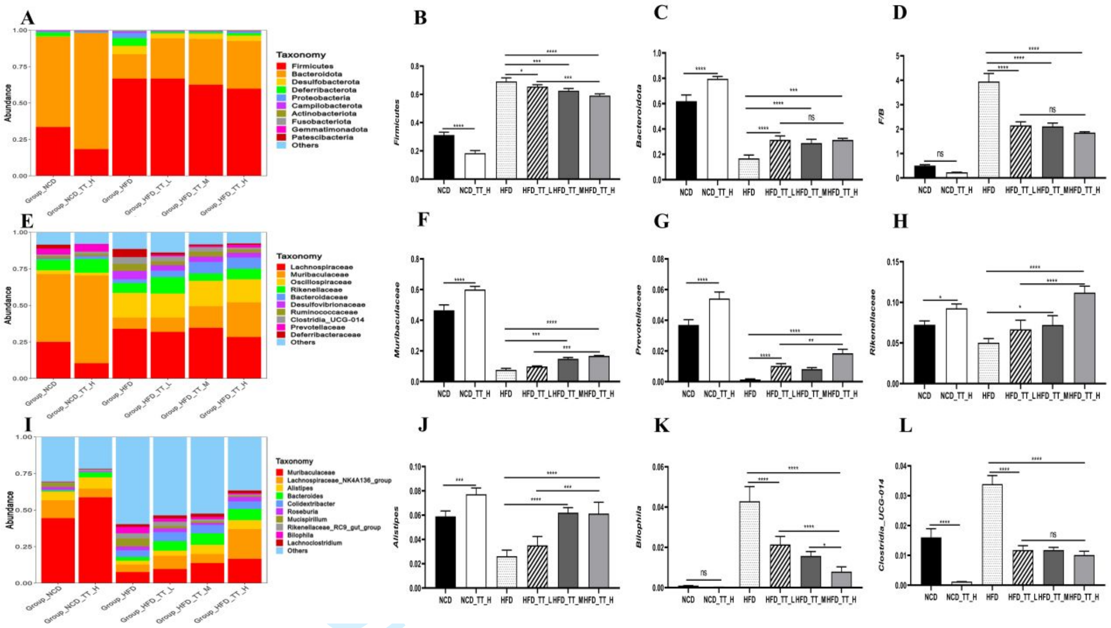

Figure 3. TT modulated the structure of the faecal microbiota in mice. Phylum-(A), family-(E), and genus-level(I) distribution of faecal microbiota. Relative abundance of the phyla *Firmicutes* (B) and *Bacteroidota* (C); (D)Relative population abundance ratio of *Firmicutes* and *Bacteroidota*; (F-H, J-L) relative abundance of some microorganisms at the family and genus levels. Differences between groups were assessed using a one-way ANOVA (ns for P >0.05, *P <0.05, **P <0.01, ***P <0.001 and ****P <0.0001).

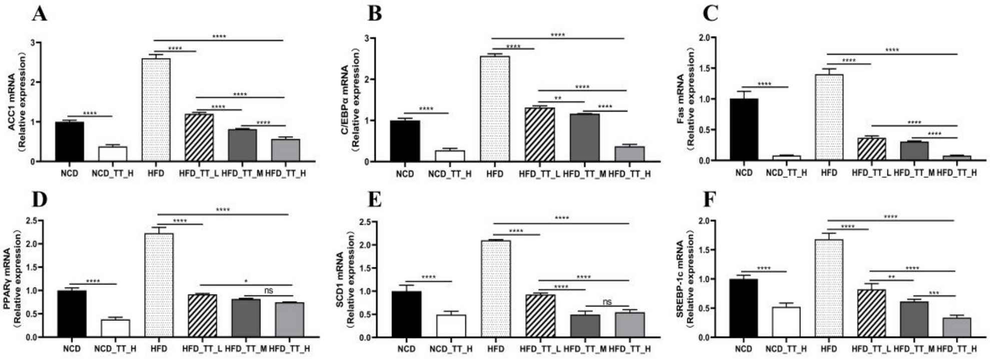

Figure 4. TT regulated the expression of genes related to lipid synthesis. Relative expression of (A)ACC1, (B)C/EBPα, (C)Fas， (D)PPARγ, (E)SCD1, and (F)SREBP-1c in the epididymal fat compared to the *NCD* group. Differences between groups were assessed using a one-way ANOVA (ns for P >0.05, *P <0.05, **P <0.01, ***P <0.001 and ****P <0.0001).

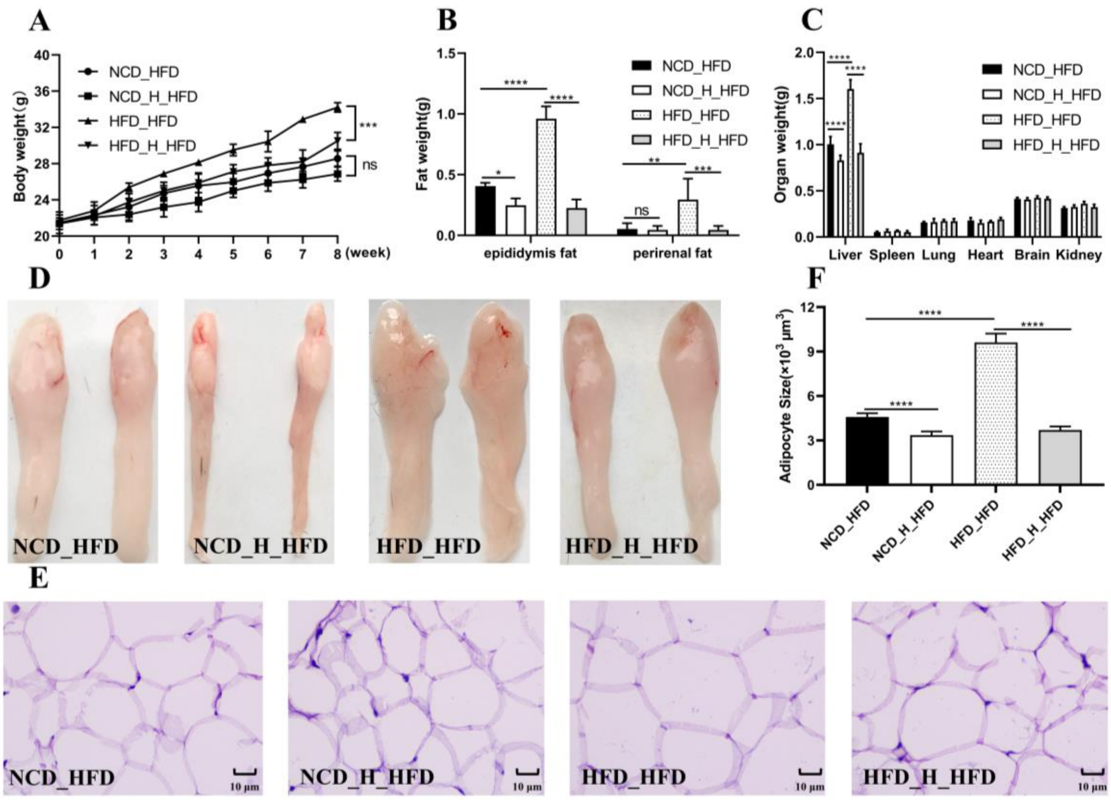

Figure 5. FMT attenuated fat accumulation in obese mice. (A) Body weight. (B) Epididymal fat and perirenal fat weight. (C)Weight of different organs. (D) Morphological observations of the epididymis fat. (E)Hematoxylin and eosin staining of epididymis fat. (F) Epididymal adipocyte size. Differences between groups were assessed using a one-way ANOVA (ns for $P > 0.05$, $*P < 0.05$, $**P < 0.01$, $***P < 0.001$ and $****P < 0.0001$).

Review Only

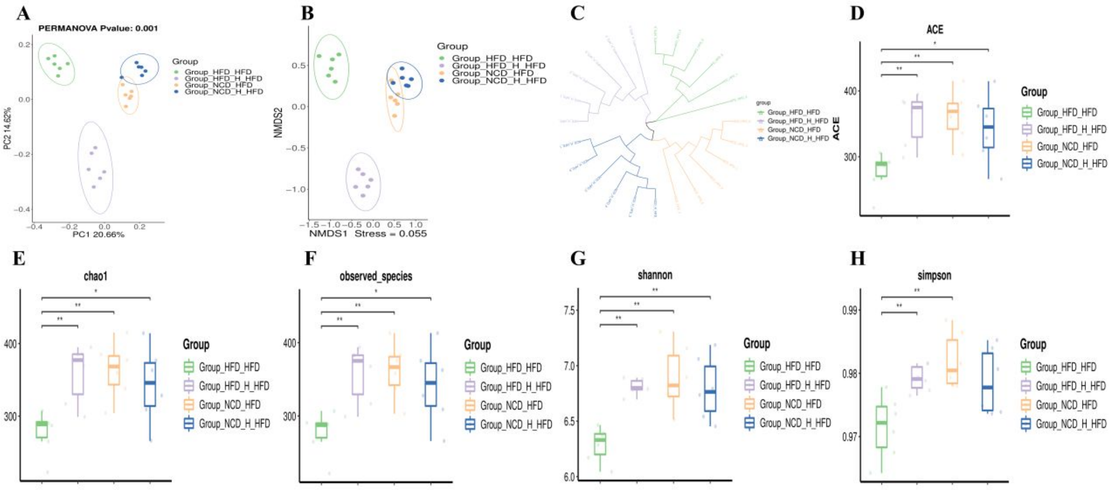

Figure 6. FMT modulated the diversity and structure of the faecal microbiota in mice. Beta diversity analysis includs (A) PCoA, (B) non-metric multidimensional scaling (NMDS) analysis, and (C)hierarchical clustering. Alpha Diversity-related boxplot analysis includs(D) ACE, (E) Chao 1, (F) Observed-species, (G)Shannon index, (H)Simpson index. Differences between groups were assessed using a one-way ANOVA (ns for *P* >0.05, **P* <0.05, ***P* <0.01, ****P* <0.001 and *****P* <0.0001).

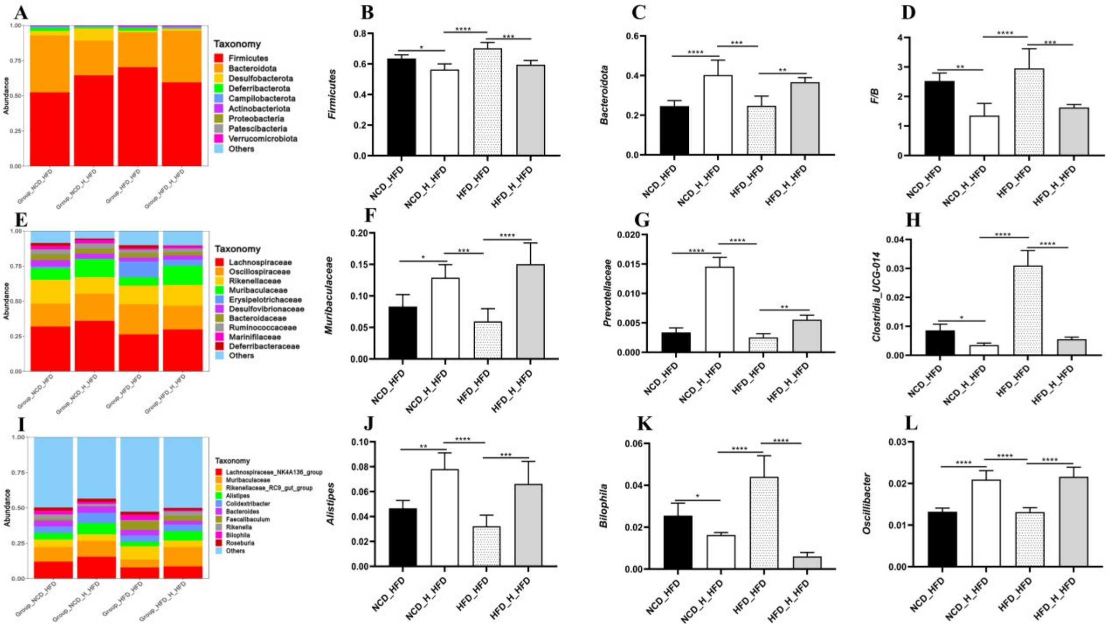

Figure 7. FMT regulated the structure of the faecal microbiota. Phylum-(A), family-(E), and genus-level(I) distribution of faecal microbiota. Relative abundance of the phyla *Firmicutes*(B) and *Bacteroidota*(C); (D)Relative population abundance ratio of *Firmicutes* and *Bacteroidota*; (F-H, J-L) relative abundance of some microorganisms at the family and genus levels. Differences between groups were assessed using a one-way ANOVA (ns for *P* >0.05, **P* <0.05, ***P* <0.01, ****P* <0.001 and *****P* <0.0001).

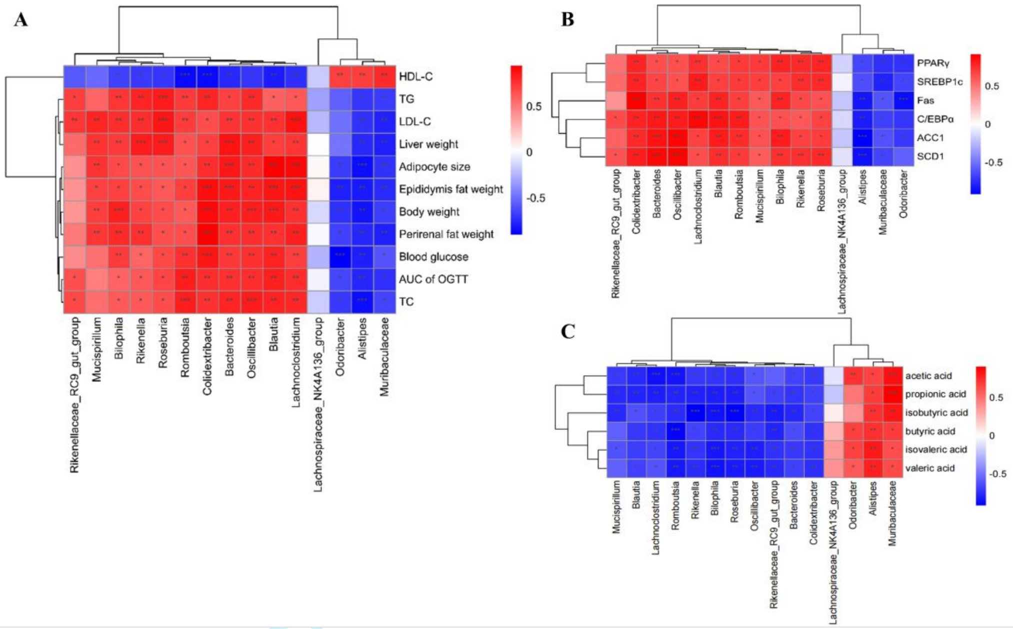

Figure 8. Heatmap of Spearman' s correlation between gut microbiota relative abundances and obesity-related indexes, SCFAs and genes related to lipid synthesis in mice altered by HFD or TT intervention. (A), (B) and (C) represent the correlation analysis between obesity-related indexes, SCFAs and genes related to lipid synthesis and the top 15 most abundant genera of gut microbes, respectively. The red and blue blocks represent positive and negative correlations, respectively. Significant correlations are presented as **P* < 0.05, ***P* < 0.01, and ****P* < 0.001 (n=6).

For Review Only

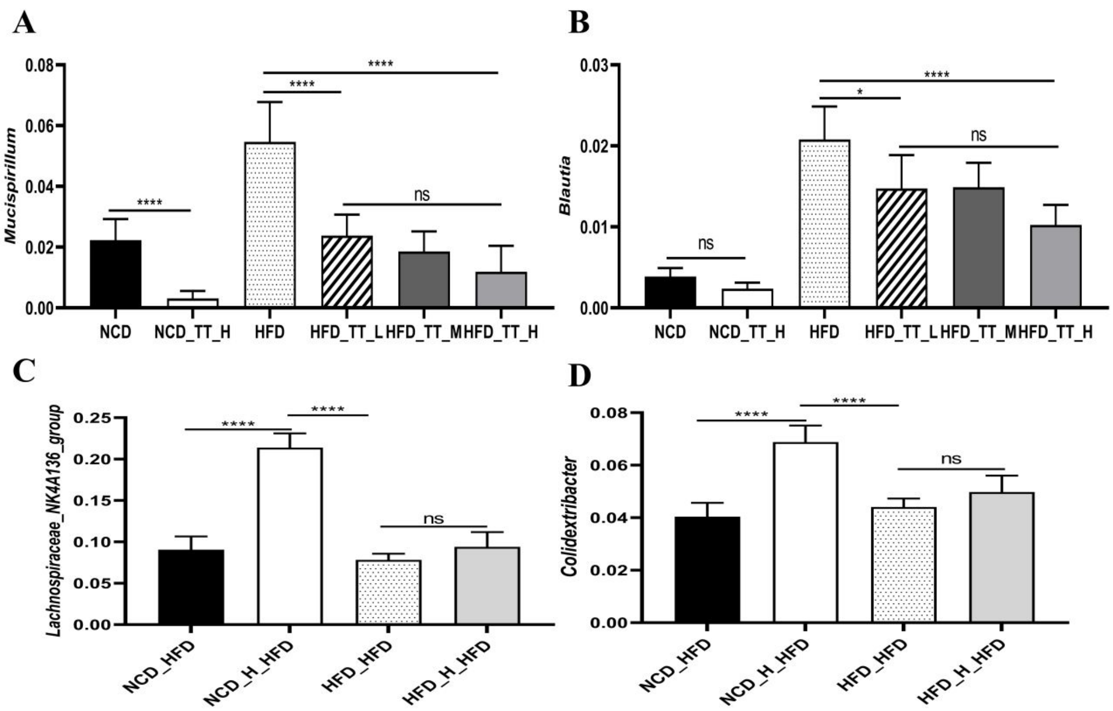

Supplementary Figure 1. A, B, C and D are the relative population abundances of *Mucispirillum*, *Blautia*, *Lachnospiraceae_NK4A136_group* and *Colidextribacter* respectively. Differences between groups were assessed using a one-way ANOVA (ns for *P* >0.05, **P* <0.05, ***P* <0.01, ****P* <0.001 and *****P* <0.0001).

Review Only

Empower™ 3 SOFTWARE

230619

SAMPLE INFORMATION

Sample Name:	Mixture Standard	Acquired By:	System
Sample Type:	Standard	Sample Set Name	230411
Vial:	79	Acq. Method Set:	CTC
Injection #:	1	Processing Method	230619
Injection Volume:	10.00 ul	Channel Name:	PDA Ch1 278nm@4.8nm
Run Time:	40.0 Minutes	Proc. Chnl. Descr.:	PDA Ch1 278nm@4.8nm
Date Acquired:	2023/4/11 12:49:11 CST		
Date Processed:	2023/6/19 13:02:06 CST		

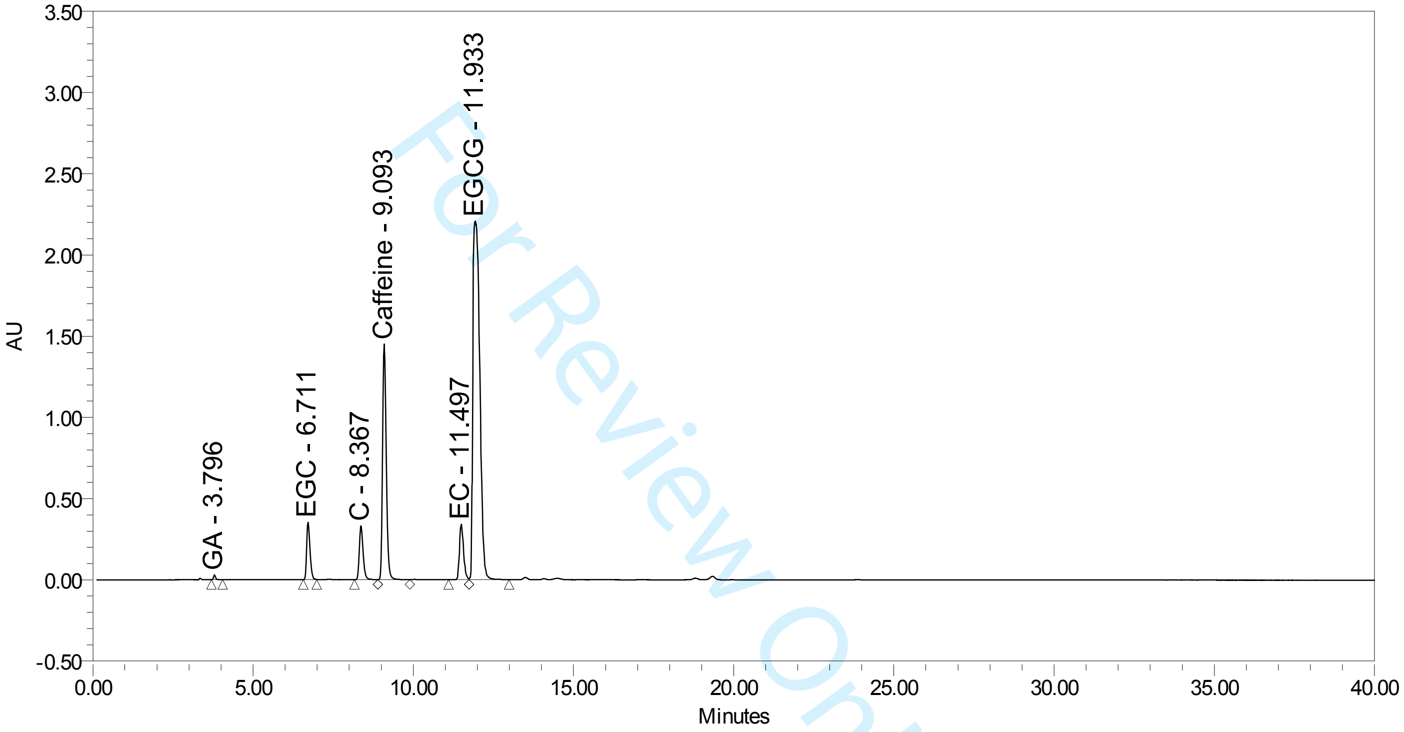

For Review Only

	Peak Name	RT	Area	Height
1	GA	3.796	136294.61	29507
2	EGC	6.711	2587467.37	353714
3	C	8.367	2788000.70	332577
4	Caffeine	9.093	11720855.50	1451995
5	EC	11.497	3070887.98	342346
6	EGCG	11.933	32871243.09	2207231

Empower™ 3 SOFTWARE

230619

SAMPLE INFORMATION

Sample Name:	TT E	Acquired By:	System
Sample Type:	Unknown	Sample Set Name	230411
Vial:	84	Acq. Method Set:	CTC
Injection #:	1	Processing Method	230619
Injection Volume:	10.00 ul	Channel Name:	PDA Ch1 278nm@4.8nm
Run Time:	40.0 Minutes	Proc. Chnl. Descr.:	PDA Ch1 278nm@4.8nm
Date Acquired:	2023/4/11 13:33:39 CST		
Date Processed:	2023/6/19 12:58:59 CST		

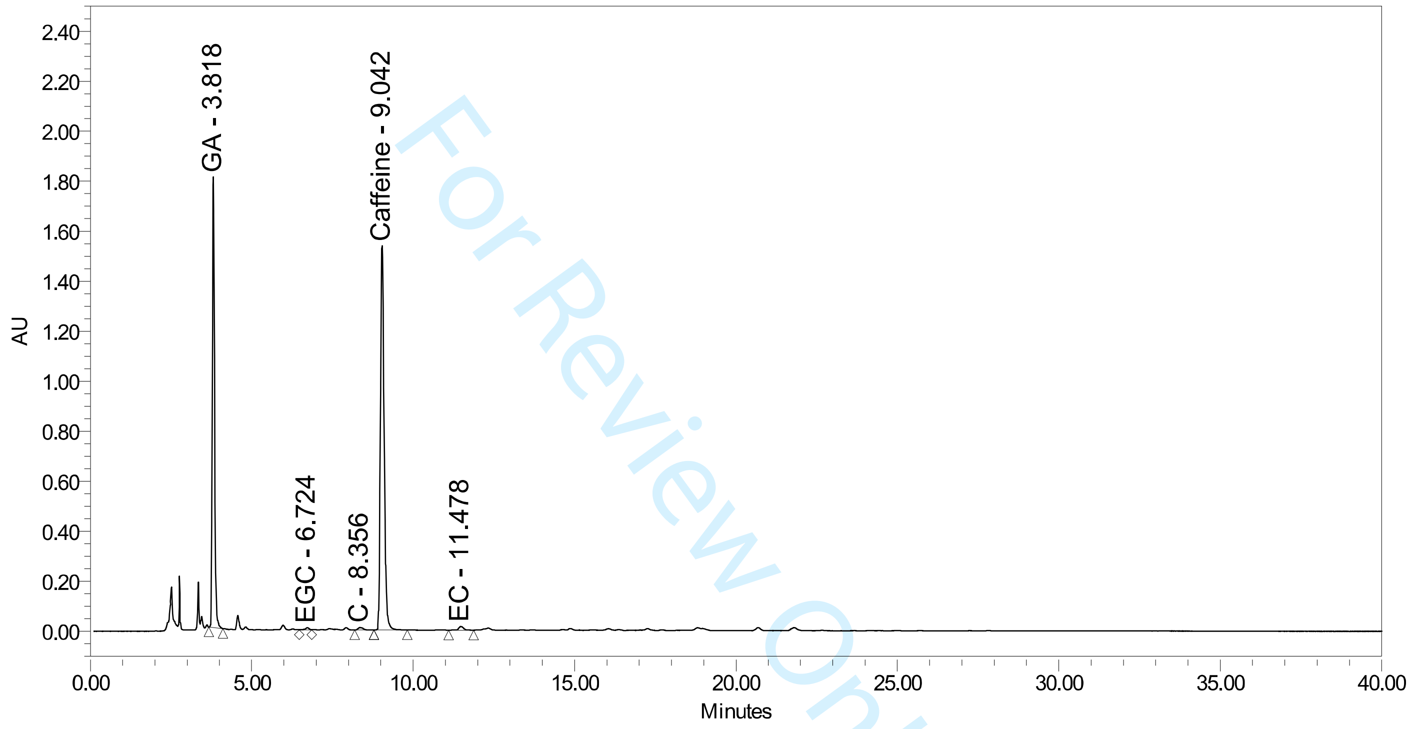

For Review Only

	Peak Name	RT	Area	Height
1	GA	3.818	8676221.86	1801995
2	EGC	6.724	77266.65	8793
3	C	8.356	114310.53	10300
4	Caffeine	9.042	12375579.65	1536906
5	EC	11.478	160666.57	15321

Reported by User: System
Report Method: 230619
Report Method ID 8151
Page: 1 of 1

Project Name: test
Date Printed:
2023/6/19
13:00:29 PRC

Supplementary Table 1 Identification of chemical constituents of Tibetan tea extracts by HPLC-MS/M

Checked	Name	Formula	Annotation S	Annotation S
TRUE	Caffeine	C8 H10 N4 C	Full match	Full match
TRUE	Gallic acid	C7 H6 O5	No results	Invalid mass
TRUE	Gallic acid	C7 H6 O5	No results	Invalid mass
TRUE	Gallic acid	C7 H6 O5	No results	Invalid mass
TRUE	Palmitic Acid	C16 H32 O2	No match	Invalid mass
TRUE	Lorcaserin	C11 H14 Cl N	No results	Invalid mass
TRUE	Tropine	C8 H15 N O	Full match	Full match
TRUE			Unused	No results
TRUE	(-)-Gallocatechin	C15 H14 O7	Full match	No results
TRUE			No results	No results
TRUE	Epigallocatechin gallate	C22 H18 O1	Not the top	Full match
TRUE	Betaine	C5 H11 N O2	Full match	Full match
TRUE	Gallic acid	C7 H6 O5	No results	Invalid mass
TRUE	Marginatoside	C33 H40 O2	Not the top	No results
TRUE			Unused	No results
TRUE			Unused	No results
TRUE			Unused	No results
TRUE			Unused	No results
TRUE	Eplerenone	C24 H30 O6	Full match	No results
TRUE	Pyrogallol	C6 H6 O3	No results	Invalid mass
TRUE	Corymboside	C26 H28 O1	Not the top	Full match
TRUE	1-Linoleoylglycerophosphocholine	C26 H50 N C	Full match	No results
TRUE	Theobromine	C7 H8 N4 O2	Full match	Full match
TRUE	Tropine	C8 H15 N O	Full match	Full match
TRUE	Epigallocatechin (-)	C15 H14 O7	Full match	No results
TRUE	Valpromide	C8 H17 N O	Full match	Full match
TRUE			Unused	No results
TRUE	D-Pipecolicacid	C6 H11 N O2	Full match	No results
TRUE			No results	No results
TRUE	Adenine	C5 H5 N5	Full match	Full match
TRUE	Adenine	C5 H5 N5	No match	Invalid mass
TRUE	1-Palmitoylglycerophosphocholine	C24 H50 N C	Full match	No results
TRUE			Unused	No results
TRUE			Unused	No results
TRUE	Valpromide	C8 H17 N O	Full match	Full match
TRUE			Unused	No results
TRUE			No results	No results
TRUE			Unused	No results
TRUE	Diphenylphosphine oxide	C12 H11 O P	No match	No results
TRUE	8-Hydroxyluteolin 8-glucoside-3'-rutinoside	C33 H40 O2	Not the top	No results
TRUE			Unused	No results
TRUE			No results	No results
TRUE	Valpromide	C8 H17 N O	Full match	No results
TRUE	Vitexin 7-O-sulfate	C21 H20 O1	No match	No results
TRUE			Unused	No results
TRUE	Citric acid	C6 H8 O7	No results	Invalid mass
TRUE	1,5-Anhydro-1-[5,7-dihydroxy-2-(4-hydroxyphenyl)-4	C21 H20 O1	Not the top	Full match
TRUE	(2S,4R,5S,6S,7R)-5,6,12,14-tetrahydroxy-4-(hydroxym	C14 H16 O9	No match	Invalid mass

TRUE	Adenine	C5 H5 N5	Full match	Full match
TRUE	Adenine	C5 H5 N5	No match	Invalid mass
TRUE			No results	No results
TRUE			Unused	No results
TRUE			Unused	No results
TRUE			No results	No results
TRUE	gamma-Glutamyl-gamma-aminobutyraye	C9 H16 N2 C	Full match	No results
TRUE	Triethyl phosphate	C6 H15 O4 P	Full match	Full match
TRUE			Unused	No results
TRUE			Unused	No results
TRUE	Gallic acid	C7 H6 O5	No results	Invalid mass
TRUE			Unused	No results
TRUE			No results	No results
TRUE			Unused	No results
TRUE	6-O-(beta-D-Xylopyranosyl)-beta-D-glucopyranose	C11 H20 O1(	Not the top	No results
TRUE			Unused	No results
TRUE			No results	No results
TRUE			Unused	No results
TRUE	sn-glycero-3-Phosphocholine	C8 H20 N O6	Full match	No results
TRUE			Unused	No results
TRUE			Unused	No results
TRUE	3-Methylcrotonylglycine	C7 H11 N O3	Full match	Full match
TRUE			Unused	No results
TRUE			Unused	No results
TRUE	4-Hydroxyphenylacetylglutamicacid	C13 H15 N C	Full match	No results
TRUE			Unused	No results
TRUE	6-Gingerol	C17 H26 O4	Full match	No results
TRUE	1-Oleoylglycerophosphocholine	C26 H52 N C	Full match	No results
TRUE	Gentisic acid	C7 H6 O4	No results	Invalid mass
TRUE	Theobromine	C7 H8 N4 O2	Full match	Full match
TRUE			Unused	No results
TRUE	4-Oxoproline	C5 H7 N O3	No results	Invalid mass
TRUE			Unused	No results
TRUE	Procyanidin B1	C30 H26 O12	Not the top	No results
TRUE	1,8,15,22-Tetraazacyclooctacosane-2,9,16,23-tetrone	C24 H44 N4	Full match	No results
TRUE			Unused	No results
TRUE	3-{[(2S,3R,4S,5R,6R)-3,5-dihydroxy-6-(hydroxymethyl	C27 H30 O15	Full match	Full match
TRUE	2-Amino-1,3,4-octadecanetriol	C18 H39 N C	Full match	Full match
TRUE	myricetin 3-O-beta-D-galactopyranoside	C21 H20 O13	Not the top	Full match
TRUE			Unused	No results
TRUE	(2S,3R,4S,5R)-2-{[(2R,3R,4S,5S,6R)-4,5-dihydroxy-6-(h	C19 H28 O1(	Full match	Full match
TRUE			Unused	No results
TRUE	Theophylline	C7 H8 N4 O2	No match	Invalid mass
TRUE			Unused	No results
TRUE	2-[(1-benzothiophen-3-ylmethyl)amino]-1-phenyleth	C17 H17 N C	No match	Invalid mass
TRUE	2-[(1-benzothiophen-3-ylmethyl)amino]-1-phenyleth	C17 H17 N C	No match	Invalid mass
TRUE	2-[(1-benzothiophen-3-ylmethyl)amino]-1-phenyleth	C17 H17 N C	No match	Invalid mass
TRUE	2-[(1-benzothiophen-3-ylmethyl)amino]-1-phenyleth	C17 H17 N C	No match	Invalid mass
TRUE	trans-3-Indoleacrylic acid	C11 H9 N O2	No results	Full match
TRUE	D-(+)-Tryptophan	C11 H12 N2	Full match	Full match

TRUE	Vitexin 7-O-sulfate	C21 H20 O13	Not the top	No results
TRUE	Ellagic acid	C14 H6 O8	Full match	No results
TRUE			Unused	No results
TRUE	Prolylleucine	C11 H20 N2	No match	Invalid mass
TRUE	all-trans-Heptaprenyldiphosphate	C35 H60 O7	No match	No results
TRUE			Unused	No results
TRUE	3-Methylcrotonylglycine	C7 H11 N O3	Full match	Full match
TRUE	Gentisic acid	C7 H6 O4	No results	Invalid mass
TRUE			Unused	No results
TRUE			Unused	No results
TRUE			Unused	No results
TRUE	Choline	C5 H13 N O	Full match	Full match
TRUE			Unused	No results
TRUE	Adenosine	C10 H13 N5	Full match	Full match
TRUE	NP-000921	C15 H10 O8	Full match	Full match
TRUE			Unused	No results
TRUE	gamma-Glutamyl-gamma-aminobutyraldehyde	C9 H16 N2 C	Full match	No results
TRUE			Unused	No results
TRUE	Glutaconic acid	C5 H6 O4	No results	Invalid mass
TRUE			Unused	No results
TRUE			Unused	No results
TRUE	Prolylleucine	C11 H20 N2	Full match	Full match
TRUE			Unused	No results
TRUE			Unused	No results
TRUE	Rutin	C27 H30 O16	Not the top	Full match
TRUE			Unused	No results
TRUE			Unused	No results
TRUE			Unused	No results
TRUE	Vicenin II	C27 H30 O15	Full match	No results
TRUE	gamma-Glutamyl-gamma-aminobutyraye	C9 H16 N2 C	Full match	No results
TRUE			Unused	No results
TRUE			Unused	No results
TRUE	2,2'-Methylenebis(4-methyl-6-tert-butylphenol)	C23 H32 O2	Full match	Full match
TRUE	3-[(Carboxycarbonyl)amino]-L-alanine	C5 H8 N2 O5	No match	No results
TRUE			Unused	No results
TRUE	Arachidic Acid	C20 H40 O2	No match	Invalid mass
TRUE	Meperidine-d5	C15 H16 [2]H	No match	Invalid mass
TRUE			Unused	No results
TRUE			Unused	No results
TRUE	(2S,3R,4S,5R)-2-{[(2R,3R,4S,5S,6R)-4,5-dihydroxy-6-(h	C19 H28 O10	No match	Invalid mass
TRUE			Unused	No results
TRUE	4-Dodecylbenzenesulfonic acid	C18 H30 O3	Full match	Full match
TRUE	Propachlor	C11 H14 Cl N	No results	No results
TRUE			Unused	No results
TRUE			Unused	No results
TRUE			Unused	No results
TRUE	Methyl gallate	C8 H8 O5	No results	No results
TRUE			Unused	No results
TRUE			Unused	No results
TRUE			Unused	No results

TRUE	4-Oxoproline	C5 H7 N O3	No results	Invalid mass
TRUE	6-(2-hydroxy-3-methyl-3-{[(2S,3R,4S,5S,6R)-3,4,5-trih	C22 H30 O1:	No match	Invalid mass
TRUE	Stearamide	C18 H37 N C	Full match	Full match
TRUE			Unused	No results
TRUE			Unused	No results
TRUE	5,7-dihydroxy-2-(4-hydroxyphenyl)-6,8-bis(3,4,5-trihy	C25 H26 O1:	Not the top	Full match
TRUE			Unused	No results
TRUE	L-Phenylalanine	C9 H11 N O2	No match	Invalid mass
TRUE			Unused	No results
TRUE			Unused	No results
TRUE	L-Phenylalanine	C9 H11 N O2	Full match	Full match
TRUE	Methyl gallate	C8 H8 O5	No results	No results
TRUE			Unused	No results
TRUE	Nitrendipine	C18 H20 N2	Full match	No results
TRUE	DL-Malic acid	C4 H6 O5	No results	Invalid mass
TRUE			Unused	No results
TRUE			Unused	No results
TRUE			Unused	No results
TRUE	Theophylline	C7 H8 N4 O2	Full match	Full match
TRUE			Unused	No results
TRUE	(4-chlorophenyl)[4,6-dimethyl-3-(1H-pyrrol-1-yl)thier	C20 H15 Cl N	No match	Invalid mass
TRUE			Unused	No results
TRUE	SB236057A	C33 H34 N4	Full match	Full match
TRUE			Unused	No results
TRUE	Methylsuccinic acid	C5 H8 O4	No results	Invalid mass
TRUE			Unused	No results
TRUE			Unused	No results
TRUE	3-Vinylaniline	C8 H9 N	Full match	No results
TRUE	Stearic acid	C18 H36 O2	No match	Invalid mass
TRUE			Unused	No results
TRUE	Epicatechin	C15 H14 O6	No match	No results
TRUE			No results	No results
TRUE			Unused	No results
TRUE			Unused	No results
TRUE			No results	No results
TRUE			Unused	No results
TRUE			No results	No results
TRUE	Gluconic acid	C6 H12 O7	No match	Full match
TRUE			No results	No results
TRUE			Unused	No results
TRUE			No results	No results
TRUE			Unused	No results
TRUE			Unused	No results
TRUE			Unused	No results
TRUE	Procyanidin B2	C30 H26 O1:	No match	No results
TRUE			Unused	No results
TRUE			Unused	No results
TRUE	Ecgoninemethylester	C10 H17 N C	Full match	No results
TRUE			Unused	No results
TRUE	2-(2,4-dihydroxyphenyl)-3,5,7-trihydroxy-4H-chrome	C15 H10 O7	Full match	Full match

TRUE	Pirbuterol	C12 H20 N2	Full match	No results
TRUE			No results	No results
TRUE			Unused	No results
TRUE			Unused	No results
TRUE			Unused	No results
TRUE			No results	No results
TRUE	3-Vinyltoluene	C9 H10	Full match	No results
TRUE			No results	No results
TRUE			Unused	No results
TRUE			No results	No results
TRUE			Unused	No results
TRUE	(±)9-HpODE	C18 H32 O4	No match	Invalid mass
TRUE	Trifolin	C21 H20 O1:	Not the top	Full match
TRUE			No results	No results
TRUE	Oxaloglutarate	C7 H8 O7	Full match	No results
TRUE	NP-002999	C27 H32 O1!	No match	Invalid mass
TRUE			Unused	No results
TRUE			No results	No results
TRUE	Valpromide	C8 H17 N O	Full match	No results
TRUE			Unused	No results
TRUE	(2R,3R,4S,5S,6R)-2-{[(2E,6R)-6-hydroxy-2,6-dimethylc	C16 H28 O7	Full match	Full match
TRUE			Unused	No results
TRUE			Unused	No results
TRUE	Isoniazide	C6 H7 N3 O	Full match	No results
TRUE			Unused	No results
TRUE			Unused	No results
TRUE			Unused	No results
TRUE	Thiaminmonophosphate	C12 H18 N4	No match	No results
TRUE			Unused	No results
TRUE	FL64DBGM0001_a	C19 H22 O5	Full match	No results
TRUE	Bufexamac	C12 H17 N C	Full match	No results
TRUE	Aminocarb	C11 H16 N2	Full match	No results
TRUE	Leu-Pro(Leucyl-Proline)	C11 H20 N2	Full match	No results
TRUE			Unused	No results
TRUE	Pirbuterol	C12 H20 N2	Full match	No results
TRUE			Unused	No results
TRUE	[FAoxo_amino(6:0)]3-oxo-5S-amino-hexanoicacid	C6 H11 N O3	Full match	No results
TRUE	Uridine	C9 H12 N2 C	Full match	No results
TRUE			No results	No results
TRUE	1-O_2-O_6-O-Trigalloyl-beta-D-glucose	C27 H24 O18	Not the top	No results
TRUE			No results	No results
TRUE	Erucic amide	C22 H43 N C	Full match	No results
TRUE	Trifolin	C21 H20 O1:	Not the top	Full match
TRUE			No results	No results
TRUE	N-Methyl-N'-(4-vinylbenzyl)-1,2-ethanediamine	C12 H18 N2	Full match	No results
TRUE			Unused	No results
TRUE	N,N-Dimethyloctadecanamide	C20 H41 N C	Full match	No results
TRUE			Unused	No results
TRUE			No results	No results
TRUE			Unused	No results

TRUE			No results	No results
TRUE			Unused	No results
TRUE			Unused	No results
TRUE			Unused	No results
TRUE			Unused	No results
TRUE	Aspalathin	C21 H24 O11	Not the top	No results
TRUE			Unused	No results
TRUE	L-gamma-Glutamyl-L-hypoglycin	C12 H18 N2	Full match	No results
TRUE			Unused	No results
TRUE			No results	No results
TRUE	sn-glycero-3-Phosphocholine	C8 H20 N O6	Not the top	No results
TRUE			No results	No results
TRUE	Gallicacid	C7 H6 O5	Full match	No results
TRUE	myricetin 3-O-beta-D-galactopyranoside	C21 H20 O13	Invalid mass	Invalid mass
TRUE	12H-benzo[5,6][1,4]oxazino[2,3-b]quinoxaline	C14 H9 N3 C	No match	Invalid mass
TRUE			Unused	No results
TRUE			Unused	No results
TRUE			Unused	No results
TRUE			Unused	No results
TRUE			Unused	No results
TRUE			Unused	No results
TRUE	2-Furoylglycine;Pyromucuricacid	C7 H7 N O4	Full match	No results
TRUE	Cytidine	C9 H13 N3 C	Full match	No results
TRUE			Unused	No results
TRUE			Unused	No results
TRUE			Invalid mass	No results
TRUE			Unused	No results
TRUE			Unused	No results
TRUE			Unused	No results
TRUE	2-(Acetamidomethylene)succinate	C7 H9 N O5	No results	No results
TRUE			Unused	No results
TRUE			Unused	No results
TRUE	7-methylthioheptanaldoxime	C8 H17 N O S	No results	No results
TRUE			Unused	No results
TRUE			Unused	No results
TRUE			Unused	No results
TRUE			Unused	No results
TRUE			Unused	No results
TRUE			Unused	No results
TRUE			Unused	No results
TRUE	1-O-Galloyl-beta-D-glucose	C13 H16 O10	Not the top	No results
TRUE			Unused	No results
TRUE			Unused	No results
TRUE			Unused	No results
TRUE	1-Methyladenosine	C11 H15 N5	Full match	No results
TRUE	Mebutamate	C10 H20 N2	Full match	No results
TRUE	4-Guanidinobutanoate	C5 H11 N3 C	Full match	No results
TRUE	5-(3-Hydroxy-4-acetoxybut-1-ynyl)-2_2'-bithiophene	C14 H12 O3	Not the top	No results
TRUE			No results	No results
TRUE	3,4-DMMC	C12 H17 N C	Full match	No results

TRUE			Unused	No results
TRUE			Unused	No results
TRUE	9,10-Dihydro-10- (3,4-dihydroxyphenyl) -pyrano [2,3	C24 H20 O9	Not the top	No results
TRUE			Unused	No results
TRUE			Unused	No results
TRUE	Felbamate	C11 H14 N2	Full match	No results
TRUE	Syringin	C17 H24 O9	No match	No results
TRUE			Unused	No results
TRUE	(2R,3R)-2-(3,4-dihydroxyphenyl)-5,7-dihydroxy-3,4-di	C22 H18 O1(	No match	Full match
TRUE	2'-Deoxymugineicacid	C12 H20 N2	Not the top	No results
TRUE	?-(Methylenecyclopropyl)glycine	C6 H9 N O2	Full match	No results
TRUE	5,7,3',4',5'-Pentahydroxy-3,6-dimethoxyflavone	C17 H14 O9	Not the top	No results
TRUE			Unused	No results
TRUE			Unused	No results
TRUE			Unused	No results
TRUE	Bendiocarb	C11 H13 N C	Full match	No results
TRUE			Unused	No results
TRUE			Unused	No results
TRUE			Unused	No results
TRUE	6-(2-hydroxy-3-methyl-3-{[(2S,3R,4S,5S,6R)-3,4,5-trih	C22 H30 O1	Unused	Invalid mass
TRUE	Myricetin 3- (2''-galloylgalactoside)	C28 H24 O1	No match	No results
TRUE			No results	No results
TRUE	3,5,7-trihydroxy-2-(3-hydroxy-4-{[(2S,3R,4S,5S,6R)-3,	C21 H20 O1	Full match	Full match
TRUE			Unused	No results
TRUE			Unused	No results
TRUE			Unused	No results
TRUE			Unused	No results
TRUE			Unused	No results
TRUE			Unused	No results
TRUE			No results	No results
TRUE	Pseudouridine	C9 H12 N2 C	Full match	No results
TRUE			Unused	No results
TRUE	FL6DDAGI0001_a	C22 H26 O6	Full match	No results
TRUE	N-Acetyl-L-glutamate5-semialdehyde	C7 H11 N O4	Full match	No results
TRUE	N,N-Bis(2-hydroxyethyl)dodecanamide	C16 H33 N C	Full match	No results
TRUE			Unused	No results
TRUE			Unused	No results
TRUE			No results	No results
TRUE	p-Hydroxy-cinnamic acid	C9 H8 O3	No results	No results
TRUE			Unused	No results
TRUE	Gibberellin A17	C20 H26 O7	Not the top	No results
TRUE			Unused	No results
TRUE			Unused	No results
TRUE			Unused	No results
TRUE			Unused	No results
TRUE			Unused	No results
TRUE	2,6,3',4'-Tetrahydroxy-2-benzylcoumaranone	C15 H12 O6	Full match	No results
TRUE			Unused	No results
TRUE			Unused	No results
TRUE			No results	No results

TRUE	8'-Hydroxyabscisate	C15 H20 O5	Full match	No results
TRUE			Unused	No results
TRUE			Unused	No results
TRUE			Unused	No results
TRUE			Unused	No results
TRUE	Ohmefentanyl	C23 H30 N2	Not the top	No results
TRUE	Pongamoside B	C24 H22 O1(	No match	No results
TRUE			No results	No results
TRUE			Unused	No results
TRUE	1-(5'-Phosphoribosyl)-5-formamido-4-imidazolecarbo	C10 H15 N4	Not the top	No results
TRUE			Unused	No results
TRUE	2-oxo-1_2-dihydroquinoline-4-carboxylate	C10 H7 N O3	Full match	No results
TRUE			Unused	No results
TRUE	2(alpha-D-Mannosyl)-D-glycerate	C9 H16 O9	Full match	No results
TRUE	Cerarvensin	C20 H18 O9	Not the top	No results
TRUE			Unused	No results
TRUE			Unused	No results
TRUE	Adenosine2'-phosphate	C10 H14 N5	Not the top	No results
TRUE	Saccharopine	C11 H20 N2	Full match	No results
TRUE	Camphor	C10 H16 O	Full match	No results
TRUE	Amaronol A	C15 H12 O8	Not the top	No results
TRUE	Thiaminmonophosphate	C12 H18 N4	Invalid mass	No results
TRUE	Quercetin 3-sophorotrioside	C33 H40 O2:	Full match	No results
TRUE	Protocatechuic acid	C7 H6 O4	No results	No results
TRUE			Unused	No results
TRUE	3-Vinyl-2-pyrrolidinone	C6 H9 N O	Full match	No results
TRUE			Unused	No results
TRUE			No results	No results
TRUE	Protocatechuic acid	C7 H6 O4	No results	No results
TRUE			Unused	No results
TRUE			No results	No results
TRUE			Unused	No results
TRUE			Unused	No results
TRUE			Unused	No results
TRUE	Palasitrin	C27 H30 O1!	Full match	No results
TRUE	Procyanidin B1	C30 H26 O12	Not the top	No results
TRUE	L-Theanine	C7 H14 N2 C	No results	No results
TRUE			Unused	No results
TRUE			Unused	No results
TRUE	N-Acetyl-beta-D-galactosamine	C8 H15 N O6	Full match	No results
TRUE	Phenobarbital-d5	C12 H7 [2]H!	No match	Invalid mass
TRUE			Unused	No results
TRUE	2-Hydroxymethylserine	C4 H9 N O4	Full match	No results
TRUE	Caprolactam	C6 H11 N O	Full match	No results
TRUE	Guanosine	C10 H13 N5	Full match	No results
TRUE	2-oxa-4-azatetracyclo[6.3.1.1~6,10~.0~1,5~]tridecan-	C11 H15 N C	Full match	Full match
TRUE			Unused	No results
TRUE	Myricetin	C15 H10 O8	Full match	No results
TRUE			Unused	No results
TRUE	Thymidine	C10 H14 N2	Full match	No results

TRUE	Camphor	C10 H16 O	No match	No results
TRUE	Dimetridazole	C5 H7 N3 O2	Full match	No results
TRUE			Unused	No results
TRUE	3-(4-methylphenyl)-7,9-diphenylpyrido[3',2':4,5]furo	C28 H19 N3	No match	Invalid mass
TRUE			Unused	No results
TRUE			No results	No results
TRUE	Phenylmethanesulfonylfluoride	C7 H7 F O2 S	No results	No results
TRUE			Unused	No results
TRUE			Unused	No results
TRUE			Unused	No results
TRUE	1-Palmitoylglycerophosphocholine	C24 H50 N C	Full match	No results
TRUE	Sedoheptulose	C7 H14 O7	Full match	No results
TRUE			Unused	No results
TRUE			Unused	No results
TRUE			Unused	No results
TRUE	N-Succinyl-LL-2_6-diaminoheptanedioate	C11 H18 N2	Not the top	No results
TRUE			Unused	No results
TRUE			Unused	No results
TRUE			Unused	No results
TRUE			Unused	No results
TRUE			No results	No results
TRUE	2-Ethoxy-2-oxoethyl ethyl phthalate	C14 H16 O6	Full match	No results
TRUE			Unused	No results
TRUE			No results	No results
TRUE			No results	No results
TRUE	7-(2-hydroxypropan-2-yl)-1,4a-dimethyl-decahydrona	C15 H28 O2	No match	Invalid mass
TRUE			Unused	No results
TRUE	Flamprop-methyl	C17 H15 Cl F	No match	No results
TRUE			No results	No results
TRUE			Unused	No results
TRUE			Unused	No results
TRUE	Oxaloglutarate	C7 H8 O7	Full match	No results
TRUE			Unused	No results
TRUE	2_6-Diaminoheptanedioicacid	C7 H14 N2 C	Full match	No results
TRUE			Unused	No results
TRUE	N-Acetyl-L-2-amino-6-oxopimelate	C9 H13 N O6	Full match	No results
TRUE	2-Methylguanosine	C11 H15 N5	No match	No results
TRUE			Unused	No results
TRUE			Unused	No results
TRUE			Unused	No results
TRUE			No results	No results
TRUE	Benzyl2-methyl-3-oxobutanoate	C12 H14 O3	Full match	No results
TRUE			Unused	No results
TRUE			Unused	No results
TRUE			Unused	No results
TRUE	Kaempferol-3-Galactoside-6''-Rhamnoside-3'''-Rham	C33 H40 O19	Not the top	Full match
TRUE			No results	No results
TRUE	2-(alpha-D-Galactosyl)-sn-glycerol3-phosphate	C9 H19 O11	Not the top	No results
TRUE			Unused	No results
TRUE	Methyl gallate	C8 H8 O5	No results	No results

TRUE	2-(2,4-dihydroxyphenyl)-3,5,7-trihydroxy-4H-chrome	C15 H10 O7	Full match	Full match
TRUE	Cerarvensin	C20 H18 O9	No match	No results
TRUE			Unused	No results
TRUE			Unused	No results
TRUE			Unused	No results
TRUE			Unused	No results
TRUE			Invalid mass	No results
TRUE			No results	No results
TRUE			Unused	No results
TRUE			Unused	No results
TRUE			Unused	No results
TRUE	Hispidulin 7-glucuronide	C22 H20 O12	Not the top	No results
TRUE			Unused	No results
TRUE			Unused	No results
TRUE	Cryptochlorogenic acid	C16 H18 O9	Not the top	No results
TRUE			Unused	No results
TRUE			Unused	No results
TRUE			Unused	No results
TRUE			Unused	No results
TRUE	Ohmefentanyl	C23 H30 N2	Full match	No results
TRUE	Mebutamate	C10 H20 N2	Full match	No results
TRUE	Ethyl 3-oxo-3-(2,4,5-trifluorophenyl)propanoate	C11 H9 F3 O	No match	No results
TRUE			Unused	No results
TRUE	Adenosine2'-phosphate	C10 H14 N5	Not the top	No results
TRUE	1-O-Galloyl-beta-D-glucose	C13 H16 O10	Not the top	No results
TRUE			Unused	No results
TRUE			Unused	No results
TRUE	D-Arginine	C6 H14 N4 C	Full match	No results
TRUE			Unused	No results
TRUE			Unused	No results
TRUE			No results	No results
TRUE	2_7-Anhydro-alpha-N-acetylneuraminicacid	C11 H17 N C	Full match	No results
TRUE			Unused	No results
TRUE			Unused	No results
TRUE			Unused	No results
TRUE			Unused	No results
TRUE			Unused	No results
TRUE			Unused	No results
TRUE	3,5,3'-Trimethoxy-6,7:4',5'-bis (methylenedioxy) flavo	C20 H16 O9	Not the top	No results
TRUE			Unused	No results
TRUE			Unused	No results
TRUE	3'-Deoxyderhamnosylmaysin	C21 H18 O9	Not the top	No results
TRUE	Epiafzelechin 3-O-gallate	C22 H18 O9	Not the top	No results
TRUE	2_7-Anhydro-alpha-N-acetylneuraminicacid	C11 H17 N C	Full match	No results
TRUE			Unused	No results
TRUE			Unused	No results
TRUE			No results	No results
TRUE			Unused	No results
TRUE			Unused	No results
TRUE	Tris(trimethylsiloxy)ethylene	C11 H28 O3	No match	No results

TRUE			Unused	No results
TRUE			Unused	No results
TRUE	1-Methyl-2-azepanone	C7 H13 N O	Full match	No results
TRUE			Unused	No results
TRUE			Unused	No results
TRUE			Unused	No results
TRUE			Unused	No results
TRUE			Unused	No results
TRUE	Abscisic acid	C15 H20 O4	Full match	No results
TRUE	L-alpha-Glutamyl-L-lysine	C11 H21 N3	Full match	No results
TRUE			Unused	No results
TRUE	Proclavaminicacid	C8 H14 N2 C	Full match	No results
TRUE			Unused	No results
TRUE			Unused	No results
TRUE			No results	No results
TRUE	Ellagic acid	C14 H6 O8	Not the top	No results
TRUE			Unused	No results
TRUE			Unused	No results
TRUE			Unused	No results
TRUE			Unused	No results
TRUE	1_8-Dihydroxy-3-methylnaphthalene	C11 H10 O2	Full match	No results
TRUE			Unused	No results
TRUE			No results	No results
TRUE			Unused	No results
TRUE			Unused	No results
TRUE			Unused	No results
TRUE			Unused	No results
TRUE			Unused	No results
TRUE			Unused	No results
TRUE			Unused	No results
TRUE			Unused	No results
TRUE	3,5,3'-Trimethoxy-6,7:4',5'-bis (methylenedioxy) flavo	C20 H16 O9	Not the top	No results
TRUE			Unused	No results
TRUE	Dopamineglucuronide	C14 H19 N C	Not the top	No results
TRUE			No results	No results
TRUE			Unused	No results
TRUE	cis-Zeatinribosidemonophosphate	C15 H22 N5	No match	No results
TRUE	Pantothenicacid(VitaminB5)	C9 H17 N O5	Full match	No results
TRUE			Unused	No results
TRUE	D-Saccharicacid	C6 H10 O8	Full match	No results
TRUE			Unused	No results
TRUE			Unused	No results
TRUE	5,7,3',6'-Tetrahydroxy-6,8,2'-trimethoxyflavone	C18 H16 O9	Not the top	No results
TRUE	(S)-Carnitine	C7 H15 N O3	Full match	No results
TRUE	p-Coumaroylquinicacid	C16 H18 O8	Full match	No results
TRUE			Unused	No results
TRUE			Unused	No results
TRUE	NP-008882	C27 H48 O9	Not the top	Full match
TRUE			Unused	No results
TRUE			Unused	No results

TRUE			Unused	No results
TRUE			Unused	No results
TRUE	3'-UMP	C9 H13 N2 C	Not the top	No results
TRUE			Unused	No results
TRUE	Maritimein	C21 H20 O1:	Not the top	No results
TRUE			Unused	No results
TRUE	3,5,3'-Trimethoxy-6,7:4',5'-bis (methylenedioxy) flavo	C20 H16 O9	Not the top	No results
TRUE			Unused	No results
TRUE			Unused	No results
TRUE	(1E,3E)-4-Hydroxybuta-1,3-diene-1,2,4-tricarboxylate	C7 H6 O7	Invalid mass	No results
TRUE			Unused	No results
TRUE			Unused	No results
TRUE	N1-(2,6-dimethylphenyl)-2-morpholinoacetamide	C14 H20 N2	Full match	Full match
TRUE			Unused	No results
TRUE			Unused	No results
TRUE			No results	No results
TRUE			Unused	No results
TRUE			Unused	No results
TRUE			Unused	No results
TRUE			Unused	No results
TRUE	3'-Deoxyderhamnosylmaysin	C21 H18 O9	Not the top	No results
TRUE	6-O-(beta-D-Xylopyranosyl)-beta-D-glucopyranose	C11 H20 O1(	Not the top	No results
TRUE	benzyl 4-{[(2-morpholinoanilino)carbonyl]amino}tetr	C24 H30 N4	No match	Invalid mass
TRUE	GMP;5'-GMP;Guanosine5'-monophosphate	C10 H14 N5	Full match	No results
TRUE			Unused	No results
TRUE			Unused	No results
TRUE	7-methylthioheptanaldoxime	C8 H17 N O S	No results	No results
TRUE			No results	No results
TRUE	Rishitin	C14 H22 O2	Full match	No results
TRUE			No results	No results
TRUE	Linderoflavone A	C18 H14 O8	Full match	No results
TRUE			Unused	No results
TRUE			Unused	No results
TRUE			Unused	No results
TRUE			Unused	No results
TRUE	Brosimacutin H	C20 H24 O6	Not the top	No results
TRUE			Unused	No results
TRUE	1-O_6-O-Digalloyl-beta-D-glucose	C20 H20 O14	Full match	No results
TRUE			Unused	No results
TRUE			Unused	No results
TRUE	Orientin 7-O-sulfate	C21 H20 O14	No match	No results
TRUE			Unused	No results
TRUE			Unused	No results
TRUE	Indol-3-ylacetyl-myo-inositolL-arabinoside	C21 H27 N C	Full match	No results
TRUE			Unused	No results
TRUE			No results	No results
TRUE	Chalconaringenin 2'-xyloside	C20 H20 O9	Not the top	No results
TRUE			Unused	No results
TRUE			Unused	No results
TRUE			Unused	No results

TRUE			Unused	No results
TRUE			Unused	No results
TRUE			Unused	No results
TRUE	HordatineA	C28 H38 N8	Not the top	No results
TRUE			Unused	No results
TRUE			Unused	No results
TRUE			Unused	No results
TRUE			Unused	No results
TRUE			Unused	No results
TRUE	GMP;5'-GMP;Guanosine5'-monophosphate	C10 H14 N5	Not the top	No results
TRUE			Unused	No results
TRUE	1-alpha-D-Galactosyl-myo-inositol	C12 H22 O1:	Not the top	No results
TRUE			Unused	No results
TRUE			Unused	No results
TRUE			Unused	No results
TRUE			Unused	No results
TRUE			No results	No results
TRUE	6,3'-Dihydroxy-4,4'-dimethoxy-5-methylaurone	C18 H16 O6	Full match	No results
TRUE	9,10-Dihydro-10- (4-hydroxyphenyl) -pyrano [2,3-h]	C24 H20 O8	Not the top	No results
TRUE	Orientin 2''-O-beta-L-arabinofuranoside	C26 H28 O1!	No match	No results
TRUE			Unused	No results
TRUE			Unused	No results
TRUE			Unused	No results
TRUE			Unused	No results
TRUE			No results	No results
TRUE			Unused	No results
TRUE	Protocatechuic acid	C7 H6 O4	No results	No results
TRUE			Unused	No results
TRUE			No results	No results
TRUE	1-O-coumaroyl-β-D-glucose	C15 H18 O8	Not the top	No results
TRUE	5-Methoxy-N_N-dimethyltryptamine	C13 H18 N2	Full match	No results
TRUE			Unused	No results
TRUE	3-Isopropenyl-N-isopropylidenebenzamide	C13 H15 N C	Full match	No results
TRUE			Unused	No results
TRUE			Unused	No results
TRUE			Unused	No results
TRUE			Unused	No results
TRUE			Unused	No results
TRUE	9,10-Dihydro-10- (4-hydroxyphenyl) -pyrano [2,3-h]	C24 H20 O8	Not the top	No results
TRUE			Unused	No results
TRUE			Unused	No results
TRUE			Unused	No results
TRUE	Aureusidin 6-glucuronide	C21 H18 O1:	No match	No results
TRUE			No results	No results
TRUE			Unused	No results
TRUE	Furmecyclox	C14 H21 N C	Full match	No results
TRUE			No results	No results
TRUE			Unused	No results
TRUE			Unused	No results
TRUE			Unused	No results

TRUE	2-Hydroxymethylserine	C4 H9 N O4	Full match	No results
TRUE			No results	No results
TRUE	Hovetrichoside D	C27 H32 O15	Full match	No results
TRUE			No results	No results
TRUE			Unused	No results
TRUE			Unused	No results
TRUE	Coniferylaldehyde	C10 H10 O3	Full match	No results
TRUE			Unused	No results
TRUE	Flunitrazepam	C16 H12 F N	No match	No results
TRUE			Unused	No results
TRUE			Unused	No results
TRUE	5-(4-Acetoxybut-1-ynyl)-2_2'-bithiophene	C14 H12 O2	Not the top	No results
TRUE	1-O-Galloyl-beta-D-glucose	C13 H16 O1(	Not the top	No results
TRUE			Unused	No results
TRUE	6-Hydroxypseudooxynicotine	C10 H14 N2	Full match	No results
TRUE			Unused	No results
TRUE			Unused	No results
TRUE			Unused	No results
TRUE	Ala-Pro(Alanyl-Proline)	C8 H14 N2 C	Full match	No results
TRUE			No results	No results
TRUE			Unused	No results
TRUE			Unused	No results
TRUE			Unused	No results
TRUE	(2R,3S,4S,5R,6R)-2-({[(2R,3R,4R)-3,4-dihydroxy-4-(hy(	C21 H36 O1(	No match	Invalid mass
TRUE			Unused	No results
TRUE	9,10-Dihydro-10- (4-hydroxyphenyl) -pyrano [2,3-h]	C24 H20 O8	Not the top	No results
TRUE	2- (1,3-Benzodioxol-5-yl) -5-hydroxy-6,8-dimethoxy-4	C18 H14 O7	Not the top	No results
TRUE	Caprolactam	C6 H11 N O	Full match	No results
TRUE	Monospermoside	C21 H22 O1(	No match	No results
TRUE			Unused	No results
TRUE	2- (1,3-Benzodioxol-5-yl) -5-hydroxy-6,8-dimethoxy-4	C18 H14 O7	Full match	No results
TRUE			Unused	No results
TRUE			No results	No results
TRUE			Unused	No results
TRUE			Unused	No results
TRUE			Unused	No results
TRUE	Galiposin	C17 H12 O6	Full match	No results
TRUE			Unused	No results
TRUE			Unused	No results
TRUE			Unused	No results
TRUE			Unused	No results
TRUE			Unused	No results
TRUE			Unused	No results
TRUE			Unused	No results
TRUE			Unused	No results
TRUE			No results	No results
TRUE	9,10-Dihydro-10- (3,4-dihydroxyphenyl) -pyrano [2,3	C24 H20 O9	Not the top	No results
TRUE	Norflurazon-desmethyl	C11 H7 Cl F3	No match	No results
TRUE	Sulfurein	C21 H20 O1(	Not the top	No results
TRUE	Sanguinarine	C20 H14 N C	No match	No results

TRUE			Unused	No results
TRUE	Apigenin 7- (6"-methylglucuronide)	C22 H20 O11	Not the top	No results
TRUE			No results	No results
TRUE			Unused	No results
TRUE	Leucinamide	C6 H14 N2 C	Full match	No results
TRUE			Unused	No results
TRUE			Unused	No results
TRUE			Unused	No results
TRUE			Unused	No results
TRUE	5-O-Caffeoylshikimicacid	C16 H16 O8	Not the top	No results
TRUE	2-(Acetamidomethylene)succinate	C7 H9 N O5	Full match	No results
TRUE			No results	No results
TRUE	1-{[5-(2-Hydroxyethoxy)-4-oxopentanoyl]oxy}-2,5-py	C11 H15 N C	Not the top	No results
TRUE	2',4',3,4,alpha-Pentahydroxydihydrochalcone 3'-C-xy	C20 H22 O1(	Full match	No results
TRUE	Bractein	C21 H20 O12	No match	No results
TRUE			Unused	No results
TRUE			Unused	No results
TRUE			Unused	No results
TRUE	8'-Hydroxyabscisate	C15 H20 O5	Full match	No results
TRUE			Unused	No results
TRUE			No results	No results
TRUE	3'-hydroxyacetophenone;3-ACETYLPHENOL	C8 H8 O2	Full match	No results
TRUE			Unused	No results
TRUE			Unused	No results
TRUE			Unused	No results
TRUE			Unused	No results
TRUE			Unused	No results
TRUE			Unused	No results
TRUE	3,5,3'-Trimethoxy-6,7:4',5'-bis (methylenedioxy) flavo	C20 H16 O9	Full match	No results
TRUE			Unused	No results
TRUE			Unused	No results
TRUE			Unused	No results
TRUE			Unused	No results
TRUE			Unused	No results
TRUE	(1R,4S,4aS,8aS)-1-(Hydroxymethyl)-2,5,5,8a-tetrame	C15 H26 O3	Full match	No results
TRUE			Unused	No results
TRUE	2'-Deoxymugineicacid	C12 H20 N2	Full match	No results
TRUE			Unused	No results
TRUE			Unused	No results
TRUE			Unused	No results
TRUE	1-O_2-O_6-O-Trigalloyl-beta-D-glucose	C27 H24 O18	Not the top	No results
TRUE			Unused	No results
TRUE			Unused	No results
TRUE	1H-indol-3-yl(pyridin-2-yl)methanol	C14 H12 N2	No match	Invalid mass
TRUE	Leu-Pro(Leucyl-Proline)	C11 H20 N2	Full match	No results
TRUE			No results	No results
TRUE	9,10-Dihydro-10- (3,4-dihydroxyphenyl) -pyrano [2,3	C24 H20 O9	Not the top	No results
TRUE			Unused	No results
TRUE			Unused	No results
TRUE			Unused	No results

TRUE			Unused	No results
TRUE			Unused	No results
TRUE			Unused	No results
TRUE			Unused	No results
TRUE			Unused	No results
TRUE			Unused	No results
TRUE	Celestolide	C17 H24 O	No match	Invalid mass
TRUE			Unused	No results
TRUE			Unused	No results
TRUE			Unused	No results
TRUE	Diethyl2-methyl-3-oxosuccinate	C9 H14 O5	Full match	No results
TRUE			Unused	No results
TRUE			No results	No results
TRUE			Unused	No results
TRUE			No results	No results
TRUE			Unused	No results
TRUE	2-(Hydroxymethyl)-3-(acetamidomethylene)succinat	C8 H11 N O6	Full match	No results
TRUE			Unused	No results
TRUE			Unused	No results
TRUE			Unused	No results
TRUE	Loganin	C17 H26 O1(	No match	No results
TRUE	(E)-Glutaconate	C5 H6 O4	Full match	No results
TRUE	Phenylmethanesulfonylfluoride	C7 H7 F O2 S	No results	No results
TRUE			Unused	No results
TRUE			No results	No results
TRUE	N-Butyryl-L-homoserinelactone	C8 H13 N O3	Full match	No results
TRUE			Unused	No results
TRUE			Unused	No results
TRUE			Unused	No results
TRUE	Fenobucarb	C12 H17 N C	No results	No results
TRUE			Unused	No results
TRUE	NP-010770	C33 H40 N2	No match	Invalid mass
TRUE			Unused	No results
TRUE			Unused	No results
TRUE			Unused	No results
TRUE	Amoritin	C31 H38 O6	Invalid mass	No results
TRUE			Unused	No results
TRUE	dehypoxanthinefutalosine	C14 H16 O7	Full match	No results
TRUE			Unused	No results
TRUE			Unused	No results
TRUE	Isoorientin 2"-O- (E) -ferulate	C31 H28 O14	No results	No results
TRUE			Unused	No results
TRUE	1_8-Diazacyclotetradecane-2_9-dione	C12 H22 N2	Full match	No results
TRUE	2,6,3',4'-Tetrahydroxy-2-benzylcoumaranone	C15 H12 O6	Full match	No results
TRUE			Unused	No results
TRUE	6-C-Xylosylluteolin	C20 H18 O1(	Not the top	No results
TRUE			Unused	No results
TRUE	2-Hydroxyethylphosphonate	C2 H7 O4 P	Full match	No results
TRUE			No results	No results
TRUE	Thiaminmonophosphate	C12 H18 N4	No match	No results

TRUE			Unused	No results
TRUE	Glutamic Acid	C5 H9 N O4	Full match	No results
TRUE			Unused	No results
TRUE			Unused	No results
TRUE			Unused	No results
TRUE			Unused	No results
TRUE	6-Methoxyluteolin 7-glucuronide	C22 H20 O13	Full match	No results
TRUE			Unused	No results
TRUE			Unused	No results
TRUE			Unused	No results
TRUE			Unused	No results
TRUE			Unused	No results
TRUE			Unused	No results
TRUE	Methyl gallate	C8 H8 O5	No results	No results
TRUE			Unused	No results
TRUE	2-(Acetamidomethylene)succinate	C7 H9 N O5	Full match	No results
TRUE	Maritimetin 6- (6"-p-coumarylglucoside)	C30 H26 O13	Full match	No results
TRUE	AZT	C10 H13 N5	Not the top	No results
TRUE			Unused	No results
TRUE	Carbofuran, 3OH-	C12 H15 N C	Full match	No results
TRUE			No results	No results
TRUE	3',5,5'-Trihydroxy-4',6,7,8-tetramethoxyflavone	C19 H18 O9	Not the top	No results
TRUE	Tuberonicacidglucoside	C18 H28 O9	Full match	No results
TRUE	[FAtrihydroxy(18:0)]9_10_13-trihydroxy-11-octadece	C18 H34 O5	Full match	No results
TRUE	Tranexamic Acid	C8 H15 N O2	Full match	No results
TRUE	(9Z_15Z)-(13S)-12_13-Epoxyoctadeca-9_11_15-trien	C18 H28 O3	Full match	No results
TRUE			Unused	No results
TRUE			Unused	No results
TRUE			No results	No results
TRUE	Flamprop-methyl	C17 H15 Cl F	No match	No results
TRUE			No results	No results
TRUE			Unused	No results
TRUE			Unused	No results
TRUE			Unused	No results
TRUE	Elephantorrhizol	C15 H14 O8	Full match	No results
TRUE			Unused	No results
TRUE	[FAhydroxy(18:0)]12_13-dihydroxy-9Z-octadecenoica	C18 H34 O4	Full match	No results
TRUE	Cerarvensin	C20 H18 O9	Full match	No results
TRUE			Unused	No results
TRUE			Unused	No results
TRUE			Unused	No results
TRUE			Unused	No results
TRUE			No results	No results
TRUE	Isoorientin 6"-O-alpha-L-arabinoside	C25 H26 O15	No match	No results
TRUE			Unused	No results
TRUE			Unused	No results
TRUE	5-O-Caffeoylshikimicacid	C16 H16 O8	Full match	No results
TRUE			Unused	No results
TRUE			Unused	No results
TRUE			Unused	No results

TRUE			Unused	No results
TRUE			Unused	No results
TRUE			Unused	No results
TRUE	Kaempferol 3- (2"-galloyl-alpha-L-arabinopyranoside)	C27 H22 O14	Not the top	No results
TRUE			Unused	No results
TRUE			Unused	No results
TRUE			Unused	No results
TRUE			Unused	No results
TRUE			Unused	No results
TRUE			Unused	No results
TRUE			Unused	No results
TRUE			Unused	No results
TRUE			Unused	No results
TRUE			Unused	No results
TRUE			Unused	No results
TRUE	Okanin 3,4,3',4'-tetramethyl ether	C19 H20 O6	Not the top	No results
TRUE	1-Methyl-2-azepanone	C7 H13 N O	Full match	No results
TRUE			No results	No results
TRUE	Dethiobiotin	C10 H18 N2	Full match	No results
TRUE			Unused	No results
TRUE			No results	No results
TRUE	4α-Carboxy-5α-cholesta-8-en-3β-ol	C28 H46 O3	No results	No results
TRUE	2-Methylguanosine	C11 H15 N5	Not the top	No results
TRUE			Unused	No results
TRUE			Unused	No results
TRUE			Unused	No results
TRUE			No results	No results
TRUE	Maritimetin 6- (6"-acetylglucoside)	C23 H22 O12	Not the top	No results
TRUE			Unused	No results
TRUE			Unused	No results
TRUE	NP-001134	C16 H22 N4	Not the top	Full match
TRUE	Ala-Pro(Alanyl-Proline)	C8 H14 N2 C	Full match	No results
TRUE	1-Aminocyclopropane-1-carboxylate	C4 H7 N O2	Full match	No results
TRUE			Unused	No results
TRUE	2-Amino-3,7-dideoxy-D-threo-hept-6-ulosonate	C7 H13 N O5	Full match	No results
TRUE	Naringenin chalcone	C15 H12 O5	Full match	No results
TRUE	3-Dehydroxycarnitine	C7 H15 N O2	Full match	No results
TRUE			No results	No results
TRUE			Unused	No results
TRUE			Unused	No results
TRUE			Unused	No results
TRUE			Unused	No results
TRUE			Unused	No results
TRUE			Unused	No results
TRUE			Unused	No results
TRUE			Unused	No results
TRUE			Unused	No results
TRUE	Oxalosuccinate	C6 H6 O7	Full match	No results
TRUE			Unused	No results
TRUE	D-2-Amino-hexano-6-lactam	C6 H12 N2 C	Full match	No results

TRUE			Unused	No results
TRUE			Unused	No results
TRUE			No results	No results
TRUE			No results	No results
TRUE	Cytidine;1-beta-delta-Ribofuranosyl-Cytosine	C9 H13 N3 C	Not the top	No results
TRUE			Unused	No results
TRUE			No results	No results
TRUE	Choline	C5 H13 N O	Full match	No results
TRUE			Unused	No results
TRUE	Xanthosine5'-phosphate	C10 H13 N4	Not the top	No results
TRUE			Unused	No results
TRUE	9,10-Dihydro-10- (3,4-dihydroxyphenyl) -pyrano [2,3	C24 H20 O9	Not the top	No results
TRUE			Unused	No results
TRUE			Unused	No results
TRUE	Secologanin	C17 H24 O1(	No match	No results
TRUE			Unused	No results
TRUE	Luteolin 7-sulfate	C15 H10 O9	Full match	No results
TRUE	2_2'-Iminodipropanoate	C6 H11 N O4	Full match	No results
TRUE			Unused	No results
TRUE			Unused	No results
TRUE			Unused	No results
TRUE			Unused	No results
TRUE			Unused	No results
TRUE			Unused	No results
TRUE			Unused	No results
TRUE			Unused	No results
TRUE			Unused	No results
TRUE			Unused	No results
TRUE			No results	No results
TRUE			Unused	No results
TRUE	gamma-Glutamyl-gamma-aminobutyraldehyde	C9 H16 N2 C	Full match	No results
TRUE			Unused	No results
TRUE	Chalconaringenin 2'-xyloside	C20 H20 O9	Not the top	No results
TRUE	1-{[5-(2-Hydroxyethoxy)-4-oxopentanoyl]oxy}-2,5-py	C11 H15 N C	Full match	No results
TRUE			No results	No results
TRUE	Methyl gallate	C8 H8 O5	No results	No results
TRUE			Unused	No results
TRUE	Carbofuran-3-Keto	C12 H13 N C	Full match	No results
TRUE			Unused	No results
TRUE			Unused	No results
TRUE	3-methylthiopropyl-desulfoglucosinolate	C11 H21 N C	No results	No results
TRUE			Unused	No results
TRUE	Chinomethionate	C10 H6 N2 C	Not the top	No results
TRUE			Unused	No results
TRUE			Unused	No results
TRUE			Unused	No results
TRUE			Unused	No results
TRUE			Unused	No results
TRUE	Tributyl citrate acetate	C20 H34 O8	Full match	No results
TRUE			Unused	No results

TRUE			Unused	No results
TRUE			Unused	No results
TRUE			Unused	No results
TRUE	9,10-Dihydro-10- (3,4-dihydroxyphenyl) -pyrano [2,3	C24 H20 O9	Not the top	No results
TRUE			Unused	No results
TRUE	gamma-Glutamyl-gamma-aminobutyraldehyde	C9 H16 N2 C	Full match	No results
TRUE	Agamanone	C18 H16 O8	Not the top	No results
TRUE			Unused	No results
TRUE			Unused	No results
TRUE	5D-5-O-Methyl-2_3_5/4_6-pentahydroxycyclohexano	C7 H12 O6	Full match	No results
TRUE	Isovitexin 2''-O- (6'''- (E) -p-coumaroyl) glucoside 4'-O	C42 H46 O22	Full match	No results
TRUE			Unused	No results
TRUE			Unused	No results
TRUE	2_3'_4_6-Tetrahydroxybenzophenone	C13 H10 O5	Full match	No results
TRUE			No results	No results
TRUE			Unused	No results
TRUE			Invalid mass	No results
TRUE			Unused	No results
TRUE			Unused	No results
TRUE			Unused	No results
TRUE	9,10-Dihydro-10- (4-hydroxyphenyl) -pyrano [2,3-h]	C24 H20 O8	Not the top	No results
TRUE			Unused	No results
TRUE			Unused	No results
TRUE	L-Aspartyl-4-phosphate	C4 H9 N O7 I	Unused	No results
TRUE			Unused	No results
TRUE			Unused	No results
TRUE	Bufexamac	C12 H17 N C	Full match	No results
TRUE			No results	No results
TRUE			Unused	No results
TRUE			Unused	No results
TRUE			Unused	No results
TRUE			Unused	No results
TRUE			Unused	No results
TRUE			Invalid mass	No results
TRUE			Unused	No results
TRUE			Unused	No results
TRUE			Unused	No results
TRUE	9,10-Dihydro-10- (4-hydroxyphenyl) -pyrano [2,3-h]	C24 H20 O8	Not the top	No results
TRUE			Unused	No results
TRUE	Azimsulfuron	C13 H16 N10	No match	No results
TRUE			Unused	No results
TRUE	Chrysin 5-xyloside	C20 H18 O8	Not the top	No results
TRUE	6-Acetyl-D-glucose	C8 H14 O7	Full match	No results
TRUE			Unused	No results
TRUE	Lacosamide	C13 H18 N2	Full match	No results
TRUE			Unused	No results
TRUE	2',4',3,4,alpha-Pentahydroxydihydrochalcone 3'-C-xy	C20 H22 O10	Full match	No results
TRUE			Unused	No results
TRUE			Unused	No results
TRUE	Catechin-4-ol 3-O-beta-D-galactopyranoside	C21 H24 O12	Full match	No results

TRUE			Unused	No results
TRUE			Unused	No results
TRUE	1-Amino-1-deoxy-scyllo-inositol	C6 H13 N O5	Full match	No results
TRUE			Unused	No results
TRUE			No results	No results
TRUE			Unused	No results
TRUE			Unused	No results
TRUE	alpha,4,2'-Trihydroxy-4-O-geranyldihydrochalcone	C24 H28 O5	Full match	No results
TRUE			No results	No results
TRUE			Unused	No results
TRUE			Unused	No results
TRUE	(R)-Prunasin	C14 H17 N C	Full match	No results
TRUE			No results	No results
TRUE	Protocatechuic acid	C7 H6 O4	No results	No results
TRUE			Unused	No results
TRUE			Unused	No results
TRUE			Unused	No results
TRUE			Unused	No results
TRUE			Unused	No results
TRUE			Unused	No results
TRUE			Unused	No results
TRUE			Unused	No results
TRUE			Unused	No results
TRUE			Unused	No results
TRUE			Unused	No results
TRUE	3-Hydroxy-4-((6-hydroxy-m-tolyl)azo)-1-naphthalene	C17 H14 N2	No match	No results
TRUE	Sulfurein	C21 H20 O1(	No match	No results
TRUE	5-Methylcytosine	C5 H7 N3 O	Full match	No results
TRUE	Quercetagetin 4'-methyl ether 7- (6- (E) -caffeylgluco	C31 H28 O1(	Not the top	No results
TRUE	Ecgoninemethylester	C10 H17 N C	Full match	No results
TRUE			Unused	No results
TRUE	Azelaic acid	C9 H16 O4	No results	No results
TRUE			Unused	No results
TRUE			Unused	No results
TRUE			Unused	No results
TRUE			Unused	No results
TRUE			Unused	No results
TRUE			Unused	No results
TRUE			Unused	No results
TRUE			Unused	No results
TRUE			No results	No results
TRUE	Sulfurein	C21 H20 O1(	Not the top	No results
TRUE			Unused	No results
TRUE			Unused	No results
TRUE	Pentadecanoic Acid	C15 H30 O2	No match	Invalid mass
TRUE			Unused	No results
TRUE			Unused	No results
TRUE			Unused	No results
TRUE	albaflavenone	C15 H22 O	Full match	No results
TRUE			Unused	No results

TRUE			Unused	No results
TRUE			Unused	No results
TRUE			Unused	No results
TRUE			No results	No results
TRUE	Aminopropylcadaverine	C8 H21 N3	Full match	No results
TRUE			Unused	No results
TRUE			No results	No results
TRUE			Unused	No results
TRUE	Octamethylcyclotetrasiloxane	C8 H24 O4 S	No match	No results
TRUE			Unused	No results
TRUE	FL64DBGM0001_a	C19 H22 O5	Full match	No results
TRUE			Unused	No results
TRUE			No results	No results
TRUE	NP-008993	C18 H34 O4	No match	Invalid mass
TRUE	2-Propenoic acid, 2-phenoxyethyl ester	C11 H12 O3	Full match	No results
TRUE	N-Succinyl-LL-2_6-diaminoheptanedioate	C11 H18 N2	Full match	No results
TRUE			Unused	No results
TRUE	Monospermoside	C21 H22 O1(	No match	No results
TRUE			Unused	No results
TRUE			Unused	No results
TRUE			Unused	No results
TRUE			No results	No results
TRUE			No results	No results
TRUE			Unused	No results
TRUE			Unused	No results
TRUE	gamma-Glutamyl-gamma-aminobutyraldehyde	C9 H16 N2 C	Full match	No results
TRUE			Unused	No results
TRUE			Unused	No results
TRUE			Unused	No results
TRUE			Unused	No results
TRUE			Unused	No results
TRUE			Unused	No results
TRUE			Unused	No results
TRUE			Unused	No results
TRUE	Caprolactam	C6 H11 N O	Full match	No results
TRUE	6-Phospho-beta-D-glucosyl-(1_4)-D-glucose	C12 H23 O14	Not the top	No results
TRUE			Unused	No results
TRUE	FL63ACGCN001_a	C19 H19 N C	Full match	No results
TRUE			No results	No results
TRUE			Unused	No results
TRUE	2_5-Dihydroxybenzaldehyde	C7 H6 O3	Full match	No results
TRUE			Unused	No results
TRUE			Unused	No results
TRUE			Unused	No results
TRUE			Unused	No results
TRUE			Unused	No results
TRUE			Unused	No results
TRUE	3-hydroxy-1H-quinolin-4-one	C9 H7 N O2	Full match	No results
TRUE			Unused	No results
TRUE	Phytosphingosine;D-Ribo-2-amino-1_3_4-Octadecan	C18 H39 N C	Full match	No results

TRUE			Unused	No results
TRUE			No results	No results
TRUE	Dihydrophaseicacid	C15 H22 O5	Full match	No results
TRUE	2_3_4_5-Tetrahydrodipicolinate	C7 H9 N O4	Full match	No results
TRUE			Unused	No results
TRUE			Unused	No results
TRUE			Unused	No results
TRUE	2-Furoylglycine;Pyromucuricacid	C7 H7 N O4	Full match	No results
TRUE			Unused	No results
TRUE			Unused	No results
TRUE			Unused	No results
TRUE			Unused	No results
TRUE			Unused	No results
TRUE			Unused	No results
TRUE			Unused	No results
TRUE			Unused	No results
TRUE			Unused	No results
TRUE			Unused	No results
TRUE	6-(2-hydroxy-3-methyl-3-{[(2S,3R,4S,5S,6R)-3,4,5-trih	C22 H30 O1	No match	Invalid mass
TRUE			Unused	No results
TRUE			Unused	No results
TRUE			Unused	No results
TRUE	Alternariol	C14 H10 O5	Full match	No results
TRUE			Unused	No results
TRUE			Unused	No results
TRUE	N-D-Glucosylarylamine	C12 H17 N C	Full match	No results
TRUE			Unused	No results
TRUE			Unused	No results
TRUE			Unused	No results
TRUE			Unused	No results
TRUE			No results	No results
TRUE			Unused	No results
TRUE			Unused	No results
TRUE			Unused	No results
TRUE	N-Acetyl-L-glutamate5-semialdehyde	C7 H11 N O4	Full match	No results
TRUE			Unused	No results
TRUE	Verrucarol	C15 H22 O4	Full match	No results
TRUE			Unused	No results
TRUE			Unused	No results
TRUE			Unused	No results
TRUE			Unused	No results
TRUE			Unused	No results
TRUE			No results	No results
TRUE	3,5-Diacetyltambulin	C22 H20 O9	No match	No results
TRUE	Leucinamide	C6 H14 N2 C	Full match	No results
TRUE			No results	No results
TRUE			Unused	No results
TRUE			Unused	No results
TRUE	Brosimacutin C	C20 H22 O5	Not the top	No results
TRUE			Unused	No results

TRUE	5,7,2'-Trihydroxyflavone 7-glucuronide	C21 H18 O11	Full match	No results
TRUE			Unused	No results
TRUE			No results	No results
TRUE	Poriolide	C29 H26 O12	No match	No results
TRUE			Unused	No results
TRUE			No results	No results
TRUE	FL2FEGGS0001_a	C20 H22 O8	Full match	No results
TRUE			Unused	No results
TRUE			Unused	No results
TRUE			No results	No results
TRUE	(±)9-HpODE	C18 H32 O4	No match	Invalid mass
TRUE	ArachidoylEthanolamide	C22 H45 N C	Full match	No results
TRUE	Miglitol	C8 H17 N O5	Full match	No results
TRUE	9,10-Dihydro-10- (3,4-dihydroxyphenyl) -pyrano [2,3	C24 H20 O9	Not the top	No results
TRUE			Unused	No results
TRUE			Unused	No results
TRUE			Unused	No results
TRUE	Xanthine	C5 H4 N4 O2	Full match	No results
TRUE	N-Butyryl-L-homoserinelactone	C8 H13 N O3	Full match	No results
TRUE			Unused	No results
TRUE	4-[(3-Acetamido-4-methylphenyl)amino]-1-amino-9,1	C23 H19 N3	Not the top	No results
TRUE	3-Deoxy-D-manno-octulosonate	C8 H14 O8	Full match	No results
TRUE			Unused	No results
TRUE	Desmosdumotin C	C19 H20 O4	Not the top	No results
TRUE			Unused	No results
TRUE			Unused	No results
TRUE			Unused	No results
TRUE			Unused	No results
TRUE			No results	No results
TRUE	Chicoricacid	C22 H18 O12	Not the top	No results
TRUE			Unused	No results
TRUE			Unused	No results
TRUE			Unused	No results
TRUE			Unused	No results
TRUE			Unused	No results
TRUE			Unused	No results
TRUE			Unused	No results
TRUE			Unused	No results
TRUE			Unused	No results
TRUE			Unused	No results
TRUE			Unused	No results
TRUE	Atenolol	C14 H22 N2	Full match	No results
TRUE			Unused	No results
TRUE			Unused	No results
TRUE			Unused	No results
TRUE			Unused	No results
TRUE			Unused	No results
TRUE			Unused	No results
TRUE			Unused	No results
TRUE			Unused	No results

TRUE			Unused	No results
TRUE			Unused	No results
TRUE	Methylone	C11 H13 N C	Full match	No results
TRUE			Unused	No results
TRUE			Unused	No results
TRUE			Unused	No results
TRUE			Unused	No results
TRUE			Unused	No results
TRUE	D-2-Amino-hexano-6-lactam	C6 H12 N2 C	Full match	No results
TRUE	2-Hydroxy-3-carboxy-6-oxo-7-methylocta-2,4-dienoa	C10 H12 O6	Full match	No results
TRUE			No results	No results
TRUE			Unused	No results
TRUE			Unused	No results
TRUE			Unused	No results
TRUE	Aesculin(Esculin)	C15 H16 O9	Not the top	No results
TRUE	6-Phospho-beta-D-glucosyl-(1_4)-D-glucose	C12 H23 O14	No match	No results
TRUE	Pirimicarb	C11 H18 N4	Not the top	No results
TRUE			No results	No results
TRUE			No results	No results
TRUE			Unused	No results
TRUE			Unused	No results
TRUE	Chicoricacid	C22 H18 O12	Not the top	No results
TRUE			Unused	No results
TRUE	p-cresolsulfatepotassium;p-Cresolsulfate	C7 H8 O4 S	Full match	No results
TRUE			Unused	No results
TRUE			Unused	No results
TRUE			Unused	No results
TRUE			Unused	No results
TRUE			Unused	No results
TRUE	Ligustilide	C12 H14 O2	No match	No results
TRUE			No results	No results
TRUE			Unused	No results
TRUE			Unused	No results
TRUE			Unused	No results
TRUE			Unused	No results
TRUE			Unused	No results
TRUE	6-Hydroxyluteolin 7-sulfate	C15 H10 O1(	Not the top	No results
TRUE			Unused	No results
TRUE			Unused	No results
TRUE	p-Coumaroylquinicacid	C16 H18 O8	No match	No results
TRUE	Pseudosindorin	C15 H12 O5	Full match	No results
TRUE	Elephantorrhizol	C15 H14 O8	Full match	No results
TRUE			No results	No results
TRUE			Unused	No results
TRUE			Unused	No results
TRUE			Unused	No results
TRUE			No results	No results
TRUE	Caprolactam	C6 H11 N O	Full match	No results
TRUE			Unused	No results
TRUE			Unused	No results

TRUE			Unused	No results
TRUE			Unused	No results
TRUE			Unused	No results
TRUE			Unused	No results
TRUE			Unused	No results
TRUE			Unused	No results
TRUE	Allyl 5-methyl-2-oxo-1,3-dioxane-5-carboxylate	C9 H12 O5	Not the top	No results
TRUE			Unused	No results
TRUE			Unused	No results
TRUE			No results	No results
TRUE			Unused	No results
TRUE	Quinoxyfen	C15 H8 Cl2 F	No match	No results
TRUE			Unused	No results
TRUE	Thiaminmonophosphate	C12 H18 N4	No match	No results
TRUE			No results	No results
TRUE			Unused	No results
TRUE			No results	No results
TRUE			Invalid mass	No results
TRUE			Unused	No results
TRUE			Unused	No results
TRUE			Unused	No results
TRUE	Dethiobiotin	C10 H18 N2	Full match	No results
TRUE			Unused	No results
TRUE			Unused	No results
TRUE			Unused	No results
TRUE			Unused	No results
TRUE			Unused	No results
TRUE	Mesosulfuron-methyl	C17 H21 N5	No match	No results
TRUE	Epigallocatechin 3-O-caffeate	C24 H20 O1(	Not the top	No results
TRUE	all-trans-Heptaprenyldiphosphate	C35 H60 O7	Full match	No results
TRUE			Unused	No results
TRUE			Unused	No results
TRUE	3-Vinyl-2-pyrrolidinone	C6 H9 N O	Full match	No results
TRUE	1-Methylguanine	C6 H7 N5 O	Full match	No results
TRUE			Unused	No results
TRUE	Benzoyl-β-D-glucopyranose	C13 H16 O7	Not the top	No results
TRUE	3,4-DMMC	C12 H17 N C	Full match	No results
TRUE	Aspalathin	C21 H24 O1:	Unused	No results
TRUE			Unused	No results
TRUE			Unused	No results
TRUE	1-Methyladenine	C6 H7 N5	Full match	No results
TRUE			Unused	No results
TRUE			Unused	No results
TRUE			Unused	No results
TRUE			Unused	No results
TRUE			Unused	No results
TRUE			Unused	No results
TRUE			Unused	No results
TRUE			Unused	No results
TRUE			Unused	No results

TRUE	3'-Deoxyderhamnosylmaysin	C21 H18 O9	Not the top	No results
TRUE			Unused	No results
TRUE			No results	No results
TRUE	Isoswertisin 2"-O- (2"'-methylbutyrate)	C27 H30 O1:	Not the top	No results
TRUE			Unused	No results
TRUE			Unused	No results
TRUE			Unused	No results
TRUE			Invalid mass	No results
TRUE			Unused	No results
TRUE	Butylone	C12 H15 N C	Full match	No results
TRUE			Unused	No results
TRUE			Unused	No results
TRUE			Unused	No results
TRUE			Unused	No results
TRUE	dehypoxanthinefutalosine	C14 H16 O7	Not the top	No results
TRUE			Unused	No results
TRUE	(-)-MenthylO-beta-D-glucoside	C16 H30 O6	Not the top	No results
TRUE			Unused	No results
TRUE	5,3'-Dihydroxy-6,7,4',5'-tetramethoxyflavanone	C19 H20 O8	Not the top	No results
TRUE	Epigallocatechin 3-O-caffeate	C24 H20 O1(	No match	No results
TRUE			Unused	No results
TRUE			Unused	No results
TRUE			Unused	No results
TRUE			Unused	No results
TRUE			Unused	No results
TRUE	5-Amino-6-(5'-phosphoribitylamino)uracil	C9 H17 N4 C	No match	No results
TRUE			Unused	No results
TRUE			No results	No results
TRUE	5-Methylcytosine	C5 H7 N3 O	Full match	No results
TRUE			Unused	No results
TRUE	Lenacil	C13 H18 N2	Full match	No results
TRUE			Unused	No results
TRUE			Unused	No results
TRUE			Unused	No results
TRUE			Unused	No results
TRUE			Unused	No results
TRUE			No results	No results
TRUE	3-amino-1H-pyrazolo[4,3-c]pyridine-4,6-diol	C6 H6 N4 O2	No match	Invalid mass
TRUE			Unused	No results
TRUE	Aesculin(Esculin)	C15 H16 O9	Not the top	No results
TRUE			Unused	No results
TRUE	Flufenacet	C14 H13 F4 I	No match	No results
TRUE			Unused	No results
TRUE			Unused	No results
TRUE	Citrinin	C13 H14 O5	Full match	No results
TRUE	1-palmitoylglycerol;MAG(16:0)	C19 H38 O4	Not the top	No results
TRUE	3-[(Carboxycarbonyl)amino]-L-alanine	C5 H8 N2 O5	No match	No results
TRUE			Unused	No results
TRUE			Unused	No results
TRUE	NP-012534	C15 H24 O5	No match	Invalid mass

TRUE	3,4,2',3',4',6',alpha-Heptahydroxychalcone 2'-glucosi	C21 H22 O1:	Not the top	No results
TRUE			Unused	No results
TRUE			Unused	No results
TRUE	3,5,7-Tris (acetyloxy) -2- [4- (acetyloxy) -3-hydroxypl	C23 H18 O1:	No match	No results
TRUE			No results	No results
TRUE			Unused	No results
TRUE	3-Vinyltoluene	C9 H10	Full match	No results
TRUE	Bufencarb	C13 H19 N C	Full match	No results
TRUE			No results	No results
TRUE			Unused	No results
TRUE	Cerarvensin	C20 H18 O9	Not the top	No results
TRUE	Hispidol 6-glucoside	C21 H20 O9	No match	No results
TRUE	Apigenin 7- (6"-crotonylglucoside)	C25 H24 O1:	Not the top	No results
TRUE			Unused	No results
TRUE			Unused	No results
TRUE	Chrysin 7-glucuronide	C21 H18 O1(	Not the top	No results
TRUE			Unused	No results
TRUE	3,5,7,3',4',5'-Hexahydroxy-6,8-dimethylflavanone	C17 H16 O8	Full match	No results
TRUE	Methohexital	C14 H18 N2	Full match	No results
TRUE			Unused	No results
TRUE			Unused	No results
TRUE			Unused	No results
TRUE			Unused	No results
TRUE			Unused	No results
TRUE			Unused	No results
TRUE			Unused	No results
TRUE	4-Hydroxybenzoic acid	C7 H6 O3	No results	No results
TRUE			Unused	No results
TRUE			No results	No results
TRUE			Unused	No results
TRUE			Invalid mass	No results
TRUE			Unused	No results
TRUE			Unused	No results
TRUE			Unused	No results
TRUE	NP-005406	C32 H41 N3	No match	Invalid mass
TRUE	NP-012534	C15 H24 O5	No match	Invalid mass
TRUE	[FA(18:1)]9Z-Octadecen-12-ynoicacid	C18 H30 O2	Full match	No results
TRUE			Unused	No results
TRUE			Unused	No results
TRUE	Inosine5'-monophosphate;Inosinicacid	C10 H11 N4	Not the top	No results
TRUE			No results	No results
TRUE			Unused	No results
TRUE	Apigenin 7- (6"-methylglucuronide)	C22 H20 O1:	Not the top	No results
TRUE			Unused	No results
TRUE	2,2-Bis(hydroxymethyl)-1,3-propanediyl bis{3-[4-hydi	C39 H60 O8	Full match	No results
TRUE			Unused	No results
TRUE	Hamilcone	C18 H18 O7	Full match	No results
TRUE			Unused	No results
TRUE	Biotin	C10 H16 N2	Full match	No results
TRUE	2-(2,4-dihydroxyphenyl)-3,5,7-trihydroxy-4H-chrome	C15 H10 O7	Full match	Full match

TRUE			Unused	No results
TRUE	(2S) -5,7,3',4'-Tetrahydroxyflavanone 7- (6-galloylgluc	C28 H26 O15	No match	No results
TRUE			Unused	No results
TRUE			Unused	No results
TRUE			Unused	No results
TRUE			Unused	No results
TRUE			Unused	No results
TRUE	Cryptochlorogenic acid	C16 H18 O9	Not the top	No results
TRUE			Unused	No results
TRUE			Unused	No results
TRUE			Unused	No results
TRUE	Cymoxanil	C7 H10 N4 C	Full match	No results
TRUE	5-Amino-6-(5'-phosphoribitylamino)uracil	C9 H17 N4 C	Not the top	No results
TRUE			Unused	No results
TRUE			Unused	No results
TRUE			Unused	No results
TRUE			No results	No results
TRUE			Unused	No results
TRUE			Unused	No results
TRUE			No results	No results
TRUE	Maritimetin 6- (6"-p-coumarylglucoside)	C30 H26 O13	Full match	No results
TRUE			Unused	No results
TRUE			Unused	No results
TRUE			Unused	No results
TRUE			Unused	No results
TRUE			Unused	No results
TRUE	N-Palmitoyl-D-erythro-sphingosine	C34 H67 N C	Full match	No results
TRUE			Unused	No results
TRUE			Unused	No results
TRUE			Unused	No results
TRUE			Unused	No results
TRUE	Isorhamnetin 3-O-beta-D-2",3",4"-triacetylglucopyra	C28 H28 O15	Not the top	No results
TRUE			Unused	No results
TRUE			Unused	No results
TRUE	Taxifolin 3'- (6"-phenylacetylglucoside)	C29 H28 O13	Not the top	No results
TRUE			Unused	No results
TRUE			Unused	No results
TRUE	1-deoxynojirimycin	C6 H13 N O4	Full match	No results
TRUE			Unused	No results
TRUE			Unused	No results
TRUE	Raucaffricine	C27 H32 N2	No match	No results
TRUE	L-Indospicine	C7 H15 N3 C	Full match	No results
TRUE			Unused	No results
TRUE	Saccharopine	C11 H20 N2	Not the top	No results
TRUE	3,6-Dimethoxy-6",6"-dimethyl-3',4'-methylenedioxyp	C23 H20 O7	Not the top	No results
TRUE			Unused	No results
TRUE			Unused	No results
TRUE			Unused	No results
TRUE			Unused	No results
TRUE	Poriolide	C29 H26 O12	Not the top	No results

TRUE	2-Hydroxy-6-oxonona-2_4-diene-1_9-dioate	C9 H10 O6	Not the top	No results
TRUE	N,N-Dimethyloctadecanamide	C20 H41 N C	Full match	No results
TRUE			Unused	No results
TRUE	2-Succinylbenzoate	C11 H10 O5	Full match	No results
TRUE	Meprobamate	C9 H18 N2 C	Full match	No results
TRUE			Unused	No results
TRUE			Unused	No results
TRUE			Unused	No results
TRUE			Unused	No results
TRUE			Unused	No results
TRUE			Unused	No results
TRUE			Unused	No results
TRUE	3,5,3'-Trimethoxy-6,7:4',5'-bis (methylenedioxy) flavo	C20 H16 O9	Not the top	No results
TRUE	(R)-Prunasin	C14 H17 N C	Not the top	No results
TRUE	Dehydrohistidyltryptophanyldiketopiperazine	C17 H15 N5	Not the top	No results
TRUE	Tricetin 7,3',4',5'-trimethyl eter 5-xylosyl- (1->2) -rhar	C30 H36 O15	No match	No results
TRUE			Unused	No results
TRUE			Unused	No results
TRUE	Decylubiquinol	C19 H32 O4	Full match	No results
TRUE			Unused	No results
TRUE			Unused	No results
TRUE			Unused	No results
TRUE			Unused	No results
TRUE	Valethamate	C19 H31 N C	Full match	No results
TRUE			Unused	No results
TRUE			Unused	No results
TRUE	Carbinoxamine	C16 H19 Cl N	No match	No results
TRUE			Unused	No results
TRUE			No results	No results
TRUE	4-(Diethylamino)benzaldehyde	C11 H15 N C	Full match	Full match
TRUE			No results	No results
TRUE			Unused	No results
TRUE			No results	No results
TRUE	Aromadendrin 3-beta-L-arabinopyranoside	C20 H20 O10	Not the top	No results
TRUE	Tixocortol Pivalate	C26 H38 O5	No match	No results
TRUE			Unused	No results
TRUE	NP-005406	C32 H41 N3	No match	Invalid mass
TRUE	6-Hydroxyluteolin 7-sulfate	C15 H10 O10	Not the top	No results
TRUE	3'-UMP	C9 H13 N2 C	Not the top	No results
TRUE			Unused	No results
TRUE	1-Deoxy-D-altro-heptulose7-phosphate	C7 H15 O9 P	Unused	No results
TRUE			Unused	No results
TRUE			Unused	No results
TRUE	Phenethylamineglucuronide	C14 H19 N C	Not the top	No results
TRUE			No results	No results
TRUE	Dihydrothymine	C5 H8 N2 O2	Full match	No results
TRUE			Unused	No results
TRUE			Unused	No results
TRUE	7-Hydroxy-6-methyl-8-ribityllumazine	C12 H16 N4	Full match	No results
TRUE	(2S,3S,4S,5R,6R)-6-{[(3S,6aR,6bS,8aS,14bR)-4,4,6a,6b	C54 H86 O24	No match	Invalid mass

TRUE			Unused	No results
TRUE			Unused	No results
TRUE			Unused	No results
TRUE	6-Gingerol	C17 H26 O4	Not the top	No results
TRUE			Unused	No results
TRUE	Chicoricacid	C22 H18 O12	Full match	No results
TRUE			No results	No results
TRUE	Maritimetin 6- (6"-acetylglucoside)	C23 H22 O12	Not the top	No results
TRUE			Unused	No results
TRUE			Unused	No results
TRUE	3_4-Dihydroxyphthalate	C8 H6 O6	Not the top	No results
TRUE			Unused	No results
TRUE			Unused	No results
TRUE			Unused	No results
TRUE			Unused	No results
TRUE			Unused	No results
TRUE			Unused	No results
TRUE			Unused	No results
TRUE			Unused	No results
TRUE			Unused	No results
TRUE			Unused	No results
TRUE			Unused	No results
TRUE	Cerarvensin	C20 H18 O9	Not the top	No results
TRUE	Trinexapac-ethyl	C13 H16 O5	Full match	No results
TRUE			Invalid mass	No results
TRUE			Unused	No results
TRUE			No results	No results
TRUE	N-Succinyl-LL-2_6-diaminoheptanedioate	C11 H18 N2	Not the top	No results
TRUE			Unused	No results
TRUE			Unused	No results
TRUE			Unused	No results
TRUE	beta-Ionone	C13 H20 O	Full match	No results
TRUE			Unused	No results
TRUE	Pentadecanoic Acid	C15 H30 O2	No match	Invalid mass
TRUE	2_2'-Iminodipropanoate	C6 H11 N O4	Full match	No results
TRUE			Unused	No results
TRUE			Unused	No results
TRUE			Unused	No results
TRUE			Unused	No results
TRUE			No results	No results
TRUE			Unused	No results
TRUE			Unused	No results
TRUE			Unused	No results
TRUE			Unused	No results
TRUE	?-(Methylenecyclopropyl)glycine	C6 H9 N O2	Full match	No results
TRUE			Unused	No results
TRUE			Unused	No results
TRUE			Unused	No results
TRUE			No results	No results
TRUE			No results	No results

TRUE			Unused	No results
TRUE	(S)-4-Amino-5-oxopentanoate	C5 H9 N O3	Full match	No results
TRUE			Unused	No results
TRUE			Unused	No results
TRUE			Unused	No results
TRUE			Unused	No results
TRUE			Unused	No results
TRUE			Unused	No results
TRUE	(S)-Methylmalonatesemialdehyde	C4 H6 O3	Full match	No results
TRUE	Norflurazon	C12 H9 Cl F3	No match	No results
TRUE	Isocarbamid	C8 H15 N3 C	No results	No results
TRUE			No results	No results
TRUE			Unused	No results
TRUE			Unused	No results
TRUE	Dethiobiotin	C10 H18 N2	Full match	No results
TRUE			Unused	No results
TRUE			Unused	No results
TRUE			Unused	No results
TRUE			Unused	No results
TRUE	2,2'-[(4-Methylphenyl)imino]diethanol	C11 H17 N C	Full match	No results
TRUE			Unused	No results
TRUE			Unused	No results
TRUE			Unused	No results
TRUE			Unused	No results
TRUE			Unused	No results
TRUE			Unused	No results
TRUE			Unused	No results
TRUE	Quercetin 3- (2",3",4"-triacetylgalactoside)	C27 H26 O15	No match	No results
TRUE			Unused	No results
TRUE			Unused	No results
TRUE			Unused	No results
TRUE			Unused	No results
TRUE			Unused	No results
TRUE			Unused	No results
TRUE			Unused	No results
TRUE	Gossypetin 7-methyl ether 8-acetate	C18 H14 O9	Not the top	No results
TRUE			Unused	No results
TRUE	Okanin 4'-O- (4",6"-di-O-acetylglucoside)	C25 H26 O13	Full match	No results
TRUE	2-Hydroxy-6-oxonona-2_4-diene-1_9-dioate	C9 H10 O6	Full match	No results
TRUE			Unused	No results
TRUE			Unused	No results
TRUE			Unused	No results
TRUE			No results	No results
TRUE	AminoDHQ	C7 H11 N O5	Full match	No results
TRUE			Unused	No results
TRUE			No results	No results
TRUE			Unused	No results
TRUE			Unused	No results
TRUE	1'-Acetoxyeugenolacetate	C14 H16 O5	Not the top	No results
TRUE	4-Guanidinobutanal	C5 H11 N3 C	Full match	No results

TRUE			Unused	No results
TRUE			Unused	No results
TRUE			Unused	No results
TRUE			Unused	No results
TRUE	Irebsartan	C25 H28 N6	No match	No results
TRUE			Unused	No results
TRUE			No results	No results
TRUE	4-(beta-D-Glucosyloxy)benzoate	C13 H16 O8	Not the top	No results
TRUE			Unused	No results
TRUE			Unused	No results
TRUE			Unused	No results
TRUE			Unused	No results
TRUE			Unused	No results
TRUE	3'-CMP	C9 H14 N3 C	Full match	No results
TRUE			Unused	No results
TRUE			Unused	No results
TRUE			Unused	No results
TRUE			Unused	No results
TRUE	Pantothenicacid(VitaminB5)	C9 H17 N O5	Full match	No results
TRUE	[FA(18:3)]13S-hydroperoxy-9Z_11E_14Z-octadecatrie	C18 H30 O4	Not the top	No results
TRUE			Unused	No results
TRUE	Phenylglyoxylate	C8 H6 O3	Full match	No results
TRUE	3,4',6'-Trihydroxy-4,2'-dimethoxychalcone 4'-O-rutin	C29 H36 O15	Full match	No results
TRUE			Unused	No results
TRUE			Unused	No results
TRUE	L-isoglutamine	C6 H12 N2 C	Full match	No results
TRUE	3'-C-Glucosylisoliquiritigenin	C21 H22 O9	No results	No results
TRUE	Pongamoside A	C23 H20 O9	No match	No results
TRUE			Unused	No results
TRUE	Kaempferol 3-glucosyl- (1->3) (4'''-acetylrhamnosyl) (	C35 H42 O21	Not the top	No results
TRUE			Unused	No results
TRUE			Unused	No results
TRUE			Unused	No results
TRUE			Unused	No results
TRUE			No results	No results
TRUE			Unused	No results
TRUE			Unused	No results
TRUE			No results	No results
TRUE			No results	No results
TRUE	IBMX	C10 H14 N4	No match	No results
TRUE			Unused	No results
TRUE	UNII:OL56191M6N	C12 H18 O4	Full match	No results
TRUE	Tephrodin	C25 H22 O8	Not the top	No results
TRUE			Unused	No results
TRUE	3,4',6'-Trihydroxy-4,2'-dimethoxychalcone 4'-O-rutin	C29 H36 O15	Full match	No results
TRUE			Invalid mass	No results
TRUE			Unused	No results
TRUE			Unused	No results
TRUE			Unused	No results
TRUE			No results	No results

TRUE			No results	No results
TRUE	Hypoglycin A	C7 H11 N O2	Full match	No results
TRUE	FL64DBGM0001_a	C19 H22 O5	Full match	No results
TRUE			Unused	No results
TRUE			Unused	No results
TRUE			Unused	No results
TRUE			Unused	No results
TRUE	2',4',3,4,alpha-Pentahydroxydihydrochalcone 3'-C-xy	C20 H22 O1(	Full match	No results
TRUE	6a,12b-Dihydro-3,10,11,12-tetrahydroxy-6- (3,4,5-tri	C22 H16 O1(	Full match	No results
TRUE			Unused	No results
TRUE	Fenpropimorph	C20 H33 N C	Full match	No results
TRUE	Maritimetin 6- (6"-p-coumarylglucoside)	C30 H26 O1:	Not the top	No results
TRUE			Unused	No results
TRUE			Unused	No results
TRUE			Unused	No results
TRUE			Unused	No results
TRUE			Unused	No results
TRUE	Ecgonine	C9 H15 N O3	No results	No results
TRUE	Monospermoside	C21 H22 O1(	Not the top	No results
TRUE	3,4-Methylenedioxy-2',4'-dimethoxychalcone	C18 H16 O5	Not the top	No results
TRUE			Unused	No results
TRUE			Unused	No results
TRUE			Unused	No results
TRUE	Vitexin 2"-O- (E) -ferulate	C31 H28 O1:	No match	No results
TRUE			Unused	No results
TRUE			Unused	No results
TRUE	benzocatechol	C10 H8 O2	Full match	No results
TRUE			Unused	No results
TRUE			Unused	No results
TRUE	5,7,3'-Trihydroxy-6,4',5'-trimethoxyflavanone	C18 H18 O8	Full match	No results
TRUE			Unused	No results
TRUE			Unused	No results
TRUE			Unused	No results
TRUE			Unused	No results
TRUE			Unused	No results
TRUE			Unused	No results
TRUE			Unused	No results
TRUE			No results	No results
TRUE			Unused	No results
TRUE			No results	No results
TRUE			Unused	No results
TRUE			Unused	No results
TRUE			Unused	No results
TRUE	Eplerenone	C24 H30 O6	Not the top	No results
TRUE	Amfepramone	C13 H19 N C	Full match	No results
TRUE			Unused	No results
TRUE			Unused	No results
TRUE	2'-Hydroxyaurone 2'-gentiobioside	C27 H30 O1:	Not the top	No results
TRUE	AicarMonophosphate;5-Aminoimidazole-4-carboxam	C9 H15 N4 C	Not the top	No results
TRUE			No results	No results

TRUE			Unused	No results
TRUE			Unused	No results
TRUE			Unused	No results
TRUE			Unused	No results
TRUE			Unused	No results
TRUE			Unused	No results
TRUE	1-(5'-Phosphoribosyl)-5-formamido-4-imidazolecarbo	C10 H15 N4	No match	No results
TRUE			Unused	No results
TRUE			No results	No results
TRUE	Thymidine5'-monophosphate;dTMP	C10 H15 N2	No match	No results
TRUE	S-(-)-Dropropizine	C13 H20 N2	Full match	No results
TRUE	Alminoprofen	C13 H17 N C	Full match	No results
TRUE			Unused	No results
TRUE			Unused	No results
TRUE			Unused	No results
TRUE			No results	No results
TRUE			Unused	No results
TRUE			Unused	No results
TRUE			No results	No results
TRUE			No results	No results
TRUE			Unused	No results
TRUE			Unused	No results
TRUE	FL63ACGCN001_a	C19 H19 N C	Not the top	No results
TRUE	Pantothenicacid(VitaminB5)	C9 H17 N O5	No match	No results
TRUE			Unused	No results
TRUE			Unused	No results
TRUE			Unused	No results
TRUE	Bendiocarb	C11 H13 N C	Full match	No results
TRUE			Unused	No results
TRUE			Unused	No results
TRUE	Sulfosalicylic Acid	C7 H6 O6 S	Full match	No results
TRUE	Remerin	C17 H14 O7	No match	No results
TRUE			Unused	No results
TRUE			No results	No results
TRUE			Unused	No results
TRUE	2-coumarate	C9 H8 O3	Full match	No results
TRUE	5,4'-Dihidroxy-6,7,3',5'-tetramethoxyflavone	C19 H18 O8	Full match	No results
TRUE	Isocordoin	C20 H20 O3	Not the top	No results
TRUE			Unused	No results
TRUE	N1-[4-(cyanomethyl)phenyl]-4-chlorobenzamide	C15 H11 Cl N	No match	Invalid mass
TRUE	2-(Hydroxymethyl)-3-(acetamidomethylene)succinat	C8 H11 N O6	Full match	No results
TRUE	exo-1,7,7-Trimethylbicyclo(2.2.1)hept-2-yl acrylate	C13 H20 O2	Full match	No results
TRUE	3-Hydroxykynurenine	C10 H12 N2	Full match	No results
TRUE	Norcimifugin	C15 H16 O6	Unused	No results
TRUE			Unused	No results
TRUE	Porphobilinogen	C10 H14 N2	Full match	No results
TRUE	N6-(delta2-Isopentenyl)-adenosine5'-monophosphat	C15 H22 N5	No results	No results
TRUE			Unused	No results
TRUE	Leu-Pro(Leucyl-Proline)	C11 H20 N2	Full match	No results
TRUE			No results	No results

TRUE			Unused	No results
TRUE			Unused	No results
TRUE			Unused	No results
TRUE			No results	No results
TRUE			No results	No results
TRUE	Gly-Pro(Glycylproline)	C7 H12 N2 C	Full match	No results
TRUE			Unused	No results
TRUE	NP-008915	C15 H24 O3	No match	Invalid mass
TRUE	3-[(1-Carboxyvinyl)oxy]benzoate	C10 H8 O5	No match	No results
TRUE			No results	No results
TRUE			Unused	No results
TRUE			Unused	No results
TRUE			Unused	No results
TRUE			Unused	No results
TRUE			Unused	No results
TRUE			Unused	No results
TRUE			Unused	No results
TRUE			Unused	No results
TRUE			Unused	No results
TRUE			Unused	No results
TRUE	AicarMonophosphate;5-Aminoimidazole-4-carboxam	C9 H15 N4 C	Not the top	No results
TRUE	Citrinin	C13 H14 O5	Not the top	No results
TRUE			Unused	No results
TRUE			Unused	No results
TRUE			Unused	No results
TRUE			Unused	No results
TRUE			Unused	No results
TRUE	[FAtrihydroxy(18:0)]9_10_13-trihydroxy-11-octadece	C18 H34 O5	Full match	No results
TRUE			Unused	No results
TRUE	Phloretin	C15 H14 O5	Not the top	No results
TRUE			Unused	No results
TRUE	L-Aspartyl-4-phosphate	C4 H9 N O7	No match	No results
TRUE			Unused	No results
TRUE			Unused	No results
TRUE			Unused	No results
TRUE			Unused	No results
TRUE	Furfural;Furan-2-carbaldehyde	C5 H4 O2	Full match	No results
TRUE	Glucoiberverin	C11 H20 N C	No match	No results
TRUE			Unused	No results
TRUE			Unused	No results
TRUE			Unused	No results
TRUE			Unused	No results
TRUE			No results	No results
TRUE			Unused	No results
TRUE			Unused	No results
TRUE	2-aminoethylphosphonicacid	C2 H8 N O3	Full match	No results
TRUE	N-Butyryl-L-homoserinelactone	C8 H13 N O3	Full match	No results
TRUE			No results	No results
TRUE			Unused	No results
TRUE			No results	No results

TRUE	Thonningianin B	C35 H30 O1	Not the top	No results
TRUE	Saccharopine	C11 H20 N2	Not the top	No results
TRUE			Unused	No results
TRUE	(R)-Prunasin	C14 H17 N C	Not the top	No results
TRUE	Isoorientin 2"-O-gallate	C28 H24 O1	Not the top	No results
TRUE	Amfepramone	C13 H19 N C	Full match	No results
TRUE			Unused	No results
TRUE	DR 30	C18 H16 O7	Not the top	No results
TRUE			Unused	No results
TRUE			Unused	No results
TRUE	Gossypetin 3-O-sulfate	C15 H10 O1	Not the top	No results
TRUE			Unused	No results
TRUE			Unused	No results
TRUE	(-)threo-iso(homo)3citrate	C9 H14 O7	Full match	No results
TRUE			No results	No results
TRUE			Unused	No results
TRUE			Unused	No results
TRUE			Unused	No results
TRUE			Unused	No results
TRUE			Unused	No results
TRUE			Unused	No results
TRUE	Zosterin	C25 H28 O1	Not the top	No results
TRUE			Unused	No results
TRUE			Unused	No results
TRUE	Pseudosindorin	C15 H12 O5	Not the top	No results
TRUE			Invalid mass	No results
TRUE			Unused	No results
TRUE			No results	No results
TRUE			Unused	No results
TRUE			Unused	No results
TRUE			Unused	No results
TRUE			No results	No results
TRUE	Tridemorph	C19 H39 N C	Full match	No results
TRUE			Invalid mass	No results
TRUE	FL2FAAGC0001_a	C23 H18 O8	Not the top	No results
TRUE	Methyl trimellitate	C12 H12 O6	No results	No results
TRUE	Quercetin 4'-galactoside	C20 H18 O1	Full match	No results
TRUE			Unused	No results
TRUE	Maritimetin 6- (6"-p-coumarylglucoside)	C30 H26 O1	No match	No results
TRUE	Variabiloside A	C42 H46 O2	No match	No results
TRUE			Unused	No results
TRUE			Unused	No results
TRUE			Unused	No results
TRUE			Unused	No results
TRUE	Carbofuran, 3OH-	C12 H15 N C	Full match	No results
TRUE			No results	No results
TRUE			No results	No results
TRUE			Unused	No results
TRUE			Unused	No results
TRUE	7-Methoxy-4-methylcoumarin	C11 H10 O3	No match	No results

TRUE	Scutellarein 4'-methyl ether 7- (2",6"-diacetylalloside	C26 H26 O13	No match	No results
TRUE			Unused	No results
TRUE			Unused	No results
TRUE	Xanthine	C5 H4 N4 O2	Full match	No results
TRUE			Unused	No results
TRUE			Unused	No results
TRUE			Unused	No results
TRUE			Unused	No results
TRUE			Unused	No results
TRUE			No results	No results
TRUE			Unused	No results
TRUE			Unused	No results
TRUE			No results	No results
TRUE			Unused	No results
TRUE	FL5FFGGS0001_a	C16 H12 O9	Full match	No results
TRUE			Unused	No results
TRUE			Unused	No results
TRUE			Unused	No results
TRUE			No results	No results
TRUE			Unused	No results
TRUE			Unused	No results
TRUE			Unused	No results
TRUE			Unused	No results
TRUE			Unused	No results
TRUE	6-Pyruvoyltetrahydropterin	C9 H11 N5 C	Unused	No results
TRUE	Ggamma-L-Glutamyl-L-glutamicacid	C10 H16 N2	Full match	No results
TRUE			Unused	No results
TRUE			Unused	No results
TRUE	Carbofuran-3-Keto	C12 H13 N C	Full match	No results
TRUE			No results	No results
TRUE			No results	No results
TRUE	(S)-2-Acetolactate	C5 H8 O4	Full match	No results
TRUE			Unused	No results
TRUE			Unused	No results
TRUE			Unused	No results
TRUE			Unused	No results
TRUE	1-Methyladenosine	C11 H15 N5	Not the top	No results
TRUE			Unused	No results
TRUE	6-Hydroxyluteolin 7-sulfate	C15 H10 O1(	Not the top	No results
TRUE			Unused	No results
TRUE	Isoschaftoside 2"'-ferulate	C36 H36 O17	No results	No results
TRUE	3-Methyloxindole	C9 H9 N O	No results	No results
TRUE	7_10_13_16-Docosatetraenoylethanolamine	C24 H41 N C	Full match	No results
TRUE	5,7,3'-Trihydroxy-6,4',5'-trimethoxyflavanone	C18 H18 O8	No match	No results
TRUE			Unused	No results
TRUE	N-(Tetradecanoyl)-sphing-4-enine	C32 H63 N C	Not the top	No results
TRUE			Unused	No results
TRUE			Unused	No results
TRUE			No results	No results
TRUE			Unused	No results

TRUE	5,7,2',5'-Tetrahydroxy-6-methoxyflavanone	C16 H14 O7	Not the top	No results
TRUE			Unused	No results
TRUE			Unused	No results
TRUE	UNII:TYL476W27Y	C18 H30 O	Full match	No results
TRUE			Unused	No results
TRUE			Unused	No results
TRUE			Unused	No results
TRUE			Unused	No results
TRUE	Aesculin(Esculin)	C15 H16 O9	Not the top	No results
TRUE	Deoxycholicacid3-glucuronide	C30 H48 O1(	Full match	No results
TRUE			Unused	No results
TRUE			Unused	No results
TRUE			Unused	No results
TRUE			No results	No results
TRUE			Unused	No results
TRUE	8'-Hydroxyabscisate	C15 H20 O5	Full match	No results
TRUE			Unused	No results
TRUE	1-(beta-D-Ribofuranosyl)-1_4-dihydronicotinamide	C11 H16 N2	Full match	No results
TRUE			Unused	No results
TRUE			Unused	No results
TRUE	Orientin 7-O-sulfate	C21 H20 O1	No match	No results
TRUE			Unused	No results
TRUE			Unused	No results
TRUE	(9Z_15Z)-(13S)-12_13-Epoxyoctadeca-9_11_15-trien	C18 H28 O3	Full match	No results
TRUE			Unused	No results
TRUE			Unused	No results
TRUE			Unused	No results
TRUE			Unused	No results
TRUE			Unused	No results
TRUE	(1-Ribosylimidazole)-4-acetate	C10 H14 N2	No results	No results
TRUE			Unused	No results
TRUE			Unused	No results
TRUE			Unused	No results
TRUE	N-acetyl-DL-tryptophan	C13 H14 N2	Full match	No results
TRUE			Unused	No results
TRUE			Unused	No results
TRUE			No results	No results
TRUE			Unused	No results
TRUE			Unused	No results
TRUE	Zalcitabine	C9 H13 N3 C	Full match	No results
TRUE			Unused	No results
TRUE			Unused	No results
TRUE	Valethamate	C19 H31 N C	Full match	No results
TRUE	FL1ABGGS0001_a	C16 H12 O7	No match	No results
TRUE	3'-Deoxyderhamnosylmaysin	C21 H18 O9	Full match	No results
TRUE			Unused	No results
TRUE	2-C-Methyl-D-erythritol4-phosphate	C5 H13 O7 P	Not the top	No results
TRUE	Lacosamide	C13 H18 N2	Full match	No results
TRUE			Unused	No results
TRUE			Unused	No results

TRUE			Unused	No results
TRUE			Unused	No results
TRUE	Isoliquiritigenin 4,4'-diglucoside	C27 H32 O14	Full match	No results
TRUE			Unused	No results
TRUE	?-(Methylenecyclopropyl)glycine	C6 H9 N O2	Full match	No results
TRUE			Unused	No results
TRUE			No results	No results
TRUE			Unused	No results
TRUE	Hexadecanamide;Palmiticamide	C16 H33 N C	Full match	No results
TRUE	[6]-Gingerol	C17 H26 O4	Full match	No results
TRUE			Unused	No results
TRUE			Unused	No results
TRUE	Laxiflorin	C23 H26 O7	Full match	No results
TRUE			Unused	No results
TRUE	Patulin	C7 H6 O4	Full match	No results
TRUE			Unused	No results
TRUE	3_4-Dihydroxyphthalate	C8 H6 O6	Full match	No results
TRUE			Unused	No results
TRUE			Unused	No results
TRUE			Unused	No results
TRUE			Unused	No results
TRUE			Unused	No results
TRUE			Unused	No results
TRUE			Unused	No results
TRUE	N,N-Dimethyloctadecanamide	C20 H41 N C	Full match	No results
TRUE			Unused	No results
TRUE			Unused	No results
TRUE			Unused	No results
TRUE			No results	No results
TRUE			Unused	No results
TRUE			Unused	No results
TRUE	AicarMonophosphate;5-Aminoimidazole-4-carboxam	C9 H15 N4 C	Not the top	No results
TRUE			Unused	No results
TRUE			Unused	No results
TRUE	?-Linolenic Acid	C18 H30 O2	Not the top	Full match
TRUE			Unused	No results
TRUE			Unused	No results
TRUE			Unused	No results
TRUE			Unused	No results
TRUE			Unused	No results
TRUE			Unused	No results
TRUE			Unused	No results
TRUE	Chalconaringenin 2'-xyloside	C20 H20 O9	Not the top	No results
TRUE	2'_4'-Dihydroxyacetophenone	C8 H8 O3	Full match	No results
TRUE			Unused	No results
TRUE			Unused	No results
TRUE			Unused	No results
TRUE			Unused	No results
TRUE			Unused	No results
TRUE			Unused	No results

TRUE			Unused	No results
TRUE			Unused	No results
TRUE			Unused	No results
TRUE			Unused	No results
TRUE	Scoparone	C11 H10 O4	Full match	No results
TRUE			Unused	No results
TRUE			Unused	No results
TRUE			No results	No results
TRUE			No results	No results
TRUE			Unused	No results
TRUE	Lilaline	C20 H17 N C	No match	No results
TRUE	NP-020705	C17 H24 O8	No match	Invalid mass
TRUE	4,2',4'-Trihydroxy-6'-methoxychalcone 4,4'-di-beta-gl	C28 H34 O15	No match	No results
TRUE	Nothofagin	C21 H24 O1(	Not the top	No results
TRUE			No results	No results
TRUE			Unused	No results
TRUE			Unused	No results
TRUE			Unused	No results
TRUE			No results	No results
TRUE			Unused	No results
TRUE			Unused	No results
TRUE			Unused	No results
TRUE			Unused	No results
TRUE			Unused	No results
TRUE			Unused	No results
TRUE	Xanthurenicacid	C10 H7 N O4	Full match	No results
TRUE			Unused	No results
TRUE			No results	No results
TRUE			Unused	No results
TRUE	(S)-2-Acetolactate	C5 H8 O4	No results	No results
TRUE	NP-018716	C11 H20 O4	Full match	Full match
TRUE	Catechin-4-ol 3-O-beta-D-galactopyranoside	C21 H24 O12	No match	No results
TRUE			Unused	No results
TRUE			Unused	No results
TRUE	Dihydrofolicacid	C19 H21 N7	No results	No results
TRUE			Unused	No results
TRUE	2-Methylserine	C4 H9 N O3	Full match	No results
TRUE			Unused	No results
TRUE			Unused	No results
TRUE	Chalconaringenin 2'-xyloside	C20 H20 O9	No match	No results
TRUE			Unused	No results
TRUE			Unused	No results
TRUE			Unused	No results
TRUE			Unused	No results
TRUE			Unused	No results
TRUE			No results	No results
TRUE			Unused	No results
TRUE			Unused	No results
TRUE			Unused	No results
TRUE			Unused	No results

TRUE			Unused	No results
TRUE	3-Methyl-1-(2_4_6-trihydroxyphenyl)butan-1-one	C11 H14 O4	Full match	No results
TRUE			No results	No results
TRUE			Unused	No results
TRUE			Invalid mass	No results
TRUE			Unused	No results
TRUE			Unused	No results
TRUE			Unused	No results
TRUE			Unused	No results
TRUE	5,4'-Dihidroxy-6,7,3',5'-tetramethoxyflavone	C19 H18 O8	No match	No results
TRUE			Unused	No results
TRUE			Unused	No results
TRUE			Unused	No results
TRUE			Unused	No results
TRUE	3-(3_4-Dihydroxypyridin-1-yl)-L-alanine	C8 H12 N2 C	Not the top	No results
TRUE			Unused	No results
TRUE	Chrysin 7-glucuronide	C21 H18 O1(	Not the top	No results
TRUE			Unused	No results
TRUE			Unused	No results
TRUE	Scopolin	C16 H20 O9	Full match	No results
TRUE	FL1AAKGM0001_a	C19 H18 O7	No match	No results
TRUE			Unused	No results
TRUE			No results	No results
TRUE			No results	No results
TRUE			Unused	No results
TRUE			Unused	No results
TRUE			Unused	No results
TRUE			No results	No results
TRUE	2,6,3'-Trihydroxy-4'-methoxy-2-benzylcoumaranone	C16 H14 O6	Not the top	No results
TRUE			Unused	No results
TRUE	Ala-Tyr(Alanyltyrosine)	C12 H16 N2	Not the top	No results
TRUE	Bractein	C21 H20 O12	Not the top	No results
TRUE			Unused	No results
TRUE			Unused	No results
TRUE			Unused	No results
TRUE			Unused	No results
TRUE			Unused	No results
TRUE			No results	No results
TRUE			Unused	No results
TRUE			Unused	No results
TRUE			Unused	No results
TRUE			No results	No results
TRUE			Unused	No results
TRUE	2,2,10,10-Tetramethyl-3,6,9-trioxa-2,10-disilaundeca	C10 H26 O3	No match	No results
TRUE	Lacosamide	C13 H18 N2	Full match	No results
TRUE			Unused	No results
TRUE			Unused	No results
TRUE	Ala-Tyr(Alanyltyrosine)	C12 H16 N2	Not the top	No results
TRUE			Unused	No results
TRUE			Unused	No results

TRUE			No results	No results
TRUE			Unused	No results
TRUE			Invalid mass	No results
TRUE			Unused	No results
TRUE			No results	No results
TRUE			Unused	No results
TRUE			Unused	No results
TRUE			Unused	No results
TRUE			Unused	No results
TRUE	(S)-Abscisicacid	C15 H20 O4	Not the top	No results
TRUE			Unused	No results
TRUE	Monospermoside	C21 H22 O1(	No results	No results
TRUE	(R)-3-(4-Hydroxyphenyl)lactate	C9 H10 O4	Full match	No results
TRUE	Apigenin 7- (6"-crotonylglucoside)	C25 H24 O1:	Not the top	No results
TRUE	Clavulanicacid	C8 H9 N O5	Not the top	No results
TRUE			No results	No results
TRUE	2-Propenoic acid, 2-phenoxyethyl ester	C11 H12 O3	Full match	No results
TRUE			Unused	No results
TRUE			Unused	No results
TRUE	octyl methoxycinnamate	C18 H26 O3	Full match	No results
TRUE			Unused	No results
TRUE			Unused	No results
TRUE			Unused	No results
TRUE			Unused	No results
TRUE			Unused	No results
TRUE	Cerarvensin	C20 H18 O9	Not the top	No results
TRUE			Unused	No results
TRUE			Unused	No results
TRUE			Unused	No results
TRUE	(1-Ribosylimidazole)-4-acetate	C10 H14 N2	Not the top	No results
TRUE			Unused	No results
TRUE			Unused	No results
TRUE			No results	No results
TRUE	2"-trans-Caffeoylisoorientin	C30 H26 O14	Not the top	No results
TRUE			Unused	No results
TRUE			Unused	No results
TRUE			Unused	No results
TRUE			Unused	No results
TRUE			Unused	No results
TRUE			No results	No results
TRUE			Unused	No results
TRUE			No results	No results
TRUE			No results	No results
TRUE			Unused	No results
TRUE			Unused	No results
TRUE	Napropamide	C17 H21 N C	No results	No results
TRUE			Unused	No results
TRUE			Unused	No results
TRUE			Unused	No results
TRUE			No results	No results

TRUE			Unused	No results
TRUE			Unused	No results
TRUE	NP-008515	C15 H20 O4	Full match	Full match
TRUE			No results	No results
TRUE			Unused	No results
TRUE			Unused	No results
TRUE			Unused	No results
TRUE			Unused	No results
TRUE			Unused	No results
TRUE			No results	No results
TRUE	Kinetin;N-Furfuryl-Adenine	C10 H9 N5 C	Full match	No results
TRUE			Unused	No results
TRUE			Unused	No results
TRUE	6-Hydroxyluteolin-7- (6'''-p-coumarylsophoroside)	C36 H36 O19	No match	No results
TRUE			Unused	No results
TRUE	2-Deamino-2-hydroxy-6-dehydroparomamine	C12 H22 N2	No results	No results
TRUE			No results	No results
TRUE			Unused	No results
TRUE			Unused	No results
TRUE			Unused	No results
TRUE	7,8,4'-Trihydroxy-3',5'-dimethoxyflavanone 4'-O-gluc	C23 H26 O12	Full match	No results
TRUE			Unused	No results
TRUE			Unused	No results
TRUE			Unused	No results
TRUE			Unused	No results
TRUE			Unused	No results
TRUE	2'-Deoxymugineicacid	C12 H20 N2	Not the top	No results
TRUE			Unused	No results
TRUE			Unused	No results
TRUE			Unused	No results
TRUE	3,5,7,3',4',5'-Hexahydroxy-6,8-dimethylflavanone	C17 H16 O8	Not the top	No results
TRUE			Unused	No results
TRUE	[STtrihydrox]3alpha_11beta_21-5alpha-trihydroxy-pr	C21 H34 O4	Full match	No results
TRUE			Unused	No results
TRUE	dTDP-4-dehydro-2,6-dideoxy-β-L-galactose	C16 H24 N2	Not the top	No results
TRUE			No results	No results
TRUE	1-Methyl-3-Phenylpiperazine	C11 H16 N2	Full match	No results
TRUE			Unused	No results
TRUE			Unused	No results
TRUE	Isoorientin 2"-O-gallate	C28 H24 O15	Full match	No results
TRUE			Unused	No results
TRUE	HT2-Toxin	C22 H32 O8	Not the top	No results
TRUE	Okanin 3,4,3',4'-tetramethyl ether	C19 H20 O6	Not the top	No results
TRUE	Pyridostigmine	C9 H12 N2 C	Full match	No results
TRUE			Unused	No results
TRUE			No results	No results
TRUE			Unused	No results
TRUE			Unused	No results
TRUE	Chrysin 7-glucuronide	C21 H18 O1(	Not the top	No results
TRUE			Unused	No results

TRUE	Chorismate	C10 H10 O6	Full match	No results
TRUE	3'-Amino-3'-deoxy-AMP	C10 H15 N6	No match	No results
TRUE	Taxillusin	C28 H26 O16	Not the top	No results
TRUE			Unused	No results
TRUE			No results	No results
TRUE			Unused	No results
TRUE			Unused	No results
TRUE			No results	No results
TRUE			Invalid mass	No results
TRUE			Unused	No results
TRUE			Unused	No results
TRUE			No results	No results
TRUE	3,4,2',3',4',6',alpha-Heptahydroxychalcone 2'-glucosi	C21 H22 O13	Full match	No results
TRUE			Unused	No results
TRUE	Maritimetin 6- (6"-p-coumarylglucoside)	C30 H26 O13	Not the top	No results
TRUE			No results	No results
TRUE			Unused	No results
TRUE			Unused	No results
TRUE			Unused	No results
TRUE			Unused	No results
TRUE			Unused	No results
TRUE			Unused	No results
TRUE			Unused	No results
TRUE			No results	No results
TRUE			Unused	No results
TRUE			Unused	No results
TRUE			Unused	No results
TRUE			Unused	No results
TRUE	Hippuric Acid	C9 H9 N O3	Full match	No results
TRUE			Unused	No results
TRUE			Unused	No results
TRUE	Triethyl citrate	C12 H20 O7	Not the top	No results
TRUE	Gabapentin	C9 H17 N O2	Full match	No results
TRUE			No results	No results
TRUE	Kaempferol 3- (2"-p-coumaryl-alpha-L-arabinopyrano	C29 H24 O12	No match	No results
TRUE			Invalid mass	No results
TRUE			Unused	No results
TRUE	N-Acetyl-D-glucosaminate	C8 H15 N O7	Full match	No results
TRUE			Unused	No results
TRUE			No results	No results
TRUE			Unused	No results
TRUE			Unused	No results
TRUE			Unused	No results
TRUE	(2R_3S)-2_3-Dimethylmalate	C6 H10 O5	Full match	No results
TRUE			Unused	No results
TRUE	Catechin 5,3'-di-O-beta-D-glucopyranoside	C27 H34 O16	No match	No results
TRUE	Chrysin 7-glucuronide	C21 H18 O10	No match	No results
TRUE			Unused	No results
TRUE			No results	No results
TRUE	4-dimethylaminobenzophenone	C15 H15 N O	Full match	No results

TRUE			Unused	No results
TRUE			Unused	No results
TRUE			Unused	No results
TRUE			No results	No results
TRUE			Unused	No results
TRUE			Unused	No results
TRUE			Unused	No results
TRUE	3,5,7,3',4',5'-Hexahydroxy-6,8-dimethylflavanone	C17 H16 O8	Not the top	No results
TRUE	1-Salicylateglucuronide	C13 H14 O9	Full match	No results
TRUE			Unused	No results
TRUE	3,4,2',3',4',6',alpha-Heptahydroxychalcone 2'-glucosi	C21 H22 O13	No match	No results
TRUE	Mebutamate	C10 H20 N2	Full match	No results
TRUE			Unused	No results
TRUE	Maritimein	C21 H20 O1	No match	No results
TRUE			Unused	No results
TRUE			Unused	No results
TRUE	DIMETHYLDIPHENYLMETHANE	C15 H16	No results	No results
TRUE	5-Amino-6-(5'-phosphoribitylamino)uracil	C9 H17 N4 C	No match	No results
TRUE			Unused	No results
TRUE			Unused	No results
TRUE			Unused	No results
TRUE			Unused	No results
TRUE	2'_3'-CyclicGMP	C10 H12 N5	No match	No results
TRUE			Unused	No results
TRUE	7,9-di-tert-butyl-1-oxaspiro[4.5]deca-6,9-diene-2,8-d	C17 H24 O3	Full match	No results
TRUE	4-Hydroxyphenylacetylglutamicacid	C13 H15 N C	Full match	No results
TRUE			Unused	No results
TRUE			Unused	No results
TRUE			Unused	No results
TRUE			Unused	No results
TRUE			Unused	No results
TRUE			Unused	No results
TRUE			Unused	No results
TRUE			Unused	No results
TRUE			Unused	No results
TRUE			Unused	No results
TRUE			Unused	No results
TRUE			No results	No results
TRUE			Unused	No results
TRUE	Dihexyl azelaate	C21 H40 O4	Full match	No results
TRUE			Unused	No results
TRUE	Erucic amide	C22 H43 N C	Full match	No results
TRUE	6-Acetamido-3-aminohexanoate	C8 H16 N2 C	Full match	No results
TRUE			Unused	No results
TRUE			Unused	No results
TRUE	Dethiobiotin	C10 H18 N2	Full match	No results
TRUE			Unused	No results
TRUE			Unused	No results
TRUE			Unused	No results
TRUE			Unused	No results

TRUE			No results	No results
TRUE			No results	No results
TRUE			Unused	No results
TRUE			Unused	No results
TRUE			Unused	No results
TRUE	Lupinate	C13 H18 N6	No results	No results
TRUE			No results	No results
TRUE			Unused	No results
TRUE	2',5'-Dihydroxy-4,3',4',6'-tetramethoxychalcone	C19 H20 O7	No match	No results
TRUE			Unused	No results
TRUE	Rubone	C20 H22 O7	Full match	No results
TRUE			No results	No results
TRUE			Unused	No results
TRUE			Unused	No results
TRUE			Unused	No results
TRUE			Unused	No results
TRUE	N,N-Dimethyloctadecanamide	C20 H41 N C	Full match	No results
TRUE			Unused	No results
TRUE			Unused	No results
TRUE	Isophthalic acid	C8 H6 O4	Full match	No results
TRUE			Unused	No results
TRUE			Unused	No results
TRUE	[STtrihydrox]3alpha_11beta_21-5alpha-trihydroxy-pr	C21 H34 O4	Full match	No results
TRUE			Unused	No results
TRUE			Unused	No results
TRUE			No results	No results
TRUE			No results	No results
TRUE			No results	No results
TRUE			Unused	No results
TRUE			Unused	No results
TRUE			Unused	No results
TRUE			Unused	No results
TRUE			Unused	No results
TRUE			Unused	No results
TRUE			No results	No results
TRUE			Unused	No results
TRUE			Unused	No results
TRUE			Unused	No results
TRUE			Unused	No results
TRUE			Unused	No results
TRUE			Unused	No results
TRUE	Ecgonine	C9 H15 N O3	No results	No results
TRUE			Unused	No results
TRUE	2,2'-[(4-Methylphenyl)imino]diethanol	C11 H17 N C	No match	No results
TRUE			Unused	No results
TRUE			Unused	No results
TRUE			Unused	No results
TRUE			Invalid mass	No results
TRUE	2-Hydroxy-3-(4-hydroxyphenyl)propenoate	C9 H8 O4	Full match	No results
TRUE			Unused	No results

TRUE	Isopropalin	C15 H23 N3	Full match	No results
TRUE			Unused	No results
TRUE	Tolmetin	C15 H15 N C	Full match	No results
TRUE			Unused	No results
TRUE			Unused	No results
TRUE	Gallicacid	C7 H6 O5	Full match	No results
TRUE			Unused	No results
TRUE			Unused	No results
TRUE			Unused	No results
TRUE			Unused	No results
TRUE			Unused	No results
TRUE			Unused	No results
TRUE			Unused	No results
TRUE			Unused	No results
TRUE			Unused	No results
TRUE	2-Furoylglycine;Pyromucuricacid	C7 H7 N O4	Full match	No results
TRUE			Unused	No results
TRUE			Unused	No results
TRUE			Unused	No results
TRUE	N6-(delta2-Isopentenyl)-adenosine5'-monophosphat	C15 H22 N5	No results	No results
TRUE			Unused	No results
TRUE			Unused	No results
TRUE			Unused	No results
TRUE			Unused	No results
TRUE			Unused	No results
TRUE			Unused	No results
TRUE			Unused	No results
TRUE			Unused	No results
TRUE	Elephantorrhizol	C15 H14 O8	Full match	No results
TRUE	Styrene	C8 H8	Full match	No results
TRUE			Unused	No results
TRUE			Unused	No results
TRUE	Pyrazoxyfen	C20 H16 Cl2	No match	No results
TRUE	Carbinoxamine	C16 H19 Cl N	No match	No results
TRUE			Unused	No results
TRUE	5,7,2',5'-Tetrahydroxy-6-methoxyflavanone	C16 H14 O7	Not the top	No results
TRUE			No results	No results
TRUE			Unused	No results
TRUE			Unused	No results
TRUE			Unused	No results
TRUE			Invalid mass	No results
TRUE			Unused	No results
TRUE			Unused	No results
TRUE	3-Ketolactose	C12 H20 O1	Not the top	No results
TRUE			Unused	No results
TRUE			Unused	No results
TRUE			Unused	No results
TRUE	Acetyl-N-formyl-5-methoxykynurenamine	C13 H16 N2	Not the top	No results
TRUE			Unused	No results
TRUE			Unused	No results

TRUE			Unused	No results
TRUE			Unused	No results
TRUE			Unused	No results
TRUE	Lotaustralin	C11 H19 N C	Not the top	No results
TRUE			No results	No results
TRUE			No results	No results
TRUE			No results	No results
TRUE			Unused	No results
TRUE	2_3_4_5-Tetrahydrodipicolinate	C7 H9 N O4	Full match	No results
TRUE	3alpha_11beta-Dihydroxy-5alpha-androstane-17-one	C19 H30 O3	Full match	No results
TRUE			Unused	No results
TRUE	5-Hydroxy-DL-tryptophan	C11 H12 N2	Full match	No results
TRUE			Unused	No results
TRUE	5,7,4'-Trihydroxyflavanone 7-sulfate	C15 H12 O8	Not the top	No results
TRUE	Proclavaminicacid	C8 H14 N2 C	Full match	No results
TRUE	Isorhamnetin 3- (6"-malonylglucoside)	C25 H24 O15	No match	No results
TRUE			Unused	No results
TRUE	Carbofuran-3-Keto	C12 H13 N C	Not the top	No results
TRUE	2-Hydroxy-3-(4-hydroxyphenyl)propenoate	C9 H8 O4	Not the top	No results
TRUE			Unused	No results
TRUE			Unused	No results
TRUE			No results	No results
TRUE			Unused	No results
TRUE	Clavulanicacid	C8 H9 N O5	No match	No results
TRUE	1-Pyrroline-4-hydroxy-2-carboxylate	C5 H7 N O3	No results	No results
TRUE			Unused	No results
TRUE			Unused	No results
TRUE			Unused	No results
TRUE			Unused	No results
TRUE			Unused	No results
TRUE			Unused	No results
TRUE			Unused	No results
TRUE			Unused	No results
TRUE			Unused	No results
TRUE			Unused	No results
TRUE	Discadenine	C14 H20 N6	No results	No results
TRUE			Unused	No results
TRUE			No results	No results
TRUE			Unused	No results
TRUE			No results	No results
TRUE			Unused	No results
TRUE			Unused	No results
TRUE			Unused	No results
TRUE			Unused	No results
TRUE			Invalid mass	No results
TRUE			No results	No results
TRUE			Unused	No results
TRUE			Unused	No results
TRUE	2_3'_4_6-Tetrahydroxybenzophenone	C13 H10 O5	Full match	No results
TRUE	Epigallocatechin 3,5,-di-O-gallate	C29 H22 O15	Full match	No results

TRUE			No results	No results
TRUE			Unused	No results
TRUE			Unused	No results
TRUE			No results	No results
TRUE			Unused	No results
TRUE			Unused	No results
TRUE	3-(3_4-Dihydroxypyridin-1-yl)-L-alanine	C8 H12 N2 C	Full match	No results
TRUE			Unused	No results
TRUE			Unused	No results
TRUE			Unused	No results
TRUE			No results	No results
TRUE			Unused	No results
TRUE	5,7-Dimethoxy-6-C-methylflavone	C18 H16 O4	Full match	No results
TRUE			Unused	No results
TRUE			No results	No results
TRUE	Isorhamnetin 3- (6"-malonylglucoside)	C25 H24 O15	No match	No results
TRUE	Cerarvensin	C20 H18 O9	No match	No results
TRUE			Unused	No results
TRUE			Unused	No results
TRUE			No results	No results
TRUE			Unused	No results
TRUE			Unused	No results
TRUE			Unused	No results
TRUE			No results	No results
TRUE			Unused	No results
TRUE			Unused	No results
TRUE			Unused	No results
TRUE			Unused	No results
TRUE			Unused	No results
TRUE			Unused	No results
TRUE			Unused	No results
TRUE	dehypoxanthinefutalosine	C14 H16 O7	Full match	No results
TRUE			No results	No results
TRUE			Unused	No results
TRUE			Unused	No results
TRUE			Unused	No results
TRUE			Unused	No results
TRUE			Unused	No results
TRUE	2,2'-(Tridecylimino)diethanol	C17 H37 N C	Full match	No results
TRUE	Isovitexin 2"-O- (6"'- (E) -p-coumaroyl) glucoside 4'-O	C42 H46 O22	Not the top	No results
TRUE			Unused	No results
TRUE			Unused	No results
TRUE			Unused	No results
TRUE			Unused	No results
TRUE	5,3'-Dihydroxy-6,7,4',5'-tetramethoxyflavanone	C19 H20 O8	No match	No results
TRUE	N-D-Glucosylarylamine	C12 H17 N C	No match	No results
TRUE			Unused	No results
TRUE			Unused	No results
TRUE			Unused	No results
TRUE	6-Methoxyluteolin 7-glucoside	C22 H22 O12	No match	No results

TRUE	5-(2'-Carboxyethyl)-4_6-dihydroxypicolinate	C9 H9 N O6	Not the top	No results
TRUE			No results	No results
TRUE			Unused	No results
TRUE	(2E) -5,7-Dihydroxy-3,6-dimethoxy-4-oxo-2-phenyl-4I	C22 H20 O8	Not the top	No results
TRUE			Unused	No results
TRUE			Unused	No results
TRUE	Remerin	C17 H14 O7	Not the top	No results
TRUE			Unused	No results
TRUE			Unused	No results
TRUE			Unused	No results
TRUE			Unused	No results
TRUE			Unused	No results
TRUE	[STtrihydrox]3alpha_11beta_21-5alpha-trihydroxy-pr	C21 H34 O4	Full match	No results
TRUE			Unused	No results
TRUE			Unused	No results
TRUE			Unused	No results
TRUE			No results	No results
TRUE			Unused	No results
TRUE			Unused	No results
TRUE			Unused	No results
TRUE	[FA(18:1)]9Z-Octadecen-12-ynoicacid	C18 H30 O2	Full match	No results
TRUE	Kaempferol 3- (2",6"-di- (E) -p-coumarylglucoside)	C39 H32 O15	Full match	No results
TRUE			No results	No results
TRUE			No results	No results
TRUE			Unused	No results
TRUE			Unused	No results
TRUE	N4-Acetylcytidine;N-Acetyl-Cytidine	C11 H15 N3	Not the top	No results
TRUE	AZT	C10 H13 N5	Not the top	No results
TRUE			Unused	No results
TRUE	Telephioidin	C21 H20 O13	No match	No results
TRUE			Unused	No results
TRUE			Unused	No results
TRUE			Unused	No results
TRUE	Rubone	C20 H22 O7	Not the top	No results
TRUE			Unused	No results
TRUE			Unused	No results
TRUE			Unused	No results
TRUE			Unused	No results
TRUE			Unused	No results
TRUE			Unused	No results
TRUE			Unused	No results
TRUE			Unused	No results
TRUE			Unused	No results
TRUE	Linoleic Acid	C18 H32 O2	Full match	Full match
TRUE	Edrophonium	C10 H15 N C	Full match	No results
TRUE			No results	No results
TRUE			Unused	No results
TRUE	5-Hydroxy-DL-tryptophan	C11 H12 N2	Full match	No results
TRUE			Unused	No results
TRUE			Unused	No results

TRUE			Unused	No results
TRUE			Unused	No results
TRUE	2,5,8,11,14,17,20,23-Octaoxapentacosan-25-ol	C17 H36 O9	Not the top	No results
TRUE			No results	No results
TRUE			Unused	No results
TRUE			Unused	No results
TRUE	FL2FAAGC0001_a	C23 H18 O8	Not the top	No results
TRUE	DR 30	C18 H16 O7	Full match	No results
TRUE			Unused	No results
TRUE			No results	No results
TRUE			Unused	No results
TRUE	1,1,1,5,5,5-Hexamethyl-3-[(trimethylsilyl)oxy]trisiloxa	C9 H28 O3 S	No match	No results
TRUE			Unused	No results
TRUE			Unused	No results
TRUE	Linderoflavone A	C18 H14 O8	Full match	No results
TRUE			Unused	No results
TRUE	Silybin	C25 H22 O1(	No match	No results
TRUE	Glabrachalcone	C23 H24 O6	Full match	No results
TRUE			Unused	No results
TRUE			Unused	No results
TRUE			Unused	No results
TRUE			Unused	No results
TRUE			Unused	No results
TRUE			Unused	No results
TRUE			No results	No results
TRUE			Unused	No results
TRUE	FL1ABGGS0001_a	C16 H12 O7	Not the top	No results
TRUE			Unused	No results
TRUE	7,9-di-tert-butyl-1-oxaspiro[4.5]deca-6,9-diene-2,8-d	C17 H24 O3	Full match	No results
TRUE			Unused	No results
TRUE			Unused	No results
TRUE			Unused	No results
TRUE			Unused	No results
TRUE			Unused	No results
TRUE			Unused	No results
TRUE			Unused	No results
TRUE			No results	No results
TRUE			Unused	No results
TRUE	Epigallocatechin 3-O- (3-O-methylgallate)	C23 H20 O1	Not the top	No results
TRUE			Unused	No results
TRUE			Invalid mass	No results
TRUE	Pedicellin	C20 H22 O6	No match	No results
TRUE			Unused	No results
TRUE	Diethylene glycol n-butyl ether	C8 H18 O3	Full match	No results
TRUE			Unused	No results
TRUE			Unused	No results
TRUE	Crotono-betaine	C8 H15 N O	Full match	No results
TRUE	2-Hydroxyflemichapparin C	C17 H10 O7	Not the top	No results
TRUE	Isoproturon	C12 H18 N2	Full match	No results
TRUE			No results	No results

TRUE			Unused	No results
TRUE			Unused	No results
TRUE			No results	No results
TRUE			Unused	No results
TRUE			Unused	No results
TRUE			Unused	No results
TRUE			Unused	No results
TRUE			Unused	No results
TRUE			Unused	No results
TRUE			Unused	No results
TRUE			Unused	No results
TRUE			Unused	No results
TRUE			Unused	No results
TRUE			Unused	No results
TRUE			No results	No results
TRUE			Unused	No results
TRUE	Dihydrolipoamide	C8 H17 N O S	No match	No results
TRUE	Gallicacid	C7 H6 O5	Full match	No results
TRUE			Unused	No results
TRUE			Unused	No results
TRUE	Brosimacutin G	C20 H20 O6	Unused	No results
TRUE			Unused	No results
TRUE			Unused	No results
TRUE			Unused	No results
TRUE			Unused	No results
TRUE	Chlorpyrifos-methyl	C7 H7 Cl3 N	No match	No results
TRUE			No results	No results
TRUE			Unused	No results
TRUE			Unused	No results
TRUE	Citicoline;cytidine5'diphosphocholine	C14 H26 N4	No results	No results
TRUE	Capryloylglycine	C10 H19 N C	Full match	No results
TRUE			Unused	No results
TRUE	Taxifolin 3-apioside	C20 H20 O1	No results	No results
TRUE			No results	No results
TRUE			Unused	No results
TRUE			No results	No results
TRUE	N-Feruloylglycine	C12 H13 N C	Not the top	No results
TRUE			Unused	No results
TRUE	Phenylglyoxylate	C8 H6 O3	Full match	No results
TRUE			Unused	No results
TRUE			No results	No results
TRUE	2-Succinyl-5-enolpyruvyl-6-hydroxy-3-cyclohexene-1-	C14 H16 O9	Not the top	No results
TRUE			Unused	No results
TRUE			Unused	No results
TRUE			Unused	No results
TRUE			Unused	No results
TRUE			No results	No results
TRUE			Unused	No results
TRUE			Unused	No results
TRUE			Unused	No results

TRUE	2-Phytyl-1_4-naphthoquinone	C30 H44 O2	Full match	No results
TRUE			Unused	No results
TRUE			No results	No results
TRUE	2-Methylguanosine	C11 H15 N5	Not the top	No results
TRUE	9,10-Dihydro-10- (4-hydroxyphenyl) -pyrano [2,3-h]	C24 H20 O8	Not the top	No results
TRUE			Unused	No results
TRUE	2,5-Dihydroxybenzoate 2-O-β-D-glucoside	C13 H16 O9	Full match	No results
TRUE			Unused	No results
TRUE			Unused	No results
TRUE	Scutellarein 7- (6"-malonylglucoside)	C24 H22 O1	No match	No results
TRUE			No results	No results
TRUE	1_2-beta-D-Glucuronosyl-D-glucuronate	C12 H18 O1	No match	No results
TRUE			Unused	No results
TRUE			Unused	No results
TRUE			Unused	No results
TRUE			Unused	No results
TRUE	5-Hydroxy-DL-tryptophan	C11 H12 N2	Invalid mass	No results
TRUE			Unused	No results
TRUE			Unused	No results
TRUE	Sphinganine;2-Amion-1_3-octadecanediol	C18 H39 N C	Full match	No results
TRUE			Unused	No results
TRUE			Unused	No results
TRUE	SORBITOL HEXAACETATE	C18 H26 O1	No results	No results
TRUE			Unused	No results
TRUE			No results	No results
TRUE			Unused	No results
TRUE			Unused	No results
TRUE			Unused	No results
TRUE			Unused	No results
TRUE	Naphthalen-2-amine	C10 H9 N	Full match	No results
TRUE	1_5-Anhydro-D-mannitol	C6 H12 O5	Full match	No results
TRUE			Unused	No results
TRUE	N-lauroylethanolamine	C14 H29 N C	Full match	No results
TRUE	Chrysin 5-xyloside	C20 H18 O8	Full match	No results
TRUE			Unused	No results
TRUE	N-Desmethylvenlafaxine	C16 H25 N C	Not the top	No results
TRUE			Unused	No results
TRUE			Unused	No results
TRUE			Unused	No results
TRUE	Napropamide	C17 H21 N C	No results	No results
TRUE			No results	No results
TRUE	FL2FAAGC0001_a	C23 H18 O8	Full match	No results
TRUE			Unused	No results
TRUE	2_6-Dioxo-6-phenylhexanoate	C12 H12 O4	Full match	No results
TRUE			No results	No results
TRUE			Unused	No results
TRUE			Unused	No results
TRUE			Unused	No results
TRUE			Unused	No results
TRUE	Vitexin 2"-p-hydroxybenzoate	C28 H24 O1	No match	No results

TRUE			Unused	No results
TRUE			Unused	No results
TRUE	4-(beta-D-Glucosyloxy)benzoate	C13 H16 O8	No match	No results
TRUE	2-Carboxy-2_3-dihydro-5_6-dihydroxyindole	C9 H9 N O4	Not the top	No results
TRUE			Unused	No results
TRUE			Unused	No results
TRUE			Unused	No results
TRUE			No results	No results
TRUE			Unused	No results
TRUE			Unused	No results
TRUE	2,4,6-TRIS(ALLYLOXY)-S-TRIAZINE	C12 H15 N3	Full match	No results
TRUE	Artonin L	C22 H20 O7	Not the top	No results
TRUE	Actinorhodine	C32 H26 O1	Full match	No results
TRUE			Unused	No results
TRUE	Anatabine	C10 H12 N2	Full match	No results
TRUE	Naphthalen-2-amine	C10 H9 N	Full match	No results
TRUE	Geranylacetate	C12 H20 O2	Full match	No results
TRUE			Unused	No results
TRUE	Aminocyclopyrachlor	C8 H8 Cl N3	No match	No results
TRUE	Flucarbazone	C12 H11 F3 I	No match	No results
TRUE			Unused	No results
TRUE			Unused	No results
TRUE	Scutellarein 7-glucuronide-6-ferulate	C31 H26 O1	No match	No results
TRUE	Desmethyl Nefopam	C16 H17 N C	Full match	No results
TRUE	Stipitatate	C8 H6 O5	Invalid mass	No results
TRUE			Unused	No results
TRUE			Unused	No results
TRUE	Quercetin 7- [xylosyl- (1->2) -rhamnosyl- (1->2) -rhar	C38 H48 O2	No match	No results
TRUE			Unused	No results
TRUE	Clavulanicacid	C8 H9 N O5	Full match	No results
TRUE			Unused	No results
TRUE	Mycothiol	C17 H30 N2	Not the top	No results
TRUE			Unused	No results
TRUE			Unused	No results
TRUE	Quercetin 3-isobutyrate	C19 H16 O8	Full match	No results
TRUE			Unused	No results
TRUE			Unused	No results
TRUE			Unused	No results
TRUE			Unused	No results
TRUE	Sigmoidin G	C20 H20 O8	Full match	No results
TRUE			Unused	No results
TRUE	Fenuron	C9 H12 N2 C	No results	No results
TRUE	[6]-Gingerol	C17 H26 O4	Full match	No results
TRUE			Unused	No results
TRUE			Unused	No results
TRUE			Unused	No results
TRUE			Unused	No results
TRUE			Unused	No results
TRUE			Unused	No results
TRUE	Hoslundin	C23 H18 O7	Not the top	No results

TRUE			No results	No results
TRUE			Unused	No results
TRUE	dTDP-4-dehydro-2,6-dideoxy-β-L-galactose	C16 H24 N2	Not the top	No results
TRUE			Unused	No results
TRUE			Unused	No results
TRUE			Unused	No results
TRUE			Unused	No results
TRUE	Cerarvensin	C20 H18 O9	Not the top	No results
TRUE			Unused	No results
TRUE			Unused	No results
TRUE	Cortol	C21 H36 O5	Full match	No results
TRUE	Taxifolin 3'- (6"-phenylacetylglucoside)	C29 H28 O13	Not the top	No results
TRUE			Unused	No results
TRUE	1-Salicylateglucuronide	C13 H14 O9	No match	No results
TRUE			Unused	No results
TRUE			Unused	No results
TRUE	Nabumetone	C15 H16 O2	Full match	No results
TRUE	Remerin	C17 H14 O7	Not the top	No results
TRUE			Unused	No results
TRUE			No results	No results
TRUE			Unused	No results
TRUE			Unused	No results
TRUE			Unused	No results
TRUE			No results	No results
TRUE			Unused	No results
TRUE			Unused	No results
TRUE			Unused	No results
TRUE			Unused	No results
TRUE			Unused	No results
TRUE			No results	No results
TRUE			Unused	No results
TRUE			Unused	No results
TRUE			Unused	No results
TRUE			No results	No results
TRUE			Unused	No results
TRUE			Unused	No results
TRUE	3'-CMP	C9 H14 N3 C	No results	No results
TRUE			Unused	No results
TRUE			Unused	No results
TRUE			Unused	No results
TRUE			Unused	No results
TRUE			Unused	No results
TRUE			Unused	No results
TRUE			Unused	No results
TRUE			Unused	No results
TRUE			Unused	No results
TRUE	Kaempferol 3- (2"- (E) -p-coumaroyl-alpha-L-arabinof	C35 H34 O16	No match	No results
TRUE			Invalid mass	No results
TRUE			Unused	No results
TRUE			Unused	No results

TRUE	Quercetin 3- (2"-caffeylglucuronide)	C30 H24 O16	No match	No results
TRUE			Unused	No results
TRUE			Unused	No results
TRUE	4-Amino-4-deoxychorismate	C10 H11 N C	No match	No results
TRUE			Unused	No results
TRUE			Unused	No results
TRUE			Unused	No results
TRUE	Prilocaine	C13 H20 N2	Full match	No results
TRUE	alpha-Tocotrienol	C29 H44 O2	Full match	No results
TRUE			Unused	No results
TRUE			Unused	No results
TRUE	3-Dimethylallyl-4-hydroxyphenylpyruvate	C14 H16 O4	Full match	No results
TRUE	2-coumarate	C9 H8 O3	No match	No results
TRUE			Unused	No results
TRUE			Unused	No results
TRUE			Unused	No results
TRUE			Unused	No results
TRUE			Unused	No results
TRUE			Unused	No results
TRUE	UNII:DUA9665YBG	C13 H18 O2	Full match	No results
TRUE			Unused	No results
TRUE			Unused	No results
TRUE			Unused	No results
TRUE			Unused	No results
TRUE			Unused	No results
TRUE			No results	No results
TRUE	Kaempferol 3- (4"-acetyl-6"-p-coumarylglucoside)	C32 H28 O14	No match	No results
TRUE			Unused	No results
TRUE			Unused	No results
TRUE	Botrydial	C17 H26 O5	Not the top	No results
TRUE	sn-glycero-3-Phosphocholine	C8 H20 N O6	Not the top	No results
TRUE	Benzyl2-methyl-3-oxobutanoate	C12 H14 O3	Full match	No results
TRUE			Unused	No results
TRUE	Pongamoside A	C23 H20 O9	No match	No results
TRUE			Unused	No results
TRUE			Unused	No results
TRUE			Unused	No results
TRUE			Unused	No results
TRUE			No results	No results
TRUE			Unused	No results
TRUE			Unused	No results
TRUE			Unused	No results
TRUE			No results	No results
TRUE			Unused	No results
TRUE			Unused	No results
TRUE			Unused	No results
TRUE	Alminoprofen	C13 H17 N C	No match	No results
TRUE	Citrinin	C13 H14 O5	Not the top	No results
TRUE			Unused	No results
TRUE	6-C-Xylosylluteolin	C20 H18 O10	Not the top	No results

TRUE			Unused	No results
TRUE			No results	No results
TRUE			Unused	No results
TRUE			Unused	No results
TRUE			Unused	No results
TRUE	FL2F3GGS0001_a	C17 H16 O7	Not the top	No results
TRUE			No results	No results
TRUE			Unused	No results
TRUE			Unused	No results
TRUE	Maritimetin 6- (6"-acetylglucoside)	C23 H22 O12	Not the top	No results
TRUE			Unused	No results
TRUE			Unused	No results
TRUE			Unused	No results
TRUE			Unused	No results
TRUE			Unused	No results
TRUE	Quercetin 3- (2"-galloylgalactoside)	C28 H24 O16	Full match	No results
TRUE			Unused	No results
TRUE			No results	No results
TRUE			No results	No results
TRUE	Ditetradecyl 3,3′-thiodipropionate	C34 H66 O4	Not the top	No results
TRUE			Unused	No results
TRUE	Butyryl-L-carnitine	C11 H21 N C	Full match	No results
TRUE			Unused	No results
TRUE			No results	No results
TRUE	Denticulaflavonol	C35 H42 O6	No results	No results
TRUE			Unused	No results
TRUE			No results	No results
TRUE			Unused	No results
TRUE	N-Palmitoyl-D-erythro-sphingosine	C34 H67 N C	Full match	No results
TRUE			Unused	No results
TRUE			Unused	No results
TRUE			Unused	No results
TRUE	3,6,9,12-Tetraoxahexacosan-1-ol	C22 H46 O5	Not the top	No results
TRUE			Unused	No results
TRUE			No results	No results
TRUE			Unused	No results
TRUE			Unused	No results
TRUE			Unused	No results
TRUE			No results	No results
TRUE	Aeruginopeptin95B	C56 H79 N9	No results	No results
TRUE			Unused	No results
TRUE			Unused	No results
TRUE			No results	No results
TRUE			Unused	No results
TRUE			Unused	No results
TRUE			Unused	No results
TRUE			Unused	No results
TRUE	Aminopyralid	C6 H4 Cl2 N2	Not the top	No results
TRUE			Unused	No results
TRUE			Unused	No results

TRUE			No results	No results
TRUE			No results	No results
TRUE			No results	No results
TRUE			Unused	No results
TRUE			Unused	No results
TRUE	Mycothiol	C17 H30 N2	Not the top	No results
TRUE			No results	No results
TRUE	Docosanamide	C22 H45 N C	No results	No results
TRUE			Unused	No results
TRUE			Unused	No results
TRUE			Unused	No results
TRUE	NP-020705	C17 H24 O8	No match	Invalid mass
TRUE			Unused	No results
TRUE			Unused	No results
TRUE	Rishitin	C14 H22 O2	Full match	No results
TRUE			No results	No results
TRUE			Unused	No results
TRUE			No results	No results
TRUE			Unused	No results
TRUE	2-Methylhippuricacid	C10 H11 N C	Full match	No results
TRUE			Unused	No results
TRUE			Unused	No results
TRUE			Unused	No results
TRUE	AicarMonophosphate;5-Aminoimidazole-4-carboxam	C9 H15 N4 C	No match	No results
TRUE			Unused	No results
TRUE			Unused	No results
TRUE			Unused	No results
TRUE	Aeruginopeptin917S-B	C54 H76 N8	No match	No results
TRUE			No results	No results
TRUE			Unused	No results
TRUE			Unused	No results
TRUE			Unused	No results
TRUE			No results	No results
TRUE			Unused	No results
TRUE			Unused	No results
TRUE			Unused	No results
TRUE			Unused	No results
TRUE			Unused	No results
TRUE			Unused	No results
TRUE			Unused	No results
TRUE	6-Hydroxyluteolin 6-glucuronide	C21 H18 O1:	No match	No results
TRUE			Unused	No results
TRUE			Unused	No results
TRUE			Unused	No results
TRUE			Unused	No results
TRUE			Unused	No results
TRUE			Unused	No results
TRUE			Unused	No results
TRUE			No results	No results
TRUE			Unused	No results

TRUE	2-Amino-3-phosphonopropanoate	C3 H8 N O5 I	Full match	No results
TRUE			Unused	No results
TRUE			Unused	No results
TRUE			Invalid mass	No results
TRUE			Unused	No results
TRUE			Unused	No results
TRUE			Unused	No results
TRUE			Unused	No results
TRUE			No results	No results
TRUE	FL5FFGGS0001_a	C16 H12 O9	No results	No results
TRUE			Unused	No results
TRUE			No results	No results
TRUE			Unused	No results
TRUE			Unused	No results
TRUE	4-Hydroxyphenylacetylglutamicacid	C13 H15 N C	Not the top	No results
TRUE	2-Phenylethanolglucuronide	C14 H18 O7	Not the top	No results
TRUE			Unused	No results
TRUE			No results	No results
TRUE			Unused	No results
TRUE			Unused	No results
TRUE	Zalcitabine	C9 H13 N3 C	No results	No results
TRUE	dTDP-4-dehydro-2,3,6-trideoxy-α-D-hexopyranose	C16 H24 N2	No match	No results
TRUE			Unused	No results
TRUE			Unused	No results
TRUE			Unused	No results
TRUE	Isoorientin 2"-p-hydroxybenzoate	C28 H24 O1:	No results	No results
TRUE			Unused	No results
TRUE	Chrysin 6-C-glucoside-8-C-alpha-L-arabinopyranoside	C26 H28 O1:	No match	No results
TRUE	1-Palmitoylglycerol3-phosphate	C19 H39 O7	Not the top	No results
TRUE			Unused	No results
TRUE			Unused	No results
TRUE	[FA(18:4)]6Z_9Z_12Z_15Z-octadecatetraenoicacid	C18 H28 O2	Full match	No results
TRUE			Unused	No results
TRUE	DL-Erythrono-1_4-lactone;Erythrono-1_4-lactone	C4 H6 O4	No results	No results
TRUE	Quercetin 3- (2"-galloyl-alpha-L-arabinopyranoside)	C27 H22 O1!	No match	No results
TRUE	2,5-Dimethyl-3-hexyne-2,5-diol	C8 H14 O2	Full match	No results
TRUE			Unused	No results
TRUE			Unused	No results
TRUE	6-Acetyl-D-glucose	C8 H14 O7	Not the top	No results
TRUE			Unused	No results
TRUE	2",6"-Di-O-Acetyl isovitexin	C25 H24 O1:	No match	No results
TRUE			Unused	No results
TRUE			No results	No results
TRUE	Citrinin	C13 H14 O5	No match	No results
TRUE			Unused	No results
TRUE			Unused	No results
TRUE	Okanin 4'- (6"-acetylglucoside)	C23 H24 O1:	No match	No results
TRUE			No results	No results
TRUE	Chrysin 7-glucuronide	C21 H18 O1(	No match	No results
TRUE			Unused	No results

TRUE			No results	No results
TRUE			Unused	No results
TRUE			Unused	No results
TRUE			Unused	No results
TRUE			No results	No results
TRUE			Invalid mass	No results
TRUE			Unused	No results
TRUE	ferroxamine	C25 H45 Fe I	No match	No results
TRUE	4-Hydroxyphenylacetylglycine	C10 H11 N C	Full match	No results
TRUE			Unused	No results
TRUE			Unused	No results
TRUE	Hyperforin	C35 H52 O4	Not the top	No results
TRUE			Unused	No results
TRUE			No results	No results
TRUE			No results	No results
TRUE			Unused	No results
TRUE			Unused	No results
TRUE	Flunitrazepam	C16 H12 F N	No match	No results
TRUE			Unused	No results
TRUE			Unused	No results
TRUE			Unused	No results
TRUE			Unused	No results
TRUE			Unused	No results
TRUE			No results	No results
TRUE			Unused	No results
TRUE			Unused	No results
TRUE			Unused	No results
TRUE			Unused	No results
TRUE	Nicotianamine	C12 H21 N3	Not the top	No results
TRUE			Unused	No results
TRUE			Unused	No results
TRUE			Unused	No results
TRUE			No results	No results
TRUE	5,7,2',5'-Tetrahydroxy-6-methoxyflavanone	C16 H14 O7	Not the top	No results
TRUE	Tiotropium	C19 H21 N C	No results	No results
TRUE			Unused	No results
TRUE			No results	No results
TRUE			No results	No results
TRUE			Unused	No results
TRUE			Unused	No results
TRUE			Unused	No results
TRUE			Unused	No results
TRUE			Unused	No results
TRUE			Unused	No results
TRUE	cyclo-dopa5-O-glucoside	C15 H19 N C	No match	No results
TRUE			Unused	No results
TRUE	bumetrizole	C17 H18 Cl N	No match	No results
TRUE			Unused	No results
TRUE			Unused	No results
TRUE			Unused	No results

TRUE	Torosaflavone D	C19 H14 O8	Not the top	No results
TRUE			No results	No results
TRUE	Dihydroneopterinphosphate	C9 H14 N5 C	Not the top	No results
TRUE	5-Hydroxyxanthotoxin	C12 H8 O5	Full match	No results
TRUE			Unused	No results
TRUE			Unused	No results
TRUE			Unused	No results
TRUE			Unused	No results
TRUE	Rubone	C20 H22 O7	Not the top	No results
TRUE			Unused	No results
TRUE	2-Methyl-3-hydroxy-5-formylpyridine-4-carboxylate	C8 H7 N O4	No match	No results
TRUE			Unused	No results
TRUE			No results	No results
TRUE			Unused	No results
TRUE	Bractein	C21 H20 O1	Full match	No results
TRUE	Diacetone acrylamide	C9 H15 N O2	No results	No results
TRUE			Unused	No results
TRUE			Unused	No results
TRUE			Unused	No results
TRUE			Unused	No results
TRUE	(1-Ribosylimidazole)-4-acetate	C10 H14 N2	Not the top	No results
TRUE			Unused	No results
TRUE			Invalid mass	No results
TRUE			Unused	No results
TRUE			No results	No results
TRUE			Unused	No results
TRUE			Unused	No results
TRUE			Unused	No results
TRUE			No results	No results
TRUE			Unused	No results
TRUE			Unused	No results
TRUE	Chrysin 7- (4"-acetylglucoside)	C23 H22 O1	No match	No results
TRUE			Unused	No results
TRUE	Oxadixyl	C14 H18 N2	Full match	No results
TRUE			Unused	No results
TRUE			Unused	No results
TRUE	Isorhamnetin 3- (6"-malonylglucoside)	C25 H24 O1	No match	No results
TRUE			Unused	No results
TRUE			Unused	No results
TRUE			Unused	No results
TRUE			Unused	No results
TRUE	2-Succinylbenzoate	C11 H10 O5	Not the top	No results
TRUE			Unused	No results
TRUE	4-Nitroacetophenone	C8 H7 N O3	Full match	No results
TRUE			Unused	No results
TRUE			Unused	No results
TRUE	2-Hydroxyethyl laurate	C14 H28 O3	Full match	No results
TRUE			Unused	No results
TRUE			Unused	No results
TRUE			Unused	No results

TRUE			Unused	No results
TRUE			No results	No results
TRUE			Unused	No results
TRUE			No results	No results
TRUE			Unused	No results
TRUE	2,2'-(Tridecylimino)diethanol	C17 H37 N C	Full match	No results
TRUE			Unused	No results
TRUE			No results	No results
TRUE			No results	No results
TRUE			No results	No results
TRUE			No results	No results
TRUE			No results	No results
TRUE			No results	No results
TRUE			Unused	No results
TRUE			No results	No results
TRUE			Unused	No results
TRUE	Scutellarein 7-glucuronide-6-ferulate	C31 H26 O15	No match	No results
TRUE	(R)-3-(4-Hydroxyphenyl)lactate	C9 H10 O4	No match	No results
TRUE			Unused	No results
TRUE			No results	No results
TRUE			No results	No results
TRUE			No results	No results
TRUE	4',7-Dimethoxy-6,8-dimethyl-5-hydroxyflavone	C19 H18 O5	Not the top	No results
TRUE			Unused	No results
TRUE			Unused	No results
TRUE			Unused	No results
TRUE			Unused	No results
TRUE			Unused	No results
TRUE			Unused	No results
TRUE			Unused	No results
TRUE			No results	No results
TRUE	FL2FAAGC0001_a	C23 H18 O8	Full match	No results
TRUE			Unused	No results
TRUE			Unused	No results
TRUE	(-)-Epigallocatechin 3-gallate	C22 H18 O11	No results	No results
TRUE			Unused	No results
TRUE			Unused	No results
TRUE			Unused	No results
TRUE			No results	No results
TRUE			Unused	No results
TRUE	Silandrin	C25 H22 O9	Not the top	No results
TRUE	Chrysin 7-glucuronide	C21 H18 O10	Not the top	No results
TRUE			No results	No results
TRUE	Propoxur	C11 H15 N C	Full match	No results
TRUE			Unused	No results
TRUE			Unused	No results
TRUE			Unused	No results
TRUE			Unused	No results
TRUE			Unused	No results
TRUE	6,3',4'-Trihydroxy-4-methoxy-5-methylaurone	C17 H14 O6	Not the top	No results

TRUE	2-Carboxy-2_3-dihydro-5_6-dihydroxyindole	C9 H9 N O4	Not the top	No results
TRUE			No results	No results
TRUE			Unused	No results
TRUE			Unused	No results
TRUE			Unused	No results
TRUE			No results	No results
TRUE	Quercetin 3-isobutyrate	C19 H16 O8	Not the top	No results
TRUE			Unused	No results
TRUE			Unused	No results
TRUE			Unused	No results
TRUE			Unused	No results
TRUE			Unused	No results
TRUE			Unused	No results
TRUE			No results	No results
TRUE	4-Hydroxyphenylacetylglutamicacid	C13 H15 N C	No match	No results
TRUE	Isoschaftoside 2"'-ferulate	C36 H36 O1	Not the top	No results
TRUE	Decamethyltetrasiloxane	C10 H30 O3	No match	No results
TRUE			Unused	No results
TRUE			Unused	No results
TRUE			Unused	No results
TRUE			No results	No results
TRUE			Unused	No results
TRUE	FL2FEGGS0001_a	C20 H22 O8	Not the top	No results
TRUE			Unused	No results
TRUE			Unused	No results
TRUE			No results	No results
TRUE			Unused	No results
TRUE			Unused	No results
TRUE			No results	No results
TRUE			Unused	No results
TRUE			Unused	No results
TRUE			Unused	No results
TRUE	[STtrihydrox]3alpha_11beta_21-5alpha-trihydroxy-pı	C21 H34 O4	Not the top	No results
TRUE			No results	No results
TRUE			Unused	No results
TRUE			Unused	No results
TRUE	Dihydromacarpine	C22 H19 N C	Full match	No results
TRUE			Unused	No results
TRUE			Unused	No results
TRUE			Unused	No results
TRUE			No results	No results
TRUE			Unused	No results
TRUE			Unused	No results
TRUE			No results	No results
TRUE	N-Desmethyl Mephenytoin	C11 H12 N2	Full match	No results
TRUE			Unused	No results
TRUE			Unused	No results
TRUE			Unused	No results
TRUE			Unused	No results
TRUE			No results	No results

TRUE	delta-Guanidinovalericacid	C6 H13 N3 C	Full match	No results
TRUE			No results	No results
TRUE			Unused	No results
TRUE			No results	No results
TRUE			No results	No results
TRUE			Unused	No results
TRUE			No results	No results
TRUE			Unused	No results
TRUE			Unused	No results
TRUE			Unused	No results
TRUE			Unused	No results
TRUE			No results	No results
TRUE			Unused	No results
TRUE			Unused	No results
TRUE			Unused	No results
TRUE			Unused	No results
TRUE			Unused	No results
TRUE			Unused	No results
TRUE			Unused	No results
TRUE			Unused	No results
TRUE			No results	No results
TRUE			Unused	No results
TRUE			No results	No results
TRUE			No results	No results
TRUE			Unused	No results
TRUE			Unused	No results
TRUE	Propylnorapomorphine	C19 H21 N C	Full match	No results
TRUE			Unused	No results
TRUE	2-Hydroxy-6-oxonona-2_4-diene-1_9-dioate	C9 H10 O6	Full match	No results
TRUE			No results	No results
TRUE			Unused	No results
TRUE			No results	No results
TRUE			No results	No results
TRUE			Unused	No results
TRUE			Unused	No results
TRUE			Unused	No results
TRUE	2-Hydroxy-6-oxonona-2_4-diene-1_9-dioate	C9 H10 O6	Full match	No results
TRUE			Unused	No results
TRUE			No results	No results
TRUE	3_4-Dimethoxycinnamicacid	C11 H12 O4	Full match	No results
TRUE			Unused	No results
TRUE	2,5-Cyclohexadien-1-one, 4,4'-ethanediylidenebis[2,6	C30 H42 O2	Not the top	No results
TRUE			Unused	No results
TRUE			No results	No results
TRUE	1,3-Diethoxy-1,1,3,3-tetramethyldisiloxane	C8 H22 O3 S	No match	No results
TRUE			Unused	No results
TRUE	FL2F3GGS0001_a	C17 H16 O7	Full match	No results
TRUE			No results	No results
TRUE			Unused	No results
TRUE			Unused	No results

TRUE			Unused	No results
TRUE			Unused	No results
TRUE			No results	No results
TRUE	4-Methylene-L-glutamate	C6 H9 N O4	Full match	No results
TRUE			Unused	No results
TRUE			Unused	No results
TRUE			Unused	No results
TRUE			Unused	No results
TRUE			Unused	No results
TRUE			Unused	No results
TRUE			Unused	No results
TRUE	Pantetheine	C11 H22 N2	No results	No results
TRUE	3-Indolepropionicacid	C11 H11 N C	Full match	No results
TRUE			Unused	No results
TRUE			Invalid mass	No results
TRUE			No results	No results
TRUE	Derricin	C21 H22 O3	Not the top	No results
TRUE			Unused	No results
TRUE			No results	No results
TRUE	Apigenin 7- (6"-methylglucuronide)	C22 H20 O1	Not the top	No results
TRUE	Isoswertisin 2"-O- (2"'-methylbutyrate)	C27 H30 O1	No match	No results
TRUE			Unused	No results
TRUE			Unused	No results
TRUE			No results	No results
TRUE	Riboflavinmonophosphate	C17 H21 N4	No match	No results
TRUE	Chicoricacid	C22 H18 O1	Not the top	No results
TRUE	Nothofagin	C21 H24 O1	No match	No results
TRUE	6-Hydroxyluteolin 6-xyloside	C20 H18 O1	No match	No results
TRUE			No results	No results
TRUE			Unused	No results
TRUE			Unused	No results
TRUE	N-Acetyl-L-2-amino-6-oxopimelate	C9 H13 N O6	No match	No results
TRUE	Mesquitol-4beta-ol 3,8-dimethyl ether	C17 H18 O7	No results	No results
TRUE			No results	No results
TRUE			Unused	No results
TRUE			No results	No results
TRUE			Unused	No results
TRUE			Unused	No results
TRUE			Invalid mass	No results
TRUE			Unused	No results
TRUE			No results	No results
TRUE			Unused	No results
TRUE	Sanguinarine	C20 H14 N C	No match	No results
TRUE	5,7,2'-Trihydroxyflavone 7-glucuronide	C21 H18 O1	Full match	No results
TRUE			Unused	No results
TRUE			Unused	No results
TRUE			Unused	No results
TRUE			Unused	No results
TRUE			Unused	No results
TRUE			Unused	No results

TRUE	Tributyl Phosphate	C12 H27 O4	Full match	No results
TRUE			Unused	No results
TRUE			No results	No results
TRUE			Unused	No results
TRUE			Unused	No results
TRUE			Unused	No results
TRUE			Unused	No results
TRUE			Unused	No results
TRUE			Unused	No results
TRUE			No results	No results
TRUE			Unused	No results
TRUE			No results	No results
TRUE	2-Oxoglutaramate	C5 H7 N O4	No results	No results
TRUE			Unused	No results
TRUE			Unused	No results
TRUE			Unused	No results
TRUE			Unused	No results
TRUE	5,7,2',5'-Tetrahydroxy-6-methoxyflavanone	C16 H14 O7	Not the top	No results
TRUE			Unused	No results
TRUE			Unused	No results
TRUE	N-Benzoyl-4-hydroxyanthranilate	C14 H11 N C	Full match	No results
TRUE			Unused	No results
TRUE			Unused	No results
TRUE			No results	No results
TRUE			Unused	No results
TRUE			Unused	No results
TRUE			Unused	No results
TRUE	Benzyl(2R_3S)-2-methyl-3-hydroxybutanoate	C12 H16 O3	Full match	No results
TRUE	3,5,7,3',4',5'-Hexahydroxy-6,8-dimethylflavanone	C17 H16 O8	Full match	No results
TRUE			Unused	No results
TRUE			Unused	No results
TRUE	Ramipril	C23 H32 N2	Not the top	No results
TRUE			Unused	No results
TRUE			No results	No results
TRUE			Unused	No results
TRUE			No results	No results
TRUE			Unused	No results
TRUE	Variabiloside A	C42 H46 O2:	Full match	No results
TRUE			No results	No results
TRUE			Unused	No results
TRUE			Unused	No results
TRUE			Unused	No results
TRUE			Unused	No results
TRUE			Unused	No results
TRUE	2-(1-Hydroxycyclohexyl)-1-phenylethanone	C14 H18 O2	Full match	No results
TRUE			Unused	No results
TRUE	2,2'-ETHYLIDENEBIS(4,6-DI-TERT-BUTYLPHENOL)	C30 H46 O2	Full match	No results
TRUE			Unused	No results
TRUE			Unused	No results
TRUE	3,5,7,3',4',5'-Hexahydroxy-6,8-dimethylflavanone	C17 H16 O8	Not the top	No results

TRUE			Unused	No results
TRUE			Unused	No results
TRUE			Unused	No results
TRUE	(1R_6R)-6-Hydroxy-2-succinylcyclohexa-2_4-diene-1-	C11 H12 O6	Not the top	No results
TRUE			Unused	No results
TRUE			No results	No results
TRUE			No results	No results
TRUE			Unused	No results
TRUE			Unused	No results
TRUE			Unused	No results
TRUE	Patulin	C7 H6 O4	Full match	No results
TRUE			Unused	No results
TRUE			Unused	No results
TRUE			Unused	No results
TRUE			Unused	No results
TRUE	2-Ethoxy-2-oxoethyl ethyl phthalate	C14 H16 O6	Not the top	No results
TRUE			Unused	No results
TRUE			Unused	No results
TRUE			Unused	No results
TRUE			Unused	No results
TRUE			No results	No results
TRUE			No results	No results
TRUE			Unused	No results
TRUE			Unused	No results
TRUE			Unused	No results
TRUE			Unused	No results
TRUE			No results	No results
TRUE	Anhydroicaritin 3,7-diglucoside	C33 H40 O1(	Not the top	No results
TRUE			Unused	No results
TRUE			Unused	No results
TRUE			No results	No results
TRUE			Unused	No results
TRUE			Unused	No results
TRUE			Unused	No results
TRUE			Unused	No results
TRUE	UNII:DUA9665YBG	C13 H18 O2	No results	No results
TRUE			Unused	No results
TRUE			Unused	No results
TRUE			Unused	No results
TRUE			No results	No results
TRUE			Unused	No results
TRUE			No results	No results
TRUE			No results	No results
TRUE			Unused	No results
TRUE	Hyperforin	C35 H52 O4	Not the top	No results
TRUE			Unused	No results
TRUE			Unused	No results
TRUE			Unused	No results
TRUE			Unused	No results
TRUE			No results	No results

TRUE	[FA(16:2)]N-hexadecyl-ethanolamine	C18 H37 N C	Full match	No results
TRUE	8-Oxocoformycin	C11 H14 N4	Not the top	No results
TRUE	5,7,4'-Trihydroxyflavanone 7-sulfate	C15 H12 O8	Full match	No results
TRUE			Unused	No results
TRUE	3_5-Dihydroxy-phenylglycine	C8 H9 N O4	Full match	No results
TRUE			Unused	No results
TRUE			Unused	No results
TRUE	Phenylthiotrimethylsilane	C9 H14 S Si	No results	No results
TRUE			Unused	No results
TRUE	14-Oxolanosterol	C30 H48 O2	Not the top	No results
TRUE			Unused	No results
TRUE			Unused	No results
TRUE			Unused	No results
TRUE	3-[(1-Carboxyvinyl)oxy]benzoate	C10 H8 O5	No match	No results
TRUE			No results	No results
TRUE			No results	No results
TRUE			No results	No results
TRUE			Unused	No results
TRUE	Hydroxyversicolorone	C20 H16 O8	Full match	No results
TRUE			No results	No results
TRUE	5-Hydroxy-6-methoxyindoleglucuronide	C15 H17 N C	No results	No results
TRUE			Unused	No results
TRUE			Unused	No results
TRUE			Unused	No results
TRUE			Unused	No results
TRUE	2"-O-Vanilloylvitexin	C29 H26 O13	No match	No results
TRUE			Unused	No results
TRUE			Unused	No results
TRUE			Unused	No results
TRUE			Unused	No results
TRUE	5,3',4'-Trihydroxy-3-methoxy-6,7-methylenedioxyflav	C17 H12 O8	Full match	No results
TRUE			Unused	No results
TRUE			Unused	No results
TRUE	dTDP-4-dehydro-2,3,6-trideoxy-α-D-hexopyranose	C16 H24 N2	No match	No results
TRUE			Unused	No results
TRUE			Unused	No results
TRUE			Unused	No results
TRUE			Unused	No results
TRUE			Unused	No results
TRUE			Unused	No results
TRUE			Invalid mass	No results
TRUE	Betalamicacid	C9 H9 N O5	No match	No results
TRUE	L-Kynurenine	C10 H12 N2	Full match	No results
TRUE			Unused	No results
TRUE	Flucarbazone	C12 H11 F3 I	No match	No results
TRUE			Unused	No results
TRUE			No results	No results
TRUE	2,5-Dimethyl-3-hexyne-2,5-diol	C8 H14 O2	Full match	No results
TRUE			Unused	No results
TRUE			No results	No results

TRUE			Unused	No results
TRUE			Unused	No results
TRUE			Unused	No results
TRUE	Linderoflavone A	C18 H14 O8	Full match	No results
TRUE	Isoorientin 6"-O-alpha-L-arabinoside	C25 H26 O15	No match	No results
TRUE			No results	No results
TRUE			Unused	No results
TRUE			Unused	No results
TRUE			Unused	No results
TRUE	Nicotianamine	C12 H21 N3	Full match	No results
TRUE	[FA(18:1)]9Z-Octadecen-12-ynoicacid	C18 H30 O2	Not the top	No results
TRUE	[FA(16:2)]N-hexadecyl-ethanolamine	C18 H37 N C	Full match	No results
TRUE			Unused	No results
TRUE	Penicillic-Acid	C8 H10 O4	No results	No results
TRUE			Unused	No results
TRUE			Unused	No results
TRUE			Unused	No results
TRUE			Unused	No results
TRUE			Unused	No results
TRUE			Unused	No results
TRUE			Unused	No results
TRUE			No results	No results
TRUE			Unused	No results
TRUE			Unused	No results
TRUE	Telephioidin	C21 H20 O13	No match	No results
TRUE			Unused	No results
TRUE			Unused	No results
TRUE			Unused	No results
TRUE			Unused	No results
TRUE			Unused	No results
TRUE	2,2'-ETHYLIDENEBIS(4,6-DI-TERT-BUTYLPHENOL)	C30 H46 O2	Full match	No results
TRUE			Unused	No results
TRUE			Unused	No results
TRUE			Unused	No results
TRUE			Unused	No results
TRUE			Unused	No results
TRUE	3_6-Nonadienal	C9 H14 O	Full match	No results
TRUE			No results	No results
TRUE			Unused	No results
TRUE			Unused	No results
TRUE			Unused	No results
TRUE	Aromadendrin 3-beta-L-arabinopyranoside	C20 H20 O10	Not the top	No results
TRUE	Bis[4-amino-1-anthraquinonyl]amine	C28 H17 N3	Full match	No results
TRUE	Isoliquiritigenin 4,4'-diglucoside	C27 H32 O14	Full match	No results
TRUE			Unused	No results
TRUE			Unused	No results
TRUE	Patulin	C7 H6 O4	Full match	No results
TRUE			No results	No results
TRUE	2"-trans-Caffeoylisoorientin	C30 H26 O14	No match	No results
TRUE	Zimelidine	C16 H17 Br N	No match	No results

TRUE			No results	No results
TRUE			No results	No results
TRUE	Quercetin 3- (2"-p-hydroxybenzoyl-4"-p-coumarylrha	C37 H30 O15	No match	No results
TRUE	Aspartyl-Phenylalanine	C13 H16 N2	Not the top	No results
TRUE			Unused	No results
TRUE			No results	No results
TRUE			Unused	No results
TRUE			No results	No results
TRUE			Unused	No results
TRUE	Epicatechin 3,5-di-O-gallate	C29 H22 O14	Not the top	No results
TRUE			No results	No results
TRUE			Unused	No results
TRUE	3,4,2',3',4',6',alpha-Heptahydroxychalcone 2'-glucosi	C21 H22 O13	No results	No results
TRUE			Unused	No results
TRUE	Scoparone	C11 H10 O4	Full match	No results
TRUE			Unused	No results
TRUE	Telephioidin	C21 H20 O13	Not the top	No results
TRUE			Unused	No results
TRUE			Unused	No results
TRUE			Unused	No results
TRUE			Unused	No results
TRUE			Unused	No results
TRUE	2-Propenoic acid, 2-phenoxyethyl ester	C11 H12 O3	Full match	No results
TRUE			Unused	No results
TRUE			Unused	No results
TRUE			Unused	No results
TRUE			Unused	No results
TRUE			Unused	No results
TRUE			Unused	No results
TRUE			Unused	No results
TRUE			Unused	No results
TRUE			Unused	No results
TRUE			No results	No results
TRUE			Unused	No results
TRUE	FL2F3GGS0001_a	C17 H16 O7	Not the top	No results
TRUE			Unused	No results
TRUE			Unused	No results
TRUE			Unused	No results
TRUE			Unused	No results
TRUE			Unused	No results
TRUE			Unused	No results
TRUE			Unused	No results
TRUE	Glandicoline B	C22 H21 N5	Not the top	No results
TRUE			No results	No results
TRUE	Dhurrin	C14 H17 N C	Not the top	No results
TRUE	1,3-Bis(3-(2,3-epoxypropoxy)propyl)tetramethyldisilo	C16 H34 O5	No match	No results
TRUE	11-Oxo-androsteroneglucuronide	C25 H36 O9	No match	No results
TRUE			Unused	No results
TRUE			Unused	No results
TRUE			Unused	No results

TRUE			Unused	No results
TRUE			Unused	No results
TRUE			No results	No results
TRUE			Unused	No results
TRUE			Unused	No results
TRUE			Unused	No results
TRUE	5-Hydroxyferulate	C10 H10 O5	No match	No results
TRUE			Unused	No results
TRUE	Phyllospadine	C21 H21 N C	No match	No results
TRUE			Unused	No results
TRUE			No results	No results
TRUE			Unused	No results
TRUE			Unused	No results
TRUE	3,6-Dimethoxy-6",6"-dimethyl-3',4'-methylenedioxyp	C23 H20 O7	Not the top	No results
TRUE			Unused	No results
TRUE			Unused	No results
TRUE	7,9-di-tert-butyl-1-oxaspiro[4.5]deca-6,9-diene-2,8-d	C17 H24 O3	Full match	No results
TRUE	L-Formylkynurenine	C11 H12 N2	Full match	No results
TRUE			Unused	No results
TRUE			Unused	No results
TRUE			Unused	No results
TRUE			No results	No results
TRUE	6-Hydroxyluteolin 7- (6"- (E) -caffeoylglucoside)	C30 H26 O15	Not the top	No results
TRUE			No results	No results
TRUE	Agamanone	C18 H16 O8	Not the top	No results
TRUE			Unused	No results
TRUE			Unused	No results
TRUE			Unused	No results
TRUE			No results	No results
TRUE	Methylophiopogonanone A	C19 H18 O6	Full match	No results
TRUE			Unused	No results
TRUE			No results	No results
TRUE			No results	No results
TRUE			Unused	No results
TRUE			No results	No results
TRUE	Patulin	C7 H6 O4	Full match	No results
TRUE			Unused	No results
TRUE			Unused	No results
TRUE			Unused	No results
TRUE			Unused	No results
TRUE			Unused	No results
TRUE			Unused	No results
TRUE	Unsaturated archaetidylserine	C46 H78 N C	No match	No results
TRUE			Unused	No results
TRUE			Unused	No results
TRUE			Invalid mass	No results
TRUE			Unused	No results
TRUE			Unused	No results
TRUE			Unused	No results
TRUE	(-)-beta-Pinene	C10 H16	Full match	No results

TRUE			No results	No results
TRUE			No results	No results
TRUE			Unused	No results
TRUE	Quercetin 3- (2"-galloyl-alpha-L-arabinopyranoside)	C27 H22 O15	Full match	No results
TRUE			Unused	No results
TRUE			Unused	No results
TRUE			No results	No results
TRUE			Unused	No results
TRUE	Flurochloridone	C12 H10 Cl2	No match	No results
TRUE			Unused	No results
TRUE			No results	No results
TRUE			No results	No results
TRUE			No results	No results
TRUE			Unused	No results
TRUE			Unused	No results
TRUE			Unused	No results
TRUE			Unused	No results
TRUE	WithanolideD	C28 H38 O6	Not the top	No results
TRUE	Convallatoxin	C29 H42 O10	No match	No results
TRUE	7_8-Dihydroxycoumarin	C9 H6 O4	No results	No results
TRUE			No results	No results
TRUE	UNII:TYL476W27Y	C18 H30 O	Full match	No results
TRUE	Silybin	C25 H22 O10	Full match	No results
TRUE	Flaccidine	C23 H22 O9	No match	No results
TRUE			Unused	No results
TRUE			Unused	No results
TRUE			Unused	No results
TRUE			Unused	No results
TRUE	8'-apo-beta-Carotenol	C30 H42 O	Full match	No results
TRUE			Unused	No results
TRUE			Unused	No results
TRUE			No results	No results
TRUE	Indol-3-ylacetyl-myo-inositolL-arabinoside	C21 H27 N C	No match	No results
TRUE			Unused	No results
TRUE	4-Hydroxy-6-methylpretetramide	C20 H15 N C	No match	No results
TRUE			No results	No results
TRUE			Unused	No results
TRUE			Unused	No results
TRUE	3-Methyl-1-(2_4_6-trihydroxyphenyl)butan-1-one	C11 H14 O4	Full match	No results
TRUE	Butyryl-L-carnitine	C11 H21 N C	Full match	No results
TRUE			Unused	No results
TRUE	2,5-Dihydroxybenzoate 2-O-β-D-glucoside	C13 H16 O9	Not the top	No results
TRUE	Aminocarb	C11 H16 N2	No results	No results
TRUE			Unused	No results
TRUE			Unused	No results
TRUE			Unused	No results
TRUE			Unused	No results
TRUE			No results	No results
TRUE			Unused	No results
TRUE			Unused	No results

TRUE			Unused	No results
TRUE			No results	No results
TRUE			Unused	No results
TRUE	Anadanthoside	C20 H22 O9	No match	No results
TRUE	3alpha-Hydroxyglycyrrhetinate	C30 H46 O4	Not the top	No results
TRUE			Unused	No results
TRUE			Unused	No results
TRUE			No results	No results
TRUE	trans-Cinnamoylbeta-D-glucoside	C15 H18 O7	Not the top	No results
TRUE	Undecaprenyldiphosphate	C55 H92 O7	No match	No results
TRUE			Unused	No results
TRUE			Unused	No results
TRUE			Unused	No results
TRUE			Unused	No results
TRUE			Unused	No results
TRUE	Trichlorfon	C4 H8 Cl3 O4	No match	No results
TRUE	L-gamma-Glutamyl-L-hypoglycin	C12 H18 N2	Not the top	No results
TRUE			No results	No results
TRUE			No results	No results
TRUE			Unused	No results
TRUE			Unused	No results
TRUE			Unused	No results
TRUE	Chenodeoxyglycocholate	C26 H43 N C	Not the top	No results
TRUE			No results	No results
TRUE			Unused	No results
TRUE			Unused	No results
TRUE			No results	No results
TRUE			Unused	No results
TRUE			Unused	No results
TRUE			Unused	No results
TRUE	2_3-Dihydro-2_3-dihydroxybenzoate	C7 H8 O4	Full match	No results
TRUE			Unused	No results
TRUE			No results	No results
TRUE			Unused	No results
TRUE	6,3'-Dihydroxy-4,4'-dimethoxy-5-methylaurone	C18 H16 O6	Not the top	No results
TRUE			Unused	No results
TRUE			Unused	No results
TRUE			Unused	No results
TRUE			Unused	No results
TRUE			Unused	No results
TRUE			Unused	No results
TRUE			Unused	No results
TRUE			No results	No results
TRUE			Unused	No results
TRUE			No results	No results
TRUE			No results	No results
TRUE			Unused	No results
TRUE			Unused	No results
TRUE			Unused	No results
TRUE			No results	No results

TRUE			Unused	No results
TRUE			Unused	No results
TRUE			Unused	No results
TRUE			Unused	No results
TRUE	Lupinisoflavone N	C25 H28 O9	Full match	No results
TRUE	Formononetin 7-O- (6"-acetylglucoside)	C24 H24 O1(	Not the top	No results
TRUE			No results	No results
TRUE	CMP-3-deoxy-D-glycero-D-galacto-non-2-ulopyranos	C18 H28 N3	Not the top	No results
TRUE			Unused	No results
TRUE	Pongamoside A	C23 H20 O9	Not the top	No results
TRUE	Silybin	C25 H22 O1(	No results	No results
TRUE			Unused	No results
TRUE	FL2F3GGS0001_a	C17 H16 O7	Not the top	No results
TRUE			Unused	No results
TRUE			No results	No results
TRUE	Xanthosine	C10 H12 N4	No match	No results
TRUE			Unused	No results
TRUE			No results	No results
TRUE			Unused	No results
TRUE			No results	No results
TRUE			Unused	No results
TRUE			Unused	No results
TRUE			Unused	No results
TRUE			Unused	No results
TRUE			Unused	No results
TRUE			No results	No results
TRUE	1,1'-[Oxybis(2,1-ethanediyloxy-2,1-ethanediyl)]bis(1H	C16 H20 N2	No results	No results
TRUE			Unused	No results
TRUE			Unused	No results
TRUE			Unused	No results
TRUE			No results	No results
TRUE			Unused	No results
TRUE			Unused	No results
TRUE			No results	No results
TRUE			No results	No results
TRUE			Unused	No results
TRUE			No results	No results
TRUE	FL5FAGGN0001_a	C24 H18 O1:	No match	No results
TRUE			No results	No results
TRUE	Actinorhodine	C32 H26 O14	Not the top	No results
TRUE			Unused	No results
TRUE			Unused	No results
TRUE			No results	No results
TRUE			Unused	No results
TRUE			Unused	No results
TRUE			No results	No results
TRUE	2-(alpha-D-Galactosyl)-sn-glycerol3-phosphate	C9 H19 O11	No match	No results
TRUE	Hexestrol	C18 H22 O2	No results	No results
TRUE			Unused	No results
TRUE			No results	No results

TRUE	Apigenin 7- (2"-glucosyllactate)	C24 H24 O12	No match	No results
TRUE			Unused	No results
TRUE			Unused	No results
TRUE			No results	No results
TRUE			Unused	No results
TRUE			No results	No results
TRUE			Unused	No results
TRUE			Unused	No results
TRUE			No results	No results
TRUE			Unused	No results
TRUE			Unused	No results
TRUE			Unused	No results
TRUE			Unused	No results
TRUE			Unused	No results
TRUE			Unused	No results
TRUE			Unused	No results
TRUE	Miglitol	C8 H17 N O5	Full match	No results
TRUE			No results	No results
TRUE			Unused	No results
TRUE			Unused	No results
TRUE			Unused	No results
TRUE	8'-apo-beta-Carotenol	C30 H42 O	Full match	No results
TRUE	Lupinisoflavone N	C25 H28 O9	Not the top	No results
TRUE			Unused	No results
TRUE			Unused	No results
TRUE			No results	No results
TRUE			Unused	No results
TRUE			No results	No results
TRUE			No results	No results
TRUE	6,3',4'-Trihydroxy-4-methoxy-5-methylaurone	C17 H14 O6	Not the top	No results
TRUE			Unused	No results
TRUE			Unused	No results
TRUE	Propyphenazone	C14 H18 N2	No results	No results
TRUE			Unused	No results
TRUE			Unused	No results
TRUE			Unused	No results
TRUE			Unused	No results
TRUE			Unused	No results
TRUE	4α-Formyl-stigmasta-7,24(241)-dien-3β-ol	C30 H48 O2	Full match	No results
TRUE			No results	No results
TRUE			Unused	No results
TRUE			Unused	No results
TRUE			Unused	No results
TRUE			Unused	No results
TRUE			No results	No results
TRUE			Unused	No results
TRUE			No results	No results
TRUE			No results	No results
TRUE			Unused	No results
TRUE			Unused	No results

TRUE			Unused	No results
TRUE			Unused	No results
TRUE			Unused	No results
TRUE			Unused	No results
TRUE	3',5,5'-Trihydroxy-4',6,7,8-tetramethoxyflavone	C19 H18 O9	Not the top	No results
TRUE	Anhydroicaritin 3,7-diglucoside	C33 H40 O16	Full match	No results
TRUE			Unused	No results
TRUE			No results	No results
TRUE	Glycocholicacid	C26 H43 N C	Not the top	No results
TRUE			Unused	No results
TRUE			No results	No results
TRUE	FL2F3GGS0001_a	C17 H16 O7	Not the top	No results
TRUE			Unused	No results
TRUE			Unused	No results
TRUE			Unused	No results
TRUE			Unused	No results
TRUE			Unused	No results
TRUE			Unused	No results
TRUE			No results	No results
TRUE			No results	No results
TRUE	6a,12b-Dihydro-3,10,11,12-tetrahydroxy-6- (3,4,5-tri	C22 H16 O10	No match	No results
TRUE			Unused	No results
TRUE			Unused	No results
TRUE	2,5,8,11,14,17,20,23-Octaoxapentacosan-25-ol	C17 H36 O9	Not the top	No results
TRUE			Unused	No results
TRUE	6",6"-Dimethylpyrano [2",3":7,8] kaempferol 4'-met	C27 H28 O10	Full match	No results
TRUE			Unused	No results
TRUE	L-Histidine	C6 H9 N3 O2	No results	No results
TRUE	Hispidulin 7-glucuronide	C22 H20 O12	Not the top	No results
TRUE			No results	No results
TRUE			Unused	No results
TRUE			Unused	No results
TRUE			Unused	No results
TRUE			Unused	No results
TRUE			Unused	No results
TRUE			Unused	No results
TRUE			Unused	No results
TRUE			Unused	No results
TRUE			Unused	No results
TRUE			Unused	No results
TRUE			Unused	No results
TRUE			No results	No results
TRUE			Unused	No results
TRUE			Unused	No results
TRUE			Unused	No results
TRUE			Unused	No results
TRUE			Unused	No results
TRUE			No results	No results
TRUE			Unused	No results
TRUE	methyl azelate	C11 H20 O4	No results	No results

TRUE	Deoxyuridine	C9 H12 N2 C	No match	No results
TRUE			Unused	No results
TRUE			Unused	No results
TRUE			Unused	No results
TRUE			Unused	No results
TRUE	Phthalic Acid, Bis-N-Pentyl Ester	C18 H26 O4	Full match	No results
TRUE			Unused	No results
TRUE	2-Heptyl-4-hydroxyquinoline-N-oxide	C16 H21 N C	Full match	No results
TRUE	gamma-Tocotrienol	C28 H42 O2	No results	No results
TRUE			No results	No results
TRUE			Unused	No results
TRUE			Unused	No results
TRUE			Unused	No results
TRUE			Unused	No results
TRUE			Unused	No results
TRUE			No results	No results
TRUE			Unused	No results
TRUE	Scopolin	C16 H20 O9	Not the top	No results
TRUE	7-Hydroxy-5,6-dimethoxyflavone 7-glucoside	C23 H24 O1(	No match	No results
TRUE			Unused	No results
TRUE			Unused	No results
TRUE	Brosimacutin C	C20 H22 O5	No match	No results
TRUE			Unused	No results
TRUE	a-Hydroxycyclohexylphenylketone	C13 H16 O2	Full match	No results
TRUE			Unused	No results
TRUE			Unused	No results
TRUE			Unused	No results
TRUE			Unused	No results
TRUE			Unused	No results
TRUE			No results	No results
TRUE			No results	No results
TRUE	Voriconazole	C16 H14 F3 I	No match	No results
TRUE			Unused	No results
TRUE			Invalid mass	No results
TRUE	N-(4-Guanidinobutyl)-4-hydroxycinnamide	C14 H20 N4	Not the top	No results
TRUE			Unused	No results
TRUE			Unused	No results
TRUE	Patulin	C7 H6 O4	No results	No results
TRUE			No results	No results
TRUE			Unused	No results
TRUE			Unused	No results
TRUE			No results	No results
TRUE			No results	No results
TRUE			Unused	No results
TRUE			Unused	No results
TRUE	Chrysin 7-glucuronide	C21 H18 O1(	Not the top	No results
TRUE			Unused	No results
TRUE			Unused	No results
TRUE	3-Hydroxy-2-methylpyridine-4_5-dicarboxylate	C8 H7 N O5	Full match	No results
TRUE			Unused	No results

TRUE			Unused	No results
TRUE	Torosaflavone A	C21 H20 O8	Full match	No results
TRUE			Unused	No results
TRUE			Unused	No results
TRUE	BaccatinIII	C31 H38 O1	Full match	No results
TRUE			Unused	No results
TRUE	Kaempferol 3- (4"-acetyl-6"-p-coumarylglucoside)	C32 H28 O1	Not the top	No results
TRUE			Unused	No results
TRUE			Unused	No results
TRUE			Unused	No results
TRUE			Unused	No results
TRUE			No results	No results
TRUE			Unused	No results
TRUE			Unused	No results
TRUE	bumetrizole	C17 H18 Cl N	No match	No results
TRUE			Invalid mass	No results
TRUE			No results	No results
TRUE			No results	No results
TRUE	4,2'-Dihydroxy-4',6'-dimethoxychalcone 4-O- (5"'-O-p	C37 H40 O1	No match	No results
TRUE			Unused	No results
TRUE			Unused	No results
TRUE	Aeruginopeptin917S-B	C54 H76 N8	No match	No results
TRUE			Unused	No results
TRUE	Epigallocatechin 3,5,-di-O-gallate	C29 H22 O1	Not the top	No results
TRUE	3,6-Dimethoxy-6",6"-dimethyl-3',4'-methylenedioxyp	C23 H20 O7	Not the top	No results
TRUE	Dihydrophaseicacid	C15 H22 O5	Not the top	No results
TRUE			Unused	No results
TRUE	2"-O-Galloylisovitexin	C28 H24 O1	Not the top	No results
TRUE			Unused	No results
TRUE	2-Oxooctadecanoicacid	C18 H34 O3	Full match	No results
TRUE			Unused	No results
TRUE			Unused	No results
TRUE			Unused	No results
TRUE			Unused	No results
TRUE			Unused	No results
TRUE			No results	No results
TRUE			No results	No results
TRUE			No results	No results
TRUE			Unused	No results
TRUE	Vitexin 2"-p-hydroxybenzoate	C28 H24 O1	No match	No results
TRUE			No results	No results
TRUE			No results	No results
TRUE	3-Methyldioxyindole	C9 H9 N O2	Full match	No results
TRUE			Unused	No results
TRUE			Unused	No results
TRUE			Unused	No results
TRUE			Unused	No results
TRUE	Maritimetin 6- (6"-p-coumarylglucoside)	C30 H26 O1	Not the top	No results
TRUE			Unused	No results
TRUE			Unused	No results

TRUE			Unused	No results
TRUE	(6Z)-Octadecenoicacid	C18 H34 O2	Full match	No results
TRUE	Pongapinone A	C23 H22 O7	No match	No results
TRUE			Unused	No results
TRUE			No results	No results
TRUE	Taxifolin 3-apioside	C20 H20 O11	No match	No results
TRUE			No results	No results
TRUE			No results	No results
TRUE			No results	No results
TRUE			Unused	No results
TRUE	Vitexin 2"-O-p-coumarate	C30 H26 O12	No results	No results
TRUE			No results	No results
TRUE	1,3-Bis(3-(2,3-epoxypropoxy)propyl)tetramethyldisilo	C16 H34 O5	No match	No results
TRUE			Unused	No results
TRUE	6a,13a-Didehydrodolineone	C19 H10 O6	No match	No results
TRUE			Unused	No results
TRUE			Unused	No results
TRUE			No results	No results
TRUE	Hispidol 6-glucoside	C21 H20 O9	Not the top	No results
TRUE	Dhurrin	C14 H17 N O	Not the top	No results
TRUE			Unused	No results
TRUE			Unused	No results
TRUE			No results	No results
TRUE	3,4-Dihydroxyphenylethanol	C8 H10 O3	No results	No results
TRUE			Unused	No results
TRUE			No results	No results
TRUE			No results	No results
TRUE	2',4',3,4,alpha-Pentahydroxydihydrochalcone 3'-C-xy	C20 H22 O10	No match	No results
TRUE			Unused	No results
TRUE	(-) -Semiglabrin	C23 H20 O6	Full match	No results
TRUE			Unused	No results
TRUE			Unused	No results
TRUE			Unused	No results
TRUE	Picaridin	C12 H23 N O	Full match	No results
TRUE			Unused	No results
TRUE			No results	No results
TRUE	8'-apo-beta-Carotenol	C30 H42 O	Full match	No results
TRUE			Unused	No results
TRUE			Unused	No results
TRUE	Dimethyl diselenide	C2 H6 Se2	No results	No results
TRUE			Unused	No results
TRUE	2,2'-ETHYLIDENEBIS(4,6-DI-TERT-BUTYLPHENOL)	C30 H46 O2	Full match	No results
TRUE			No results	No results
TRUE			Unused	No results
TRUE			Unused	No results
TRUE	2'_4'-Dihydroxyacetophenone	C8 H8 O3	Full match	No results
TRUE			Unused	No results
TRUE			Unused	No results
TRUE			Unused	No results
TRUE			Unused	No results

TRUE			Unused	No results
TRUE			Unused	No results
TRUE			Unused	No results
TRUE			Unused	No results
TRUE			Unused	No results
TRUE			Unused	No results
TRUE			Unused	No results
TRUE			Unused	No results
TRUE			Unused	No results
TRUE			Unused	No results
TRUE			No results	No results
TRUE			No results	No results
TRUE			Unused	No results
TRUE	Dodecylaldehyde	C12 H24 O	Full match	No results
TRUE			No results	No results
TRUE			Unused	No results
TRUE			Unused	No results
TRUE			Unused	No results
TRUE	3_5-Dimethoxy-4-hydroxycinnamicacid;Sinapicacid	C11 H12 O5	Not the top	No results
TRUE			Unused	No results
TRUE	1,6-Dioxacyclododecane-7,12-dione	C10 H16 O4	Full match	No results
TRUE			Unused	No results
TRUE			Unused	No results
TRUE			Unused	No results
TRUE			No results	No results
TRUE			Unused	No results
TRUE			Unused	No results
TRUE			Unused	No results
TRUE			Unused	No results
TRUE			Unused	No results
TRUE			Unused	No results
TRUE			Unused	No results
TRUE	Flaccidine	C23 H22 O9	Full match	No results
TRUE	Poriolide	C29 H26 O1	Not the top	No results
TRUE	ReducedFMN	C17 H23 N4	No match	No results
TRUE			No results	No results
TRUE			Unused	No results
TRUE			Unused	No results
TRUE			Unused	No results
TRUE	6",6"-Dimethylpyrano [2",3":7,8] kaempferol 4'-met	C27 H28 O1(	Not the top	No results
TRUE			No results	No results
TRUE			Unused	No results
TRUE	Tributyl Phosphate	C12 H27 O4	Full match	No results
TRUE	Gossypol	C30 H30 O8	Not the top	No results
TRUE			Unused	No results
TRUE			Unused	No results
TRUE			Unused	No results
TRUE			No results	No results
TRUE			Unused	No results
TRUE	Chlorpheniramine	C16 H19 Cl N	No match	No results

TRUE			Unused	No results
TRUE			Unused	No results
TRUE	Isoprothiolane	C12 H18 O4	No match	No results
TRUE			No results	No results
TRUE			Unused	No results
TRUE			Unused	No results
TRUE			Unused	No results
TRUE			Unused	No results
TRUE	3-Devinyl-3-(1-hydroxyethyl)-chlorophyllide a	C35 H36 Mg	No match	No results
TRUE			Unused	No results
TRUE			Unused	No results
TRUE			Unused	No results
TRUE			No results	No results
TRUE			Unused	No results
TRUE			Unused	No results
TRUE			Unused	No results
TRUE			Unused	No results
TRUE			No results	No results
TRUE			No results	No results
TRUE	Tetrazepam	C16 H17 Cl N	No match	No results
TRUE	6,8-Dimethoxy-2,3-trans-2- (4-hydroxy-2,3-dimethox	C29 H28 O12	No results	No results
TRUE			No results	No results
TRUE			Unused	No results
TRUE			No results	No results
TRUE			Unused	No results
TRUE			Unused	No results
TRUE			Unused	No results
TRUE			Unused	No results
TRUE			No results	No results
TRUE			Unused	No results
TRUE			Unused	No results
TRUE			Unused	No results
TRUE			No results	No results
TRUE			Unused	No results
TRUE			Unused	No results
TRUE			Unused	No results
TRUE			Unused	No results
TRUE	cyclo-dopa5-O-glucoside	C15 H19 N C	No results	No results
TRUE			Unused	No results
TRUE			No results	No results
TRUE			Unused	No results
TRUE			Unused	No results
TRUE			Unused	No results
TRUE			Unused	No results
TRUE			Unused	No results
TRUE			Unused	No results
TRUE			Unused	No results
TRUE			Unused	No results
TRUE			Unused	No results
TRUE	(9S_10S)-9_10-Dihydroxyoctadecanoate	C18 H36 O4	Full match	No results

TRUE	3-Vinyl-7-oxabicyclo[4.1.0]heptane	C8 H12 O	Full match	No results
TRUE			Unused	No results
TRUE	Citicoline;cytidine5'diphosphocholine	C14 H26 N4	No match	No results
TRUE			Unused	No results
TRUE			No results	No results
TRUE	(25S)-5β-spirostan-3β-ol3-O-β-D-glu	C33 H54 O8	Not the top	No results
TRUE			Unused	No results
TRUE	Nothofagin	C21 H24 O1(	No results	No results
TRUE			Unused	No results
TRUE			Unused	No results
TRUE	6beta-OH-Budesonide	C25 H34 O7	Not the top	No results
TRUE			Unused	No results
TRUE			Unused	No results
TRUE			No results	No results
TRUE			Unused	No results
TRUE			Unused	No results
TRUE			Unused	No results
TRUE	(R)-Malate	C4 H6 O5	No results	No results
TRUE			Unused	No results
TRUE	Hypnum acid	C22 H16 O9	Not the top	No results
TRUE	3-Acetyl-3-devinyl-chlorophyllide a	C35 H34 Mg	No match	No results
TRUE			Unused	No results
TRUE	Clotiazepam	C16 H15 Cl N	No match	No results
TRUE			Unused	No results
TRUE			Unused	No results
TRUE			No results	No results
TRUE			Unused	No results
TRUE	Alpha-Tetrasaccharide	C26 H45 N C	Full match	No results
TRUE			Unused	No results
TRUE			Unused	No results
TRUE	[FA(18:3)]13S-hydroperoxy-9Z_11E_14Z-octadecatrie	C18 H30 O4	Not the top	No results
TRUE			Unused	No results
TRUE			Unused	No results
TRUE	Isophthalic acid	C8 H6 O4	No results	No results
TRUE			No results	No results
TRUE			Unused	No results
TRUE			Unused	No results
TRUE			No results	No results
TRUE			Unused	No results
TRUE	Mg-protoporphyrin	C34 H32 Mg	No match	No results
TRUE	Carbetamide	C12 H16 N2	Not the top	No results
TRUE			Unused	No results
TRUE			Unused	No results
TRUE			Unused	No results
TRUE	2-Hydroxy-4'-(2-hydroxyethoxy)-2-methylpropiopher	C12 H16 O4	Full match	No results
TRUE			Unused	No results
TRUE			Unused	No results
TRUE			Unused	No results
TRUE			No results	No results
TRUE	Quercetin 3-isobutyrate	C19 H16 O8	Full match	No results

TRUE	Quercetin 3-isobutyrate	C19 H16 O8	Full match	No results
TRUE	Isovitexin 7-O- (6"'-caffeoyl) -beta-D-glucopyranoside	C36 H36 O18	No match	No results
TRUE			Unused	No results
TRUE	Chlorpheniramine	C16 H19 Cl N	No match	No results
TRUE			Unused	No results
TRUE			Unused	No results
TRUE			No results	No results
TRUE	FL2F3GGS0001_a	C17 H16 O7	Full match	No results
TRUE			No results	No results
TRUE	5,7,3',6'-Tetrahydroxy-6,8,2'-trimethoxyflavone	C18 H16 O9	No match	No results
TRUE			Unused	No results
TRUE	2"-O-Vanilloylvitexin	C29 H26 O13	No match	No results
TRUE			Unused	No results
TRUE			Unused	No results
TRUE			Unused	No results
TRUE			Unused	No results
TRUE	Terbutaline	C12 H19 N C	Full match	No results
TRUE			Unused	No results
TRUE			No results	No results
TRUE	1,2-Ethanediylbis(oxy-2,1-ethanediyl) bis{3-[4-hydrox	C34 H50 O8	Not the top	No results
TRUE			Unused	No results
TRUE			Unused	No results
TRUE	5-Methoxyindoleacetate	C11 H11 N C	Full match	No results
TRUE			Unused	No results
TRUE			Unused	No results
TRUE			Unused	No results
TRUE			No results	No results
TRUE			Unused	No results
TRUE			No results	No results
TRUE			Unused	No results
TRUE			No results	No results
TRUE			Unused	No results
TRUE	Apigenin 8-C- (6"-acetylgalactoside)	C23 H22 O11	No match	No results
TRUE			Unused	No results
TRUE	3,5-Dicyclohexyl-3,5-bis[(dimethylsilyl)oxy]-1,1,7,7-te	C20 H50 O5	No match	No results
TRUE			Unused	No results
TRUE			Unused	No results
TRUE			Unused	No results
TRUE			Unused	No results
TRUE			No results	No results
TRUE			Unused	No results
TRUE			Unused	No results
TRUE			No results	No results
TRUE	1,5-Bis[2-(bicyclo[2.2.1]hept-5-en-2-yl)ethyl]-1,1,3,3,	C24 H44 O2	No match	No results
TRUE			Unused	No results
TRUE			No results	No results
TRUE			Unused	No results
TRUE			Unused	No results
TRUE	3',5,5'-Trihydroxy-4',6,7,8-tetramethoxyflavone	C19 H18 O9	Full match	No results
TRUE			Unused	No results

TRUE			Unused	No results
TRUE	5,7,3'-Trimethoxy-4',5'-methylenedioxyflavone	C19 H16 O7	No match	No results
TRUE	(25S)-5β-spirostan-3β-ol3-O-β-D-glu	C33 H54 O8	Not the top	No results
TRUE			Unused	No results
TRUE			Unused	No results
TRUE			Unused	No results
TRUE			Unused	No results
TRUE			No results	No results
TRUE			Unused	No results
TRUE			Unused	No results
TRUE	Metaflumizone	C24 H16 F6 I	No match	No results
TRUE	Isoorientin 2"-p-hydroxybenzoate	C28 H24 O13	Not the top	No results
TRUE			Unused	No results
TRUE			Unused	No results
TRUE			Unused	No results
TRUE	Silandrin	C25 H22 O9	No results	No results
TRUE			Unused	No results
TRUE			Unused	No results
TRUE			Unused	No results
TRUE			Unused	No results
TRUE			No results	No results
TRUE			Unused	No results
TRUE			Unused	No results
TRUE			Unused	No results
TRUE			Unused	No results
TRUE			Unused	No results
TRUE			Unused	No results
TRUE			Unused	No results
TRUE			Unused	No results
TRUE			Unused	No results
TRUE			No results	No results
TRUE	6,3'-Dihydroxy-4,4'-dimethoxy-5-methylaurone	C18 H16 O6	No match	No results
TRUE			Unused	No results
TRUE			No results	No results
TRUE			No results	No results
TRUE			Unused	No results
TRUE			Unused	No results
TRUE			No results	No results
TRUE			Unused	No results
TRUE	Silybin	C25 H22 O10	No match	No results
TRUE			Unused	No results
TRUE			Unused	No results
TRUE			Unused	No results
TRUE			No results	No results
TRUE			Unused	No results
TRUE			No results	No results
TRUE			Unused	No results
TRUE			Unused	No results
TRUE			No results	No results
TRUE			Unused	No results

TRUE			Unused	No results
TRUE			Unused	No results
TRUE	Thiocolchicoside	C27 H33 N C	No match	No results
TRUE	trans-Cinnamoylbeta-D-glucoside	C15 H18 O7	Not the top	No results
TRUE			Unused	No results
TRUE			Unused	No results
TRUE			Unused	No results
TRUE	N,N-Dimethyl-1-dodecanamine	C14 H31 N	Full match	No results
TRUE			Unused	No results
TRUE			Unused	No results
TRUE			Unused	No results
TRUE			No results	No results
TRUE			No results	No results
TRUE			Unused	No results
TRUE			Unused	No results
TRUE			Unused	No results
TRUE			Unused	No results
TRUE			Unused	No results
TRUE			Unused	No results
TRUE			Unused	No results
TRUE			Unused	No results
TRUE			Unused	No results
TRUE			No results	No results
TRUE			Unused	No results
TRUE			Unused	No results
TRUE	Apigenin 7- (2"-glucosyllactate)	C24 H24 O12	Not the top	No results
TRUE			Unused	No results
TRUE			Unused	No results
TRUE			Unused	No results
TRUE			Unused	No results
TRUE			Unused	No results
TRUE			Unused	No results
TRUE			Unused	No results
TRUE			Unused	No results
TRUE			Unused	No results
TRUE			Unused	No results
TRUE	(R)-2-Hydroxyglutarate	C5 H8 O5	No results	No results
TRUE	Hyperforin	C35 H52 O4	Not the top	No results
TRUE			Unused	No results
TRUE			No results	No results
TRUE			Unused	No results
TRUE			Unused	No results
TRUE	2,2-Bis(hydroxymethyl)-1,3-propanediyl bis{3-[4-hydı	C39 H60 O8	No match	No results
TRUE			No results	No results
TRUE			Unused	No results
TRUE			Unused	No results
TRUE	(1R,4S,4aS,8aS)-1-(Hydroxymethyl)-2,5,5,8a-tetrame	C15 H26 O3	Full match	No results
TRUE	Lacosamide	C13 H18 N2	Full match	No results
TRUE			Unused	No results
TRUE			No results	No results

TRUE			Unused	No results
TRUE			No results	No results
TRUE			No results	No results
TRUE			Unused	No results
TRUE			Unused	No results
TRUE			No results	No results
TRUE			No results	No results
TRUE			Unused	No results
TRUE			Unused	No results
TRUE			Unused	No results
TRUE			Unused	No results
TRUE			Unused	No results
TRUE			No results	No results
TRUE			Unused	No results
TRUE			Unused	No results
TRUE			Unused	No results
TRUE			Unused	No results
TRUE			Unused	No results
TRUE			Unused	No results
TRUE			Unused	No results
TRUE			Unused	No results
TRUE			Unused	No results
TRUE			Unused	No results
TRUE			Unused	No results
TRUE	Bractein	C21 H20 O12	No results	No results
TRUE			Unused	No results
TRUE			Unused	No results
TRUE			Unused	No results
TRUE			Unused	No results
TRUE			No results	No results
TRUE			Unused	No results
TRUE			Unused	No results
TRUE			Unused	No results
TRUE			Unused	No results
TRUE			Unused	No results
TRUE			Unused	No results
TRUE			No results	No results
TRUE			Unused	No results
TRUE	Convallatoxin	C29 H42 O10	Not the top	No results
TRUE			Unused	No results
TRUE	[STtrihydrox]3alpha_11beta_21-5alpha-trihydroxy-pr	C21 H34 O4	Full match	No results
TRUE			Unused	No results
TRUE			No results	No results
TRUE	[FAamino_oxo(6:0/2:0)]2-amino-3-oxo-hexanedioica	C6 H9 N O5	Not the top	No results
TRUE			Unused	No results
TRUE			Unused	No results
TRUE	Cyclic dehypoxanthine futalosine	C14 H14 O7	No match	No results
TRUE	(9Z)-(13S)-12_13-Epoxyoctadeca-9_11-dienoicacid	C18 H30 O3	Full match	No results
TRUE	(9S_10S)-9_10-Dihydroxyoctadecanoate	C18 H36 O4	Full match	No results
TRUE			Unused	No results

TRUE			Unused	No results
TRUE			Unused	No results
TRUE	Silybin	C25 H22 O1(	Not the top	No results
TRUE	dTDP-6-deoxy-L-mannose	C16 H26 N2	No results	No results
TRUE			Unused	No results
TRUE			Unused	No results
TRUE			No results	No results
TRUE			Unused	No results
TRUE			No results	No results
TRUE	(R)-2-Hydroxyglutarate	C5 H8 O5	Full match	No results
TRUE			Unused	No results
TRUE			Unused	No results
TRUE			Unused	No results
TRUE			Unused	No results
TRUE			No results	No results
TRUE			No results	No results
TRUE			Unused	No results
TRUE			No results	No results
TRUE			Unused	No results
TRUE			No results	No results
TRUE			No results	No results
TRUE	Scopolin	C16 H20 O9	No results	No results
TRUE	Luteolin 7-sulfate	C15 H10 O9	No match	No results
TRUE			Unused	No results
TRUE			Unused	No results
TRUE	Chrysin 5-xyloside	C20 H18 O8	No match	No results
TRUE			No results	No results
TRUE			Unused	No results
TRUE			Unused	No results
TRUE			No results	No results
TRUE			Unused	No results
TRUE			Unused	No results
TRUE			Unused	No results
TRUE	Gericudranin C	C22 H18 O8	Not the top	No results
TRUE			Unused	No results
TRUE			Unused	No results
TRUE			Unused	No results
TRUE	Hypnum acid	C22 H16 O9	No results	No results
TRUE			Unused	No results
TRUE			Unused	No results
TRUE			Unused	No results
TRUE			Unused	No results
TRUE			Unused	No results
TRUE			Unused	No results
TRUE	Cortisol-21-Hemisuccinate	C25 H34 O8	Not the top	No results
TRUE			No results	No results
TRUE			Unused	No results
TRUE			Unused	No results
TRUE			Unused	No results
TRUE			No results	No results

TRUE			Unused	No results
TRUE			Unused	No results
TRUE			Unused	No results
TRUE			Unused	No results
TRUE	[FA(18:3)]13S-hydroperoxy-9Z_11E_14Z-octadecatrie	C18 H30 O4	Not the top	No results
TRUE			Unused	No results
TRUE	METHYL 3,5-DI-TERT-BUTYL-4-HYDROXYBENZOATE	C16 H24 O3	Not the top	No results
TRUE			No results	No results
TRUE	Telephioidin	C21 H20 O13	Not the top	No results
TRUE			Unused	No results
TRUE			Unused	No results
TRUE			No results	No results
TRUE			Unused	No results
TRUE	2-[3-Carboxy-3-(methylammonio)propyl]-L-histidine	C11 H19 N4	No match	No results
TRUE			Unused	No results
TRUE			Unused	No results
TRUE			Unused	No results
TRUE			Unused	No results
TRUE	Diospyrin	C21 H24 O13	No match	No results
TRUE			No results	No results
TRUE			Unused	No results
TRUE			Unused	No results
TRUE			Unused	No results
TRUE	CDP-N-dimethylethanolamine	C13 H24 N4	No match	No results
TRUE			Unused	No results
TRUE			No results	No results
TRUE			Unused	No results
TRUE	131-Hydroxy-magnesium-protoporphyrin IX13-mono	C35 H34 Mg	No match	No results
TRUE			Unused	No results
TRUE			Unused	No results
TRUE			Unused	No results
TRUE			Unused	No results
TRUE			Unused	No results
TRUE			No results	No results
TRUE	1,3-Bis(3-(2,3-epoxypropoxy)propyl)tetramethyldisilo	C16 H34 O5	No match	No results
TRUE			No results	No results
TRUE	Luteolin 7-sulfate	C15 H10 O9	No results	No results
TRUE	Leu-Pro(Leucyl-Proline)	C11 H20 N2	Full match	No results
TRUE			Unused	No results
TRUE			Unused	No results
TRUE			Invalid mass	No results
TRUE	L-Kynurenine	C10 H12 N2	No results	No results
TRUE			Unused	No results
TRUE			Unused	No results
TRUE	Pseudosindorin	C15 H12 O5	Full match	No results
TRUE			No results	No results
TRUE			Unused	No results
TRUE			No results	No results
TRUE			Unused	No results
TRUE			No results	No results

TRUE			Unused	No results
TRUE			Unused	No results
TRUE			No results	No results
TRUE	Pedicellin	C20 H22 O6	No match	No results
TRUE			Unused	No results
TRUE			No results	No results
TRUE			Unused	No results
TRUE			Unused	No results
TRUE			Unused	No results
TRUE	3_4-Dihydroxy-L-phenylalanine(L-Dopa)	C9 H11 N O4	No match	No results
TRUE			Unused	No results
TRUE			Unused	No results
TRUE			Unused	No results
TRUE			Unused	No results
TRUE			No results	No results
TRUE			Unused	No results
TRUE			No results	No results
TRUE			Unused	No results
TRUE			Unused	No results
TRUE			Unused	No results
TRUE			No results	No results
TRUE			Unused	No results
TRUE			Unused	No results
TRUE	2-Formylglutarate	C6 H8 O5	Full match	No results
TRUE			No results	No results
TRUE			Unused	No results
TRUE			Unused	No results
TRUE	Methyl 3-[(trimethylsilyl)oxy]-2-butenoate	C8 H16 O3 S	No match	No results
TRUE			Unused	No results
TRUE			Unused	No results
TRUE			Unused	No results
TRUE			Unused	No results
TRUE			No results	No results
TRUE			Unused	No results
TRUE			No results	No results
TRUE			Unused	No results
TRUE			Unused	No results
TRUE			Unused	No results
TRUE			No results	No results
TRUE	Formononetin 7-O- (6"-acetylglucoside)	C24 H24 O1(	No results	No results
TRUE			Unused	No results
TRUE			Unused	No results
TRUE			No results	No results
TRUE			Unused	No results
TRUE			Unused	No results
TRUE			Unused	No results
TRUE			Unused	No results
TRUE			No results	No results
TRUE			Unused	No results
TRUE	Hypnum acid	C22 H16 O9	No match	No results

TRUE			Unused	No results
TRUE	Rengasin trimethyl ether	C19 H18 O6	No match	No results
TRUE			No results	No results
TRUE			No results	No results
TRUE			Unused	No results
TRUE			Unused	No results
TRUE			Unused	No results
TRUE			Unused	No results
TRUE	5,4'-Dihydroxy-6-C-prenylflavanone 4'-xylosyl- (1->2)	C31 H38 O12	No match	No results
TRUE			Unused	No results
TRUE			Unused	No results
TRUE	3-Methyldioxyindole	C9 H9 N O2	No results	No results
TRUE	Chlorpheniramine	C16 H19 Cl N	No match	No results
TRUE			Unused	No results
TRUE			Unused	No results
TRUE			Unused	No results
TRUE			Unused	No results
TRUE			Unused	No results
TRUE	(E)-2-(Methoxycarbonylmethyl)butenedioate	C7 H8 O6	No match	No results
TRUE			Unused	No results
TRUE			No results	No results
TRUE	(7S_8S)-DiHODE	C18 H32 O4	No results	No results
TRUE			Unused	No results
TRUE	Aromadendrin 3-beta-L-arabinopyranoside	C20 H20 O10	Not the top	No results
TRUE			Unused	No results
TRUE	Kaempferol 3- (2''-p-coumaryl-alpha-L-arabinopyrano	C29 H24 O12	Not the top	No results
TRUE			No results	No results
TRUE	[FAoxo(6:0)]N-(3-oxo-hexanoyl)-homoserinelactone	C10 H15 N O	No match	No results
TRUE			Unused	No results
TRUE			Unused	No results
TRUE			No results	No results
TRUE			No results	No results
TRUE			No results	No results
TRUE			Unused	No results
TRUE			No results	No results
TRUE	(2E) -5,7-Dihydroxy-3,6-dimethoxy-4-oxo-2-phenyl-4	C22 H20 O8	No match	No results
TRUE			No results	No results
TRUE			Unused	No results
TRUE	9,10-Dihydro-10- (4-hydroxyphenyl) -pyrano [2,3-h]	C24 H20 O8	Not the top	No results
TRUE			Invalid mass	No results
TRUE			Unused	No results
TRUE			Unused	No results
TRUE			No results	No results
TRUE			No results	No results
TRUE			No results	No results
TRUE			Unused	No results
TRUE			Unused	No results
TRUE			Unused	No results
TRUE	(3E)-4-(2-Carboxyphenyl)-2-oxobut-3-enoate	C11 H8 O5	Full match	No results
TRUE	5,3',4',5'-Tetrahydroxy-6,7-dimethoxyflavone	C17 H14 O8	Not the top	No results

TRUE			Unused	No results
TRUE			No results	No results
TRUE			Unused	No results
TRUE	6-Phospho-beta-D-glucosyl-(1_4)-D-glucose	C12 H23 O14	No match	No results
TRUE			No results	No results
TRUE			Unused	No results
TRUE			Unused	No results
TRUE			Unused	No results
TRUE	[FAdihydoxy(2:0/2:0)]6_9S-epoxy-11R_15S-dihydoxy	C20 H32 O5	Not the top	No results
TRUE			No results	No results
TRUE			No results	No results
TRUE			No results	No results
TRUE			Unused	No results
TRUE			Unused	No results
TRUE			Unused	No results
TRUE			Unused	No results
TRUE	Dehydrohistidyltryptophanyldiketopiperazine	C17 H15 N5	No results	No results
TRUE			Unused	No results
TRUE			Unused	No results
TRUE			Unused	No results
TRUE			Unused	No results
TRUE			No results	No results
TRUE			Unused	No results
TRUE			No results	No results
TRUE			Unused	No results
TRUE	8'-apo-beta-Carotenol	C30 H42 O	Not the top	No results
TRUE			Invalid mass	No results
TRUE	6a,12b-Dihydro-3,10,11,12-tetrahydroxy-6- (3,4,5-tri	C22 H16 O1(	Not the top	No results
TRUE			Unused	No results
TRUE	[FA(16:2)]N-hexadecyl-ethanolamine	C18 H37 N C	Full match	No results
TRUE			Unused	No results
TRUE			Unused	No results
TRUE			Unused	No results
TRUE			Unused	No results
TRUE	[FAtrihydroxy(18:0)]9_10_13-trihydroxy-11-octadece	C18 H34 O5	Full match	No results
TRUE			Unused	No results
TRUE			Unused	No results
TRUE			Unused	No results
TRUE	2,2-Bis(hydroxymethyl)-1,3-propanediyl bis{3-[4-hydr	C39 H60 O8	Not the top	No results
TRUE	[ST(3:0)]estra-1_3_5(10)-triene-2_4_17beta-triol	C18 H24 O3	Not the top	No results
TRUE			Unused	No results
TRUE			No results	No results
TRUE			Unused	No results
TRUE	8_11_14-Eicosatrienoicacid	C20 H34 O2	No results	No results
TRUE			Unused	No results
TRUE	Apigenin 5- (6"-malonylglucoside)	C24 H22 O13	No match	No results
TRUE			Unused	No results
TRUE	Kaempferol 3- (2",6"-di- (E) -p-coumarylglucoside)	C39 H32 O15	Not the top	No results
TRUE			Unused	No results
TRUE			No results	No results

TRUE			Unused	No results
TRUE			Unused	No results
TRUE			Unused	No results
TRUE			Unused	No results
TRUE			Unused	No results
TRUE			Unused	No results
TRUE			Unused	No results
TRUE			Unused	No results
TRUE			No results	No results
TRUE	Spiroxamine	C18 H35 N C	Full match	No results
TRUE			No results	No results
TRUE			Unused	No results
TRUE			Unused	No results
TRUE	Spiroxamine	C18 H35 N C	Full match	No results
TRUE			No results	No results
TRUE			No results	No results
TRUE			Unused	No results
TRUE			Unused	No results
TRUE			Unused	No results
TRUE			No results	No results
TRUE			Unused	No results
TRUE			Unused	No results
TRUE			Unused	No results
TRUE			Unused	No results
TRUE			No results	No results
TRUE			Unused	No results
TRUE			Unused	No results
TRUE			Unused	No results
TRUE			Unused	No results
TRUE			Unused	No results
TRUE	2,6-di-tert-butyl-4-ethylphenol	C16 H26 O	Full match	No results
TRUE			Unused	No results
TRUE			Unused	No results
TRUE	3-Dimethylallyl-4-hydroxymandelate	C13 H16 O4	No results	No results
TRUE			Unused	No results
TRUE	N-alpha-Acetylarginine	C8 H16 N4 C	No results	No results
TRUE			Unused	No results
TRUE			Unused	No results
TRUE	Icosadienoicacid	C20 H36 O2	Full match	No results
TRUE			Unused	No results
TRUE			No results	No results
TRUE			Unused	No results
TRUE			Unused	No results
TRUE			No results	No results
TRUE			Unused	No results
TRUE			Unused	No results
TRUE			No results	No results
TRUE			No results	No results
TRUE			No results	No results
TRUE			No results	No results

TRUE			No results	No results
TRUE			Unused	No results
TRUE			Unused	No results
TRUE			Unused	No results
TRUE			Unused	No results
TRUE			Unused	No results
TRUE			Unused	No results
TRUE			No results	No results
TRUE			Unused	No results
TRUE			No results	No results
TRUE			Unused	No results
TRUE			Unused	No results
TRUE			No results	No results
TRUE	(-) -Semiglabrin	C23 H20 O6	Not the top	No results
TRUE			Unused	No results
TRUE			Unused	No results
TRUE	Kanzakiflavone 1	C17 H12 O7	Full match	No results
TRUE	Silandrin	C25 H22 O9	No results	No results
TRUE			No results	No results
TRUE			Unused	No results
TRUE			Unused	No results
TRUE			No results	No results
TRUE			Unused	No results
TRUE			No results	No results
TRUE			Unused	No results
TRUE			No results	No results
TRUE			Unused	No results
TRUE			Unused	No results
TRUE			Unused	No results
TRUE			Unused	No results
TRUE			Unused	No results
TRUE	UNII:OL56191M6N	C12 H18 O4	Full match	No results
TRUE			Unused	No results
TRUE			Unused	No results
TRUE			Unused	No results
TRUE			Unused	No results
TRUE			No results	No results
TRUE			Unused	No results
TRUE			Unused	No results
TRUE			Unused	No results
TRUE			Unused	No results
TRUE	1-Pyrroline-4-hydroxy-2-carboxylate	C5 H7 N O3	No results	No results
TRUE	Hexyl fumarate	C16 H28 O4	Not the top	No results
TRUE			Unused	No results
TRUE			Unused	No results
TRUE			Unused	No results
TRUE	9,10-Dihydro-10- (4-hydroxyphenyl) -pyrano [2,3-h]	C24 H20 O8	No match	No results
TRUE			Unused	No results
TRUE			Unused	No results
TRUE			No results	No results

TRUE			Unused	No results
TRUE			Unused	No results
TRUE			Unused	No results
TRUE			Unused	No results
TRUE			Unused	No results
TRUE	7-Hydroxy-5,4'-dimethoxyflavone 8-C-rhamnoside	C23 H24 O9	No results	No results
TRUE			Unused	No results
TRUE			Unused	No results
TRUE			No results	No results
TRUE			Unused	No results
TRUE			No results	No results
TRUE			Unused	No results
TRUE			No results	No results
TRUE			Unused	No results
TRUE			Unused	No results
TRUE	Hovetrichoside C	C21 H22 O1	Not the top	No results
TRUE	2"-trans-Caffeoylisoorientin	C30 H26 O1	No results	No results
TRUE			No results	No results
TRUE			Unused	No results
TRUE			Unused	No results
TRUE	5,7,2',5'-Tetrahydroxy-6-methoxyflavanone	C16 H14 O7	Not the top	No results
TRUE			Unused	No results
TRUE			Unused	No results
TRUE			Unused	No results
TRUE			Unused	No results
TRUE			Unused	No results
TRUE			No results	No results
TRUE	6,7,3',4'-Tetrahydroxyaurone 6- (4",6"-diacetylglucos	C25 H24 O1	No match	No results
TRUE			No results	No results
TRUE			Unused	No results
TRUE			Unused	No results
TRUE			Unused	No results
TRUE			No results	No results
TRUE			Unused	No results
TRUE			Unused	No results
TRUE			No results	No results
TRUE			Unused	No results
TRUE			Unused	No results
TRUE			Unused	No results
TRUE			Unused	No results
TRUE			Unused	No results
TRUE			Unused	No results
TRUE			Unused	No results
TRUE			Unused	No results
TRUE			Unused	No results
TRUE			Unused	No results
TRUE			No results	No results
TRUE	Baohuoside 1	C27 H30 O1	Not the top	No results
TRUE			Unused	No results
TRUE	Quercetin 3-isobutyrate	C19 H16 O8	Full match	No results

TRUE			No results	No results
TRUE			No results	No results
TRUE			Unused	No results
TRUE			Unused	No results
TRUE			Unused	No results
TRUE			Unused	No results
TRUE			No results	No results
TRUE			Unused	No results
TRUE			Unused	No results
TRUE			No results	No results
TRUE			Invalid mass	No results
TRUE			No results	No results
TRUE			Unused	No results
TRUE			Unused	No results
TRUE			Unused	No results
TRUE	2,5-Cyclohexadien-1-one, 4,4'-ethanediylidenebis[2,6	C30 H42 O2	Not the top	No results
TRUE			No results	No results
TRUE	2,2'-ETHYLIDENEBIS(4,6-DI-TERT-BUTYLPHENOL)	C30 H46 O2	Full match	No results
TRUE	(2E_6E)-(10R_11S)-10_11-Epoxy-3_7_11-trimethyltri	C16 H26 O3	Full match	No results
TRUE			Unused	No results
TRUE			Unused	No results
TRUE			Unused	No results
TRUE			Unused	No results
TRUE			Unused	No results
TRUE			No results	No results
TRUE			Unused	No results
TRUE			Unused	No results
TRUE			Unused	No results
TRUE			No results	No results
TRUE			Unused	No results
TRUE	2- [3,5-Dimethoxy-4- [(3-methyl-2-butenyl) oxy] ph	C25 H28 O8	Not the top	No results
TRUE			Unused	No results
TRUE			Unused	No results
TRUE			Unused	No results
TRUE			No results	No results
TRUE			Unused	No results
TRUE			Unused	No results
TRUE			Unused	No results
TRUE			Unused	No results
TRUE			Unused	No results
TRUE	7-Hydroxy-5,4'-dimethoxyflavone 8-C-rhamnoside	C23 H24 O9	Not the top	No results
TRUE			Unused	No results
TRUE			No results	No results
TRUE	Vitexin 2''-O-p-coumarate	C30 H26 O1	Not the top	No results
TRUE			Unused	No results
TRUE			Unused	No results
TRUE			Unused	No results
TRUE			Unused	No results
TRUE			Unused	No results
TRUE			No results	No results

TRUE			No results	No results
TRUE	(R)-10-Hydroxystearate	C18 H36 O3	Full match	No results
TRUE			No results	No results
TRUE	[FA(18:3)]13S-hydroperoxy-9Z_11E_14Z-octadecatrie	C18 H30 O4	No results	No results
TRUE			No results	No results
TRUE			No results	No results
TRUE	3,3-Dimethyl-1,5-dioxacyclopentadecane-6,15-dione	C15 H26 O4	Not the top	No results
TRUE			No results	No results
TRUE			No results	No results
TRUE	Isocarbamid	C8 H15 N3 C	Full match	No results
TRUE			Unused	No results
TRUE	Zearalenone	C18 H22 O5	Full match	No results
TRUE			Unused	No results
TRUE			Unused	No results
TRUE			Unused	No results
TRUE			No results	No results
TRUE			Unused	No results
TRUE			No results	No results
TRUE			No results	No results
TRUE			No results	No results
TRUE			Unused	No results
TRUE	Quercetagetin 7- (6"-acetylglucoside)	C23 H22 O14	No results	No results
TRUE			Unused	No results
TRUE			Unused	No results
TRUE			Unused	No results
TRUE	Gibberellin A17	C20 H26 O7	No match	No results
TRUE			Unused	No results
TRUE			Unused	No results
TRUE			Unused	No results
TRUE			No results	No results
TRUE			Unused	No results
TRUE	[6]-Gingerol	C17 H26 O4	Full match	No results
TRUE			No results	No results
TRUE			Unused	No results
TRUE			Unused	No results
TRUE	[FA(18:2)]9Z_11E-octadecadienoicacid	C18 H32 O2	Full match	No results
TRUE			Unused	No results
TRUE			Unused	No results
TRUE			No results	No results
TRUE			Unused	No results
TRUE			Unused	No results
TRUE			Unused	No results
TRUE			Unused	No results
TRUE			Unused	No results
TRUE			No results	No results
TRUE			No results	No results
TRUE			No results	No results
TRUE			Unused	No results
TRUE			Unused	No results
TRUE			No results	No results

TRUE	Leptosidin	C16 H12 O6	Not the top	No results
TRUE			Unused	No results
TRUE			Unused	No results
TRUE			Unused	No results
TRUE			No results	No results
TRUE			Unused	No results
TRUE			Unused	No results
TRUE	FL1ABGGS0001_a	C16 H12 O7	Not the top	No results
TRUE			Unused	No results
TRUE			Unused	No results
TRUE			Unused	No results
TRUE			Unused	No results
TRUE			Unused	No results
TRUE	7-Methoxy-3',4'-methylenedioxyflavone	C17 H12 O5	Full match	No results
TRUE			Unused	No results
TRUE			No results	No results
TRUE			Unused	No results
TRUE			Unused	No results
TRUE			Unused	No results
TRUE			Unused	No results
TRUE			Unused	No results
TRUE			Unused	No results
TRUE			No results	No results
TRUE			Unused	No results
TRUE			Unused	No results
TRUE			Unused	No results
TRUE			Unused	No results
TRUE			No results	No results
TRUE			No results	No results
TRUE	Glabratephrin	C24 H20 O7	Full match	No results
TRUE			No results	No results
TRUE	Spiroxamine	C18 H35 N C	No results	No results
TRUE			No results	No results
TRUE			Unused	No results
TRUE			No results	No results
TRUE			Unused	No results
TRUE			Unused	No results
TRUE			No results	No results
TRUE			Unused	No results
TRUE			Unused	No results
TRUE	Etodolac	C17 H21 N C	No results	No results
TRUE			Unused	No results
TRUE			Unused	No results
TRUE			Unused	No results
TRUE			Unused	No results
TRUE			Unused	No results
TRUE			Unused	No results
TRUE			No results	No results
TRUE	Triadimenol	C14 H18 Cl N	Not the top	No results
TRUE	3-Methyloxindole	C9 H9 N O	Full match	No results

TRUE			No results	No results
TRUE			Unused	No results
TRUE			Unused	No results
TRUE			Unused	No results
TRUE			Unused	No results
TRUE			Unused	No results
TRUE			No results	No results
TRUE	Exiguaflavanone D	C31 H38 O7	No match	No results
TRUE			No results	No results
TRUE			No results	No results
TRUE			Unused	No results
TRUE			Unused	No results
TRUE			Unused	No results
TRUE			Unused	No results
TRUE			Unused	No results
TRUE			Unused	No results
TRUE			No results	No results
TRUE			Unused	No results
TRUE			Unused	No results
TRUE			Unused	No results
TRUE			Unused	No results
TRUE			Unused	No results
TRUE			Unused	No results
TRUE			Unused	No results
TRUE			Unused	No results
TRUE			Unused	No results
TRUE			Unused	No results
TRUE			Unused	No results
TRUE			No results	No results
TRUE			No results	No results
TRUE			Unused	No results
TRUE			Unused	No results
TRUE			No results	No results
TRUE			Unused	No results
TRUE			No results	No results
TRUE			Unused	No results
TRUE			No results	No results
TRUE			No results	No results
TRUE			Unused	No results
TRUE			Unused	No results
TRUE	3',4'-Methylenedioxy-2,4,6,beta-tetramethoxychalco	C20 H20 O7	No match	No results
TRUE			No results	No results
TRUE			Unused	No results
TRUE			Unused	No results
TRUE	Viscutin 1	C27 H26 O1	No results	No results
TRUE			Unused	No results
TRUE			Unused	No results
TRUE			No results	No results
TRUE			Unused	No results
TRUE			No results	No results

TRUE			Unused	No results
TRUE			No results	No results
TRUE			Unused	No results
TRUE			Unused	No results
TRUE			Unused	No results
TRUE			No results	No results
TRUE	Pteridine	C6 H4 N4	Full match	No results
TRUE	Geranylacetate	C12 H20 O2	No results	No results
TRUE			No results	No results
TRUE			No results	No results
TRUE			Unused	No results
TRUE			Unused	No results
TRUE			Unused	No results
TRUE			No results	No results
TRUE			Unused	No results
TRUE			Unused	No results
TRUE	Verrucarol	C15 H22 O4	No results	No results
TRUE			No results	No results
TRUE			Unused	No results
TRUE			Unused	No results
TRUE			No results	No results
TRUE	Torosaflavone A	C21 H20 O8	Not the top	No results
TRUE			No results	No results
TRUE			Unused	No results
TRUE			Unused	No results
TRUE	6",6"-Dimethylpyrano [2",3":7,8] kaempferol 4'-met	C27 H28 O1(	No results	No results
TRUE			Unused	No results
TRUE	Triadimenol	C14 H18 Cl N	Full match	No results
TRUE			Unused	No results
TRUE			No results	No results
TRUE			No results	No results
TRUE			Unused	No results
TRUE			Unused	No results
TRUE			Unused	No results
TRUE			No results	No results
TRUE			Unused	No results
TRUE			Unused	No results
TRUE			No results	No results
TRUE			Unused	No results
TRUE			Unused	No results
TRUE			No results	No results
TRUE			No results	No results
TRUE			Unused	No results
TRUE			Unused	No results
TRUE			No results	No results
TRUE			Unused	No results
TRUE			No results	No results
TRUE			No results	No results
TRUE			No results	No results
TRUE			No results	No results

TRUE			Unused	No results
TRUE			Unused	No results
TRUE			Unused	No results
TRUE			No results	No results
TRUE			No results	No results
TRUE	all-trans-Heptaprenyldiphosphate	C35 H60 O7	No match	No results
TRUE			Unused	No results
TRUE			Unused	No results
TRUE	Metamitron	C10 H10 N4	No results	No results
TRUE			Unused	No results
TRUE	Xanthoxin;Methoxsalen	C15 H22 O3	No results	No results
TRUE			Unused	No results
TRUE			No results	No results
TRUE			No results	No results
TRUE			No results	No results
TRUE			Unused	No results
TRUE			No results	No results
TRUE			Unused	No results
TRUE	Luteolin 3'- (3"-acetylglucuronide)	C23 H20 O1:	No match	No results
TRUE			Unused	No results
TRUE			No results	No results
TRUE			No results	No results
TRUE			No results	No results
TRUE			No results	No results
TRUE			No results	No results
TRUE			No results	No results
TRUE			Unused	No results
TRUE			Unused	No results
TRUE			Unused	No results
TRUE			No results	No results
TRUE			No results	No results
TRUE			No results	No results
TRUE			Unused	No results
TRUE	4-Ethoxy ethylbenzoate	C11 H14 O3	No results	No results
TRUE			Unused	No results
TRUE			Unused	No results
TRUE			Unused	No results
TRUE			No results	No results
TRUE			No results	No results
TRUE			No results	No results
TRUE			Unused	No results
TRUE			No results	No results
TRUE			Unused	No results
TRUE			Unused	No results

MS

Annotation S	Annotation S	Annotation S	Annotation S	FISh Coverag	Molecular W	RT [min]
Full match	Full match	No results	Full match		194.08033	6.275
Invalid mass	No results	No results	No results		124.01483	2.075
Invalid mass	No results	No results	No results		170.02032	2.121
Invalid mass	No results	No results	No results		170.02032	1.743
No results	No results	No results	No match		273.26659	10
No results	No results	No results	No results		195.08362	6.455
No results	Not the top	No results	Full match		141.11544	1.714
No results	No results	No results	No results		791.58857	7.006
Full match	No results	No results	Not the top		306.07367	3.904
No results	No results	No results	No results		132.04121	0.955
Full match	No results	No results	Not the top		458.08453	6.385
Partial matcl	Partial matcl	No results	Full match		117.07924	0.929
Invalid mass	No results	No results	No results		170.0203	1.21
No results	Partial matcl	No results	Full match		756.21108	6.563
No results	No results	No results	No results		678.50445	6.892
No results	No results	No results	No results		904.67256	7.089
No results	No results	No results	No results		401.1473	6.657
No results	No results	No results	No results		401.14727	6.525
No results	No results	No results	Full match		414.204	12.343
No results	No results	No results	No results		126.03033	2.1
Partial matcl	No results	No results	Not the top		564.14781	6.329
No results	No results	No results	Full match		519.33262	13.442
Invalid mass	Not the top	No results	Full match		180.0648	3.936
No results	Not the top	No results	Full match		141.11544	1.042
Full match	No results	No results	Not the top		306.07366	6.014
No results	No results	No results	Full match		143.13114	2.561
No results	No results	No results	No results		417.14223	6.257
No results	Partial matcl	No results	Full match		129.07918	2.234
No results	No results	No results	No results		274.2698	9.984
Partial matcl	Full match	No results	Full match		135.05457	0.994
Invalid mass	No results	No results	No results		118.02807	0.986
No results	No results	No results	Full match		495.33258	14.004
No results	No results	No results	No results		417.14223	6.37
No results	No results	No results	No results		514.31436	13.719
No results	No results	No results	Full match		143.13115	2.218
No results	No results	No results	No results		565.42041	6.752
No results	No results	No results	No results		282.71007	6.767
No results	No results	No results	No results		464.22572	6.896
No results	No results	No results	Full match		202.05415	18.146
No results	Partial matcl	No results	Full match		772.20582	6.415
No results	No results	No results	No results		676.36683	12.361
No results	No results	No results	No results		792.59141	7.018
No results	No results	No results	Full match		143.13122	2.306
No results	No results	No results	Full match		512.06196	6.514
No results	No results	No results	No results		289.26163	10.203
Invalid mass	No results	No results	No results		146.02053	1.001
Partial matcl	Partial matcl	No results	Not the top		432.10542	6.629
No results	No results	No results	No results		350.06165	0.892

Partial matcl	Full match	No results	Full match		135.0546	1.411
Invalid mass	No results	No results	No results		118.02807	1.421
No results	No results	No results	No results		905.67602	7.116
No results	No results	No results	No results		517.31706	12.548
No results	No results	No results	No results		572.07741	6.4
No results	No results	No results	No results		124.97918	18.159
No results	Partial matcl	No results	Full match		232.10583	0.999
No results	No results	No results	Full match		182.07097	7.862
No results	No results	No results	No results		204.16261	6.614
No results	No results	No results	No results		415.20735	12.341
Invalid mass	No results	No results	No results		188.03087	1.498
No results	No results	No results	No results		229.24063	10.088
No results	No results	No results	No results		166.04658	0.921
No results	No results	No results	No results		578.27594	14.229
No results	No results	No results	Full match		312.10557	0.884
No results	No results	No results	No results		260.16355	6.734
No results	No results	No results	No results		1017.7574	7.185
No results	No results	No results	No results		448.23077	8.082
No results	No results	No results	Full match		257.10277	0.923
No results	No results	No results	No results		578.27576	14.404
No results	No results	No results	No results		166.0057	18.166
No results	No results	No results	Full match		157.07396	1.004
No results	No results	No results	No results		276.06319	7.049
No results	No results	No results	No results		295.05882	0.885
No results	No results	No results	Full match		281.08978	5.929
No results	No results	No results	No results		205.9876	3.935
Full match	No results	No results	Full match		294.18278	11.378
No results	No results	No results	Full match		521.34844	14.398
Invalid mass	No results	No results	No results		154.02541	4.212
Invalid mass	Not the top	No results	Full match		180.06478	3.726
No results	No results	No results	No results		578.27585	14.034
No results	No results	No results	No results		129.04131	1.038
No results	No results	No results	No results		109.00161	0.766
Full match	No results	No results	Not the top		578.14235	6.214
No results	No results	No results	Full match		452.33628	6.575
No results	No results	No results	No results		774.52839	18.093
Partial matcl	Partial matcl	No results	Not the top		594.15841	6.735
No results	No results	No results	Full match		317.29304	10.124
No results	No results	No results	Partial matcl		480.08983	6.453
No results	No results	No results	No results		243.25628	10.331
No results	No results	No results	No results		416.16806	6.533
No results	No results	No results	No results		757.21406	6.561
No results	No match	No results	No match		180.06368	5.832
No results	No results	No results	No results		558.3231	13.719
No results	No results	No results	No match		251.07945	2.162
No results	No results	No results	No results		300.13213	2.036
No results	No results	No results	No results		266.07891	2.036
No results	No results	No results	No results		283.10552	2.037
No results	No results	No results	Full match		187.06357	5.776
Full match	Partial matcl	No results	Full match		204.09013	5.888

No results	No results	No results	Full match		512.06204	6.154
Full match	No results	No results	Full match		302.00583	6.732
No results	No results	No results	No results		592.26891	16.748
No results	No results	No results	No match		211.12091	1.006
No results	Partial matcl	No results	Full match		654.38273	13.802
No results	No results	No results	No results		612.14753	3.932
No results	No results	No results	Full match		157.07396	1.425
Invalid mass	No results	No results	No results		154.02536	4.764
No results	No results	No results	No results		352.26134	13.717
No results	No results	No results	No results		556.08227	6.77
No results	No results	No results	No results		477.28563	13.355
No results	No results	No results	Full match		103.10015	0.892
No results	No results	No results	No results		254.93703	18.161
Full match	Full match	No results	Full match		267.0967	1.465
Partial matcl	Partial matcl	No results	Not the top		318.03723	7.383
No results	No results	No results	No results		563.34205	13.445
No results	Partial matcl	No results	Full match		216.11116	2.27
No results	No results	No results	No results		418.1455	6.265
No results	No results	No results	No results		130.02539	1.036
No results	No results	No results	No results		150.02805	0.768
No results	No results	No results	No results		140.01074	2.103
No results	No results	No results	Full match		228.14747	1.434
No results	No results	No results	No results		402.15058	6.654
No results	No results	No results	No results		294.16542	15.388
Full match	Not the top	No results	Not the top		610.15327	6.533
No results	No results	No results	No results		402.15061	6.524
No results	No results	No results	No results		612.14741	4.552
No results	No results	No results	No results		796.18477	6.396
Full match	Partial matcl	No results	Not the top		594.15832	6.191
No results	Partial matcl	No results	Full match		232.10582	1.419
No results	No results	No results	No results		260.10084	2.067
No results	No results	No results	No results		312.08446	6.199
No results	No results	No results	Partial matcl		340.23968	16.077
Invalid mass	No results	No results	No results		175.96234	18.206
No results	No results	No results	No results		720.37731	12.38
No results	No results	No results	No results		329.32942	12.738
No results	No results	No results	No results		252.18362	6.593
No results	No results	No results	No results		539.34137	14.003
No results	No results	No results	No results		592.01907	6.195
No results	No results	No results	No results		433.19454	6.541
No results	No results	No results	No results		264.96744	2.076
No results	No results	No results	Full match		326.19123	17.995
No results	No results	No results	Full match		211.07615	0.962
No results	No results	No results	No results		594.28043	13.313
No results	No results	No results	No results		400.1271	6.044
No results	No results	No results	No results		418.14543	6.379
Invalid mass	No results	No results	No results		138.03052	6.133
No results	No results	No results	No results		561.32587	12.558
No results	No results	No results	No results		347.03213	2.097
No results	No results	No results	No results		569.15334	6.659

No results	No results	No results	No results		129.04127	1.418
No results	No results	No results	No results		452.15972	12.343
No results	No results	No results	Full match		283.28753	17.849
No results	No results	No results	No results		612.14766	6.015
No results	No results	No results	No results		434.18044	6.039
No results	No results	No results	Partial matcl		534.13727	6.498
No results	No results	No results	No results		417.15351	6.039
Invalid mass	No match	No results	No match		148.0526	2.819
No results	No results	No results	No results		566.42402	6.719
No results	No results	No results	No results		387.13183	7.001
Full match	Full match	No results	Full match		165.07907	2.808
Invalid mass	No results	No results	No results		184.03617	6.23
No results	No results	No results	No results		432.23382	12.35
No results	Partial matcl	No results	Full match		360.13171	6.246
No results	No results	No results	No results		134.02024	0.996
No results	No results	No results	No results		443.09267	6.764
No results	No results	No results	No results		870.2035	6.56
No results	No results	No results	No results		461.1316	6.391
Invalid mass	Partial matcl	No results	Full match		180.06484	5.858
No results	No results	No results	No results		401.15846	6.246
No results	No results	No results	No match		344.07436	2.063
No results	No results	No results	No results		213.91041	18.163
No results	No results	No results	No results		534.26346	17.558
No results	No results	No results	No results		453.28544	13.918
No results	No results	No results	No results		132.04096	3.29
No results	No results	No results	No results		556.29124	17.831
No results	No results	No results	No results		205.98756	4.459
No results	No results	No results	Full match		119.07385	2.817
No results	No match	No results	No match		301.29817	11.414
No results	No results	No results	No results		916.1692	6.401
Invalid mass	No results	No results	No match		258.05251	6.357
No results	No results	No results	No results		187.04713	1.039
No results	No results	No results	No results		253.97365	2.083
No results	No results	No results	No results		358.11101	0.874
No results	No results	No results	No results		141.04159	1.028
No results	No results	No results	No results		140.04378	0.766
No results	No results	No results	No results		273.966	0.766
No results	Partial matcl	No results	Full match		196.05732	0.915
No results	No results	No results	No results		176.0309	1.585
No results	No results	No results	No results		122.0333	0.768
No results	No results	No results	No results		148.03593	1.597
No results	No results	No results	No results		573.08069	6.414
No results	No results	No results	No results		401.11045	6.387
No results	No results	No results	No results		556.2913	17.507
Invalid mass	No results	No results	No results		278.0801	6.098
No results	No results	No results	No results		138.01079	18.302
No results	No results	No results	No results		531.13502	6.274
No results	No results	No results	Full match		199.1208	1.487
No results	No results	No results	No results		405.14244	7
Partial matcl	Partial matcl	No results	Not the top		302.04237	6.41

No results	No results	No results	Full match		240.14732	1.498
No results	No results	No results	No results		793.59519	6.945
No results	No results	No results	No results		205.98758	2.156
No results	No results	No results	No results		724.50969	6.872
No results	No results	No results	No results		266.12672	5.917
No results	No results	No results	No results		850.52058	18.104
No results	No results	No results	Full match		118.07852	12.346
No results	No results	No results	No results		126.03033	2.564
No results	No results	No results	No results		612.47545	14.764
No results	No results	No results	No results		127.01214	0.766
No results	No results	No results	No results		557.31967	13.719
No results	No match	No results	No match		294.21922	13.473
Full match	Not the top	No results	Not the top		448.10078	6.934
No results	No results	No results	No results		148.03593	1.134
No results	No results	No results	Full match		204.02613	0.995
No match	No match	No results	No match		578.16338	6.504
No results	No results	No results	No results		553.15863	6.882
No results	No results	No results	No results		145.02272	0.763
No results	No results	No results	Full match		143.13116	1.071
No results	No results	No results	No results		195.0772	6.489
No results	No results	No results	Full match		332.18358	7.134
No results	No results	No results	No results		392.27883	12.058
No results	No results	No results	No results		518.32051	12.562
No results	No results	No results	Full match		137.05882	6.281
No results	No results	No results	No results		492.23878	8.087
No results	No results	No results	No results		348.08206	0.903
No results	No results	No results	No results		208.05761	0.918
No results	Partial matcl	No results	Full match		345.07746	2.059
No results	No results	No results	No results		773.2093	6.412
No results	Partial matcl	No results	Full match		330.14667	6.921
No results	No results	No results	Full match		223.12078	0.998
No results	No results	No results	Full match		208.12109	1.004
No results	No results	No results	Full match		228.14736	1.715
No results	No results	No results	No results		357.36079	13.94
No results	No results	No results	Full match		240.14733	1.004
No results	No results	No results	No results		565.15106	6.325
No results	Partial matcl	No results	Full match		145.07409	2.161
Full match	Partial matcl	No results	Full match		244.06912	1.417
No results	No results	No results	No results		832.50992	18.1
No results	Partial matcl	No results	Full match		636.09617	6.29
No results	No results	No results	No results		173.88868	0.87
No results	No results	No results	Full match		337.33447	16.255
Full match	Not the top	No results	Not the top		448.10071	6.546
No results	No results	No results	No results		140.00973	2.108
No results	No results	No results	Full match		190.14697	6.231
No results	No results	No results	No results		828.40806	12.351
No results	No results	No results	Full match		311.31883	16.921
No results	No results	No results	No results		426.09835	0.894
No results	No results	No results	No results		222.98176	0.765
No results	No results	No results	No results		154.03959	7.866

No results	No results	No results	No results		138.98082	18.169
No results	No results	No results	No results		762.5178	18.055
No results	No results	No results	No results		410.08221	0.908
No results	No results	No results	No results		223.07065	2.057
No results	No results	No results	No results		138.01076	18.153
No results	No results	No results	Full match		452.13169	6.012
No results	No results	No results	No results		817.53731	14.631
No results	No results	No results	Full match		270.12163	6.108
No results	No results	No results	No results		392.95422	2.139
No results	No results	No results	No results		141.0416	1.426
No results	No results	No results	Full match		257.10268	1.027
No results	No results	No results	No results		837.59384	7
No results	Partial match	No results	Full match		170.02164	2.077
No results	No results	No results	No results		221.06724	6.449
No results	No results	No results	No results		197.11648	0.851
No results	No results	No results	No results		719.37256	12.368
No results	No results	No results	No results		412.0309	2.6
No results	No results	No results	No results		305.92101	18.155
No results	No results	No results	No results		272.05624	0.913
No results	No results	No results	No results		199.96579	0.765
No results	No results	No results	No results		475.26973	12.477
No results	No results	No results	Full match		169.03757	2.05
Full match	Full match	No results	Full match		243.08553	0.965
No results	No results	No results	No results		936.58107	15.287
No results	No results	No results	No results		522.21029	6.943
No results	No results	No results	No results		477.28542	14.42
No results	No results	No results	No results		459.26173	12.345
No results	No results	No results	No results		152.01108	2.125
No results	No results	No results	No results		194.04147	0.961
No results	Partial match	No results	Full match		187.04715	1.414
No results	No results	No results	No results		721.51005	6.906
No results	No results	No results	No results		386.0305	6.273
No results	No results	No results	Full match		175.10376	0.996
No results	No results	No results	No results		569.15342	6.515
No results	No results	No results	No results		295.96367	18.16
No results	No results	No results	No results		445.24606	12.346
No results	No results	No results	No results		436.1856	12.346
No results	No results	No results	No results		404.07133	6.159
No results	No results	No results	No results		166.03008	0.944
No results	No results	No results	No results		358.06601	6.16
No results	Partial match	No results	Full match		332.07421	1.596
No results	No results	No results	No results		562.33912	13.444
No results	No results	No results	No results		215.11585	1.524
No results	No results	No results	No results		332.25607	9.118
No results	No results	No results	Full match		281.11244	2.142
No results	No results	No results	Full match		232.14235	1.492
No results	No results	No results	Full match		145.08518	0.995
No results	No results	No results	Full match		292.02164	6.182
No results	No results	No results	No results		159.98466	0.769
No results	No results	No results	Full match		191.1313	18.179

No results	No results	No results	No results		220.02676	18.164
No results	No results	No results	No results		423.15255	6.295
No results	No results	No results	Full match		452.11042	7.046
No results	No results	No results	No results		596.2964	14.74
No results	No results	No results	No results		390.02579	3.914
No results	No results	No results	Full match		238.09526	6.115
No results	No results	No results	Full match		372.14036	6.38
No results	No results	No results	No results		205.98764	1.858
Full match	No results	No results	Not the top		442.08991	6.762
No results	No results	No results	Full match		304.127	1.005
No results	Partial matcl	No results	Full match		127.06362	2.154
No results	No results	No results	Full match		362.06364	6.254
No results	No results	No results	No results		364.00096	2.067
No results	No results	No results	No results		389.14727	6.949
No results	No results	No results	No results		243.08034	18.151
No results	No results	No results	Full match		223.0839	3.073
No results	No results	No results	No results		248.06804	6.92
No results	No results	No results	No results		640.18522	3.03
No results	No results	No results	No results		282.97807	2.171
No results	No results	No results	No match		414.20397	11.685
No results	No results	No results	Full match		632.10054	6.313
No results	No results	No results	No results		150.01523	0.951
Partial matcl	Partial matcl	No results	Not the top		464.09554	6.694
No results	No results	No results	No results		744.49313	18.035
No results	No results	No results	No results		306.05592	0.958
No results	No results	No results	No results		250.98131	2.06
No results	No results	No results	No results		443.12134	6.871
No results	No results	No results	No results		385.15242	6.853
No results	No results	No results	No results		238.16816	6.121
No results	No results	No results	No results		762.50411	18.061
No results	Partial matcl	No results	Full match		244.06918	1.007
No results	No results	No results	No results		445.13674	6.624
No results	No results	No results	Full match		386.173	11.154
No results	Partial matcl	No results	Full match		173.06882	0.983
No results	No results	No results	Full match		287.24608	10.572
No results	No results	No results	No results		816.50548	18.019
No results	No results	No results	No results		458.06679	2.084
No results	No results	No results	No results		1176.5562	8.664
Invalid mass	No results	No results	No results		164.04619	6.842
No results	No results	No results	No results		227.18865	10.31
No results	Full match	No results	No results		378.16778	6.93
No results	No results	No results	No results		272.10081	0.993
No results	No results	No results	No results		678.38287	13.235
No results	No results	No results	No results		404.07133	6.361
No results	No results	No results	No results		352.26134	12.314
No results	No results	No results	No results		554.31023	12.356
No results	Partial matcl	No results	Full match		288.06307	6.374
No results	No results	No results	No results		248.03151	6.576
No results	No results	No results	No results		339.1032	6.394
No results	No results	No results	No results		579.14566	6.211

No results	No results	No results	Full match		280.13084	12.347
No results	No results	No results	No results		264.99712	6.724
No results	No results	No results	No results		680.39847	14.205
No results	No results	No results	No results		232.07296	7.046
No results	No results	No results	No results		515.3247	12.349
No results	No results	No results	Full match		366.23109	18.131
No results	No results	No results	Full match		470.12085	6.188
No results	No results	No results	No results		183.99982	1.062
No results	No results	No results	No results		416.20996	12.348
No results	Partial match	No results	Full match		366.05654	2.145
No results	No results	No results	No results		490.07445	6.315
No results	Partial match	No results	Full match		189.04271	6.029
No results	No results	No results	No results		210.16208	10.299
No results	Partial match	No results	Full match		268.07912	0.939
No results	Partial match	No results	Full match		402.09474	7.586
No results	No results	No results	No results		270.11023	6.459
No results	No results	No results	No results		449.10358	6.927
No results	Partial match	No results	Full match		347.0629	1.418
No results	Partial match	No results	Full match		276.132	0.996
Full match	No results	No results	Full match		152.1202	7.092
No results	Partial match	No results	Full match		320.05271	6.013
No results	Partial match	No results	Full match		345.07746	1.058
No results	No results	No results	Full match		788.20125	6.266
Invalid mass	No results	No results	No results		154.02539	6.507
No results	No results	No results	No results		245.23559	8.623
No results	No results	No results	Full match		111.06855	0.985
No results	No results	No results	No results		742.47816	18.047
No results	No results	No results	No results		797.51317	14.551
Invalid mass	No results	No results	No results		154.02541	3.283
No results	No results	No results	No results		352.26144	15.282
No results	No results	No results	No results		174.0993	1.01
No results	No results	No results	No results		180.06232	0.978
No results	No results	No results	No results		701.48979	6.84
No results	No results	No results	No results		456.27321	15.387
No results	Partial match	No results	Full match		594.15876	6.428
Full match	No results	No results	Not the top		578.14251	6.049
Invalid mass	No results	No results	No results		174.0993	1.418
No results	No results	No results	No results		421.06975	6.032
No results	No results	No results	No results		689.21062	6.341
No results	Partial match	No results	Full match		221.0896	0.926
No results	No results	No results	No results		237.11205	11.383
No results	No results	No results	No results		596.29626	15.04
No results	No results	No results	Full match		135.05321	1.023
No results	No results	No results	Full match		113.08425	0.996
Full match	Partial match	No results	Full match		283.09163	1.557
No results	No results	No results	Full match		193.11046	18.123
No results	No results	No results	No results		443.12131	7.201
Full match	Full match	No results	Not the top		318.03742	6.446
No results	No results	No results	No results		251.03714	6.572
Full match	Partial match	No results	Full match		242.08991	2.696

Invalid mass	No results	No results	No match		170.13078	7.148
No results	No results	No results	Full match		141.05398	2.128
No results	No results	No results	No results		266.07874	6.39
No results	No results	No results	No results		399.13133	6.631
No results	No results	No results	No results		886.19862	6.411
No results	No results	No results	No results		183.1155	18.129
No results	No results	No results	Full match		174.01535	1.016
No results	No results	No results	No results		469.13433	6.656
No results	No results	No results	No results		351.2774	14.154
No results	No results	No results	No results		140.10622	18.095
No results	No results	No results	Full match		495.33255	13.7
No results	No results	No results	Full match		210.07309	0.959
No results	No results	No results	No results		333.26692	14.125
No results	No results	No results	No results		427.12668	7.279
No results	No results	No results	No results		511.49668	16.018
No results	Partial match	No results	Full match		290.11133	2.127
No results	No results	No results	No results		295.18612	11.385
No results	No results	No results	No results		377.14727	6.63
No results	No results	No results	No results		515.13993	6.651
No results	No results	No results	No results		152.01097	1.061
No results	No results	No results	No results		110.03544	4.212
No results	No results	No results	Full match		280.09445	7.313
No results	No results	No results	No results		565.3572	14.397
No results	No results	No results	No results		834.52465	18.111
No results	No results	No results	No results		798.54805	18.064
No results	No results	No results	No results		257.23538	14.401
No results	No results	No results	No results		244.0579	3.45
No results	No results	No results	Full match		335.07202	6.398
No results	No results	No results	No results		148.03593	1.435
No results	No results	No results	No results		386.06348	6.312
No results	No results	No results	No results		308.07865	4.549
No results	No results	No results	Full match		204.02608	1.414
No results	No results	No results	No results		528.29318	13.817
No results	Partial match	No results	Full match		190.09542	0.947
No results	No results	No results	No results		353.26466	13.72
No results	No results	No results	Full match		231.074	0.996
No results	No results	No results	Full match		297.10611	0.99
No results	No results	No results	No results		274.17934	6.502
No results	No results	No results	No results		277.06624	7.066
No results	No results	No results	No results		630.10642	0.854
No results	No results	No results	No results		227.97259	0.765
No results	Partial match	No results	Full match		206.09361	6.46
No results	No results	No results	No results		415.20735	11.679
No results	No results	No results	No results		484.27971	10.801
No results	No results	No results	No results		263.95949	2.074
Partial match	No results	No results	Not the top		740.21639	6.645
No results	No results	No results	No results		593.02219	6.151
No results	No results	No results	Full match		334.06652	0.843
No results	No results	No results	No results		936.58127	15.518
Invalid mass	No results	No results	No results		184.03617	6.714

Partial matc	Partial matc	No results	Not the top		302.04239	6.696
No results	Partial matc	No results	Full match		402.09484	6.851
No results	No results	No results	No results		514.09523	2.066
No results	No results	No results	No results		340.20674	18.004
No results	No results	No results	No results		511.4969	16.268
No results	No results	No results	No results		592.26891	17.188
No results	No results	No results	No results		475.2696	13.075
No results	No results	No results	No results		1019.7633	7.212
No results	No results	No results	No results		284.05443	1.42
No results	No results	No results	No results		260.21393	13.719
No results	No results	No results	No results		553.15865	7.027
No results	No results	No results	Full match		476.09503	6.278
No results	No results	No results	No results		777.52983	18.042
No results	No results	No results	No results		248.99486	2.08
Full match	Partial matc	No results	Full match		354.09483	6.153
No results	No results	No results	No results		595.16163	6.724
No results	No results	No results	No results		394.18358	6.587
No results	No results	No results	No results		475.2695	12.883
No results	No results	No results	No results		914.59679	15.913
No results	No results	No results	Full match		366.23111	6.076
No results	No results	No results	Full match		232.14234	1.016
No results	No results	No results	Full match		246.04998	0.918
No results	No results	No results	No results		423.03821	2.077
No results	Partial matc	No results	Full match		347.06281	0.99
No results	Partial matc	No results	Full match		332.07406	1.002
No results	No results	No results	No results		538.33802	14.005
No results	No results	No results	No results		539.23711	6.947
No results	Partial matc	No results	Full match		174.11177	0.865
No results	No results	No results	No results		214.09529	1.008
No results	No results	No results	No results		285.01741	2.153
No results	No results	No results	No results		136.03587	0.944
No results	No results	No results	Full match		291.09514	0.996
No results	No results	No results	No results		203.05722	18.163
No results	No results	No results	No results		467.43399	14.617
No results	No results	No results	No results		370.03555	6.249
No results	No results	No results	No results		246.12139	1.487
No results	No results	No results	No results		182.07796	0.913
No results	No results	No results	No results		312.35059	10.494
No results	Partial matc	No results	Full match		400.07888	7.255
No results	No results	No results	No results		204.00847	2.893
No results	No results	No results	No results		217.09486	0.994
No results	No results	No results	Full match		414.09494	6.852
No results	No results	No results	Full match		426.09493	7.118
No results	No results	No results	Full match		291.09524	1.419
No results	No results	No results	No results		412.03087	1.007
No results	No results	No results	No results		147.96723	18.213
No results	No results	No results	No results		736.48921	18.043
No results	No results	No results	No results		267.00557	2.107
No results	No results	No results	No results		523.36405	15.307
No results	No results	No results	Full match		292.1342	6.93

No results	No results	No results	No results		792.15347	6.397
No results	No results	No results	No results		343.86852	18.161
No results	No results	No results	Full match		127.09989	0.997
No results	No results	No results	No results		405.02788	2.222
No results	No results	No results	No results		444.25434	16.832
No results	No results	No results	No results		262.11641	0.99
No results	No results	No results	No results		295.00055	2.157
No results	No results	No results	No results		345.32443	11.486
Full match	Partial match	No results	Full match		264.13607	8.164
No results	No results	No results	Full match		275.14812	0.994
No results	No results	No results	No results		238.16815	5.935
No results	No results	No results	Full match		202.0954	1.893
No results	No results	No results	No results		392.14705	6.95
No results	No results	No results	No results		238.95941	18.136
No results	No results	No results	No results		950.67813	7.105
Full match	No results	No results	Full match		302.00584	6.867
No results	No results	No results	No results		725.51307	6.904
No results	No results	No results	No results		243.18329	9.117
No results	No results	No results	No results		458.06683	1.041
No results	No results	No results	No results		246.12144	1.016
No results	No results	No results	Full match		174.06809	6.922
No results	No results	No results	No results		427.12669	7.59
No results	No results	No results	No results		183.99981	1.497
No results	No results	No results	No results		265.94145	0.837
No results	No results	No results	No results		258.12145	1.004
No results	No results	No results	No results		129.06075	0.993
No results	No results	No results	No results		312.08442	6.015
No results	No results	No results	No results		298.14159	7.191
No results	No results	No results	No results		387.13181	7.155
No results	No results	No results	No results		434.16869	6.465
No results	No results	No results	No results		239.86503	0.398
No results	Partial match	No results	Full match		400.07914	7.065
No results	No results	No results	No results		351.06682	6.228
No results	No results	No results	Full match		329.111	6.185
No results	No results	No results	No results		177.06272	0.963
No results	No results	No results	No results		279.08206	6.099
No results	No results	No results	Full match		431.12128	6.182
No results	Partial match	No results	Full match		219.11049	3.596
No results	No results	No results	No results		562.22322	7.94
No results	Partial match	No results	Full match		210.03668	0.929
No results	No results	No results	No results		792.18739	6.559
No results	No results	No results	No results		417.10574	6.092
No results	No results	No results	Full match		376.07929	6.158
No results	No results	No results	Full match		161.10522	0.915
No results	No results	No results	Full match		338.10008	6.4
No results	No results	No results	No results		143.05834	2.037
No results	No results	No results	No results		169.01257	2.223
No results	No results	No results	No results		516.33004	14.506
No results	No results	No results	No results		287.05084	6.562
No results	No results	No results	No results		359.13684	6.849

No results	No results	No results	No results		343.22061	6.195
No results	No results	No results	No results		633.32501	13.442
No results	Partial matcl	No results	Full match		324.03553	0.974
No results	No results	No results	No results		583.40953	15.386
No results	Partial matcl	No results	Full match		448.10082	6.397
No results	No results	No results	No results		812.48338	14.55
No results	Partial matcl	No results	Full match		400.07908	7.203
No results	No results	No results	No results		802.29472	6.525
No results	No results	No results	No results		539.52787	14.855
No results	Invalid mass	No results	No results		202.01031	2.059
No results	No results	No results	No results		377.14732	6.502
No results	No results	No results	No results		373.35559	12.789
No results	No results	No results	Full match		248.15271	6.093
No results	No results	No results	No results		746.5095	18.052
No results	No results	No results	No results		810.50477	14.641
No results	No results	No results	No results		130.02531	2.114
No results	No results	No results	No results		458.06673	1.471
No results	No results	No results	No results		296.0092	6.1
No results	No results	No results	No results		303.99881	2.085
No results	No results	No results	No results		308.12893	6.308
No results	No results	No results	Full match		414.0951	7.116
No results	No results	No results	Full match		312.1056	1.077
No results	No results	No results	No results		416.24116	7.501
No results	Partial matcl	No results	Full match		363.05777	1.471
No results	No results	No results	No results		277.06774	6.189
No results	No results	No results	No results		155.13118	1.846
No results	No results	No results	Full match		175.10374	1.424
No results	No results	No results	No results		148.07229	2.568
No results	No results	No results	Full match		222.16118	11.383
No results	No results	No results	No results		193.02932	0.998
No results	Partial matcl	No results	Full match		358.06864	6.66
No results	No results	No results	No results		530.30889	14.569
No results	No results	No results	No results		341.95323	0.767
No results	No results	No results	No results		539.528	15.129
No results	No results	No results	No results		251.97361	2.133
No results	No results	No results	Full match		360.15713	6.93
No results	No results	No results	No results		356.09513	0.913
No results	Partial matcl	No results	Full match		484.08493	6.006
No results	No results	No results	No results		556.23102	7.457
No results	No results	No results	No results		515.13996	6.529
No results	No match	No results	Full match		528.0569	6.365
No results	No results	No results	No results		264.02846	6.365
No results	No results	No results	No results		272.90987	18.174
No results	Partial matcl	No results	Full match		469.15876	6.012
No results	No results	No results	No results		387.13204	6.597
No results	No results	No results	No results		817.50911	18.025
No results	Partial matcl	No results	Full match		404.11032	6.61
No results	No results	No results	No results		307.07736	6.04
No results	No results	No results	No results		361.3192	10.219
No results	No results	No results	No results		183.97847	18.168

No results	No results	No results	No results		620.13758	6.036
No results	No results	No results	No results		413.31413	15.282
No results	No results	No results	No results		207.03229	18.159
No results	No results	No results	Full match		550.29918	7.367
No results	No results	No results	No results		486.07939	6.96
No results	No results	No results	No results		249.07593	6.339
No results	No results	No results	No results		391.94644	2.245
No results	No results	No results	No results		405.14213	6.718
No results	No results	No results	No results		247.97197	18.474
No results	Partial matcl	No results	Full match		363.05779	1.001
No results	No results	No results	No results		172.00299	0.942
No results	Partial matcl	No results	Full match		342.11601	0.933
No results	No results	No results	No results		378.0949	6.254
No results	No results	No results	No results		262.97244	2.225
No results	No results	No results	No results		181.97962	18.295
No results	No results	No results	No results		531.13472	6.371
No results	No results	No results	No results		110.03543	4.768
No results	Partial matcl	No results	Full match		328.09421	6.779
No results	No results	No results	Full match		436.11542	7.412
No results	No results	No results	Full match		580.14457	6.204
No results	No results	No results	No results		232.07293	6.273
No results	No results	No results	No results		274.06864	2.241
No results	No results	No results	No results		530.30907	10.694
No results	No results	No results	No results		333.26691	14.327
No results	No results	No results	No results		117.02806	0.768
No results	No results	No results	No results		576.26962	13.441
Invalid mass	No results	No results	No results		154.02536	3.603
No results	No results	No results	No results		611.42542	6.772
No results	No results	No results	No results		147.0201	0.766
No results	Partial matcl	No results	Full match		326.10005	6.763
No results	No results	No results	Full match		218.14194	6.487
No results	No results	No results	No results		203.13118	18.111
No results	No results	No results	Full match		201.11516	6.481
No results	No results	No results	No results		478.06586	6.768
No results	No results	No results	No results		768.1533	6.356
No results	No results	No results	No results		315.27761	10.996
No results	No results	No results	No results		462.21025	8.082
No results	No results	No results	No results		697.38822	13.8
No results	No results	No results	Full match		436.11526	7.867
No results	No results	No results	No results		290.02157	2.129
No results	No results	No results	No results		362.16998	11.387
No results	No results	No results	No results		400.11137	5.945
No results	No results	No results	Full match		462.07872	6.3
No results	No results	No results	No results		142.95209	18.172
No results	No results	No results	No results		914.59671	16.121
No results	No results	No results	Full match		251.15181	15.187
No results	No results	No results	No results		740.51787	18.052
No results	No results	No results	No results		378.18864	6.855
No results	No results	No results	No results		186.01545	1.125
No results	No results	No results	No results		379.32976	15.686

No results	No results	No results	Full match		135.05322	1.428
No results	No results	No results	No results		189.06269	1.462
No results	No results	No results	Full match		596.17403	6.131
No results	No results	No results	No results		110.00235	0.765
No results	No results	No results	No results		481.39798	16.078
No results	No results	No results	No results		281.8982	18.181
No results	No results	No results	Full match		178.06294	6.177
No results	No results	No results	No results		264.01715	3.926
No results	No results	No results	Full match		313.08756	6.234
No results	No results	No results	No results		256.1309	6.389
No results	No results	No results	No results		342.05012	6.032
No results	No results	No results	Full match		276.0268	6.518
No results	Partial match	No results	Full match		332.07406	1.441
No results	No results	No results	No results		243.08686	0.904
No results	No results	No results	Full match		194.10567	5.356
No results	No results	No results	No results		354.04067	10.61
No results	No results	No results	No results		236.05279	0.974
No results	No results	No results	No results		496.05766	6.394
No results	No results	No results	Full match		186.10046	0.998
No results	No results	No results	No results		258.10613	0.917
No results	No results	No results	No results		1134.5455	7.351
No results	No results	No results	No results		373.15267	6.215
No results	No results	No results	No results		272.05303	5.895
No results	No results	No results	No results		470.21288	8.071
No results	No results	No results	No results		301.11601	0.992
No results	No results	No results	Full match		436.11545	7.777
No results	No results	No results	Full match		342.07363	7.218
No results	No results	No results	Full match		113.08429	1.418
No results	No results	No results	Full match		434.12108	6.584
No results	No results	No results	No results		431.27278	6.385
No results	No results	No results	Full match		342.07364	7.065
No results	No results	No results	No results		109.00155	18.151
No results	No results	No results	No results		206.84604	18.372
No results	No results	No results	No results		263.06374	0.951
No results	No results	No results	No results		437.3717	16.093
No results	No results	No results	No results		1193.5843	8.669
No results	Partial match	No results	Full match		312.06314	6.627
No results	No results	No results	No results		477.28557	14.73
No results	No results	No results	No results		708.15077	6.726
No results	No results	No results	No results		238.09265	2.052
No results	No results	No results	No results		169.98732	0.939
No results	No results	No results	No results		555.30899	13.447
No results	No results	No results	No results		295.07719	6.825
No results	No results	No results	No results		258.12143	1.416
No results	No results	No results	No results		351.06682	6.056
No results	No results	No results	No results		127.06222	1.072
No results	No results	No results	Full match		452.11057	7.416
No results	No results	No results	Full match		289.02208	6.216
No results	Partial match	No results	Full match		432.10519	7.115
No results	No results	No results	Full match		332.09177	6.28

No results	No results	No results	No results		454.12616	6.482
No results	No results	No results	Full match		460.10027	6.69
No results	No results	No results	No results		130.02531	1.716
No results	No results	No results	No results		546.09853	6.638
No results	No results	No results	Full match		130.11084	1.416
No results	No results	No results	No results		602.16326	6.178
No results	No results	No results	No results		316.02186	7.428
No results	No results	No results	No results		225.97879	2.076
No results	No results	No results	No results		1176.556	7.918
No results	No results	No results	Full match		336.08433	6.277
No results	Partial matcl	No results	Full match		187.04811	1.034
No results	No results	No results	No results		206.11086	18.158
No results	No results	No results	Full match		273.08471	1.006
No results	No results	No results	Full match		422.12119	6.09
No results	Partial matcl	No results	Full match		464.0956	6.414
No results	No results	No results	No results		813.48729	18.121
No results	No results	No results	No results		207.03215	18.359
No results	No results	No results	No results		554.51946	10.017
No results	No results	No results	Full match		280.13071	7.225
No results	No results	No results	No results		569.45073	16.058
No results	No results	No results	No results		244.0665	18.145
No results	Partial matcl	No results	Full match		136.05245	6.42
No results	No results	No results	No results		189.02648	0.972
No results	No results	No results	No results		582.30728	18.102
No results	No results	No results	No results		484.2805	10.371
No results	No results	No results	No results		152.01087	1.438
No results	No results	No results	No results		253.1539	1.888
No results	No results	No results	No results		283.10526	1.01
No results	Partial matcl	No results	Full match		400.07946	6.148
No results	No results	No results	No results		204.07776	7.052
No results	No results	No results	No results		582.30684	17.927
No results	No results	No results	No results		282.02767	3.894
No results	No results	No results	No results		411.21026	6.593
No results	No results	No results	No results		716.45148	6.859
No results	No results	No results	Full match		254.18816	9.424
No results	No results	No results	No results		316.10783	0.857
No results	No results	No results	Full match		304.12704	1.421
No results	No results	No results	No results		458.06677	2.535
No results	No results	No results	No results		299.19437	6.071
No results	No results	No results	No results		724.388	13.221
No results	Partial matcl	No results	Full match		636.09628	6.19
No results	No results	No results	No results		338.3663	10.808
No results	No results	No results	No results		486.07959	7.188
No results	No results	No results	No results		224.09107	7.152
No results	No results	No results	Full match		228.14753	5.899
No results	No results	No results	No results		164.03094	0.987
No results	No results	No results	Full match		452.11022	7.31
No results	No results	No results	No results		169.98944	3.401
No results	No results	No results	No results		406.26252	18.11
No results	No results	No results	No results		539.43993	16.673

No results	No results	No results	No results		491.23623	8.083
No results	No results	No results	No results		692.13535	6.215
No results	No results	No results	No results		428.02674	2.054
No results	No results	No results	No results		713.54439	17.232
No results	No results	No results	No results		679.50794	6.898
No results	No results	No results	No results		829.53515	6.999
No results	No results	No results	No match		266.16303	1.898
No results	No results	No results	No results		313.08449	1.417
No results	No results	No results	No results		224.05248	0.958
No results	No results	No results	No results		383.94697	0.276
No results	No results	No results	Full match		202.08423	3.461
No results	No results	No results	No results		219.97676	18.449
No results	No results	No results	No results		157.08552	0.996
No results	No results	No results	No results		304.07919	2.02
No results	No results	No results	No results		140.97621	18.16
No results	No results	No results	No results		244.10579	0.992
No results	No results	No results	Full match		217.05798	0.999
No results	No results	No results	No results		484.27949	18.231
No results	No results	No results	No results		802.29436	6.657
No results	No results	No results	No results		335.30372	15.68
No results	No results	No results	Full match		390.15068	6.948
No results	Partial matcl	No results	Full match		130.02659	0.927
No results	No results	No results	Full match		174.01537	1.429
No results	No results	No results	No results		325.33447	14.909
No results	No results	No results	No results		158.02029	1.13
No results	No results	No results	Full match		171.08957	0.993
No results	No results	No results	No results		200.11618	1.015
No results	No results	No results	No results		151.9889	18.389
No results	No results	No results	No results		232.07293	7.519
No results	No results	No results	Full match		207.12609	18.137
No results	No results	No results	No results		587.40221	18.056
No results	No results	No results	No results		608.26378	16.478
No results	No results	No results	No results		167.03321	4.087
No results	No results	No results	No results		129.00977	0.763
No results	No results	No results	No results		834.28455	6.314
No results	No results	No results	Full match		506.26449	14.659
No results	No results	No results	No results		266.12645	6.63
No results	Partial matcl	No results	Full match		296.08933	6.083
No results	No results	No results	No results		186.01539	1.633
No results	No results	No results	No results		466.07203	6.285
No results	No results	No results	Full match		624.14828	6.359
No results	No results	No results	No results		541.34037	12.344
No results	No results	No results	Full match		226.16841	5.482
No results	Partial matcl	No results	Full match		288.063	8.176
No results	No results	No results	No results		385.06838	7.113
No results	No results	No results	Full match		418.09	7.071
No results	No results	No results	No results		730.15282	6.418
No results	No results	No results	Full match		126.00841	7.871
No results	No results	No results	No results		937.58469	15.291
No results	Partial matcl	No results	Full match		345.0773	2.507

No results	No results	No results	No results		344.04713	3.906
No results	Partial matcl	No results	Full match		147.05308	0.936
No results	No results	No results	No results		147.96722	0.452
No results	No results	No results	No results		245.03187	6.22
No results	No results	No results	No results		294.16537	8.67
No results	No results	No results	No results		631.3094	12.55
No results	No results	No results	Full match		492.08993	6.126
No results	No results	No results	No results		433.13684	6.71
No results	No results	No results	No results		157.08539	1.414
No results	No results	No results	No results		478.28828	13.363
No results	No results	No results	No results		279.98945	3.951
No results	No results	No results	No results		888.52095	14.695
No results	No results	No results	No results		532.08268	6.751
Invalid mass	No results	No results	No results		184.03617	5.767
No results	No results	No results	No results		583.46629	16.634
No results	Partial matcl	No results	Full match		187.04816	1.419
No results	No results	No results	Full match		594.13777	6.005
No results	Partial matcl	No results	Full match		267.09676	0.999
No results	No results	No results	No results		347.03213	1.224
No results	No results	No results	Full match		237.09957	6.23
No results	No results	No results	No results		156.00483	2.206
No results	Partial matcl	No results	Full match		390.09488	6.175
No results	No results	No results	Full match		388.17332	6.28
No results	No results	No results	Full match		330.2406	9.279
No results	No results	No results	Full match		157.1103	2.074
No results	Partial matcl	No results	Full match		292.20379	13.954
No results	No results	No results	No results		262.09528	5.908
No results	No results	No results	No results		574.24124	6.904
No results	No results	No results	No results		130.02533	1.442
No results	No results	No results	Full match		335.07202	6.546
No results	No results	No results	No results		182.12591	18.14
No results	No results	No results	No results		382.03019	1.084
No results	No results	No results	No results		222.02213	18.154
No results	No results	No results	No results		308.07888	6.014
No results	No results	No results	Full match		322.06896	6.111
No results	No results	No results	No results		228.11069	5.806
No results	No results	No results	Full match		314.24583	9.111
No results	Partial matcl	No results	Full match		402.09526	7.108
No results	No results	No results	No results		389.14759	5.985
No results	No results	No results	No results		726.40361	14.199
No results	No results	No results	No results		421.13703	6.084
No results	No results	No results	No results		284.0544	0.982
No results	No results	No results	No results		126.03033	6.397
No results	No results	No results	Full match		566.12473	6.045
No results	No results	No results	No results		270.13275	2.128
No results	No results	No results	No results		406.32962	15.674
No results	No results	No results	Full match		336.08433	6.686
No results	No results	No results	No results		600.32727	13.776
No results	No results	No results	No results		389.11095	6.602
No results	No results	No results	No results		562.09338	6.934

No results	No results	No results	No results		479.30111	14.326
No results	No results	No results	No results		418.05314	6.594
No results	No results	No results	No results		387.13202	6.861
No results	No results	No results	Full match		570.10082	7.427
No results	No results	No results	No results		349.03175	2.13
No results	No results	No results	No results		279.98601	18.155
No results	No results	No results	No results		1060.545	9.422
No results	No results	No results	No results		746.14838	6.254
No results	No results	No results	No results		274.11633	2.764
No results	No results	No results	No results		247.99864	6.65
No results	No results	No results	No results		390.02601	4.545
No results	No results	No results	No results		215.99395	3.322
No results	No results	No results	No results		211.01583	3.244
No results	No results	No results	No results		319.04059	7.366
No results	No results	No results	No results		336.15332	0.993
No results	No results	No results	Full match		344.12605	6.948
No results	No results	No results	Full match		127.0999	1.428
No results	No results	No results	No results		128.01303	0.767
No results	Full match	No results	Full match		214.1318	0.995
No results	No results	No results	No results		267.936	7.07
No results	No results	No results	No results		1151.5734	7.351
No results	Full match	No results	No results		430.34496	17.629
No results	No results	No results	Full match		297.10741	2.117
No results	No results	No results	No results		423.35603	15.677
No results	No results	No results	No results		309.30316	14.956
No results	No results	No results	No results		290.00602	8.449
No results	No results	No results	No results		1193.584	7.923
No results	No results	No results	Full match		490.11072	6.745
No results	No results	No results	No results		539.29888	12.558
No results	No results	No results	No results		557.24761	6.464
No results	No results	No results	No match		302.17298	14.995
No results	No results	No results	Full match		186.10048	1.42
No results	Partial matcl	No results	Full match		101.0478	0.932
No results	No results	No results	No results		674.48825	18.043
No results	Full match	No results	No results		191.07896	0.948
Full match	Partial matcl	No results	Not the top		272.06818	8.889
No results	No results	No results	Full match		145.11045	1.42
No results	No results	No results	No results		151.94032	18.198
No results	No results	No results	No results		676.20018	6.104
No results	No results	No results	No results		268.09992	1.461
No results	No results	No results	No results		451.38753	16.71
No results	No results	No results	No results		1700.7782	15.327
No results	No results	No results	No results		531.30891	14.003
No results	No results	No results	No results		609.32506	14.005
No results	No results	No results	No results		421.13701	6.614
No results	No results	No results	No results		292.05797	6.289
No results	No results	No results	No results		264.15206	18.213
No results	No results	No results	Full match		190.0107	1.008
No results	No results	No results	No results		736.16387	6.805
No results	No results	No results	Full match		128.09515	1.006

No results	No results	No results	No results		217.9764	0.765
No results	No results	No results	No results		1096.8176	17.097
No results	No results	No results	No results		140.00963	1.743
No results	No results	No results	No results		159.05196	1.007
No results	Partial match	No results	Full match		243.08553	1.416
No results	No results	No results	No results		474.31798	6.615
No results	No results	No results	No results		453.13556	6.034
No results	No results	No results	Full match		103.10009	17.964
No results	No results	No results	No results		515.32481	11.685
No results	Partial match	No results	Full match		364.04296	6.227
No results	No results	No results	No results		596.29606	15.455
No results	No results	No results	Full match		452.1104	7.25
No results	No results	No results	No results		228.9673	1.056
No results	No results	No results	No results		183.9998	2.208
No results	No results	No results	Full match		388.13516	7.144
No results	No results	No results	No results		792.49715	6.872
No results	No results	No results	Full match		366.00417	7.839
No results	Partial match	No results	Full match		161.06876	0.956
No results	No results	No results	No results		305.06605	6.02
No results	No results	No results	No results		391.07902	6.172
No results	No results	No results	No results		435.87865	18.168
No results	No results	No results	No results		1035.7646	17.142
No results	No results	No results	No results		905.58146	7.018
No results	No results	No results	No results		495.41371	16.661
No results	No results	No results	No results		267.94054	0.753
No results	No results	No results	No results		511.40877	15.568
No results	No results	No results	No results		790.52205	17.092
No results	No results	No results	No results		449.10377	6.551
No results	No results	No results	No results		156.08906	2.338
No results	No results	No results	No results		875.22745	6.366
No results	Partial match	No results	Full match		216.11017	2.001
No results	No results	No results	No results		274.11639	0.998
No results	Partial match	No results	Full match		404.11055	6.239
No results	No results	No results	Full match		273.08473	1.419
No results	No results	No results	No results		143.05699	1.046
Invalid mass	No results	No results	No results		184.03616	5.925
No results	No results	No results	No results		209.09007	0.938
No results	No results	No results	Full match		235.08417	6.591
No results	No results	No results	No results		278.07888	6.592
No results	No results	No results	No results		281.97016	2.163
No results	No results	No results	Full match		327.08116	0.995
No results	No results	No results	No results		205.11066	18.186
No results	No results	No results	Full match		233.99303	0.435
No results	No results	No results	No results		225.14783	1.529
No results	No results	No results	No results		323.28252	14.392
No results	No results	No results	No results		289.94355	18.153
No results	No results	No results	No results		367.41756	15.205
No results	No results	No results	No results		317.88139	0.112
No results	No results	No results	Full match		402.22546	15.517
No results	No results	No results	No results		817.53714	18.148

No results	No results	No results	No results		435.11579	6.143
No results	No results	No results	No results		445.13683	6.802
No results	No results	No results	No results		334.08991	1.814
No results	No results	No results	Full match		452.11027	7.48
No results	No results	No results	No results		294.11013	12.346
No results	Partial matcl	No results	Full match		216.11008	2.329
No results	Partial matcl	No results	Full match		360.08405	7.102
No results	No results	No results	No results		382.09002	6.819
No results	No results	No results	No results		185.12771	18.15
No results	Partial matcl	No results	Full match		192.06347	0.973
No results	No results	No results	Full match		902.24832	7.436
No results	No results	No results	No results		195.95276	0.921
No results	No results	No results	No results		367.96669	2.227
No results	Partial matcl	No results	Full match		246.05246	7.45
No results	No results	No results	No results		866.51548	18.118
No results	No results	No results	No results		276.08439	0.927
No results	No results	No results	No results		434.24288	18.134
No results	No results	No results	No results		487.29323	12.349
No results	No results	No results	No results		657.21239	3.055
No results	No results	No results	No results		344.0471	4.562
No results	No results	No results	Full match		436.11519	7.482
No results	No results	No results	No results		1243.9258	7.343
No results	No results	No results	No results		419.09307	7.069
No results	Full match	No results	No results		214.01058	2.159
No results	No results	No results	No results		278.8911	4.222
No results	No results	No results	No results		478.13187	2.015
No results	No results	No results	Full match		223.12108	18.108
No results	No results	No results	No results		1177.5595	8.676
No results	No results	No results	No results		450.07738	6.666
No results	No results	No results	No results		460.28576	16.572
No results	No results	No results	No results		722.37206	12.049
No results	No results	No results	No results		271.25106	10.346
No results	No results	No results	No results		553.29347	12.56
No results	No results	No results	No results		153.98458	18.293
No results	No results	No results	No results		336.1533	1.41
No results	No results	No results	No results		723.40411	14.203
No results	No results	No results	No results		202.01391	0.925
No results	No results	No results	Full match		436.11542	7.205
No results	No results	No results	No results		1322.6291	10.373
No results	No results	No results	Full match		424.10049	6.602
No results	No results	No results	No results		249.10002	6.616
No results	Partial matcl	No results	Full match		386.09987	7.331
No results	No results	No results	Full match		222.07346	0.988
No results	No results	No results	No results		127.99602	0.765
No results	No results	No results	Full match		250.13182	2.352
No results	No results	No results	No results		326.05537	6.359
No results	No results	No results	Full match		422.12117	6.295
No results	No results	No results	No results		194.02082	6.359
No results	No results	No results	No results		263.93712	0.753
No results	No results	No results	Full match		468.12721	5.795

No results	No results	No results	No results		332.05805	13.931
No results	No results	No results	No results		433.10886	7.071
No results	Partial matcl	No results	Full match		179.07934	0.937
No results	No results	No results	No results		434.04536	6.019
No results	No results	No results	No results		1052.7918	17.194
No results	No results	No results	No results		283.3239	13.631
No results	No results	No results	No results		436.18567	12.722
No results	No results	No results	Full match		396.19341	12.346
No results	No results	No results	No results		138.03039	6.374
No results	No results	No results	No results		231.88339	18.176
No results	No results	No results	No results		387.14272	6.726
No results	No results	No results	Full match		295.10549	6.643
No results	No results	No results	No results		769.56987	15.298
Invalid mass	No results	No results	No results		154.02539	3.153
No results	No results	No results	No results		453.11884	6.273
No results	No results	No results	No results		418.05091	7.285
No results	No results	No results	No results		156.00822	0.926
No results	No results	No results	No results		481.31635	13.689
No results	No results	No results	No results		386.18587	12.095
No results	No results	No results	No results		613.47746	16.076
No results	No results	No results	No results		415.20735	12.716
No results	No results	No results	No results		556.29115	18.119
No results	No results	No results	No results		406.05318	2.257
No results	No results	No results	No results		600.32734	13.486
No results	No results	No results	No results		622.13209	6.462
No results	No results	No results	Full match		358.06212	1
No results	Partial matcl	No results	Full match		432.10542	6.169
No results	No results	No results	Full match		125.05915	0.959
No results	No results	No results	Full match		656.13753	6.049
No results	No results	No results	Full match		199.12099	2.33
No results	No results	No results	No results		272.10077	1.421
Invalid mass	No results	No results	No results		188.10386	7.409
No results	No results	No results	No results		573.29945	18.132
No results	No results	No results	No results		360.01571	7.072
No results	No results	No results	No results		348.26647	15.169
No results	No results	No results	No results		482.26388	18.011
No results	No results	No results	No results		178.04665	1.058
No results	No results	No results	No results		516.08975	6.338
No results	No results	No results	No results		187.9999	3.279
No results	No results	No results	No results		848.504	18.091
No results	No results	No results	No results		796.54504	14.699
No results	Partial matcl	No results	Full match		432.10549	7.354
No results	No results	No results	No results		333.28786	10.178
No results	No results	No results	No results		1058.5291	8.858
No results	No results	No results	No results		259.25124	10.503
No results	No results	No results	No results		472.06131	6.598
No results	No results	No results	No results		541.33742	13.693
No results	No results	No results	No results		248.06804	7.66
No results	No results	No results	Full match		218.16728	9.395
No results	No results	No results	No results		339.22337	8.68

No results	No results	No results	No results		156.08995	0.998
No results	No results	No results	No results		388.163	6.477
No results	No results	No results	No results		1140.8437	17.029
No results	No results	No results	No results		127.06221	1.437
No results	Full match	No results	Full match		159.17401	18.316
No results	No results	No results	No results		812.47568	18.148
No results	No results	No results	No results		414.25193	18.115
No results	No results	No results	No results		810.1307	6.562
No results	No results	No results	Full match		296.07393	0.98
No results	No results	No results	No results		414.12904	6.947
No results	Partial matcl	No results	Full match		330.14673	7.726
No results	No results	No results	No results		610.45983	17.085
No results	No results	No results	No results		110.03228	18.147
No results	No results	No results	No match		296.23531	9.117
No results	No results	No results	Full match		192.07883	7.274
No results	Partial matcl	No results	Full match		290.11122	1.766
No results	No results	No results	No results		249.09991	6.943
No results	No results	No results	Full match		434.12116	6.134
No results	No results	No results	No results		640.18521	5.793
No results	No results	No results	No results		371.09521	0.906
No results	No results	No results	No results		245.05336	0.974
No results	No results	No results	No results		188.0311	1.102
No results	No results	No results	No results		212.07986	2.074
No results	No results	No results	No results		180.02591	0.936
No results	No results	No results	No results		502.14747	6.299
No results	Partial matcl	No results	Full match		216.1104	1.079
No results	No results	No results	No results		438.11577	6.076
No results	No results	No results	No results		323.10832	7.483
No results	No results	No results	No results		235.99846	2.106
No results	No results	No results	No results		439.14711	6.099
No results	No results	No results	No results		235.14209	0.962
No results	No results	No results	No results		306.16152	5.898
No results	No results	No results	No results		485.15383	5.798
No results	No results	No results	No results		235.06847	0.995
No results	No results	No results	Full match		113.0844	5.963
No results	Partial matcl	No results	Full match		422.08266	0.913
No results	No results	No results	No results		426.25221	18.124
No results	No results	No results	Full match		373.11583	6.446
No results	No results	No results	No results		145.03628	0.972
No results	No results	No results	No results		676.36696	12.054
No results	Partial matcl	No results	Full match		138.0316	6.37
No results	No results	No results	No results		326.05535	6.17
No results	No results	No results	No results		403.21244	12.096
No results	No results	No results	No results		554.31078	11.695
No results	No results	No results	No results		545.32031	13.815
No results	No results	No results	No results		1118.5502	8.866
No results	No results	No results	No results		213.1366	2.148
No results	Partial matcl	No results	Full match		161.04764	6.033
No results	No results	No results	No results		302.03143	3.274
No results	No results	No results	Full match		317.29305	11.46

No results	No results	No results	No results		233.10531	7.275
No results	No results	No results	No results		819.5395	14.641
No results	No results	No results	Full match		282.14631	6.461
No results	Partial matcl	No results	Full match		171.05288	0.996
No results	No results	No results	No results		498.15637	12.341
No results	No results	No results	No results		468.14236	8.97
No results	No results	No results	No results		281.01171	1.906
No results	No results	No results	Full match		169.03754	1.61
No results	No results	No results	No results		418.12216	4.006
No results	No results	No results	No results		600.32728	14.083
No results	No results	No results	No results		229.09497	2.006
No results	No results	No results	No results		445.24619	11.685
No results	No results	No results	No results		194.14199	6.054
No results	No results	No results	No results		350.19387	6.272
No results	No results	No results	No results		313.93663	18.181
No results	No results	No results	No results		378.18864	7.081
No results	No results	No results	No results		405.07478	6.185
No results	No results	No results	No results		1786.8155	15.387
No results	No results	No results	No results		452.15974	12.723
No results	No results	No results	No results		272.1372	1.526
No results	No results	No results	No results		393.34544	16.087
No results	No results	No results	No results		271.9845	2.115
No results	No results	No results	Full match		258.05254	6.142
No results	No results	No results	No results		345.30326	17.645
No results	No results	No results	No results		284.1621	8.938
No results	No results	No results	Full match		255.1106	1.524
No results	No results	No results	No results		291.08215	6.257
No results	No results	No results	No results		620.42846	14.176
No results	No results	No results	No results		303.04592	6.418
No results	No results	No results	No results		1338.624	10.035
No results	No results	No results	No results		1272.9223	16.776
No results	No results	No results	No results		438.09788	7.318
No results	No results	No results	No results		305.0902	2.913
No results	No results	No results	No results		1228.8968	16.862
No results	Partial matcl	No results	Full match		173.06883	1.432
No results	No results	No results	No results		274.11643	2.096
No results	No results	No results	Full match		266.15152	8.937
No results	No results	No results	No results		264.99733	6.123
No results	No results	No results	No results		502.38862	12.057
No results	No results	No results	No results		433.13695	5.991
No results	No results	No results	No results		1184.8701	16.938
No results	No results	No results	No results		262.08422	7.337
No results	No results	No results	No results		114.95707	18.16
No results	No results	No results	Full match		428.11038	6.798
No results	No results	No results	Full match		130.11069	0.998
No results	No results	No results	No results		942.61985	7.078
No results	No results	No results	No results		266.04144	7.077
No results	No results	No results	No results		380.0717	0.931
No results	No results	No results	Full match		342.1469	6.909
No results	No results	No results	No results		586.22617	6.281

No results	No results	No results	Full match		446.08489	7.063
No results	No results	No results	No results		354.36122	10.673
No results	No results	No results	No results		145.07262	2.209
No results	No results	No results	Full match		566.14213	6.676
No results	No results	No results	No results		257.08986	2.771
No results	No results	No results	No results		182.93565	18.253
No results	No results	No results	Full match		390.13124	6.881
No results	No results	No results	No results		111.9713	18.144
No results	No results	No results	No results		354.11602	0.953
No results	No results	No results	No results		161.06757	1.417
No results	No match	No results	No match		294.21922	14.581
No results	No results	No results	Full match		355.34513	13.13
No results	No results	No results	Full match		207.1108	1.097
No results	No results	No results	Full match		452.1103	6.787
No results	No results	No results	No results		396.06862	3.917
No results	No results	No results	No results		330.10649	3.901
No results	No results	No results	No results		168.03855	0.767
No results	Full match	No results	Full match		152.03344	1.416
No results	No results	No results	Full match		171.08964	1.428
No results	No results	No results	No results		280.02159	1.042
No results	No results	No results	Full match		465.09871	6.689
No results	Partial matcl	No results	Full match		238.06834	0.955
No results	No results	No results	No results		714.32274	12.395
No results	No results	No results	Full match		312.13608	6.938
No results	No results	No results	No results		365.11076	6.385
No results	No results	No results	No results		692.36177	9.855
No results	No results	No results	No results		260.10072	1.133
No results	No results	No results	No results		264.02672	7.034
No results	No results	No results	No results		819.58617	14.248
No results	No results	No results	Full match		474.07921	6.689
No results	No results	No results	No results		451.14759	6.13
No results	No results	No results	No results		243.11059	1.641
No results	No results	No results	No results		328.34549	10.355
No results	No results	No results	No results		364.07951	6.125
No results	No results	No results	No results		192.95252	18.276
No results	No results	No results	No results		627.4926	16.587
No results	No results	No results	No results		555.43489	15.553
No results	No results	No results	No results		240.08404	0.917
No results	No results	No results	No results		1008.7648	17.264
No results	No results	No results	No results		373.15673	11.382
No results	No results	No results	No results		254.93151	0.758
No results	No results	No results	Full match		266.16292	1.007
No results	No results	No results	No results		748.52493	18.056
No results	No results	No results	No results		562.33497	14.504
No results	No results	No results	No results		224.15266	3.026
No results	No results	No results	No results		343.05354	3.9
No results	No results	No results	No results		600.32759	13.169
No results	No results	No results	No results		311.93636	18.182
No results	No results	No results	No results		688.14839	2.12
No results	No results	No results	No results		448.158	6.783

No results	No results	No results	No results		407.36112	16.733
No results	No results	No results	No results		282.07364	6.278
No results	No results	No results	Full match		207.08862	6.771
No results	No results	No results	No results		180.00731	18.161
No results	No results	No results	No results		222.03703	0.948
No results	No results	No results	No results		342.02203	0.998
No results	No results	No results	No results		243.98953	2.099
No results	No results	No results	No results		432.02994	7.367
No results	No results	No results	Full match		128.09511	1.425
No results	Full match	No results	No results		228.06271	6.142
No results	No results	No results	No results		175.08323	1.885
No results	No results	No results	No results		152.99787	18.146
No results	No results	No results	No results		200.06948	6.088
No results	No results	No results	No results		459.26186	11.684
No results	Partial matcl	No results	Full match		340.07886	7.06
No results	Partial matcl	No results	Full match		422.08451	6.269
No results	No results	No results	Full match		238.1419	5.819
No results	No results	No results	No results		140.00962	1.056
No results	No results	No results	No results		498.34079	10.295
No results	No results	No results	No results		714.48112	6.908
No results	No results	No results	No results		395.28242	14.721
No results	No results	No results	Full match		474.07918	6.253
No results	No results	No results	No results		312.13189	6.21
No results	No results	No results	Full match		188.01443	2.064
No results	No results	No results	No results		164.04622	1.116
No results	No results	No results	No results		485.15378	5.558
No results	No results	No results	No results		131.98963	0.759
No results	No results	No results	No results		669.46076	17.202
No results	No results	No results	No results		262.11645	1.421
Invalid mass	No results	No results	No match		208.10993	6.67
No results	No results	No results	No results		820.5353	18.001
No results	No results	No results	No results		592.43511	16.9
No results	No results	No results	No results		404.05064	3.901
No results	No results	No results	No results		1614.7412	15.263
No results	No results	No results	No results		324.14183	5.895
No results	No results	No results	No results		287.9449	18.166
No results	Partial matcl	No results	Full match		381.99919	7.911
No results	No results	No results	No results		404.05086	4.554
No results	No results	No results	No results		295.22235	13.43
No results	No results	No results	Full match		338.10013	6.098
No results	Partial matcl	No results	Full match		272.06825	6.763
No results	No results	No results	Full match		322.06863	1.062
No results	No results	No results	No results		130.02531	2.975
No results	No results	No results	No results		532.08259	7.066
No results	No results	No results	No results		204.0084	3.181
No results	No results	No results	No results		598.26683	13.718
No results	No results	No results	No results		141.96947	18.39
No results	No results	No results	Full match		113.0844	2.031
No results	No results	No results	No results		148.03826	0.999
No results	No results	No results	No results		260.21383	12.376

No results	No results	No results	No results		640.18508	1.008
No results	No results	No results	No results		245.97083	0.763
No results	No results	No results	No results		269.13735	6.428
No results	No results	No results	No results		513.06554	6.516
No results	No results	No results	No results		519.25959	12.966
No results	No results	No results	No results		426.07703	6.234
No results	No results	No results	Full match		200.06798	5.996
No results	No results	No results	No results		602.39773	12.642
No results	No results	No results	No results		521.27533	14.151
No results	No results	No results	No results		198.1208	18.124
No results	No results	No results	No results		459.15284	6.516
No results	No results	No results	Full match		306.99745	2.058
No results	No results	No results	No results		354.2771	14.504
No results	Partial matcl	No results	Full match		345.07725	1.434
No results	No results	No results	No results		147.05196	0.908
No results	No results	No results	No results		422.21493	7.536
No results	No results	No results	No results		189.1156	18.149
No results	No results	No results	No results		405.15346	6.489
No results	No results	No results	No results		612.47606	18.108
No results	No results	No results	No results		397.22539	15.893
No results	No results	No results	No results		447.15289	6.638
No results	Full match	No results	Full match		214.13179	1.83
No results	No results	No results	No results		266.07874	5.827
No results	No results	No results	No results		261.02701	6.805
No results	No results	No results	No results		618.15809	6.014
No results	No results	No results	No results		498.137	6.017
No results	No results	No results	No results		266.079	7.27
No results	No results	No results	Full match		503.0776	6.592
No results	No results	No results	Full match		468.10503	7.001
No results	Partial matcl	No results	Full match		654.38275	13.424
No results	No results	No results	No results		407.15764	6.342
No results	No results	No results	No results		657.50287	15.979
No results	No results	No results	Full match		111.06859	1.417
No results	No results	No results	Full match		165.06514	2.095
No results	No results	No results	No results		148.03856	2.101
No results	Full match	No results	No results		284.08946	5.693
No results	No results	No results	Full match		191.13129	0.994
No results	No results	No results	Full match		452.13153	2.973
No results	No results	No results	No results		378.1312	7.561
No results	No results	No results	No results		637.16459	6.047
No results	No results	No results	Full match		149.0702	1.435
No results	No results	No results	No results		760.31883	12.381
No results	No results	No results	No results		530.165	6.543
No results	No results	No results	No results		468.14236	9.464
No results	No results	No results	No results		338.12139	0.997
No results	No results	No results	No results		888.52107	18.151
No results	No results	No results	No results		579.1459	6.06
No results	No results	No results	No results		204.07776	6.828
No results	No results	No results	No results		1077.5726	9.422
No results	No results	No results	No results		484.13729	8.395

No results	No results	No results	Full match		414.095	7.279
No results	No results	No results	No results		584.32271	14.562
No results	No results	No results	No results		162.08805	5.633
No results	No results	No results	Full match		530.17871	7.818
No results	No results	No results	No results		375.13191	5.998
No results	No results	No results	No results		1176.556	7.715
No results	No results	No results	No results		418.12217	4.311
No results	No results	No results	No results		188.08368	6.924
No results	No results	No results	No results		362.23603	6.147
No results	No results	No results	Full match		221.105	6.056
No results	No results	No results	No results		396.24171	18.128
No results	No results	No results	No results		359.13701	6.191
No results	No results	No results	No results		274.0686	1.034
No results	No results	No results	No results		271.2148	9.375
No results	Partial match	No results	Full match		296.08938	6.84
No results	No results	No results	No results		238.01072	6.018
No results	No results	No results	Full match		318.20437	15.741
No results	No results	No results	No results		181.11812	6.157
No results	No results	No results	Full match		376.11726	6.74
No results	No results	No results	Full match		468.10485	6.711
No results	No results	No results	No results		604.12141	7.555
No results	No results	No results	No results		126.97218	1.095
No results	No results	No results	No results		557.11445	6.867
No results	No results	No results	No results		280.14227	6.065
No results	No results	No results	No results		433.13681	6.457
No results	No results	No results	Full match		356.07388	6.412
No results	No results	No results	No results		315.15807	6.658
No results	No results	No results	No results		139.02568	1.423
No results	No results	No results	Full match		125.05882	1.424
No results	No results	No results	No results		248.10243	6.638
No results	No results	No results	Full match		234.13694	5.457
No results	No results	No results	No results		467.38229	15.658
No results	No results	No results	No results		444.15003	1.003
No results	No results	No results	No results		259.05594	6.149
No results	No results	No results	No results		286.11647	2.101
No results	No results	No results	No results		175.1209	1.837
No results	No results	No results	No results		103.9961	0.767
No results	No match	No results	No match		166.04802	2.229
No results	No results	No results	No results		326.11142	5.397
No results	Partial match	No results	Full match		340.07914	5.778
No results	No results	No results	No results		413.03422	2.824
No results	No results	No results	Full match		363.06718	6.256
No results	No results	No results	No results		167.03325	3.899
No results	No results	No results	No results		1134.5453	7.665
No results	No results	No results	Full match		250.08402	4.447
No results	No results	No results	Full match		330.27707	13.802
Invalid mass	No results	No results	No results		175.96226	1.29
No results	No results	No results	No results		468.14286	7.103
No results	No results	No results	No results		552.29152	10.732
No results	No results	No results	No results		266.15492	15.201

No results	No results	No results	Full match		482.10561	5.749
No results	No results	No results	No results		469.14476	8.973
No results	No results	No results	No results		435.12498	6.652
No results	No results	No results	Full match		470.08281	6.869
No results	No results	No results	No results		385.1638	6.481
No results	No results	No results	No results		295.07721	6.182
No results	No results	No results	Full match		118.07852	11.684
No results	No results	No results	Full match		221.1416	18.086
No results	No results	No results	No results		199.12833	18.118
No results	No results	No results	No results		259.09904	0.988
No results	Partial matcl	No results	Full match		402.09471	6.661
No results	No results	No results	Full match		416.11141	6.09
No results	No results	No results	Full match		500.13185	7.409
No results	No results	No results	No results		478.28845	14.616
No results	No results	No results	No results		305.08991	1.486
No results	No results	No results	Full match		430.08944	6.81
No results	No results	No results	No results		282.0948	0.996
No results	No results	No results	Full match		348.08443	6.391
No results	No results	No results	Full match		262.13182	6.07
No results	No results	No results	No results		405.07486	6.359
No results	No results	No results	No results		260.10083	1.613
No results	No results	No results	No results		205.05805	0.937
No results	No results	No results	No results		603.28496	13.442
No results	No results	No results	No results		177.06374	0.935
No results	No results	No results	No results		370.29135	15.282
No results	No results	No results	No results		402.0579	5.97
Invalid mass	No results	No results	No results		138.03043	7.861
No results	No results	No results	No results		552.0904	7.408
No results	No results	No results	No results		189.09904	1.89
No results	No results	No results	No results		478.13163	2.488
No results	No results	No results	No results		389.11082	6.054
No results	No results	No results	No results		323.07197	6.109
No results	No results	No results	No results		1322.6292	10.945
No results	No results	No results	No results		545.35639	13.719
No results	No results	No results	No results		580.29139	16.46
No results	No results	No results	No results		266.15486	14.8
No results	Partial matcl	No results	Full match		278.22444	9.121
No results	No results	No results	No results		222.01576	7.276
No results	No results	No results	No results		395.96943	4.548
No results	No results	No results	Full match		346.0315	1.446
No results	No results	No results	No results		442.28373	18.099
No results	No results	No results	No results		413.03414	2.6
No results	No results	No results	Full match		460.10014	4.622
No results	No results	No results	No results		359.13684	7.088
No results	No results	No results	Full match		656.429	16.316
No results	No results	No results	No results		514.05733	6.504
No results	No results	No results	Full match		346.10519	6.612
No results	No results	No results	No results		805.55843	18.054
No results	Full match	No results	Full match		244.08823	2.706
Partial matcl	Partial matcl	No results	Not the top		302.04228	7.417

No results	No results	No results	No results		287.0508	6.921
No results	No results	No results	Full match		602.12712	6.225
No results	No results	No results	No results		557.11427	7.181
No results	No results	No results	No results		285.1576	2.275
No results	No results	No results	No results		417.10588	6.031
No results	No results	No results	No results		237.11139	6.029
No results	No results	No results	No results		484.13727	8.639
Full match	Partial matcl	No results	Full match		354.0948	5.83
No results	No results	No results	No results		226.98828	3.347
No results	No results	No results	No results		265.08108	0.995
No results	No results	No results	No results		242.07854	5.983
No results	No results	No results	Full match		198.0755	4.076
No results	No results	No results	Full match		356.07417	6.054
No results	No results	No results	No results		442.05107	1.019
No results	No results	No results	No results		749.55576	14.703
No results	No results	No results	No results		248.13674	18.174
No results	No results	No results	No results		1872.8509	15.446
No results	No results	No results	No results		292.05815	7.218
No results	No results	No results	No results		443.99929	2.072
No results	No results	No results	No results		140.00963	1.493
No results	No results	No results	Full match		594.13792	7.809
No results	No results	No results	No results		446.14248	6.385
No results	No results	No results	No results		221.97249	18.443
No results	No results	No results	No results		307.99425	2.219
No results	No results	No results	No results		530.10563	6.512
No results	No results	No results	No results		412.1398	6.77
No results	No results	No results	Full match		537.51258	16.361
No results	No results	No results	No results		473.98515	4.56
No results	No results	No results	No results		308.07933	3.93
No results	No results	No results	No results		484.1375	8.724
No results	No results	No results	No results		313.08438	0.999
No results	No results	No results	Full match		604.14283	6.29
No results	No results	No results	No results		492.28578	11.827
No results	No results	No results	No results		422.0717	3.911
No results	No results	No results	Full match		584.15274	6.166
No results	No results	No results	No results		325.11595	6.265
No results	No results	No results	No results		308.97978	0.765
No results	Partial matcl	No results	Full match		163.08444	0.936
No results	No results	No results	No results		692.36165	9.726
No results	No results	No results	No results		438.11578	5.896
No results	No results	No results	Full match		512.21606	6.632
No results	No results	No results	Full match		173.11656	1.509
No results	No results	No results	No results		307.28755	16.937
No results	Partial matcl	No results	Full match		276.13178	1.416
No results	No results	No results	Full match		408.12047	8.098
No results	No results	No results	No results		511.29869	12.926
No results	No results	No results	No results		685.37094	5.618
No results	No results	No results	No results		478.28837	14.814
No results	No results	No results	No results		488.09493	6.56
No results	No results	No results	Full match		566.14217	7.31

No results	Partial matcl	No results	Full match		214.04751	1.059
No results	No results	No results	Full match		311.31871	14.797
No results	No results	No results	No results		1174.5765	9.903
No results	Full match	No results	Full match		222.05249	6.953
No results	No results	No results	Full match		218.12657	1.423
No results	No results	No results	No results		584.38698	12.616
No results	No results	No results	No results		547.33597	14.568
No results	No results	No results	No results		177.07916	3.074
No results	No results	No results	No results		484.1375	8.467
No results	No results	No results	No results		1316.9484	16.705
No results	No results	No results	No results		762.14196	6.082
No results	No results	No results	No results		423.99501	2.225
No results	Partial matcl	No results	Full match		400.07969	7.113
No results	No results	No results	Full match		295.10593	6.014
No results	Full match	No results	No results		321.12294	6.172
No results	No results	No results	Full match		636.20285	6.936
No results	No results	No results	No results		498.34169	6.604
No results	No results	No results	No results		472.06358	6.588
No results	No results	No results	Full match		324.22992	13.806
No results	No results	No results	No results		1193.584	7.717
No results	No results	No results	No results		560.32238	12.55
No results	No results	No results	No results		456.06639	7.126
No results	No results	No results	No results		326.11144	5.661
No results	No results	No results	Full match		305.23545	13.672
No results	No results	No results	No results		468.43733	14.599
No results	No results	No results	No results		328.22528	8.899
No results	No results	No results	Full match		290.11822	7.499
No results	No results	No results	No results		321.05621	6.026
No results	No results	No results	No results		797.55683	15.906
No results	No results	No results	Full match		177.11559	8.577
No results	No results	No results	No results		197.1312	18.125
No results	No results	No results	No results		510.29595	12.266
No results	No results	No results	No results		120.07716	2.802
No results	No results	No results	Full match		420.10567	7.045
No results	No results	No results	Full match		462.24625	7.5
No results	No results	No results	No results		1338.6241	10.558
No results	No results	No results	No results		580.29152	16.752
No results	Partial matcl	No results	Full match		381.99916	7.988
No results	Partial matcl	No results	Full match		324.03552	1.418
No results	No results	No results	No results		908.22247	6.602
No results	No results	No results	Full match		274.04406	2.087
No results	No results	No results	No results		218.01543	0.766
No results	No results	No results	No results		505.31662	13.443
No results	No results	No results	Full match		297.12138	5.898
No results	No results	No results	No results		182.10571	6.033
No results	No results	No results	Full match		128.05872	0.922
No results	No results	No results	No results		511.29881	11.802
No results	No results	No results	No results		440.23855	7.635
No results	No results	No results	Full match		328.10029	0.941
No results	No results	No results	No results		1135.5784	8.869

No results	No results	No results	No results		396.06853	4.557
No results	No results	No results	No results		286.09546	6.024
No results	No results	No results	No results		457.10122	5.87
Full match	No results	No results	Full match		294.18284	12.361
No results	No results	No results	No results		506.26425	15.158
No results	No results	No results	Full match		474.07919	6.814
No results	No results	No results	No results		223.07234	1.01
No results	No results	No results	Full match		490.11033	6.954
No results	No results	No results	No results		369.06907	3.908
No results	No results	No results	No results		403.09807	7.61
No results	No results	No results	Full match		198.01551	6.011
No results	No results	No results	No results		483.1378	6.071
No results	No results	No results	No results		705.20576	6.274
No results	No results	No results	No results		255.07413	1.423
No results	No results	No results	No results		264.0266	6.371
No results	No results	No results	No results		193.09651	1.929
No results	No results	No results	No results		337.25029	14.499
No results	No results	No results	No results		155.00683	0.761
No results	No results	No results	No results		535.2909	14.185
No results	No results	No results	No results		506.30884	14.992
No results	No results	No results	No results		354.022	1.489
No results	No results	No results	No results		615.45616	12.182
No results	Partial match	No results	Full match		402.09526	7.823
No results	No results	No results	Full match		252.09922	6.095
No results	No results	No results	No results		162.0156	0.954
No results	No results	No results	No results		265.05848	3.403
No results	No results	No results	No results		1131.8452	7.2
No results	Partial match	No results	Full match		290.11115	1.063
No results	No results	No results	No results		513.06603	6.177
No results	No results	No results	No results		667.15162	6.891
No results	No results	No results	No results		322.10547	7.484
No results	No results	No results	Full match		192.15157	10.313
No results	No results	No results	No results		569.1649	6.279
No results	No results	No results	No match		288.2298	9.65
No results	Partial match	No results	Full match		161.06883	1.419
No results	No results	No results	No results		398.25681	18.126
No results	No results	No results	No results		385.04789	4.553
No results	No results	No results	No results		255.14702	2.094
No results	No results	No results	No results		274.83424	5.246
No results	No results	No results	No results		163.76482	18.154
No results	No results	No results	No results		500.09516	6.683
No results	No results	No results	No results		1151.5732	7.663
No results	No results	No results	No results		292.05815	8.083
No results	No results	No results	No results		648.11126	5.994
No results	Partial match	No results	Full match		127.06356	1.602
No results	No results	No results	No results		254.12656	6.157
No results	No results	No results	No results		191.11311	18.172
No results	No results	No results	No results		231.11073	2.047
No results	No results	No results	No results		1194.5879	7.898
No results	No results	No results	No results		128.972	18.156

No results	No results	No results	No results		506.30883	14.789
No results	Full match	No results	Full match		131.05833	0.93
No results	No results	No results	No results		334.23559	16.46
No results	No results	No results	No results		396.08012	6.054
No results	No results	No results	No results		289.26178	8.752
No results	No results	No results	No results		239.07939	1.528
No results	No results	No results	No results		454.34487	15.86
No results	No results	No results	No results		793.57057	17.823
No results	Partial match	No results	Full match		102.03177	0.931
No results	No results	No results	Full match		303.03755	7.062
No results	No results	No results	Full match		185.11551	18.171
No results	No results	No results	No results		206.84608	0.387
No results	No results	No results	No results		457.34046	15.278
No results	No results	No results	No results		377.14728	6.859
No results	Full match	No results	Full match		214.13181	1.424
No results	No results	No results	No results		510.29604	12.639
No results	No results	No results	No results		236.03179	6.213
No results	No results	No results	No results		546.09838	6.255
No results	No results	No results	No results		287.05077	8.988
No results	No results	No results	Full match		195.1261	18.097
No results	No results	No results	No results		259.91698	1.805
No results	No results	No results	No results		278.82814	0.33
No results	No results	No results	No results		341.36588	14.234
No results	No results	No results	No results		1134.5454	8.3
No results	No results	No results	No results		246.08487	1.072
No results	No results	No results	No results		132.05775	18.172
No results	No results	No results	No results		277.06636	5.92
No results	No results	No results	Full match		590.12688	6.267
No results	No results	No results	No results		155.13116	1.057
No results	No results	No results	No results		204.0529	18.019
No results	No results	No results	No results		608.2638	16.247
No results	No results	No results	No results		321.26675	12.879
No results	No results	No results	No results		321.26676	13.086
No results	No results	No results	No results		268.05762	6.3
No results	No results	No results	No results		172.01247	0.917
No results	No results	No results	Full match		374.06329	6.889
No results	No results	No results	No results		652.17487	17.084
No results	No results	No results	Full match		534.13714	6.602
No results	Partial match	No results	Full match		214.04684	2.132
No results	No results	No results	No results		840.50569	18.015
No results	No results	No results	No results		421.39188	14.918
No results	No results	No results	No results		416.03514	8.164
No results	No results	No results	No results		286.88131	0.091
No results	Partial match	No results	Full match		189.06374	1.456
No results	No results	No results	No results		483.46523	15.171
No results	No results	No results	No results		818.51143	17.959
No results	No results	No results	No results		110.0049	0.768
No results	No results	No results	No results		202.96912	18.404
No results	No results	No results	Full match		264.0995	6.854
No results	No results	No results	Full match		129.09032	1.391

No results	No results	No results	No results		616.14152	5.974
No results	No results	No results	No results		577.30921	13.719
No results	No results	No results	No results		550.10847	7.785
No results	No results	No results	No results		492.28541	12.905
No results	No results	No results	Full match		428.23311	0.855
No results	No results	No results	No results		738.36736	9.75
No results	No results	No results	No results		130.52651	18.145
No results	Partial match	No results	Full match		300.08464	6.023
No results	No results	No results	No results		459.26128	12.738
No results	No results	No results	No results		248.10075	0.946
No results	No results	No results	No results		1160.5609	9.484
No results	No results	No results	No results		395.9692	3.904
No results	No results	No results	No results		550.10859	7.433
No results	Partial match	No results	Full match		323.05143	0.968
No results	No results	No results	No results		484.13727	8.81
No results	No results	No results	No results		394.08968	6.039
No results	No results	No results	No results		406.17374	6.666
No results	No results	No results	No results		673.4554	16.327
No results	Partial match	No results	Full match		219.11099	3.406
No results	Partial match	No results	Full match		310.21455	11.937
No results	No results	No results	No results		382.01156	2.142
No results	Full match	No results	No results		150.03234	4.55
No results	No results	No results	Full match		624.20521	6.613
No results	No results	No results	No results		265.05855	3.655
No results	No results	No results	No results		452.093	6.102
No results	No results	No results	Full match		144.09011	1.597
No results	No results	No results	Full match		418.12652	7.542
No results	No results	No results	Full match		440.11235	7.119
No results	No results	No results	No results		557.27911	13.448
No results	No results	No results	Full match		798.22198	6.678
No results	No results	No results	No results		575.21593	14.579
No results	No results	No results	No results		311.12285	3.959
No results	No results	No results	No results		1063.7626	7.207
No results	No results	No results	No results		673.49797	13.119
No results	No results	No results	No results		769.5645	14.805
No results	No results	No results	No results		260.13707	1.645
No results	No results	No results	No results		312.19355	10.911
No results	No results	No results	No results		207.11221	18.182
No results	No results	No results	No results		239.13841	18.159
No results	No results	No results	Full match		222.11147	18.169
No results	No results	No results	No results		474.15816	0.931
No results	No results	No results	Full match		226.12036	6.664
No results	No results	No results	Full match		450.13178	9.166
No results	No results	No results	No results		612.14413	1.862
No results	No results	No results	Full match		624.20519	6.752
No results	No results	No results	No results		183.01583	1.027
No results	No results	No results	No results		511.29872	12.275
No results	No results	No results	No results		253.01195	0.888
No results	No results	No results	No results		644.10176	6.286
No results	No results	No results	No results		484.09993	6.599

No results	No results	No results	No results		964.73897	17.36
No results	No results	No results	Full match		141.07905	1.003
No results	Partial matcl	No results	Full match		330.14662	6.458
No results	No results	No results	No results		244.10578	1.417
No results	No results	No results	No results		484.13747	8.878
No results	No results	No results	No results		305.17842	5.479
No results	No results	No results	No results		176.96559	18.189
No results	No results	No results	Full match		422.12114	5.84
No results	No results	No results	Full match		440.07368	7.065
No results	No results	No results	No results		438.11564	3.398
No results	No results	No results	Full match		303.25624	13.048
No results	No results	No results	Full match		594.13789	8.012
No results	No results	No results	No results		285.95104	0.762
No results	No results	No results	No results		308.19844	10.51
No results	No results	No results	No results		236.06811	5.957
No results	No results	No results	No results		261.05285	18.163
No results	No results	No results	No results		265.08098	1.426
No results	No results	No results	Full match		185.10526	0.997
No results	No results	No results	Full match		434.12082	7.087
No results	No results	No results	Full match		312.0996	8.273
No results	No results	No results	No results		278.18587	8.767
No results	No results	No results	No results		244.05789	2.332
No results	No results	No results	No results		1022.2327	6.341
No results	No results	No results	Full match		608.15311	6.749
No results	No results	No results	No results		394.04783	4.547
No results	No results	No results	No results		292.08421	6.153
No results	Full match	No results	Full match		160.0524	6.933
No results	No results	No results	No results		424.14057	11.383
No results	No results	No results	No results		575.2162	14.808
No results	No results	No results	Full match		362.10071	6.485
No results	No results	No results	No results		496.27272	18.166
No results	No results	No results	No results		614.41876	15.863
No results	No results	No results	No results		115.99452	0.755
No results	No results	No results	No results		295.89211	18.175
No results	No results	No results	No results		425.92214	18.166
No results	No results	No results	No results		359.13684	6.052
No results	No results	No results	No results		567.42076	16.434
No results	No results	No results	No results		228.11126	2.164
No results	No results	No results	No results		437.03453	3.903
No results	No results	No results	No results		211.78324	18.16
No results	No results	No results	No results		477.33017	10.119
No results	No results	No results	No results		409.1138	1.845
No results	No results	No results	No results		511.14797	7.419
No results	No results	No results	Full match		414.20398	12.981
No results	No results	No results	Full match		205.14687	5.894
No results	No results	No results	No results		595.14039	6.029
No results	No results	No results	No results		373.33452	16.449
No results	No results	No results	Full match		562.16891	6.124
No results	Partial matcl	No results	Full match		338.06367	6.185
No results	No results	No results	No results		154.02539	6.212

No results	No results	No results	No results		408.14165	7.263
No results	No results	No results	No results		366.04313	1.057
No results	No results	No results	No results		411.85599	18.165
No results	No results	No results	No results		530.30887	10.588
No results	No results	No results	No results		428.02651	2.502
No results	No results	No results	No results		637.17958	14.351
No results	Partial match	No results	Full match		366.05836	6.28
No results	No results	No results	No results		180.04112	6.367
No results	No results	No results	No results		139.95443	0.777
No results	Partial match	No results	Full match		322.05501	0.957
No results	No results	No results	Full match		236.15254	2.374
No results	No results	No results	Full match		219.12595	2.417
No results	No results	No results	No results		676.20015	6.406
No results	No results	No results	No results		481.31642	14.006
No results	No results	No results	No results		594.28022	12.901
No results	No results	No results	No results		142.93795	18.175
No results	No results	No results	No results		255.14699	1.654
No results	No results	No results	No results		837.52351	18.141
No results	No results	No results	No results		122.03544	6.662
No results	No results	No results	No results		189.09904	1.158
No results	No results	No results	No results		770.16834	6.436
No results	No results	No results	No results		285.13239	2.383
No results	No results	No results	Full match		373.11525	6.28
No results	Partial match	No results	Full match		219.11067	1.014
No results	No results	No results	No results		479.3007	18.089
No results	No results	No results	No results		467.38239	15.523
No results	No results	No results	No results		474.17328	7.244
No results	No results	No results	Full match		223.08467	2.054
No results	No results	No results	No results		701.48982	18.072
No results	No results	No results	No results		156.98497	18.164
No results	No results	No results	Full match		217.9877	3.894
No results	Partial match	No results	Full match		330.07367	6.916
No results	No results	No results	No results		630.13689	6.688
No results	No results	No results	No results		396.74712	18.155
No results	No results	No results	No results		671.51857	16.601
No results	Partial match	No results	Full match		164.0471	6.879
No results	Partial match	No results	Full match		374.09991	7.02
No results	No results	No results	Full match		308.14188	6.83
No results	No results	No results	No results		511.4088	15.668
No match	No match	No results	No match		270.05259	8.833
No results	No results	No results	Full match		217.05783	1.417
No results	No results	No results	Full match		208.14547	11.476
No results	Partial match	No results	Full match		224.07981	2.115
Invalid mass	No results	No results	No match		310.1054	6.81
No results	No results	No results	No results		457.24612	12.347
No results	Full match	No results	Full match		226.0947	3.348
No results	No results	No results	Full match		415.12645	6.705
No results	No results	No results	No results		515.32467	12.73
No results	No results	No results	Full match		228.1474	3.992
No results	No results	No results	No results		1360.9749	16.676

No results	No results	No results	No results		794.15872	6.566
No results	No results	No results	No results		354.04067	8.971
No results	No results	No results	No results		440.40599	14.031
No results	No results	No results	No results		188.0311	1.592
No results	No results	No results	No results		1009.162	6.268
No results	No results	No results	Full match		172.08489	0.955
No results	No results	No results	No results		266.13787	2.106
No results	No match	No results	No match		234.16135	14.414
No results	Full match	No results	No results		208.03666	6.326
No results	No results	No results	No results		114.95052	18.326
No results	No results	No results	No results		325.33458	15.174
No results	No results	No results	No results		194.07777	0.982
No results	No results	No results	No results		866.20613	6.294
No results	No results	No results	No results		221.06807	6.219
No results	No results	No results	No results		303.04573	7.415
No results	No results	No results	No results		493.31704	12.842
No results	No results	No results	No results		553.17002	6.512
No results	No results	No results	No results		234.08865	6.11
No results	No results	No results	No results		526.42744	16.051
No results	No results	No results	No results		230.16313	6.002
No results	Partial matcl	No results	Full match		338.06364	0.965
No results	No results	No results	Full match		250.08394	4.715
No results	No results	No results	No results		298.04493	7.085
No results	No results	No results	No results		303.04558	8.14
No results	No results	No results	No results		372.12648	0.954
No results	No results	No results	No results		237.03462	4.475
No results	No results	No results	No results		596.29571	14.291
No results	No results	No results	Full match		330.24065	8.74
No results	No results	No results	No results		508.21483	8.075
Full match	Partial matcl	No results	Not the top		274.08393	9.238
No results	No results	No results	No results		312.06881	0.92
No results	Full match	No results	No results		214.01054	2.476
No results	No results	No results	No results		192.0412	1.751
No results	No results	No results	No results		301.2618	10.86
No results	No results	No results	No results		312.17567	16.998
No results	No results	No results	No results		469.14476	9.465
No results	No results	No results	Full match		96.0213	1.435
No results	No results	No results	Full match		406.03145	2.063
No results	No results	No results	No results		433.137	6.648
No results	No results	No results	No results		538.22079	6.909
No results	No results	No results	No results		598.31185	18.119
No results	No results	No results	No results		336.08643	6.269
No results	No results	No results	No results		255.14436	18.152
No results	No results	No results	No results		776.4834	18.053
No results	No results	No results	No results		854.49288	18.055
No results	No results	No results	Full match		125.02371	5.984
No results	No results	No results	Full match		171.08968	2.096
No results	No results	No results	No results		179.11213	5.856
No results	No results	No results	No results		749.5281	18.054
No results	No results	No results	No results		955.61308	15.271

No results	No results	No results	Full match		722.1475	6.239
No results	Partial matcl	No results	Full match		276.132	2.059
No results	No results	No results	No results		373.90837	18.162
No results	No results	No results	Full match		295.10539	6.221
No results	No results	No results	Full match		600.11143	6.722
No results	No results	No results	Full match		205.14665	18.109
No results	No results	No results	No results		594.2801	12.703
No results	Partial matcl	No results	Full match		344.08923	7.548
No results	No results	No results	No results		286.11628	2.689
No results	No results	No results	No results		363.95122	18.157
No results	No results	No results	Full match		397.99385	8.04
No results	No results	No results	No results		485.13983	8.409
No results	No results	No results	No results		492.28529	12.255
No results	No results	No results	Full match		234.07305	3.846
No results	No results	No results	No results		188.05044	1.42
No results	No results	No results	No results		438.24629	8.826
No results	No results	No results	No results		596.29583	14.077
No results	No results	No results	No results		327.13177	6.034
No results	No results	No results	No results		280.02159	1.748
No results	No results	No results	No results		140.01081	1.735
No results	No results	No results	No results		254.04234	6.063
No results	No results	No results	Full match		520.15784	6.908
No results	No results	No results	No results		437.12359	6.616
No results	No results	No results	No results		654.08544	6.508
No results	Partial matcl	No results	Full match		272.06827	7.325
No results	No results	No results	No results		362.06136	6.817
No results	No results	No results	No results		343.14217	8.009
No results	No results	No results	No results		248.15472	18.162
No results	No results	No results	No results		798.52593	17.93
No results	No results	No results	No results		197.11312	18.115
No results	No results	No results	No results		237.03485	4.024
No results	No results	No results	No results		932.58774	7.155
No results	No results	No results	Full match		297.30315	17.947
No results	No results	No results	No results		533.31202	9.863
No results	No results	No results	Full match		422.09983	7.502
No results	No results	No results	Full match		252.06284	6.289
No results	No results	No results	Full match		450.07927	6.262
No results	No results	No results	No results		438.11572	5.508
No results	No results	No results	Full match		594.13775	1.053
No results	Partial matcl	No results	Full match		918.24334	7.221
No results	No results	No results	No results		434.17897	6.714
No results	No results	No results	No results		759.50078	16.013
No results	No results	No results	No results		340.02486	10.093
No results	No results	No results	No results		361.15255	7.359
No results	No results	No results	Full match		237.0996	6.373
No results	No results	No results	No results		714.52057	18.046
No results	No results	No results	No results		736.16261	6.261
No results	No results	No results	No results		172.12134	2.873
No results	No results	No results	No results		202.13185	2.038
Invalid mass	No results	No results	No match		207.08958	6.084

No results	No results	No results	Full match		546.13749	6.566
No results	No results	No results	No results		277.8883	18.174
No results	No results	No results	No results		242.20328	13.722
No results	Full match	No results	Full match		152.03347	0.994
No results	No results	No results	No results		384.37139	14.108
No results	No results	No results	No results		512.11492	0.932
No results	No results	No results	No results		739.36197	12.373
No results	No results	No results	No results		304.00681	6.033
No results	No results	No results	No results		375.25189	12.055
No results	No results	No results	No results		293.14728	1.615
No results	No results	No results	No results		438.11589	1.036
No results	No results	No results	No results		601.26944	12.554
No results	No results	No results	No results		187.08342	1.845
No results	No results	No results	No results		248.99491	1.209
No results	No match	No results	Full match		348.0479	6.217
No results	No results	No results	No results		188.14416	5.783
No results	No results	No results	No results		599.46126	15.464
No results	No results	No results	No results		364.07952	5.926
No results	No results	No results	No results		223.11826	18.159
No results	No results	No results	No results		582.11959	5.764
No results	No results	No results	No results		163.05962	18.153
No results	No results	No results	No results		220.01609	3.296
No results	No results	No results	No results		535.49712	15.427
No results	No results	No results	No results		1528.7043	15.189
No results	No results	No results	Full match		237.0862	2.016
No results	No results	No results	Full match		276.09571	1.033
No results	No results	No results	No results		697.49248	17.198
No results	No results	No results	No results		288.19369	6.82
No results	No results	No results	Full match		235.08451	2.117
No results	No results	No results	No results		357.13052	6.36
No results	No results	No results	No results		212.11613	1.446
No results	Not the top	No results	Full match		132.0429	2.558
No results	No results	No results	No results		380.12114	5.932
No results	No results	No results	No results		559.37245	13.725
No results	No results	No results	No results		478.13162	1.004
No results	No results	No results	No results		269.93309	7.048
No results	No results	No results	Full match		281.11245	2.539
No results	No results	No results	No results		395.09641	5.316
No results	Partial matcl	No results	Full match		381.99919	7.796
No results	No results	No results	No results		172.96268	18.171
No results	No results	No results	Full match		740.19533	6.042
No results	No results	No results	Full match		147.06836	6.198
No results	No results	No results	Full match		375.31378	14.984
No results	No results	No results	Full match		362.10047	6.146
No results	No results	No results	No results		434.06933	1.052
No results	No results	No results	Full match		509.48128	15.35
No results	No results	No results	No results		701.52908	15.946
No results	No results	No results	No results		349.21014	6.899
No results	No results	No results	No results		188.86724	18.414
No results	No results	No results	No results		419.01665	2.149

No results	No results	No results	Full match		318.07361	7.347
No results	No results	No results	No results		163.05959	0.763
No results	No results	No results	No results		312.08425	5.229
No results	No results	No results	Full match		262.22951	14.501
No results	No results	No results	No results		444.28099	7.104
No results	No results	No results	No results		1160.5609	9.7
No results	No results	No results	No results		350.06511	0.947
No results	No results	No results	No results		626.23414	7.114
No results	Partial matcl	No results	Full match		340.07927	6.264
No results	No results	No results	Full match		568.32561	12.365
No results	No results	No results	No results		543.35679	12.344
No results	No results	No results	No results		338.12107	1.41
No results	No results	No results	No results		434.31423	14.492
No results	No results	No results	No results		187.4363	18.145
No results	No results	No results	No results		166.13586	9.428
No results	No results	No results	Full match		280.13087	11.682
No results	No results	No results	No results		395.15787	6.19
No results	No results	No results	Full match		256.10568	3.633
No results	No results	No results	No results		596.40816	11.03
No results	No results	No results	No results		292.10582	5.944
No results	No match	No results	Full match		528.05692	6.037
No results	No results	No results	No results		424.10032	7.334
No results	No results	No results	No results		795.51004	18.149
No results	Partial matcl	No results	Full match		292.20357	13.742
No results	No results	No results	No results		141.05821	5.887
No results	No results	No results	No results		361.23433	18.045
No results	No results	No results	No results		547.29374	16.082
No results	No results	No results	No results		402.86821	18.176
No results	No results	No results	No results		579.19536	6.059
No results	No results	No results	Full match		258.08575	0.998
No results	No results	No results	No results		676.20039	6.263
No results	No results	No results	No results		308.08942	6.992
No results	No results	No results	No results		600.38191	12.057
No results	Partial matcl	No results	Full match		246.10003	6.989
No results	No results	No results	No results		433.13627	6.277
No results	No results	No results	No results		278.04228	8.05
No results	No results	No results	No results		136.05159	1.417
No results	No results	No results	No results		498.11586	6.987
No results	No results	No results	No results		628.14234	6.045
No results	No results	No results	Full match		211.09576	1.64
No results	No results	No results	No results		343.14232	7.361
No results	No results	No results	No results		540.44328	16.679
No results	No results	No results	Full match		305.2354	12.312
No results	Partial matcl	No results	Full match		316.05806	6.393
No results	No results	No results	Full match		414.09498	7.63
No results	No results	No results	No results		731.54051	14.124
No results	Partial matcl	No results	Full match		216.03956	0.895
No results	No results	No results	Full match		250.13149	1.052
No results	No results	No results	No results		184.0361	2.871
No results	No results	No results	No results		485.16878	7.098

No results	No results	No results	No results		203.04208	0.991
No results	No results	No results	No results		394.04748	3.902
No results	No results	No results	Full match		580.17885	6.915
No results	No results	No results	No results		827.56514	7.019
No results	Partial match	No results	Full match		127.06346	1.432
No results	No results	No results	No results		434.04534	5.883
No results	No results	No results	No results		312.16896	5.85
No results	No results	No results	No results		1034.2907	7.272
No results	No results	No results	Full match		255.25621	10.566
No results	No results	No results	Full match		294.18314	10.909
No results	No results	No results	No results		364.20991	15.746
No results	No results	No results	No results		468.08854	6.158
No results	No results	No results	Full match		414.16817	7.436
No results	No results	No results	No results		499.13324	5.401
No results	Partial match	No results	Full match		154.02648	6.502
No results	No results	No results	No results		314.12173	1.001
No results	No results	No results	Full match		198.01737	1.025
No results	No results	No results	No results		442.04045	1.027
No results	No results	No results	No results		562.09309	6.476
No results	No results	No results	No results		976.6148	7.186
No results	No results	No results	No results		311.3552	14.473
No results	No results	No results	No results		303.04592	6.696
No results	No results	No results	No results		295.22233	13.58
No results	No results	No results	No results		252.04781	0.94
No results	No results	No results	Full match		311.31889	17.876
No results	No results	No results	No results		269.18521	4.947
No results	No results	No results	No results		438.11575	2.424
No results	No results	No results	No results		510.3207	7.14
No results	No results	No results	No results		175.04714	0.98
No results	No results	No results	No results		185.12761	0.99
No results	No results	No results	No results		271.94521	0.77
No results	Partial match	No results	Full match		338.06369	7.115
No results	No results	No results	No results		373.35554	14.065
No results	No results	No results	No results		155.97711	18.336
No results	Partial match	No results	Full match		278.22418	15.72
No results	No results	No results	No results		269.1852	5.173
No results	No results	No results	No results		286.1163	3.404
No results	No results	No results	No results		606.13719	6.758
No results	No results	No results	No results		473.13165	6.262
No results	No results	No results	No results		344.16258	6.924
No results	No results	No results	No results		400.01231	4.555
No results	No results	No results	No results		1020.6414	7.273
No results	Partial match	No results	Full match		404.11032	7.512
No results	Partial match	No results	Full match		152.0473	6.398
No results	No results	No results	No results		247.08441	6.081
No results	No results	No results	No results		268.10513	2.032
No results	No results	No results	No results		530.16481	6.415
No results	No results	No results	No results		488.30742	7.224
No results	No results	No results	No results		416.21032	11.687
No results	No results	No results	No results		690.15778	6.691

No results	No results	No results	No results		353.36584	17.97
No results	No results	No results	No results		416.24128	7.245
No results	No results	No results	No results		268.14237	2.064
No results	No results	No results	No results		221.02993	18.16
No results	No results	No results	Full match		206.05791	6.178
No results	No results	No results	No results		464.11618	0.996
No results	No results	No results	No results		464.26203	7.664
No results	No results	No results	No results		129.51918	3.971
No results	No results	No results	No results		800.04469	2.067
No results	No results	No results	No results		437.03419	4.567
No results	No results	No results	Full match		383.10026	6.76
No results	No results	No results	No match		392.12564	8.823
No results	No results	No results	Full match		610.18945	6.819
No results	No results	No results	Full match		436.13655	6.871
No results	No results	No results	No results		245.07228	0.997
No results	No results	No results	No results		892.46394	14.697
No results	No results	No results	No results		224.11611	6.064
No results	No results	No results	No results		577.21065	14.587
No results	No results	No results	No results		1958.8895	15.502
No results	No results	No results	No results		252.14736	6.071
No results	No results	No results	No results		333.07757	1.567
No results	No results	No results	No results		418.05101	6.708
No results	No results	No results	No results		305.05351	6.27
No results	No results	No results	No results		307.05986	0.769
No results	No results	No results	No results		256.15232	18.135
No results	Partial matcl	No results	Full match		205.03735	5.863
No results	No results	No results	No results		533.31217	9.99
No results	No results	No results	No results		222.13131	18.135
No results	No results	No results	No results		379.084	5.967
No results	Partial matcl	No results	Full match		132.04211	0.978
No results	No results	No results	Partial matcl		216.13532	8.879
No results	No results	No results	Full match		468.12624	1.01
No results	No results	No results	No results		222.01603	6.253
No results	No results	No results	No results		333.1214	6.01
No results	Partial matcl	No results	Full match		443.15697	6.527
No results	No results	No results	No results		368.91744	18.167
No results	Partial matcl	No results	Full match		119.0585	0.919
No results	No results	No results	No results		406.20082	7.397
No results	No results	No results	No results		604.33023	7.506
No results	Partial matcl	No results	Full match		404.11037	7.212
No results	No results	No results	No results		455.12136	6.148
No results	No results	No results	No results		289.94343	0.761
No results	No results	No results	No results		156.12639	10.718
No results	No results	No results	No results		190.12332	0.982
No results	No results	No results	No results		253.93465	0.776
No results	No results	No results	No results		321.17397	18.211
No results	No results	No results	No results		374.09657	1.024
No results	No results	No results	No results		371.00926	7.095
No results	No results	No results	No results		532.08253	6.24
No results	No results	No results	No results		536.11378	6.298

No results	No results	No results	No results		274.19325	13.945
No results	Partial matcl	No results	Full match		210.08941	7.274
No results	No results	No results	No results		139.97675	0.942
No results	No results	No results	No results		353.3294	16.542
No results	No results	No results	No results		152.01016	7.684
No results	No results	No results	No results		514.13183	5.795
No results	No results	No results	No results		482.4015	16.069
No results	No results	No results	No results		178.04661	1.435
No results	No results	No results	No results		469.1299	5.794
No results	Partial matcl	No results	Full match		374.10023	6.636
No results	No results	No results	No results		515.32483	12.981
No results	No results	No results	No results		870.45787	18.147
No results	No results	No results	No results		1064.6676	7.333
No results	No results	No results	No results		806.16891	6.375
No results	No results	No results	Full match		200.07917	1.07
No results	No results	No results	No results		368.01986	7.733
No results	No results	No results	Full match		430.08912	7.111
No results	No results	No results	No results		521.27266	13.921
No results	No results	No results	No results		276.89562	4.213
No results	Partial matcl	No results	Full match		356.11221	6.24
No results	Partial matcl	No results	Full match		358.10495	7.603
No results	No results	No results	No results		266.0424	8.383
No results	No results	No results	No results		216.11519	18.137
No results	No results	No results	No results		491.28591	12.741
No results	No results	No results	No results		288.13229	5.814
No results	No results	No results	No results		320.07222	5.9
No results	No results	No results	No results		296.967	18.159
No results	No results	No results	No results		1405.0018	16.577
No results	Partial matcl	No results	Full match		302.07903	6.134
No results	No results	No results	No results		267.00554	1.22
No results	No results	No results	Full match		252.11095	1.745
No results	Partial matcl	No results	Full match		464.09569	6.037
No results	No results	No results	No results		444.06909	6.249
No results	No results	No results	No results		657.21241	1.02
No results	No results	No results	No results		310.18115	17.095
No results	No results	No results	No results		299.19444	5.988
No results	No results	No results	No results		776.23721	5.892
No results	No results	No results	No results		697.54888	14.828
No results	No results	No results	No results		892.46321	14.249
No results	No results	No results	No results		166.02547	6.312
No results	No results	No results	No results		457.10117	5.981
No results	No results	No results	No results		170.1057	0.984
No results	No results	No results	No results		345.32439	12.887
No results	No results	No results	Full match		250.14294	2.101
No results	No results	No results	Full match		250.13156	1.509
No results	No results	No results	No results		482.05136	7.046
No results	No results	No results	No results		582.11928	1.528
No results	No results	No results	Full match		252.11097	2.169
No results	No results	No results	No results		594.27072	10.457
No results	No results	No results	No results		345.25166	8.836

No results	No results	No results	No results		1152.7205	7.393
No results	No results	No results	No results		695.53618	17.408
No results	No results	No results	No results		286.94387	18.146
No results	No results	No results	No results		596.408	16.319
No results	No results	No results	No results		478.09526	1.009
No results	No results	No results	No results		461.4081	15.624
No results	No results	No results	No results		175.13645	18.192
No results	No results	No results	No results		454.06615	7.095
No results	No results	No results	No results		1338.624	8.754
No results	Partial matcl	No results	Full match		264.1364	6.009
No results	No results	No results	No results		206.01043	18.161
No results	No results	No results	Full match		434.12065	6.077
No results	Not the top	No results	Full match		182.05814	5.999
No results	No results	No results	Full match		500.13186	7.194
No results	No results	No results	Full match		199.04715	4.54
No results	No results	No results	No results		182.62096	5.586
No results	No results	No results	Full match		192.07883	6.102
No results	No results	No results	No results		604.25211	7.122
No results	No results	No results	No results		640.18508	2.362
No results	No results	No results	Full match		290.18825	10.508
No results	No results	No results	No results		516.08754	7.594
No results	No results	No results	No results		295.12816	5.863
No results	No results	No results	No results		489.12687	6.329
No results	No results	No results	No results		250.98131	1.063
No results	No results	No results	No results		289.05833	8.874
No results	Partial matcl	No results	Full match		402.09699	7.59
No results	No results	No results	No results		282.15778	6.1
No results	No results	No results	No results		614.0005	6.131
No results	No results	No results	No results		567.42045	16.875
No results	No results	No results	Full match		258.08467	1.499
No results	No results	No results	No results		274.11641	1.419
No results	No results	No results	No results		532.18363	8.078
No results	No results	No results	No results		168.87727	18.334
No results	No results	No results	Full match		610.13285	1.024
No results	No results	No results	No results		669.3763	5.775
No results	No results	No results	No results		583.16894	6.668
No results	No results	No results	No results		485.08805	6.007
No results	No results	No results	No results		358.05102	3.445
No results	No results	No results	No results		503.30091	12.565
No results	No results	No results	No results		111.06753	0.99
No results	No results	No results	No results		262.04761	6.906
No results	No results	No results	No results		807.5761	18.06
No results	No results	No results	No results		154.0254	6.871
No results	No results	No results	No results		251.08714	6.266
No results	No results	No results	No results		248.03974	3.901
No results	No results	No results	Full match		271.15748	18.144
No results	No results	No results	No results		278.16299	6.479
No results	No results	No results	No results		249.03139	2.816
No results	No results	No results	No results		245.07229	1.419
No results	No results	No results	No results		264.14991	18.153

No results	No results	No results	No results		237.99592	2.109
No results	No results	No results	No results		216.14748	2.308
Full match	Partial match	No results	Full match		264.13588	7.858
No results	No results	No results	No results		159.08838	3.205
No results	No results	No results	No results		485.13988	8.808
No results	No results	No results	No results		407.99898	3.887
No results	No results	No results	No results		196.03616	5.582
No results	No results	No results	No results		591.09034	6.134
No results	No results	No results	No results		526.04231	6.762
No results	No results	No results	No results		531.34052	12.374
No results	No results	No results	Full match		215.0809	1.898
No results	No results	No results	No results		721.20119	6.251
No results	No results	No results	No results		271.99065	18.413
No results	No results	No results	Full match		772.1842	5.997
No results	No results	No results	No results		138.99474	18.144
No results	Full match	No results	No results		322.13745	1.451
No results	No results	No results	No results		111.95973	0.775
No results	No results	No results	No results		445.17349	6.046
No results	No results	No results	No results		1470.0938	7.508
No results	No results	No results	No results		766.47629	18.01
No results	No results	No results	Full match		494.14228	7.276
No results	No results	No results	No results		246.0179	6.4
No results	No results	No results	No results		538.03981	6.448
No results	No results	No results	No results		520.19417	6.906
No results	No results	No results	No results		362.08477	1.107
No results	No results	No results	No results		369.06917	4.562
No results	No results	No results	Full match		304.12703	1.818
No results	No results	No results	No results		147.96722	1.856
No results	No results	No results	No results		1108.6942	7.324
No results	No results	No results	No results		189.13356	6.144
No results	No results	No results	Full match		348.08441	6.212
No results	No results	No results	No results		378.26731	6.149
No results	No results	No results	Full match		350.24584	10.72
No results	No results	No results	No results		477.33029	10.275
No results	Full match	No results	No results		530.06959	6.493
No results	No results	No results	No results		806.2421	7.795
No results	No results	No results	Full match		176.13145	3.714
No results	No results	No results	No results		215.94339	0.759
No results	No results	No results	No results		772.51245	17.076
No results	No results	No results	Full match		600.11143	6.841
No results	No results	No results	No results		305.12618	6.715
No results	No results	No results	Full match		424.21108	6.909
No results	No results	No results	Full match		344.12594	7.742
No results	No results	No results	Full match		180.09001	2.713
No results	No results	No results	No results		548.17573	6.226
No results	No results	No results	No results		447.11708	6.022
No results	No results	No results	No results		390.02636	6.008
No results	No results	No results	No results		250.09546	6.167
No results	No results	No results	Full match		430.08966	2.66
No results	No results	No results	No results		812.50521	17.089

No results	Full match	No results	Full match		226.04713	5.802
No results	No results	No results	Full match		346.0796	1.027
No results	No results	No results	Full match		618.12221	6.118
No results	No results	No results	No results		408.10644	6.161
No results	No results	No results	No results		123.03083	1.432
No results	No results	No results	No results		413.0341	1.012
No results	No results	No results	No results		1151.5737	8.296
No results	No results	No results	No results		152.04606	6.74
No results	No results	No results	No results		341.67789	5.62
No results	No results	No results	No results		665.05856	4.566
No results	No results	No results	No results		421.28307	10.633
No results	No results	No results	No results		129.06188	0.992
No results	No results	No results	Full match		482.10591	1.558
No results	No results	No results	No results		820.59032	14.276
No results	No results	No results	Full match		594.13715	11.538
No results	No results	No results	No results		376.19173	16.503
No results	No results	No results	No results		1084.6694	13.432
No results	No results	No results	No results		283.97468	1.203
No results	No results	No results	No results		588.40593	18.071
No results	No results	No results	No results		453.28511	13.602
No results	No results	No results	No results		447.15293	6.11
No results	No results	No results	No results		516.22116	13.041
No results	No results	No results	No results		257.12641	2.127
No results	No results	No results	No results		172.0107	4.567
No results	No results	No results	No results		554.21521	8.07
No results	No results	No results	No results		240.13563	12.102
No results	No results	No results	No results		233.09021	1.422
No results	No results	No results	No results		400.01189	3.877
No results	Partial matcl	No results	Full match		179.05833	2.086
No results	No results	No results	No results		455.43396	14.797
No results	No results	No results	No results		383.10766	4.073
No results	No results	No results	Full match		276.12097	7.258
No results	No results	No results	Full match		171.12597	6.937
No results	No results	No results	No results		138.52446	3.905
No results	No results	No results	Full match		564.12713	6.905
No results	No results	No results	No results		546.30464	9.487
No results	No results	No results	No results		484.49708	14.898
No results	No results	No results	Full match		237.08482	0.946
No results	No results	No results	No results		213.17304	9.36
No results	No results	No results	No results		2044.9257	15.55
No results	No results	No results	No results		484.27968	18.007
No results	No results	No results	No results		700.38795	13.426
No results	No results	No results	No results		149.95583	0.748
No results	Partial matcl	No results	Full match		162.05275	0.942
No results	No results	No results	No results		508.27931	17.006
No results	No results	No results	Full match		614.18521	5.904
No results	No results	No results	Full match		430.09001	2.944
No results	No results	No results	No results		238.95935	0.768
No results	No results	No results	No results		845.54131	18.01
No results	No results	No results	Full match		225.11543	11.292

No results	No results	No results	No results		584.46984	16.652
No results	No results	No results	No results		708.13093	6.019
No results	No results	No results	No results		305.9941	1.214
No results	No results	No results	No results		153.12346	7.15
No results	No results	No results	No results		389.35061	11.554
No results	No results	No results	No results		1177.5894	9.517
No results	No results	No results	No results		568.28143	9.453
No results	No results	No results	Full match		348.08504	6.925
No results	No results	No results	Full match		314.06384	6.017
No results	No results	No results	No results		501.27249	12.348
No results	No results	No results	Full match		482.10607	1.811
No results	No results	No results	Full match		232.14224	3.921
No results	No results	No results	No results		335.31893	15.568
No results	Partial match	No results	Full match		448.10022	6.135
No results	No results	No results	No results		501.16335	6.703
No results	No results	No results	No results		190.04604	6.056
No results	No results	No results	Full match		196.12571	18.158
No results	No results	No results	Full match		356.0738	3.932
No results	No results	No results	No results		582.12065	1.009
No results	No results	No results	No results		338.28234	11.827
No results	No results	No results	No results		201.06274	1.421
No results	No results	No results	No results		678.20088	14.294
No results	No results	No results	Full match		345.04792	1.416
No results	No results	No results	No results		539.23674	6.657
No results	No results	No results	Full match		276.17259	10.919
No results	No results	No results	Full match		281.08967	6.414
No results	No results	No results	No results		238.08358	8.436
No results	No results	No results	No results		286.95183	0.754
No results	No results	No results	No results		770.26308	6.609
No results	No results	No results	No results		257.12627	2.792
No results	No results	No results	No results		588.12737	6.594
No results	No results	No results	No results		617.42848	14.165
No results	No results	No results	No results		485.13986	8.638
No results	No results	No results	No results		183.9612	18.351
No results	No results	No results	No results		576.31398	10.535
No results	No results	No results	No results		237.97865	4.221
No results	No results	No results	No results		266.0636	0.978
No results	No results	No results	No results		499.13271	1.026
No results	No results	No results	No results		569.11677	6.407
No results	No results	No results	Full match		356.29271	14.205
No results	No results	No results	No results		280.02155	4.476
No results	No results	No results	Full match		337.33449	17.066
No results	Partial match	No results	Full match		188.11613	2.095
No results	No results	No results	No results		319.25125	12.897
No results	No results	No results	No results		597.29939	15.362
No results	Full match	No results	Full match		214.13167	2.209
No results	No results	No results	No results		806.32568	12.369
No results	No results	No results	No results		233.04229	2.252
No results	No results	No results	No results		192.16281	6.747
No results	No results	No results	No results		238.04759	6.079

No results	No results	No results	No results		849.5383	18.107
No results	No results	No results	No results		100.0013	0.766
No results	No results	No results	No results		587.40251	15.707
No results	No results	No results	No results		417.38174	15.638
No results	No results	No results	No results		1177.5894	9.701
No results	No results	No results	Full match		306.14274	1.418
No results	No results	No results	No results		263.16094	18.189
No results	No results	No results	No results		505.43456	15.596
No results	No results	No results	Full match		360.12101	6.896
No results	No results	No results	No results		248.97264	4.733
No results	No results	No results	Full match		374.13656	6.947
No results	No results	No results	No results		409.13714	6.145
No results	No results	No results	No results		250.98131	3.07
No results	No results	No results	No results		419.90134	18.151
No results	No results	No results	No results		470.10536	6.239
No results	No results	No results	No results		336.13223	5.985
No results	No results	No results	Full match		311.31874	15.091
No results	No results	No results	No results		328.05563	3.908
No results	No results	No results	No results		386.06369	6.636
No results	Partial matcl	No results	Full match		166.02653	6.303
No results	No results	No results	No results		352.06862	6.21
No results	No results	No results	No results		443.9993	2.535
No results	No results	No results	Full match		350.24588	10.573
No results	No results	No results	No results		596.29594	18.161
No results	No results	No results	No results		708.13016	7.827
No results	No results	No results	No results		204.12082	18.133
No results	No results	No results	No results		451.56316	5.886
No results	No results	No results	No results		171.11656	0.99
No results	No results	No results	No results		279.11041	6.458
No results	No results	No results	No results		330.10712	2.063
No results	No results	No results	No results		615.43467	13.714
No results	No results	No results	No results		225.10027	2.619
No results	No results	No results	No results		672.16902	6.397
No results	No results	No results	No results		310.18111	16.852
No results	No results	No results	No results		1449.027	16.504
No results	No results	No results	No results		323.10834	6.887
No results	No results	No results	No results		213.97827	2.91
No results	No results	No results	No results		426.00236	3.941
No results	No results	No results	No results		256.06933	0.995
No results	No results	No results	No results		289.99922	4.756
No results	No results	No results	No results		162.03061	7.102
No results	No results	No results	Full match		185.10521	1.431
No results	No results	No results	No results		401.38701	13.946
No results	No results	No results	Full match		195.12578	1.023
No results	No results	No results	No results		404.00474	3.876
No results	No results	No results	No results		224.11616	6.793
No results	No results	No results	No results		151.04009	1.323
No results	No results	No results	No results		194.0933	10.455
No results	Partial matcl	No results	Full match		180.04229	6.018
No results	No results	No results	No results		314.12273	1.5

No results	No results	No results	Full match		309.16884	2.064
No results	No results	No results	No results		402.05817	7.047
No results	No results	No results	Full match		257.10521	8.802
No results	No results	No results	No results		205.1288	18.114
No results	No results	No results	No results		679.50835	18.108
No results	Partial match	No results	Full match		170.02159	1.569
No results	No results	No results	No results		288.08459	2.923
No results	No results	No results	No results		635.34075	14.399
No results	No results	No results	No results		356.03764	1.645
No results	No results	No results	No results		329.2281	8.849
No results	No results	No results	No results		322.91114	18.159
No results	No results	No results	No results		358.12858	6.851
No results	No results	No results	No results		415.35753	16.917
No results	No results	No results	No results		358.16261	6.842
No results	No results	No results	No results		282.15795	1.591
No results	No results	No results	Full match		169.03747	1.434
No results	No results	No results	No results		361.15224	6.736
No results	No results	No results	No results		514.36618	15.863
No results	No results	No results	No results		557.32451	14.397
No results	No results	No results	Full match		415.12635	6.27
No results	No results	No results	No results		1196.7466	7.498
No results	No results	No results	No results		496.24138	8.088
No results	No results	No results	No results		479.10598	6.079
No results	No results	No results	No results		381.02788	2.052
No results	No results	No results	No results		227.07725	2.007
No results	No results	No results	No results		530.09087	1.103
No results	No results	No results	No results		699.44048	15.056
No results	No results	No results	No results		555.26356	12.568
No results	No results	No results	Full match		322.06852	1.788
No results	No results	No results	Full match		104.06302	4.368
No results	No results	No results	No results		374.04836	3.898
No results	No results	No results	No results		387.38649	14.694
No results	No results	No results	Full match		402.05568	8.067
No results	No results	No results	Full match		290.11861	7.308
No results	No results	No results	No results		533.24785	13.077
No results	No results	No results	Full match		318.07372	6.891
No results	No results	No results	No results		128.04601	2.538
No results	No results	No results	No results		262.08389	3.894
No results	No results	No results	No results		537.22095	6.914
No results	No results	No results	No results		726.0956	2.051
No results	No results	No results	No results		201.10977	5.975
No results	No results	No results	No results		1210.7262	7.533
No results	No results	No results	No results		503.86629	18.168
No results	No results	No results	Full match		340.10002	0.963
No results	No results	No results	No results		271.06934	0.978
No results	No results	No results	No results		274.06865	1.729
No results	No results	No results	No results		328.22514	8.498
No results	Partial match	No results	Full match		264.11116	6.059
No results	No results	No results	No results		253.95104	4.217
No results	No results	No results	No results		591.41413	14.44

No results	No results	No results	No results		189.13371	18.124
No results	No results	No results	No results		177.10007	0.935
No results	No results	No results	No results		233.98285	3.6
No results	Full match	No results	Full match		261.12125	1.873
No results	No results	No results	No results		240.62475	18.172
No results	No results	No results	No results		175.90653	18.255
No results	No results	No results	No results		203.04217	1.412
No results	No results	No results	No results		179.98392	1.295
No results	Partial match	No results	Full match		171.05328	2.203
No results	No results	No results	Full match		306.21955	13.822
No results	No results	No results	No results		389.11086	6.851
No results	No results	No results	Full match		220.08478	1.907
No results	No results	No results	No results		389.14745	6.159
No results	No results	No results	Full match		352.02507	7.85
No results	No results	No results	Full match		202.09549	1.576
No results	No results	No results	Full match		564.11018	4.227
No results	No results	No results	No results		234.05299	6.112
No results	No results	No results	Full match		235.08432	6.115
No results	Partial match	No results	Full match		180.04231	4.553
No results	No results	No results	No results		1317.9498	16.681
No results	No results	No results	No results		179.08084	1.881
No results	No results	No results	No results		136.0512	6.937
No results	No results	No results	No results		206.11828	18.13
No results	No results	No results	Full match		199.0486	2.088
No results	Partial match	No results	Full match		129.04238	1.028
No results	No results	No results	No results		563.12669	3.283
No results	No results	No results	No results		305.08986	0.998
No results	No results	No results	No results		262.03555	0.915
No results	No results	No results	No results		510.0792	7.054
No results	No results	No results	No results		508.21023	7.481
No results	No results	No results	No results		614.48018	16.011
No results	No results	No results	No results		279.07399	6.265
No results	No results	No results	No results		559.33558	14.504
No results	No results	No results	No results		272.1469	18.133
No results	No results	No results	No results		526.27947	11.16
No results	No results	No results	Full match		304.16333	6.018
No results	No results	No results	No results		716.23755	6.029
No results	No results	No results	No results		142.87415	18.385
No results	No results	No results	No results		1167.5312	14.808
No results	No results	No results	No results		451.81459	5.876
No results	No results	No results	No results		285.30313	12.849
No results	No results	No results	No results		189.13364	5.892
No results	No results	No results	No results		225.13462	0.995
No results	No results	No results	No results		316.04294	1.02
No results	No results	No results	No results		395.10887	5.973
No results	No results	No results	No results		1136.582	8.854
No results	No results	No results	No results		485.13969	8.716
No results	No results	No results	No results		233.09238	1.415
No results	Partial match	No results	Full match		246.05278	6.038
No results	No results	No results	Full match		610.09595	6.844

No results	No results	No results	No results		1583.177	7.583
No results	No results	No results	No results		548.48339	18.103
No results	No results	No results	No results		476.17938	6.144
No results	No results	No results	No results		442.12391	6.88
No results	No results	No results	No results		231.96646	3.316
No results	No results	No results	No results		570.45255	16.039
No results	No results	No results	Full match		200.07957	1.641
No results	No results	No results	No results		668.13735	6.397
No results	No results	No results	No results		363.13176	6.611
No results	No results	No results	No results		248.97247	4.228
No results	No results	No results	No results		195.13112	5.739
No results	No results	No results	No results		181.11807	18.112
No results	No results	No results	Full match		296.10445	10.156
No results	No results	No results	No results		644.17439	6.075
No results	No results	No results	No results		182.1259	0.983
No results	No results	No results	Full match		564.10969	4.591
No results	Partial matcl	No results	Full match		402.09535	5.98
No results	No results	No results	No results		349.31923	16.092
No results	No results	No results	No results		436.11867	7.246
No results	No results	No results	No results		101.01497	0.769
No results	No results	No results	No results		441.0388	3.942
No results	No results	No results	No results		1322.6295	9.072
No results	No results	No results	No results		974.46415	5.477
No results	No results	No results	No results		176.9576	18.166
No results	No results	No results	No results		234.08871	6.818
No results	No results	No results	No results		417.38183	12.847
No results	No results	No results	No results		549.46092	15.55
No results	No results	No results	No results		332.19643	11.953
No results	No results	No results	No results		598.09651	6.931
No results	No results	No results	No results		421.13725	6.876
No results	No results	No results	No results		506.37644	10.757
No results	Partial matcl	No results	Full match		296.09078	6.838
No results	No results	No results	No results		185.06779	5.365
No results	No results	No results	No results		452.09483	7.574
No results	No results	No results	No results		810.5047	18.139
No results	No results	No results	No results		132.02141	3.897
No results	No results	No results	No results		403.12683	6.142
No results	No results	No results	No results		169.9551	18.419
No results	No results	No results	Full match		287.28247	10.74
No results	No results	No results	Full match		902.24833	7.87
No results	No results	No results	No results		485.13969	8.877
No results	No results	No results	No results		982.58638	15.279
No results	No results	No results	No results		579.31712	9.844
No results	No results	No results	No results		233.13482	18.174
No results	No results	No results	Full match		376.11577	6.502
No results	No results	No results	Full match		255.11061	0.994
No results	No results	No results	No results		488.09168	7.074
No results	No results	No results	No results		872.23716	7.51
No results	No results	No results	No results		289.127	0.995
No results	No results	No results	Full match		478.11119	6.13

No results	No results	No results	Full match		227.04317	3.312
No results	No results	No results	No results		144.92979	18.19
No results	No results	No results	No results		249.1364	18.08
No results	No results	No results	Full match		412.11554	6.619
No results	No results	No results	No results		247.08442	6.907
No results	No results	No results	No results		337.25062	13.237
No results	Partial matcl	No results	Full match		330.07374	6.891
No results	No results	No results	No results		327.21569	6.159
No results	No results	No results	No results		1055.706	7.128
No results	No results	No results	No results		452.39073	16.709
No results	No results	No results	No results		271.02734	1.224
No results	No results	No results	No results		363.97418	4.032
No results	No results	No results	Full match		350.24588	9.81
No results	No results	No results	No results		380.24107	16.499
No results	No results	No results	No results		211.12882	18.12
No results	No results	No results	No results		785.19543	15.134
No results	No results	No results	No results		1132.5301	7.69
No results	No results	No results	No results		1156.8387	17.96
No results	No results	No results	No results		474.28921	17.88
No results	No results	No results	No results		252.08397	1.43
No results	Partial matcl	No results	Full match		278.22401	13.718
No results	No results	No results	Full match		740.17416	9.248
No results	No results	No results	No results		233.46932	18.15
No results	No results	No results	No results		233.10859	1.421
No results	No results	No results	No results		147.96722	1.279
No results	No results	No results	No results		294.09505	8.059
No results	No results	No results	Full match		285.09603	1.982
No results	Partial matcl	No results	Full match		267.09725	2.068
No results	No results	No results	No results		484.13721	7.868
No results	No results	No results	Full match		480.09025	3.129
No results	No results	No results	No results		283.98176	3.262
No results	No results	No results	No results		295.03678	2.813
No results	No results	No results	No results		695.29545	13.444
No results	No results	No results	Full match		374.13622	7.753
No results	No results	No results	No results		359.13677	6.504
No results	No results	No results	No results		587.13798	4.862
No results	No results	No results	No results		591.13689	6.808
No results	No results	No results	No results		224.11593	6.462
No results	No results	No results	No results		191.99773	18.131
No results	No results	No results	No results		384.01475	7.933
No results	No results	No results	No results		514.11066	6.605
No results	No results	No results	No results		251.08488	6.249
No results	No results	No results	No results		538.15913	6.106
No results	Partial matcl	No results	Full match		280.23981	16.31
No results	No results	No results	Full match		165.11545	18.08
No results	No results	No results	No results		1358.013	7.464
No results	No results	No results	No results		383.30351	15.592
No results	No results	No results	Full match		220.08495	2.089
No results	No results	No results	No results		265.08118	1.84
No results	No results	No results	No results		340.15184	6.994

No results	No results	No results	No results		311.12309	4.254
No results	No results	No results	No results		276.1473	6.634
No results	No results	No results	Full match		384.23719	2.063
No results	No results	No results	No results		212.11612	6.031
No results	No results	No results	No results		588.12765	6.347
No results	No results	No results	No results		577.21034	14.329
No results	No results	No results	Full match		422.09989	7.587
No results	Partial matcl	No results	Full match		344.08979	6.15
No results	No results	No results	No results		435.11675	7.397
No results	No results	No results	No results		415.16301	6.039
No results	No results	No results	No results		264.01737	5.829
No results	No results	No results	Full match		296.11027	1.453
No results	No results	No results	No results		157.08463	0.998
No results	No results	No results	No results		287.96461	0.346
No results	Partial matcl	No results	Full match		358.0687	9.223
No results	No results	No results	No results		678.2007	14.058
No results	No results	No results	Full match		482.12092	7.465
No results	No results	No results	Full match		396.15743	7.433
No results	No results	No results	No results		320.07244	3.628
No results	No results	No results	No results		182.0206	2.989
No results	No results	No results	No results		318.14284	2.106
No results	No results	No results	No results		325.09509	6.074
No results	No results	No results	No results		248.12567	6.572
No results	No results	No results	No results		1112.8133	17.999
No results	No results	No results	No results		193.62767	5.623
No results	No results	No results	No results		270.08519	1.801
No results	Partial matcl	No results	Full match		316.05802	7.568
No results	No results	No results	No results		752.15827	6.454
No results	No results	No results	Full match		276.17253	14.74
No results	No results	No results	No results		979.58708	15.293
No results	No results	No results	No results		209.05406	1.427
No results	No results	No results	No results		760.48829	18.045
No results	No results	No results	No results		239.04259	1.752
No results	No results	No results	No results		261.08456	1.003
No results	No results	No results	No results		343.05355	6.014
No results	No results	No results	No results		379.19196	6.912
No results	No results	No results	No results		279.15495	18.15
No results	No results	No results	No results		787.53844	14.525
No results	No results	No results	Full match		472.09998	6.908
No results	No results	No results	No results		304.07918	1.498
No results	No results	No results	No results		496.35562	10.737
No results	No results	No results	Full match		358.14186	6.848
No results	No results	No results	No results		496.35559	11.41
No results	No results	No results	Full match		162.12565	7.525
No results	No results	No results	No results		338.28224	12.929
No results	No results	No results	No results		319.0692	1.089
No results	Partial matcl	No results	Full match		141.11543	7.305
No results	No results	No results	Full match		326.04293	2.692
No results	No results	No results	Full match		206.14208	3.152
No results	No results	No results	No results		1361.9762	16.602

No results	No results	No results	No results		459.26173	12.987
No results	No results	No results	No results		158.08457	5.776
No results	No results	No results	No results		160.88463	18.423
No results	No results	No results	No results		455.1429	6.071
No results	No results	No results	No results		285.15761	1.715
No results	No results	No results	No results		550.07473	6.261
No results	No results	No results	No results		406.27476	18.013
No results	No results	No results	No results		373.03094	3.979
No results	No results	No results	No results		482.10362	0.915
No results	No results	No results	No results		632.22766	13.297
No results	No results	No results	No results		274.19343	11.924
No results	No results	No results	No results		608.01362	6.039
No results	No results	No results	No results		510.28891	5.623
No results	No results	No results	No results		322.21751	16.292
No results	No results	No results	No results		163.09297	18.129
No results	No results	No results	No results		508.27955	17.514
No results	No results	No results	Full match		207.07578	1.541
No results	Partial matcl	No results	Full match		170.02125	1.205
No results	No results	No results	No results		504.10369	5.559
No results	No results	No results	No results		223.97175	1.118
No results	Partial matcl	No results	Full match		356.12595	6.945
No results	No results	No results	No results		305.06142	7.283
No results	No results	No results	No results		139.98855	18.169
No results	No results	No results	No results		254.13673	18.166
No results	No results	No results	No results		979.58632	15.515
No results	No results	No results	Full match		320.89412	18.16
No results	No results	No results	No results		308.14669	6.575
No results	No results	No results	No results		733.26447	6.034
No results	No results	No results	No results		199.03832	4.027
No results	No results	No results	Full match		488.10834	6.01
No results	No results	No results	Full match		201.13665	3.336
No results	No results	No results	No results		179.97363	1.985
No results	No results	No results	Full match		436.09942	1.002
No results	No results	No results	No results		513.30902	12.328
No results	No results	No results	No results		924.21773	6.443
No results	No results	No results	No results		198.13884	18.12
No results	No results	No results	Full match		251.07933	6.116
No results	No results	No results	No results		288.14333	1.475
No results	Full match	No results	No results		150.0318	6.039
No results	No results	No results	No results		764.4605	18.004
No results	No results	No results	No results		614.09021	6.248
No results	Partial matcl	No results	Full match		328.07909	6.071
No results	No results	No results	No results		715.54485	16.556
No results	No results	No results	No results		274.88865	18.174
No results	No results	No results	No results		246.15787	2.674
No results	No results	No results	No results		188.14411	18.174
No results	No results	No results	No results		129.06185	1.429
No results	No results	No results	No results		539.32206	12.839
No results	No results	No results	No results		411.11653	6.019
No results	No results	No results	No results		248.13686	5.537

No results	Partial matcl	No results	Full match		436.33434	15.857
No results	No results	No results	No results		533.2753	13.031
No results	No results	No results	No results		616.39804	7.538
No results	No results	No results	Full match		297.10702	2.363
No results	No results	No results	Full match		436.11526	8.04
No results	No results	No results	No results		361.29811	14.649
No results	Full match	No results	No results		316.07927	3.378
No results	No results	No results	No results		532.24932	7.695
No results	No results	No results	No results		608.26372	16.93
No results	No results	No results	Full match		534.09865	5.981
No results	No results	No results	No results		944.67281	18.011
No results	No results	No results	Full match		370.07464	0.93
No results	No results	No results	No results		206.17848	7.207
No results	No results	No results	No results		288.16866	6.165
No results	No results	No results	No results		245.0612	3.449
No results	No results	No results	No results		274.04765	7.111
No results	No results	No results	Full match		220.08489	2.203
No results	No results	No results	No results		345.17885	14.995
No results	No results	No results	No results		466.31089	18.018
No results	Partial matcl	No results	Full match		301.29818	12.295
No results	No results	No results	No results		445.10074	6.033
No results	No results	No results	No results		201.0638	1.766
No results	No results	No results	Full match		434.14171	5.975
No results	No results	No results	No results		354.36101	14.071
No results	No results	No results	No results		185.46235	18.142
No results	No results	No results	No results		461.09553	6.101
No results	No results	No results	No results		253.1678	3.659
No results	No results	No results	No results		288.14328	1.565
No results	No results	No results	No results		606.28429	14.805
No results	No results	No results	Full match		143.07357	5.778
No results	Partial matcl	No results	Full match		164.0685	0.948
No results	No results	No results	No results		226.01065	2.985
No results	No results	No results	Full match		243.21984	12.715
No results	Partial matcl	No results	Full match		386.09998	7.451
No results	No results	No results	No results		238.04701	5.901
No results	No results	No results	Full match		263.18862	11.016
No results	No results	No results	No results		224.09148	6.104
No results	No results	No results	No results		495.26796	6.893
No results	No results	No results	No results		283.10525	2.223
No results	No results	No results	Full match		271.15756	5.745
No results	No results	No results	No results		473.98591	3.917
No results	No results	No results	Full match		422.09981	7.676
No results	No results	No results	No results		221.9375	3.289
No results	No results	No results	Full match		220.07348	6.946
No results	No results	No results	No results		612.07527	5.96
No results	No results	No results	No results		838.48797	18.013
No results	No results	No results	No results		310.06895	5.959
No results	No results	No results	No results		421.17369	6.937
No results	No results	No results	No results		219.07575	2.044
No results	No results	No results	Full match		552.12552	7.85

No results	No results	No results	No results		595.14067	7.814
No results	No results	No results	No results		469.12976	5.568
No results	Partial matcl	No results	Full match		300.08455	1.012
No results	Partial matcl	No results	Full match		195.05234	6.087
No results	No results	No results	No results		770.26316	6.766
No results	No results	No results	No results		673.45545	16.144
No results	No results	No results	No results		584.32271	15.699
No results	No results	No results	No results		476.05937	6.03
No results	No results	No results	No results		563.12631	3.749
No results	No results	No results	No results		620.4286	14.581
No results	No results	No results	Full match		249.11127	12.569
No results	No results	No results	Full match		396.12025	7.04
No results	No results	No results	Full match		634.13228	6.918
No results	No results	No results	No results		721.45567	16.466
No results	No results	No results	Full match		160.10012	6.081
No results	No results	No results	Full match		143.07358	6.096
No results	No results	No results	Full match		196.14539	12.101
No results	No results	No results	No results		454.0926	7.638
No results	No results	No results	Full match		213.03141	2.24
No results	No results	No results	Full match		396.0367	5.246
No results	No results	No results	No results		484.25144	8.825
No results	No results	No results	No results		238.04709	6.769
No results	No results	No results	Full match		638.12769	6.272
No results	No results	No results	Full match		239.13101	12.429
No results	No results	No results	Full match		182.02062	2.692
No results	No results	No results	No results		167.97371	3.124
No results	No results	No results	No results		225.09221	6.174
No results	No results	No results	Full match		888.25407	6.514
No results	No results	No results	No results		296.00928	1.075
No results	No results	No results	Full match		199.04715	5.006
No results	No results	No results	No results		432.1908	12.096
No results	Full match	No results	Full match		486.15234	8.007
No results	No results	No results	No results		355.06914	0.923
No results	No results	No results	No results		890.22762	6.207
No results	No results	No results	Full match		372.0841	8.293
No results	No results	No results	No results		497.09886	6.47
No results	No results	No results	No results		498.16416	6.165
No results	No results	No results	No results		824.52835	15.148
No results	No results	No results	No results		226.0107	2.691
No results	Partial matcl	No results	Full match		388.11574	6.961
No results	No results	No results	No results		211.12891	5.811
No results	No results	No results	Full match		164.09492	6.117
No results	No results	No results	Full match		294.18314	13.016
No results	No results	No results	No results		555.52302	15.537
No results	No results	No results	No results		354.20728	17.803
No results	No results	No results	No results		318.07581	6.917
No results	No results	No results	No results		432.06877	6.886
No results	No results	No results	No results		170.05921	6.124
No results	No results	No results	No results		355.27219	14.829
No results	No results	No results	Full match		406.10481	8.298

No results	No results	No results	No results		445.13753	6.114
No results	No results	No results	No results		688.14881	1.022
No results	Full match	No results	No results		530.06996	6.286
No results	No results	No results	No results		417.21523	11.157
No results	No results	No results	No results		282.03734	5.987
No results	No results	No results	No results		243.92216	4.75
No results	No results	No results	No results		358.99549	4.462
No results	Partial matc	No results	Full match		402.09463	9.311
No results	No results	No results	No results		338.28215	12.623
No results	No results	No results	No results		231.11069	6.154
No results	No results	No results	Full match		368.25635	13.972
No results	No results	No results	Full match		584.15272	6.445
No results	No results	No results	No results		399.37132	13.167
No results	No results	No results	Full match		314.06349	6.564
No results	No results	No results	No results		423.16454	6.129
No results	No results	No results	No results		508.26842	10.902
No results	No results	No results	Full match		228.1153	8.161
No results	Partial matc	No results	Full match		330.07378	7.081
No results	No results	No results	No results		221.06892	5.919
No results	No results	No results	No results		548.10194	1.008
No results	No results	No results	No results		373.04742	6.813
No results	No results	No results	No results		504.10367	5.8
No results	No results	No results	No results		447.40802	17.722
No results	No results	No results	No results		1190.5703	9.452
No results	No results	No results	No results		532.24923	7.831
No results	No results	No results	No results		602.39761	11.026
No results	No results	No results	No results		336.26656	14.353
No results	No results	No results	No results		608.28442	12.342
No results	No results	No results	No results		584.32258	16.019
No results	No results	No results	No results		201.88758	18.186
No results	No results	No results	No results		333.26693	12.94
No results	No results	No results	No results		1158.5822	9.939
No results	No results	No results	No results		165.07787	2.818
No results	No results	No results	No results		100.01828	0.772
No results	No results	No results	No results		230.05355	1.005
No results	No results	No results	No results		405.01769	4.554
No results	Partial matc	No results	Full match		323.05207	1.416
No results	No results	No results	No results		536.12968	9.478
No results	No results	No results	No results		549.38011	15.606
No results	No results	No results	No results		290.07461	0.996
No results	No results	No results	No results		594.27074	10.291
No results	No results	No results	No results		289.06655	3.926
No results	No results	No results	No results		279.10533	13.387
No results	No results	No results	No results		806.5174	16.368
No results	No results	No results	No results		536.13189	7.3
No results	No results	No results	No results		546.18566	6.16
No results	No results	No results	Full match		710.183	5.934
No results	No results	No results	No results		545.3932	15.246
No results	No results	No results	No results		195.08851	12.361
No results	No results	No results	No results		464.11601	1.407

No results	No results	No results	Full match		640.10553	6.011
No results	No results	No results	No results		538.33498	14.935
No results	No results	No results	No results		358.99556	4.041
No results	Full match	No results	Full match		225.0638	3.91
No results	No results	No results	No results		162.05521	3.909
No results	No results	No results	No results		471.12477	6.169
No results	No results	No results	No results		466.0749	5.966
No results	No results	No results	Full match		220.15766	6.186
No results	No results	No results	Full match		424.33371	14.428
No results	No results	No results	No results		662.4186	16.096
No results	No results	No results	No results		1166.6997	7.515
No results	Full match	No results	No results		248.10492	6.891
No results	Partial matc	No results	Full match		164.04742	5.984
No results	No results	No results	No results		366.14282	5.974
No results	No results	No results	No results		390.16748	6.855
No results	No results	No results	No results		359.28226	14.58
No results	No results	No results	No results		618.13717	7.05
No results	No results	No results	No results		231.91549	0.753
No results	No results	No results	No results		304.91887	0.781
No results	No results	No results	Full match		206.13071	8.796
No results	No results	No results	No results		264.06318	6.595
No results	No results	No results	No results		674.22127	7.637
No results	No results	No results	No results		298.07452	2.026
No results	No results	No results	No results		274.06591	1.05
No results	No results	No results	No results		794.52138	18.019
No results	No results	No results	No results		1200.8655	17.917
No results	No results	No results	Full match		636.14796	6.977
No results	No results	No results	No results		450.09293	12.216
No results	No results	No results	No results		310.05819	8.189
No results	No results	No results	Full match		310.17787	11.725
No results	No results	No results	Full match		257.10338	13.378
No results	Partial matc	No results	Full match		206.09439	6.071
No results	No results	No results	No results		492.28517	12.599
No results	No results	No results	Full match		440.11248	6.934
No results	No results	No results	No results		229.09441	5.343
No results	No results	No results	No results		460.06411	7.526
No results	No results	No results	No results		375.13207	5.84
No results	No results	No results	No results		554.39765	10.288
No results	No results	No results	No results		213.6277	18.135
No results	No results	No results	No results		352.06874	6.023
No results	No results	No results	No results		224.02257	18.394
No results	No results	No results	No results		499.13378	5.74
No results	No results	No results	No results		469.13725	6.734
No results	No results	No results	No results		684.40849	14.778
No results	No results	No results	No results		321.05589	5.918
No results	No results	No results	No results		498.164	5.977
No results	No results	No results	Full match		219.12615	18.13
No results	No results	No results	Full match		250.08402	6.733
No results	No results	No results	No results		632.11715	6.127
No results	No results	No results	Full match		418.09004	7.339

No results	No results	No results	No results		392.27142	15.566
No results	No results	No results	No results		171.05221	1.454
No results	No results	No results	No results		302.10022	2.379
No results	No results	No results	No results		184.93599	18.173
No results	No results	No results	No results		332.16667	13.404
No results	No results	No results	Full match		332.0896	7.468
No results	No results	No results	No results		239.47279	18.17
No results	No results	No results	No results		278.18578	8.917
No results	No results	No results	No results		960.6017	16.12
No results	No results	No results	Full match		490.11072	7.396
No results	No results	No results	No results		265.00373	2.828
No results	No results	No results	No results		549.18459	6.175
No results	No results	No results	No results		243.92208	4.233
No results	No results	No results	No results		259.91706	2.176
No results	No results	No results	No results		436.09797	7.479
No results	No results	No results	Full match		616.10607	6.567
No results	No results	No results	No results		564.35379	14.399
No results	No results	No results	No results		428.13107	5.918
No results	No results	No results	No results		708.16994	5.919
No results	No results	No results	Full match		570.46612	18.079
No results	No results	No results	No results		643.48726	15.447
No results	No results	No results	Full match		231.14728	4.093
No results	No results	No results	No results		567.13072	2.807
No results	No results	No results	No results		228.93793	18.16
No results	No results	No results	Full match		558.29573	17.509
No results	No results	No results	No results		693.22802	6.14
No results	No results	No results	No results		585.08009	6.199
No results	No results	No results	No results		469.14473	9.158
No results	No results	No results	Full match		537.51225	16.686
No results	No results	No results	No results		567.13068	3.268
No results	No results	No results	No results		288.04253	5.76
No results	No results	No results	No results		223.13155	18.153
No results	No results	No results	Full match		390.33455	16.964
No results	No results	No results	No results		239.04267	1.068
No results	No results	No results	No results		201.10995	18.125
No results	No results	No results	No results		255.14714	5.945
No results	No results	No results	No results		283.98408	4.745
No results	No results	No results	No results		449.42336	17.157
No results	No results	No results	No results		241.14735	18.167
No results	No results	No results	Full match		1149.5579	7.682
No results	No results	No results	No results		317.89656	18.172
No results	No results	No results	No results		417.13853	5.906
No results	No results	No results	No results		946.68703	17.984
No results	No results	No results	No results		792.37538	13.214
No results	No results	No results	No results		815.63292	17.592
No results	No results	No results	No results		582.32765	13.443
No results	No results	No results	No results		182.92362	18.384
No results	No results	No results	Full match		205.96503	3.282
No results	No results	No results	No results		794.57469	17.755
No results	No results	No results	No results		241.13152	5.95

No results	No results	No results	No results		257.14149	18.15
No results	No results	No results	No results		852.5264	18.086
No results	No results	No results	No results		238.81329	18.188
No results	No results	No results	No results		401.11501	5.906
No results	No results	No results	No results		424.06145	6.344
No results	Full match	No results	Full match		486.15234	7.606
No results	No results	No results	No results		249.14406	18.116
No results	No results	No results	Full match		339.3501	16.842
No results	No results	No results	No results		228.15148	11.003
No results	No results	No results	No results		582.37126	12.112
No results	No results	No results	No results		568.26552	10.734
No results	No results	No results	No match		392.12562	8.915
No results	No results	No results	No results		224.15273	2.821
No results	No results	No results	No results		1164.5564	7.305
No results	No results	No results	Full match		222.16121	14.326
No results	No results	No results	No results		173.03134	1.017
No results	No results	No results	No results		525.1638	6.434
No results	No results	No results	No results		882.16296	6.586
No results	No results	No results	No results		568.01573	2.048
No results	No results	No results	Full match		193.07384	6.143
No results	No results	No results	No results		209.00023	2.941
No results	No results	No results	No results		436.18566	13.162
No results	No results	No results	No results		445.24607	12.988
No results	Partial match	No results	Full match		338.06347	4.571
No results	No results	No results	No results		257.27193	11.524
No results	No results	No results	No results		674.22701	3.449
No results	No results	No results	No results		462.20969	8.189
No results	No results	No results	Full match		1076.5395	8.96
No results	No results	No results	No results		169.11794	18.128
No results	No results	No results	No results		185.9842	2.937
No results	No results	No results	No results		1081.4941	14.659
No results	No results	No results	No results		315.24929	9.116
No results	No results	No results	No results		924.51193	18.165
No results	No results	No results	No results		665.05805	3.919
No results	No results	No results	No results		260.06834	6.155
No results	No results	No results	No results		364.26148	14.529
No results	No results	No results	No results		1191.6052	9.899
No results	No results	No results	No results		576.26107	10.405
No results	No results	No results	No results		277.06621	6.181
No results	No results	No results	No results		599.16428	7.104
No results	No results	No results	Full match		478.0728	6.108
No results	No results	No results	No results		356.0377	1.148
No results	No results	No results	No results		309.12103	6.548
No results	No results	No results	No results		401.29784	8.664
No results	No results	No results	No results		302.06068	8.957
No results	No results	No results	No results		859.6599	17.506
No results	No results	No results	No results		444.06888	7.326
No results	No results	No results	No results		693.27983	12.563
No results	No results	No results	No results		1068.7866	18.058
No results	No results	No results	No results		240.95684	18.157

No results	Partial matcl	No results	Full match		169.01356	2.212
No results	No results	No results	No results		582.11934	2.039
No results	No results	No results	No results		453.85163	18.169
No results	No results	No results	No results		208.06792	7.171
No results	No results	No results	No results		273.22152	16.291
No results	No results	No results	No results		221.09141	2.438
No results	No results	No results	No results		341.09021	6.006
No results	No results	No results	No results		333.26685	13.629
No results	No results	No results	No results		250.15178	6.172
No results	No match	No results	Full match		348.04788	6.069
No results	No results	No results	No results		302.16515	6.734
No results	No results	No results	No results		559.32625	13.725
No results	No results	No results	No results		428.26828	18.113
No results	No results	No results	No results		165.15186	6.821
No results	No results	No results	Full match		281.08993	7.267
No results	No results	No results	Full match		298.10543	6.469
No results	No results	No results	No results		507.26731	14.859
No results	No results	No results	No results		130.94236	18.188
No results	No results	No results	No results		355.09421	7.079
No results	No results	No results	No results		351.09538	6.76
No results	No results	No results	Full match		211.09575	1.107
No results	Full match	No results	No results		514.07503	6.829
No results	No results	No results	No results		813.50884	17.093
No results	No results	No results	No results		373.19303	13.502
No results	No results	No results	No results		262.0479	6.128
No results	No results	No results	Full match		568.12117	6.422
No results	No results	No results	No results		579.28459	14.004
No results	No results	No results	Full match		548.15286	6.92
No results	No results	No results	Full match		410.24338	18.159
No results	No results	No results	No results		739.54451	17.332
No results	No results	No results	No results		493.15859	6.868
No results	No results	No results	Full match		276.20875	13.408
No results	No results	No results	No results		250.15208	18.093
No results	Partial matcl	No results	Full match		118.02716	1.415
No results	No results	No results	Full match		586.09547	4.499
No results	Partial matcl	No results	Full match		142.09949	9.418
No results	No results	No results	No results		240.11125	6.054
No results	No results	No results	No results		596.07997	6.255
No results	No results	No results	Full match		222.07347	1.412
No results	No results	No results	No results		554.16145	7.023
No results	Partial matcl	No results	Full match		516.12601	7.023
No results	No results	No results	No results		634.11464	6.04
No results	No results	No results	No results		962.46879	18.157
No results	No results	No results	Full match		250.08364	2.682
No results	No results	No results	No results		315.88664	0.073
No results	No results	No results	No results		590.49155	15.931
No results	No results	No results	Full match		492.12665	7.098
No results	No results	No results	No results		242.15475	18.135
No results	No results	No results	Full match		430.09005	6.119
No results	No results	No results	No results		287.07706	2.03

No results	No results	No results	No results		199.08459	5.88
No results	No results	No results	No results		214.13373	18.134
No results	No results	No results	No results		851.52449	18.114
No results	No results	No results	No results		604.12139	6.08
No results	No results	No results	No results		391.12786	6.021
No results	No results	No results	No results		223.13813	18.211
No results	No results	No results	No results		679.41295	18.099
No results	No results	No results	Full match		613.26784	14.007
No results	No results	No results	Full match		209.06889	6.128
No results	No results	No results	No results		270.97788	1.562
No results	No results	No results	No results		582.31737	14.083
No results	No results	No results	Full match		536.38693	11.031
No results	No results	No results	No results		528.24133	6.202
No results	No results	No results	No results		503.14003	6.96
No results	No results	No results	No results		159.03772	4.027
No results	No results	No results	No results		195.01773	13.499
No results	No results	No results	No results		320.01651	8.102
No results	No results	No results	Full match		313.08757	5.643
No results	No results	No results	No results		572.13201	6.868
No results	No results	No results	No results		278.89085	4.777
No results	No results	No results	No results		437.33775	10.302
No results	No results	No results	No results		535.32804	10.504
No results	No results	No results	No results		261.09984	8.661
No results	No results	No results	No results		365.08282	6.126
No results	No results	No results	No results		856.50668	18.068
No results	No results	No results	No results		231.04192	2.249
No results	No results	No results	No results		244.1307	7.485
No results	No results	No results	No results		770.16914	6.032
No results	No results	No results	Full match		303.14262	1.434
No results	No results	No results	No results		193.02637	6.128
No results	No results	No results	No results		166.02551	6.013
No results	No results	No results	No results		641.23149	6.609
No results	No results	No results	No results		154.96709	0.107
No results	No results	No results	Full match		318.07359	7.176
No results	No results	No results	Full match		391.09022	3.915
No results	No results	No results	No results		225.00524	18.167
No results	No results	No results	No results		528.29428	6.106
No results	No results	No results	No results		138.03048	6.92
No results	No results	No results	No results		514.13191	5.573
No results	No results	No results	No results		421.25737	12.076
No results	No results	No results	No results		205.05976	0.958
No results	No results	No results	No results		468.14238	7.417
No results	No results	No results	No results		340.89791	18.187
No results	No results	No results	No results		445.24611	12.748
No results	Partial matcl	No results	Full match		357.1067	5.779
No results	No results	No results	No results		159.98463	18.187
No results	No results	No results	Full match		315.11476	4.941
No results	No results	No results	No results		202.13181	3.864
No results	No results	No results	No results		539.06855	12.349
No results	No results	No results	No results		331.24368	9.278

No results	No results	No results	Full match		370.06837	7.621
No results	No results	No results	No results		155.90455	18.385
No results	Not the top	No results	Full match		335.06413	6.051
No results	No results	No results	Full match		232.03728	6.16
No results	No results	No results	No results		158.01869	4.014
No results	No results	No results	No results		382.23848	18.019
No results	No results	No results	No results		364.26422	15.926
No results	No results	No results	No results		745.55507	15.923
No results	No results	No results	Full match		374.13625	7.606
No results	No results	No results	No results		612.11146	6.448
No results	Partial match	No results	Full match		181.03686	1.034
No results	No results	No results	No results		179.98398	1.857
No results	No results	No results	No results		793.52653	17.107
No results	No results	No results	No results		488.08728	7.042
No results	Partial match	No results	Full match		464.09547	1.065
No results	No results	No results	Full match		169.11088	1.516
No results	No results	No results	No results		1254.7516	7.632
No results	No results	No results	No results		454.34464	16.09
No results	No results	No results	No results		328.15233	15.515
No results	No results	No results	No results		582.31743	13.505
No results	No results	No results	Full match		258.08487	2.263
No results	No results	No results	No results		368.15969	6.91
No results	No results	No results	No results		389.11086	6.199
No results	No results	No results	No results		238.0836	2.632
No results	No results	No results	No results		212.13661	18.267
No results	No results	No results	No results		514.36615	7.918
No results	No results	No results	No results		819.20564	6.56
No results	No results	No results	No results		552.04326	2.048
No results	No results	No results	No results		235.47267	5.88
No results	No results	No results	No results		380.25603	13.531
No results	No results	No results	No results		671.35617	5.606
No results	No match	No results	Full match		458.12079	7.09
No results	No results	No results	No results		251.01073	4.22
No results	No results	No results	Full match		278.12672	2.676
No results	No results	No results	No results		214.09535	1.614
No results	No results	No results	No results		433.98223	2.084
No results	No results	No results	Full match		564.10914	5.953
No results	No results	No results	No results		280.14221	4.549
No results	No results	No results	No results		448.14102	11.051
No results	No results	No results	No results		163.05127	6.028
No results	No results	No results	No results		364.26142	14.043
No results	Full match	No results	Full match		222.05241	6.591
No results	No results	No results	No results		253.09495	1.998
No results	Partial match	No results	Full match		165.04281	2.123
No results	No results	No results	No results		337.93327	2.145
No results	No results	No results	No results		487.14735	7.094
No results	No results	No results	Full match		244.20331	14.347
No results	No results	No results	No results		176.9843	18.292
No results	No results	No results	No results		579.31702	9.981
No results	No results	No results	No results		201.07502	1.847

No results	No results	No results	No results		598.12984	7.546
No results	No results	No results	No results		756.51344	18.083
No results	No results	No results	No results		295.22243	14.535
No results	No results	No results	No results		928.67594	18.002
No results	No results	No results	No results		554.10595	8.02
No results	No results	No results	Full match		287.2825	10.449
No results	No results	No results	No results		246.15787	2.14
No results	No results	No results	No results		363.3355	16.712
No results	No results	No results	No results		395.12162	6.145
No results	No results	No results	No results		104.00977	1.429
No results	No results	No results	No results		1475.9735	16.076
No results	No results	No results	No results		401.08463	6.131
No results	No results	No results	No results		189.61969	18.148
No results	No results	No results	No results		223.04579	2.063
No results	No results	No results	No results		528.08754	7.07
No results	No results	No results	No results		200.12558	18.171
No results	No results	No results	Full match		638.12752	6.005
No results	Not the top	No results	Full match		182.05724	6.262
No results	No results	No results	No results		512.31104	18.091
No results	No results	No results	No results		369.14268	6.124
No results	No results	No results	No results		136.05038	1.417
No results	No results	No results	No results		441.14202	7.043
No results	No results	No results	Full match		326.11439	1.817
No results	No results	No results	No results		589.41942	15.221
No results	No results	No results	No results		259.10523	7.337
No results	No results	No results	No results		216.14751	6.152
No results	No results	No results	No results		474.28938	17.507
No results	No results	No results	No results		342.09503	5.868
No results	No results	No results	No results		605.1252	7.511
No results	No results	No results	No results		708.13015	8.035
No results	No results	No results	No results		1146.5454	8.833
No results	No results	No results	Full match		422.09978	8.209
No results	No results	No results	No results		478.41347	14.125
No results	No results	No results	No results		827.59753	17.262
No results	No results	No results	Full match		458.08479	5.989
No results	No results	No results	No results		233.00016	4.229
No results	No results	No results	No results		646.26309	14.132
No results	No results	No results	No results		570.09149	3.904
No results	No results	No results	No results		903.68663	17.415
No results	No results	No results	No results		537.16435	7.249
No results	No results	No results	Full match		466.12666	7.725
No results	No results	No results	Full match		430.08931	7.622
No results	No results	No results	No results		161.06756	1.014
No results	No results	No results	Full match		209.10535	5.91
No results	No results	No results	No results		431.05281	3.896
No results	No results	No results	No results		381.36066	14.277
No results	No results	No results	No results		280.0215	3.985
No results	No results	No results	No results		484.30902	18.14
No results	No results	No results	No results		701.36637	5.708
No results	Partial matcl	No results	Full match		314.07891	7.696

No results	Partial matcl	No results	Full match		195.05244	3.48
No results	No results	No results	No results		1113.6123	13.437
No results	No results	No results	No results		313.33453	14.052
No results	No results	No results	No results		383.2156	6.148
No results	No results	No results	No results		568.28653	9.472
No results	No results	No results	No results		791.03954	18.042
No results	No results	No results	Full match		372.084	8.367
No results	No results	No results	No results		674.22694	3.02
No results	No results	No results	No results		311.21751	11.975
No results	No results	No results	No results		295.08453	10.968
No results	No results	No results	No results		494.08465	7.563
No results	No results	No results	No results		512.29914	10.707
No results	No results	No results	No results		1126.5553	11.345
No results	No results	No results	No results		807.57695	16.305
No results	No results	No results	Full match		281.0902	2.233
No results	No results	No results	Full match		740.19546	7.726
No results	No results	No results	Full match		310.12621	5.527
No results	No results	No results	No results		279.13192	2.095
No results	No results	No results	No results		376.20964	7.51
No results	No results	No results	No results		302.09794	2.237
No results	No results	No results	No results		385.19508	6.007
No results	No results	No results	No results		604.1214	7.176
No results	No results	No results	Full match		390.13118	7.111
No results	No results	No results	No results		253.01659	1.233
No results	No results	No results	No results		426.07428	6.044
No results	No results	No results	No results		760.57958	18.104
No results	No results	No results	No results		536.12956	8.984
No results	No results	No results	No results		183.9951	7.623
No results	No results	No results	No results		300.6946	5.731
No results	No results	No results	No results		408.06904	6.062
No results	No results	No results	No results		388.12037	13.921
No results	No results	No results	No results		354.09276	3.734
No results	No results	No results	Full match		350.24567	13.961
No results	No results	No results	No results		335.67839	5.622
No results	No results	No results	No results		558.18666	6.95
No results	No results	No results	No results		487.29369	11.696
No results	No match	No results	Full match		393.12131	7.191
No results	No results	No results	No results		813.55182	13.435
No results	No results	No results	No results		535.32812	10.685
No results	No results	No results	No results		473.13145	6.605
No results	No results	No results	No results		144.87113	18.403
No results	No results	No results	No results		286.07976	5.713
No results	No results	No results	No results		620.11661	7.242
No results	No results	No results	No results		768.49162	18.012
No results	Partial matcl	No results	Full match		204.08909	5.759
No results	No results	No results	No results		262.13388	5.854
No results	No results	No results	No results		231.00558	1.441
No results	No results	No results	No results		358.05101	3.739
No results	No results	No results	No results		687.5135	15.413
No results	No results	No results	No results		1430.9458	16.142

No results	No results	No results	Full match		159.10076	1.821
No results	No results	No results	No results		193.62743	18.155
No results	No results	No results	No results		262.15267	5.571
No results	No results	No results	No results		362.11159	6.106
No results	No results	No results	No results		190.0492	0.999
No results	No results	No results	No results		651.32907	5.853
No results	No results	No results	No results		679.44518	15.939
No results	No results	No results	No results		403.0981	7.353
No results	No results	No results	No results		350.20719	8.897
No results	No results	No results	No results		431.16969	6.081
No results	No results	No results	No results		398.07644	3.903
No results	No results	No results	No results		158.0774	2.212
No results	No results	No results	No results		134.02303	1.419
No results	No results	No results	No results		148.98113	18.241
No results	No results	No results	No results		296.19874	12.463
No results	No results	No results	No results		496.35546	15.866
No results	No results	No results	No results		253.13149	4.146
No results	No results	No results	No results		575.04558	12.338
No results	No results	No results	No results		446.1761	6.906
No results	No results	No results	No results		348.04265	4.551
No results	No results	No results	No results		858.52346	18.113
No results	No results	No results	No results		383.28835	8.669
No results	No results	No results	No results		817.50831	15.68
No results	No results	No results	No results		514.11064	6.253
No results	No results	No results	No results		478.13163	2.859
No results	No results	No results	No results		349.03169	1.236
No results	No results	No results	Full match		295.15737	8.432
No results	No results	No results	No results		460.20949	11.678
No results	Partial matc	No results	Full match		214.04703	5.352
No results	No results	No results	No results		805.55919	15.717
No results	No results	No results	No results		650.10346	3.906
No results	No results	No results	No results		926.66157	18.004
No results	No results	No results	No results		670.37918	5.799
No results	No results	No results	No results		310.06853	3.097
No results	No results	No results	No results		323.07199	7.123
No results	No results	No results	No results		395.09591	5.701
No results	Partial matc	No results	Full match		214.04712	5.718
No results	No results	No results	No results		268.95283	18.145
No results	No results	No results	No results		1288.9186	17.846
No results	Partial matc	No results	Full match		208.07353	7.233
No results	No results	No results	No results		412.13436	5.981
No results	No results	No results	Full match		434.31851	11.923
No results	No results	No results	No results		716.37459	14.199
No results	No results	No results	No results		860.53888	18.071
No results	No results	No results	Full match		222.1103	18.153
No results	No results	No results	No results		363.97386	4.476
No results	No results	No results	Full match		332.0895	7.308
No results	No results	No results	No results		295.22266	14.675
No results	No results	No results	No results		429.05765	2.597
No results	No results	No results	No results		328.95215	2.252

No results	No results	No results	No results		332.15178	2.072
No results	No results	No results	No results		823.54479	16.343
No results	No results	No results	No results		198.1392	5.78
No results	No results	No results	Full match		159.05302	0.991
No results	No results	No results	No results		628.10597	6.654
No results	No results	No results	No results		285.13243	1.065
No results	No results	No results	No results		866.20598	6.073
No results	No results	No results	No results		237.03488	2.295
No results	No results	No results	No results		364.03221	3.886
No results	No results	No results	No results		586.15325	3.549
No results	No results	No results	No results		435.11619	1.051
No results	No results	No results	Full match		278.13027	1.528
No results	Partial matcl	No results	Full match		189.07907	7.551
No results	No results	No results	No results		206.10575	7.557
No results	No results	No results	No results		584.387	11.04
No results	No results	No results	No results		327.68068	5.746
No results	No results	No results	Full match		322.15601	6.045
No results	No results	No results	No results		240.04388	4.012
No results	No results	No results	No results		248.15474	5.575
No results	No results	No results	Full match		460.10074	7.235
No results	No results	No results	Full match		530.17867	7.379
No results	No results	No results	No results		457.13729	7.117
No results	No results	No results	No results		637.26802	13.451
No results	No results	No results	No results		945.6182	13.859
No results	Partial matcl	No results	Full match		456.10462	6.134
No results	No results	No results	Full match		474.07872	3.799
No results	No results	No results	Full match		436.13611	7.203
No results	No results	No results	Full match		434.08433	7.362
No results	No results	No results	No results		797.55644	15.702
No results	No results	No results	No results		172.12132	2.432
No results	No results	No results	No results		610.3996	14.17
No results	No results	No results	Full match		231.07447	1.62
No results	No results	No results	Full match		334.10545	6.117
No results	No results	No results	No results		325.66451	5.85
No results	No results	No results	No results		234.08859	6.55
No results	No results	No results	No results		520.10609	1.01
No results	No results	No results	No results		229.02649	3.266
No results	No results	No results	No results		776.53957	17.277
No results	No results	No results	No results		212.9951	1.44
No results	No results	No results	No results		595.14061	8.019
No results	No results	No results	No results		771.60649	17.672
No results	No results	No results	No results		400.12725	5.957
No results	No results	No results	Full match		332.09156	7.531
No results	No results	No results	Full match		446.08287	7.667
No results	No results	No results	No results		280.96215	1.149
No results	No results	No results	No results		238.08359	5.839
No results	No results	No results	No results		411.13134	7.601
No results	No results	No results	No results		644.40804	11.719
No results	No results	No results	No results		1190.572	8.638
No results	No results	No results	No results		654.30787	13.22

No results	No results	No results	Full match		266.16438	12.593
No results	No results	No results	No results		444.12659	5.966
No results	No results	No results	No results		348.68627	5.691
No results	No results	No results	No results		492.32982	13.805
No results	No results	No results	No results		760.24196	5.943
No results	No results	No results	No results		702.25451	18.156
No results	No results	No results	No results		601.38904	5.744
No results	No results	No results	No results		253.13115	3.421
No results	No results	No results	No results		531.31243	10.595
No results	No results	No results	No results		307.19377	7.383
No results	No results	No results	No results		343.13836	2.075
No results	No results	No results	No results		289.12546	0.984
No results	Full match	No results	Full match		145.03742	0.989
No results	No results	No results	No results		332.92757	18.351
No results	No results	No results	No results		674.5015	13.269
No results	No results	No results	No results		240.02653	4.957
No results	No results	No results	No results		424.27269	5.904
No results	No results	No results	Full match		318.07371	7.596
No results	No results	No results	No results		296.00923	18.16
No results	No results	No results	No results		255.03236	4.221
No results	No results	No results	Full match		257.06859	7.1
No results	No results	No results	No results		239.94567	0.767
No results	No results	No results	No results		415.16225	6.552
No results	No results	No results	No results		1244.8913	17.876
No results	No results	No results	No results		795.55385	15.384
No results	No results	No results	No results		219.17049	9.421
No results	No results	No results	No results		478.13171	5.333
No results	No results	No results	Full match		208.10907	10.303
No results	No results	No results	Full match		348.08421	7.498
No results	No results	No results	No results		327.19091	6.013
No results	No results	No results	No results		171.11753	0.979
No results	No results	No results	Full match		416.23226	14.117
No results	No results	No results	No results		414.04833	3.96
No results	No results	No results	No results		841.55388	15.186
No results	No results	No results	No results		484.13723	7.532
No results	No results	No results	No results		1024.7604	18.095
No results	No results	No results	No results		639.17911	13.723
No results	Partial matcl	No results	Full match		918.24338	7.573
No results	No results	No results	No results		308.09857	2.099
No results	No results	No results	No results		315.3138	12.046
No results	No results	No results	No results		528.29295	13.462
No results	No results	No results	No results		422.25298	6.052
No results	No results	No results	No results		1410.6085	10.295
No results	No results	No results	No results		265.09471	7.421
No results	No results	No results	Full match		218.13084	8.189
No results	No results	No results	No results		213.97906	1.565
No results	Partial matcl	No results	Full match		438.34993	8.868
No results	No results	No results	No results		1020.7287	17.117
No results	No results	No results	No results		1380.6345	10.202
No results	No results	No results	Full match		348.0842	7.314

No results	No results	No results	No results		405.99892	4.556
No results	No results	No results	No results		426.06145	6.288
No results	No results	No results	No results		403.03889	2.066
No results	Partial matcl	No results	Full match		240.06331	1.65
No results	No results	No results	No results		1064.7555	17.086
No results	No results	No results	No results		127.95084	0.887
No results	No results	No results	No results		192.96617	1.297
No results	No results	No results	No results		794.39123	14.198
No results	No results	No results	No results		408.13959	5
No results	No results	No results	No results		655.36109	5.743
No results	Partial matcl	No results	Full match		154.02631	4.772
No results	No results	No results	No results		265.05849	5.885
No results	No results	No results	No results		582.10078	5.982
No results	No results	No results	No results		312.08464	5.634
No results	No results	No results	No results		429.05786	2.813
No results	No results	No results	Full match		280.09443	6.452
No results	No results	No results	No results		200.15651	11.039
No results	No results	No results	No results		368.03435	1.224
No results	No results	No results	No results		202.11764	18.134
No results	No results	No results	No results		341.0902	5.813
No results	No results	No results	No results		589.13487	6.96
No results	No results	No results	No results		125.96511	18.258
No results	No results	No results	No results		379.19197	7.143
No results	No results	No results	No results		217.94371	0.752
No results	No results	No results	No results		724.24278	6.657
No results	No results	No results	No results		787.29003	6.611
No results	No results	No results	No results		818.50557	18.102
No results	No results	No results	Full match		692.23191	7.095
No results	No results	No results	No results		555.14776	4.577
No results	No results	No results	No results		258.03727	3.492
No results	No results	No results	No results		286.85988	0.406
No results	No results	No results	No results		646.26317	14.356
No results	No results	No results	No results		441.10518	6.547
No results	No results	No results	No results		492.17493	6.001
No results	No results	No results	No results		472.14839	6.149
No results	No results	No results	Full match		206.13072	8.354
No results	No results	No results	No results		529.32566	10.634
No results	No results	No results	No results		678.13282	3.034
No results	No results	No results	No results		511.14805	7.196
No results	No results	No results	No results		165.93343	1.3
No results	No results	No results	No results		241.09517	6.044
No results	No results	No results	No results		350.68312	18.139
No results	No results	No results	No results		454.12873	6.474
No results	No results	No results	No results		428.02681	1.173
No results	No results	No results	Full match		536.38684	10.304
No results	No results	No results	No results		747.34022	5.902
No results	No results	No results	No results		382.23891	15.903
No results	No results	No results	No results		307.10549	6.214
No results	No results	No results	No results		269.08979	6.019
No results	No results	No results	No results		544.29422	18.127

No results	Partial matcl	No results	Full match		299.28243	11.911
No results	No results	No results	Full match		282.09615	2.492
No results	No results	No results	Full match		352.0251	7.723
No results	No results	No results	No results		354.2073	17.513
No results	No results	No results	Full match		183.0531	2.074
No results	No results	No results	No results		253.05858	5.881
No results	No results	No results	No results		356.34029	12.895
No results	No results	No results	Full match		182.05932	6.007
No results	No results	No results	No results		289.06642	8.07
No results	Full match	No results	No results		440.36519	14.455
No results	No results	No results	No results		290.24884	16.966
No results	No results	No results	No results		472.35535	15.237
No results	No results	No results	No results		194.96091	1.28
No results	Full match	No results	No results		208.03655	6.814
No results	No results	No results	No results		671.55405	18.503
No results	No results	No results	No results		957.60241	15.898
No results	No results	No results	No results		189.09909	2.335
No results	No results	No results	No results		484.27311	18.125
No results	Full match	No results	No results		384.08465	7.615
No results	No results	No results	No results		509.11704	6.113
No results	No results	No results	Full match		339.09482	6.634
No results	No results	No results	No results		413.14734	7.034
No results	No results	No results	No results		469.13056	2.031
No results	No results	No results	No results		922.52357	18.009
No results	No results	No results	No results		317.17464	6.565
No results	No results	No results	Full match		582.13515	9.446
No results	No results	No results	No results		312.04006	7.049
No results	No results	No results	No results		483.10908	5.385
No results	No results	No results	No results		264.99002	1.14
No results	No results	No results	No results		199.23016	10.479
No results	No results	No results	Full match		344.05306	7.624
No results	No results	No results	No results		348.17808	7.224
No results	No results	No results	No results		453.14217	7.152
No results	Full match	No results	No results		514.07448	7.206
No results	No results	No results	No results		634.08046	6.061
No results	No results	No results	No results		627.18706	17.08
No results	No results	No results	No results		304.12858	1.037
No results	No results	No results	No results		400.31307	11.028
No results	No results	No results	No results		374.9678	4.465
No results	No results	No results	No results		405.10628	8.637
No results	No results	No results	No results		358.13776	2.073
No results	Partial matcl	No results	Full match		211.04797	1.564
No results	Full match	No results	Full match		208.08498	3.318
No results	No results	No results	No results		636.40074	15.864
No results	No results	No results	Full match		396.03578	3.076
No results	No results	No results	No results		1181.5846	7.306
No results	No results	No results	No results		451.56443	18.196
No results	Partial matcl	No results	Full match		142.09955	7.527
No results	No results	No results	No results		488.36209	12.533
No results	No results	No results	No results		566.37683	12.569

No results	No results	No results	No results		444.14772	0.984
No results	No results	No results	No results		357.12132	6.913
No results	No results	No results	No results		218.09435	7.555
No results	Partial matcl	No results	Full match		358.06862	7.455
No results	No results	No results	Full match		566.12552	7.071
No results	No results	No results	No results		144.93336	18.182
No results	No results	No results	No results		255.21986	12.231
No results	No results	No results	No results		185.9842	1.601
No results	No results	No results	No results		275.99382	3.134
No results	No results	No results	Full match		303.14299	2.058
No results	Partial matcl	No results	Full match		278.22453	14.167
No results	Partial matcl	No results	Full match		299.28256	11.973
No results	No results	No results	No results		197.98423	1.966
No results	No results	No results	Full match		170.05815	6.132
No results	No results	No results	No results		299.18486	5.971
No results	No results	No results	No results		376.11159	2.364
No results	No results	No results	No results		327.09576	5.847
No results	No results	No results	No results		322.88971	18.177
No results	No results	No results	No results		1386.6239	10.505
No results	No results	No results	No results		688.16391	6.091
No results	No results	No results	No results		788.50686	17.323
No results	No results	No results	No results		451.08085	6.666
No results	No results	No results	No results		564.31483	7.595
No results	No results	No results	No results		444.06618	7.139
No results	No results	No results	Full match		480.09016	3.372
No results	No results	No results	No results		994.33638	6.08
No results	No results	No results	No results		320.05625	10.908
No results	No results	No results	No results		657.21223	2.357
No results	No results	No results	No results		197.00382	18.357
No results	No results	No results	No results		717.37758	13.811
No results	Partial matcl	No results	Full match		438.35027	10.597
No results	No results	No results	No results		344.14696	7.172
No results	No results	No results	No results		438.22682	6.997
No results	No results	No results	No results		348.04245	3.899
No results	No results	No results	No results		292.16512	7.878
No results	No results	No results	No results		523.33522	10.127
No results	Partial matcl	No results	Full match		138.10453	0.058
No results	No results	No results	No results		1275.6257	11.041
No results	No results	No results	No results		332.13771	1.95
No results	No results	No results	No results		383.10793	4.493
No results	No results	No results	No results		155.13118	9.202
No results	No results	No results	Full match		420.10567	7.366
No results	No results	No results	Full match		459.12142	7.217
No results	No results	No results	Full match		580.17854	6.698
No results	No results	No results	No results		243.25624	10.792
No results	No results	No results	No results		225.10025	1.591
No results	Partial matcl	No results	Full match		154.02639	4.225
No results	No results	No results	No results		379.12658	6.13
No results	No results	No results	Full match		610.13331	3.045
No results	No results	No results	Full match		316.05604	6.861

No results	No results	No results	No results		702.46416	15.91
No results	No results	No results	No results		627.18749	15.412
No results	No results	No results	Full match		714.15919	6.56
No results	No results	No results	Full match		280.10561	4.363
No results	No results	No results	No results		263.07922	4.234
No results	No results	No results	No results		871.62309	17.217
No results	No results	No results	No results		278.0312	2.705
No results	No results	No results	No results		243.14705	2.111
No results	No results	No results	No results		254.13485	3.433
No results	No results	No results	Full match		594.1008	7.044
No results	No results	No results	No results		216.13008	5.844
No results	No results	No results	No results		636.10554	3.915
No results	No results	No results	Full match		482.10631	6.602
No results	No results	No results	No results		452.32619	17.995
No results	No results	No results	Full match		206.05702	7.184
No results	No results	No results	No results		439.11968	6.08
No results	No results	No results	Full match		480.08978	5.765
No results	No results	No results	No results		204.07776	7.825
No results	No results	No results	No results		554.14244	6.815
No results	No results	No results	No results		224.12401	18.13
No results	No results	No results	No results		523.33526	10.278
No results	No results	No results	No results		506.26411	14.304
No results	No results	No results	Full match		192.07772	6.103
No results	No results	No results	No results		308.13711	1.946
No results	No results	No results	No results		656.21663	17.785
No results	No results	No results	No results		604.11897	9.023
No results	No results	No results	No results		495.159	2.49
No results	No results	No results	No results		535.95582	3.93
No results	No results	No results	No results		454.34459	12.567
No results	No results	No results	No results		432.06883	7.171
No results	No results	No results	No results		582.10066	7.261
No results	No results	No results	No results		604.12144	8.47
No results	No results	No results	No results		240.62485	5.563
No results	No results	No results	No results		181.01577	4.544
No results	No results	No results	Full match		332.0895	6.62
No results	No results	No results	No results		617.3834	18.136
No results	No results	No results	No results		522.09793	6.839
No results	No results	No results	No results		654.30757	13.744
No results	No results	No results	No results		297.0847	6.038
No results	No results	No results	No results		1090.5555	9.338
No results	No results	No results	No results		720.16806	6.979
No results	No results	No results	No results		502.07457	5.416
No results	Full match	No results	No results		419.15773	7.033
No results	No results	No results	No results		247.14669	18.176
No results	No results	No results	Full match		311.10063	6.837
No results	No results	No results	Full match		362.1939	8.65
No results	No results	No results	Full match		480.23547	9.224
No results	No results	No results	No results		186.10469	8.161
No results	No results	No results	No results		214.09462	3.424
No results	No results	No results	No results		420.10809	4.032

No results	No results	No results	No results		293.27205	15.923
No results	No results	No results	No results		1018.2961	7.352
No results	No results	No results	No results		239.11579	6.013
No results	No results	No results	No results		598.12984	7.851
No results	No results	No results	No results		701.20659	17.517
No results	No results	No results	No results		421.10879	7.034
No results	Partial matcl	No results	Full match		210.05282	6.115
No results	No results	No results	No results		240.06097	2.012
No results	No results	No results	Full match		383.13729	6.648
No results	No results	No results	No results		552.30641	14.503
No results	No results	No results	No results		193.8784	18.167
No results	No results	No results	No results		155.97369	1.608
No results	No results	No results	No results		454.34448	15.231
No results	No results	No results	Full match		408.1202	7.415
No results	No results	No results	No results		528.29965	5.739
No results	No results	No results	No results		243.0745	1.546
No results	No results	No results	Full match		276.17256	13.35
No results	Partial matcl	No results	Full match		236.07975	1.635
No results	No results	No results	No results		547.36388	14.727
No results	No results	No results	No results		305.05351	6.448
No results	No results	No results	No results		712.34358	12.068
No results	No results	No results	No results		242.15494	5.612
No results	No results	No results	Full match		626.12666	3.896
No results	No results	No results	No results		1104.7502	16.939
No results	Partial matcl	No results	Full match		360.08427	6.54
No results	No results	No results	No results		496.35536	10.973
No results	No results	No results	No results		251.97362	3.055
No results	No results	No results	No results		277.16738	15.162
No results	No results	No results	No results		575.50127	18.13
Full match	No results	No results	Partial matcl		342.10996	7.344
No results	No results	No results	No results		339.10784	2.063
No results	No results	No results	No results		223.07074	1.023
No results	No results	No results	No results		247.89316	1.332
No results	No results	No results	No results		188.08786	1.828
No results	No results	No results	No results		179.44811	18.168
No results	Partial matcl	No results	Full match		154.0265	3.311
No results	No results	No results	No results		268.01903	3.269
No results	No results	No results	No results		266.04239	5.246
No results	No results	No results	No results		528.29406	10.869
No results	No results	No results	No results		266.05017	3.878
No results	No results	No results	No results		189.99263	4.61
No results	No results	No results	No results		460.06387	6.822
No results	Full match	No results	No results		803.54381	16.963
No results	No results	No results	No results		508.27962	18.139
No results	No results	No results	No results		563.12605	4.049
No results	No results	No results	No results		315.18021	6.083
No results	No results	No results	No results		581.33267	10.502
No results	No results	No results	No results		524.2465	7.515
No results	No results	No results	No results		523.3362	14.931
No results	Partial matcl	No results	Full match		136.12527	8.082

No results	No results	No results	No results		915.65053	17.192
No results	No results	No results	No results		342.68584	18.149
No results	No results	No results	No results		368.15963	7.147
No results	No results	No results	Full match		586.09523	3.955
No results	No results	No results	No results		1093.568	8.96
No results	No results	No results	No results		279.91458	18.146
No results	No results	No results	No results		727.58073	17.778
No results	No results	No results	No results		406.05779	7.486
No results	No results	No results	Full match		311.00914	2.817
No results	No results	No results	No results		578.21324	7.11
No results	No results	No results	No results		118.02724	0.996
No results	No results	No results	No results		176.1255	5.945
No results	No results	No results	No results		162.88175	18.44
No results	No results	No results	No results		260.06822	7.209
No results	No results	No results	No results		849.39366	12.868
No results	No results	No results	No results		345.07329	6.169
No results	No results	No results	No results		123.98392	3.246
No results	No results	No results	Full match		470.26728	12.993
No results	No results	No results	Full match		550.27611	10.817
No results	Partial matcl	No results	Full match		178.02655	6.133
No results	No results	No results	No results		143.94575	18.161
No results	No results	No results	Full match		262.22959	13.235
No results	No results	No results	Full match		482.12181	7.395
No results	No results	No results	Full match		442.12595	8.309
No results	No results	No results	No results		454.34488	14.429
No results	No results	No results	No results		567.42046	17.343
No results	No results	No results	No results		257.27193	10.707
No results	No results	No results	No results		364.07912	7.554
No results	No results	No results	Full match		418.32363	10.246
No results	No results	No results	No results		643.48727	15.576
No results	No results	No results	No results		256.90682	3.239
No results	No results	No results	No results		145.07256	1.431
No results	Partial matcl	No results	Full match		469.15887	2.97
No results	No results	No results	No results		1158.5818	10.522
No results	Full match	No results	No results		381.08634	7.727
No results	No results	No results	No results		160.03605	1.437
No results	No results	No results	No results		242.11499	7.254
No results	No results	No results	No results		318.00359	3.249
No results	Partial matcl	No results	Full match		210.08942	6.116
No results	No results	No results	Full match		231.14728	4.481
No results	No results	No results	No results		285.16903	2.074
No results	Full match	No results	No results		316.07918	5.885
No results	No results	No results	Full match		208.12148	5.843
No results	No results	No results	No results		660.2682	18.156
No results	No results	No results	No results		610.45949	16.1
No results	No results	No results	No results		593.25691	13.444
No results	No results	No results	No results		274.19324	12.438
No results	No results	No results	No results		266.13106	18.158
No results	No results	No results	No results		694.10556	3.036
No results	No results	No results	No results		246.01977	6.377

No results	No results	No results	No results		530.16448	7.019
No results	No results	No results	No results		389.88258	18.155
No results	No results	No results	No results		641.23143	6.761
No results	No results	No results	Full match		406.12648	7.498
No results	No results	No results	Full match		470.33976	12.096
No results	No results	No results	No results		378.09486	7.544
No results	No results	No results	No results		1102.5553	10.352
No results	No results	No results	No results		239.14529	5.876
No results	No results	No results	Full match		310.10496	7.465
No results	Partial matcl	No results	Full match		926.63624	14.404
No results	No results	No results	No results		295.25093	14.23
No results	No results	No results	No results		213.97893	1.154
No results	No results	No results	No results		491.26431	9.817
No results	No results	No results	No results		427.33022	15.586
No results	No results	No results	No results		478.3449	11.044
No results	No results	No results	Full match		255.92319	18.24
No results	No results	No results	Full match		270.12135	5.815
No results	No results	No results	No results		847.62212	15.174
No results	No results	No results	No results		338.25291	14.505
No results	No results	No results	No results		350.2071	8.502
No results	No results	No results	No results		198.01539	1.009
No results	No results	No results	No results		224.97269	3.088
No results	No results	No results	Full match		449.31432	8.971
No results	No results	No results	No results		139.98416	18.172
No results	No results	No results	No results		737.1963	6.826
No results	No results	No results	No results		202.00596	3.253
No results	No results	No results	No results		166.13582	6.821
No results	No results	No results	No results		696.10106	4.558
No results	No results	No results	No results		522.35618	7.925
No results	No results	No results	No results		741.57707	17.768
No results	Partial matcl	No results	Full match		156.04245	6.134
No results	No results	No results	No results		364.11574	6.514
No results	No results	No results	No results		798.55214	18.08
No results	No results	No results	No results		486.07722	7.934
No results	Partial matcl	No results	Full match		328.09434	8.207
No results	No results	No results	No results		484.19471	7.514
No results	No results	No results	No results		708.07665	2.049
No results	No results	No results	No results		464.26202	7.83
No results	No results	No results	No results		478.34471	10.758
No results	No results	No results	No results		633.44579	15.213
No results	No results	No results	No results		587.12807	7.414
No results	No results	No results	No results		506.26405	14.008
No results	No results	No results	No results		262.13423	18.153
No results	No results	No results	No results		522.11403	8.058
No results	No results	No results	No results		435.10381	5.885
No results	No results	No results	No results		450.81532	18.216
No results	No results	No results	No results		1340.6526	11.637
No results	No results	No results	No results		454.1472	2.695
No results	No results	No results	No results		514.36647	8.661
No results	No results	No results	No results		805.55899	17.087

No results	No results	No results	No results		680.51141	18.114
No results	No results	No results	No results		780.15316	6.488
No results	No results	No results	No results		256.14221	1.694
No results	No results	No results	No results		274.01469	7.277
No results	No results	No results	Full match		472.17277	8.104
No results	No results	No results	Full match		472.13714	12.085
No results	No results	No results	No results		232.62735	18.148
No results	Full match	No results	No results		573.12196	1.736
No results	No results	No results	No results		267.11038	12.607
No results	No results	No results	Full match		440.11055	7.796
No results	No results	No results	Full match		482.1207	6.866
No results	No results	No results	No results		1493.0553	16.564
No results	No results	No results	Full match		332.08962	7.807
No results	No results	No results	No results		465.35887	17.969
No results	No results	No results	No results		583.12578	2.014
No results	Full match	No results	Full match		284.07642	1.982
No results	No results	No results	No results		222.10045	3.923
No results	No results	No results	No results		290.14777	2.104
No results	No results	No results	No results		574.3032	18.139
No results	No results	No results	No results		277.67354	18.151
No results	No results	No results	No results		125.02259	5.988
No results	No results	No results	No results		957.60151	16.127
No results	No results	No results	No results		436.13243	2.358
No results	No results	No results	No results		185.95692	18.27
No results	No results	No results	No results		206.13696	5.699
No results	No results	No results	No results		1191.5745	9.434
No results	No results	No results	Full match		352.12685	6.088
No results	No results	No results	No results		267.07393	6.512
No results	No results	No results	No results		454.34491	11.407
No results	No results	No results	No results		616.2228	14.109
No results	No results	No results	No results		805.55724	16.288
No results	No results	No results	No results		280.14232	2.437
No results	No results	No results	No results		870.22185	3.004
No results	No results	No results	No results		222.63407	5.49
No results	No results	No results	No results		1492.9817	15.911
No results	No results	No results	No results		390.09245	7.502
No results	No results	No results	No results		219.13101	18.181
No results	No results	No results	Full match		482.08464	7.605
No results	No results	No results	No results		947.71294	17.324
No results	No results	No results	Full match		634.13229	7.422
No results	No results	No results	No results		415.14797	2.1
No results	No results	No results	No results		598.12965	8.713
No results	No results	No results	No results		791.54277	18.07
No results	No results	No results	No results		221.06863	6.447
No results	No results	No results	No results		674.22724	5.869
No results	No results	No results	No results		558.18511	6.163
No results	No results	No results	Full match		334.06679	2.109
No results	Partial matcl	No results	Full match		270.16158	18.143
No results	No results	No results	No results		227.11509	8.569
No results	No results	No results	No results		144.02117	3.358

No results	No results	No results	Full match		504.12663	6.839
No results	No results	No results	No results		190.05019	0.982
No results	No results	No results	No results		366.0956	7.52
No results	No results	No results	No results		1696.2611	7.632
No results	No results	No results	No results		714.3446	9.842
No results	No results	No results	No results		106.06034	18.162
No results	No results	No results	No results		280.14241	3.677
No results	No results	No results	No results		291.06323	7.441
No results	No results	No results	No results		162.07781	6.115
No results	No results	No results	No results		455.27327	8.822
No results	No results	No results	No results		280.05797	7.623
No results	No results	No results	No results		675.15605	13.722
No results	No results	No results	No results		1148.5608	8.796
No results	No results	No results	No results		601.38694	18.14
No results	No results	No results	No results		636.11225	6.891
No results	No results	No results	No results		610.2753	10.102
No results	No results	No results	Full match		207.11106	18.178
No results	No results	No results	No results		295.18983	17.859
No results	No results	No results	No results		469.13082	1.505
No results	No results	No results	No results		552.10896	4.937
No results	No results	No results	No results		321.09605	14.608
No results	No results	No results	Full match		418.32383	8.668
No results	No results	No results	Full match		472.17317	7.653
No results	No results	No results	No results		507.33244	13.715
No results	No results	No results	No results		316.89294	3.692
No results	No results	No results	No results		761.49171	18.051
No results	No results	No results	No results		995.45812	14.473
No results	No results	No results	No results		437.33756	12.65
No results	No results	No results	No results		255.79091	18.221
No results	Partial match	No results	Full match		314.07892	7.413
No results	No results	No results	No results		531.37798	14.659
No results	No results	No results	No results		580.31184	12.563
No results	No results	No results	Full match		230.14133	6.032
No results	No results	No results	No results		334.21208	11.607
No results	No results	No results	No results		305.27165	16.168
No results	No results	No results	No results		264.07226	2.702
No results	No results	No results	No results		588.12673	7.511
No results	No results	No results	No results		530.23345	7.495
No results	Full match	No results	No results		440.36614	14.784
No results	No results	No results	No results		111.06758	1.412
No results	No results	No results	No results		402.17845	17.242
No results	No results	No results	No results		554.10597	7.214
No results	No results	No results	No results		363.33481	16.934
No results	No results	No results	No results		459.31993	10.458
No results	No results	No results	No results		159.08835	4.191
No results	No results	No results	No results		1322.6292	9.546
No results	No results	No results	No results		530.16545	6.167
No results	No results	No results	No results		1537.0104	15.811
No results	No results	No results	No results		342.32475	12.052
No results	No results	No results	No results		617.42889	16.021

No results	No results	No results	No results		470.23583	8.11
No results	No results	No results	No results		514.36623	16.097
No results	No results	No results	No results		489.09056	7.089
No results	No results	No results	No results		347.16962	2.121
No results	Partial match	No results	Full match		390.09465	8.115
No results	No results	No results	Full match		692.23146	6.889
No results	No results	No results	No results		972.55757	15.276
No results	No results	No results	No results		221.0688	1.622
No results	No results	No results	Full match		465.3094	10.759
No results	No results	No results	No results		756.5489	17.388
No results	No results	No results	No results		958.46903	5.587
No results	No results	No results	Full match		332.08934	7.53
No results	No results	No results	No results		728.41942	15.057
No results	No results	No results	No results		317.09294	10.067
No results	No results	No results	No results		724.51076	18.162
No results	No results	No results	No results		495.15902	2.861
No results	No results	No results	No results		228.02638	2.666
No results	No results	No results	No results		1122.6729	7.467
No results	No results	No results	No results		906.52684	17.994
No results	No results	No results	No results		845.51622	18.066
No results	No results	No results	Full match		440.07391	8.792
No results	No results	No results	No results		403.09807	6.664
No results	No results	No results	No results		527.14305	6.121
No results	No results	No results	Full match		384.23735	1.772
No results	No results	No results	No results		928.24818	6.122
No results	No results	No results	Full match		512.16788	8.66
No results	No results	No results	No results		514.09474	1.181
No results	Full match	No results	Full match		155.06985	2.062
No results	No results	No results	Full match		476.09529	5.771
No results	No results	No results	No results		165.93345	1.852
No results	No results	No results	No results		294.11018	7.264
No results	No results	No results	No results		588.12663	8.028
No results	No results	No results	No results		623.35432	13.448
No results	No results	No results	No results		255.18349	4.577
No results	No results	No results	No results		454.34456	7.351
No results	No results	No results	No results		598.12965	8.875
No results	No results	No results	No results		237.022	4.741
No results	No results	No results	No results		317.01918	2.844
No results	No results	No results	No results		776.23752	4.198
No results	No results	No results	No results		437.33774	11.418
No results	No results	No results	No results		244.05141	5.829
No results	No results	No results	No results		461.15431	5.982
No results	No results	No results	No results		188.08792	1.116
No results	No results	No results	No results		350.01496	4.014
No results	No results	No results	No results		328.30935	11.432
No results	No results	No results	No results		243.03718	1.586
No results	No results	No results	No results		414.02388	2.603
No results	No results	No results	No results		820.53071	14.854
No results	No results	No results	No results		190.1037	1.935
No results	No results	No results	Full match		216.13544	6.938

No results	Partial matcl	No results	Full match		228.07419	1.424
No results	No results	No results	No results		689.50722	18.024
No results	No results	No results	No results		313.16366	5.934
No results	No results	No results	No results		630.35959	4.453
No results	No results	No results	No results		627.1881	15.171
No results	No results	No results	Full match		306.18317	11.308
No results	No results	No results	No results		286.06624	3.263
No results	Partial matcl	No results	Full match		259.15706	15.171
No results	No results	No results	Full match		410.31856	14.515
No results	No results	No results	No results		1132.529	8.064
No results	No results	No results	No results		805.56102	15.027
No results	No results	No results	No results		478.24199	7.698
No results	No results	No results	No results		433.13634	7.137
No results	No results	No results	No results		324.14174	3.034
No results	No results	No results	No results		614.1328	18.032
No results	No results	No results	No results		194.12212	5.732
No results	No results	No results	No results		326.20842	8.944
No results	Partial matcl	No results	Full match		356.11088	2.699
No results	No results	No results	Full match		460.13628	6.97
No results	No results	No results	No results		270.15984	18.177
No results	No results	No results	No results		318.09291	7.029
No results	No results	No results	Full match		342.14668	7.362
No results	No results	No results	No results		721.40848	5.944
No results	No results	No results	Full match		204.11521	8.159
No results	No results	No results	No results		258.07359	4.101
No results	No results	No results	No results		569.33351	7.569
No results	No results	No results	No results		1146.545	8.116
No results	No results	No results	No results		592.15843	7.043
No results	No results	No results	No results		1180.5665	10.061
No results	No results	No results	No results		419.24434	18.205
No results	No results	No results	No results		255.1682	5.885
No results	No results	No results	Full match		349.11634	7.307
No results	No results	No results	No results		265.02634	4.564
No results	No results	No results	No results		389.08272	7.489
No results	No results	No results	Full match		276.15883	6.006
No results	No results	No results	No results		650.12698	6.967
No results	No results	No results	No results		186.14813	1.514
No results	Partial matcl	No results	Full match		154.02655	6.251
No results	No results	No results	No results		229.48174	18.166
No results	No results	No results	No results		496.35566	8.67
No results	No results	No results	No results		233.8985	18.175
No results	No results	No results	No results		468.81136	5.857
No results	No results	No results	No results		1011.364	6.085
No results	No results	No results	No results		488.2534	15.085
No results	No results	No results	No results		281.1265	6.207
No results	No results	No results	Full match		430.08943	2.322
No results	No results	No results	No results		620.1168	8.182
No results	No results	No results	No results		274.04399	1.197
No results	No results	No results	Full match		197.03255	2.072
No results	No results	No results	No results		480.23088	16.963

No results	No results	No results	No results		1021.3855	5.967
No results	No results	No results	Full match		400.11547	8.2
No results	No results	No results	No results		618.43275	15.962
No results	No results	No results	No results		308.08961	8.02
No results	No results	No results	Full match		586.24121	7.864
No results	No results	No results	No results		718.11506	7.505
No results	No results	No results	Full match		636.1476	7.475
No results	No results	No results	No results		449.97824	2.594
No results	No results	No results	No results		572.29687	18.099
No results	No results	No results	No results		425.85006	18.167
No results	No results	No results	No results		141.99447	3.259
No results	No results	No results	No results		249.16236	5.622
No results	No results	No results	No results		595.13264	7.541
No results	No results	No results	No results		733.26402	6.161
No results	No results	No results	Full match		315.1147	5.198
No results	No results	No results	No results		468.56032	5.873
No results	No results	No results	No results		1174.5777	8.725
No results	No results	No results	No results		293.20712	13.934
No results	No results	No results	Full match		724.23785	13.3
No results	No results	No results	No results		249.89392	0.732
No results	No results	No results	No results		426.26481	16.339
No results	No results	No results	Full match		1076.5398	8.473
No results	No results	No results	No results		1175.6099	10.531
No results	No results	No results	Full match		610.09604	7.389
No results	No results	No results	Full match		408.12047	8.81
No results	No results	No results	Full match		282.14635	7.736
No results	No results	No results	No results		292.04865	2.987
No results	No results	No results	Full match		584.1169	7.081
No results	No results	No results	No results		706.47871	18.041
No results	No results	No results	Full match		298.25037	14.449
No results	No results	No results	No results		316.10665	2.26
No results	No results	No results	No results		625.14306	5.887
No results	No results	No results	No results		165.08369	8.669
No results	No results	No results	No results		567.31745	7.283
No results	No results	No results	No results		165.01052	3.25
No results	No results	No results	No results		818.53975	18.16
No results	No results	No results	No results		124.05112	5.582
No results	No results	No results	No results		827.53349	14.487
No results	No results	No results	No results		522.3562	7.775
No results	No results	No results	Full match		552.12632	8.431
No results	No results	No results	No results		263.15163	6.029
No results	No results	No results	No results		247.89316	1.878
No results	No results	No results	Full match		163.06334	5.896
No results	No results	No results	No results		206.01034	3.423
No results	No results	No results	No results		691.1221	4.553
No results	No results	No results	No results		472.3558	13.479
No results	No results	No results	No results		418.11	6.148
No results	No results	No results	Full match		594.1371	8.69
No results	No results	No results	No results		486.0784	8.292
No results	No results	No results	No results		362.2065	13.41

No results	No results	No results	No results		488.12938	7.266
No results	Partial matcl	No results	Full match		282.25552	17.168
No results	No results	No results	Full match		410.13651	7.389
No results	No results	No results	No results		272.13705	5.357
No results	No results	No results	No results		419.24427	5.449
No results	No results	No results	Full match		436.10008	3.079
No results	No results	No results	No results		216.45545	5.654
No results	No results	No results	No results		819.58647	18.06
No results	No results	No results	No results		500.22402	7.518
No results	No results	No results	No results		280.14235	6.161
No results	No results	No results	Full match		578.14298	7.962
No results	No results	No results	No results		117.08004	0.948
No results	No results	No results	Full match		362.19391	8.479
No results	No results	No results	No results		620.11639	7.757
No results	No results	No results	Full match		334.04782	5.758
No results	No results	No results	No results		252.209	9.644
No results	No results	No results	No results		709.38916	9.834
No results	No results	No results	No results		959.67625	17.178
No results	No results	No results	Full match		416.11	7.283
No results	No results	No results	Full match		311.10067	2.537
No results	No results	No results	No results		1190.572	7.909
No results	No results	No results	No results		179.16751	9.12
No results	No results	No results	No results		1391.9273	17.331
Invalid mass	No results	No results	No results		154.06184	4.444
No results	No results	No results	No results		191.99771	18.446
No results	No results	No results	No results		767.51103	18.077
No results	No results	No results	No results		893.46644	14.406
No results	No results	No results	Full match		422.12132	3.887
No results	No results	No results	No results		1172.5758	12.891
No results	No results	No results	Full match		392.12562	8.745
No results	No results	No results	No results		366.27662	15.173
No results	No results	No results	No results		662.26738	13.2
No results	No results	No results	No results		317.14753	2.012
No results	No results	No results	Full match		229.16795	8.356
No results	No results	No results	No results		508.14837	6.119
No results	No results	No results	No results		750.53212	18.053
No results	No results	No results	Full match		418.32379	9.868
No results	No results	No results	No results		392.22304	17.479
No results	No results	No results	No results		566.32995	12.547
No results	Full match	No results	No results		189.87963	5.673
No results	No results	No results	No results		367.1054	7.353
No results	Partial matcl	No results	Full match		438.35016	9.945
No results	No results	No results	No results		118.9895	4.774
No results	No results	No results	No results		495.15978	5.299
No results	No results	No results	No results		265.01667	4.22
No results	Partial matcl	No results	Full match		152.04737	4.556
No results	No results	No results	No results		419.0475	11.377
No results	No results	No results	No results		696.22138	15.071
No results	No results	No results	No results		199.15724	8.444
No results	No results	No results	No results		588.12668	7.825

No results	No results	No results	No results		254.04247	1.592
No results	No results	No results	No results		256.16731	9.169
No results	No results	No results	No results		1322.6293	8.31
No results	No results	No results	No results		793.26483	6.036
No results	No results	No results	No results		752.30443	7.339
No results	No results	No results	No results		1098.3858	5.946
No results	No results	No results	No results		477.09089	7.54
No results	No results	No results	No results		299.13719	5.993
No results	No results	No results	No results		260.13718	5.956
No results	No results	No results	No results		560.13156	8.458
No results	No results	No results	No results		1380.6339	9.249
No results	No results	No results	No results		239.63518	5.842
No results	No results	No results	No results		310.9874	3.982
No results	No results	No results	Full match		184.18286	8.673
No results	No results	No results	No results		200.62622	5.623
No results	No results	No results	No results		651.49187	17.399
No results	No results	No results	No results		558.11842	6.869
No results	No results	No results	No results		1158.5818	10.815
No results	No results	No results	Full match		224.06841	6.057
No results	No results	No results	No results		715.51552	18.2
No results	No results	No results	Full match		200.10506	7.314
No results	No results	No results	No results		396.25065	11.735
No results	No results	No results	No results		1099.6234	14.387
No results	No results	No results	No results		436.36647	14.124
No results	No results	No results	No results		425.14778	7.52
No results	No results	No results	No results		182.13086	7.504
No results	No results	No results	No results		413.22022	14.633
No results	No results	No results	No results		696.22151	14.744
No results	No results	No results	No results		341.16872	5.909
No results	No results	No results	No results		310.0226	5.83
No results	No results	No results	No results		271.28765	12.208
No results	No results	No results	No results		609.32515	13.69
No results	No results	No results	Full match		442.12599	8.725
No results	No results	No results	Full match		566.1421	7.658
No results	Partial matcl	No results	Full match		458.12077	7.569
No results	No results	No results	No results		166.03669	1.581
No results	No results	No results	No results		191.10497	5.46
No results	No results	No results	No results		513.03612	7.111
No results	No results	No results	No results		1386.6239	9.236
No results	No results	No results	Full match		512.16789	8.362
No results	No results	No results	No results		900.49749	18.084
No results	No results	No results	No results		213.04875	3.234
No results	No results	No results	Full match		266.16472	14.175
No results	No results	No results	Full match		518.1917	6.005
No results	No results	No results	No results		454.34502	10.738
No results	No results	No results	No results		588.14802	7.15
No results	No results	No results	No results		581.33265	10.695
No results	No results	No results	No results		163.76398	18.153
No results	No results	No results	No results		214.98635	0.787
No results	No results	No results	Full match		274.1237	10.3

No results	No results	No results	No results		307.0367	3.081
No results	No results	No results	No results		507.33163	14.399
No results	No results	No results	Full match		290.06358	2.843
No results	No results	No results	No results		120.91351	18.252
No results	No results	No results	No results		584.38717	11.718
No results	No results	No results	No results		599.35453	14.002
No results	No results	No results	No results		215.15217	7.667
No results	No results	No results	No results		453.1351	2.848
No results	Full match	No results	No results		632.24977	13.721
No results	No results	No results	No results		148.94567	0.792
No results	No results	No results	No results		399.29848	14.835
No results	No results	No results	No results		500.37575	10.495
No results	No results	No results	No results		335.23889	16.465
No results	No results	No results	No results		201.04267	1.972
No results	No results	No results	No results		1058.5294	9.808
No results	No results	No results	No results		315.17983	4.477
No results	No results	No results	No results		639.28379	14.396
No results	No results	No results	No results		484.2529	5.941
No results	No results	No results	No results		969.60271	16.114
No results	No results	No results	Full match		288.10304	7.111
No results	No results	No results	Full match		568.15825	6.982
No results	No results	No results	No results		453.10588	6.157
No results	No results	No results	No results		619.44457	16.862
No results	No results	No results	No results		244.12863	2.086
No results	No results	No results	No results		562.29872	9.86
No results	No results	No results	No results		1322.6292	10.005
No results	No results	No results	No results		1352.6391	10.285
No results	No results	No results	No results		589.11702	1.66
No results	No results	No results	No results		192.12644	5.994
No results	No results	No results	No results		514.20311	7.483
No results	No results	No results	No results		533.31197	10.727
No results	No results	No results	No results		501.16405	8.365
No results	No results	No results	No results		435.08763	6.887
No results	No results	No results	No results		1338.6242	9.224
No results	No results	No results	No results		508.27372	5.673
No results	No results	No results	No results		274.02681	4.763
No results	No results	No results	No results		244.13064	7.727
No results	Partial matcl	No results	Full match		357.10576	6.077
No results	No results	No results	No results		179.98265	1.201
No results	No results	No results	No results		809.52245	14.739
No results	No results	No results	No results		210.16217	8
No results	No results	No results	No results		750.40339	13.836
No results	No results	No results	No results		1158.5819	11.018
No results	No results	No results	No results		306.91606	18.164
No results	No results	No results	No results		656.21675	17.517
No results	No results	No results	No results		293.07691	3.584
No results	No results	No results	No results		606.18954	15.1
No results	No results	No results	No results		432.22694	18.198
No results	No results	No results	No results		350.03135	5.254
No results	No results	No results	Full match		316.26093	13.393

No results	No results	No results	Full match		124.08908	9.422
No results	No results	No results	No results		313.08797	5.258
No results	No results	No results	Full match		488.10756	2.831
No results	No results	No results	No results		231.92096	1.302
No results	No results	No results	No results		222.63446	18.226
No results	No results	No results	Full match		578.38167	12.792
No results	No results	No results	No results		626.39762	11.697
No results	No results	No results	Full match		436.13717	5.973
No results	No results	No results	No results		576.20289	8.074
No results	No results	No results	No results		598.12995	8.639
No results	No results	No results	Full match		446.23001	12.032
No results	No results	No results	No results		318.00194	3.251
No results	No results	No results	No results		1368.6348	9.954
No results	No results	No results	No results		194.07665	0.997
No results	No results	No results	No results		820.28395	5.911
No results	No results	No results	No results		168.93304	18.245
No results	No results	No results	No results		598.12989	8.811
No results	Partial matcl	No results	Full match		134.02218	1.426
No results	No results	No results	No results		781.52455	18.052
No results	No results	No results	Full match		424.07924	9.638
No results	Full match	No results	No results		630.23427	14.571
No results	No results	No results	No results		626.10237	2.534
No results	No results	No results	Full match		318.05821	2.307
No results	No results	No results	No results		373.1931	13.621
No results	No results	No results	No results		243.14652	10.734
No results	No results	No results	No results		308.69159	5.679
No results	No results	No results	No results		1090.5162	6.982
No results	No results	No results	Full match		691.25401	3.45
No results	No results	No results	No results		363.31362	12.729
No results	No results	No results	No results		406.32411	12.443
No results	Partial matcl	No results	Full match		310.21445	11.737
No results	No results	No results	No results		438.12724	6.649
No results	No results	No results	No results		190.0002	18.08
No results	Partial matcl	No results	Full match		166.0266	6.022
No results	No results	No results	No results		1117.7674	17.997
No results	No results	No results	No results		291.16237	9.008
No results	No results	No results	No results		560.13165	8.341
No results	No results	No results	No results		143.07205	6.106
No results	No results	No results	No results		228.06121	2.118
No results	Partial matcl	No results	Full match		584.22578	8.609
No results	Partial matcl	No results	Full match		236.11632	5.848
No results	No results	No results	No results		204.07771	7.543
No results	No results	No results	No results		449.97823	2.826
No results	No results	No results	No results		1160.5599	10.016
No results	No results	No results	Full match		224.10419	8.654
No results	No results	No results	No results		582.28248	10.927
No results	No results	No results	No results		163.05219	6.039
No results	No results	No results	No results		261.96435	2.234
No results	No results	No results	No results		475.31866	14.086
No results	No results	No results	Full match		372.08415	8.467

No results	No results	No results	Full match		372.084	8.543
No results	No results	No results	Full match		756.18916	7.443
No results	No results	No results	No results		343.13765	3.388
No results	No results	No results	Full match		274.12358	10.239
No results	No results	No results	No results		1054.5168	15.288
No results	No results	No results	No results		398.23323	17.92
No results	No results	No results	No results		171.08845	5.065
No results	No results	No results	Full match		332.08974	6.292
No results	No results	No results	No results		145.11432	13.718
No results	No results	No results	Full match		376.07903	7.639
No results	No results	No results	No results		452.06274	7.477
No results	No results	No results	Full match		582.13496	9.165
No results	No results	No results	No results		1126.5557	11.055
No results	No results	No results	No results		263.07752	4.217
No results	No results	No results	No results		764.08721	4.549
No results	No results	No results	No results		240.02654	3.344
No results	No results	No results	Full match		225.13661	1.488
No results	No results	No results	No results		275.07925	3.619
No results	No results	No results	No results		200.62638	18.14
No results	No results	No results	Full match		586.35066	18.154
No results	No results	No results	No results		587.37335	5.741
No results	No results	No results	No results		374.19424	8.295
No results	Partial matcl	No results	Full match		205.07395	6.769
No results	No results	No results	No results		524.2466	7.698
No results	No results	No results	No results		712.46724	18.133
No results	No results	No results	No results		453.28529	16.401
No results	No results	No results	No results		993.35435	5.896
No results	No results	No results	No results		311.06169	2.096
No results	No results	No results	No results		207.46097	5.673
No results	No results	No results	No results		404.0379	4.836
No results	No results	No results	No results		179.4465	18.159
No results	No results	No results	No results		361.11808	6.001
No results	No results	No results	Full match		474.11589	7.341
No results	No results	No results	No results		779.50993	18.076
No results	No results	No results	Full match		538.2256	7.493
No results	No results	No results	No results		696.22114	15.443
No results	No results	No results	No results		197.14173	5.465
No results	No results	No results	No results		195.01584	3.648
No results	No results	No results	No results		620.26906	10.883
No results	No results	No results	No results		364.26161	15.92
No results	No results	No results	No results		263.16066	5.707
No results	No results	No results	No results		302.06077	8.547
No results	No results	No results	No results		472.35536	15.519
No results	No results	No results	Full match		448.26668	8.856
No results	No results	No results	No results		972.55812	15.526
No results	No results	No results	No results		492.22061	7.477
No results	No results	No results	No results		318.14241	4.24
No results	No results	No results	No results		454.34497	15.521
No results	Partial matcl	No results	Full match		390.09521	8.312
No results	No results	No results	No results		512.29905	9.86

No results	No results	No results	No results		439.11961	4.884
No results	Partial matcl	No results	Full match		356.08927	8.757
No results	No results	No results	Full match		578.38154	12.218
No results	No results	No results	No results		411.13222	6.116
No results	No results	No results	No results		490.31444	14.047
No results	No results	No results	No results		1143.5835	11.053
No results	No results	No results	No results		1074.5612	9.395
No results	No results	No results	No results		237.80673	18.154
No results	No results	No results	No results		255.07441	2.566
No results	No results	No results	No results		873.48121	14.493
No results	No results	No results	Full match		506.11802	8.889
No results	No results	No results	Full match		568.12092	6.825
No results	No results	No results	No results		1180.5663	10.653
No results	No results	No results	No results		191.00795	18.066
No results	No results	No results	No results		309.20186	10.508
No results	No results	No results	Full match		466.12595	7.284
No results	No results	No results	No results		238.15635	11.593
No results	No results	No results	No results		562.29859	10.659
No results	No results	No results	No results		454.34517	7.917
No results	No results	No results	No results		341.08252	5.462
No results	No results	No results	No results		247.12485	2.007
No results	No results	No results	No results		587.30754	13.448
No results	No results	No results	No results		171.9685	1.273
No results	No results	No results	No results		420.08995	2.886
No results	No results	No results	No results		331.17459	5.895
No results	No results	No results	No results		378.17151	13.561
No results	No results	No results	No results		606.18965	14.738
No results	No results	No results	No results		350.20714	9.481
No results	No results	No results	No results		1074.5246	7.702
No results	No results	No results	No results		162.05434	3.903
No results	No results	No results	No results		238.13172	6.137
No results	Partial matcl	No results	Full match		328.09434	7.775
No results	No results	No results	No results		239.95961	0.733
No results	No results	No results	No results		613.17204	5.281
No results	No results	No results	No results		342.68547	5.806
No results	No results	No results	No results		687.51347	15.553
No results	No results	No results	No results		1108.7818	17.065
No results	No results	No results	No results		193.06154	6.749
No results	No results	No results	No results		446.25116	8.72
No results	No results	No results	Full match		482.12174	7.29
No results	No results	No results	No results		536.31944	14.044
No results	No results	No results	No results		794.30147	12.389
No results	No results	No results	No results		369.1057	3.125
No results	No results	No results	No results		243.81806	18.177
No results	No results	No results	No results		149.04676	5.848
No results	No results	No results	No results		293.2075	8.908
No results	No results	No results	No results		1104.5348	7.493
No results	No results	No results	No results		964.48189	18.165
No results	No results	No results	No results		819.52266	18.02
No results	No results	No results	No results		604.33036	7.822

No results	No results	No results	No results		528.52373	13.264
No results	No results	No results	No results		758.52821	18.036
No results	No results	No results	Full match		563.18001	7.132
No results	No results	No results	Full match		310.10553	7.161
No results	No results	No results	No results		237.94313	18.407
No results	No results	No results	No results		602.16442	2.463
No results	No results	No results	No results		572.07771	8.313
No results	No results	No results	Full match		213.24587	10.781
No results	No results	No results	No results		571.14259	4.542
No results	No results	No results	No results		452.86904	18.157
No results	No results	No results	No results		310.0581	7.795
No results	No results	No results	No results		234.1573	5.654
No results	No results	No results	No results		146.09311	7.588
No results	No results	No results	No results		235.10588	1.555
No results	No results	No results	No results		148.97959	18.256
No results	No results	No results	No results		511.15512	1.586
No results	No results	No results	No results		288.04878	3.276
No results	No results	No results	No results		266.88428	0.09
No results	No results	No results	No results		587.40239	14.941
No results	No results	No results	No results		195.03835	3.205
No results	No results	No results	No results		666.23164	6.801
No results	No results	No results	No results		311.91301	0.77
No results	No results	No results	No results		760.16365	7.053
No results	No results	No results	No results		602.14821	3.464
No results	No results	No results	No results		1118.5502	9.18
No results	No results	No results	Full match		504.12685	7.076
No results	No results	No results	No results		950.63571	14.118
No results	No results	No results	No results		554.27578	15.054
No results	No results	No results	No results		210.14104	11.04
No results	No results	No results	No results		255.03276	4.768
No results	No results	No results	No results		448.07905	7.671
No results	No results	No results	No results		1358.6054	10.381
No results	No results	No results	No results		646.19032	7.616
No results	No results	No results	No results		473.25444	9.748
No results	No results	No results	No results		502.32899	11.756
No results	No results	No results	No results		624.18988	3.515
No results	Partial matcl	No results	Full match		148.03695	1.414
No results	No results	No results	Full match		536.38699	10.726
No results	No results	No results	No results		602.16406	4.396
No results	No results	No results	No results		284.17389	5.827
No results	No results	No results	No results		592.30876	9.492
No results	No results	No results	No results		532.32444	15.236
No results	No results	No results	Full match		656.4287	11.048
No results	No results	No results	No results		393.53838	5.376
No results	No results	No results	No results		362.15799	8.189
No results	No results	No results	No results		606.23399	14.99
No results	No results	No results	Full match		254.18821	9.915
No results	No results	No results	Full match		250.13173	5.97
No results	No results	No results	No results		576.12929	7.131
No results	No results	No results	No results		468.06718	7.263

No results	No results	No results	No results		892.46334	18.09
No results	No results	No results	No results		466.08958	8.098
No results	No results	No results	No results		215.63558	18.182
No results	No results	No results	No results		903.25148	7.862
No results	No results	No results	No results		714.48085	18.106
No results	No results	No results	No results		250.12354	6.075
No results	No results	No results	No results		202.11187	5.698
No results	No results	No results	No results		726.25272	7.63
No results	No results	No results	No results		457.10085	6.814
No results	No results	No results	No results		443.97731	17.987
No results	No results	No results	No results		1163.5735	8.087
No results	No results	No results	No results		569.36912	15.309
No results	No results	No results	No results		131.09865	13.716
No results	No results	No results	No results		549.30717	8.865
No results	No results	No results	No results		189.0513	1.048
No results	No results	No results	No results		185.17801	11.493
No results	No results	No results	No results		230.04269	1.589
No results	No results	No results	No results		411.24785	5.574
No results	No results	No results	No results		148.03948	2.088
No results	No results	No results	No results		724.38826	12.912
No results	No results	No results	No results		528.08772	7.506
No results	No results	No results	No results		1174.5553	11.777
No results	No results	No results	No results		278.89123	3.303
No results	No results	No results	No results		1044.5134	8.131
No results	Partial matcl	No results	Full match		464.09566	7.213
No results	No results	No results	No results		454.34462	13.481
No results	No results	No results	No results		292.88368	3.242
No results	No results	No results	No results		455.02421	11.363
No results	No results	No results	No results		377.24135	16.466
No results	No results	No results	No results		210.13923	6.095
No results	No results	No results	No results		228.96668	3.256
No results	No results	No results	No results		611.42592	18.185
No results	No results	No results	No results		225.13483	5.755
No results	No results	No results	No results		277.24065	12.805
No results	No results	No results	No results		248.93033	18.415
No results	No results	No results	No results		264.02843	7.038
No results	No results	No results	No results		315.69972	5.764
No results	No results	No results	No results		632.18925	8.494
No results	No results	No results	Full match		550.27611	11.112
No results	No results	No results	No results		566.2878	11.657
No results	No results	No results	Full match		350.24565	14.154
No results	No results	No results	No results		498.13786	2.873
No results	No results	No results	No results		145.93002	0.078
No results	No results	No results	Full match		175.04818	1.535
No results	No results	No results	No results		598.09572	7.271
No results	No results	No results	No results		737.49614	18.059
No results	Full match	No results	No results		294.0742	2.764
No results	Partial matcl	No results	Full match		294.21922	12.896
No results	No results	No results	Full match		316.26093	13.052
No results	No results	No results	No results		512.35069	11.201

No results	No results	No results	No results		630.19563	7.792
No results	No results	No results	No results		294.12163	1.99
No results	No results	No results	Full match		482.12096	7.936
No results	Partial match	No results	Full match		548.08308	2.624
No results	No results	No results	No results		291.07426	8.18
No results	No results	No results	No results		554.37647	11.463
No results	No results	No results	No results		760.58025	13.789
No results	No results	No results	No results		712.26527	18.174
No results	No results	No results	No results		210.12953	5.582
No results	Partial match	No results	Full match		148.03751	2.085
No results	No results	No results	No results		389.0827	7.617
No results	No results	No results	No results		578.29414	8.132
No results	No results	No results	No results		461.10414	7.268
No results	No results	No results	No results		203.04317	1.652
No results	No results	No results	No results		361.11617	5.999
No results	No results	No results	No results		437.33767	10.693
No results	No results	No results	No results		306.93376	18.158
No results	No results	No results	No results		511.08217	7.198
No results	No results	No results	No results		472.09184	7.784
No results	No results	No results	No results		163.09204	18.145
No results	No results	No results	No results		482.29407	5.756
No results	Partial match	No results	Full match		356.11015	6.239
No results	No results	No results	Full match		366.00459	5.643
No results	No results	No results	No results		222.00076	4.219
No results	No results	No results	No results		400.3131	10.736
No results	Partial match	No results	Full match		386.0998	8.103
No results	No results	No results	No results		201.60871	5.588
No results	No results	No results	No results		566.369	15.311
No results	No results	No results	No results		644.40817	11.634
No results	No results	No results	No results		455.34841	10.751
No results	No results	No results	No results		1050.5738	15.284
No results	No results	No results	No results		452.27692	14.662
No results	No results	No results	No results		1050.5744	15.516
No results	No results	No results	Full match		410.09989	7.75
No results	No results	No results	No results		257.05784	3.596
No results	No results	No results	No results		313.23329	11.621
No results	No results	No results	No results		453.28526	16.672
No results	No results	No results	Full match		424.07872	7.716
No results	No results	No results	No results		698.3723	12.623
No results	No results	No results	No results		450.85213	18.145
No results	No results	No results	No results		226.13869	5.558
No results	No results	No results	No results		528.26015	13.939
No results	No results	No results	No results		596.40792	10.759
No results	No results	No results	No results		542.10597	2.314
No results	No results	No results	Full match		462.22444	9.826
No results	No results	No results	No results		488.24118	11.526
No results	No results	No results	No results		165.07801	6.968
No results	No results	No results	No results		411.99041	18.083
No results	No results	No results	No results		377.11137	8.138
No results	No results	No results	No results		233.15002	5.553

No results	No results	No results	No results		169.04578	4.539
No results	No results	No results	No results		519.10187	5.353
No results	No results	No results	No results		671.29585	14.006
No results	No results	No results	No results		239.07886	2.768
No results	Partial matcl	No results	Full match		310.21445	8.913
No results	No results	No results	No results		465.52654	16.129
No results	No results	No results	Full match		264.17222	12.81
No results	No results	No results	No results		226.13402	5.792
No results	No results	No results	Full match		480.08845	7.162
No results	No results	No results	No results		644.40721	11.354
No results	No results	No results	No results		376.15247	7.942
No results	No results	No results	No results		167.05705	5.856
No results	No results	No results	No results		810.46577	18.076
No results	No results	No results	Full match		271.14198	2.511
No results	No results	No results	No results		650.09555	4.545
No results	No results	No results	No results		950.5732	15.902
No results	No results	No results	No results		334.90187	18.226
No results	No results	No results	No results		612.10308	14.508
No results	No results	No results	Full match		484.12145	4.918
No results	No results	No results	No results		216.11998	5.653
No results	No results	No results	No results		1143.5834	11.35
No results	No results	No results	No results		185.17801	11.723
No results	No results	No results	No results		324.95753	0.706
No results	Full match	No results	No results		474.09263	4.899
No results	No results	No results	No results		603.13303	4.143
No results	No results	No results	No results		248.1546	5.809
No results	No results	No results	No results		709.38835	9.735
No results	Full match	No results	No results		614.23891	18.175
No results	No results	No results	No results		396.14491	8.63
No results	No results	No results	No results		252.98996	1.2
No results	No results	No results	No results		1091.5527	7.704
No results	No results	No results	No results		490.08782	2.804
No results	No results	No results	No results		376.15246	7.125
No results	No results	No results	No results		295.66142	5.335
No results	No results	No results	Full match		362.1944	7.244
No results	No results	No results	No results		263.86534	0.081
No results	No results	No results	Full match		366.00403	5.262
No results	No results	No results	Full match		228.14677	5.127
No results	No results	No results	No results		228.15147	10.758
No results	No results	No results	No results		1374.6013	10.037
No results	No results	No results	No results		484.19427	7.167
No results	Full match	No results	Full match		208.08495	2.687
No results	No results	No results	No results		603.40134	12.513
No results	No results	No results	No results		1132.5449	11.737
No results	Partial matcl	No results	Full match		272.06841	8.253
No results	No results	No results	No results		680.78667	11.598
No results	No results	No results	No results		1361.5725	11.653
No results	No results	No results	No results		546.30452	11.002
No results	No results	No results	No results		305.05335	6.971
No results	No results	No results	No results		195.07809	7.269

No results	No results	No results	No results		526.15965	6.101
No results	No results	No results	No results		382.26988	8.45
No results	No results	No results	No results		347.67848	5.666
No results	No results	No results	Full match		358.14125	7.969
No results	No results	No results	No results		323.28249	12.747
No results	No results	No results	No results		127.94267	18.237
No results	No results	No results	No results		483.13735	2.823
No results	No results	No results	No results		233.05576	2.813
No results	No results	No results	No results		509.28325	17.212
No results	Partial matcl	No results	Full match		197.06802	2.337
No results	No results	No results	No results		228.02777	2.675
No results	No results	No results	No results		406.16414	7.452
No results	No results	No results	No results		199.10636	6.09
No results	No results	No results	No results		531.13667	4.842
No results	No results	No results	No results		303.14457	2.042
No results	No results	No results	No results		476.31682	17.16
No results	No results	No results	No results		874.45126	18.166
No results	No results	No results	No results		511.22594	8.088
No results	No results	No results	No results		776.23706	3.221
No results	No results	No results	No results		368.92283	3.439
No results	No results	No results	No results		140.92767	1.339
No results	No results	No results	No results		1082.7655	16.87
No results	No results	No results	No results		270.1496	5.81
No results	Partial matcl	No results	Full match		160.03694	1.431
No results	No results	No results	No results		146.07609	2.227
No results	No results	No results	No results		596.14372	6.001
No results	No results	No results	No results		1336.6454	10.36
No results	No results	No results	Full match		188.08776	1.536
No results	No results	No results	No results		521.76979	0.079
No results	No results	No results	No results		1175.6103	11.017
No results	No results	No results	No results		265.09462	6.782
No results	No results	No results	No results		496.35555	11.733
No results	No results	No results	No results		497.35655	16.931
No results	No results	No results	No results		499.04054	4.509
No results	No results	No results	No results		488.15086	7.083
No results	No results	No results	No results		411.08474	7.258
No results	No results	No results	No results		1061.5409	8.892
No results	No results	No results	No results		210.16216	8.568
No results	No results	No results	No results		221.1862	8.781
No results	No results	No results	Full match		472.13612	7.614
No results	No results	No results	No results		330.18071	10.703
No results	No results	No results	No results		358.16246	7.215
No results	No results	No results	No results		260.13677	4.213
No results	No results	No results	No results		618.13723	7.951
No results	No results	No results	No results		189.97903	1.333
No results	No results	No results	No results		536.12985	9.158
No results	No results	No results	No results		882.20198	5.92
No results	No results	No results	No results		458.19163	7.61
No results	No results	No results	No results		1175.61	10.817
No results	No results	No results	Full match		424.07919	10.022

No results	No results	No results	No results		1121.5635	7.496
No results	No results	No results	Full match		342.11012	7.465
No results	No results	No results	No results		145.94116	18.191
No results	No results	No results	No results		261.66017	5.854
No results	No results	No results	No results		718.44353	12.108
No results	No results	No results	No results		255.04219	7.336
No results	No results	No results	No results		493.28009	10.461
No results	No results	No results	No results		424.17725	13.43
No results	No results	No results	Full match		602.23701	7.14
No results	No results	No results	No results		654.30861	12.236
No results	No results	No results	No results		247.96475	4.784
No results	No results	No results	Full match		163.06345	1.605
No results	No results	No results	Full match		274.12367	10.494
No results	No results	No results	No results		302.1653	7.915
No results	No results	No results	No results		476.18956	7.365
No results	No results	No results	No results		906.47768	5.476
No results	No results	No results	No results		531.13653	2.859
No results	No results	No results	No results		582.1894	17.996
No results	No results	No results	Full match		188.0318	2.213
No results	No results	No results	No results		406.32367	10.768
No results	No results	No results	No results		1448.9536	15.898
No results	Partial matcl	No results	Full match		312.23007	12.475
No results	No results	No results	No results		343.30898	13.155
No results	No results	No results	Full match		420.1039	3.733
No results	No results	No results	No results		1338.6244	9.465
No results	No results	No results	Full match		564.12678	7.697
No results	No results	No results	No results		307.70235	5.823
No results	No results	No results	Full match		213.10013	1.581
No results	No results	No results	No results		538.22031	8.333
No results	No results	No results	No results		890.4816	5.564
No results	No results	No results	No results		1651.0746	15.94
No results	No results	No results	No results		307.19539	5.615
No results	No results	No results	No results		1074.5242	7.247
No results	No results	No results	No results		578.29353	7.556
No results	No results	No results	No results		193.06276	6.751
No results	No results	No results	Full match		412.11525	6.834
No results	No results	No results	No results		297.16179	18.184
No results	No results	No results	No results		1054.517	15.516
No results	No results	No results	Full match		436.11541	8.387
No results	No results	No results	No results		306.1459	10.677
No results	No results	No results	No results		1368.6363	10.444
No results	No results	No results	No results		447.11524	7.622
No results	No results	No results	No results		501.3214	14.205
No results	No results	No results	No results		106.06111	18.142
No results	No results	No results	No results		822.49594	5.568
No results	No results	No results	No results		865.38927	12.847
No results	No results	No results	No results		544.13674	8.999
No results	No results	No results	No results		950.57234	16.125
No results	No results	No results	Full match		220.03662	7.978
No results	Partial matcl	No results	Full match		346.06852	7.792

No results	No results	No results	No results		1032.5321	15.909
No results	No results	No results	No results		220.15446	7.337
No results	No results	No results	No results		658.39216	8.421
No results	Partial match	No results	Full match		422.08449	7.738
No results	No results	No results	No results		405.11555	7.193
No results	No results	No results	No results		454.34505	9.909
No results	No results	No results	No results		588.12213	5.096
No results	No results	No results	No results		210.05399	6.108
No results	No results	No results	Full match		352.2253	7.406
No results	No results	No results	No results		200.12023	10.772
No results	No results	No results	No results		512.13156	7.74
No results	No results	No results	No results		818.5113	14.748
No results	No results	No results	No results		296.88719	0.767
No results	No results	No results	No results		290.04252	7.819
No results	No results	No results	No results		288.13189	4.758
No results	No results	No results	No results		490.12438	9.243
No results	Full match	No results	No results		321.12126	6.162
No results	No results	No results	No results		1581.0284	15.883
No results	No results	No results	No results		512.2989	9.736
No results	No results	No results	No results		1084.5447	10.568
No results	No results	No results	No results		746.14833	7.16
No results	No results	No results	No results		1074.5242	8.136
No results	No results	No results	No results		533.31188	11.004
No results	No results	No results	No results		876.53718	18.126
No results	No results	No results	No results		463.36608	1.285
No results	No results	No results	Full match		418.32357	9.505
No results	No results	No results	No results		386.15746	8.257
No results	No results	No results	Full match		440.0739	9.086
No results	No results	No results	No results		564.35072	15.146
No results	Partial match	No results	Full match		299.28245	11.731
No results	No results	No results	No results		636.23562	14.35
No results	No results	No results	No results		1135.5783	9.173
No results	No results	No results	No results		554.142	7.916
No results	No results	No results	No results		1364.6389	10.662
No results	No results	No results	Full match		330.24048	10.613
No results	No results	No results	No results		1174.5765	10.374
No results	No results	No results	No results		458.2876	14.459
No results	No results	No results	No results		714.25093	14.084
No results	No results	No results	Full match		656.42912	9.527
No results	No results	No results	Full match		288.17232	11.308
No results	No results	No results	No results		283.14238	5.244
No results	No results	No results	No results		363.13179	7.884
No results	No results	No results	No results		291.15816	1.679
No results	No results	No results	Full match		306.25607	8.51
No results	No results	No results	No results		724.13718	3.007
No results	No results	No results	Full match		518.10383	7.18
No results	No results	No results	No results		598.09987	8.957
No results	No results	No results	Full match		740.17396	9.842
No results	No results	No results	No results		768.37483	13.403
No results	No results	No results	No results		370.18556	5.883

No results	No results	No results	No results		870.2209	5.787
No results	No results	No results	No results		216.87502	0.73
No results	No results	No results	No results		563.32228	11.818
No results	No results	No results	No results		376.31301	14.627
No results	No results	No results	No results		406.29263	8.494
No results	No results	No results	No results		689.42679	14.551
No results	No results	No results	No results		1422.6455	10.527
No results	No results	No results	No results		275.88957	0.088
No results	No results	No results	No results		283.14238	5.65
No results	No results	No results	Full match		297.2667	11.405
No results	No results	No results	No results		129.97399	0.741
No results	No results	No results	No results		203.99478	1.607
No results	No results	No results	No results		650.23603	7.11
No results	No results	No results	Full match		297.2667	11.455
No results	No results	No results	No results		349.67522	5.677
No results	No results	No results	No results		270.2191	0.067
No results	No results	No results	No results		324.19325	9.453
No results	No results	No results	No results		460.20923	12.463
No results	No results	No results	No results		436.17286	9.083
No results	No results	No results	No results		823.53462	18.001
No results	No results	No results	No results		279.91464	0.727
No results	No results	No results	No results		617.42842	14.609
No results	No results	No results	No results		251.08696	4.444
No results	No results	No results	No results		806.35505	9.726
No results	No results	No results	No results		356.14686	7.13
No results	No results	No results	No results		217.99995	2.944
No results	No results	No results	No results		624.27888	17.887
No results	No results	No results	No results		383.90907	4.209
No results	No results	No results	No results		640.25361	12.917
No results	No results	No results	No results		798.33403	14.21
No results	No results	No results	Full match		234.19842	9.652
No results	No results	No results	No results		265.09627	6.773
No results	No results	No results	No results		558.24751	16.94
No results	Full match	No results	No results		236.10491	8.391
No results	No results	No results	No results		569.15404	8.135
No results	No results	No results	Full match		216.12143	5.669
No results	No results	No results	No results		610.26659	9.091
No results	No results	No results	No results		688.46809	18.085
No results	No results	No results	Full match		308.27166	8.248
No results	No results	No results	No results		685.26704	13.45
No results	No results	No results	No results		227.13715	18.206
No results	No results	No results	No results		434.07213	3.273
No results	No results	No results	No results		552.07913	5.534
No results	No results	No results	No results		467.82268	0.07
No results	No results	No results	No results		219.92184	2.933
No results	No results	No results	No results		294.07139	8.184
No results	No results	No results	No results		1560.1564	14.358
No results	No results	No results	No results		164.90352	18.231
No results	No results	No results	No results		846.54078	17.995
No results	No results	No results	No results		777.49257	18.122

No results	No results	No results	No results		489.09447	7.032
No results	No results	No results	No results		308.1985	11.131
No results	No results	No results	No results		605.12532	8.942
No results	No results	No results	No results		760.58025	13.249
No results	No results	No results	No results		1032.5327	16.128
No results	No results	No results	No results		285.12117	2.644
No results	No results	No results	No results		568.08882	8.4
No results	No results	No results	No results		215.14374	5.575
No results	No results	No results	No results		228.02649	5.775
No results	No results	No results	No results		884.49151	18.025
No results	No results	No results	No results		243.92383	18.4
No results	No results	No results	No results		570.30726	11.795
No results	No results	No results	No results		309.87065	3.656
No results	No results	No results	Full match		392.12568	9.468
No results	No results	No results	No results		135.08158	0.087
No results	No results	No results	No results		397.89747	13.721
No results	No results	No results	Full match		328.05803	8.176
No results	No results	No results	Full match		466.12584	7.96
No results	No results	No results	No results		215.15219	3.496
No results	No results	No results	No results		448.22218	11.003
No results	No results	No results	No results		570.21016	7.362
No results	No results	No results	No results		853.51821	17.122
No results	No results	No results	No results		288.0414	3.226
No results	No results	No results	No results		962.61317	15.797
No results	No results	No results	No results		1369.6371	9.925
No results	No results	No results	No results		481.33364	5.736
No results	No results	No results	No results		339.1061	2.068
No results	No results	No results	No results		774.34254	12.387
No results	No results	No results	No results		330.18071	10.524
No results	No results	No results	No results		1368.597	9.749
No results	No results	No results	No results		342.0175	1.603
No results	No results	No results	Full match		226.12036	8.57
No results	No results	No results	No results		235.06967	0.994
No results	No results	No results	No results		634.08064	2.779
No results	No results	No results	No results		556.2848	14.006
No results	No results	No results	No results		506.37578	12.522
No results	No results	No results	No results		821.54414	17.989
No results	No results	No results	No results		500.27461	14.431
No results	No results	No results	No results		314.08088	7.696
No results	No results	No results	No results		548.08375	2.995
No results	No results	No results	No results		691.37817	9.866
No results	Partial matcl	No results	Full match		129.04245	1.417
No results	No results	No results	Full match		284.19895	10.674
No results	No results	No results	No results		639.33838	12.908
No results	No results	No results	No results		1380.6342	10.806
No results	No results	No results	No results		1174.5551	12.053
No results	No results	No results	Full match		436.11549	9.123
No results	No results	No results	No results		793.26409	4.208
No results	No results	No results	No results		554.27565	15.398
No results	No results	No results	No results		821.52971	13.043

No results	No results	No results	No results		1359.6101	10.385
No results	No results	No results	No results		660.20736	9.022
No results	No results	No results	No results		1336.6454	10.942
No results	No results	No results	No results		1191.5825	11.775
No results	No results	No results	No results		403.12666	7.625
No results	No results	No results	Full match		444.1414	7.038
No results	No results	No results	No results		518.27289	17.487
No results	No results	No results	No results		257.99229	10.593
No results	No results	No results	No results		318.07581	7.657
No results	No results	No results	No results		332.01127	4.222
No results	No results	No results	No results		223.90564	18.207
No results	No results	No results	No results		710.45308	18.076
No results	No results	No results	No results		228.15128	10.3
No results	No results	No results	No results		354.95738	4.755
No results	No results	No results	No results		1386.6235	9.974
No results	No results	No results	Full match		450.11656	7.13
No results	No results	No results	Full match		610.13323	3.955
No results	No results	No results	No results		308.19838	12.969
No results	No results	No results	No results		512.11644	3.47
No results	No results	No results	No results		640.28334	18.115
No results	No results	No results	Full match		318.07367	8.013
No results	No results	No results	No results		256.15236	10.574
No results	No results	No results	No results		319.07728	7.175
No results	No results	No results	No results		454.34493	8.298
No results	No results	No results	No results		612.11275	6.968
No results	No results	No results	No results		279.8947	4.275
No results	No results	No results	No results		146.93098	18.207
No results	No results	No results	Full match		532.11928	7.46
No results	No results	No results	No results		246.12111	4.657
No results	No results	No results	No results		551.3228	9.332
No results	No results	No results	No results		700.43465	12.103
No results	No results	No results	No results		994.51311	11.533
No results	No results	No results	No results		147.92957	18.187
No results	No results	No results	No results		547.11587	8.96
No results	No results	No results	No results		1368.6343	8.753
No results	No results	No results	No results		872.43599	18.172
No results	No results	No results	No results		760.55275	17.398
No results	No results	No results	No results		500.27495	14.169
No results	No results	No results	No results		612.41528	16.853
No results	No results	No results	No results		604.12242	8.922
No results	No results	No results	No results		488.29753	14.001
No results	No results	No results	No results		618.39324	11.182
No results	No results	No results	No results		1148.561	7.656
No results	No results	No results	No results		500.27452	14.594
No results	No results	No results	No results		422.23341	17.845
No results	No results	No results	No results		1084.5448	11.029
No results	No results	No results	No results		1190.5717	7.709
No results	No results	No results	Full match		514.18317	7.907
No results	No results	No results	No results		406.32419	11.381
No results	No results	No results	Full match		372.084	11.135

No results	No results	No results	No results		494.24004	5.499
No results	No results	No results	No results		452.21739	7.52
No results	No results	No results	No results		312.88249	18.387
No results	No results	No results	No results		404.30773	9.261
No results	No results	No results	No results		271.28749	11.986
No results	No results	No results	No results		293.05345	5.482
No results	No results	No results	No results		248.65906	18.222
No results	No results	No results	No results		429.14339	7.617
No results	No results	No results	No results		718.11434	8.46
No results	No results	No results	No results		147.06691	7.469
No results	No results	No results	No results		207.16235	9.107
No results	No results	No results	No results		247.64896	18.208
No results	No results	No results	No results		617.14924	4.375
No results	No results	No results	No results		1196.562	10.072
No results	No results	No results	No results		566.30635	7.986
No results	No results	No results	Full match		434.3185	11.228
No results	No results	No results	No results		127.94991	0.901
No results	Partial matcl	No results	Full match		438.35017	9.476
No results	No results	No results	Full match		266.18814	10.666
No results	No results	No results	No results		207.9476	1.361
No results	No results	No results	No results		618.39202	11.913
No results	No results	No results	No results		453.78328	0.093
No results	No results	No results	No results		315.86088	18.165
No results	No results	No results	No results		1188.5939	9.874
No results	No results	No results	No results		1337.6429	12.105
No results	No results	No results	No results		1158.5815	10.752
No results	No results	No results	No results		482.26393	17.345
No results	No results	No results	No results		548.28413	7.389
No results	No results	No results	No results		322.14132	8.777
No results	No results	No results	No results		266.89666	0.747
No results	No results	No results	Full match		456.17812	8.815
No results	No results	No results	No results		475.2701	10.644
No results	No results	No results	No results		574.29866	10.879
No results	No results	No results	No results		464.0746	7.275
No results	No results	No results	No results		168.15136	12.46
No results	No results	No results	No results		211.96129	4.204
No results	No results	No results	No results		1351.5286	9.552
No results	No results	No results	No results		191.10396	5.471
No results	No results	No results	No results		291.11019	7.187
No results	No results	No results	No results		315.27739	11.949
No results	No results	No results	Full match		444.14399	7.029
No results	No results	No results	No results		708.24136	7.932
No results	No results	No results	No results		1038.5019	11.035
No results	No results	No results	Full match		578.14277	8.302
No results	No results	No results	No results		301.00173	1.246
No results	No results	No results	No results		256.82902	18.384
No results	No results	No results	No results		610.39992	14.6
No results	No results	No results	No results		505.32484	10.465
No results	No results	No results	No results		1096.5451	12.014
No results	No results	No results	No results		224.06687	6.065

No results	No results	No results	No results		494.32512	8.412
No results	No results	No results	Full match		300.26621	16.953
No results	No results	No results	No results		116.93473	18.181
No results	Partial matcl	No results	Full match		310.2144	11.393
No results	No results	No results	No results		228.1516	8.672
No results	No results	No results	No results		1369.6374	10.49
No results	No results	No results	Full match		270.1834	7.2
No results	No results	No results	No results		1032.6788	8.467
No results	No results	No results	No results		1176.5561	9.14
No results	No results	No results	Full match		185.11556	1.553
No results	No results	No results	No results		782.01037	18.237
No results	No results	No results	Full match		318.1465	11.31
No results	No results	No results	No results		386.10316	11.678
No results	No results	No results	No results		555.35319	14.665
No results	No results	No results	No results		858.25752	8.318
No results	No results	No results	No results		1344.5486	11.74
No results	No results	No results	No results		598.09999	9.455
No results	No results	No results	No results		368.18774	9.275
No results	No results	No results	No results		750.5244	17.217
No results	No results	No results	No results		361.29033	12.704
No results	No results	No results	No results		608.42579	18.136
No results	No results	No results	Full match		522.10076	6.82
No results	No results	No results	No results		779.99459	18.251
No results	No results	No results	No results		226.15615	10.652
No results	No results	No results	No results		610.39956	15.966
No results	Full match	No results	No results		378.16769	7.964
No results	No results	No results	No results		248.13686	5.041
No results	No results	No results	No results		566.28767	12.982
No results	No results	No results	No results		619.19029	4.389
No results	No results	No results	No results		972.48655	5.647
No results	No results	No results	No results		588.12666	9.627
No results	No results	No results	Full match		294.18278	13.34
No results	No results	No results	No results		474.17631	7.28
No results	No results	No results	No results		161.90387	18.261
No results	No results	No results	No results		672.37196	7.846
No results	Partial matcl	No results	Full match		280.23982	13.44
No results	No results	No results	No results		546.22005	15.288
No results	No results	No results	No results		595.1407	8.694
No results	No results	No results	No results		481.23341	16.964
No results	No results	No results	No results		994.51332	11.244
No results	No results	No results	No results		486.28895	15.738
No results	No results	No results	No results		513.16328	7.663
No results	No results	No results	No results		839.53891	17.805
No results	No results	No results	No results		625.37035	14.399
No results	No results	No results	No results		1188.5712	12.237
No results	No results	No results	No results		299.68639	5.803
No results	No results	No results	No results		1323.6325	10.016
No results	No results	No results	No results		206.11211	18.18
No results	No results	No results	No results		340.88347	18.18
No results	No results	No results	No results		150.95653	0.755

No results	Partial matcl	No results	Full match		300.06297	9.055
No results	No results	No results	No results		560.13154	8.598
No results	No results	No results	No results		340.2004	10.505
No results	No results	No results	No results		544.2881	10.71
No results	No results	No results	No results		211.78345	0.219
No results	No results	No results	No results		826.50007	17.095
No results	No results	No results	No results		1096.5456	11.749
No results	Partial matcl	No results	Full match		316.05815	8.147
No results	No results	No results	No results		362.20684	13.595
No results	No results	No results	No results		1148.5393	11.237
No results	No results	No results	No results		568.0888	8.687
No results	No results	No results	No results		233.05414	2.811
No results	No results	No results	No results		250.89624	2.557
No results	No results	No results	Full match		296.06758	8.162
No results	No results	No results	No results		305.17757	1.88
No results	No results	No results	No results		805.57857	18.205
No results	No results	No results	No results		382.27153	14.501
No results	No results	No results	No results		314.08067	7.419
No results	No results	No results	No results		1338.624	8.488
No results	No results	No results	No results		461.09575	4.93
No results	No results	No results	No results		700.43456	11.998
No results	No results	No results	No results		1170.5448	10.795
No results	No results	No results	No results		200.1382	5.751
No results	No results	No results	No results		837.53262	17.092
No results	No results	No results	No results		496.26193	16.331
No results	No results	No results	No results		322.14127	8.995
No results	No results	No results	No results		508.27971	16.691
No results	No results	No results	No results		506.37661	12.151
No results	No results	No results	No results		647.16298	7.22
No results	No results	No results	Full match		420.12068	8.344
No results	No results	No results	No results		498.18699	8.075
No results	No results	No results	Full match		297.26665	11.259
No results	No results	No results	No results		284.88637	3.674
No results	No results	No results	No results		203.04429	1.641
No results	No results	No results	No results		424.23286	8.134
No results	No results	No results	No results		1191.6052	10.196
No results	No results	No results	No results		398.22991	7.555
No results	No results	No results	No results		477.09938	5.748
No results	No results	No results	No results		187.93507	18.183
No results	No results	No results	No results		544.13661	9.132
No results	No results	No results	Full match		287.15114	5.696
No results	No results	No results	No results		354.22246	17.942
No results	No results	No results	No results		1060.5451	8.848
No results	No results	No results	No results		387.13167	7.626
No results	No results	No results	No results		400.31304	11.742
No results	No results	No results	No results		586.16878	4.428
No results	No results	No results	No results		545.27266	14.312
No results	No results	No results	No results		162.07936	6.115
No results	No results	No results	Full match		295.10884	11.523
No results	No results	No results	Full match		147.06832	7.476

No results	No results	No results	No results		255.64769	11.138
No results	No results	No results	No results		588.12678	9.287
No results	No results	No results	No results		419.08399	13.565
No results	No results	No results	No results		493.28004	10.641
No results	No results	No results	No results		451.26958	12.75
No results	No results	No results	No results		432.23567	9.54
No results	No results	No results	No results		166.91221	17.926
No results	No results	No results	Full match		522.25929	10.744
No results	No results	No results	No results		1189.6047	12.887
No results	No results	No results	No results		293.20724	8.498
No results	No results	No results	No results		734.29317	11.087
No results	No results	No results	No results		626.1598	4.522
No results	No results	No results	No results		481.31639	15.167
No results	No results	No results	No results		431.2737	9.936
No results	No results	No results	No results		493.28023	10.836
No results	No results	No results	No results		608.20582	17.364
No results	No results	No results	No results		900.49885	14.41
No results	No results	No results	No results		1116.536	8.5
No results	No results	No results	No results		284.86271	13.562
No results	No results	No results	No results		406.32366	11.142
No results	No results	No results	No results		1352.6394	10.019
No results	No results	No results	No results		641.26851	17.874
No results	No results	No results	No results		462.24635	8.247
No results	No results	No results	No results		570.2802	13.838
No results	No results	No results	No results		521.27485	13.609
No results	No results	No results	No results		223.83644	18.413
No results	No results	No results	No results		450.23765	11.748
No results	No results	No results	No results		826.50062	16.983
No results	No results	No results	No results		1028.5894	15.908
No results	No results	No results	No results		206.10571	5.945
No results	No results	No results	No results		468.14295	7.842
No results	No results	No results	No results		751.51735	17.396
No results	No results	No results	No results		249.14351	9.635
No results	No results	No results	No results		796.44081	18.08
No results	No results	No results	No results		256.15199	0.229
No results	No results	No results	No results		574.27821	16.665
No results	No results	No results	No results		117.92682	16.97
No results	No results	No results	No results		356.68312	5.649
No results	No results	No results	No results		679.28081	12.1
No results	No results	No results	No results		532.15181	15.511
No results	Partial matcl	No results	Full match		372.1206	7.878
No results	No results	No results	No results		217.7998	5.727
No results	No results	No results	No results		430.25624	10.903
No results	No results	No results	No results		664.28364	14.732
No results	No results	No results	Full match		526.14686	8.293
No results	No results	No results	No results		276.85308	0.076
No results	No results	No results	No results		660.20747	8.588
No results	No results	No results	No results		117.92676	16.303
No results	No results	No results	No results		584.29841	11.466
No results	No results	No results	No results		186.14715	1.504

No results	No results	No results	No results		588.12691	9.81
No results	No results	No results	No results		263.16011	10.864
No results	No results	No results	No results		562.14747	7.641
No results	No results	No results	No results		425.14717	8.694
No results	No results	No results	No results		546.21986	15.502
No results	No results	No results	No results		1014.5037	8.669
No results	No results	No results	Full match		132.04366	2.551
No results	No results	No results	Full match		196.1455	9.756
No results	No results	No results	No results		437.33754	11.737
No results	No results	No results	No results		258.14943	12.839
No results	No results	No results	No results		426.26164	14.686
No results	No results	No results	No results		382.27156	12.783
No results	No results	No results	No results		818.5113	15.31
No results	No results	No results	No results		529.29317	12.878
No results	No results	No results	No results		582.28245	11.241
No results	No results	No results	No results		1000.5037	11.925
No results	No results	No results	Full match		266.15245	9.241
No results	No results	No results	No results		1370.6311	9.592
No results	No results	No results	No results		986.61915	16.508
No results	No results	No results	No results		544.28791	10.827
No results	No results	No results	No results		223.64177	5.841
No results	No results	No results	Full match		400.11775	8.206
No results	No results	No results	No results		322.14126	9.817
No results	No results	No results	No results		249.14275	10.722
No results	No results	No results	No results		612.29091	10.945
No results	No results	No results	Full match		512.16765	7.668
No results	No results	No results	No results		488.36455	12.517
No results	No results	No results	Full match		295.10884	11.294
No results	No results	No results	No results		228.91167	18.415
No results	No results	No results	No results		546.11556	8.429
No results	No results	No results	No results		854.48343	5.424
No results	No results	No results	No results		275.83452	19.298
No results	No results	No results	No results		1351.5294	9.796
No results	No results	No results	No results		1370.6293	10.961
No results	No results	No results	No results		424.23045	8.141
No results	No results	No results	No results		999.57723	15.286
No results	No results	No results	No results		450.23765	11.995
No results	No results	No results	No results		1175.56	12.067
No results	No results	No results	No results		1386.6245	8.909
No results	No results	No results	No results		166.03781	1.595
No results	No results	No results	No results		245.14179	12.509
No results	No results	No results	No results		567.14613	7.645
No results	No results	No results	No results		584.29835	11.905
No results	No results	No results	No results		857.45099	16.299
No results	No results	No results	No results		115.99525	18.155
No results	No results	No results	No results		123.03622	10.97
No results	No results	No results	No results		998.58204	16.115
No results	No results	No results	No results		143.94429	18.178
No results	No results	No results	No results		611.13643	7.68
No results	No results	No results	No results		591.32313	10.292

No results	No results	No results	No results		579.31715	11.064
No results	No results	No results	No results		1364.64	9.562
No results	No results	No results	No results		368.24717	5.657
No results	No results	No results	No results		1190.5498	11.173
No results	No results	No results	No results		873.48311	14.662
No results	Partial matcl	No results	Full match		654.38008	17.924
No results	No results	No results	No results		597.32734	9.333
No results	No results	No results	No results		999.57588	15.512
No results	No results	No results	Full match		202.08548	3.467
No results	No results	No results	No results		352.16505	8.662
No results	Partial matcl	No results	Full match		250.15648	15.082
No results	No results	No results	No results		1028.5181	9.741
No results	No results	No results	No results		184.18447	8.678
No results	No results	No results	No results		1042.5118	11.994
No results	No results	No results	No results		226.08372	8.452
No results	No results	No results	No results		597.32734	9.516
No results	No results	No results	No results		981.59364	15.506
No results	No results	No results	No results		822.43324	16.288
No results	No results	No results	Full match		504.08807	8.007
No results	No results	No results	No results		751.51985	17.196
No results	No results	No results	No results		189.07741	7.533
No results	No results	No results	No results		1452.585	10.389
No results	No results	No results	No results		223.38921	18.865
No results	No results	No results	No results		977.59199	15.931
No results	No results	No results	No results		199.11919	5.386
No results	No results	No results	No results		1096.5438	10.433
No results	No results	No results	No results		408.25441	15.621
No results	No results	No results	No results		1188.5916	10.588
No results	No results	No results	No results		1054.5341	11.176
No results	No results	No results	No results		787.58076	17.877
No results	No results	No results	No results		1130.5507	9.702
No results	No results	No results	No results		952.50178	10.713
No results	No results	No results	No results		652.41865	14.704
No results	No results	No results	Full match		194.09446	10.424
No results	No results	No results	No results		459.11876	7.209
No results	No results	No results	No results		388.03255	9.957
No results	No results	No results	No results		253.15318	9.262
No results	No results	No results	No results		145.92929	0.085
No results	No results	No results	No results		490.30949	16.304
No results	No results	No results	No results		126.90607	16.948
No results	No results	No results	No results		680.21718	16.793
No results	No results	No results	No results		126.90616	16.299
No results	No results	No results	No results		115.99445	18.171
No results	No results	No results	No results		570.27168	16.298

Area (Max.)	# ChemSpid	# mzCloud R	# mzVault R	# Metabolika	Metabolika I
1.27726E+11	0	0	2	1	Other
37436939740	0	0	3	0	
37333747824	0	0	3	0	
19352152338	0	0	5	0	
15892901032	0	0	0	0	
10026621527	0	0	0	0	
8156811560	0	0	0	1	Other
7706333278	0	0	0	0	
7472718067	0	0	8	0	
6682366943	0	0	0	0	
6117120759	0	0	2	0	
5815924848	0	0	2	8	Superpathw
5564170359	0	0	5	0	
5545888584	0	0	0	1	Superpathw
4306805958	0	0	0	0	
4215652461	0	0	0	0	
4147213657	0	0	0	0	
4090107839	0	0	0	0	
3999099844	0	0	0	0	
3939911358	0	0	0	0	
3928364583	0	0	6	0	
3808585746	0	0	0	0	
3533771152	0	0	5	1	Other
3412899614	0	0	0	1	Other
3324041541	0	0	2	0	
3302224061	0	0	0	0	
3034275702	0	0	0	0	
3009023843	0	0	0	1	Superpathw
2899011602	0	0	0	0	
2892058980	0	0	1	8	Superpathw
2882526033	0	0	1	0	
2802617722	0	0	0	0	
2788135706	0	0	0	0	
2684459349	0	0	0	0	
2576654602	0	0	0	0	
2574512419	0	0	0	0	
2540677776	0	0	0	0	
2401642371	0	0	0	0	
2392802006	0	0	0	0	
2358221246	0	0	0	1	Superpathw
2310763982	0	0	0	0	
2258923439	0	0	0	0	
2246724596	0	0	0	0	
2245690824	0	0	0	0	
2068029941	0	0	0	0	
2061038535	0	0	2	0	
1936319462	0	0	2	1	Superpathw
1930553652	0	0	0	0	

1890488169	0	0	2	8	Superpathw
1864864170	0	0	1	0	
1850708815	0	0	0	0	
1847140799	0	0	0	0	
1791617044	0	0	0	0	
1784076010	0	0	0	0	
1769286112	0	0	0	3	Other
1767764014	0	0	0	0	
1738853497	0	0	0	0	
1717681215	0	0	0	0	
1539702130	0	0	2	0	
1511205199	0	0	0	0	
1499490883	0	0	0	0	
1495429506	0	0	0	0	
1484946827	0	0	0	0	
1477556421	0	0	0	0	
1473891103	0	0	0	0	
1469746564	0	0	0	0	
1459247515	0	0	0	0	
1457891594	0	0	0	0	
1443053813	0	0	0	0	
1435045925	0	0	0	0	
1423419652	0	0	0	0	
1408450423	0	0	0	0	
1406165574	0	0	0	0	
1393453579	0	0	0	0	
1352424016	0	0	2	0	
1319721356	0	0	0	0	
1299906655	0	0	8	0	
1278400076	0	0	4	1	Other
1252648929	0	0	0	0	
1223406314	0	0	0	0	
1205366082	0	0	0	0	
1200918174	0	0	2	0	
1198598213	0	0	0	0	
1178220254	0	0	0	0	
1168178099	0	0	6	1	Superpathw
1150529061	0	0	0	0	
1139270949	0	0	0	0	
1089760625	0	0	0	0	
1079584077	0	0	0	0	
1075333000	0	0	0	0	
1055506774	0	0	0	14	Superpathw
1047087863	0	0	0	0	
1031417141	0	0	0	0	
1028230743	0	0	0	0	
1023643437	0	0	0	0	
1019640367	0	0	0	0	
1019035507	0	0	0	0	
1016364782	0	0	1	14	Superpathw

1013634479	0	0	0	0	
1013500939	0	0	1	0	
997977504.1	0	0	0	0	
965343911.6	0	0	0	0	
955573302.8	0	0	0	1	Other
946540737.3	0	0	0	0	
934309353.3	0	0	0	0	
897426821.6	0	0	10	0	
896074337.9	0	0	0	0	
894910670.6	0	0	0	0	
886628448.6	0	0	0	0	
878896073.4	0	0	0	0	
875223417	0	0	0	0	
865219704.6	0	0	1	5	Superpathw
862790291.6	0	0	1	1	Superpathw
857879286	0	0	0	0	
849617571.8	0	0	0	3	Other
845616618.4	0	0	0	0	
835428371.8	0	0	0	0	
833294007.7	0	0	0	0	
819523770.9	0	0	0	0	
815971792.7	0	0	0	0	
810615736.4	0	0	0	0	
805404324.9	0	0	0	0	
788658133.3	0	0	1	1	Superpathw
788574258.1	0	0	0	0	
784768443.2	0	0	0	0	
782127989.1	0	0	0	0	
773462500.7	0	0	1	1	Superpathw
769027732.4	0	0	0	3	Other
761042330	0	0	0	0	
759838052	0	0	0	0	
755085522.7	0	0	0	0	
747389053	0	0	3	0	
745237566.9	0	0	0	0	
741175652.2	0	0	0	0	
739106987.4	0	0	0	0	
736586069.1	0	0	0	0	
734725681	0	0	0	0	
734110657.1	0	0	0	0	
726794997.8	0	0	0	0	
720595761.6	0	0	0	0	
717825703.8	0	0	0	0	
716198311.3	0	0	0	0	
712100491	0	0	0	0	
700368513.2	0	0	0	0	
689507179.1	0	0	1	0	
687982663.2	0	0	0	0	
682666529.8	0	0	0	0	
680883792.6	0	0	0	0	

680328554.2	0	0	0	0	
676266285.8	0	0	0	0	
675175704	0	0	0	0	
674916438.5	0	0	0	0	
666915455	0	0	0	0	
666181209.4	0	0	0	0	
661561126	0	0	0	0	
657180101.8	0	0	3	5	Superpathw
656284961.5	0	0	0	0	
650761184.2	0	0	0	0	
648525353	0	0	3	13	Superpathw
643995927.4	0	0	1	0	
635507739.1	0	0	0	0	
635205839	0	0	0	1	Superpathw
633395899.4	0	0	0	0	
630173316.1	0	0	0	0	
629101759.4	0	0	0	0	
622721768.4	0	0	0	0	
621540767	0	0	2	1	Other
612371517.9	0	0	0	0	
605712619.9	0	0	0	0	
602331583.4	0	0	0	0	
600570083.4	0	0	0	0	
598101538.8	0	0	0	0	
595583783.3	0	0	0	0	
594369868.7	0	0	0	0	
589800625.7	0	0	0	0	
589147003.9	0	0	0	0	
589055367.3	0	0	0	1	Other
585863680.3	0	0	0	0	
584936629.2	0	0	3	0	
581847865.1	0	0	0	0	
575694285.8	0	0	0	0	
574245045.7	0	0	0	0	
566532230.5	0	0	0	0	
563047218.9	0	0	0	0	
558492181.5	0	0	0	0	
551828269.6	0	0	0	5	Other
545020599.3	0	0	0	0	
543766720.3	0	0	0	0	
543546287.8	0	0	0	0	
536871896.6	0	0	0	0	
536453652.2	0	0	0	0	
532639343	0	0	0	0	
531186049.8	0	0	2	0	
523880986.6	0	0	0	0	
522913092.8	0	0	0	0	
516273097.5	0	0	0	0	
516138955.6	0	0	0	0	
514914490.9	0	0	3	1	Superpathw

513574394.5	0	0	0	0	
509617820.5	0	0	0	0	
493368904.8	0	0	0	0	
490658334.3	0	0	0	0	
486671910.2	0	0	0	0	
482186399.5	0	0	0	0	
480581707.8	0	0	0	0	
478654193.5	0	0	0	0	
471643539	0	0	0	0	
470696408	0	0	0	0	
463316764.4	0	0	0	0	
459549268.8	0	0	0	1	Superpathw
458648118.6	0	0	4	1	Superpathw
458594491.9	0	0	0	0	
457674595.7	0	0	0	0	
457574048.1	0	0	1	1	Superpathw
454735710.7	0	0	0	0	
454633012.8	0	0	0	0	
452346988.1	0	0	0	0	
451927513.3	0	0	0	0	
451338358.2	0	0	0	0	
447944583.8	0	0	0	0	
446633288.7	0	0	0	0	
445628541.2	0	0	0	0	
444227491.1	0	0	0	0	
444086994.2	0	0	0	0	
443864722.1	0	0	0	0	
442728831.1	0	0	0	4	Other
439493592.1	0	0	0	0	
438954914.4	0	0	0	1	Other
436743874.3	0	0	0	0	
433540182.3	0	0	0	0	
431663320.5	0	0	0	0	
429734800.8	0	0	0	0	
429010976.9	0	0	0	0	
428419376.7	0	0	0	0	
426390218.7	0	0	0	2	Superpathw
423433503.3	0	0	1	2	Other
418619367	0	0	0	0	
408783752.2	0	0	0	1	Other
406714242.6	0	0	0	0	
406513220.5	0	0	0	0	
406374875.8	0	0	4	1	Superpathw
406296498	0	0	0	0	
406085531	0	0	0	0	
405459627.2	0	0	0	0	
405338932.9	0	0	0	0	
404684241.9	0	0	0	0	
404297771.5	0	0	0	0	
400760471.9	0	0	0	0	

397335231.3	0	0	0	0	
396787291.6	0	0	0	0	
392901911.9	0	0	0	0	
387429930.3	0	0	0	0	
385626025.7	0	0	0	0	
384298962.7	0	0	0	0	
383558029.7	0	0	0	0	
382461368.4	0	0	0	0	
378283445.6	0	0	0	0	
376071341.6	0	0	0	0	
372463792	0	0	0	0	
372070949.5	0	0	0	0	
371533662.5	0	0	0	2	Other
370921115.9	0	0	0	0	
370569476.9	0	0	0	0	
370071276.9	0	0	0	0	
367639900.4	0	0	0	0	
363393761.2	0	0	0	0	
363193962.8	0	0	0	0	
362081773.8	0	0	0	0	
360561874.2	0	0	0	0	
359998383.9	0	0	0	0	
358915507.4	0	0	1	2	Other
358246598.3	0	0	0	0	
357573086.7	0	0	0	0	
355190319.8	0	0	0	0	
355023876.3	0	0	0	0	
352352154.8	0	0	0	0	
351994974.1	0	0	0	0	
351780822.3	0	0	0	2	Aspartate su
350898194.2	0	0	0	0	
349832422	0	0	0	0	
347402670.5	0	0	0	0	
343829861	0	0	0	0	
340248618.6	0	0	0	0	
339793011	0	0	0	0	
339384326.9	0	0	0	0	
339054978.7	0	0	0	0	
338229817.2	0	0	0	0	
337188382.7	0	0	0	0	
336914797.5	0	0	0	1	Other
335047901.3	0	0	0	0	
333635517.8	0	0	0	0	
333247098	0	0	0	0	
332079677.9	0	0	0	0	
331592696.9	0	0	0	0	
331297523.1	0	0	0	0	
330392696.3	0	0	0	0	
330054614.7	0	0	0	0	
328300304.6	0	0	0	0	

328124213.1	0	0	0	0	
325522487.3	0	0	0	0	
323425105.8	0	0	0	0	
320923467.9	0	0	0	0	
317811134.4	0	0	0	0	
314616795.2	0	0	0	0	
312172321.4	0	0	0	0	
310759922.3	0	0	0	0	
310749864.9	0	0	2	0	
309115143	0	0	0	0	
307079834.3	0	0	0	1	Superpathwa
304949927.8	0	0	0	0	
304187299.9	0	0	0	0	
302626740.1	0	0	0	0	
302334864.1	0	0	0	0	
299927385.9	0	0	0	0	
294872715.4	0	0	0	0	
292622343.9	0	0	0	0	
292345850.8	0	0	0	0	
291510760.3	0	0	0	0	
290055265.8	0	0	0	0	
289748068.4	0	0	0	0	
289299753.2	0	0	2	1	Superpathwa
289019373.6	0	0	0	0	
288672857.6	0	0	0	0	
288532652.5	0	0	0	0	
288421892.1	0	0	0	0	
288325686.6	0	0	0	0	
284964755	0	0	0	0	
284689712.1	0	0	0	0	
283809732.5	0	0	0	2	Other
283527350.5	0	0	0	0	
282360176.5	0	0	0	0	
281973943.3	0	0	0	2	Other
281026217.4	0	0	0	0	
280873546.3	0	0	0	0	
280650169.2	0	0	0	0	
280600341.3	0	0	0	0	
280010081.3	0	0	2	0	
278931589.8	0	0	0	0	
276873087.7	0	0	0	1	Other
275941993	0	0	0	0	
275841179.3	0	0	0	0	
274781238.5	0	0	0	0	
274262508.1	0	0	0	0	
273814031.5	0	0	0	0	
273120298.8	0	0	0	2	Superpathwa
271852049.6	0	0	0	0	
271302494.3	0	0	0	0	
270924330.2	0	0	0	0	

269618771.9	0	0	0	0	
267781711.6	0	0	0	0	
267140281.1	0	0	0	0	
266932738	0	0	0	0	
266383941.9	0	0	0	0	
266287140.1	0	0	0	0	
266011590.9	0	0	0	0	
265501807.4	0	0	0	0	
265193823.4	0	0	0	0	
264800071.2	0	0	0	3	Superpathw
263427588.9	0	0	0	0	
262851817	0	0	0	1	Other
262097356.6	0	0	0	0	
262079374.1	0	0	0	1	Other
261820479.2	0	0	0	1	Superpathw
261425869.2	0	0	0	0	
258118249	0	0	0	0	
255799139.8	0	0	0	93	Superpathw
255663522.1	0	0	0	1	Superpathw
254982742.1	0	0	1	0	
254488500.1	0	0	0	1	Superpathw
253974904.6	0	0	0	4	Other
253935674.4	0	0	0	0	
252274961	0	0	2	0	
251737050.5	0	0	0	0	
251284178.3	0	0	0	0	
250893508.8	0	0	0	0	
250826020.4	0	0	0	0	
250229248.7	0	0	4	0	
250209866.9	0	0	0	0	
250190603.4	0	0	0	0	
247937578.6	0	0	0	0	
244937483.6	0	0	0	0	
244513695.4	0	0	0	0	
242768808.9	0	0	0	1	Superpathw
242707606.2	0	0	2	0	
241910560.3	0	0	1	0	
241028340.9	0	0	0	0	
240094594.1	0	0	0	0	
239398355.1	0	0	0	4	Other
239331055.9	0	0	0	0	
238962092.8	0	0	0	0	
238513441.4	0	0	0	0	
238352401.2	0	0	0	0	
237258132	0	0	2	6	Superpathw
236807953.1	0	0	0	0	
236480640.2	0	0	0	0	
234891731.7	0	0	1	1	Superpathw
234536101.3	0	0	0	0	
233777677.8	0	0	2	2	Other

233767506.1	0	0	1	0	
233077348.1	0	0	0	0	
232887450	0	0	0	0	
232486827.9	0	0	0	0	
231193373.3	0	0	0	0	
230688533.3	0	0	0	0	
229247230.5	0	0	0	0	
227315197.2	0	0	0	0	
223129912.6	0	0	0	0	
222791897.4	0	0	0	0	
222137536.9	0	0	0	0	
222111996.1	0	0	0	0	
220956828.6	0	0	0	0	
220794286	0	0	0	0	
220175114.6	0	0	0	0	
219704455.9	0	0	0	1	Aspartate su
219277407.2	0	0	0	0	
218551200.3	0	0	0	0	
218380530.4	0	0	0	0	
217012864.7	0	0	0	0	
216748123.4	0	0	0	0	
216179342.1	0	0	0	0	
214591085.1	0	0	0	0	
214031146.9	0	0	0	0	
214031146.9	0	0	0	0	
212140469.3	0	0	0	0	
211265565.2	0	0	0	0	
210370633.8	0	0	0	0	
210206367.8	0	0	0	0	
209562540.4	0	0	0	0	
208869500.2	0	0	0	0	
207906113.5	0	0	0	0	
207795730.1	0	0	0	0	
207300749.7	0	0	0	4	Aspartate su
206698468	0	0	0	0	
205741446	0	0	0	0	
204290903.4	0	0	0	0	
204100517.9	0	0	0	0	
203848059.2	0	0	0	0	
203706573.3	0	0	0	0	
203329380.6	0	0	0	0	
203123700.5	0	0	0	1	Other
202792653.8	0	0	0	0	
202353306.7	0	0	0	0	
200681761.5	0	0	0	0	
200093897.8	0	0	1	0	
199408393.3	0	0	0	0	
197057605.5	0	0	0	0	
196747276.2	0	0	0	0	
196228196.2	0	0	1	0	

194893206.7	0	0	3	1	Superpathw
193857266.6	0	0	0	1	Superpathw
193739635.5	0	0	0	0	
193568106.5	0	0	0	0	
193544167.6	0	0	0	0	
193331826.1	0	0	0	0	
192956459.2	0	0	0	0	
192367649.4	0	0	0	0	
192218924.3	0	0	0	0	
192114572.1	0	0	0	0	
191821426.6	0	0	0	0	
191349958.6	0	0	0	0	
191233462.5	0	0	0	0	
191047774.3	0	0	0	0	
189745368.3	0	0	3	1	Superpathw
188982102.3	0	0	0	0	
188071381.9	0	0	0	0	
187637376	0	0	0	0	
186736317.7	0	0	0	0	
186329806.7	0	0	0	0	
186292910.2	0	0	0	0	
185881552.3	0	0	0	0	
185778071	0	0	0	0	
185772601.4	0	0	0	93	Superpathw
185257429.4	0	0	0	1	Other
184736207.8	0	0	0	0	
184325866.6	0	0	0	0	
184169860.1	0	0	0	9	Other
183508177.5	0	0	0	0	
182997313.6	0	0	0	0	
182762136.5	0	0	0	0	
182518382.1	0	0	0	0	
182197953	0	0	0	0	
181926377.1	0	0	0	0	
181594046.8	0	0	0	0	
181518590.2	0	0	0	0	
181001010.4	0	0	0	0	
179766792.6	0	0	0	0	
179729139.6	0	0	0	1	Superpathw
179702669.7	0	0	0	0	
179147942.8	0	0	0	0	
178884825.3	0	0	0	0	
178787616.4	0	0	0	0	
178272428.5	0	0	0	0	
178150049.3	0	0	0	0	
177705685.4	0	0	0	0	
177534283.9	0	0	0	0	
177292386.1	0	0	0	0	
177286592.2	0	0	0	0	
176141256.3	0	0	0	0	

176026823.1	0	0	0	0	
175845327.9	0	0	0	0	
175206611.8	0	0	0	0	
174335249.2	0	0	0	0	
173570865.5	0	0	0	0	
172912463	0	0	0	0	
172745948.9	0	0	0	0	
171997511.3	0	0	0	0	
171765910.2	0	0	1	1	Other
171223121.3	0	0	0	0	
171012087.2	0	0	0	0	
170700377.1	0	0	0	0	
170478528	0	0	0	0	
170108327.7	0	0	0	0	
169944645.7	0	0	0	0	
169497279.3	0	0	2	0	
169430978.8	0	0	0	0	
168542490	0	0	0	0	
168321358.5	0	0	0	0	
167009091.4	0	0	0	0	
166896739.3	0	0	0	0	
166397726.1	0	0	0	0	
166307159.5	0	0	0	0	
166002830.8	0	0	0	0	
165354119.5	0	0	0	0	
165354119.5	0	0	0	0	
164950388.4	0	0	0	0	
164122735.7	0	0	0	0	
163846022.2	0	0	0	0	
161242104.6	0	0	0	0	
161203523.5	0	0	0	0	
161084198.9	0	0	0	1	Superpathw
160880285.7	0	0	0	0	
160739410.8	0	0	0	0	
160528860.2	0	0	0	0	
160177082.9	0	0	0	0	
159820139.6	0	0	0	0	
159636995.8	0	0	0	5	Other
159066284.2	0	0	0	0	
158409002.4	0	0	0	2	Other
158240291.9	0	0	0	0	
157691834.6	0	0	0	0	
157677226.9	0	0	0	0	
157552359.9	0	0	0	0	
157369062.4	0	0	0	0	
156832109.9	0	0	0	0	
156344908.4	0	0	0	0	
156187747.4	0	0	0	0	
155941269.9	0	0	0	0	
155823648.7	0	0	0	0	

155055993.3	0	0	0	0	
154985454.3	0	0	0	0	
154605327.9	0	0	0	15	Superpathw
154465554.4	0	0	0	0	
154251807.5	0	0	0	1	Superpathw
154223768.3	0	0	0	0	
154052988.3	0	0	0	1	Superpathw
153581405.6	0	0	0	0	
153302623.3	0	0	0	0	
153215657.3	0	0	0	2	Other
152805506	0	0	0	0	
152567008	0	0	0	0	
152306357.4	0	0	0	0	
152037510.9	0	0	0	0	
149385997	0	0	0	0	
149142097.6	0	0	0	0	
149134780.3	0	0	0	0	
148932791.2	0	0	0	0	
148902776.9	0	0	0	0	
148890761.2	0	0	0	0	
148219285.4	0	0	0	0	
148048903	0	0	0	0	
147316267.5	0	0	0	0	
147195303.8	0	0	0	13	Superpathw
146845061	0	0	0	0	
146706134.7	0	0	0	0	
146447129.5	0	0	0	0	
146435010.8	0	0	0	0	
146386544.2	0	0	0	0	
146285939.4	0	0	0	0	
146223383.1	0	0	0	1	Superpathw
146105948	0	0	0	0	
145391114.8	0	0	0	0	
145058055.8	0	0	0	0	
144985962.1	0	0	0	0	
144704178.4	0	0	0	0	
144558069	0	0	0	0	
143268799.7	0	0	0	1	Other
143102962.6	0	0	0	0	
142951587	0	0	0	0	
142459399.1	0	0	0	3	Other
142459399.1	0	0	0	0	
142410141.3	0	0	0	0	
142379324.5	0	0	0	1	Other
141891692.5	0	0	0	0	
140958987.3	0	0	0	0	
140861898.3	0	0	0	1	Superpathw
140790829.7	0	0	0	0	
140457856.4	0	0	0	0	
140175974.3	0	0	0	0	

140136311.3	0	0	0	0	
139687850.5	0	0	0	0	
139547628.9	0	0	0	0	
139457860.1	0	0	0	0	
139449025.6	0	0	0	0	
139338045.4	0	0	0	0	
139256723.3	0	0	0	0	
139158187.6	0	0	0	0	
138951090	0	0	0	0	
138708015.8	0	0	0	13	Superpathw
138493512.7	0	0	0	0	
138433813.6	0	0	0	4	Other
138382088.2	0	0	0	0	
137583951.3	0	0	0	0	
137075526.3	0	0	0	0	
137036954.4	0	0	0	0	
136787901.8	0	0	0	0	
136600902.3	0	0	0	2	Superpathw
136500396.8	0	0	0	0	
136304186.8	0	0	0	0	
136161536.8	0	0	0	0	
136067114.1	0	0	0	0	
136066163.1	0	0	0	0	
135577526	0	0	0	0	
135545136.8	0	0	0	0	
134862040.4	0	0	0	0	
134398352.3	0	0	6	0	
134207600.9	0	0	0	0	
134156458.3	0	0	0	0	
134084927	0	0	0	1	Superpathw
133235638.3	0	0	0	0	
133223347.6	0	0	0	0	
132940524.7	0	0	0	0	
132928715.5	0	0	0	0	
132650456.3	0	0	0	0	
132360950.1	0	0	0	0	
131785452	0	0	0	0	
131587579.1	0	0	0	0	
131362394.3	0	0	0	0	
131277284.2	0	0	0	0	
131053196.1	0	0	0	0	
130912497.8	0	0	0	0	
130670565.5	0	0	0	0	
130020752.6	0	0	0	0	
129435276.1	0	0	0	0	
129334504	0	0	0	0	
129246847.2	0	0	0	0	
129237673.6	0	0	0	0	
128798019.6	0	0	0	0	
128441523.4	0	0	0	0	

128233511.9	0	0	0	0	
128156612.3	0	0	0	0	
128109383.5	0	0	0	0	
128004205.9	0	0	0	0	
127570692.7	0	0	0	0	
127327493.9	0	0	0	0	
127313533.5	0	0	0	0	
127182466.1	0	0	0	0	
126728009.3	0	0	0	0	
126305125.2	0	0	0	0	
126294272.3	0	0	0	0	
126272324.3	0	0	0	0	
126015618.8	0	0	0	1	Other
125882165.9	0	0	0	0	
125671619	0	0	0	0	
125547528.5	0	0	0	0	
125416305.5	0	0	0	0	
125411513	0	0	0	0	
125382766	0	0	0	0	
125185922.3	0	0	0	0	
124064283.6	0	0	0	0	
123850905.2	0	0	0	0	
123694331.6	0	0	0	0	
123495547.1	0	0	0	0	
123477740.4	0	0	0	0	
123336415.6	0	0	0	0	
123315909.5	0	0	0	0	
123288714.2	0	0	0	0	
123223395.8	0	0	0	0	
122786939.3	0	0	0	0	
122428151.4	0	0	0	0	
121654696.6	0	0	0	0	
121622487.2	0	0	0	0	
121560456.6	0	0	0	0	
121520009.2	0	0	0	0	
121078214	0	0	0	0	
121074269.8	0	0	0	1	Superpathw
121056726.8	0	0	0	0	
120991885.7	0	0	0	0	
120938629.3	0	0	0	0	
120595202.3	0	0	0	0	
120522279.6	0	0	0	0	
120112668.8	0	0	0	0	
120104755.8	0	0	0	0	
120070548.9	0	0	0	0	
120068301.9	0	0	0	0	
120067245.1	0	0	0	0	
120035508.1	0	0	0	0	
119986294.4	0	0	0	1	Superpathw
119955410.2	0	0	0	0	

119896410.4	0	0	0	0	
119801370.5	0	0	0	0	
119207761.2	0	0	0	0	
119153686.8	0	0	0	0	
118991205.4	0	0	0	0	
118922584.4	0	0	0	0	
118770099	0	0	0	0	
118472106.2	0	0	0	0	
118241917.1	0	0	0	0	
117976428.2	0	0	0	0	
117444856.7	0	0	0	2	Aspartate su
116988269.3	0	0	0	0	
116845351.6	0	0	0	0	
116577311.1	0	0	0	0	
116495192.2	0	0	0	1	Superpathwa
116048692.2	0	0	0	0	
115853880.4	0	0	0	0	
115440062.6	0	0	0	0	
115175077	0	0	0	0	
114986150	0	0	0	0	
114921679.2	0	0	0	0	
114714335.8	0	0	0	2	Anaerobic ar
114154010.9	0	0	0	0	
114069869.5	0	0	0	0	
114006293.7	0	0	0	0	
113763908.5	0	0	0	0	
113752947.4	0	0	0	0	
113734148.2	0	0	0	0	
113718029.5	0	0	0	1	Superpathwa
113716217.5	0	0	0	0	
113498975.2	0	0	0	0	
113334835.2	0	0	0	0	
113279011.3	0	0	0	0	
112949408.9	0	0	0	0	
112753396.2	0	0	0	0	
112291390.1	0	0	0	0	
112179428.8	0	0	0	0	
112116056.4	0	0	0	0	
111985448.2	0	0	0	0	
111684835.1	0	0	0	0	
111669092.1	0	0	0	1	Other
111605927.7	0	0	0	0	
111590507.8	0	0	0	0	
111414856.2	0	0	0	0	
111221850.4	0	0	0	0	
110896256.3	0	0	0	0	
110576627.1	0	0	0	0	
110531705.1	0	0	0	0	
110224354.1	0	0	0	0	
110031034	0	0	0	0	

109114801.6	0	0	0	0	
109029088.6	0	0	0	0	
108716859.4	0	0	0	0	
108666493.5	0	0	0	0	
108660142.9	0	0	0	0	
108635998.3	0	0	0	0	
108627910.4	0	0	0	0	
108579647.9	0	0	0	0	
108462883.5	0	0	0	0	
108219761.1	0	0	0	0	
108184752.7	0	0	0	0	
107953556	0	0	0	0	
107624968.4	0	0	0	0	
107334536.7	0	0	0	0	
107270179.1	0	0	0	0	
107229706.7	0	0	0	0	
107115632.9	0	0	0	0	
106686315.2	0	0	0	0	
106538868.3	0	0	0	0	
106131638.1	0	0	0	0	
106087478.2	0	0	0	0	
105748078.6	0	0	0	4	Other
105405585.8	0	0	0	0	
105333605.2	0	0	0	0	
105260738.5	0	0	0	0	
105252275.3	0	0	0	0	
105192655.5	0	0	0	0	
105173304	0	0	0	0	
105145019	0	0	0	0	
105096200.4	0	0	0	0	
105037355.9	0	0	0	0	
104964756	0	0	0	0	
104888815	0	0	0	0	
104509052.8	0	0	0	0	
104139129.9	0	0	0	0	
104048993.6	0	0	0	0	
103992088.7	0	0	0	0	
103947245.6	0	0	0	1	Other
103420955.1	0	0	0	0	
103417166.5	0	0	0	0	
103372215.7	0	0	0	0	
103371951.6	0	0	0	0	
103262969.7	0	0	0	0	
103141045.7	0	0	0	2	Superpathw
103021396	0	0	0	0	
102934835.2	0	0	0	0	
102843388.7	0	0	0	0	
102735192.8	0	0	0	0	
102701142.4	0	0	0	0	
102613717.5	0	0	0	4	Other

102149087	0	0	0	0	
102069680.4	0	0	0	95	Superpathwa
101714250	0	0	0	0	
101671547.2	0	0	0	0	
101604598.2	0	0	0	0	
101359701.4	0	0	0	0	
101252729.4	0	0	0	0	
100549987.5	0	0	0	0	
100503276.9	0	0	0	0	
100447091.2	0	0	0	0	
100415724.2	0	0	0	0	
100344469.7	0	0	0	0	
100259473.7	0	0	0	0	
100140101.7	0	0	2	0	
99997045.87	0	0	0	0	
99894431.34	0	0	0	2	Aspartate su
99562384.18	0	0	0	0	
99520607.13	0	0	0	5	Superpathwa
99241230.29	0	0	0	0	
98958317.28	0	0	0	0	
98925496.82	0	0	0	0	
98921858.11	0	0	0	1	Superpathwa
98614404.8	0	0	0	0	
98500446.81	0	0	0	0	
98247950.84	0	0	0	0	
98234097.23	0	0	0	1	Superpathwa
98217544.78	0	0	0	0	
98119753.96	0	0	0	0	
98116301.38	0	0	0	0	
98058373.21	0	0	0	0	
97513044.75	0	0	0	0	
97274389.57	0	0	0	0	
96900824.77	0	0	0	0	
96693752.91	0	0	0	0	
96653220.23	0	0	0	0	
96456824.98	0	0	0	0	
96438443.56	0	0	0	0	
96349188.45	0	0	0	1	Superpathwa
96186759.31	0	0	0	0	
95810871.19	0	0	0	0	
95723123.81	0	0	0	0	
95493701.95	0	0	0	0	
95309655.46	0	0	0	0	
95115500.35	0	0	0	0	
94792357.78	0	0	0	0	
94777932.83	0	0	0	0	
94483013.21	0	0	0	0	
94234841.14	0	0	0	0	
94168237.1	0	0	0	0	
94093662.82	0	0	0	0	

93931474.31	0	0	0	0	
93894752.13	0	0	0	0	
93868401.45	0	0	0	0	
93802583.12	0	0	0	0	
93629687.32	0	0	0	0	
93612040.16	0	0	0	0	
93363710.82	0	0	0	0	
93224416.14	0	0	0	0	
93181085.71	0	0	0	0	
93145889.5	0	0	0	0	
93122258.16	0	0	0	0	
93081656.81	0	0	0	0	
93062704.97	0	0	0	0	
93046328.27	0	0	0	0	
92718597.7	0	0	0	0	
92441838.77	0	0	0	0	
92222227.77	0	0	0	0	
92196219.24	0	0	0	0	
92073917.57	0	0	0	2	Other
91935566.48	0	0	0	0	
91927509.94	0	0	0	0	
91917971.55	0	0	0	3	Other
91875845.52	0	0	0	0	
91775423.55	0	0	0	0	
91770857.1	0	0	0	0	
91719911.92	0	0	0	0	
91716893.72	0	0	0	0	
91565341.08	0	0	0	0	
91265632.09	0	0	0	0	
91140988.37	0	0	0	0	
90980602.19	0	0	0	0	
90780830.65	0	0	0	0	
90721679.25	0	0	0	7	Other
90688100.07	0	0	0	0	
90428213.93	0	0	0	1	Other
90332939.33	0	0	2	3	Other
90139194.62	0	0	0	0	
90107880.87	0	0	0	0	
89830179.65	0	0	0	0	
89808770.48	0	0	0	0	
89744022.77	0	0	0	0	
89406072.91	0	0	0	0	
89212266.23	0	0	0	0	
88989964.51	0	0	0	0	
88919712.73	0	0	0	0	
88833252.53	0	0	0	0	
88601508.88	0	0	0	0	
88509173.76	0	0	0	0	
88482024.21	0	0	0	0	
88418385.35	0	0	0	0	

88307508.43	0	0	0	0	
88286393.42	0	0	0	0	
88232414.33	0	0	0	0	
88164471.76	0	0	0	0	
87922751.33	0	0	0	2	Other
87867918.9	0	0	0	0	
87586638.44	0	0	0	0	
87542292.69	0	0	0	0	
87512885.65	0	0	0	0	
87223852.16	0	0	0	10	Superpathw
87181229.89	0	0	0	0	
87169864.74	0	0	0	0	
87160473.53	0	0	0	0	
87148288.63	0	0	0	0	
87085114.99	0	0	0	0	
86910841.21	0	0	0	0	
86711072.68	0	0	0	0	
86702094.71	0	0	0	10	Superpathw
86691573.54	0	0	0	0	
86522184.85	0	0	0	0	
86410767.74	0	0	0	0	
86334540.27	0	0	0	0	
86323824.62	0	0	0	0	
85904907.11	0	0	0	0	
85824099.36	0	0	0	0	
85798892.48	0	0	0	0	
85793200.31	0	0	0	0	
85631797.82	0	0	0	0	
85476607.71	0	0	0	0	
85363098.12	0	0	0	0	
85292970.5	0	0	0	3	Other
85226126.98	0	0	0	0	
85163552.36	0	0	0	1	Superpathw
85054903.73	0	0	0	0	
85019500.62	0	0	0	0	
84942261.17	0	0	1	0	
84816424.59	0	0	0	0	
84632323.35	0	0	0	0	
84568582.32	0	0	0	0	
84545397.53	0	0	0	0	
84473208.45	0	0	0	0	
83948766.08	0	0	0	0	
83919958.88	0	0	0	0	
83867990.41	0	0	0	0	
83850322.61	0	0	0	0	
83836553.44	0	0	0	0	
83809921	0	0	0	0	
83526582.63	0	0	0	0	
83518158.78	0	0	0	0	
83497726.86	0	0	0	0	

83424866.65	0	0	0	0	
83390884.1	0	0	0	0	
83215873.72	0	0	0	0	
82928117.76	0	0	0	0	
82847030	0	0	0	0	
82844425.21	0	0	0	3	Other
82805474.13	0	0	0	3	Superpathw
82798805.75	0	0	0	0	
82710184.14	0	0	0	0	
82418075.31	0	0	0	2	Superpathw
82171617.88	0	0	0	0	
81918105.11	0	0	0	0	
81775778.27	0	0	0	0	
81681707.63	0	0	0	1	Other
81676607.67	0	0	0	0	
81577279.4	0	0	0	0	
81526547.17	0	0	0	0	
81398986.14	0	0	0	0	
81366921.73	0	0	0	0	
81316690.09	0	0	0	0	
81076856.28	0	0	0	0	
81054196.35	0	0	0	0	
80986227.8	0	0	0	0	
80984772.32	0	0	0	1	Other
80827873.67	0	0	0	0	
80808845.32	0	0	0	0	
80771998.31	0	0	0	0	
80762865.44	0	0	0	0	
80753824.82	0	0	0	0	
80748324.27	0	0	0	0	
80629907.47	0	0	0	0	
80551531.03	0	0	0	0	
80514291.59	0	0	0	0	
80434044.04	0	0	0	0	
80158584.73	0	0	0	0	
80099958.7	0	0	0	0	
80018200.38	0	0	0	0	
80014542.66	0	0	0	0	
79972818.47	0	0	0	0	
79937025.32	0	0	0	0	
79850379.73	0	0	0	0	
79746251.86	0	0	0	1	Superpathw
79738746.33	0	0	0	0	
79705240.73	0	0	0	0	
79551898.22	0	0	0	0	
79499175.94	0	0	0	0	
79429066.4	0	0	0	0	
79385999.09	0	0	0	0	
79350008.25	0	0	0	0	
79249172.43	0	0	0	0	

79123348.05	0	0	0	0	
78843485.01	0	0	0	0	
78719777.06	0	0	0	1	Other
78628976.54	0	0	0	0	
78569939.59	0	0	0	0	
78546252.07	0	0	0	0	
78456140.64	0	0	0	0	
78353847.18	0	0	0	0	
78302067.71	0	0	0	0	
78266175.52	0	0	0	0	
78188213.98	0	0	0	0	
77851172.52	0	0	0	0	
77725803.9	0	0	0	0	
77384969.67	0	0	4	0	
77338966.87	0	0	0	0	
77222201.68	0	0	0	0	
77173367.15	0	0	0	0	
76949373.51	0	0	0	0	
76891044.79	0	0	0	0	
76819717.68	0	0	0	0	
76678597.49	0	0	0	0	
76660909.41	0	0	0	0	
76586306.86	0	0	0	0	
76431967.29	0	0	0	0	
76320038.87	0	0	0	0	
76283937.61	0	0	0	0	
76049104.81	0	0	0	1	Superpathw
76021455.22	0	0	0	0	
75823410.81	0	0	0	0	
75730764.09	0	0	0	0	
75660409.78	0	0	0	0	
75654158.94	0	0	1	0	
75587487.73	0	0	0	0	
75533678	0	0	0	0	
75475997.26	0	0	0	0	
75254469.23	0	0	0	0	
75229481.59	0	0	0	0	
75149386.79	0	0	0	0	
75133674.6	0	0	0	0	
75100314.69	0	0	0	0	
75090248.21	0	0	0	0	
75013360.91	0	0	0	1	Superpathw
74816462.43	0	0	0	0	
74721415.1	0	0	0	0	
74688840.38	0	0	0	0	
74589667.64	0	0	0	0	
74450361.09	0	0	0	0	
74371379.85	0	0	0	0	
74292617.21	0	0	0	0	
74095984.74	0	0	0	0	

74059185.96	0	0	0	0	
74054956.74	0	0	0	0	
74047297.98	0	0	0	0	
74012411.38	0	0	0	0	
73986986.09	0	0	0	2	Other
73844938.37	0	0	0	0	
73759758.35	0	0	0	0	
73640790.62	0	0	0	0	
73503209.22	0	0	0	0	
73441757.84	0	0	0	0	
73387092.47	0	0	0	1	Other
73290134.69	0	0	0	0	
73195830.97	0	0	0	0	
73138627.52	0	0	0	0	
73051186.36	0	0	0	0	
72873993.74	0	0	0	1	Aspartate su
72731384.52	0	0	0	0	
72702657.83	0	0	0	0	
72608718.19	0	0	0	0	
72379285.77	0	0	0	0	
72333581.83	0	0	0	0	
72056619.97	0	0	0	0	
72021862	0	0	0	0	
71828299.61	0	0	0	0	
71715607.81	0	0	0	0	
71409718.16	0	0	0	3	Other
71366261.93	0	0	0	0	
71365455.77	0	0	0	0	
71046714.4	0	0	0	0	
70989133.01	0	0	0	0	
70896770.91	0	0	0	0	
70793276.81	0	0	0	0	
70736637.73	0	0	0	0	
70669887.09	0	0	0	0	
70624933.96	0	0	0	0	
70589958.83	0	0	0	1	Other
70557315.21	0	0	0	0	
70535132.45	0	0	0	0	
70526941	0	0	0	0	
70458904.22	0	0	0	0	
70314019.06	0	0	0	13	Superpathw
70310667.03	0	0	0	0	
70261269.81	0	0	0	0	
70221612.24	0	0	0	0	
70142966.38	0	0	0	0	
69884665.31	0	0	0	0	
69869037.09	0	0	0	0	
69798570.98	0	0	0	1	Other
69637309.75	0	0	0	0	
69632060.11	0	0	0	0	

69617055.08	0	0	0	0	
69568157.88	0	0	0	0	
69549415.8	0	0	0	0	
69522156.42	0	0	0	3	Aspartate su
69470142.75	0	0	0	0	
69424575.32	0	0	0	0	
69164182.47	0	0	0	0	
69152054.84	0	0	0	0	
69108219.86	0	0	0	0	
68927299.3	0	0	0	0	
68923472.05	0	0	0	0	
68891556.31	0	0	0	0	
68762720.77	0	0	0	0	
68508223.84	0	0	0	0	
68465983.01	0	0	0	0	
68391105.13	0	0	0	0	
68385199.7	0	0	0	0	
68312602.97	0	0	0	0	
68203166.87	0	0	0	0	
68092895.22	0	0	0	0	
68079480.32	0	0	0	0	
68069296.64	0	0	0	0	
67915144.06	0	0	0	0	
67896838.03	0	0	0	0	
67768192.95	0	0	0	0	
67601310.24	0	0	0	0	
67577484.51	0	0	0	0	
67559064.64	0	0	0	0	
67495862.2	0	0	0	0	
67409636.1	0	0	0	0	
67318028.66	0	0	0	0	
67245030.59	0	0	0	0	
67218743.14	0	0	0	0	
67174238.77	0	0	0	0	
67146339.14	0	0	0	2	Other
67097513.24	0	0	0	0	
66874451.87	0	0	0	0	
66816141.64	0	0	0	0	
66756690.99	0	0	0	0	
66710051.47	0	0	0	0	
66699090.22	0	0	0	0	
66671661.37	0	0	0	0	
66653737.08	0	0	0	0	
66618938.98	0	0	0	0	
66558842.14	0	0	0	0	
66078583.99	0	0	0	0	
65987703.09	0	0	0	0	
65932915.78	0	0	0	0	
65875013.48	0	0	0	0	
65824307.44	0	0	0	0	

65727345.07	0	0	0	0	
65701999.14	0	0	0	0	
65543069.87	0	0	0	0	
65354148.03	0	0	0	0	
65331177.85	0	0	0	0	
65306147.11	0	0	0	0	
65201894.34	0	0	0	0	
65170333.94	0	0	0	0	
65163457.76	0	0	0	0	
65069471.45	0	0	0	0	
65000191.53	0	0	0	1	Superpathw
64967162.44	0	0	0	0	
64959455.89	0	0	0	0	
64945757.51	0	0	0	0	
64881129.15	0	0	0	0	
64841422.51	0	0	0	0	
64825376.89	0	0	0	0	
64779838.82	0	0	0	6	Purine nucle
64756148.15	0	0	0	0	
64666150.45	0	0	0	0	
64634125.29	0	0	0	0	
64532401	0	0	0	2	Other
64492219.43	0	0	0	0	
64426766.54	0	0	0	0	
64379214.61	0	0	0	0	
64353549.98	0	0	0	0	
64296235.44	0	0	0	0	
64227728.53	0	0	0	0	
64214678.52	0	0	0	0	
64030656.73	0	0	0	0	
64022816.03	0	0	0	0	
63997594.86	0	0	0	0	
63989570.01	0	0	0	0	
63929715.38	0	0	0	0	
63762698.67	0	0	0	0	
63614906.7	0	0	0	0	
63572910.3	0	0	0	0	
63358737.78	0	0	0	0	
63223883.39	0	0	0	0	
63216810.77	0	0	0	0	
63099794.61	0	0	0	0	
63085618.91	0	0	0	0	
63013537	0	0	0	0	
62935124.98	0	0	0	0	
62922429.48	0	0	0	0	
62901899.97	0	0	0	0	
62740973.06	0	0	0	0	
62480565.53	0	0	0	0	
62440959.11	0	0	0	0	
62401433.43	0	0	0	0	

62299707.48	0	0	0	0	
61993102.47	0	0	0	0	
61892191.08	0	0	0	0	
61852287.86	0	0	0	0	
61846168.4	0	0	0	0	
61827756.39	0	0	0	0	
61800631.08	0	0	0	0	
61570467.15	0	0	0	0	
61451563.17	0	0	0	0	
61445363.01	0	0	0	2	Other
61389737.5	0	0	0	0	
61301534.94	0	0	0	0	
61253444.51	0	0	0	0	
61176185.8	0	0	0	0	
61160735.48	0	0	0	1	Superpathw
61151448.47	0	0	0	1	Other
61006725.47	0	0	0	0	
60869645.84	0	0	0	0	
60865621.51	0	0	0	0	
60707838.27	0	0	0	0	
60682890.81	0	0	0	0	
60648916.49	0	0	0	0	
60640943.1	0	0	0	0	
60541479.23	0	0	0	0	
60539973.5	0	0	0	0	
60513683.33	0	0	0	0	
60471211.7	0	0	0	0	
60427428.24	0	0	0	0	
60332632.74	0	0	0	0	
60316526.4	0	0	1	0	
60297605.33	0	0	0	0	
60269115.05	0	0	0	0	
60198972.22	0	0	0	0	
60161845.72	0	0	0	0	
60147170.46	0	0	0	0	
60092388.79	0	0	0	0	
59951966.48	0	0	0	1	Superpathw
59756838.7	0	0	0	0	
59755304.74	0	0	0	0	
59712034.72	0	0	0	0	
59668531.98	0	0	0	3	Other
59639411.7	0	0	0	0	
59616429.67	0	0	0	0	
59609120.41	0	0	0	0	
59608580.47	0	0	0	0	
59559280.54	0	0	0	0	
59510432.69	0	0	0	0	
59477738.14	0	0	0	0	
59468730.98	0	0	0	0	
59465095.83	0	0	0	0	

59448135.73	0	0	0	0	
59379772.2	0	0	0	0	
59233350.15	0	0	0	0	
59218176.48	0	0	0	0	
59180746.58	0	0	0	0	
59149711.61	0	0	0	0	
59133716.08	0	0	0	0	
59115133.54	0	0	0	0	
59103072.21	0	0	0	0	
59048029.14	0	0	0	0	
58983631.13	0	0	0	0	
58958290.94	0	0	0	0	
58940956.86	0	0	0	0	
58672223.85	0	0	0	4	Other
58665897.05	0	0	0	0	
58440948.69	0	0	0	0	
58430930.4	0	0	0	0	
58415024.02	0	0	0	0	
58306385.89	0	0	0	0	
58289209.64	0	0	0	0	
58282087.74	0	0	0	0	
58277180.93	0	0	0	2	Other
58176079.03	0	0	0	0	
58156296.7	0	0	0	0	
58120775.99	0	0	0	0	
58066163.8	0	0	0	0	
57984950.22	0	0	0	0	
57950642.93	0	0	0	0	
57826461.97	0	0	0	0	
57811778.55	0	0	0	1	Other
57641434.99	0	0	0	0	
57580088.64	0	0	0	0	
57470050.84	0	0	0	0	
57446603.01	0	0	0	0	
57446603.01	0	0	0	0	
57342475.47	0	0	0	1	Other
57135156.78	0	0	0	0	
57130204.33	0	0	0	0	
57088680.04	0	0	0	0	
57006152.82	0	0	0	0	
56966713.33	0	0	0	0	
56935241.51	0	0	0	0	
56882906.05	0	0	0	0	
56698745.55	0	0	0	0	
56468056.13	0	0	0	0	
56453786.41	0	0	0	0	
56442255.95	0	0	0	0	
56357736.12	0	0	0	0	
56309448.19	0	0	0	0	
56298519.51	0	0	0	0	

56260149.44	0	0	0	0	
56254579.2	0	0	0	0	
56251772.56	0	0	0	0	
56247601.65	0	0	0	0	
56096748.08	0	0	0	0	
55994544.17	0	0	0	0	
55945671.12	0	0	0	0	
55906723.14	0	0	0	0	
55901292.54	0	0	0	0	
55715044.09	0	0	0	0	
55705035.6	0	0	0	0	
55669601.56	0	0	0	0	
55658686	0	0	0	0	
55635960.18	0	0	0	0	
55625684.51	0	0	0	1	Other
55624418.62	0	0	0	0	
55535700.83	0	0	0	0	
55506748.51	0	0	0	0	
55296936.78	0	0	0	0	
55288903.42	0	0	0	0	
55270563.72	0	0	0	0	
55193250.86	0	0	0	0	
55165223.5	0	0	0	0	
55128614.38	0	0	0	0	
55116303.96	0	0	0	0	
54980917.19	0	0	0	0	
54912425.2	0	0	0	0	
54886957.98	0	0	0	0	
54842947.49	0	0	0	0	
54840356.62	0	0	0	0	
54828508.78	0	0	0	0	
54756357.63	0	0	0	0	
54752759.67	0	0	0	0	
54729879.36	0	0	0	0	
54721458.7	0	0	0	0	
54683960.14	0	0	0	0	
54662760.23	0	0	0	0	
54538352.16	0	0	0	1	Other
54384401	0	0	0	0	
54365950.03	0	0	0	1	Superpathw
54323573.92	0	0	0	0	
54267603.08	0	0	0	0	
54253533.67	0	0	0	0	
54250999.26	0	0	0	0	
54044814.24	0	0	0	0	
54010901.55	0	0	0	0	
53916370.97	0	0	2	0	
53914004.62	0	0	0	0	
53809203.52	0	0	0	0	
53772848.65	0	0	0	0	

53763740.8	0	0	0	0	
53697028.89	0	0	0	0	
53656944.85	0	0	0	0	
53607806.6	0	0	0	0	
53552095.67	0	0	0	0	
53433780.64	0	0	0	0	
53425998.49	0	0	0	0	
53404018.93	0	0	0	0	
53390233.94	0	0	0	0	
53340691.67	0	0	0	0	
53323590.91	0	0	0	1	Superpathw
53158287.27	0	0	0	0	
53155880.69	0	0	0	0	
53102256.23	0	0	0	0	
53092191.86	0	0	0	0	
53085215.15	0	0	0	0	
52973273.47	0	0	0	0	
52882629.29	0	0	0	0	
52873632.89	0	0	0	0	
52720913.06	0	0	0	0	
52690059.83	0	0	0	0	
52624192.22	0	0	0	0	
52560479.15	0	0	0	0	
52362293.31	0	0	0	0	
52278213.84	0	0	0	0	
52031708.46	0	0	0	0	
52005251.73	0	0	3	0	
51967696.4	0	0	0	0	
51857557.38	0	0	0	0	
51765899.12	0	0	0	0	
51755851.77	0	0	0	0	
51747260.05	0	0	0	0	
51723794.99	0	0	0	0	
51654336.67	0	0	0	0	
51601391.41	0	0	0	0	
51600223.49	0	0	0	0	
51590370.62	0	0	0	1	Superpathw
51502052.35	0	0	0	0	
51493021.62	0	0	0	0	
51480905.16	0	0	0	0	
51476026.81	0	0	0	0	
51458523.25	0	0	0	0	
51432721.17	0	0	0	0	
51427263.19	0	0	0	0	
51372907.42	0	0	0	0	
51372524.62	0	0	0	0	
51340349.31	0	0	0	0	
51265029.24	0	0	0	0	
51259797.87	0	0	0	2	Other
51240036.41	0	0	3	1	Superpathw

51128381.18	0	0	0	0	
51119286.46	0	0	0	0	
50749765.96	0	0	0	0	
50628439.27	0	0	0	0	
50555822.63	0	0	0	0	
50508841.51	0	0	0	0	
50489693.49	0	0	0	0	
50486393.68	0	0	3	1	Superpathw
50466023.55	0	0	0	0	
50437783.03	0	0	0	0	
50417812.57	0	0	0	0	
50293909.62	0	0	0	0	
50201942	0	0	0	0	
50185416.08	0	0	0	0	
50156186.31	0	0	0	0	
50154686.73	0	0	0	0	
50143820.06	0	0	0	0	
50129671.08	0	0	0	0	
50000566.69	0	0	0	0	
49881710.22	0	0	0	0	
49856090.76	0	0	0	0	
49853496.92	0	0	0	0	
49777493.94	0	0	0	0	
49708150.65	0	0	0	0	
49698899.08	0	0	0	0	
49684352.7	0	0	0	0	
49674112.88	0	0	0	0	
49567550.27	0	0	0	0	
49559624.79	0	0	0	0	
49544768.89	0	0	0	0	
49469761.54	0	0	0	0	
49469721.51	0	0	0	0	
49468314.56	0	0	0	0	
49455186.78	0	0	0	0	
49330384.37	0	0	0	0	
49327117.11	0	0	0	0	
49190949.16	0	0	0	0	
49180489.4	0	0	0	4	Other
49121922.99	0	0	0	0	
49111372.78	0	0	0	0	
49009072	0	0	0	0	
49003530.82	0	0	0	0	
49001235.93	0	0	0	0	
48992898.33	0	0	0	1	Superpathw
48959722.01	0	0	0	0	
48872754.23	0	0	0	0	
48862389.5	0	0	0	0	
48800807.23	0	0	0	0	
48704674.11	0	0	0	0	
48702201.47	0	0	0	0	

48683604.65	0	0	0	1	Other
48674624.04	0	0	0	0	
48673988.31	0	0	0	0	
48652308.69	0	0	0	13	Superpathw
48635661.96	0	0	0	0	
48508929.79	0	0	0	0	
48458175.5	0	0	0	0	
48447043.61	0	0	0	0	
48440983.3	0	0	0	0	
48377883.34	0	0	0	0	
48288253.81	0	0	0	0	
48284862.5	0	0	0	0	
48257991.78	0	0	0	1	Superpathw
48256721.2	0	0	0	0	
48180496.17	0	0	0	1	Other
48167782.03	0	0	0	0	
48097652.31	0	0	0	0	
47986621.92	0	0	0	0	
47955215.41	0	0	0	0	
47915061.26	0	0	0	0	
47877117.69	0	0	0	0	
47777507	0	0	0	0	
47757292.9	0	0	0	0	
47744316.2	0	0	0	0	
47715198.97	0	0	0	0	
47692004.46	0	0	0	0	
47689796.71	0	0	0	0	
47667926.81	0	0	0	0	
47295607.73	0	0	0	0	
47262703.3	0	0	0	0	
47254643.23	0	0	0	0	
47241324.31	0	0	0	0	
47192842.52	0	0	0	0	
47143457.78	0	0	0	0	
47133530.04	0	0	0	0	
47112769.65	0	0	0	0	
47070441.44	0	0	0	0	
47040604.77	0	0	0	1	Superpathw
47032698.71	0	0	0	15	Superpathw
46886173.74	0	0	0	0	
46867416.98	0	0	0	0	
46848134.65	0	0	0	0	
46719164.62	0	0	0	0	
46705242.01	0	0	0	0	
46696670.07	0	0	0	0	
46691362.68	0	0	0	0	
46660555.23	0	0	0	0	
46632412.39	0	0	0	0	
46620881.22	0	0	0	0	
46490544.73	0	0	0	0	

46431426.38	0	0	0	0	
46429432.77	0	0	0	0	
46368084.47	0	0	0	0	
46287910.65	0	0	1	0	
46277595.88	0	0	0	0	
46268777.61	0	0	0	0	
46236067.51	0	0	0	0	
46228824.09	0	0	0	0	
46123291.25	0	0	0	0	
46099685.87	0	0	0	0	
46079945.79	0	0	0	0	
46047408.49	0	0	0	0	
45958934.18	0	0	0	0	
45922033.48	0	0	0	0	
45910512.05	0	0	0	0	
45857017.08	0	0	0	0	
45842413.66	0	0	0	0	
45823477.56	0	0	0	0	
45821029.36	0	0	0	0	
45795296.55	0	0	0	0	
45772374.17	0	0	0	0	
45748039.38	0	0	0	0	
45641530.04	0	0	0	1	Superpathw
45532256.85	0	0	0	0	
45516059.12	0	0	0	0	
45496999	0	0	0	0	
45415147.95	0	0	0	0	
45349683.71	0	0	0	1	Aspartate su
45346129.85	0	0	0	0	
45270597.35	0	0	0	0	
45187490.67	0	0	0	0	
45080851.41	0	0	0	0	
45058224.51	0	0	0	0	
45021457.74	0	0	0	0	
44942878.67	0	0	0	10	Superpathw
44937810.6	0	0	0	0	
44856853.35	0	0	0	0	
44820808.66	0	0	0	0	
44804365.25	0	0	0	0	
44726497.4	0	0	0	0	
44720995.06	0	0	0	0	
44710507.49	0	0	0	0	
44662339.44	0	0	0	0	
44613923.16	0	0	0	0	
44569020.32	0	0	0	1	Superpathw
44564811.36	0	0	0	0	
44526063.12	0	0	0	0	
44427909.08	0	0	0	0	
44426861.44	0	0	0	0	
44325571.14	0	0	0	0	

44305015.25	0	0	0	0	
44178169.71	0	0	0	7	Other
44065588.75	0	0	0	0	
43982264.41	0	0	0	0	
43921206.94	0	0	0	0	
43836134.37	0	0	0	0	
43825164.79	0	0	0	0	
43722803.32	0	0	0	0	
43660424.37	0	0	0	15	Superpathw
43609160.35	0	0	0	0	
43592431.95	0	0	0	0	
43591272.56	0	0	0	0	
43542866.69	0	0	0	0	
43531727.42	0	0	0	0	
43485146.92	0	0	0	2	Other
43384356.83	0	0	0	0	
43358834.77	0	0	0	0	
43288697.84	0	0	0	0	
43229831.9	0	0	0	0	
43219081.48	0	0	0	0	
43205697.48	0	0	0	0	
43153723.92	0	0	0	0	
43133558.7	0	0	0	0	
43131463.9	0	0	0	0	
43118162.8	0	0	0	0	
43085598.46	0	0	0	0	
43078288.13	0	0	0	0	
43035212.83	0	0	0	0	
42996594.67	0	0	0	0	
42759142.72	0	0	0	0	
42737296.41	0	0	0	0	
42730277	0	0	0	0	
42730277	0	0	0	0	
42650544.5	0	0	0	0	
42622185.57	0	0	0	0	
42601624.1	0	0	0	0	
42555856.7	0	0	0	0	
42418218.16	0	0	0	0	
42380686.99	0	0	0	1	Other
42368203.37	0	0	0	0	
42328473.34	0	0	0	0	
42262925.96	0	0	0	0	
42245417.52	0	0	0	0	
42112024.13	0	0	0	3	Other
42106256.42	0	0	0	0	
42036068.81	0	0	0	0	
42034721.94	0	0	0	0	
41875391.04	0	0	0	0	
41852825.77	0	0	0	0	
41810311.67	0	0	0	0	

41789268.49	0	0	0	0	
41728137.97	0	0	0	0	
41681736.14	0	0	0	0	
41665101.83	0	0	0	0	
41585303.6	0	0	0	0	
41556883.53	0	0	0	0	
41549415.21	0	0	0	0	
41515026.03	0	0	0	1	Other
41473032	0	0	0	0	
41393849.49	0	0	0	0	
41390092.57	0	0	0	0	
41378651.73	0	0	0	0	
41367728.64	0	0	0	0	
41277831.63	0	0	0	21	Other
41261685.31	0	0	0	0	
41258100.39	0	0	0	0	
41257316.72	0	0	0	0	
41254452.76	0	0	0	0	
41249756.67	0	0	0	5	Other
41234809.52	0	0	0	1	Superpathw
41166207.13	0	0	0	0	
41159937.06	0	0	0	4	Superpathw
41152396.61	0	0	0	0	
41112230.19	0	0	0	0	
41107838.51	0	0	0	0	
41063491.7	0	0	0	0	
41056731.39	0	0	0	0	
41047433.32	0	0	0	0	
41039731.11	0	0	0	0	
41026923.4	0	0	0	0	
41009263.49	0	0	0	0	
40998577.65	0	0	0	0	
40949828.66	0	0	0	0	
40944599.56	0	0	0	0	
40879972.6	0	0	0	0	
40829948.34	0	0	0	0	
40808823.46	0	0	0	0	
40788365.01	0	0	0	0	
40788365.01	0	0	0	0	
40788365.01	0	0	0	0	
40777070.54	0	0	0	0	
40768257.99	0	0	0	0	
40763690.06	0	0	0	0	
40755618.95	0	0	0	0	
40748101.14	0	0	0	0	
40713607.11	0	0	0	0	
40712046.17	0	0	0	0	
40658765.21	0	0	0	0	
40635186.76	0	0	0	0	
40581284.83	0	0	0	0	

40534976.81	0	0	0	0	
40456778.98	0	0	0	0	
40426135.73	0	0	0	1	Other
40391617.34	0	0	0	0	
40368412.77	0	0	0	0	
40352381.88	0	0	0	0	
40338311.6	0	0	0	0	
40260942.1	0	0	0	0	
40245240.02	0	0	0	0	
40219433.49	0	0	0	0	
40217783.57	0	0	0	0	
40206302.77	0	0	0	0	
40169304.21	0	0	0	0	
40165980.54	0	0	0	0	
40130349.09	0	0	0	0	
40123571.59	0	0	0	0	
40070446.04	0	0	0	0	
40059512.05	0	0	0	0	
40046577.84	0	0	0	0	
40033101.62	0	0	0	0	
40030091.34	0	0	0	0	
40018217.1	0	0	0	0	
39989389.3	0	0	0	0	
39977100.59	0	0	0	0	
39955718.64	0	0	0	0	
39949394.68	0	0	0	0	
39797394.36	0	0	0	2	Superpathw
39763818.16	0	0	0	0	
39672662.92	0	0	0	0	
39643720.85	0	0	0	0	
39605371.51	0	0	0	0	
39576794.98	0	0	0	0	
39547650.19	0	0	0	0	
39536121.63	0	0	0	0	
39475972.11	0	0	0	0	
39399814.49	0	0	0	0	
39375910.28	0	0	0	0	
39338539.59	0	0	0	0	
39336097.06	0	0	0	0	
39228488.03	0	0	0	0	
39210415.32	0	0	0	0	
39133545.07	0	0	0	0	
39127253.33	0	0	0	0	
39117821.02	0	0	0	0	
39098681.43	0	0	0	0	
39071604.84	0	0	0	0	
39048952.49	0	0	0	0	
39045508.83	0	0	0	0	
39025783.01	0	0	0	3	Superpathw
39024881.56	0	0	0	0	

39016062.66	0	0	0	0	
39000821.01	0	0	0	0	
38938415.08	0	0	0	0	
38914313.66	0	0	0	0	
38860799.29	0	0	0	0	
38851935.56	0	0	0	0	
38840401.01	0	0	0	3	Superpathw
38818580.52	0	0	0	0	
38781683.03	0	0	0	0	
38775267.24	0	0	0	3	Other
38751775.87	0	0	0	0	
38751775.87	0	0	0	0	
38747857.93	0	0	0	0	
38693658.23	0	0	0	0	
38682518.83	0	0	0	0	
38668958.39	0	0	0	0	
38633871.52	0	0	0	0	
38624590.17	0	0	0	0	
38620678.43	0	0	0	0	
38591971.21	0	0	0	0	
38585436.05	0	0	0	0	
38554753.44	0	0	0	0	
38549346.37	0	0	0	0	
38503589.82	0	0	0	5	Other
38500612.01	0	0	0	0	
38477392.66	0	0	0	0	
38440432.56	0	0	0	0	
38431797.1	0	0	0	0	
38391554.94	0	0	0	0	
38367069.14	0	0	0	0	
38364591.01	0	0	0	0	
38356123.13	0	0	0	2	Superpathw
38304526.59	0	0	0	0	
38220099.29	0	0	0	0	
38183364.98	0	0	0	0	
38177741.64	0	0	0	6	Superpathw
38177741.64	0	0	0	1	Superpathw
38160629.99	0	0	0	0	
38156782.97	0	0	0	0	
38144411.5	0	0	6	2	Other
38107148.14	0	0	0	0	
38092057.32	0	0	0	0	
38058940.99	0	0	0	5	Other
38022409.84	0	0	1	0	
37984674.78	0	0	0	0	
37983786.14	0	0	0	5	Other
37919197.34	0	0	0	0	
37896567.17	0	0	0	0	
37869219.74	0	0	0	0	
37850655.96	0	0	0	0	

37832242.58	0	0	0	0	
37815254.88	0	0	0	0	
37795669.32	0	0	0	0	
37724587.26	0	0	0	0	
37724371.28	0	0	0	0	
37700756.67	0	0	0	0	
37660542.3	0	0	0	0	
37659396.67	0	0	0	1	Other
37640419.58	0	0	0	1	Other
37635135.81	0	0	0	0	
37625597.68	0	0	0	0	
37574163.52	0	0	0	0	
37568352.89	0	0	0	0	
37544389.53	0	0	0	0	
37479092.44	0	0	0	0	
37450721.72	0	0	0	0	
37334913.4	0	0	0	0	
37314404.96	0	0	0	0	
37223304.22	0	0	0	0	
37189820.36	0	0	0	0	
37149751.39	0	0	0	3	Superpathw
37139509.53	0	0	0	0	
37050577.45	0	0	0	0	
37033867.95	0	0	0	0	
37026270.22	0	0	0	0	
36960521.44	0	0	0	0	
36949345.32	0	0	0	0	
36851449.22	0	0	0	0	
36828343.12	0	0	0	0	
36799490.42	0	0	1	1	Other
36779027.07	0	0	0	0	
36775175.01	0	0	0	1	Other
36745223.47	0	0	0	0	
36745206.48	0	0	0	0	
36736065.53	0	0	0	0	
36722055.37	0	0	0	0	
36688115.61	0	0	0	0	
36616778.86	0	0	0	0	
36591933.49	0	0	0	0	
36548460.57	0	0	0	0	
36387733.84	0	0	0	0	
36385329.9	0	0	0	0	
36357540.27	0	0	0	0	
36315635.24	0	0	0	0	
36301634.92	0	0	0	0	
36279017.34	0	0	0	0	
36248126.41	0	0	0	0	
36236810.35	0	0	0	0	
36223518.7	0	0	0	0	
36178377.5	0	0	0	0	

36145914.08	0	0	0	0	
36126079.07	0	0	0	1	Superpathw
36120274.86	0	0	0	0	
36111635.47	0	0	0	0	
36095229.05	0	0	0	0	
36023630.51	0	0	0	0	
35953375.93	0	0	0	0	
35953355.39	0	0	0	2	Superpathw
35878850.52	0	0	0	0	
35864536.11	0	0	0	0	
35853514.25	0	0	0	0	
35831377.5	0	0	0	0	
35714590.59	0	0	0	0	
35690913.49	0	0	0	0	
35679917.65	0	0	0	0	
35649594.7	0	0	0	0	
35612250.25	0	0	0	0	
35590576.27	0	0	0	0	
35586174.54	0	0	0	0	
35586174.54	0	0	0	0	
35572692.39	0	0	0	0	
35542609.33	0	0	0	0	
35514754.55	0	0	0	0	
35387684.94	0	0	0	0	
35376407.93	0	0	0	3	Other
35309458.79	0	0	0	0	
35288973.26	0	0	0	0	
35240785.63	0	0	0	0	
35210747.63	0	0	0	0	
35209264.2	0	0	0	0	
35196741.76	0	0	0	0	
35168646.51	0	0	0	0	
35165594.6	0	0	0	0	
35159690.14	0	0	0	0	
35156742.04	0	0	0	0	
35151184.1	0	0	0	0	
35148932.59	0	0	0	0	
35023432.46	0	0	0	0	
35016620.28	0	0	0	0	
35011724.95	0	0	0	1	Superpathw
35007692.79	0	0	0	0	
35003655.82	0	0	0	0	
35003164.97	0	0	0	0	
34914534.54	0	0	0	0	
34914247.13	0	0	0	0	
34903455.03	0	0	0	0	
34888718.87	0	0	0	0	
34874992.88	0	0	0	0	
34852933.69	0	0	0	0	
34819557.8	0	0	1	0	

34797993.42	0	0	0	0	
34782642.74	0	0	0	0	
34741934.19	0	0	0	0	
34736083.57	0	0	0	6	Purine nucle
34725171.12	0	0	0	0	
34711036.93	0	0	0	0	
34700519.26	0	0	0	0	
34683426.64	0	0	0	0	
34676360.81	0	0	0	0	
34672069.29	0	0	0	0	
34640098.05	0	0	0	0	
34638389.64	0	0	0	0	
34625229.39	0	0	0	0	
34591879	0	0	0	0	
34591382.97	0	0	0	12	Superpathw
34542121.66	0	0	0	0	
34521390.35	0	0	0	0	
34484367.79	0	0	0	0	
34419356.09	0	0	0	0	
34407009.58	0	0	0	0	
34345783.97	0	0	0	0	
34317136.37	0	0	0	0	
34309959.24	0	0	0	0	
34303255.17	0	0	0	0	
34252856.11	0	0	0	0	
34232446.46	0	0	0	0	
34220894.96	0	0	0	0	
34207794	0	0	0	0	
34125412.17	0	0	0	0	
34112939.05	0	0	0	0	
34058995.16	0	0	0	0	
34019382.12	0	0	0	23	Superpathw
34001638.88	0	0	0	0	
33986119.35	0	0	0	0	
33975528.25	0	0	0	0	
33952244.59	0	0	0	0	
33951920.48	0	0	0	0	
33939548.76	0	0	0	0	
33937836.45	0	0	0	1	Superpathw
33929615.52	0	0	0	0	
33918628.45	0	0	0	0	
33897551.77	0	0	0	0	
33861965.23	0	0	0	0	
33842666.31	0	0	0	0	
33835867.36	0	0	0	0	
33766732.03	0	0	0	0	
33744839.24	0	0	0	0	
33729604.35	0	0	0	0	
33725925.6	0	0	0	0	
33714591.07	0	0	0	0	

33700061.63	0	0	0	0	
33629413.29	0	0	0	0	
33558021.75	0	0	0	0	
33554759.1	0	0	0	0	
33542779.9	0	0	0	0	
33542411.94	0	0	0	0	
33509139.64	0	0	0	0	
33459104.19	0	0	0	0	
33434746.9	0	0	0	1	Superpathw
33404496.23	0	0	0	0	
33323182.06	0	0	0	0	
33309681.71	0	0	0	0	
33298479.41	0	0	0	0	
33270134.58	0	0	0	0	
33249862.66	0	0	0	0	
33191578.62	0	0	0	0	
33075921.66	0	0	0	0	
33056183.2	0	0	0	0	
33028993.19	0	0	0	0	
33027348.85	0	0	0	0	
32992741.86	0	0	0	3	Other
32983468.91	0	0	0	0	
32979409.15	0	0	0	0	
32973839.36	0	0	0	1	Superpathw
32962104.55	0	0	0	0	
32951103.74	0	0	0	0	
32943391.27	0	0	0	0	
32932484.57	0	0	0	0	
32928802.97	0	0	0	0	
32864767.11	0	0	0	0	
32837023.42	0	0	0	0	
32829438.91	0	0	0	0	
32765471.7	0	0	0	0	
32751812.87	0	0	0	1	Other
32742832.17	0	0	0	0	
32738316.52	0	0	0	0	
32727594.92	0	0	0	0	
32666922.58	0	0	0	0	
32654523.15	0	0	0	0	
32645512.67	0	0	0	0	
32588466.9	0	0	0	0	
32564536.1	0	0	0	0	
32558214.34	0	0	0	0	
32557336.42	0	0	0	2	Superpathw
32541084.09	0	0	0	0	
32523569.47	0	0	0	0	
32512507.48	0	0	0	4	Other
32455715.95	0	0	0	0	
32453744.43	0	0	0	0	
32388510.87	0	0	0	0	

32363755.11	0	0	0	0	
32363217.59	0	0	0	0	
32348715.16	0	0	0	0	
32333545.29	0	0	0	0	
32320556.9	0	0	0	1	Superpathw
32305456.27	0	0	0	0	
32291235.82	0	0	0	0	
32253820	0	0	0	0	
32238643.1	0	0	0	0	
32238586.84	0	0	0	0	
32174282.58	0	0	0	0	
32113270.34	0	0	0	0	
32083314.29	0	0	0	0	
32051724.6	0	0	0	0	
32035224.72	0	0	0	11	Superpathw
32035073.09	0	0	0	0	
32032934.75	0	0	0	0	
32032934.75	0	0	0	0	
31974146.33	0	0	0	0	
31961211.19	0	0	0	0	
31943891.51	0	0	0	0	
31924893.31	0	0	0	0	
31889889.75	0	0	0	0	
31854898.94	0	0	0	0	
31808155.54	0	0	0	0	
31775611.11	0	0	0	0	
31697376.17	0	0	0	0	
31636009.04	0	0	0	0	
31627165.15	0	0	0	0	
31612988.12	0	0	0	0	
31599813.35	0	0	0	0	
31591177.71	0	0	0	3	Superpathw
31583487.09	0	0	0	0	
31569020.69	0	0	0	0	
31563635.17	0	0	0	1	Superpathw
31499264.99	0	0	0	0	
31496202.8	0	0	0	0	
31492536.72	0	0	0	0	
31491228.36	0	0	0	0	
31485674.4	0	0	0	0	
31482869.77	0	0	0	0	
31423339.79	0	0	0	0	
31395248.78	0	0	0	1	Superpathw
31368923.7	0	0	0	5	Superpathw
31364900.56	0	0	0	0	
31357385.66	0	0	0	0	
31338788.24	0	0	0	0	
31320335.41	0	0	0	0	
31299684.12	0	0	0	0	
31297817.03	0	0	0	0	

31291784.88	0	0	0	0	
31281388.87	0	0	0	0	
31235527.7	0	0	0	0	
31205676.67	0	0	0	0	
31187183.74	0	0	0	0	
31181730.86	0	0	0	0	
31178711.9	0	0	0	0	
31163241.33	0	0	0	0	
31151703.52	0	0	0	0	
31132672.78	0	0	0	0	
31122176.88	0	0	0	0	
31113402.81	0	0	0	0	
31051660.68	0	0	0	0	
31045980.62	0	0	0	0	
31040660.99	0	0	0	0	
31040295.5	0	0	0	0	
30984693.83	0	0	0	0	
30966211.62	0	0	0	0	
30930304.98	0	0	0	0	
30929634.1	0	0	0	0	
30919638.49	0	0	0	0	
30911329.4	0	0	0	0	
30905173.74	0	0	0	0	
30829734.82	0	0	0	0	
30795442.89	0	0	0	0	
30783684.34	0	0	0	1	Other
30773267.52	0	0	0	0	
30742177.04	0	0	0	0	
30645904.55	0	0	0	0	
30614683.76	0	0	0	23	Superpathw
30584400.99	0	0	0	0	
30554686.19	0	0	0	0	
30536356.04	0	0	0	0	
30485931.07	0	0	0	0	
30481684.98	0	0	0	3	Superpathw
30470017.53	0	0	0	0	
30433654.26	0	0	0	17	Aspartate su
30431837.95	0	0	0	0	
30423106.77	0	0	0	0	
30367221.68	0	0	0	1	Superpathw
30316380.81	0	0	0	0	
30291459.38	0	0	0	0	
30272803.12	0	0	0	0	
30226643.76	0	0	0	0	
30210085.41	0	0	0	0	
30190176.76	0	0	0	0	
30134480.55	0	0	0	0	
30129024.08	0	0	0	0	
30119381.85	0	0	0	0	
30101344.09	0	0	0	0	

30058074.79	0	0	0	0	
30057981.95	0	0	0	1	Other
30057726.2	0	0	0	0	
30052012.8	0	0	0	0	
30002297	0	0	0	0	
29997399.43	0	0	0	0	
29997365.01	0	0	0	0	
29990434.15	0	0	0	0	
29964201.45	0	0	0	0	
29937902.58	0	0	0	1	Superpathw
29911626.57	0	0	0	0	
29897380.52	0	0	0	0	
29892345.76	0	0	0	0	
29891085.29	0	0	0	0	
29880686.49	0	0	0	0	
29857984.2	0	0	0	0	
29857830.22	0	0	0	0	
29818585.87	0	0	0	0	
29788615.63	0	0	0	0	
29782316.97	0	0	0	1	Superpathw
29760141.26	0	0	0	1	Superpathw
29742723.73	0	0	0	0	
29736402.14	0	0	0	0	
29725884.6	0	0	0	0	
29714543.89	0	0	0	0	
29690234.48	0	0	0	0	
29673024.76	0	0	0	0	
29655501.11	0	0	0	0	
29644274.83	0	0	0	3	Other
29639435.72	0	0	0	0	
29619059.14	0	0	0	0	
29608025.43	0	0	0	1	Superpathw
29581493.38	0	0	0	0	
29557064.71	0	0	0	0	
29553064.49	0	0	0	0	
29540254.73	0	0	0	0	
29520258.19	0	0	0	0	
29513923.16	0	0	0	0	
29484218.37	0	0	0	0	
29456087.21	0	0	0	0	
29454405.97	0	0	0	0	
29425029.88	0	0	0	0	
29407975.46	0	0	0	0	
29395502.51	0	0	0	0	
29392910.01	0	0	0	0	
29391737.42	0	0	0	0	
29388775.34	0	0	0	0	
29385795.88	0	0	0	0	
29379599.32	0	0	0	0	
29358792.09	0	0	0	0	

29351167.1	0	0	0	0	
29346141.41	0	0	0	0	
29346001.01	0	0	0	0	
29337880.55	0	0	0	0	
29334574.9	0	0	0	0	
29319628.56	0	0	0	0	
29289865.82	0	0	0	0	
29261825.43	0	0	0	0	
29241094.6	0	0	0	0	
29235410.9	0	0	0	1	Other
29225745.28	0	0	0	0	
29198123.08	0	0	0	0	
29162136.97	0	0	0	3	Superpathw
29149474.72	0	0	0	0	
29143253.35	0	0	0	0	
29130677.26	0	0	0	0	
29114449.64	0	0	0	0	
29091029.56	0	0	0	0	
29082623.39	0	0	0	0	
29055512.68	0	0	0	0	
29003372.54	0	0	0	0	
28984297.93	0	0	0	0	
28960636.67	0	0	0	0	
28947555.03	0	0	0	0	
28938786.3	0	0	0	0	
28916972.82	0	0	0	1	Superpathw
28906390.79	0	0	0	0	
28891817.91	0	0	0	0	
28889862.43	0	0	0	0	
28834789.98	0	0	0	0	
28834495.94	0	0	0	0	
28830742.32	0	0	0	0	
28810833.16	0	0	0	0	
28803573.59	0	0	0	0	
28796793.17	0	0	0	0	
28791927.88	0	0	0	0	
28770517.87	0	0	0	0	
28746362.52	0	0	0	0	
28737849.88	0	0	0	0	
28734187.48	0	0	0	0	
28731540.09	0	0	0	0	
28726866.49	0	0	0	0	
28702077.13	0	0	0	0	
28692758.69	0	0	0	0	
28676211.13	0	0	0	0	
28666009.89	0	0	0	0	
28654488.95	0	0	0	0	
28649881.38	0	0	0	0	
28646479.74	0	0	0	0	
28632967.28	0	0	0	0	

28624453.06	0	0	0	0	
28606807.09	0	0	0	0	
28567917.95	0	0	1	1	Other
28489805.13	0	0	0	0	
28440927.31	0	0	0	0	
28405283.13	0	0	0	0	
28384796.5	0	0	0	0	
28338609.83	0	0	0	0	
28322946.78	0	0	0	0	
28316807.12	0	0	0	0	
28302790.49	0	0	0	0	
28286789.58	0	0	0	0	
28277160.56	0	0	0	0	
28251704.74	0	0	0	0	
28171577.17	0	0	0	0	
28169688.95	0	0	0	1	Other
28135052.37	0	0	0	0	
28116290.61	0	0	0	0	
28064083.33	0	0	0	0	
28062357.26	0	0	0	0	
28058914.89	0	0	0	0	
28017483.41	0	0	0	0	
28017483.41	0	0	0	0	
28009937.51	0	0	0	0	
27998184.51	0	0	0	0	
27958148.03	0	0	0	0	
27901104.95	0	0	0	0	
27893285.67	0	0	0	0	
27891242.17	0	0	0	0	
27860088.36	0	0	0	0	
27772937.48	0	0	0	0	
27709528.38	0	0	0	0	
27682357.71	0	0	0	0	
27650511.63	0	0	0	0	
27640458.54	0	0	0	3	Other
27629238.33	0	0	0	0	
27624878.12	0	0	0	0	
27607355.7	0	0	0	0	
27590492.39	0	0	0	0	
27578720.99	0	0	0	0	
27557395.54	0	0	0	0	
27543818.95	0	0	0	0	
27523661.65	0	0	0	0	
27520504.06	0	0	0	0	
27495109.08	0	0	0	0	
27433218.09	0	0	0	0	
27402279.29	0	0	0	0	
27380205.47	0	0	0	0	
27358459.94	0	0	0	0	
27334876.72	0	0	0	0	

27325396.5	0	0	0	31	Superpathw
27304807.86	0	0	0	0	
27299808.91	0	0	0	0	
27286981.75	0	0	0	0	
27268889.14	0	0	0	0	
27232749.56	0	0	0	0	
27223013.1	0	0	0	0	
27210123.8	0	0	0	0	
27185264.42	0	0	0	0	
27136317.76	0	0	0	0	
27129518.92	0	0	0	0	
27122896.95	0	0	0	0	
27112191.41	0	0	0	0	
27099811.02	0	0	0	0	
26976349.03	0	0	0	0	
26971271.49	0	0	0	0	
26945338.01	0	0	0	0	
26893453.29	0	0	0	0	
26892006.86	0	0	0	0	
26889571.77	0	0	0	0	
26887505.47	0	0	0	0	
26846949.84	0	0	0	0	
26825072.48	0	0	0	0	
26822733.9	0	0	0	0	
26797418.82	0	0	0	0	
26793699.34	0	0	0	0	
26775588.27	0	0	0	0	
26771999.27	0	0	0	0	
26745217.58	0	0	0	1	Other
26724777.82	0	0	0	0	
26693691.51	0	0	0	0	
26667022.33	0	0	0	0	
26646767.18	0	0	0	0	
26637077.51	0	0	0	0	
26625149.21	0	0	0	0	
26617330.52	0	0	0	0	
26605432.19	0	0	0	0	
26587090.85	0	0	0	0	
26567579.71	0	0	0	0	
26553340.41	0	0	0	0	
26530570.83	0	0	0	0	
26499746.27	0	0	0	0	
26476897.12	0	0	0	0	
26471046.84	0	0	0	4	Other
26469675.19	0	0	0	0	
26432216.5	0	0	0	0	
26425639.39	0	0	0	0	
26354862.91	0	0	0	0	
26339577.86	0	0	0	0	
26334550.75	0	0	0	0	

26315176.91	0	0	0	0	
26309376.29	0	0	0	0	
26294148.4	0	0	0	0	
26237191.47	0	0	0	0	
26223275.8	0	0	0	0	
26214348.8	0	0	0	0	
26214348.8	0	0	0	0	
26177127.23	0	0	0	0	
26111524.53	0	0	0	0	
26100944.33	0	0	0	0	
26079663.86	0	0	0	0	
26066225.06	0	0	0	0	
26063682.83	0	0	0	0	
26046796.66	0	0	0	1	Superpathw
26039924.63	0	0	0	0	
26032137.24	0	0	0	0	
26012679.28	0	0	0	0	
26004351.25	0	0	0	0	
25969177.35	0	0	0	0	
25960340.01	0	0	0	0	
25922309.64	0	0	0	0	
25921743.17	0	0	0	0	
25914080.49	0	0	0	0	
25900957.27	0	0	0	0	
25891834.22	0	0	0	0	
25879465.78	0	0	0	0	
25878519.49	0	0	0	0	
25877510.73	0	0	0	0	
25831274.92	0	0	0	0	
25817582.25	0	0	0	0	
25811974.21	0	0	0	0	
25788575.33	0	0	0	0	
25782214.03	0	0	0	0	
25776702.48	0	0	0	0	
25768239.11	0	0	0	0	
25739321.72	0	0	0	0	
25733830.75	0	0	0	0	
25723186.41	0	0	0	0	
25715620.23	0	0	0	0	
25710351.4	0	0	0	0	
25683114.12	0	0	0	0	
25655880.48	0	0	0	0	
25626394.71	0	0	0	1	Superpathw
25611144.53	0	0	0	0	
25604111.43	0	0	0	0	
25583418.3	0	0	0	2	Other
25536024.44	0	0	0	0	
25524397.94	0	0	0	0	
25524165.81	0	0	0	0	
25509742.24	0	0	0	0	

25459864.69	0	0	0	0	
25446913.46	0	0	0	0	
25441682.89	0	0	0	0	
25441418.39	0	0	0	0	
25394474.26	0	0	0	0	
25390438.6	0	0	0	0	
25382780.44	0	0	0	0	
25364353.63	0	0	0	0	
25360320.91	0	0	0	0	
25337864.39	0	0	0	0	
25289787.88	0	0	0	0	
25283875.39	0	0	0	0	
25282003.99	0	0	0	0	
25268958.73	0	0	0	0	
25259471.02	0	0	0	0	
25249256.53	0	0	0	0	
25242890.2	0	0	0	0	
25231989.83	0	0	0	0	
25220456.39	0	0	0	0	
25220044.47	0	0	0	2	Superpathw
25188348.58	0	0	0	0	
25147115.42	0	0	0	0	
25139669.99	0	0	0	0	
25137544.39	0	0	0	0	
25094688.44	0	0	0	0	
25044395.58	0	0	0	0	
25013998.62	0	0	0	0	
24998699.73	0	0	0	0	
24987171.31	0	0	0	0	
24984794.23	0	0	0	0	
24957732.53	0	0	0	0	
24932750.05	0	0	0	0	
24919201.62	0	0	0	0	
24896116.62	0	0	0	0	
24887613.24	0	0	0	0	
24883517.45	0	0	0	0	
24874862.64	0	0	0	0	
24874849.82	0	0	0	0	
24864073.95	0	0	0	0	
24844605.7	0	0	0	0	
24842922.72	0	0	0	0	
24829863.73	0	0	0	0	
24807226.91	0	0	0	0	
24788109.02	0	0	0	0	
24755915.15	0	0	0	0	
24682682.52	0	0	0	0	
24661470.91	0	0	0	0	
24652032.09	0	0	0	0	
24623540.76	0	0	0	9	Superpathw
24622570.97	0	0	0	0	

24607096.45	0	0	0	0	
24578111.5	0	0	0	0	
24573230.43	0	0	0	0	
24566391.62	0	0	0	0	
24463764.76	0	0	0	0	
24455878.91	0	0	0	2	Other
24455698.88	0	0	0	0	
24451294.73	0	0	0	0	
24448258.59	0	0	0	0	
24436093.39	0	0	0	0	
24431130.58	0	0	0	0	
24425487.83	0	0	0	0	
24423222.14	0	0	0	0	
24420707.66	0	0	0	0	
24414447.75	0	0	0	0	
24413213.77	0	0	0	0	
24407120.55	0	0	0	0	
24406703.08	0	0	0	0	
24399703.26	0	0	0	0	
24389441.61	0	0	0	0	
24378655.59	0	0	0	0	
24356680.21	0	0	0	0	
24345482.5	0	0	0	0	
24344311.32	0	0	0	0	
24320409.3	0	0	0	0	
24297397.32	0	0	0	0	
24294800.9	0	0	0	0	
24293279.83	0	0	0	0	
24278059.51	0	0	0	0	
24276197.58	0	0	0	0	
24254572.88	0	0	0	0	
24251090.95	0	0	0	0	
24229035.71	0	0	0	0	
24222154.65	0	0	0	0	
24205629.69	0	0	0	0	
24198097.92	0	0	0	0	
24195174.94	0	0	0	0	
24188603.1	0	0	0	0	
24187497.39	0	0	0	0	
24172995.96	0	0	0	0	
24144795.2	0	0	0	0	
24141382.44	0	0	0	0	
24109736.34	0	0	0	0	
24108069.33	0	0	0	0	
24086844.34	0	0	0	0	
24084158.22	0	0	0	0	
24070179.23	0	0	0	0	
24036935.65	0	0	0	1	Other
24036812.14	0	0	0	0	
24012576.35	0	0	0	0	

23974437.82	0	0	0	0	
23965068.06	0	0	0	0	
23957955.02	0	0	0	0	
23953182.97	0	0	0	1	Other
23903535.38	0	0	0	0	
23875420.26	0	0	0	0	
23874802.54	0	0	0	0	
23861333.95	0	0	0	0	
23854585.14	0	0	0	3	Aspartate su
23840159.66	0	0	0	0	
23806721.78	0	0	0	0	
23799877.56	0	0	0	0	
23798085.16	0	0	0	0	
23796656.39	0	0	0	0	
23791569.29	0	0	0	0	
23774236.75	0	0	0	0	
23757824.79	0	0	0	0	
23711017.61	0	0	0	0	
23706596.25	0	0	0	9	Superpathw
23691704.21	0	0	0	0	
23680257.56	0	0	0	0	
23660902.77	0	0	0	0	
23649300.82	0	0	0	0	
23648484.89	0	0	0	0	
23637597.76	0	0	0	2	Other
23632130.7	0	0	0	0	
23619259.15	0	0	0	0	
23617426.76	0	0	0	0	
23611851.9	0	0	0	0	
23611408.04	0	0	0	0	
23604350.93	0	0	0	0	
23604307.52	0	0	0	0	
23592062.14	0	0	0	0	
23585898.98	0	0	0	0	
23580855.3	0	0	0	0	
23574981	0	0	0	0	
23567537.29	0	0	0	0	
23563835.23	0	0	0	0	
23552368.84	0	0	0	0	
23539083.25	0	0	0	0	
23520946.59	0	0	0	0	
23512237.35	0	0	0	0	
23509568.58	0	0	0	0	
23483628.24	0	0	0	0	
23472825.09	0	0	0	0	
23467899.19	0	0	0	0	
23436615.48	0	0	0	0	
23417730.59	0	0	0	0	
23416724.94	0	0	0	1	Other
23397079.13	0	0	0	0	

23385413.19	0	0	0	0	
23376800.52	0	0	0	0	
23371286.91	0	0	0	0	
23369114.41	0	0	0	0	
23344710.95	0	0	0	0	
23316931.32	0	0	0	0	
23306492.07	0	0	0	0	
23285591.44	0	0	0	0	
23277676.18	0	0	0	0	
23270048.94	0	0	0	0	
23265587.11	0	0	0	0	
23237885.91	0	0	0	0	
23236240.36	0	0	0	0	
23234312.49	0	0	0	0	
23218916.05	0	0	0	0	
23214956.57	0	0	0	0	
23195579.34	0	0	0	1	Superpathw
23191191.2	0	0	0	0	
23181475.44	0	0	0	0	
23157449.29	0	0	0	0	
23145383.89	0	0	0	0	
23140637.66	0	0	0	0	
23131961.77	0	0	0	0	
23128903.89	0	0	0	0	
23118769.27	0	0	0	0	
23113999.07	0	0	0	0	
23097691.46	0	0	0	0	
23079178.17	0	0	0	0	
23069355.84	0	0	0	0	
23065470.07	0	0	0	0	
22992579.43	0	0	0	0	
22977189.52	0	0	0	1	Other
22971608	0	0	0	0	
22961443.74	0	0	0	0	
22936661.2	0	0	0	0	
22870879.43	0	0	0	0	
22854645.54	0	0	0	0	
22831965.14	0	0	0	0	
22808286.04	0	0	0	0	
22794813.12	0	0	0	0	
22792165.81	0	0	0	0	
22766623.35	0	0	0	0	
22725227.01	0	0	0	0	
22712386.08	0	0	0	0	
22677931.58	0	0	0	0	
22677250.48	0	0	0	0	
22608851.77	0	0	0	0	
22584791.64	0	0	0	0	
22581305.73	0	0	0	0	
22548243.91	0	0	0	0	

22545387.66	0	0	0	0	
22535451.2	0	0	0	0	
22529275.26	0	0	0	0	
22512170.78	0	0	0	0	
22461124.27	0	0	0	0	
22439286.02	0	0	0	0	
22433136.91	0	0	0	2	Superpathw
22415068.72	0	0	0	0	
22414182.89	0	0	0	0	
22409933.21	0	0	0	0	
22375590.08	0	0	0	0	
22356650.01	0	0	0	0	
22348491.12	0	0	0	0	
22325148.97	0	0	0	0	
22320668.09	0	0	0	0	
22313857.88	0	0	0	0	
22296976.41	0	0	0	0	
22296497.69	0	0	0	0	
22281595.02	0	0	0	0	
22267510.56	0	0	0	0	
22256376.1	0	0	0	1	Superpathw
22234419.47	0	0	0	0	
22213434.84	0	0	0	0	
22211702.76	0	0	0	0	
22177302.77	0	0	0	0	
22148527.26	0	0	0	0	
22130487.82	0	0	0	0	
22123535.41	0	0	0	5	Superpathw
22118665.76	0	0	0	0	
22117240.7	0	0	0	0	
22115082.03	0	0	0	0	
22080465.59	0	0	0	0	
22062794.84	0	0	0	0	
22052529.77	0	0	0	0	
22047766.96	0	0	0	0	
22022107.86	0	0	0	0	
22019994.84	0	0	0	0	
21996868.6	0	0	0	0	
21965704.56	0	0	0	0	
21938127.5	0	0	0	0	
21886281.72	0	0	0	0	
21867702.31	0	0	0	0	
21854025.56	0	0	0	0	
21839220.24	0	0	0	2	Superpathw
21819000.09	0	0	0	0	
21814756.8	0	0	0	0	
21792290.2	0	0	0	0	
21791015.58	0	0	0	0	
21785208.94	0	0	0	0	
21782616.09	0	0	0	0	

21765709.29	0	0	0	0	
21746938.97	0	0	0	0	
21734764.04	0	0	0	0	
21733592.32	0	0	0	0	
21713667.81	0	0	0	0	
21631374.59	0	0	0	0	
21612112.05	0	0	0	0	
21607655.92	0	0	0	2	Superpathw
21605866.87	0	0	0	0	
21592315.62	0	0	0	0	
21579724.37	0	0	0	0	
21571991.54	0	0	0	0	
21564251.28	0	0	0	0	
21549304.99	0	0	0	0	
21538472.06	0	0	0	1	Superpathw
21533426.32	0	0	0	0	
21522896.73	0	0	0	0	
21501738.76	0	0	0	0	
21494859.65	0	0	0	0	
21490310.1	0	0	0	0	
21484580.39	0	0	0	0	
21461728.15	0	0	0	0	
21460326.06	0	0	0	0	
21455569.31	0	0	0	0	
21455436.27	0	0	0	0	
21447955.61	0	0	0	0	
21404517.81	0	0	0	2	Superpathw
21376402.08	0	0	0	0	
21367625.39	0	0	0	0	
21366419.52	0	0	0	0	
21364368.25	0	0	0	0	
21349639.39	0	0	0	0	
21342713.81	0	0	0	0	
21323432.99	0	0	0	0	
21300361.63	0	0	0	0	
21287679.99	0	0	0	0	
21281337.9	0	0	0	0	
21272283.01	0	0	0	0	
21267598.11	0	0	0	0	
21262034.67	0	0	0	0	
21260977.36	0	0	0	0	
21241425.51	0	0	0	0	
21232757.27	0	0	0	0	
21232025.87	0	0	0	0	
21228477.41	0	0	0	0	
21222512.61	0	0	0	0	
21214459.9	0	0	0	1	Other
21210473.3	0	0	0	0	
21173110.91	0	0	0	0	
21160798.72	0	0	0	0	

21131338.78	0	0	0	0	
21119524.17	0	0	0	0	
21106336.43	0	0	0	0	
21090797.31	0	0	0	0	
21070574.22	0	0	0	0	
21058292.21	0	0	0	0	
21056628.78	0	0	0	0	
21055885.85	0	0	0	0	
21013461.46	0	0	0	0	
21001494.88	0	0	0	0	
21000684.36	0	0	0	0	
20971031	0	0	0	0	
20958077.75	0	0	0	0	
20951526.86	0	0	0	0	
20945031.84	0	0	0	0	
20933877.3	0	0	0	0	
20923769.89	0	0	0	0	
20902001.41	0	0	0	2	Other
20901299.15	0	0	0	0	
20890227.46	0	0	0	0	
20889366.08	0	0	0	2	Superpathw
20885139.15	0	0	0	0	
20856040.61	0	0	0	0	
20821691.06	0	0	0	0	
20820723.06	0	0	0	0	
20817128.17	0	0	0	0	
20805788.95	0	0	0	0	
20754908.3	0	0	0	0	
20710352.73	0	0	0	0	
20705150.59	0	0	0	0	
20703895.51	0	0	0	0	
20690113.16	0	0	0	0	
20680228.38	0	0	0	0	
20646970.33	0	0	0	0	
20639268.28	0	0	0	0	
20624158.6	0	0	0	0	
20620368.91	0	0	0	0	
20618424.03	0	0	0	0	
20610780.26	0	0	0	4	Superpathw
20597330.12	0	0	0	0	
20577305.28	0	0	0	0	
20575073.28	0	0	0	13	Superpathw
20550640	0	0	0	0	
20544968.55	0	0	0	0	
20534095.75	0	0	0	0	
20530943.35	0	0	0	0	
20527157.89	0	0	0	0	
20520610.44	0	0	0	0	
20501657.36	0	0	0	0	
20488372.97	0	0	0	0	

20486278.83	0	0	0	1	Superpathw
20480927.76	0	0	0	0	
20472611.39	0	0	0	0	
20468308.58	0	0	0	0	
20443889.05	0	0	0	0	
20436298.14	0	0	0	0	
20431941.85	0	0	0	1	Other
20409819.16	0	0	0	0	
20406014.96	0	0	0	0	
20394551.43	0	0	0	0	
20371895.16	0	0	0	0	
20354536.64	0	0	0	0	
20350677.71	0	0	0	0	
20349126.22	0	0	0	0	
20346821.45	0	0	0	0	
20317909.56	0	0	0	0	
20316882.58	0	0	0	0	
20299760.14	0	0	0	0	
20298872.74	0	0	0	0	
20278798.45	0	0	0	1	Other
20253397.78	0	0	0	0	
20252474.84	0	0	0	0	
20251091.64	0	0	0	0	
20235244.02	0	0	0	0	
20216805.23	0	0	0	0	
20204347.65	0	0	0	0	
20194016.23	0	0	0	0	
20169607.05	0	0	0	0	
20146475.71	0	0	0	0	
20146136.15	0	0	0	0	
20132306.94	0	0	0	1	Other
20131450.94	0	0	0	0	
20108952.57	0	0	0	0	
20106527.13	0	0	0	1	Superpathw
20076279.58	0	0	0	0	
20065946.11	0	0	0	0	
20065941.92	0	0	0	0	
20063888.31	0	0	0	0	
20062503.28	0	0	0	0	
20014210.89	0	0	0	0	
20005092.23	0	0	0	0	
19977445.14	0	0	0	0	
19971843.27	0	0	0	0	
19962937.31	0	0	0	0	
19941272.29	0	0	0	0	
19932244.18	0	0	0	0	
19914619.78	0	0	0	0	
19905511.51	0	0	0	0	
19865353.71	0	0	0	0	
19863175.23	0	0	0	0	

19849903.34	0	0	0	0	
19832079.54	0	0	0	0	
19792932.6	0	0	0	1	Other
19791933.67	0	0	0	1	Superpathw
19791101.35	0	0	0	0	
19775592.04	0	0	0	0	
19757693.31	0	0	0	0	
19748467.35	0	0	0	0	
19730948.78	0	0	0	0	
19720353.44	0	0	0	0	
19712322.71	0	0	0	0	
19708246.65	0	0	0	0	
19693079.22	0	0	0	0	
19684717.68	0	0	0	0	
19681390.95	0	0	0	0	
19681390.95	0	0	0	0	
19681062.89	0	0	0	0	
19670937.46	0	0	0	0	
19660760.64	0	0	0	0	
19651880.02	0	0	0	0	
19636210.54	0	0	0	0	
19626587.53	0	0	0	0	
19622533.45	0	0	0	0	
19619909.68	0	0	0	0	
19599576.27	0	0	0	0	
19592956.37	0	0	0	0	
19572257.78	0	0	0	0	
19552156.01	0	0	0	0	
19550791.82	0	0	0	0	
19543533.65	0	0	0	0	
19524446.3	0	0	0	0	
19506052.46	0	0	0	1	Other
19459196.64	0	0	0	0	
19452679.36	0	0	0	0	
19451238.55	0	0	0	0	
19430131.62	0	0	0	0	
19414500.42	0	0	0	0	
19401557.23	0	0	0	0	
19400493.72	0	0	0	0	
19385374.21	0	0	0	2	Superpathw
19375981.94	0	0	0	0	
19362979.51	0	0	0	0	
19357128.09	0	0	0	0	
19334479.04	0	0	0	0	
19324859.93	0	0	0	0	
19282779.82	0	0	0	0	
19282779.82	0	0	0	0	
19281146.36	0	0	0	0	
19274654.06	0	0	0	0	
19258879.57	0	0	0	0	

19251873.91	0	0	0	0	
19241925.03	0	0	0	0	
19236726.21	0	0	0	3	Other
19213598.99	0	0	0	0	
19181283.5	0	0	0	0	
19167136.13	0	0	0	0	
19127328.12	0	0	0	0	
19124753	0	0	0	1	Superpathwa
19120630.76	0	0	0	0	
19112149.93	0	0	0	0	
19082844.79	0	0	0	0	
19079427.5	0	0	0	0	
19077306.41	0	0	0	0	
19073823.08	0	0	0	0	
19038220.99	0	0	0	0	
19034138	0	0	0	0	
19034021.42	0	0	0	0	
19023170.84	0	0	0	2	Superpathwa
19021239.35	0	0	0	0	
19017112.23	0	0	0	0	
19007961.47	0	0	0	0	
19007167.18	0	0	0	0	
19004383.17	0	0	0	0	
18978687.05	0	0	0	0	
18973427.95	0	0	0	0	
18969182.96	0	0	0	0	
18963105.07	0	0	0	0	
18934080.87	0	0	0	0	
18933857.61	0	0	0	0	
18884378.4	0	0	0	0	
18881470.83	0	0	0	0	
18875867.98	0	0	0	0	
18850708.56	0	0	0	0	
18816636.98	0	0	0	0	
18814668.08	0	0	0	0	
18813242.23	0	0	0	0	
18810261.78	0	0	0	21	Other
18807491.3	0	0	0	0	
18759185.62	0	0	0	0	
18737875.68	0	0	0	0	
18736289.62	0	0	0	0	
18723646.71	0	0	0	0	
18702466.34	0	0	0	0	
18677850.21	0	0	0	0	
18674938.07	0	0	0	0	
18672413.9	0	0	0	0	
18668558.47	0	0	0	0	
18664077.92	0	0	0	0	
18663893.5	0	0	0	0	
18643179.96	0	0	0	0	

18640707.08	0	0	0	0	
18630928.02	0	0	0	0	
18630020.67	0	0	0	0	
18628505.41	0	0	0	4	Superpathw
18628505.41	0	0	0	0	
18620019.37	0	0	0	0	
18605821.01	0	0	0	0	
18605091.46	0	0	0	0	
18598117.07	0	0	0	0	
18584947.68	0	0	0	0	
18581625.43	0	0	0	0	
18577274.73	0	0	0	1	Other
18571460.84	0	0	0	6	Superpathw
18548264.61	0	0	0	0	
18542088.23	0	0	0	0	
18539442.32	0	0	0	0	
18525446.36	0	0	0	0	
18504511.78	0	0	0	0	
18434437.11	0	0	0	0	
18430927.84	0	0	0	0	
18414531.43	0	0	0	0	
18406079.24	0	0	0	0	
18329634.58	0	0	0	0	
18325968.42	0	0	0	0	
18315748.56	0	0	0	0	
18300536.68	0	0	0	0	
18295318.13	0	0	0	0	
18291461.53	0	0	0	0	
18283618.73	0	0	0	0	
18276195.82	0	0	0	0	
18271434.06	0	0	0	0	
18265433.43	0	0	0	1	Other
18247970.67	0	0	0	0	
18239448.77	0	0	0	0	
18238761.22	0	0	0	0	
18231365.92	0	0	0	0	
18211600	0	0	0	0	
18201344.37	0	0	0	0	
18199532.35	0	0	0	0	
18183282.31	0	0	0	0	
18179980.86	0	0	0	0	
18146778.19	0	0	0	0	
18133840.81	0	0	0	0	
18130266.35	0	0	0	0	
18101360.2	0	0	0	0	
18094983.64	0	0	0	0	
18076971.63	0	0	0	0	
18057367.31	0	0	0	0	
18056950.91	0	0	0	0	
18051115.31	0	0	0	0	

18047465.9	0	0	0	0	
18041740.01	0	0	0	0	
18032081.12	0	0	0	0	
17985797.27	0	0	0	0	
17966716.34	0	0	0	0	
17956920.65	0	0	0	0	
17956898.04	0	0	0	0	
17953936.03	0	0	0	0	
17931931.56	0	0	0	0	
17925911.63	0	0	0	0	
17895305.07	0	0	0	0	
17888869.67	0	0	0	0	
17886095.87	0	0	0	0	
17853886.12	0	0	0	0	
17850149.78	0	0	0	0	
17840294.31	0	0	0	0	
17836961.04	0	0	0	0	
17829894.68	0	0	0	0	
17828571.63	0	0	0	0	
17817826.76	0	0	0	0	
17813596.22	0	0	0	0	
17801538.66	0	0	0	0	
17795575.9	0	0	0	0	
17785068.33	0	0	0	0	
17748894.18	0	0	0	0	
17748393.86	0	0	0	0	
17720702.52	0	0	0	0	
17708707.1	0	0	0	0	
17704909.12	0	0	0	0	
17691610.11	0	0	0	0	
17691149.97	0	0	0	0	
17683143.15	0	0	0	0	
17680249.38	0	0	0	0	
17658882.76	0	0	0	0	
17650573.16	0	0	0	0	
17649912.28	0	0	0	0	
17639910	0	0	0	0	
17639256.02	0	0	0	0	
17638528.86	0	0	0	0	
17623112.66	0	0	0	0	
17604210.78	0	0	0	0	
17576044.17	0	0	0	0	
17568565.65	0	0	0	0	
17568495.93	0	0	0	0	
17529362.31	0	0	0	0	
17505852.12	0	0	0	0	
17480183.49	0	0	0	0	
17466759.69	0	0	0	0	
17465173.34	0	0	0	0	
17456718.66	0	0	0	0	

17448290.78	0	0	0	0	
17445029.03	0	0	0	0	
17396860.69	0	0	0	0	
17394233.47	0	0	0	0	
17394203.47	0	0	0	0	
17346678.94	0	0	0	1	Other
17339463.77	0	0	0	0	
17327602.56	0	0	0	0	
17318720.86	0	0	0	0	
17306737.87	0	0	0	0	
17304136.18	0	0	0	0	
17297946.51	0	0	0	0	
17293745.47	0	0	0	0	
17292316.97	0	0	0	0	
17285283.8	0	0	0	0	
17281221.18	0	0	0	0	
17266347.93	0	0	0	0	
17264759.42	0	0	0	0	
17253004.39	0	0	0	0	
17247361.33	0	0	0	0	
17241589.35	0	0	0	0	
17229046	0	0	0	0	
17217439.61	0	0	0	0	
17197208.48	0	0	0	3	Superpathw
17186685.66	0	0	0	0	
17185503.39	0	0	0	0	
17183596.1	0	0	0	0	
17171999.24	0	0	0	0	
17152913.21	0	0	0	0	
17140078.32	0	0	0	0	
17125223.9	0	0	0	0	
17106865.8	0	0	0	0	
17092726.85	0	0	0	0	
17089294.53	0	0	0	0	
17072968.91	0	0	0	0	
17070273.63	0	0	0	0	
17065884.32	0	0	0	0	
17038289.33	0	0	0	0	
17034156.8	0	0	0	0	
17018869.08	0	0	0	0	
17005209.2	0	0	0	0	
16994913.2	0	0	0	0	
16967907.36	0	0	0	0	
16947702.79	0	0	0	0	
16879157.06	0	0	0	0	
16877835.38	0	0	0	0	
16859115.08	0	0	0	0	
16822307.18	0	0	0	0	
16820803.6	0	0	0	0	
16806931.62	0	0	0	0	

16796353.37	0	0	0	1	Other
16795637.31	0	0	0	0	
16784169.47	0	0	0	0	
16777211.65	0	0	0	0	
16764607.73	0	0	0	0	
16730245.97	0	0	0	0	
16725222.91	0	0	0	0	
16717176.41	0	0	0	0	
16716093.44	0	0	0	0	
16710116.48	0	0	0	12	Superpathw
16699919.23	0	0	0	0	
16699867.01	0	0	0	0	
16696195.98	0	0	0	0	
16693077.17	0	0	0	0	
16675821.76	0	0	0	0	
16670850.43	0	0	0	0	
16659842.34	0	0	0	0	
16633041.84	0	0	0	0	
16619620.2	0	0	0	0	
16603082.97	0	0	0	0	
16602564.3	0	0	0	0	
16602088.75	0	0	0	1	Other
16599025.29	0	0	0	0	
16587354.38	0	0	0	0	
16587185.8	0	0	0	0	
16583842.64	0	0	0	0	
16582887.47	0	0	0	0	
16578771.91	0	0	0	0	
16572054.23	0	0	0	0	
16564496.94	0	0	0	0	
16562382.09	0	0	0	0	
16559285.03	0	0	0	0	
16553117.82	0	0	0	0	
16541192.09	0	0	0	45	Superpathw
16527870.17	0	0	0	0	
16485341.79	0	0	0	1	Superpathw
16476777.71	0	0	0	0	
16456463.69	0	0	0	0	
16445942.16	0	0	0	0	
16443707.74	0	0	0	0	
16406886.4	0	0	0	1	Other
16406518.96	0	0	0	0	
16399917.53	0	0	0	0	
16388002.33	0	0	0	0	
16378807.42	0	0	0	0	
16368432.02	0	0	0	0	
16353062.31	0	0	0	0	
16350229.14	0	0	0	0	
16344912.71	0	0	0	0	
16322018.31	0	0	0	0	

16319676.05	0	0	0	0	
16297559.69	0	0	0	0	
16292010.34	0	0	0	0	
16287019.33	0	0	0	0	
16280666.84	0	0	0	0	
16280571.37	0	0	0	0	
16274104.23	0	0	0	0	
16258644.23	0	0	0	0	
16234272.06	0	0	0	0	
16229640.66	0	0	0	0	
16203053	0	0	0	0	
16202996.84	0	0	0	0	
16188401.79	0	0	0	0	
16185064.37	0	0	0	0	
16149724.93	0	0	0	0	
16122139.8	0	0	0	0	
16117902.72	0	0	0	0	
16109826.34	0	0	0	0	
16098181.61	0	0	0	0	
16097491.76	0	0	0	0	
16088188.69	0	0	0	0	
16085747.82	0	0	0	0	
16068994.48	0	0	0	0	
16065316.18	0	0	0	0	
16060177.64	0	0	0	0	
16047775.79	0	0	0	0	
16042318.36	0	0	0	0	
16003468.21	0	0	0	0	
15964209.31	0	0	0	0	
15961358.41	0	0	0	0	
15957015.41	0	0	0	0	
15942036.1	0	0	0	0	
15918859.21	0	0	0	0	
15916377.11	0	0	0	0	
15899851.04	0	0	0	0	
15886248.57	0	0	0	0	
15876067.92	0	0	0	0	
15874952.04	0	0	0	0	
15867976.35	0	0	0	0	
15863185.44	0	0	0	0	
15858214.64	0	0	0	0	
15857443.91	0	0	0	0	
15847784.67	0	0	0	0	
15829544.75	0	0	0	0	
15828036.55	0	0	0	1	Superpathw
15804777.48	0	0	0	0	
15804577.8	0	0	0	0	
15792007.11	0	0	0	0	
15774714.14	0	0	0	0	
15769034.02	0	0	0	0	

15766275.21	0	0	0	0	
15759677.08	0	0	0	0	
15755150.51	0	0	0	5	Superpathw
15746772.14	0	0	0	0	
15744175.06	0	0	0	0	
15739001.68	0	0	0	0	
15728109.03	0	0	0	0	
15728109.03	0	0	0	0	
15723245.34	0	0	0	0	
15708716.34	0	0	0	0	
15705133.27	0	0	0	1	Other
15703937.32	0	0	0	0	
15699822.61	0	0	0	0	
15691911.62	0	0	0	0	
15681701.48	0	0	0	1	Superpathw
15641896.47	0	0	0	0	
15600041.04	0	0	0	0	
15589908.05	0	0	0	0	
15589110.01	0	0	0	0	
15585547.14	0	0	0	0	
15578268.94	0	0	0	0	
15575395.31	0	0	0	0	
15570109.68	0	0	0	0	
15569651.64	0	0	0	0	
15558235.43	0	0	0	0	
15558167.65	0	0	0	0	
15552529.21	0	0	0	0	
15514754.2	0	0	0	0	
15502511.46	0	0	0	0	
15494833.35	0	0	0	0	
15491260.26	0	0	0	0	
15485259.48	0	0	0	10	Other
15475675.1	0	0	0	0	
15473534.27	0	0	0	0	
15467386.32	0	0	0	0	
15440158.13	0	0	0	0	
15439514.41	0	0	0	0	
15431690.62	0	0	0	0	
15427104.14	0	0	0	0	
15417932.33	0	0	0	0	
15408918.51	0	0	0	0	
15392197.57	0	0	0	13	Superpathw
15382490.82	0	0	0	0	
15382263.39	0	0	0	1	Other
15375334.98	0	0	0	0	
15372131.66	0	0	0	0	
15366597.78	0	0	0	0	
15357672.32	0	0	0	0	
15353700.64	0	0	0	0	
15325712.37	0	0	0	0	

15315838.19	0	0	0	0	
15315793.56	0	0	0	0	
15305109.62	0	0	0	0	
15302135.53	0	0	0	0	
15287615.86	0	0	0	0	
15284242.21	0	0	0	0	
15279219.3	0	0	0	0	
15275204.87	0	0	0	0	
15273515.69	0	0	0	0	
15267773.2	0	0	0	0	
15264212.67	0	0	0	0	
15247718.33	0	0	0	0	
15241957.48	0	0	0	0	
15241827.7	0	0	0	0	
15238862.71	0	0	0	0	
15223264.55	0	0	0	0	
15215125.13	0	0	0	0	
15208861.49	0	0	0	3	Superpathw
15202185.44	0	0	0	0	
15200678.87	0	0	0	0	
15199538.89	0	0	0	0	
15186413.23	0	0	0	0	
15185258.13	0	0	0	0	
15181660.43	0	0	0	0	
15161487.17	0	0	0	0	
15156143.9	0	0	0	0	
15155904.86	0	0	0	0	
15143251.46	0	0	0	0	
15130065.47	0	0	0	0	
15106375.48	0	0	0	0	
15100134.51	0	0	0	0	
15062389.35	0	0	0	0	
15056383.76	0	0	0	0	
15052248.79	0	0	0	0	
15038732.64	0	0	0	0	
15034041.35	0	0	0	0	
15025235.17	0	0	0	0	
15024664.6	0	0	0	0	
15024088.14	0	0	0	0	
15023794.13	0	0	0	0	
15012438.56	0	0	0	0	
15011866.23	0	0	0	0	
14992327.96	0	0	0	0	
14986140.65	0	0	0	0	
14985552.68	0	0	0	0	
14980932.4	0	0	0	0	
14977254.04	0	0	0	0	
14975207.87	0	0	0	0	
14957623.06	0	0	0	0	
14954810.83	0	0	0	2	Superpathw

14951502.09	0	0	0	1	Superpathw
14948153.1	0	0	0	0	
14926350.63	0	0	0	0	
14913325.55	0	0	0	0	
14907809.47	0	0	0	0	
14903342.68	0	0	0	0	
14888010.09	0	0	0	0	
14870633.08	0	0	0	0	
14870129.11	0	0	0	0	
14838342.33	0	0	0	0	
14832308.74	0	0	0	0	
14830026.07	0	0	0	0	
14828505.32	0	0	0	0	
14822886.57	0	0	0	0	
14822005.25	0	0	0	0	
14812817.8	0	0	0	0	
14812060.59	0	0	0	0	
14810694.92	0	0	0	0	
14800752.53	0	0	0	0	
14793458.07	0	0	0	0	
14781564.85	0	0	0	0	
14770370.98	0	0	0	0	
14742672.91	0	0	0	0	
14735906.72	0	0	0	0	
14729648.94	0	0	0	0	
14705673.02	0	0	0	0	
14703896.1	0	0	0	0	
14697042.81	0	0	0	0	
14677865.54	0	0	0	0	
14677810.23	0	0	0	0	
14672292.78	0	0	0	0	
14659569.63	0	0	0	0	
14629577.86	0	0	0	0	
14617806.88	0	0	0	0	
14591932.84	0	0	0	0	
14555788.41	0	0	0	0	
14536132.96	0	0	0	1	Other
14530160.44	0	0	0	0	
14519557.25	0	0	0	0	
14510036.01	0	0	0	0	
14502314.17	0	0	0	0	
14501777.31	0	0	0	0	
14497458.81	0	0	0	0	
14496736.24	0	0	0	0	
14458314.05	0	0	0	14	Superpathw
14444026.42	0	0	0	0	
14437017.6	0	0	0	0	
14420047.07	0	0	0	0	
14405217.47	0	0	0	0	
14404707.03	0	0	0	0	

14388087.15	0	0	0	0	
14365782.24	0	0	0	0	
14365332.17	0	0	0	0	
14359210.51	0	0	0	0	
14346329.09	0	0	0	0	
14341907.16	0	0	0	0	
14334476.25	0	0	0	0	
14306144.03	0	0	0	0	
14304473.03	0	0	0	0	
14302380.1	0	0	0	0	
14300502.24	0	0	0	0	
14300417.14	0	0	0	0	
14276256.97	0	0	0	0	
14274283.64	0	0	0	0	
14264092.79	0	0	0	0	
14250370.08	0	0	0	0	
14228984.57	0	0	0	0	
14228554.35	0	0	0	0	
14221182.39	0	0	0	0	
14215370.05	0	0	0	0	
14198508.94	0	0	0	0	
14195873.09	0	0	0	0	
14189196.17	0	0	0	0	
14184826.82	0	0	0	0	
14180451.69	0	0	0	0	
14173076.24	0	0	0	0	
14152727.67	0	0	0	0	
14148728.54	0	0	0	0	
14141452.72	0	0	0	1	Other
14139379.74	0	0	0	0	
14110184.09	0	0	0	0	
14096865.88	0	0	0	0	
14054408.3	0	0	0	0	
14047611.74	0	0	0	0	
14039730.19	0	0	0	0	
14034458.83	0	0	0	0	
14032112.96	0	0	0	1	Other
14031030.22	0	0	0	0	
14030279.48	0	0	0	0	
14014605.01	0	0	0	2	Anaerobic ar
13995774.07	0	0	0	0	
13992137.06	0	0	0	0	
13986704.95	0	0	0	0	
13981696.76	0	0	0	0	
13977630.91	0	0	0	0	
13974632.34	0	0	0	0	
13974348.64	0	0	0	0	
13934999.6	0	0	0	0	
13918965.61	0	0	0	0	
13912633.1	0	0	0	0	

13896967.98	0	0	0	0	
13884109.6	0	0	0	0	
13875898.35	0	0	0	0	
13861989.09	0	0	0	0	
13853923.02	0	0	0	0	
13853277.22	0	0	0	0	
13852064.17	0	0	0	0	
13844067.22	0	0	0	0	
13831657.09	0	0	0	0	
13822479.86	0	0	0	0	
13819120.01	0	0	0	0	
13810635.24	0	0	0	0	
13804552.59	0	0	0	1	Other
13804552.59	0	0	0	0	
13804520.85	0	0	0	0	
13802746.76	0	0	0	0	
13788200.37	0	0	0	0	
13781443.76	0	0	0	0	
13757997.43	0	0	0	0	
13750729.31	0	0	0	0	
13750154.88	0	0	0	0	
13744442.48	0	0	0	0	
13742053.76	0	0	0	0	
13730815.78	0	0	0	0	
13706530.78	0	0	0	10	Other
13685083.09	0	0	0	0	
13672353.11	0	0	0	0	
13670223.86	0	0	0	0	
13669913.67	0	0	0	0	
13653076.26	0	0	0	0	
13647837.44	0	0	0	0	
13638915.82	0	0	0	0	
13626227.14	0	0	0	0	
13605053.58	0	0	0	0	
13588331.38	0	0	0	0	
13587303.12	0	0	0	0	
13581310.34	0	0	0	0	
13562613.15	0	0	0	0	
13559854.15	0	0	0	0	
13559418.84	0	0	0	0	
13553172.49	0	0	0	0	
13550770.77	0	0	0	0	
13539860.77	0	0	0	0	
13539860.77	0	0	0	0	
13527502.43	0	0	0	0	
13522883.46	0	0	0	0	
13506514.99	0	0	0	0	
13483422.9	0	0	0	0	
13478415.39	0	0	0	0	
13472609.49	0	0	0	0	

13470857.37	0	0	0	0	
13470130.75	0	0	0	0	
13460835.93	0	0	0	0	
13460214.48	0	0	0	0	
13453829.65	0	0	0	0	
13440598.81	0	0	0	0	
13435259.93	0	0	0	0	
13420184.84	0	0	0	0	
13413432.94	0	0	0	0	
13411547.09	0	0	0	0	
13406870.04	0	0	0	0	
13382917.3	0	0	0	0	
13365338.64	0	0	0	7	Other
13360583.08	0	0	0	0	
13356513.21	0	0	0	0	
13328548.06	0	0	0	0	
13319293.55	0	0	0	0	
13316248.91	0	0	0	0	
13309832.01	0	0	0	0	
13301170.77	0	0	0	0	
13286999.04	0	0	0	0	
13285376.99	0	0	0	0	
13281701.02	0	0	0	0	
13278258.23	0	0	0	0	
13267793.14	0	0	0	0	
13258416.33	0	0	0	0	
13247122.05	0	0	0	0	
13218385.32	0	0	0	0	
13215032.25	0	0	0	0	
13212182.87	0	0	0	0	
13212049.82	0	0	0	0	
13202215.56	0	0	0	0	
13196958.94	0	0	0	0	
13183632.51	0	0	0	0	
13172344.94	0	0	0	0	
13161976.63	0	0	0	0	
13157063.84	0	0	0	0	
13151767.65	0	0	0	1	Superpathw
13130617.54	0	0	0	0	
13114705.41	0	0	0	0	
13106492.72	0	0	0	0	
13085591.33	0	0	0	0	
13077330.96	0	0	0	0	
13072565.72	0	0	0	0	
13071370.49	0	0	0	0	
13069112.66	0	0	0	0	
13064305.08	0	0	0	1	Superpathw
13056757.97	0	0	0	0	
13055431.17	0	0	0	0	
13053416.87	0	0	0	0	

13044417.25	0	0	0	0	
13032178.31	0	0	0	0	
13027602.01	0	0	0	0	
13019801.87	0	0	0	13	Superpathw
12983828.76	0	0	0	0	
12972320.92	0	0	0	0	
12933703.85	0	0	0	0	
12931723.75	0	0	0	0	
12922588.75	0	0	0	0	
12883941.55	0	0	0	0	
12862466.11	0	0	0	11	Superpathw
12854951.21	0	0	0	0	
12852665.55	0	0	0	0	
12852179.08	0	0	0	0	
12842818.51	0	0	0	0	
12814563.49	0	0	0	0	
12811026.34	0	0	0	0	
12797159.35	0	0	0	0	
12791118.63	0	0	0	0	
12772282.72	0	0	0	0	
12763768.99	0	0	0	0	
12760147.43	0	0	0	0	
12743182.39	0	0	0	0	
12742954.32	0	0	0	0	
12686848.85	0	0	0	0	
12684224.77	0	0	0	0	
12675804.09	0	0	0	0	
12674917.98	0	0	0	0	
12674324.87	0	0	0	0	
12672250.48	0	0	0	0	
12661196.24	0	0	0	0	
12659952.95	0	0	0	0	
12652602.64	0	0	0	0	
12642996.56	0	0	0	0	
12633575.84	0	0	0	0	
12620620.75	0	0	0	0	
12618695.62	0	0	0	0	
12611415.84	0	0	0	0	
12604684.99	0	0	0	0	
12602035.44	0	0	0	0	
12593692.68	0	0	0	0	
12585908.58	0	0	0	0	
12580740.64	0	0	0	0	
12567415.4	0	0	0	0	
12564411.81	0	0	0	0	
12564130.24	0	0	0	0	
12558025.32	0	0	0	0	
12556629.37	0	0	0	0	
12546248.82	0	0	0	0	
12545093.21	0	0	0	0	

12540842.71	0	0	0	1	Other
12540450.01	0	0	0	0	
12536570.54	0	0	0	0	
12527855.1	0	0	0	0	
12522782.67	0	0	0	0	
12520841.14	0	0	0	0	
12519544.15	0	0	0	0	
12515290.81	0	0	0	0	
12508740.61	0	0	0	0	
12505249.52	0	0	0	4	Other
12492277.01	0	0	0	0	
12486748.48	0	0	0	0	
12476882.07	0	0	0	0	
12455889.71	0	0	0	1	Other
12453197.75	0	0	0	0	
12442406.53	0	0	0	0	
12438133.88	0	0	0	0	
12430378.32	0	0	0	0	
12421494.97	0	0	0	1	Superpathw
12421387.82	0	0	0	0	
12411843.6	0	0	0	0	
12404910.38	0	0	0	0	
12387652.91	0	0	0	0	
12369638.43	0	0	0	0	
12354627.28	0	0	0	0	
12340665.33	0	0	0	0	
12337156.58	0	0	0	0	
12326887.01	0	0	0	0	
12319477.3	0	0	0	0	
12308179.19	0	0	0	0	
12302707.59	0	0	0	0	
12289190.01	0	0	0	0	
12282700.06	0	0	0	0	
12282195.95	0	0	0	1	Other
12274083.08	0	0	0	0	
12260577.75	0	0	0	0	
12257991.29	0	0	0	0	
12255955.85	0	0	0	0	
12241918.7	0	0	0	0	
12238956.64	0	0	0	0	
12227592.44	0	0	0	0	
12223131.57	0	0	0	1	Superpathw
12218669.56	0	0	0	8	Superpathw
12212444.66	0	0	0	0	
12209453.31	0	0	0	0	
12207559.12	0	0	0	0	
12200192.48	0	0	0	0	
12198605.73	0	0	0	1	Superpathw
12180256.91	0	0	0	0	
12180256.91	0	0	0	0	

12176046.62	0	0	0	0	
12175326	0	0	0	0	
12168549.94	0	0	0	0	
12167091.45	0	0	0	1	Superpathw
12160629.11	0	0	0	0	
12159798.36	0	0	0	0	
12147407.66	0	0	0	0	
12147035.33	0	0	0	0	
12145419.63	0	0	0	0	
12134390.02	0	0	0	0	
12121346.13	0	0	0	1	Superpathw
12116893.7	0	0	0	1	Other
12110852.96	0	0	0	0	
12094517.21	0	0	0	0	
12089077.98	0	0	0	0	
12088927.03	0	0	0	0	
12079029.38	0	0	0	0	
12068853.7	0	0	0	0	
12060904.05	0	0	0	0	
12018291.48	0	0	0	0	
12017913.99	0	0	0	0	
12011550.59	0	0	0	0	
11974115.36	0	0	0	0	
11954496.15	0	0	0	0	
11953871.32	0	0	0	0	
11910211.89	0	0	0	0	
11909202.43	0	0	0	0	
11899338.13	0	0	0	0	
11896850.51	0	0	0	0	
11890712	0	0	0	0	
11889526.75	0	0	0	1	Superpathw
11887888.71	0	0	0	0	
11885596.08	0	0	0	0	
11873346.58	0	0	0	0	
11869040.2	0	0	0	0	
11867574.52	0	0	0	0	
11857062.15	0	0	0	1	Superpathw
11842798.69	0	0	0	0	
11842582.67	0	0	0	0	
11842377.58	0	0	0	0	
11838296.92	0	0	0	0	
11831470.29	0	0	0	0	
11830249.51	0	0	0	0	
11820293	0	0	0	0	
11819304.19	0	0	0	0	
11818810.24	0	0	0	0	
11814414.51	0	0	0	11	Superpathw
11811233.44	0	0	0	0	
11799662.94	0	0	0	0	
11783421.36	0	0	0	0	

11779807.19	0	0	0	0	
11775054.33	0	0	0	0	
11770137.69	0	0	0	0	
11759386.93	0	0	0	0	
11759386.93	0	0	0	0	
11748671.73	0	0	0	0	
11739155.03	0	0	0	0	
11737510.65	0	0	0	0	
11733090.11	0	0	0	0	
11732238.1	0	0	0	0	
11716831.99	0	0	0	0	
11716574.98	0	0	0	0	
11713590.17	0	0	0	0	
11701873.99	0	0	0	0	
11696560.01	0	0	0	0	
11669065.31	0	0	0	0	
11668404.86	0	0	0	0	
11664054.31	0	0	0	0	
11661558.22	0	0	0	0	
11649056.13	0	0	0	0	
11620767.52	0	0	0	0	
11617584.9	0	0	0	0	
11617031.8	0	0	0	0	
11614696.89	0	0	0	0	
11608181.33	0	0	0	0	
11576076.24	0	0	0	0	
11558445.67	0	0	0	0	
11535990.71	0	0	0	0	
11525171.9	0	0	0	0	
11518351.68	0	0	0	0	
11514100.64	0	0	0	0	
11494123.87	0	0	0	0	
11483852.43	0	0	0	0	
11472094.13	0	0	0	0	
11460600.34	0	0	0	0	
11456407.27	0	0	0	0	
11443786.98	0	0	0	0	
11435125.93	0	0	0	0	
11431508.72	0	0	0	0	
11427830.61	0	0	0	0	
11422573.26	0	0	0	0	
11419165.12	0	0	0	0	
11416032.18	0	0	0	1	Other
11409423.04	0	0	0	0	
11408234.9	0	0	0	0	
11408029.34	0	0	0	0	
11405593.88	0	0	0	0	
11403541.68	0	0	0	0	
11394917.65	0	0	0	0	
11390137.32	0	0	0	0	

11349319.97	0	0	0	0	
11346713.74	0	0	0	0	
11338466.24	0	0	0	0	
11333630.66	0	0	0	0	
11332945.07	0	0	0	0	
11331114.15	0	0	0	0	
11329933.11	0	0	0	1	Superpathwa
11322487.65	0	0	0	0	
11314332.25	0	0	0	0	
11312595.16	0	0	0	0	
11312225.24	0	0	0	0	
11295323.31	0	0	0	0	
11283101.11	0	0	0	0	
11282968.65	0	0	0	0	
11254119.82	0	0	0	0	
11252877.3	0	0	0	0	
11249949.84	0	0	0	0	
11240392.86	0	0	0	8	Superpathwa
11239128.15	0	0	0	0	
11227313.15	0	0	0	0	
11219195.06	0	0	0	0	
11204684.48	0	0	0	0	
11203554.63	0	0	0	0	
11191521.73	0	0	0	0	
11191164.29	0	0	0	3	Superpathwa
11169160.7	0	0	0	0	
11159728.2	0	0	0	0	
11157028.61	0	0	0	0	
11153217.94	0	0	0	0	
11150535.85	0	0	1	0	
11148148.06	0	0	0	0	
11147661.73	0	0	0	0	
11140736.8	0	0	0	0	
11140643.92	0	0	0	0	
11130105.31	0	0	0	0	
11124609.38	0	0	0	11	Superpathwa
11124609.38	0	0	0	0	
11124408.56	0	0	0	0	
11122507.37	0	0	0	0	
11116381.7	0	0	0	0	
11108592.81	0	0	0	0	
11107834.07	0	0	0	0	
11097392.82	0	0	0	2	Other
11092281.09	0	0	0	0	
11090532.3	0	0	0	0	
11088406.79	0	0	0	0	
11077963	0	0	0	0	
11077410.19	0	0	0	0	
11067039.23	0	0	0	0	
11051608.93	0	0	0	1	Other

11048310.2	0	0	0	0	
11046468.17	0	0	0	0	
11040760.5	0	0	0	0	
11036156.63	0	0	0	0	
11032888.64	0	0	0	0	
11031481.13	0	0	0	0	
11027765.13	0	0	0	0	
11023666.02	0	0	0	0	
11013106.92	0	0	0	0	
11003072.74	0	0	0	0	
11000140.75	0	0	0	0	
10999936.61	0	0	0	0	
10995715.77	0	0	0	0	
10978112.54	0	0	0	0	
10973266.31	0	0	0	0	
10960715.7	0	0	0	0	
10956785.49	0	0	0	0	
10951278.74	0	0	0	0	
10950149.98	0	0	0	0	
10947247.44	0	0	0	1	Superpathw
10944540.94	0	0	0	0	
10932359.06	0	0	0	0	
10927370.49	0	0	0	0	
10923838.19	0	0	0	0	
10915667.12	0	0	0	0	
10909974.04	0	0	0	0	
10891421.26	0	0	0	0	
10889748.54	0	0	0	0	
10886022.15	0	0	0	0	
10869793.75	0	0	0	0	
10867578.2	0	0	0	0	
10861563.26	0	0	0	0	
10858821.9	0	0	0	1	Other
10857350.97	0	0	0	0	
10851195.28	0	0	0	1	Other
10844284.79	0	0	0	0	
10842567.3	0	0	0	0	
10841996.6	0	0	0	0	
10817703.72	0	0	0	1	Other
10812483.27	0	0	0	0	
10809641.41	0	0	0	0	
10795173.86	0	0	0	1	Other
10789401.51	0	0	0	0	
10789149.73	0	0	0	0	
10787379.92	0	0	0	0	
10786303.52	0	0	0	0	
10769618.35	0	0	0	0	
10762785.77	0	0	0	0	
10754532.06	0	0	0	0	
10745490.24	0	0	0	0	

10739090.09	0	0	0	0	
10728196.95	0	0	0	0	
10717893.73	0	0	0	0	
10716912.55	0	0	0	0	
10715045	0	0	0	0	
10713111.29	0	0	0	0	
10708851.72	0	0	0	0	
10699040.18	0	0	0	0	
10687865.7	0	0	0	0	
10687581.28	0	0	0	6	Other
10687271.69	0	0	0	0	
10683470.63	0	0	0	0	
10678998.29	0	0	0	0	
10677214.53	0	0	0	0	
10668730.56	0	0	0	0	
10650318.87	0	0	0	0	
10644721.83	0	0	0	0	
10643501.42	0	0	0	0	
10639785.52	0	0	0	0	
10636090.97	0	0	0	0	
10632874.76	0	0	0	0	
10620926.33	0	0	0	0	
10617031.59	0	0	0	0	
10598950.57	0	0	0	0	
10598431.68	0	0	0	0	
10595005.32	0	0	0	0	
10589921.3	0	0	0	0	
10589345.05	0	0	0	0	
10589308.15	0	0	0	0	
10588141.7	0	0	0	0	
10586634.98	0	0	0	13	Superpathw
10579543.65	0	0	0	0	
10578122.62	0	0	0	0	
10577141.04	0	0	0	0	
10528149.92	0	0	0	2	Superpathw
10519456.83	0	0	0	0	
10486240.46	0	0	0	0	
10481729.99	0	0	0	0	
10481305.74	0	0	0	0	
10478178.55	0	0	0	0	
10455903.19	0	0	0	0	
10427037.55	0	0	0	0	
10424539.37	0	0	0	0	
10422792.83	0	0	0	0	
10420798.03	0	0	0	0	
10419903.15	0	0	0	0	
10419377.39	0	0	0	0	
10416352.18	0	0	0	0	
10415934.04	0	0	0	0	
10405051.3	0	0	0	0	

10399164.97	0	0	0	0	
10398675.94	0	0	0	0	
10394200.43	0	0	0	0	
10384952.48	0	0	0	0	
10378640.23	0	0	0	0	
10374683.96	0	0	0	0	
10374322.16	0	0	0	0	
10373764.59	0	0	0	1	Other
10367270.45	0	0	0	0	
10355795.36	0	0	0	0	
10309103.8	0	0	0	0	
10304231.9	0	0	0	0	
10297501.78	0	0	0	0	
10295818.03	0	0	0	0	
10277615.47	0	0	0	0	
10273233.21	0	0	0	5	Purine nucle
10265596.23	0	0	0	0	
10248904.96	0	0	0	0	
10245077.68	0	0	0	0	
10244829.32	0	0	0	0	
10243279.35	0	0	0	0	
10234445.73	0	0	0	0	
10227107.52	0	0	0	0	
10223550.54	0	0	0	0	
10223051.32	0	0	0	0	
10220161.33	0	0	0	0	
10219321.28	0	0	0	0	
10203515.93	0	0	0	0	
10190983.27	0	0	0	0	
10177070.74	0	0	0	0	
10159222.03	0	0	0	0	
10158573.38	0	0	0	0	
10146656.87	0	0	0	0	
10142245.49	0	0	0	0	
10138182.11	0	0	0	0	
10126323.35	0	0	0	0	
10118978.27	0	0	0	0	
10052513.71	0	0	0	0	
10020261.34	0	0	0	0	
9996727.108	0	0	0	0	
9995002.504	0	0	0	0	
9992729.677	0	0	0	0	
9987764.731	0	0	0	0	
9974459.354	0	0	0	0	
9960108.258	0	0	0	0	
9955383.097	0	0	0	0	
9941825.66	0	0	0	0	
9928898.934	0	0	0	1	Other
9927182.2	0	0	0	0	
9926313.969	0	0	0	0	

9908514.524	0	0	0	0	
9906394.888	0	0	0	0	
9849286.972	0	0	0	0	
9844971.814	0	0	0	0	
9843800.265	0	0	0	0	
9828222.912	0	0	0	0	
9827767.441	0	0	0	0	
9827344.716	0	0	0	0	
9825867.615	0	0	0	0	
9822661.098	0	0	0	0	
9806971.834	0	0	0	0	
9800639.915	0	0	0	0	
9787383.768	0	0	0	0	
9776060.807	0	0	0	0	
9774324.634	0	0	0	0	
9774147.068	0	0	0	0	
9768941.923	0	0	0	0	
9766963.411	0	0	0	0	
9756432.245	0	0	0	0	
9748991.819	0	0	0	0	
9745898.469	0	0	0	0	
9740808.14	0	0	0	0	
9738133.578	0	0	0	0	
9729711.173	0	0	0	0	
9717882.98	0	0	0	0	
9713259.158	0	0	0	0	
9703019.414	0	0	0	0	
9698629.758	0	0	0	0	
9692324.971	0	0	0	0	
9689978.395	0	0	0	2	Superpathw
9683832.732	0	0	0	0	
9681437.815	0	0	0	0	
9672811.521	0	0	0	0	
9658155.763	0	0	0	0	
9654240.64	0	0	0	0	
9645333.292	0	0	0	0	
9613541.556	0	0	0	0	
9613385.443	0	0	0	0	
9606191.731	0	0	0	4	Other
9598695.847	0	0	0	0	
9577981.738	0	0	0	0	
9572078.676	0	0	0	0	
9561212.939	0	0	0	0	
9551058.688	0	0	0	0	
9542406.807	0	0	0	0	
9537951.331	0	0	0	0	
9537249.05	0	0	0	0	
9533889.133	0	0	0	0	
9530922.063	0	0	0	0	
9509512.798	0	0	0	0	

9505847.783	0	0	0	0	
9495494.755	0	0	0	0	
9472705.603	0	0	0	0	
9467541.95	0	0	0	0	
9453489.612	0	0	0	1	Superpathw
9446930.644	0	0	0	0	
9446283.273	0	0	0	0	
9444491.138	0	0	0	0	
9442021.808	0	0	0	0	
9435795.403	0	0	0	0	
9426819.28	0	0	0	0	
9419179.415	0	0	0	0	
9417596.086	0	0	0	0	
9395954.706	0	0	0	0	
9379847.513	0	0	0	0	
9377334.873	0	0	0	0	
9375177.704	0	0	0	0	
9374550.033	0	0	0	0	
9356940.507	0	0	0	0	
9356188.611	0	0	0	0	
9343249.99	0	0	0	0	
9341266.313	0	0	0	0	
9327391.505	0	0	0	0	
9326093.011	0	0	0	0	
9323174.862	0	0	0	0	
9320231.726	0	0	0	0	
9292462.562	0	0	0	0	
9285153.94	0	0	0	2	Superpathw
9276595.595	0	0	0	0	
9276436.956	0	0	0	0	
9272396.891	0	0	0	0	
9265304.646	0	0	0	0	
9264759.908	0	0	0	0	
9264601.917	0	0	0	0	
9256080.977	0	0	0	0	
9255858.024	0	0	0	0	
9251553.808	0	0	0	0	
9235683.069	0	0	0	0	
9219846.709	0	0	0	0	
9215501.14	0	0	0	0	
9214452.823	0	0	0	0	
9202280.172	0	0	0	0	
9197173.026	0	0	0	0	
9179473.613	0	0	0	0	
9178601.582	0	0	0	0	
9171503.108	0	0	0	0	
9161177.379	0	0	0	0	
9160589.872	0	0	0	0	
9152095.062	0	0	0	0	
9137772.026	0	0	0	0	

9132088.8	0	0	0	2	Other
9124880.641	0	0	0	0	
9123108.188	0	0	0	0	
9112774.393	0	0	0	0	
9108810.775	0	0	0	0	
9108058.982	0	0	0	0	
9103037.756	0	0	0	0	
9097735.955	0	0	0	1	Other
9094097.604	0	0	0	0	
9091264.399	0	0	0	0	
9085737.333	0	0	0	0	
9075654.422	0	0	0	0	
9046703.591	0	0	0	0	
9034313.695	0	0	0	0	
9033805.962	0	0	0	0	
9027193.153	0	0	0	0	
9021417.682	0	0	0	0	
9004811.409	0	0	0	1	Superpathw
9001577.28	0	0	0	0	
9000211.588	0	0	0	0	
8981225.778	0	0	0	0	
8973049.7	0	0	0	0	
8965878.748	0	0	0	0	
8957093.201	0	0	0	0	
8955374.066	0	0	0	0	
8952937.179	0	0	0	0	
8946597.234	0	0	0	0	
8941192.833	0	0	0	0	
8939844.178	0	0	0	0	
8926263.784	0	0	0	0	
8917663.353	0	0	0	0	
8905811.039	0	0	0	0	
8900578.794	0	0	0	0	
8899818.219	0	0	0	0	
8894083.908	0	0	0	0	
8890908.364	0	0	0	0	
8880616.572	0	0	0	0	
8869413.609	0	0	0	11	Superpathw
8864051.402	0	0	0	0	
8863914.033	0	0	0	0	
8856237.384	0	0	0	0	
8851594.59	0	0	0	0	
8838868.526	0	0	0	0	
8836411.842	0	0	0	0	
8830725.187	0	0	0	0	
8828093.84	0	0	0	0	
8825898.132	0	0	0	0	
8817132.644	0	0	0	0	
8796911.516	0	0	0	0	
8791859.115	0	0	0	0	

8786409.697	0	0	0	0	
8780827.368	0	0	0	0	
8736149.24	0	0	0	0	
8727497.705	0	0	0	0	
8726605.599	0	0	0	0	
8725629.344	0	0	0	0	
8721766.45	0	0	0	0	
8719020.896	0	0	0	0	
8715480.337	0	0	0	0	
8713832.764	0	0	0	0	
8700547.835	0	0	0	0	
8697156.422	0	0	0	0	
8696226.959	0	0	0	0	
8693857.217	0	0	0	0	
8669829.105	0	0	0	0	
8662534.117	0	0	0	0	
8633442.046	0	0	0	0	
8616078.284	0	0	0	0	
8611144.566	0	0	0	0	
8608182.29	0	0	0	0	
8607221.467	0	0	0	0	
8601799.91	0	0	0	0	
8601536.914	0	0	0	0	
8598529.65	0	0	0	0	
8588924.663	0	0	0	0	
8564890.679	0	0	0	0	
8562526.461	0	0	0	0	
8557433.345	0	0	0	0	
8557377.177	0	0	0	0	
8554623.889	0	0	0	0	
8539725.396	0	0	0	0	
8538674.805	0	0	0	0	
8536556.968	0	0	0	0	
8533992.16	0	0	0	0	
8524439.664	0	0	0	0	
8522701.977	0	0	0	0	
8502896.284	0	0	0	0	
8498390.881	0	0	0	0	
8495285.211	0	0	0	0	
8488974.829	0	0	0	0	
8483152.84	0	0	0	0	
8480431.373	0	0	0	0	
8470614.935	0	0	0	0	
8467725.33	0	0	0	0	
8466032.896	0	0	0	0	
8444679.365	0	0	0	0	
8440763.95	0	0	0	0	
8435394.113	0	0	0	0	
8434933.086	0	0	0	0	
8416796.082	0	0	0	0	

8407313.827	0	0	0	0	
8393407.617	0	0	0	3	Other
8385391.875	0	0	0	0	
8381597.019	0	0	0	0	
8380242.256	0	0	0	0	
8347122.674	0	0	0	0	
8337069.351	0	0	0	0	
8317351.166	0	0	0	0	
8305227.689	0	0	0	0	
8287204.034	0	0	0	0	
8269713.46	0	0	0	0	
8269405.535	0	0	0	0	
8266482.778	0	0	0	0	
8262479.399	0	0	0	0	
8260658.513	0	0	0	0	
8242980.272	0	0	0	0	
8237299.253	0	0	0	0	
8235347.848	0	0	0	0	
8233567.5	0	0	0	0	
8229932.434	0	0	0	0	
8229309.004	0	0	0	0	
8217382.841	0	0	0	0	
8210364.813	0	0	0	0	
8202208.208	0	0	2	0	
8190051.303	0	0	0	0	
8184259.307	0	0	0	0	
8176910.036	0	0	0	0	
8175667.921	0	0	0	0	
8171941.915	0	0	0	0	
8167600.083	0	0	0	0	
8164655.689	0	0	0	0	
8162138.855	0	0	0	0	
8159512.787	0	0	0	0	
8154030.461	0	0	0	0	
8151327.828	0	0	0	0	
8149043.534	0	0	0	0	
8137926.105	0	0	0	0	
8121373.243	0	0	0	0	
8117354.564	0	0	0	0	
8102721.099	0	0	0	1	Other
8099239.827	0	0	0	0	
8089620.076	0	0	0	1	Superpathw
8087425.636	0	0	0	0	
8076128.209	0	0	0	0	
8072472.602	0	0	0	0	
8071091.322	0	0	0	5	Superpathw
8064099.218	0	0	0	0	
8063896.696	0	0	0	0	
8061916.066	0	0	0	0	
8060557.345	0	0	0	0	

8039371.198	0	0	0	0	
8025869.457	0	0	0	0	
8025298.552	0	0	0	0	
8021788.595	0	0	0	0	
8019276.28	0	0	0	0	
8018322.888	0	0	0	0	
8017213.443	0	0	0	0	
8016104.42	0	0	0	0	
8015151.095	0	0	0	0	
8008090.542	0	0	0	0	
8006225.107	0	0	0	0	
8002620.443	0	0	0	0	
8000936.16	0	0	0	0	
7995069.904	0	0	0	0	
7982013.522	0	0	0	0	
7978314.469	0	0	0	0	
7977684.819	0	0	0	0	
7971778.41	0	0	0	0	
7966115.385	0	0	0	0	
7962019.59	0	0	0	0	
7948084.924	0	0	0	0	
7927805.387	0	0	0	0	
7915957.293	0	0	0	0	
7911612.583	0	0	0	0	
7910739.427	0	0	0	0	
7910406.659	0	0	0	0	
7909245.9	0	0	0	0	
7908600.098	0	0	0	0	
7903921.562	0	0	0	0	
7902583.972	0	0	0	0	
7898262.217	0	0	0	0	
7893084.243	0	0	0	0	
7890272.559	0	0	0	0	
7883727.694	0	0	0	0	
7876517.572	0	0	0	10	Other
7873335.432	0	0	0	0	
7868524.015	0	0	0	0	
7863491.884	0	0	0	0	
7858739.62	0	0	0	0	
7857645.735	0	0	0	0	
7847214.075	0	0	0	0	
7841796.227	0	0	0	0	
7834209.261	0	0	0	0	
7832400.975	0	0	0	0	
7820468.742	0	0	0	0	
7808426.984	0	0	0	0	
7803438.146	0	0	0	0	
7800047.403	0	0	0	0	
7797503.161	0	0	0	0	
7789747.126	0	0	0	0	

7776003.232	0	0	0	0	
7775012.518	0	0	0	0	
7769242.922	0	0	0	0	
7769188.769	0	0	0	0	
7764375.659	0	0	0	0	
7763788.069	0	0	0	0	
7762989.881	0	0	0	0	
7760188.144	0	0	0	0	
7742449.059	0	0	0	1	Other
7742028.833	0	0	0	0	
7740854.395	0	0	0	0	
7737912.336	0	0	0	0	
7737832.875	0	0	0	0	
7724957.662	0	0	0	0	
7717824.438	0	0	0	0	
7717267.013	0	0	0	0	
7714185.123	0	0	0	0	
7712666.333	0	0	0	0	
7712186.328	0	0	0	0	
7710551.626	0	0	0	0	
7708775.886	0	0	0	0	
7708343.085	0	0	0	0	
7706973.433	0	0	0	0	
7697008.141	0	0	0	0	
7690804.292	0	0	0	0	
7690706.145	0	0	0	0	
7690691.727	0	0	0	0	
7688463.06	0	0	0	0	
7686836.476	0	0	0	0	
7676199.086	0	0	0	0	
7674866.477	0	0	0	0	
7671249.674	0	0	0	0	
7658952.308	0	0	0	0	
7655450.426	0	0	0	0	
7639696.04	0	0	0	0	
7628080.55	0	0	0	0	
7622468.743	0	0	0	0	
7620033.432	0	0	0	1	Superpathw
7610166.681	0	0	0	0	
7607949.22	0	0	0	0	
7595687.348	0	0	0	0	
7591851.895	0	0	0	0	
7590669.623	0	0	0	0	
7590490.606	0	0	0	0	
7571145.799	0	0	0	0	
7568579.08	0	0	0	0	
7566200.616	0	0	0	0	
7565521.739	0	0	0	0	
7543277.338	0	0	0	0	
7538606.074	0	0	0	0	

7520062.752	0	0	0	0	
7513242.926	0	0	0	0	
7513228.303	0	0	0	0	
7506595.71	0	0	0	0	
7498273.055	0	0	0	0	
7496925.573	0	0	0	0	
7466120.276	0	0	0	0	
7462041.644	0	0	0	0	
7458551.597	0	0	0	0	
7448753.726	0	0	0	0	
7447223.698	0	0	0	0	
7443160.425	0	0	0	0	
7432651.756	0	0	0	0	
7422327.071	0	0	0	0	
7407914.796	0	0	0	0	
7387932.963	0	0	0	0	
7379024.68	0	0	0	0	
7375828.863	0	0	0	11	Other
7369569.331	0	0	0	0	
7364865.542	0	0	0	0	
7357872.442	0	0	0	1	Other
7357103.664	0	0	0	0	
7347708.721	0	0	0	0	
7346077.624	0	0	0	0	
7332099.465	0	0	0	0	
7326012.016	0	0	0	0	
7321224.902	0	0	0	0	
7316403.293	0	0	0	0	
7305735.248	0	0	0	0	
7299323.014	0	0	0	0	
7280914.123	0	0	0	1	Superpathw
7275284.437	0	0	0	0	
7270421.349	0	0	0	0	
7270121.252	0	0	0	2	Superpathw
7263081.459	0	0	0	0	
7250203.836	0	0	0	0	
7247822.752	0	0	0	0	
7246749.919	0	0	0	0	
7241534.463	0	0	0	0	
7229388.106	0	0	0	1	Other
7195566.828	0	0	0	1	Other
7187390.156	0	0	0	0	
7175979.77	0	0	0	0	
7174593.52	0	0	0	0	
7166796.522	0	0	0	0	
7161906.035	0	0	0	0	
7151249.841	0	0	0	0	
7150050.63	0	0	0	0	
7149118.825	0	0	0	0	
7144894.947	0	0	0	0	

7144894.947	0	0	0	0	
7143614.745	0	0	0	0	
7140355.47	0	0	0	0	
7132063.693	0	0	0	0	
7128207.779	0	0	0	0	
7124664.601	0	0	0	0	
7118755.23	0	0	0	0	
7104692.464	0	0	0	0	
7095072.14	0	0	0	0	
7089628.749	0	0	0	0	
7088692.575	0	0	0	0	
7083748.774	0	0	0	0	
7074129.469	0	0	0	0	
7069811.345	0	0	0	0	
7069467.547	0	0	0	0	
7069417.867	0	0	0	0	
7063914.175	0	0	0	0	
7062051.531	0	0	0	0	
7056503.058	0	0	0	0	
7048744.418	0	0	0	0	
7042657.155	0	0	0	0	
7040660.934	0	0	0	0	
7035744.819	0	0	0	1	Other
7035614.189	0	0	0	0	
7034483.427	0	0	0	0	
7030284.477	0	0	0	0	
7021399.733	0	0	0	0	
7021274.471	0	0	0	0	
7020889.169	0	0	0	0	
7013303.75	0	0	0	0	
7005915.938	0	0	0	0	
7004192.154	0	0	0	0	
6997960.96	0	0	0	0	
6985448.575	0	0	0	0	
6982635.746	0	0	0	0	
6981690.227	0	0	0	0	
6971025.313	0	0	0	0	
6964065.432	0	0	0	0	
6960808.954	0	0	0	0	
6947130.177	0	0	0	0	
6946227.354	0	0	0	0	
6945163.877	0	0	0	0	
6943497.572	0	0	0	0	
6936592.613	0	0	0	0	
6934489.716	0	0	0	0	
6932247.395	0	0	0	0	
6925087.346	0	0	0	0	
6914885.932	0	0	0	0	
6912486.431	0	0	0	1	Superpathw
6912168.122	0	0	0	0	

6912120.832	0	0	0	0	
6909314.936	0	0	0	1	Superpathw
6904960.068	0	0	0	0	
6880310.864	0	0	0	0	
6877570.039	0	0	0	0	
6874012.543	0	0	0	0	
6873905.207	0	0	0	0	
6871254.211	0	0	0	0	
6867209.302	0	0	0	0	
6855053.052	0	0	0	0	
6852149.748	0	0	0	0	
6843236.116	0	0	0	0	
6839954.219	0	0	0	0	
6832902.277	0	0	0	0	
6828746.848	0	0	0	0	
6819733.541	0	0	0	0	
6809826.074	0	0	0	0	
6801726.778	0	0	0	0	
6785559.87	0	0	0	0	
6784341.23	0	0	0	0	
6782764.043	0	0	0	0	
6771689.125	0	0	0	0	
6769693.688	0	0	0	0	
6765936.895	0	0	0	0	
6764393.129	0	0	0	0	
6761307.076	0	0	0	0	
6750861.963	0	0	0	0	
6733039.966	0	0	0	0	
6727690.452	0	0	0	0	
6726504.37	0	0	0	0	
6722423.731	0	0	0	0	
6717706.223	0	0	0	2	Superpathw
6702907.584	0	0	0	0	
6693516.194	0	0	0	0	
6691447.772	0	0	0	0	
6678522.262	0	0	0	0	
6678215.208	0	0	0	0	
6669553.583	0	0	0	0	
6657386.909	0	0	0	0	
6656872.478	0	0	0	0	
6647124.242	0	0	0	0	
6645140.249	0	0	0	0	
6639057.231	0	0	0	0	
6627406.849	0	0	0	0	
6625312.622	0	0	0	0	
6621995.329	0	0	0	0	
6621512.022	0	0	0	0	
6620694.576	0	0	0	0	
6617891.977	0	0	0	0	
6609243.217	0	0	0	0	

6606968.34	0	0	0	0	
6601349.345	0	0	0	0	
6590144.77	0	0	0	0	
6584691.738	0	0	0	0	
6575164.637	0	0	0	0	
6561271.346	0	0	0	0	
6553078.096	0	0	0	0	
6549471.914	0	0	0	0	
6538133.366	0	0	0	0	
6533208.705	0	0	0	0	
6526556.762	0	0	0	0	
6523853.169	0	0	0	0	
6504506.588	0	0	0	0	
6497609.578	0	0	0	0	
6497480.736	0	0	0	0	
6489551.921	0	0	0	0	
6478815.951	0	0	0	0	
6478315.236	0	0	0	0	
6447062.725	0	0	0	0	
6446227.027	0	0	0	0	
6445156.005	0	0	0	0	
6436673.071	0	0	0	0	
6429036.751	0	0	0	0	
6428745.515	0	0	0	0	
6424629.052	0	0	0	0	
6417473.271	0	0	0	0	
6402241.381	0	0	0	0	
6401853.762	0	0	0	0	
6398311.739	0	0	0	0	
6392720.644	0	0	0	0	
6392457.905	0	0	0	0	
6391607.057	0	0	0	0	
6378221.532	0	0	0	0	
6371059.309	0	0	0	0	
6361162.202	0	0	0	0	
6345991.398	0	0	0	0	
6345389.953	0	0	0	3	Other
6338930.299	0	0	0	0	
6315616.614	0	0	0	0	
6310052.526	0	0	0	0	
6293784.362	0	0	0	0	
6291591.331	0	0	0	0	
6288830.398	0	0	0	0	
6287898.43	0	0	0	0	
6272600.431	0	0	0	0	
6272296.721	0	0	0	0	
6271251.786	0	0	0	0	
6263148.122	0	0	0	0	
6226518.217	0	0	0	0	
6220940.348	0	0	0	0	

6220451.892	0	0	0	0	
6217711.742	0	0	0	0	
6206195.247	0	0	0	0	
6203637.505	0	0	0	0	
6203361.968	0	0	0	0	
6188639.891	0	0	0	0	
6185933.342	0	0	0	0	
6171683.896	0	0	0	0	
6170163.133	0	0	0	0	
6169696.624	0	0	0	0	
6160268.781	0	0	0	0	
6154001.561	0	0	0	0	
6148478.69	0	0	0	0	
6142430.193	0	0	0	0	
6128095.688	0	0	0	0	
6118151.083	0	0	0	0	
6109769.001	0	0	0	0	
6109360.206	0	0	0	0	
6109162.237	0	0	0	0	
6101993.304	0	0	0	0	
6095784.532	0	0	0	0	
6094552.986	0	0	0	0	
6086768.894	0	0	0	0	
6083503.061	0	0	0	0	
6069434.714	0	0	0	1	Superpathw
6068366.63	0	0	0	0	
6064180.991	0	0	0	0	
6034864.778	0	0	0	0	
6027047.311	0	0	0	0	
6019064.337	0	0	0	0	
6013516.452	0	0	0	0	
6002754.598	0	0	0	0	
5999845.579	0	0	0	0	
5994347.117	0	0	0	0	
5991799.978	0	0	0	0	
5983571.385	0	0	0	0	
5977280.851	0	0	0	0	
5975331.916	0	0	0	0	
5969230.632	0	0	0	0	
5966094.225	0	0	0	0	
5957353.196	0	0	0	0	
5955741.12	0	0	0	0	
5954075.115	0	0	0	0	
5952613.566	0	0	0	0	
5952190.822	0	0	0	0	
5942411.649	0	0	0	0	
5937049.012	0	0	0	1	Other
5926673.905	0	0	0	1	Superpathw
5919866.114	0	0	0	0	
5910881.525	0	0	0	0	

5910645.809	0	0	0	0	
5909773.869	0	0	0	0	
5895276.376	0	0	0	0	
5893512.959	0	0	0	3	Other
5893187.062	0	0	0	0	
5876585.894	0	0	0	0	
5873434.323	0	0	0	0	
5870092.354	0	0	0	0	
5868291.09	0	0	0	0	
5868008.928	0	0	0	3	Other
5867983.993	0	0	0	0	
5867873.811	0	0	0	0	
5851640.299	0	0	0	0	
5848927.54	0	0	0	0	
5840694.445	0	0	0	0	
5839945.161	0	0	0	0	
5839727.539	0	0	0	0	
5837110.649	0	0	0	0	
5832221.287	0	0	0	0	
5828617.154	0	0	0	0	
5826989.238	0	0	0	0	
5826845.522	0	0	0	1	Superpathw
5825293.863	0	0	0	0	
5821650.95	0	0	0	0	
5817471.765	0	0	0	0	
5815883.267	0	0	0	1	Superpathw
5815631.297	0	0	0	0	
5783953.342	0	0	0	0	
5783113.837	0	0	0	0	
5782524.977	0	0	0	0	
5782425.799	0	0	0	0	
5772082.164	0	0	0	0	
5765589.462	0	0	0	0	
5755648.601	0	0	0	0	
5750568.564	0	0	0	0	
5745820.199	0	0	0	0	
5745260.035	0	0	0	0	
5744714.351	0	0	0	0	
5742990.535	0	0	0	0	
5733987.863	0	0	0	0	
5720232.248	0	0	0	0	
5713064.914	0	0	0	0	
5712854.584	0	0	0	0	
5712689.24	0	0	0	0	
5707242.483	0	0	0	0	
5698511.748	0	0	0	0	
5694742.887	0	0	0	0	
5693717.189	0	0	0	0	
5690331.461	0	0	0	0	
5688782.461	0	0	0	0	

5680411.106	0	0	0	0	
5671889.366	0	0	0	0	
5668888.846	0	0	0	0	
5665279.16	0	0	0	0	
5661352.458	0	0	0	1	Superpathw
5652701.119	0	0	0	0	
5650564.624	0	0	0	0	
5649773.429	0	0	0	0	
5642156.456	0	0	0	0	
5639792.322	0	0	0	0	
5638092.109	0	0	0	0	
5637792.616	0	0	0	0	
5629007.958	0	0	0	0	
5618950.343	0	0	0	0	
5616890.183	0	0	0	0	
5613619.047	0	0	0	0	
5612405.49	0	0	0	0	
5603107.735	0	0	0	0	
5597559.146	0	0	0	0	
5589871.526	0	0	0	0	
5583227.276	0	0	0	0	
5580577.162	0	0	0	0	
5573455.207	0	0	0	0	
5570342.488	0	0	0	2	Other
5553572.779	0	0	0	0	
5549591.563	0	0	0	0	
5547287.354	0	0	0	0	
5525776.839	0	0	0	1	Other
5520266.157	0	0	0	0	
5516951.267	0	0	0	0	
5501573.961	0	0	0	0	
5501485.967	0	0	0	0	
5491391.039	0	0	0	0	
5489043.023	0	0	0	0	
5480020.183	0	0	0	0	
5476371.914	0	0	0	0	
5465298.494	0	0	0	0	
5465252.176	0	0	0	0	
5459584.786	0	0	0	0	
5456658.31	0	0	0	0	
5439821.806	0	0	0	0	
5432062.365	0	0	0	8	Superpathw
5430061.658	0	0	0	0	
5429621.953	0	0	0	0	
5417997.263	0	0	0	3	Other
5406593.911	0	0	0	0	
5406593.911	0	0	0	0	
5389724.878	0	0	0	0	
5383853.495	0	0	0	0	
5378028.428	0	0	0	0	

5373634.821	0	0	0	0	
5353086.763	0	0	0	0	
5352266.321	0	0	0	0	
5346602.237	0	0	0	0	
5338087.061	0	0	0	0	
5330163.248	0	0	0	0	
5327114.363	0	0	0	0	
5327114.363	0	0	0	0	
5326313.52	0	0	0	0	
5319881.915	0	0	0	2	Superpathw
5302048.908	0	0	0	0	
5301982.261	0	0	0	0	
5299611.229	0	0	0	0	
5298987.936	0	0	0	0	
5295309.95	0	0	0	0	
5293034.47	0	0	0	0	
5276459.719	0	0	0	0	
5273569.452	0	0	0	0	
5265885.039	0	0	0	0	
5255701.481	0	0	0	0	
5252103.252	0	0	0	0	
5244829.635	0	0	0	0	
5231270.4	0	0	0	0	
5220809.776	0	0	0	12	Superpathw
5218134.196	0	0	0	0	
5212829.599	0	0	0	0	
5212688.738	0	0	0	0	
5211107.268	0	0	0	0	
5209608.167	0	0	0	0	
5208041.445	0	0	0	0	
5198137.901	0	0	0	0	
5195347.395	0	0	0	0	
5190770.844	0	0	0	0	
5190696.484	0	0	0	0	
5185774.462	0	0	0	0	
5160897.84	0	0	0	0	
5137485.239	0	0	0	0	
5137134.104	0	0	0	0	
5133256.504	0	0	0	0	
5128659.366	0	0	0	0	
5108982.09	0	0	0	0	
5107660.109	0	0	0	0	
5101985.9	0	0	0	0	
5100580.312	0	0	0	0	
5100033.33	0	0	0	0	
5090935.734	0	0	0	0	
5073687.528	0	0	0	0	
5065794.955	0	0	0	0	
5062662.092	0	0	0	0	
5060131.285	0	0	0	0	

5057512.005	0	0	0	0	
5056143.64	0	0	0	0	
5055208.287	0	0	0	0	
5053484.098	0	0	0	0	
5053007.035	0	0	0	0	
5052188.257	0	0	0	0	
5045852.988	0	0	0	0	
5038223.25	0	0	0	0	
5028229.645	0	0	0	0	
5026333.872	0	0	0	0	
5022460.324	0	0	0	0	
5018854.967	0	0	0	0	
5009977.248	0	0	0	0	
5007921.47	0	0	0	0	
5007842.107	0	0	0	0	
5006502.208	0	0	0	0	
4994617.859	0	0	0	0	
4970140.752	0	0	0	0	
4959128.502	0	0	0	0	
4940858.544	0	0	0	0	
4940725.661	0	0	0	0	
4908919.314	0	0	0	1	Superpathw
4903983.241	0	0	0	0	
4899532.924	0	0	0	0	
4880019.566	0	0	0	0	
4875345.336	0	0	0	0	
4864571.061	0	0	0	0	
4862722.336	0	0	0	0	
4855011.848	0	0	0	0	
4854637.915	0	0	0	0	
4841662.597	0	0	0	0	
4837022.042	0	0	0	0	
4821381.743	0	0	0	0	
4820852.63	0	0	0	0	
4812102.684	0	0	0	0	
4796729.653	0	0	0	0	
4768254.182	0	0	0	0	
4761500.361	0	0	0	0	
4754859.448	0	0	0	0	
4749569.364	0	0	0	0	
4736431.693	0	0	0	0	
4729453.39	0	0	0	0	
4724809.089	0	0	0	0	
4717379.568	0	0	0	0	
4710509.476	0	0	0	0	
4702342.738	0	0	0	0	
4686832.351	0	0	0	0	
4686642.47	0	0	0	0	
4676786.777	0	0	0	0	
4668279.157	0	0	0	2	Superpathw

4664286.729	0	0	0	0	
4650206.174	0	0	0	0	
4649509.669	0	0	0	0	
4646205.512	0	0	0	1	Other
4639242.395	0	0	0	0	
4632134.614	0	0	0	0	
4623252.315	0	0	0	0	
4623209.69	0	0	0	0	
4622537.439	0	0	0	0	
4604472.98	0	0	0	0	
4591996.798	0	0	0	0	
4585285.846	0	0	0	0	
4585241.116	0	0	0	0	
4585005.054	0	0	0	0	
4574498.102	0	0	0	0	
4563010.867	0	0	0	0	
4557627.592	0	0	0	1	Other
4550971.557	0	0	0	0	
4544361.344	0	0	0	0	
4538720.488	0	0	0	0	
4531888.567	0	0	0	0	
4531646.562	0	0	0	0	
4531349.583	0	0	0	0	
4522551.99	0	0	0	0	
4521220.705	0	0	0	0	
4519260.613	0	0	0	0	
4514592.57	0	0	0	0	
4514578.525	0	0	0	0	
4500773.731	0	0	0	0	
4497630.568	0	0	0	1	Other
4494013.862	0	0	0	0	
4491008.035	0	0	0	0	
4476216.157	0	0	0	0	
4474246.038	0	0	0	0	
4470512.589	0	0	0	0	
4468859.395	0	0	0	0	
4467275.409	0	0	0	0	
4465100.351	0	0	0	0	
4453132.486	0	0	0	0	
4448161.256	0	0	0	0	
4447439.578	0	0	0	0	
4444072.482	0	0	0	0	
4440125.298	0	0	0	0	
4434079.541	0	0	0	0	
4421490.275	0	0	0	0	
4420085.644	0	0	0	0	
4412383.777	0	0	0	0	
4411900.086	0	0	0	0	
4390188.397	0	0	0	0	
4382790.799	0	0	0	0	

4369853.115	0	0	0	0	
4369450.723	0	0	0	0	
4361564.641	0	0	0	0	
4357408.835	0	0	0	0	
4356067.688	0	0	0	0	
4350832.551	0	0	0	0	
4350610.874	0	0	0	0	
4346234.025	0	0	0	0	
4334577.12	0	0	0	0	
4329578.99	0	0	0	0	
4324024.376	0	0	0	0	
4322950.442	0	0	0	0	
4314999.645	0	0	0	0	
4314460.926	0	0	0	0	
4308828.138	0	0	0	0	
4306821.151	0	0	0	0	
4303110.375	0	0	0	0	
4300751.816	0	0	0	0	
4284475.238	0	0	0	0	
4283213.858	0	0	0	0	
4279769.819	0	0	0	0	
4269509.911	0	0	0	0	
4263965.967	0	0	0	0	
4257175.858	0	0	0	0	
4250372.127	0	0	0	0	
4243385.524	0	0	0	0	
4242377.591	0	0	0	0	
4237694.46	0	0	0	0	
4236972.725	0	0	0	0	
4234380.928	0	0	0	0	
4228665.411	0	0	0	0	
4221128.442	0	0	0	0	
4220595.461	0	0	0	0	
4219817.967	0	0	0	1	Other
4219235.998	0	0	0	0	
4214920.263	0	0	0	0	
4213741.864	0	0	0	0	
4203521.384	0	0	0	0	
4203365.426	0	0	0	0	
4183506.055	0	0	0	0	
4182832.316	0	0	0	0	
4182640.07	0	0	0	0	
4175936.26	0	0	0	0	
4175781.329	0	0	0	0	
4174726.192	0	0	0	0	
4171820.96	0	0	0	0	
4158792.014	0	0	0	0	
4158200.3	0	0	0	0	
4148258.185	0	0	0	0	
4146913.382	0	0	0	0	

4144014.66	0	0	0	0	
4139221.692	0	0	0	0	
4118350.836	0	0	0	0	
4109299.321	0	0	0	0	
4107540.088	0	0	0	0	
4101843.52	0	0	0	0	
4096809.328	0	0	0	0	
4095290.912	0	0	0	0	
4090660.552	0	0	0	0	
4085816.025	0	0	0	0	
4078834.477	0	0	0	0	
4077020.443	0	0	0	0	
4066843.578	0	0	0	0	
4062298.149	0	0	0	0	
4060447.378	0	0	0	0	
4049862.239	0	0	0	0	
4046840.337	0	0	0	0	
4045963.846	0	0	0	0	
4032573.235	0	0	0	0	
4027193.724	0	0	0	0	
4026674.294	0	0	0	0	
4020254.675	0	0	0	0	
4002393.604	0	0	0	0	
3998802.837	0	0	0	0	
3992354.801	0	0	0	0	
3981471.291	0	0	0	0	
3980281.146	0	0	0	0	
3974136.626	0	0	0	0	
3969591.842	0	0	0	0	
3966168.384	0	0	0	0	
3965211.435	0	0	0	0	
3955435.401	0	0	0	0	
3953052.456	0	0	0	0	
3946714.615	0	0	0	0	
3946023.125	0	0	0	0	
3940055.112	0	0	0	0	
3933941.371	0	0	0	0	
3920269.895	0	0	0	0	
3908954.114	0	0	0	0	
3902309.693	0	0	0	0	
3901601.685	0	0	0	0	
3900159.135	0	0	0	2	Other
3899291.722	0	0	0	0	
3899035.004	0	0	0	0	
3895354.854	0	0	0	0	
3882398.779	0	0	0	0	
3877821.329	0	0	0	0	
3876601.613	0	0	0	0	
3863042.494	0	0	0	0	
3862060.025	0	0	0	0	

3860478.583	0	0	0	0	
3855495.25	0	0	0	0	
3851775.034	0	0	0	0	
3851064.856	0	0	0	0	
3850847.669	0	0	0	0	
3850213.191	0	0	0	0	
3847920.485	0	0	0	0	
3842918.331	0	0	0	0	
3842390.778	0	0	0	0	
3842060.913	0	0	0	0	
3839567.563	0	0	0	0	
3839450.967	0	0	0	0	
3837591.846	0	0	0	0	
3831559.895	0	0	0	0	
3827567.543	0	0	0	0	
3812615.015	0	0	0	0	
3811135.387	0	0	0	0	
3796812.262	0	0	0	0	
3783803.023	0	0	0	0	
3780549.376	0	0	0	0	
3779527.19	0	0	0	0	
3751957.324	0	0	0	0	
3751725.244	0	0	0	0	
3750117.416	0	0	0	0	
3750109.615	0	0	0	0	
3742389.353	0	0	0	0	
3736810.071	0	0	0	0	
3734712.614	0	0	0	0	
3724827.703	0	0	0	0	
3723710.718	0	0	0	0	
3709327.608	0	0	0	0	
3697706.113	0	0	0	0	
3695182.858	0	0	0	0	
3688192.187	0	0	0	0	
3681796.067	0	0	0	0	
3680264.648	0	0	0	0	
3676197.283	0	0	0	0	
3672467.765	0	0	0	0	
3671092.111	0	0	0	0	
3665345.259	0	0	0	0	
3665239.06	0	0	0	0	
3662128.547	0	0	0	0	
3646453.441	0	0	0	0	
3646388.283	0	0	0	0	
3644398.707	0	0	0	0	
3643741.819	0	0	0	0	
3638533.293	0	0	0	0	
3635819.605	0	0	0	0	
3633263.831	0	0	0	0	
3628004.796	0	0	0	0	

3627531.563	0	0	0	0	
3626025.489	0	0	0	0	
3622833.913	0	0	0	0	
3617492.054	0	0	0	0	
3587876.394	0	0	0	0	
3585755.936	0	0	0	0	
3584363.443	0	0	0	0	
3583140.458	0	0	0	0	
3580855.34	0	0	0	0	
3578985.518	0	0	0	0	
3573137.64	0	0	0	0	
3570478.916	0	0	0	0	
3555799.771	0	0	0	0	
3552895.274	0	0	0	0	
3535536.65	0	0	0	0	
3531458.23	0	0	0	0	
3522271.226	0	0	0	0	
3493301.801	0	0	0	1	Superpathw
3491497.61	0	0	0	0	
3491256.783	0	0	0	0	
3484607.083	0	0	0	0	
3479425.466	0	0	0	0	
3474950.328	0	0	0	0	
3474090.436	0	0	0	0	
3473914.939	0	0	0	0	
3463513.412	0	0	0	0	
3458066.319	0	0	0	0	
3446387.662	0	0	0	0	
3439582.186	0	0	0	0	
3438051.107	0	0	0	0	
3436399.109	0	0	0	0	
3436303.579	0	0	0	0	
3436127.059	0	0	0	0	
3433880.381	0	0	0	0	
3433216.607	0	0	0	0	
3431969.509	0	0	0	0	
3424211.303	0	0	0	0	
3423416.143	0	0	0	0	
3403736.719	0	0	0	0	
3393331.508	0	0	0	0	
3391564.747	0	0	0	0	
3377796.825	0	0	0	0	
3369095.725	0	0	0	0	
3368907.458	0	0	0	0	
3368901.241	0	0	0	0	
3365586.875	0	0	0	0	
3360236.247	0	0	0	0	
3359256.996	0	0	0	0	
3339974.323	0	0	0	0	
3330421.394	0	0	0	0	

3327868.582	0	0	0	0	
3318517.627	0	0	0	0	
3310846.99	0	0	0	0	
3309857.576	0	0	0	1	Superpathw
3262836.881	0	0	0	0	
3260976.368	0	0	0	0	
3256841.068	0	0	0	0	
3236722.459	0	0	0	0	
3233609.704	0	0	0	0	
3229891.039	0	0	0	0	
3219891.69	0	0	0	0	
3218066.319	0	0	0	0	
3211794.233	0	0	0	0	
3205414.589	0	0	0	0	
3201295.802	0	0	0	0	
3200959.524	0	0	0	0	
3191268.94	0	0	0	0	
3181486.608	0	0	0	0	
3177861.749	0	0	0	0	
3171194.541	0	0	0	0	
3170112.417	0	0	0	0	
3162454.943	0	0	0	0	
3153253.068	0	0	0	0	
3143755.153	0	0	0	0	
3141178.341	0	0	0	0	
3130678.951	0	0	0	1	Other
3129398.409	0	0	0	0	
3123679.9	0	0	0	0	
3116046.887	0	0	0	0	
3107899.05	0	0	0	0	
3099966.901	0	0	0	0	
3098347.379	0	0	0	0	
3090730.489	0	0	0	0	
3083773.008	0	0	0	0	
3079434.316	0	0	0	0	
3077087.485	0	0	0	2	Superpathw
3062124.963	0	0	0	0	
3050573.096	0	0	0	0	
3046572.925	0	0	0	0	
3043988.965	0	0	0	0	
3038412.74	0	0	0	0	
3036646.352	0	0	0	0	
3033167.158	0	0	0	0	
3032506.368	0	0	0	0	
3018743.727	0	0	0	0	
3018044.768	0	0	0	0	
3017697.337	0	0	0	0	
3017218.367	0	0	0	0	
3013927.828	0	0	0	0	
3010175.828	0	0	0	0	

3010087.984	0	0	0	3	Other
2982913.529	0	0	0	0	
2974284.144	0	0	0	0	
2964448.859	0	0	0	0	
2957100.745	0	0	0	0	
2949769.391	0	0	0	0	
2949089.757	0	0	0	0	
2948090.425	0	0	0	2	Superpathw
2945746.317	0	0	0	0	
2943147.75	0	0	0	0	
2943053.164	0	0	0	0	
2941741.257	0	0	0	0	
2925506	0	0	0	0	
2921201.604	0	0	0	0	
2921178.08	0	0	0	0	
2919767.756	0	0	0	0	
2916309.521	0	0	0	0	
2892202.346	0	0	0	0	
2887773.477	0	0	0	0	
2859845.587	0	0	0	0	
2859511.267	0	0	0	0	
2856327.322	0	0	0	0	
2847819.974	0	0	0	0	
2846220.409	0	0	0	0	
2842122.154	0	0	0	0	
2839363.405	0	0	0	0	
2839310.076	0	0	0	0	
2838881.19	0	0	0	0	
2830247.99	0	0	0	0	
2826517.462	0	0	0	0	
2821254.369	0	0	0	0	
2818704.574	0	0	0	0	
2817990.676	0	0	0	0	
2811068.143	0	0	0	0	
2801742.539	0	0	0	0	
2798699.755	0	0	0	0	
2797135.593	0	0	0	0	
2792234.775	0	0	0	0	
2787481.661	0	0	0	0	
2777804.046	0	0	0	0	
2772142.247	0	0	0	0	
2762935.888	0	0	0	0	
2761303.927	0	0	0	0	
2757120.121	0	0	0	0	
2756823.55	0	0	0	0	
2742304.746	0	0	0	0	
2741870.633	0	0	0	0	
2730109.715	0	0	0	0	
2720957.214	0	0	0	0	
2720716.346	0	0	0	0	

2713832.555	0	0	0	0	
2706373.782	0	0	0	0	
2695245.236	0	0	0	0	
2682759.55	0	0	0	0	
2679725.716	0	0	0	0	
2676111.705	0	0	0	0	
2668578.974	0	0	0	0	
2655183.62	0	0	0	0	
2637480.087	0	0	0	0	
2631390.433	0	0	0	0	
2614715.006	0	0	0	0	
2604804.691	0	0	0	0	
2601597.068	0	0	0	0	
2595569.633	0	0	0	0	
2594893.225	0	0	0	0	
2584992.417	0	0	0	0	
2583965.125	0	0	0	0	
2579088.272	0	0	0	0	
2565084.889	0	0	0	0	
2561733.644	0	0	0	0	
2561187.078	0	0	0	0	
2543033.363	0	0	0	0	
2542472.163	0	0	0	0	
2538660.48	0	0	0	0	
2507177.436	0	0	0	0	
2505637.263	0	0	0	0	
2489901.388	0	0	0	0	
2486475.228	0	0	0	0	
2480953.098	0	0	0	0	
2476596.851	0	0	0	0	
2459563.347	0	0	0	0	
2447373.139	0	0	0	0	
2441030.662	0	0	0	0	
2440504.587	0	0	0	0	
2437839.017	0	0	0	0	
2433669.363	0	0	0	0	
2399105.027	0	0	0	0	
2397263.267	0	0	0	0	
2394983.812	0	0	0	0	
2393229.392	0	0	0	0	
2392579.231	0	0	0	1	Superpathw
2391306.028	0	0	0	0	
2381711.04	0	0	0	0	
2378653.206	0	0	0	0	
2371879.171	0	0	0	0	
2368964.171	0	0	0	0	
2362319.252	0	0	0	0	
2354089.934	0	0	0	0	
2353254.225	0	0	0	0	
2351133.088	0	0	0	0	

2336412.09	0	0	0	0	
2335600.895	0	0	0	0	
2329772.52	0	0	0	0	
2327990.828	0	0	0	0	
2325191.196	0	0	0	0	
2314379.713	0	0	0	0	
2300708.524	0	0	0	0	
2298316.63	0	0	0	0	
2284131.832	0	0	0	0	
2278395.672	0	0	0	0	
2266234.544	0	0	0	0	
2265097.568	0	0	0	0	
2254605.023	0	0	0	0	
2247051.941	0	0	0	0	
2241216.405	0	0	0	0	
2227294.901	0	0	0	0	
2224764.862	0	0	0	0	
2212531.783	0	0	0	0	
2195991.057	0	0	0	0	
2191736.627	0	0	0	0	
2191509.57	0	0	0	0	
2183437.662	0	0	0	0	
2183051.01	0	0	0	0	
2181359.361	0	0	0	0	
2181122.105	0	0	0	0	
2168011.769	0	0	0	0	
2141608.987	0	0	0	0	
2125415.707	0	0	0	0	
2123285.92	0	0	0	0	
2110330.622	0	0	0	0	
2103742.665	0	0	0	0	
2093197.04	0	0	0	0	
2093169.099	0	0	0	0	
2085107.314	0	0	0	0	
2075344.631	0	0	0	0	
2065705.921	0	0	0	0	
2051568.723	0	0	0	0	
2038996.906	0	0	0	0	
2032160.127	0	0	0	0	
2029748.317	0	0	0	0	
1999116.132	0	0	0	0	
1980739.657	0	0	0	0	
1971382.014	0	0	0	0	
1968564.316	0	0	0	0	
1961259.789	0	0	0	0	
1957483.532	0	0	0	0	
1950951.761	0	0	0	0	
1943907.403	0	0	0	0	
1919175.266	0	0	0	0	
1911815.043	0	0	0	0	

1909971.808	0	0	0	0	
1857370.009	0	0	0	0	
1841954.223	0	0	0	0	
1744709.402	0	0	0	0	
1738968.214	0	0	0	0	
1715320.864	0	0	0	1	Other
1707520.554	0	0	0	0	
1705434.212	0	0	0	0	
1690184.302	0	0	0	0	
1686369.876	0	0	0	0	
1677855.707	0	0	0	1	Other
1663230.608	0	0	0	0	
1659105.409	0	0	0	0	
1656725.972	0	0	0	0	
1635488.377	0	0	0	0	
1593514.919	0	0	0	0	
1590199.184	0	0	0	0	
1564284.04	0	0	0	0	
1563846.493	0	0	0	0	
1561007.858	0	0	0	0	
1560399.871	0	0	0	0	
1554674.806	0	0	0	0	
1541488.781	0	0	0	0	
1529856.928	0	0	0	0	
1517300.214	0	0	0	0	
1488977.645	0	0	0	0	
1487971.829	0	0	0	0	
1453532.026	0	0	0	0	
1429601.98	0	0	0	0	
1378869.11	0	0	0	0	
1363289.396	0	0	0	0	
1362555.241	0	0	0	0	
1312816.922	0	0	0	0	
1293675.192	0	0	0	0	
1287636.163	0	0	0	0	
1280550.087	0	0	0	0	
1279589.254	0	0	0	0	
1277694.954	0	0	0	0	
1235905.17	0	0	0	0	
1161696.727	0	0	0	0	
1146158.079	0	0	0	0	
1073415.875	0	0	0	0	
1056858.527	0	0	0	0	
1013520.818	0	0	0	0	

mzCloud Bes	Mass List Ma	Mass List Ma	Mass List Ma	Mass List Ma	mzVault Bes	MS2	Group Area:
	No matches	Single match	Single match	No matches	92.4	DDA for pref	1.353E+11
	No matches	No matches	No matches	No matches	93.9	DDA for othe	4.488E+09
	No matches	No matches	No matches	No matches	94	DDA for pref	4.324E+10
	No matches	No matches	No matches	No matches	94	DDA for pref	1.823E+10
	No matches	No matches	No matches	Single match		DDA for pref	1.711E+10
	No matches	No matches	No matches	No matches		DDA for pref	2.901E+09
	No matches	No matches	Multiple ma	Single match		DDA for pref	8.865E+09
	No matches	No matches	No matches	No matches		DDA for pref	6.554E+09
	Multiple ma	No matches	Multiple ma	No matches	90.6	DDA for pref	226139685
	No matches	No matches	No matches	No matches		DDA for pref	7.058E+09
	Multiple ma	No matches	Single match	No matches	88.9	DDA for pref	5.327E+09
	No matches	No matches	Multiple ma	No matches	90.7	DDA for pref	6.805E+09
	No matches	No matches	No matches	No matches	94	DDA for pref	408621060
	Multiple ma	No matches	No matches	No matches		DDA for pref	4.995E+09
	No matches	No matches	No matches	No matches		DDA for pref	4.982E+09
	No matches	No matches	No matches	No matches		DDA for pref	3.346E+09
	No matches	No matches	No matches	No matches		DDA for pref	4.038E+09
	No matches	No matches	No matches	No matches		DDA for pref	3.876E+09
	No matches	Single match	No matches	Single match		DDA for othe	4.557E+09
	No matches	No matches	No matches	No matches		DDA for pref	4.06E+09
	Multiple ma	No matches	Single match	No matches	86.8	DDA for pref	3.943E+09
	No matches	No matches	Single match	No matches		DDA for pref	4.053E+09
	No matches	Multiple ma	Multiple ma	No matches	74.7	DDA for pref	4.328E+09
	No matches	No matches	Multiple ma	Single match		DDA for pref	3.738E+09
	Multiple ma	No matches	Multiple ma	No matches	89.8	DDA for pref	3.034E+09
	No matches	Single match	Single match	No matches		DDA for pref	3.516E+09
	No matches	No matches	No matches	No matches		DDA for pref	3.221E+09
	No matches	No matches	Multiple ma	No matches		DDA for pref	2.461E+09
	No matches	No matches	No matches	No matches		No MS2	3.067E+09
	No matches	No matches	Single match	No matches	90.4	DDA for pref	3.545E+09
	No matches	No matches	No matches	No matches	89.8	DDA for othe	3.533E+09
	No matches	No matches	Single match	No matches		DDA for pref	2.993E+09
	No matches	No matches	No matches	No matches		DDA for pref	2.935E+09
	No matches	No matches	No matches	No matches		DDA for pref	3.133E+09
	No matches	Single match	Single match	No matches		DDA for pref	3.158E+09
	No matches	No matches	No matches	No matches		DDA for pref	2.211E+09
	No matches	No matches	No matches	No matches		DDA for othe	2.184E+09
	No matches	No matches	No matches	No matches		DDA for othe	2.338E+09
	No matches	No matches	No matches	Single match		DDA for othe	2.647E+09
	Multiple ma	No matches	Single match	No matches		DDA for pref	2.352E+09
	No matches	No matches	No matches	No matches		DDA for pref	2.612E+09
	No matches	No matches	No matches	No matches		No MS2	1.645E+09
	No matches	Single match	Single match	No matches		DDA for pref	611987727
	Multiple ma	No matches	No matches	No matches		DDA for pref	2.489E+09
	No matches	No matches	No matches	No matches		DDA for pref	2.149E+09
	No matches	No matches	No matches	No matches	83.3	DDA for othe	2.526E+09
	Multiple ma	No matches	Multiple ma	No matches	90.6	DDA for pref	1.183E+09
	No matches	No matches	No matches	No matches		DDA for pref	1.748E+09

	No matches	No matches	Single match	No matches	86.5	DDA for pref	2.317E+09
	No matches	No matches	No matches	No matches	90.2	DDA for othe	2.286E+09
	No matches	No matches	No matches	No matches		No MS2	1.238E+09
	No matches	No matches	No matches	No matches		DDA for pref	2.035E+09
	No matches	No matches	No matches	No matches		DDA for pref	2.196E+09
	No matches	No matches	No matches	No matches		DDA for pref	2.187E+09
	No matches	No matches	Multiple ma	No matches		DDA for pref	1.92E+09
	No matches	No matches	Single match	Single match		DDA for pref	407078588
	No matches	No matches	No matches	No matches		DDA for pref	2.06E+09
	No matches	No matches	No matches	No matches		No MS2	1.661E+09
	No matches	No matches	No matches	No matches	94	DDA for othe	1.303E+09
	No matches	No matches	No matches	No matches		DDA for pref	1.373E+09
	No matches	No matches	No matches	No matches		DDA for pref	1.469E+09
	No matches	No matches	No matches	No matches		DDA for pref	13425137
	No matches	No matches	Multiple ma	No matches		DDA for pref	1.587E+09
	No matches	No matches	No matches	No matches		DDA for pref	1.516E+09
	No matches	No matches	No matches	No matches		DDA for othe	981384628
	No matches	No matches	No matches	No matches		DDA for pref	1.735E+09
	No matches	No matches	Single match	No matches		DDA for pref	1.375E+09
	No matches	No matches	No matches	No matches		DDA for pref	14769127
	No matches	No matches	No matches	No matches		DDA for pref	1.769E+09
	No matches	No matches	Multiple ma	No matches		DDA for pref	1.45E+09
	No matches	No matches	No matches	No matches		DDA for pref	1.612E+09
	No matches	No matches	No matches	No matches		DDA for pref	1.342E+09
	No matches	No matches	Single match	No matches		DDA for pref	1.392E+09
	No matches	No matches	No matches	No matches		DDA for pref	1031814.4
	No matches	No matches	Single match	No matches	86	DDA for pref	1.44E+09
	No matches	No matches	Single match	No matches		DDA for pref	1.113E+09
	No matches	No matches	No matches	No matches	92.1	DDA for pref	4325131.2
	No matches	Multiple ma	Multiple ma	No matches	74.3	DDA for pref	1.285E+09
	No matches	No matches	No matches	No matches		DDA for pref	1.535E+09
	No matches	No matches	No matches	No matches		DDA for pref	1.04E+09
	No matches	No matches	No matches	No matches		DDA for pref	1.477E+09
	Multiple ma	No matches	Single match	No matches	85.4	DDA for pref	1.472E+09
	No matches	No matches	No matches	Single match		DDA for pref	977811358
	No matches	No matches	No matches	No matches		DDA for othe	15600896
	Multiple ma	No matches	Multiple ma	No matches	90.4	DDA for pref	1.277E+09
	No matches	No matches	Single match	No matches		DDA for pref	1.318E+09
	Multiple ma	No matches	No matches	No matches		DDA for pref	1.083E+09
	No matches	No matches	No matches	No matches		DDA for pref	1.141E+09
	No matches	No matches	No matches	No matches		DDA for othe	1.016E+09
	No matches	No matches	No matches	No matches		No MS2	1.002E+09
	No matches	No matches	Multiple ma	No matches		DDA for pref	829075464
	No matches	No matches	No matches	No matches		No MS2	927275526
	No matches	No matches	Single match	No matches		DDA for othe	933867840
	No matches	No matches	No matches	No matches		DDA for othe	923428882
	No matches	No matches	No matches	No matches		DDA for othe	918127892
	No matches	No matches	No matches	No matches		DDA for pref	25299075
	No matches	No matches	Multiple ma	No matches		DDA for pref	1.249E+09
	No matches	Single match	Multiple ma	No matches	93.9	DDA for pref	1.246E+09

	Multiple ma	No matches	No matches	No matches		DDA for pref	927078862
	No matches	No matches	Single match	No matches	94.9	DDA for pref	1.105E+09
	No matches	No matches	No matches	No matches		DDA for pref	390626480
	No matches	Single match	No matches	No matches		DDA for othe	1.078E+09
	No matches	No matches	Multiple ma	No matches		DDA for othe	1.027E+09
	No matches	No matches	No matches	No matches		DDA for pref	2140627.9
	No matches	No matches	Multiple ma	No matches		DDA for pref	961670702
	No matches	No matches	No matches	No matches	92.2	DDA for pref	1.1E+09
	No matches	No matches	No matches	No matches		DDA for pref	875416452
	No matches	No matches	No matches	No matches		DDA for pref	1.097E+09
	No matches	No matches	No matches	No matches		DDA for pref	880555627
	No matches	No matches	Single match	No matches		DDA for pref	988671586
	No matches	No matches	No matches	No matches		DDA for pref	817534463
	No matches	Single match	Multiple ma	No matches	89.8	DDA for pref	1.047E+09
	Multiple ma	No matches	Multiple ma	No matches	91.6	DDA for pref	608109723
	No matches	No matches	No matches	No matches		No MS2	852385636
	No matches	No matches	Single match	No matches		DDA for pref	1.041E+09
	No matches	No matches	No matches	No matches		No MS2	531062793
	No matches	No matches	No matches	No matches		DDA for pref	895459916
	No matches	No matches	No matches	No matches		DDA for pref	1.021E+09
	No matches	No matches	No matches	No matches		DDA for othe	1.004E+09
	No matches	No matches	Multiple ma	No matches		DDA for pref	296657170
	No matches	No matches	No matches	No matches		No MS2	473195778
	No matches	No matches	No matches	No matches		DDA for pref	463198637
	Multiple ma	No matches	Multiple ma	No matches	90.4	DDA for pref	677449773
	No matches	No matches	No matches	No matches		No MS2	651379899
	No matches	No matches	No matches	No matches		DDA for pref	961897953
	No matches	No matches	No matches	No matches		DDA for pref	958661524
	Multiple ma	No matches	Multiple ma	No matches	85	DDA for pref	865569649
	No matches	No matches	Multiple ma	No matches		DDA for pref	840564145
	No matches	No matches	No matches	No matches		DDA for pref	95725920
	No matches	No matches	No matches	No matches		DDA for pref	850148504
	No matches	No matches	No matches	Single match		DDA for pref	336758983
	No matches	No matches	No matches	No matches	83.5	DDA for pref	707344636
	No matches	No matches	No matches	No matches		No MS2	697817437
	No matches	No matches	No matches	No matches		DDA for pref	908465865
	No matches	No matches	No matches	No matches		DDA for pref	625034145
	No matches	No matches	No matches	No matches		No MS2	644049430
	No matches	No matches	No matches	No matches		DDA for othe	689421757
	No matches	No matches	No matches	No matches		DDA for pref	631960906
	No matches	No matches	No matches	No matches		No MS2	890839364
	No matches	No matches	No matches	Multiple ma		DDA for pref	883240903
	No matches	Single match	No matches	No matches		No MS2	559611023
	No matches	No matches	No matches	No matches		DDA for pref	776687823
	No matches	No matches	No matches	No matches		DDA for othe	735535798
	No matches	No matches	No matches	No matches		No MS2	465652111
	No matches	No matches	No matches	No matches	84.7	DDA for othe	845135339
	No matches	No matches	No matches	No matches		No MS2	690951463
	No matches	No matches	No matches	No matches		No MS2	652991770
	No matches	No matches	No matches	No matches		DDA for pref	751686368

	No matches	No matches	No matches	No matches		DDA for pref	654229660
	No matches	No matches	No matches	No matches		DDA for pref	556025178
	No matches	No matches	No matches	Single match		DDA for pref	497769795
	No matches	No matches	No matches	No matches		DDA for pref	827251333
	No matches	No matches	No matches	No matches		DDA for othe	704187511
	Multiple ma	No matches	No matches	No matches		DDA for pref	701066390
	No matches	No matches	No matches	No matches		DDA for pref	695937470
	No matches	No matches	Multiple ma	Multiple ma	87.6	DDA for othe	805511741
	No matches	No matches	No matches	No matches		No MS2	511800035
	No matches	No matches	No matches	No matches		DDA for pref	611838675
	No matches	Multiple ma	Multiple ma	Single match	88.7	DDA for pref	794903535
	No matches	No matches	No matches	No matches	86.3	DDA for pref	789351776
	No matches	No matches	No matches	No matches		No MS2	722242555
	No matches	Single match	No matches	No matches		DDA for othe	778577683
	No matches	No matches	No matches	No matches		DDA for pref	699215810
	No matches	No matches	No matches	No matches		DDA for pref	673571523
	No matches	No matches	No matches	No matches		DDA for pref	771095856
	No matches	No matches	No matches	No matches		DDA for pref	434854046
	No matches	Multiple ma	Multiple ma	No matches	69.5	DDA for pref	761828278
	No matches	No matches	No matches	No matches		DDA for pref	750589444
	No matches	No matches	No matches	Single match		DDA for othe	363452412
	No matches	No matches	No matches	No matches		DDA for othe	497307569
	No matches	No matches	No matches	No matches		DDA for pref	173111744
	No matches	No matches	No matches	No matches		DDA for pref	598322291
	No matches	No matches	No matches	No matches		DDA for pref	655261292
	No matches	No matches	No matches	No matches		DDA for pref	6903227.7
	No matches	No matches	No matches	No matches		DDA for pref	722924092
	No matches	No matches	No matches	Single match		DDA for pref	722122942
	No matches	No matches	Single match	No matches		DDA for pref	607971971
	No matches	No matches	No matches	No matches		DDA for pref	630045956
	No matches	Multiple ma	No matches	No matches	89.3	DDA for othe	716962247
	No matches	No matches	No matches	No matches		DDA for pref	713176320
	No matches	No matches	No matches	No matches		No MS2	705633821
	No matches	No matches	No matches	No matches		DDA for pref	528207818
	No matches	No matches	No matches	No matches		DDA for othe	694403805
	No matches	No matches	No matches	No matches		DDA for othe	690132194
	No matches	No matches	No matches	No matches		DDA for pref	429169471
	No matches	No matches	Multiple ma	No matches		DDA for pref	634505685
	No matches	No matches	No matches	No matches		DDA for pref	594450736
	No matches	No matches	No matches	No matches		DDA for pref	666499908
	No matches	No matches	No matches	No matches		DDA for pref	484502831
	No matches	No matches	No matches	No matches		No MS2	658048859
	No matches	No matches	No matches	No matches		DDA for pref	375303138
	No matches	No matches	No matches	No matches		DDA for pref	541743884
	No matches	No matches	No matches	No matches	89	DDA for othe	180885156
	No matches	No matches	No matches	No matches		DDA for pref	184122437
	No matches	No matches	No matches	No matches		DDA for pref	640939424
	No matches	No matches	Single match	No matches		DDA for othe	480888625
	No matches	No matches	No matches	No matches		DDA for othe	431801826
	Multiple ma	No matches	Multiple ma	No matches	89.9	DDA for pref	355871089

	No matches	Single match	No matches	No matches		DDA for pref	431793747
	No matches	No matches	No matches	No matches		No MS2	381886354
	No matches	No matches	No matches	No matches		DDA for pref	501509050
	No matches	No matches	No matches	No matches		DDA for pref	389601668
	No matches	No matches	No matches	No matches		DDA for pref	377922574
	No matches	No matches	No matches	No matches		DDA for othe	323179592
	No matches	No matches	No matches	Single match		DDA for pref	419341747
	No matches	No matches	No matches	No matches		DDA for pref	430342612
	No matches	No matches	No matches	No matches		No MS2	4369170.1
	No matches	No matches	No matches	No matches		DDA for pref	576936949
	No matches	No matches	No matches	No matches		DDA for pref	415959448
	No matches	No matches	Multiple ma	No matches		DDA for pref	359200036
	Multiple ma	No matches	Multiple ma	No matches	87.8	DDA for pref	562169249
	No matches	No matches	No matches	No matches		DDA for pref	318057428
	No matches	No matches	Single match	No matches		DDA for pref	532681629
	Multiple ma	No matches	Multiple ma	No matches	87.4	DDA for pref	404819059
	No matches	No matches	No matches	No matches		DDA for pref	456815815
	No matches	No matches	No matches	No matches		DDA for othe	557247897
	No matches	Single match	Single match	No matches		DDA for othe	454778999
	No matches	No matches	No matches	No matches		DDA for pref	48299259
	No matches	No matches	Single match	No matches		DDA for othe	553209608
	No matches	No matches	No matches	No matches		DDA for pref	175287201
	No matches	No matches	No matches	No matches		No MS2	445167594
	No matches	Single match	No matches	No matches		DDA for pref	406914100
	No matches	No matches	No matches	No matches		No MS2	460241071
	No matches	No matches	No matches	No matches		DDA for pref	347685812
	No matches	No matches	No matches	No matches		DDA for pref	423955627
	No matches	No matches	Single match	No matches		No MS2	406468761
	No matches	No matches	No matches	No matches		No MS2	417445566
	Single match	No matches	Multiple ma	No matches		DDA for pref	451908310
	No matches	Single match	Single match	No matches		No MS2	477731498
	No matches	Multiple ma	No matches	No matches		No MS2	486096377
	No matches	No matches	Multiple ma	No matches		DDA for pref	529093732
	No matches	No matches	No matches	No matches		DDA for pref	464680272
	No matches	Single match	No matches	No matches		No MS2	465892227
	No matches	No matches	No matches	No matches		No MS2	479484425
	No matches	No matches	Multiple ma	No matches		DDA for othe	513811144
	No matches	No matches	Multiple ma	No matches	91	DDA for pref	499845804
	No matches	No matches	No matches	No matches		DDA for pref	268145796
	No matches	No matches	Single match	No matches		DDA for pref	307882455
	No matches	No matches	No matches	No matches		DDA for othe	420184226
	No matches	No matches	No matches	Single match		DDA for pref	26536429
	Multiple ma	No matches	Multiple ma	No matches	91.8	DDA for pref	267392221
	No matches	No matches	No matches	No matches		DDA for pref	498001383
	No matches	No matches	No matches	Single match		DDA for pref	388961642
	No matches	No matches	No matches	No matches		DDA for othe	469725065
	No matches	No matches	No matches	Single match		DDA for pref	1928125.5
	No matches	No matches	No matches	No matches		DDA for pref	496025225
	No matches	No matches	No matches	No matches		DDA for pref	395012707
	No matches	No matches	No matches	No matches		DDA for pref	85931144

	No matches	No matches	No matches	No matches		No MS2	321264891
	No matches	No matches	No matches	No matches		No MS2	486345860
	No matches	No matches	No matches	No matches		DDA for pref	340387665
	No matches	No matches	No matches	No matches		DDA for pref	125498529
	No matches	No matches	No matches	No matches		DDA for pref	399930479
	Multiple ma	No matches	No matches	No matches		No MS2	458703825
	No matches	No matches	No matches	No matches		DDA for pref	1105031.2
	No matches	No matches	Single match	No matches		DDA for pref	295315474
	No matches	No matches	No matches	No matches		DDA for pref	463665525
	No matches	No matches	No matches	No matches		DDA for othe	346127949
	No matches	No matches	Single match	No matches		DDA for pref	108582841
	No matches	No matches	No matches	No matches		DDA for pref	366563011
	No matches	No matches	Single match	Single match		DDA for othe	394472429
	No matches	No matches	No matches	No matches		DDA for othe	358367520
	No matches	No matches	No matches	No matches		DDA for othe	380273145
	No matches	No matches	No matches	No matches		No MS2	360718998
	No matches	No matches	No matches	No matches		DDA for pref	5178749.7
	No matches	No matches	No matches	No matches		DDA for pref	445415101
	No matches	No matches	No matches	No matches		No MS2	352380266
	No matches	No matches	No matches	No matches		DDA for othe	270469999
	No matches	No matches	No matches	No matches		DDA for pref	371251760
	No matches	No matches	Multiple ma	No matches		DDA for othe	310263973
	No matches	No matches	Single match	No matches	86.1	DDA for pref	424399999
	No matches	No matches	No matches	No matches		DDA for othe	3394731.3
	No matches	No matches	No matches	No matches		DDA for othe	401381942
	No matches	No matches	No matches	No matches		DDA for pref	368728800
	No matches	No matches	No matches	No matches		DDA for pref	435156055
	No matches	No matches	No matches	No matches		DDA for pref	299585675
	No matches	No matches	No matches	No matches		No MS2	431443502
	No matches	No matches	Single match	No matches		DDA for pref	323472634
	No matches	No matches	No matches	No matches		No MS2	137756275
	No matches	No matches	No matches	No matches		DDA for pref	358981068
	No matches	No matches	Single match	No matches		DDA for othe	381269857
	No matches	No matches	No matches	No matches		DDA for pref	330148423
	No matches	No matches	No matches	No matches		DDA for pref	381575671
	No matches	No matches	No matches	No matches		No MS2	360614271
	No matches	No matches	No matches	No matches		No MS2	415986515
	No matches	No matches	No matches	No matches		No MS2	415582829
	No matches	No matches	No matches	No matches		DDA for othe	382471291
	No matches	No matches	No matches	No matches		No MS2	413294925
	No matches	No matches	Single match	No matches		DDA for pref	329575253
	No matches	No matches	No matches	No matches		No MS2	332495453
	No matches	No matches	No matches	No matches		DDA for othe	297049268
	No matches	No matches	No matches	No matches		DDA for pref	375387393
	No matches	No matches	Multiple ma	No matches		DDA for pref	407033138
	No matches	Single match	No matches	No matches		DDA for pref	295577584
	No matches	No matches	Multiple ma	No matches		No MS2	324959449
	No matches	No matches	Single match	No matches		DDA for pref	302927963
	No matches	No matches	No matches	No matches		DDA for pref	211016893
	No matches	Multiple ma	No matches	No matches		DDA for pref	387210835

	No matches	No matches	No matches	No matches		DDA for othe	272625454
	No matches	No matches	No matches	No matches		No MS2	293068358
	Multiple ma	Single match	No matches	No matches		DDA for pref	396425149
	No matches	No matches	No matches	No matches		DDA for pref	393358869
	No matches	No matches	No matches	No matches		DDA for othe	15446966
	No matches	Single match	No matches	No matches		DDA for pref	237013092
	No matches	No matches	Single match	No matches		No MS2	258804234
	No matches	No matches	No matches	No matches		DDA for pref	26894864
	Multiple ma	No matches	Single match	No matches	92.4	DDA for pref	363818493
	No matches	No matches	Single match	No matches		No MS2	321391970
	No matches	No matches	Multiple ma	No matches		DDA for pref	358112589
	Multiple ma	No matches	No matches	No matches		DDA for pref	373779952
	No matches	No matches	No matches	No matches		No MS2	365127036
	No matches	No matches	No matches	No matches		No MS2	210572946
	No matches	No matches	No matches	No matches		DDA for othe	346318110
	No matches	Multiple ma	Single match	No matches		DDA for pref	346992534
	No matches	No matches	No matches	No matches		DDA for pref	360003763
	No matches	No matches	No matches	No matches		DDA for pref	333750035
	No matches	No matches	No matches	No matches		DDA for pref	358331018
	No matches	Single match	No matches	Single match		DDA for othe	357307440
	Multiple ma	No matches	No matches	No matches		No MS2	330016500
	No matches	No matches	No matches	No matches		No MS2	355146892
	Multiple ma	No matches	Multiple ma	No matches	91.4	DDA for pref	308894801
	No matches	No matches	No matches	No matches		DDA for pref	223057600
	No matches	No matches	No matches	No matches		DDA for pref	353828997
	No matches	No matches	No matches	No matches		DDA for pref	261329536
	No matches	No matches	No matches	No matches		DDA for pref	318231235
	No matches	No matches	No matches	No matches		DDA for pref	185097874
	No matches	No matches	No matches	No matches		DDA for pref	263445616
	No matches	No matches	No matches	No matches		DDA for othe	206633312
	No matches	No matches	Multiple ma	No matches		DDA for othe	292203304
	No matches	No matches	No matches	No matches		No MS2	274892713
	Multiple ma	No matches	No matches	Single match		DDA for pref	337953448
	No matches	No matches	Single match	No matches		No MS2	258038806
	No matches	No matches	No matches	Single match		DDA for pref	218692006
	No matches	No matches	No matches	No matches		DDA for pref	238126885
	No matches	No matches	No matches	No matches		DDA for pref	343995513
	No matches	No matches	No matches	No matches		DDA for pref	311421713
	No matches	No matches	No matches	No matches	93.7	DDA for pref	334793551
	No matches	No matches	No matches	No matches		DDA for pref	262475588
	No matches	No matches	No matches	No matches		DDA for pref	316635184
	No matches	No matches	No matches	No matches		No MS2	294689459
	No matches	No matches	No matches	No matches		DDA for othe	317093418
	No matches	No matches	No matches	No matches		No MS2	336801910
	No matches	No matches	No matches	No matches		No MS2	219025338
	No matches	No matches	No matches	No matches		No MS2	335616396
	Multiple ma	Single match	Multiple ma	No matches		No MS2	294906763
	No matches	No matches	No matches	No matches		DDA for pref	281606123
	No matches	No matches	No matches	No matches		No MS2	243912612
	No matches	No matches	No matches	No matches		No MS2	291433938

	No matches	No matches	Multiple ma	No matches		DDA for pref	251790805
	No matches	No matches	No matches	No matches		No MS2	290604979
	No matches	No matches	No matches	No matches		DDA for othe	257983891
	No matches	No matches	No matches	No matches		DDA for pref	258207166
	No matches	No matches	No matches	No matches		No MS2	326067494
	No matches	Single match	No matches	Single match		No MS2	235274289
	Multiple ma	No matches	No matches	No matches		No MS2	275924440
	No matches	No matches	No matches	No matches		DDA for pref	325428026
	No matches	No matches	No matches	No matches		No MS2	250691666
	No matches	No matches	Single match	No matches		No MS2	181204888
	No matches	No matches	No matches	No matches		No MS2	226374356
	No matches	No matches	Multiple ma	No matches		DDA for pref	249860069
	No matches	No matches	No matches	No matches		DDA for othe	194445086
	No matches	No matches	Single match	No matches		No MS2	264642341
	Multiple ma	No matches	Single match	No matches		DDA for pref	215008962
	No matches	No matches	No matches	No matches		No MS2	229268255
	No matches	No matches	No matches	No matches		No MS2	200300147
	No matches	No matches	Multiple ma	No matches		No MS2	183132888
	No matches	No matches	Single match	No matches		No MS2	245163529
	No matches	No matches	Multiple ma	Multiple ma	87.9	DDA for pref	255619733
	Multiple ma	No matches	Multiple ma	No matches		DDA for pref	311928913
	No matches	No matches	Single match	No matches		No MS2	174699623
	Multiple ma	No matches	Single match	No matches		No MS2	243037060
	No matches	No matches	No matches	No matches	87.4	DDA for pref	286000429
	No matches	No matches	No matches	No matches		DDA for pref	256882230
	No matches	No matches	No matches	Multiple ma		No MS2	273737197
	No matches	No matches	No matches	No matches		DDA for pref	238647090
	No matches	No matches	No matches	No matches		No MS2	2234241
	No matches	No matches	No matches	No matches	86.6	DDA for pref	2775308.3
	No matches	No matches	No matches	No matches		DDA for othe	306684552
	No matches	No matches	No matches	No matches		No MS2	156097988
	No matches	No matches	No matches	No matches		No MS2	276311286
	No matches	No matches	No matches	No matches		No MS2	300222143
	No matches	No matches	No matches	No matches		DDA for pref	154327894
	Multiple ma	No matches	Multiple ma	No matches		No MS2	189170645
	Multiple ma	No matches	Single match	No matches	83.8	DDA for pref	255990974
	No matches	No matches	No matches	No matches	78	DDA for pref	187949642
	No matches	No matches	No matches	No matches		No MS2	295430671
	No matches	No matches	No matches	No matches		No MS2	163854263
	No matches	No matches	Multiple ma	No matches		No MS2	293432782
	No matches	No matches	No matches	No matches		DDA for pref	201883462
	No matches	No matches	No matches	No matches		DDA for pref	3152245.9
	No matches	No matches	Multiple ma	No matches		No MS2	170431799
	No matches	No matches	No matches	Single match		No MS2	292150747
	No matches	No matches	Single match	No matches	91	DDA for pref	284954512
	No matches	Multiple ma	Single match	Multiple ma		DDA for pref	174732757
	No matches	No matches	No matches	No matches		DDA for pref	247836159
	Multiple ma	No matches	Multiple ma	No matches	87.7	DDA for pref	188077135
	No matches	No matches	No matches	No matches		DDA for othe	287473073
	No matches	No matches	Single match	No matches	75.4	DDA for pref	235888983

	No matches	No matches	Multiple ma	Single match	86.8	DDA for othe	230309398
	No matches	Single match	No matches	No matches		DDA for pref	199776923
	No matches	No matches	No matches	No matches		No MS2	246420289
	No matches	No matches	No matches	No matches		DDA for othe	214346050
	No matches	No matches	No matches	No matches		No MS2	283375860
	No matches	No matches	No matches	No matches		No MS2	187393555
	No matches	No matches	Single match	No matches		No MS2	252814151
	No matches	No matches	No matches	No matches		DDA for othe	278622344
	No matches	No matches	No matches	No matches		DDA for othe	167779106
	No matches	No matches	No matches	No matches		DDA for pref	273078093
	No matches	No matches	Single match	No matches		DDA for pref	237946117
	No matches	No matches	Single match	No matches		No MS2	236684135
	No matches	No matches	No matches	No matches		DDA for pref	166178630
	No matches	No matches	No matches	No matches		DDA for pref	270629602
	No matches	No matches	No matches	No matches		DDA for pref	1362484.2
	No matches	No matches	Single match	No matches		No MS2	269293788
	No matches	No matches	No matches	No matches		No MS2	227357410
	No matches	No matches	No matches	No matches		DDA for pref	179736444
	No matches	No matches	No matches	No matches		DDA for pref	267671040
	No matches	No matches	No matches	No matches		No MS2	96685238
	No matches	No matches	No matches	No matches		DDA for pref	888159.76
	No matches	No matches	No matches	Single match		DDA for pref	242499521
	No matches	No matches	No matches	No matches		No MS2	203663435
	No matches	No matches	No matches	No matches		No MS2	155371218
	No matches	No matches	No matches	No matches		No MS2	155371218
	No matches	No matches	No matches	No matches		DDA for pref	154714093
	No matches	No matches	No matches	No matches		DDA for pref	58304832
	No matches	Single match	No matches	No matches		No MS2	166858489
	No matches	No matches	No matches	No matches		DDA for pref	257651893
	No matches	No matches	No matches	No matches		DDA for pref	170466676
	No matches	No matches	No matches	No matches		No MS2	256013282
	No matches	No matches	Single match	No matches		DDA for othe	241176227
	No matches	No matches	No matches	No matches		DDA for pref	200303205
	No matches	No matches	Multiple ma	No matches		No MS2	249633979
	No matches	No matches	No matches	No matches		No MS2	180167030
	No matches	No matches	Multiple ma	No matches		No MS2	213698542
	No matches	No matches	Multiple ma	No matches		No MS2	169887290
	No matches	No matches	No matches	No matches		DDA for pref	203086728
	No matches	No matches	No matches	No matches		No MS2	221962582
	No matches	No matches	No matches	No matches		No MS2	249685035
	No matches	No matches	No matches	No matches		DDA for pref	249222706
	No matches	No matches	Single match	No matches		DDA for othe	156711961
	No matches	No matches	No matches	No matches		No MS2	179579807
	No matches	No matches	No matches	No matches		DDA for pref	16417703
	No matches	No matches	No matches	No matches		No MS2	245977495
	Multiple ma	No matches	Single match	No matches	67.6	DDA for pref	212972359
	No matches	No matches	No matches	No matches		No MS2	201785321
	No matches	No matches	Multiple ma	No matches		DDA for pref	218014951
	No matches	No matches	No matches	No matches		DDA for othe	2246926.2
	No matches	No matches	No matches	No matches	84.7	DDA for pref	184267711

	Multiple ma	No matches	Multiple ma	No matches	87.4	DDA for pref	147668875
	Multiple ma	No matches	Single match	No matches		No MS2	181562448
	No matches	No matches	No matches	No matches		No MS2	237468467
	No matches	No matches	No matches	No matches		DDA for pref	237258222
	No matches	No matches	No matches	No matches		DDA for pref	154501419
	No matches	No matches	No matches	No matches		DDA for pref	67152109
	No matches	No matches	No matches	No matches		DDA for pref	213278618
	No matches	No matches	No matches	No matches		No MS2	127330314
	No matches	No matches	No matches	No matches		No MS2	98816585
	No matches	No matches	No matches	No matches		DDA for pref	189257422
	No matches	No matches	No matches	No matches		DDA for pref	190226823
	Multiple ma	No matches	No matches	No matches		No MS2	180094639
	No matches	No matches	No matches	No matches		No MS2	234396627
	No matches	No matches	No matches	No matches		No MS2	234169027
	No matches	No matches	Multiple ma	No matches	89.2	DDA for pref	232572656
	No matches	No matches	No matches	No matches		No MS2	200797689
	No matches	No matches	No matches	No matches		No MS2	174832592
	No matches	No matches	No matches	No matches		DDA for pref	213007656
	No matches	No matches	No matches	No matches		No MS2	2931970.2
	No matches	Single match	No matches	Single match		DDA for othe	228386171
	No matches	Single match	No matches	No matches		DDA for pref	169769210
	No matches	No matches	Single match	Single match		DDA for pref	192084490
	No matches	No matches	No matches	No matches		No MS2	227709903
	No matches	No matches	Multiple ma	No matches		No MS2	172493660
	No matches	No matches	Single match	No matches		No MS2	208024267
	No matches	No matches	No matches	No matches		DDA for pref	60869933
	No matches	No matches	No matches	No matches		DDA for pref	170813987
	No matches	No matches	Multiple ma	No matches		DDA for pref	221260111
	No matches	No matches	No matches	No matches		No MS2	191368940
	No matches	No matches	No matches	No matches		No MS2	224301503
	No matches	No matches	No matches	No matches		No MS2	221040952
	No matches	No matches	Single match	No matches		No MS2	190068818
	No matches	No matches	No matches	No matches		No MS2	199090703
	No matches	No matches	No matches	No matches		DDA for pref	61041298
	No matches	No matches	No matches	No matches		No MS2	190414519
	No matches	No matches	No matches	No matches		No MS2	160737918
	No matches	No matches	No matches	No matches		No MS2	118709560
	No matches	No matches	No matches	No matches		DDA for pref	103635501
	Multiple ma	No matches	No matches	No matches		DDA for pref	220295672
	No matches	No matches	No matches	No matches		DDA for pref	54610752
	No matches	No matches	No matches	No matches		No MS2	190139406
	Multiple ma	Single match	No matches	No matches		No MS2	87543920
	Multiple ma	No matches	No matches	No matches		DDA for pref	216969307
	No matches	No matches	Single match	No matches		No MS2	174543756
	No matches	No matches	No matches	No matches		No MS2	132293028
	No matches	No matches	No matches	No matches		DDA for pref	125818420
	No matches	No matches	No matches	No matches		No MS2	205337037
	No matches	No matches	No matches	No matches		No MS2	217308921
	No matches	No matches	No matches	No matches		DDA for pref	109537996
	No matches	No matches	No matches	Single match		DDA for pref	196266036

	No matches	No matches	No matches	No matches		No MS2	212555887
	No matches	No matches	No matches	No matches		No MS2	180739071
	No matches	No matches	No matches	Single match		No MS2	209965280
	No matches	No matches	No matches	No matches		No MS2	213684330
	No matches	No matches	No matches	No matches		DDA for pref	1391968.4
	No matches	No matches	No matches	No matches		No MS2	186134813
	No matches	No matches	No matches	No matches		No MS2	211736310
	No matches	No matches	No matches	No matches		DDA for pref	174485664
	No matches	No matches	Single match	No matches	89.3	DDA for pref	195857095
	No matches	No matches	No matches	Single match		No MS2	182460152
	No matches	No matches	No matches	No matches		DDA for pref	173904217
	No matches	No matches	Single match	No matches		DDA for othe	120809217
	No matches	No matches	No matches	No matches		DDA for pref	126192714
	No matches	No matches	No matches	No matches		No MS2	175257275
	No matches	No matches	No matches	No matches		DDA for pref	108012470
	No matches	No matches	Single match	No matches	95	DDA for pref	191620304
	No matches	No matches	No matches	No matches		No MS2	119879166
	No matches	No matches	No matches	No matches		DDA for pref	112592914
	No matches	No matches	No matches	No matches		No MS2	206313049
	No matches	No matches	No matches	No matches		DDA for othe	175585626
	No matches	No matches	Single match	Multiple ma		No MS2	152194198
	No matches	No matches	No matches	No matches		DDA for pref	196579357
	No matches	No matches	No matches	No matches		DDA for pref	159196105
	No matches	No matches	No matches	No matches		DDA for pref	203471208
	No matches	No matches	No matches	No matches		No MS2	172241978
	No matches	No matches	No matches	No matches		No MS2	172241978
	No matches	No matches	No matches	No matches		DDA for pref	188234665
	No matches	No matches	No matches	No matches		DDA for othe	183100754
	No matches	No matches	No matches	No matches		DDA for pref	157973112
	No matches	No matches	No matches	No matches		No MS2	124125436
	No matches	No matches	No matches	No matches		DDA for pref	4822198.2
	Multiple ma	No matches	No matches	No matches		DDA for pref	94747015
	No matches	No matches	No matches	No matches		No MS2	197192457
	No matches	No matches	Single match	No matches		No MS2	131065694
	No matches	No matches	No matches	No matches		No MS2	117735047
	No matches	No matches	No matches	No matches		No MS2	142917191
	No matches	No matches	Multiple ma	No matches		No MS2	195893026
	No matches	No matches	Single match	No matches		DDA for pref	153718707
	No matches	No matches	No matches	No matches		DDA for pref	194969019
	No matches	No matches	Multiple ma	No matches		No MS2	190972946
	No matches	No matches	No matches	No matches		No MS2	151116241
	No matches	No matches	No matches	No matches		DDA for pref	184500942
	Multiple ma	No matches	No matches	No matches		DDA for othe	159010826
	No matches	No matches	Multiple ma	No matches		No MS2	162831979
	No matches	No matches	Single match	No matches		No MS2	176507445
	No matches	No matches	No matches	No matches		DDA for pref	42178989
	No matches	No matches	No matches	No matches		No MS2	172683709
	No matches	No matches	No matches	No matches		DDA for othe	104335525
	No matches	No matches	No matches	No matches		No MS2	118840915
	No matches	No matches	No matches	No matches		No MS2	130627143

	No matches	No matches	No matches	No matches		DDA for pref	159109539
	No matches	No matches	No matches	No matches		DDA for pref	189967108
	No matches	No matches	Multiple ma	No matches		DDA for othe	143182961
	No matches	No matches	No matches	No matches		DDA for pref	44241453
	Multiple ma	No matches	Multiple ma	No matches		No MS2	189067870
	No matches	No matches	No matches	No matches		No MS2	2560907.2
	Multiple ma	No matches	No matches	No matches		DDA for pref	5372040.2
	No matches	No matches	No matches	No matches		DDA for pref	150700222
	No matches	No matches	No matches	No matches		No MS2	5909890.4
	No matches	No matches	No matches	No matches		No MS2	187797851
	No matches	No matches	No matches	No matches		No MS2	124624451
	No matches	No matches	No matches	No matches		DDA for pref	175936952
	No matches	Multiple ma	No matches	No matches		DDA for pref	140846686
	No matches	No matches	No matches	No matches		No MS2	133555250
	No matches	No matches	No matches	No matches		No MS2	1442930.1
	No matches	No matches	No matches	No matches		No MS2	170591355
	No matches	No matches	No matches	No matches		DDA for pref	182795882
	No matches	No matches	No matches	No matches		DDA for pref	182548302
	No matches	No matches	No matches	No matches		DDA for othe	125242991
	No matches	No matches	No matches	No matches		No MS2	116127274
	Multiple ma	Single match	No matches	No matches		No MS2	152625513
	No matches	No matches	Multiple ma	No matches		DDA for pref	134816007
	No matches	No matches	No matches	No matches		DDA for othe	180566914
	No matches	No matches	Multiple ma	No matches		DDA for pref	149309616
	No matches	No matches	No matches	No matches		No MS2	119771138
	No matches	No matches	No matches	No matches		DDA for pref	179819069
	No matches	No matches	Single match	No matches		DDA for othe	177508026
	No matches	No matches	No matches	No matches		DDA for pref	148602588
	No matches	No matches	Single match	Multiple ma		DDA for pref	130880717
	No matches	No matches	No matches	No matches		No MS2	165809622
	Multiple ma	No matches	No matches	No matches		No MS2	130279188
	No matches	No matches	No matches	No matches		DDA for pref	126416308
	No matches	No matches	No matches	No matches		DDA for pref	178207237
	No matches	No matches	No matches	No matches		No MS2	10986724
	No matches	No matches	No matches	No matches		No MS2	114510636
	Multiple ma	No matches	Single match	No matches		No MS2	118463268
	No matches	No matches	No matches	No matches		No MS2	121925399
	No matches	No matches	Single match	No matches		No MS2	124518703
	No matches	No matches	No matches	No matches		No MS2	170977639
	No matches	No matches	No matches	No matches		No MS2	175217085
	Multiple ma	No matches	No matches	No matches		No MS2	140550095
	No matches	No matches	No matches	No matches		No MS2	140550095
	No matches	No matches	No matches	No matches		No MS2	121116479
	No matches	No matches	Single match	No matches		No MS2	135615256
	No matches	No matches	No matches	No matches		No MS2	96603433
	No matches	No matches	No matches	No matches		No MS2	124034910
	Multiple ma	No matches	No matches	No matches		No MS2	111010138
	No matches	No matches	No matches	No matches		No MS2	172568625
	No matches	No matches	No matches	No matches		DDA for pref	153290915
	No matches	No matches	No matches	No matches		No MS2	123302671

	No matches	No matches	No matches	No matches		No MS2	101976363
	No matches	No matches	No matches	No matches		DDA for pref	135248856
	No matches	No matches	No matches	No matches		No MS2	171044822
	No matches	No matches	Single match	No matches		No MS2	161788380
	No matches	No matches	No matches	No matches		DDA for pref	154991311
	No matches	No matches	No matches	No matches		No MS2	97465067
	No matches	No matches	No matches	No matches		No MS2	170688256
	No matches	No matches	No matches	No matches		No MS2	117145776
	No matches	No matches	No matches	No matches		DDA for pref	164980263
	No matches	No matches	Multiple ma	No matches		No MS2	126754428
	No matches	No matches	No matches	No matches		No MS2	169752782
	No matches	No matches	Multiple ma	Single match		DDA for pref	148679726
	No matches	No matches	No matches	No matches		No MS2	168962224
	No matches	No matches	No matches	No matches		No MS2	106812748
	No matches	No matches	No matches	No matches		DDA for pref	150523133
	No matches	No matches	No matches	No matches		No MS2	167967465
	No matches	No matches	No matches	No matches		DDA for pref	167662199
	Multiple ma	No matches	Multiple ma	No matches		DDA for pref	167432992
	Multiple ma	No matches	No matches	No matches		DDA for pref	14036813
	Multiple ma	No matches	No matches	No matches		No MS2	102005597
	No matches	No matches	No matches	No matches		No MS2	94873891
	No matches	No matches	No matches	No matches		DDA for pref	155738055
	No matches	No matches	No matches	No matches		DDA for othe	124849978
	No matches	No matches	No matches	No matches		DDA for pref	166178630
	No matches	No matches	No matches	No matches		DDA for pref	166138930
	No matches	No matches	No matches	No matches		DDA for pref	1523409.4
	No matches	No matches	No matches	No matches	85.5	DDA for pref	164733306
	No matches	No matches	No matches	No matches		DDA for pref	143841995
	No matches	No matches	No matches	No matches		DDA for othe	164436814
	No matches	No matches	Multiple ma	No matches		No MS2	154016191
	No matches	No matches	Single match	No matches		No MS2	149673416
	No matches	No matches	No matches	No matches		No MS2	74639808
	No matches	No matches	No matches	Single match		No MS2	147933789
	No matches	No matches	No matches	No matches		No MS2	117891107
	No matches	No matches	No matches	No matches		No MS2	119939996
	No matches	No matches	No matches	No matches		DDA for pref	108433733
	No matches	No matches	No matches	No matches		No MS2	98540885
	No matches	No matches	No matches	No matches		No MS2	133598565
	Multiple ma	No matches	No matches	No matches		DDA for pref	161012104
	No matches	No matches	No matches	No matches		No MS2	160907784
	No matches	No matches	No matches	No matches		DDA for pref	160633117
	No matches	No matches	No matches	No matches		DDA for pref	160460662
	Multiple ma	No matches	Multiple ma	No matches		No MS2	160164123
	No matches	No matches	No matches	No matches		No MS2	110095695
	No matches	No matches	No matches	No matches		No MS2	2348174.3
	No matches	Single match	No matches	Single match		DDA for pref	105409102
	No matches	No matches	No matches	No matches		No MS2	144449839
	No matches	No matches	No matches	No matches		No MS2	118769115
	No matches	No matches	No matches	No matches		No MS2	152822240
	No matches	No matches	No matches	No matches		DDA for pref	55977218

	No matches	No matches	Multiple ma	No matches		No MS2	118930438
	No matches	No matches	No matches	No matches		DDA for pref	131219558
	Multiple ma	No matches	Single match	No matches		No MS2	72312173
	No matches	No matches	No matches	No matches		No MS2	156895941
	No matches	No matches	No matches	No matches		DDA for pref	44902517
	No matches	No matches	No matches	No matches		No MS2	156066489
	No matches	No matches	Single match	No matches		DDA for othe	156049378
	No matches	No matches	No matches	No matches		No MS2	100801606
	No matches	Single match	No matches	Single match		No MS2	155331695
	No matches	No matches	No matches	No matches		No MS2	146393293
	No matches	No matches	No matches	No matches		No MS2	99500638
	No matches	Single match	Single match	No matches		No MS2	120735133
	No matches	No matches	Single match	No matches		No MS2	154458512
	No matches	No matches	No matches	No matches		No MS2	94533962
	No matches	No matches	Single match	No matches		DDA for pref	99080068
	No matches	No matches	No matches	No matches		DDA for pref	74291022
	No matches	No matches	No matches	No matches		No MS2	132233576
	No matches	No matches	No matches	No matches		No MS2	91416047
	No matches	No matches	Single match	No matches		No MS2	147447666
	No matches	No matches	No matches	No matches		No MS2	108581258
	No matches	No matches	No matches	No matches		DDA for pref	95550085
	No matches	No matches	No matches	No matches		DDA for pref	151805202
	No matches	No matches	No matches	No matches		No MS2	151613288
	No matches	No matches	No matches	No matches		DDA for pref	151369636
	No matches	No matches	No matches	No matches		No MS2	151347811
	Multiple ma	No matches	No matches	No matches		DDA for pref	14328158
	Multiple ma	No matches	No matches	No matches		DDA for pref	151149453
	No matches	No matches	No matches	Single match		No MS2	151116119
	Multiple ma	No matches	Multiple ma	No matches		DDA for pref	81678871
	No matches	No matches	No matches	No matches		No MS2	125503567
	Multiple ma	No matches	No matches	No matches		No MS2	103035702
	No matches	No matches	No matches	No matches		No MS2	71386260
	No matches	No matches	No matches	No matches		No MS2	33080386
	No matches	No matches	No matches	No matches		DDA for othe	116635122
	No matches	No matches	No matches	No matches		No MS2	58958689
	No matches	No matches	No matches	No matches		DDA for pref	129362297
	Multiple ma	Single match	Single match	No matches		No MS2	119769415
	No matches	No matches	No matches	No matches		No MS2	2088028.7
	No matches	No matches	No matches	No matches		No MS2	148300876
	No matches	No matches	No matches	No matches		No MS2	148235599
	No matches	No matches	No matches	No matches		DDA for pref	136011467
	No matches	No matches	No matches	No matches		DDA for pref	76636863
	No matches	No matches	No matches	No matches		No MS2	106289348
	No matches	No matches	No matches	No matches		No MS2	136179762
	No matches	No matches	No matches	No matches		No MS2	147171584
	No matches	No matches	No matches	No matches		No MS2	111110234
	Multiple ma	Single match	No matches	No matches		DDA for pref	70800736
	No matches	Single match	No matches	No matches		No MS2	147128635
	Multiple ma	No matches	Multiple ma	No matches		DDA for othe	122635606
	No matches	No matches	Single match	No matches		No MS2	141051200

	No matches	No matches	No matches	No matches		No MS2	108607433
	Multiple ma	No matches	No matches	No matches		No MS2	135787624
	No matches	No matches	No matches	No matches		DDA for pref	83478510
	No matches	No matches	No matches	No matches		No MS2	146047778
	No matches	No matches	Multiple ma	Multiple ma		No MS2	145848623
	No matches	No matches	No matches	No matches		No MS2	145764514
	No matches	No matches	No matches	No matches		DDA for pref	95721261
	No matches	No matches	No matches	No matches		No MS2	145212358
	No matches	No matches	No matches	No matches		DDA for pref	130611432
	No matches	No matches	Single match	No matches		No MS2	118165545
	No matches	No matches	Single match	No matches		No MS2	142486806
	No matches	No matches	No matches	No matches		No MS2	58464034
	No matches	No matches	No matches	Single match		No MS2	132196611
	Multiple ma	No matches	No matches	No matches		No MS2	111264210
	Multiple ma	No matches	Multiple ma	No matches		No MS2	90719567
	No matches	No matches	No matches	No matches		No MS2	1434011
	No matches	No matches	No matches	No matches		No MS2	13021784
	No matches	No matches	No matches	No matches		DDA for pref	86736136
	No matches	No matches	Multiple ma	No matches		DDA for pref	129102016
	No matches	No matches	No matches	No matches		No MS2	42237315
	No matches	No matches	No matches	No matches		No MS2	89100798
	No matches	No matches	Multiple ma	Multiple ma		No MS2	96135847
	No matches	No matches	No matches	No matches		No MS2	99173265
	No matches	No matches	No matches	No matches		DDA for pref	102302715
	No matches	No matches	No matches	No matches		No MS2	139738571
	No matches	No matches	No matches	No matches		No MS2	139441477
	No matches	No matches	No matches	No matches		DDA for pref	103540192
	No matches	No matches	No matches	No matches		No MS2	139404999
	Multiple ma	No matches	No matches	No matches		DDA for othe	99465821
	No matches	No matches	No matches	No matches		DDA for pref	110519537
	No matches	No matches	No matches	No matches		DDA for pref	102302715
	No matches	No matches	No matches	No matches		No MS2	96839665
	No matches	No matches	No matches	No matches		No MS2	90636502
	No matches	No matches	No matches	No matches		No MS2	138443137
	No matches	No matches	No matches	Single match		DDA for othe	138202883
	No matches	No matches	No matches	No matches		No MS2	122486378
	No matches	No matches	Single match	No matches		No MS2	88119749
	No matches	No matches	No matches	No matches		DDA for pref	137421689
	No matches	No matches	No matches	No matches		No MS2	137261602
	No matches	No matches	No matches	No matches		DDA for pref	106202987
	No matches	No matches	Single match	No matches		DDA for pref	44497062
	No matches	No matches	No matches	No matches		No MS2	42453814
	No matches	No matches	No matches	No matches		No MS2	136777520
	No matches	No matches	No matches	No matches		DDA for pref	136562222
	No matches	No matches	Multiple ma	No matches		DDA for othe	136325653
	No matches	No matches	No matches	No matches		No MS2	130709947
	Multiple ma	Single match	No matches	No matches		DDA for pref	135534796
	No matches	No matches	No matches	No matches		No MS2	107318115
	No matches	No matches	No matches	No matches		No MS2	82160415
	No matches	No matches	No matches	No matches		No MS2	5409160.2

	No matches	No matches	No matches	No matches		No MS2	114850862
	No matches	No matches	No matches	No matches		No MS2	133637964
	No matches	No matches	No matches	No matches		No MS2	77638182
	No matches	No matches	No matches	No matches		No MS2	3792674.7
	No matches	No matches	No matches	No matches		No MS2	101475696
	No matches	No matches	No matches	No matches		No MS2	122046394
	No matches	Multiple ma	No matches	No matches		DDA for pref	133146236
	No matches	No matches	No matches	No matches		No MS2	107448440
	No matches	No matches	No matches	No matches		No MS2	132943961
	No matches	No matches	No matches	No matches		No MS2	6514653.6
	No matches	No matches	Single match	No matches		DDA for othe	132603054
	No matches	No matches	No matches	No matches		No MS2	132319674
	No matches	No matches	No matches	No matches		DDA for pref	116931400
	No matches	No matches	No matches	No matches		No MS2	82931164
	No matches	No matches	No matches	No matches		No MS2	84525876
	No matches	No matches	No matches	No matches		No MS2	97032304
	No matches	No matches	Single match	Single match		No MS2	113095020
	No matches	No matches	No matches	No matches		DDA for pref	130766405
	No matches	No matches	No matches	No matches		No MS2	49822506
	No matches	No matches	No matches	No matches		No MS2	66614128
	No matches	No matches	Single match	No matches		No MS2	96521498
	No matches	No matches	Multiple ma	No matches		No MS2	129616400
	No matches	No matches	Single match	No matches		DDA for pref	120268212
	No matches	No matches	No matches	No matches		No MS2	4337607
	No matches	No matches	No matches	No matches		No MS2	97077090
	No matches	No matches	Single match	No matches		No MS2	113935028
	No matches	No matches	No matches	No matches		DDA for othe	105857363
	No matches	No matches	No matches	No matches		No MS2	57198978
	No matches	No matches	No matches	No matches		DDA for pref	119167230
	No matches	Multiple ma	No matches	Multiple ma		No MS2	106972284
	No matches	No matches	No matches	No matches		No MS2	118377268
	No matches	No matches	No matches	No matches		DDA for pref	34894021
	No matches	No matches	No matches	No matches		No MS2	49485462
	No matches	No matches	No matches	No matches		DDA for pref	128097714
	No matches	No matches	No matches	No matches		No MS2	39800536
	Multiple ma	No matches	No matches	No matches		DDA for pref	127533816
	No matches	No matches	No matches	No matches		No MS2	60160381
	No matches	No matches	Single match	No matches		DDA for pref	114692875
	No matches	No matches	No matches	No matches		No MS2	78110822
	No matches	No matches	No matches	No matches		No MS2	110759493
	Multiple ma	No matches	No matches	No matches		No MS2	95256245
	No matches	No matches	No matches	No matches		No MS2	19240814
	No matches	No matches	Single match	Multiple ma		DDA for pref	126570379
	Multiple ma	Single match	Multiple ma	No matches		No MS2	102075116
	No matches	No matches	No matches	No matches		DDA for othe	80810496
	Multiple ma	No matches	No matches	No matches		No MS2	100425579
	No matches	No matches	No matches	No matches		No MS2	88380829
	No matches	No matches	Single match	Single match		DDA for pref	29021619
	No matches	No matches	No matches	No matches		No MS2	2356319.7
	No matches	No matches	Single match	No matches		No MS2	107038855

	No matches	No matches	No matches	No matches		No MS2	3512894.6
	No matches	Single match	Multiple ma	No matches		No MS2	125107753
	No matches	No matches	No matches	No matches		DDA for pref	8186905.9
	No matches	No matches	No matches	No matches		No MS2	124033679
	No matches	No matches	No matches	No matches		DDA for pref	52505280
	No matches	No matches	No matches	No matches		DDA for pref	124237525
	Multiple ma	No matches	Single match	Single match		No MS2	109468357
	No matches	No matches	No matches	No matches		DDA for pref	73331902
	No matches	No matches	No matches	No matches		DDA for pref	86511589
	No matches	No matches	No matches	No matches		No MS2	91196430
	No matches	No matches	No matches	No matches		No MS2	123080484
	No matches	No matches	No matches	No matches		No MS2	892694.53
	No matches	No matches	No matches	No matches		No MS2	122888966
	No matches	No matches	No matches	No matches	82.2	DDA for pref	1040181.8
	No matches	No matches	No matches	No matches		No MS2	18539564
	No matches	No matches	Single match	No matches		No MS2	122441530
	Multiple ma	No matches	Multiple ma	No matches		No MS2	72036162
	No matches	Single match	Multiple ma	No matches		No MS2	100358858
	No matches	No matches	No matches	No matches		No MS2	1172558.7
	No matches	Single match	No matches	No matches		No MS2	121294127
	No matches	No matches	No matches	No matches		No MS2	121253898
	Multiple ma	No matches	No matches	No matches		No MS2	84822347
	No matches	No matches	Single match	No matches		No MS2	91162084
	No matches	No matches	Multiple ma	No matches		DDA for pref	108423298
	No matches	Single match	No matches	Multiple ma		DDA for pref	120423424
	No matches	No matches	Multiple ma	Single match		No MS2	113036506
	No matches	No matches	No matches	No matches		No MS2	104097888
	No matches	No matches	No matches	No matches		No MS2	89961920
	No matches	No matches	No matches	No matches		No MS2	109021434
	No matches	Single match	No matches	No matches		No MS2	98812313
	No matches	No matches	No matches	No matches		DDA for pref	105490561
	No matches	No matches	No matches	No matches		No MS2	60051057
	No matches	No matches	No matches	No matches		No MS2	82929030
	No matches	No matches	No matches	No matches		No MS2	116424558
	Multiple ma	No matches	Single match	No matches		No MS2	83854899
	No matches	No matches	No matches	No matches		DDA for pref	92370139
	No matches	No matches	Multiple ma	Single match		No MS2	95428356
	Multiple ma	No matches	Single match	No matches		DDA for pref	74828359
	No matches	No matches	No matches	No matches		DDA for pref	92792631
	No matches	No matches	No matches	No matches		DDA for pref	72280967
	No matches	No matches	No matches	No matches		No MS2	82076214
	No matches	No matches	No matches	No matches		No MS2	88403810
	No matches	No matches	No matches	No matches		No MS2	102992666
	Multiple ma	No matches	No matches	No matches		No MS2	116583950
	No matches	No matches	No matches	No matches		No MS2	92440473
	No matches	No matches	No matches	No matches		DDA for othe	38034093
	No matches	No matches	Single match	No matches		No MS2	115808705
	No matches	No matches	No matches	No matches		No MS2	11625209
	No matches	No matches	No matches	No matches		No MS2	42642201
	No matches	No matches	No matches	No matches		DDA for pref	115331474

	No matches	No matches	No matches	No matches		DDA for pref	84320829
	No matches	No matches	No matches	No matches		DDA for pref	90579641
	No matches	No matches	No matches	No matches		No MS2	57594192
	Single match	No matches	No matches	No matches		DDA for pref	106013392
	No matches	No matches	No matches	No matches		No MS2	94875050
	No matches	No matches	No matches	No matches		No MS2	104901944
	No matches	No matches	No matches	No matches		DDA for pref	91284649
	No matches	No matches	No matches	No matches		No MS2	68912888
	No matches	No matches	No matches	No matches		No MS2	88283634
	No matches	No matches	No matches	No matches		DDA for pref	114169780
	No matches	No matches	No matches	No matches		No MS2	114140815
	No matches	No matches	No matches	No matches		DDA for pref	79162276
	No matches	No matches	No matches	No matches		DDA for pref	100842696
	No matches	No matches	No matches	No matches		No MS2	80040423
	No matches	No matches	No matches	No matches		No MS2	95298424
	Multiple ma	No matches	No matches	No matches		No MS2	66138313
	No matches	No matches	No matches	Single match		No MS2	105654221
	No matches	No matches	No matches	No matches		No MS2	113005760
	No matches	No matches	Multiple ma	No matches		No MS2	74951638
	No matches	No matches	No matches	No matches		No MS2	65867410
	No matches	No matches	No matches	No matches		DDA for pref	81919970
	No matches	No matches	No matches	No matches		No MS2	112664710
	No matches	No matches	Multiple ma	No matches		No MS2	112613075
	No matches	No matches	No matches	No matches		DDA for pref	34117020
	No matches	No matches	No matches	No matches		No MS2	1131937
	No matches	No matches	No matches	No matches		DDA for pref	65365796
	No matches	No matches	No matches	No matches		DDA for pref	86885508
	Multiple ma	No matches	No matches	No matches		No MS2	103507431
	No matches	No matches	No matches	No matches		DDA for pref	111865131
	No matches	No matches	No matches	No matches		No MS2	72750400
	No matches	No matches	No matches	Multiple ma		DDA for pref	72150647
	No matches	No matches	Single match	No matches		No MS2	93493945
	No matches	No matches	Multiple ma	No matches		No MS2	111198403
	No matches	No matches	No matches	No matches		No MS2	68873833
	No matches	No matches	No matches	No matches		No MS2	94755463
	Multiple ma	Single match	Multiple ma	No matches	94	DDA for pref	97548819
	No matches	No matches	Multiple ma	No matches		No MS2	95096813
	No matches	No matches	No matches	No matches		No MS2	110446065
	No matches	No matches	No matches	No matches		No MS2	79948243
	No matches	No matches	No matches	No matches		No MS2	106265763
	No matches	No matches	No matches	No matches		No MS2	24026463
	No matches	No matches	No matches	No matches		No MS2	37503895
	No matches	No matches	No matches	No matches		DDA for pref	48438479
	No matches	No matches	No matches	No matches		DDA for pref	109075824
	No matches	No matches	No matches	No matches		No MS2	35505095
	No matches	No matches	No matches	No matches		DDA for pref	46477194
	No matches	No matches	No matches	No matches		No MS2	108599690
	No matches	No matches	Single match	No matches		DDA for othe	94476264
	No matches	No matches	No matches	No matches		No MS2	108453237
	No matches	No matches	Multiple ma	No matches		No MS2	83480921

	No matches	No matches	No matches	No matches		No MS2	69399963
	No matches	No matches	No matches	No matches		No MS2	1429031.8
	No matches	No matches	No matches	No matches		DDA for pref	32416989
	No matches	No matches	No matches	No matches		No MS2	86348184
	No matches	No matches	Single match	No matches		No MS2	107767731
	No matches	No matches	No matches	No matches		No MS2	107700522
	No matches	No matches	No matches	No matches		No MS2	72378541
	No matches	No matches	Single match	No matches		No MS2	18651219
	No matches	No matches	No matches	No matches		No MS2	73209254
	No matches	No matches	Single match	No matches		No MS2	95438350
	No matches	No matches	No matches	No matches		DDA for pref	510855.18
	Multiple ma	Single match	No matches	No matches		DDA for pref	81433311
	No matches	No matches	No matches	No matches		No MS2	106833400
	No matches	No matches	No matches	No matches		No MS2	106818465
	No matches	No matches	Single match	No matches		No MS2	69047277
	No matches	No matches	No matches	No matches		No MS2	106527423
	Multiple ma	No matches	No matches	No matches		DDA for pref	85230727
	No matches	No matches	Multiple ma	No matches		No MS2	100351039
	No matches	No matches	No matches	No matches		No MS2	106258665
	No matches	No matches	No matches	No matches		No MS2	78160770
	No matches	No matches	No matches	No matches		DDA for pref	83153110
	No matches	No matches	No matches	No matches		No MS2	2490936.1
	No matches	No matches	No matches	No matches		No MS2	105807912
	No matches	No matches	No matches	No matches		No MS2	17982427
	No matches	No matches	No matches	No matches		No MS2	31998041
	No matches	No matches	No matches	No matches		DDA for pref	38658204
	No matches	No matches	No matches	No matches		No MS2	43500930
	No matches	No matches	No matches	No matches		No MS2	45735158
	No matches	No matches	No matches	No matches		DDA for othe	25808856
	No matches	No matches	No matches	No matches		No MS2	70196869
	No matches	No matches	Single match	No matches		DDA for pref	90601183
	No matches	No matches	No matches	No matches		No MS2	91447644
	Multiple ma	No matches	No matches	No matches		DDA for pref	72951630
	No matches	No matches	No matches	Single match		No MS2	79285615
	No matches	No matches	No matches	No matches		No MS2	101306598
	No matches	No matches	No matches	No matches	83.1	DDA for pref	60886499
	No matches	No matches	No matches	No matches		DDA for othe	76929555
	No matches	Single match	No matches	No matches		No MS2	78975646
	No matches	No matches	No matches	No matches		No MS2	77531643
	No matches	No matches	No matches	No matches		No MS2	103628077
	No matches	No matches	Single match	No matches		No MS2	75700349
	No matches	No matches	No matches	No matches		No MS2	102896781
	No matches	Single match	No matches	No matches		DDA for pref	6739010.1
	No matches	No matches	No matches	No matches		No MS2	74207517
	No matches	No matches	No matches	No matches		No MS2	86921035
	No matches	No matches	No matches	No matches		DDA for pref	56075380
	No matches	No matches	No matches	No matches		No MS2	102726597
	No matches	No matches	No matches	No matches		DDA for pref	102379306
	No matches	No matches	No matches	Single match		No MS2	102368981
	No matches	No matches	No matches	No matches		No MS2	7673843.1

	No matches	No matches	No matches	No matches		DDA for othe	67585675
	No matches	No matches	No matches	No matches		No MS2	46867002
	No matches	No matches	No matches	No matches		DDA for pref	87524054
	Multiple ma	Single match	No matches	No matches		No MS2	101645762
	No matches	No matches	No matches	No matches		No MS2	66285673
	No matches	No matches	Single match	No matches		DDA for pref	20108625
	Multiple ma	No matches	Multiple ma	No matches		No MS2	65253561
	No matches	No matches	No matches	No matches		No MS2	46628236
	No matches	No matches	No matches	No matches		No MS2	101378639
	No matches	No matches	Multiple ma	No matches		DDA for pref	55203941
	Multiple ma	No matches	No matches	No matches		No MS2	76501235
	No matches	No matches	No matches	No matches		DDA for pref	76275164
	No matches	No matches	No matches	No matches		No MS2	100233329
	No matches	No matches	Multiple ma	No matches		DDA for pref	89912108
	No matches	No matches	No matches	No matches		No MS2	100111775
	No matches	No matches	No matches	No matches		No MS2	41031846
	No matches	No matches	No matches	No matches		No MS2	78815562
	No matches	No matches	No matches	No matches		No MS2	99771492
	No matches	No matches	No matches	No matches		No MS2	84484422
	No matches	No matches	No matches	No matches		No MS2	99670621
	Multiple ma	No matches	No matches	No matches		DDA for pref	78429959
	No matches	No matches	No matches	No matches		No MS2	50541521
	No matches	No matches	No matches	No matches		No MS2	52133832
	No matches	No matches	No matches	No matches		No MS2	17293109
	No matches	No matches	No matches	No matches		DDA for pref	2260855.4
	No matches	No matches	No matches	No matches		No MS2	89558967
	No matches	Single match	Single match	No matches		No MS2	67381452
	No matches	No matches	No matches	No matches		No MS2	85520554
	No matches	No matches	No matches	No matches		No MS2	98980711
	No matches	No matches	No matches	No matches		DDA for pref	438587.09
	No matches	No matches	No matches	No matches		DDA for pref	66382500
	No matches	No matches	No matches	No matches		DDA for pref	73028862
	No matches	No matches	No matches	No matches		DDA for pref	44441021
	No matches	No matches	No matches	No matches		DDA for pref	88909450
	No matches	No matches	No matches	No matches		No MS2	61059064
	No matches	No matches	No matches	No matches		No MS2	7545190.7
	No matches	No matches	No matches	No matches		No MS2	92754724
	Multiple ma	No matches	No matches	No matches		DDA for pref	98074566
	No matches	No matches	No matches	No matches		DDA for othe	71282529
	No matches	Single match	No matches	No matches		No MS2	56020598
	No matches	No matches	No matches	No matches		No MS2	53790159
	Multiple ma	No matches	No matches	No matches		DDA for othe	54892800
	No matches	No matches	Single match	No matches		No MS2	66814234
	No matches	No matches	No matches	No matches		No MS2	97695452
	No matches	Single match	Single match	No matches		No MS2	97507499
	No matches	No matches	No matches	No matches		No MS2	55574686
	Multiple ma	No matches	No matches	No matches		No MS2	77075984
	No matches	No matches	No matches	No matches		No MS2	82185325
	No matches	No matches	No matches	No matches		DDA for pref	47436946
	Multiple ma	No matches	No matches	No matches		DDA for othe	97136445

	No matches	No matches	No matches	No matches		DDA for pref	50457058
	No matches	No matches	No matches	No matches		No MS2	69042046
	No matches	No matches	Multiple ma	No matches		No MS2	87860924
	No matches	No matches	No matches	No matches		No MS2	96376265
	No matches	No matches	No matches	No matches		No MS2	1787500.3
	No matches	No matches	No matches	No matches		No MS2	48030648
	No matches	No matches	No matches	No matches		DDA for pref	96164419
	Single match	No matches	No matches	No matches		No MS2	71944402
	No matches	No matches	No matches	No matches		No MS2	59345470
	No matches	No matches	No matches	No matches		No MS2	57505388
	No matches	No matches	No matches	No matches		No MS2	57973210
	No matches	No matches	Multiple ma	No matches		No MS2	76804909
	No matches	No matches	No matches	No matches		No MS2	6492815.2
	No matches	No matches	No matches	No matches	86.5	DDA for pref	2528073.7
	No matches	No matches	No matches	No matches		No MS2	44784126
	No matches	No matches	No matches	No matches		DDA for pref	94651968
	No matches	No matches	No matches	No matches		No MS2	86095677
	No matches	No matches	No matches	No matches		DDA for othe	74722924
	No matches	No matches	No matches	No matches		DDA for pref	88604043
	No matches	No matches	No matches	No matches		No MS2	29695317
	No matches	No matches	No matches	No matches		No MS2	74167422
	No matches	No matches	No matches	No matches		DDA for pref	45744679
	No matches	No matches	No matches	No matches		No MS2	93872546
	No matches	No matches	No matches	No matches		No MS2	1453564.6
	No matches	No matches	No matches	No matches		No MS2	72524612
	No matches	No matches	No matches	Multiple ma		No MS2	93501929
	Multiple ma	No matches	Multiple ma	No matches		No MS2	57389586
	No matches	No matches	Single match	No matches		No MS2	87028738
	Single match	No matches	No matches	No matches		No MS2	92937457
	No matches	No matches	Single match	No matches		No MS2	92823899
	No matches	No matches	No matches	No matches		No MS2	69342598
	No matches	No matches	No matches	No matches	93.3	DDA for pref	85944438
	No matches	No matches	No matches	No matches		No MS2	55414189
	No matches	No matches	No matches	No matches		No MS2	81561115
	No matches	No matches	No matches	No matches		DDA for pref	60270741
	No matches	No matches	No matches	No matches		DDA for pref	28411653
	No matches	No matches	No matches	No matches		DDA for othe	76308482
	No matches	No matches	No matches	No matches		No MS2	66677877
	No matches	No matches	No matches	No matches		No MS2	66189735
	No matches	No matches	No matches	No matches		No MS2	92051152
	No matches	No matches	No matches	No matches		DDA for pref	12643949
	Multiple ma	No matches	Multiple ma	No matches		DDA for pref	79290636
	No matches	No matches	No matches	No matches		DDA for pref	79361303
	No matches	No matches	No matches	No matches		DDA for othe	79580611
	No matches	No matches	No matches	No matches		DDA for pref	69354529
	No matches	No matches	No matches	No matches		No MS2	91425247
	No matches	No matches	No matches	No matches		DDA for pref	72620020
	No matches	No matches	No matches	No matches		DDA for pref	90653516
	No matches	No matches	Multiple ma	No matches		No MS2	76044234
	No matches	No matches	No matches	No matches		DDA for pref	41537877

	No matches	No matches	No matches	No matches		No MS2	81392800
	No matches	No matches	No matches	No matches		No MS2	56587407
	No matches	No matches	No matches	No matches		No MS2	1285228.8
	No matches	No matches	No matches	No matches		No MS2	51456560
	No matches	No matches	Multiple ma	No matches		No MS2	90686534
	No matches	No matches	No matches	No matches		No MS2	1115105.1
	No matches	No matches	No matches	No matches		No MS2	65028085
	No matches	No matches	No matches	No matches		No MS2	90262199
	No matches	No matches	No matches	Single match		No MS2	73032759
	No matches	No matches	No matches	No matches		No MS2	22187530
	Single match	No matches	Multiple ma	No matches		DDA for pref	67986719
	No matches	No matches	No matches	No matches		No MS2	33740654
	No matches	No matches	No matches	No matches		DDA for pref	76771158
	No matches	No matches	Multiple ma	No matches		DDA for pref	70919919
	No matches	No matches	No matches	Single match		No MS2	64864587
	No matches	No matches	Single match	No matches		No MS2	70431865
	No matches	No matches	No matches	No matches		No MS2	40706519
	Multiple ma	No matches	Multiple ma	No matches		No MS2	51215158
	No matches	No matches	No matches	No matches		DDA for pref	68787564
	No matches	No matches	No matches	No matches		No MS2	88715961
	No matches	No matches	No matches	No matches		No MS2	59813103
	No matches	No matches	No matches	No matches		No MS2	66606924
	No matches	No matches	No matches	No matches		No MS2	38613204
	No matches	No matches	No matches	No matches		No MS2	81569924
	No matches	No matches	No matches	No matches		No MS2	39083846
	No matches	No matches	Single match	No matches		DDA for othe	76629904
	No matches	No matches	No matches	No matches		No MS2	87474289
	No matches	No matches	No matches	No matches		No MS2	57830425
	No matches	No matches	No matches	No matches		No MS2	76401516
	No matches	No matches	No matches	No matches		No MS2	48487668
	No matches	No matches	No matches	No matches		No MS2	75107462
	No matches	No matches	No matches	No matches		DDA for pref	82721300
	No matches	No matches	No matches	No matches		DDA for pref	86702552
	No matches	No matches	No matches	No matches		No MS2	78582803
	No matches	No matches	No matches	Single match		No MS2	44249692
	No matches	No matches	Multiple ma	No matches		No MS2	62615997
	No matches	No matches	No matches	No matches		No MS2	57600407
	Single match	No matches	No matches	No matches		No MS2	55463043
	No matches	No matches	No matches	No matches		No MS2	54349005
	No matches	No matches	No matches	No matches		No MS2	83762870
	No matches	No matches	Multiple ma	Single match		No MS2	36193986
	No matches	No matches	No matches	No matches		No MS2	86180436
	No matches	No matches	No matches	No matches		DDA for othe	76273259
	No matches	No matches	No matches	No matches		No MS2	86071281
	No matches	No matches	No matches	No matches		No MS2	77332419
	No matches	No matches	No matches	No matches		DDA for pref	74989864
	No matches	No matches	No matches	No matches		No MS2	73941123
	No matches	No matches	Multiple ma	No matches		No MS2	55197778
	No matches	No matches	No matches	No matches		No MS2	76664265
	No matches	No matches	Single match	No matches		DDA for pref	32037561

	No matches	No matches	No matches	No matches		DDA for pref	60390435
	No matches	No matches	No matches	No matches		No MS2	4926983
	No matches	No matches	Single match	No matches		No MS2	56352391
	No matches	No matches	Multiple ma	No matches		No MS2	66235512
	No matches	No matches	No matches	No matches		No MS2	49849634
	No matches	No matches	No matches	No matches		No MS2	81888581
	No matches	No matches	No matches	No matches		DDA for pref	84612971
	No matches	No matches	Multiple ma	No matches		No MS2	84760314
	No matches	No matches	No matches	No matches		No MS2	3989485
	No matches	No matches	No matches	No matches		No MS2	1178458.5
	No matches	No matches	No matches	No matches		No MS2	11876331
	No matches	No matches	No matches	No matches		DDA for pref	73969848
	No matches	No matches	No matches	No matches		DDA for pref	47137082
	No matches	No matches	No matches	No matches		DDA for pref	74693905
	No matches	No matches	No matches	No matches		No MS2	66569402
	No matches	No matches	No matches	No matches		DDA for pref	68610726
	No matches	No matches	No matches	No matches		No MS2	83820373
	No matches	No matches	No matches	No matches		No MS2	28101645
	No matches	No matches	No matches	No matches		DDA for pref	68665666
	No matches	No matches	No matches	No matches		No MS2	60457808
	No matches	No matches	No matches	No matches		No MS2	57777058
	No matches	No matches	No matches	No matches		No MS2	69680422
	No matches	Multiple ma	No matches	No matches		No MS2	27700016
	No matches	No matches	No matches	No matches		DDA for pref	38598403
	No matches	No matches	No matches	No matches		DDA for othe	71322724
	No matches	No matches	Single match	No matches		No MS2	60994671
	No matches	No matches	No matches	No matches		No MS2	56174087
	No matches	No matches	No matches	No matches		No MS2	1667082.6
	No matches	No matches	No matches	No matches		No MS2	49270828
	No matches	No matches	No matches	No matches		DDA for othe	62719014
	No matches	No matches	No matches	No matches		No MS2	1289393.3
	No matches	No matches	No matches	No matches		DDA for pref	80401893
	No matches	No matches	No matches	No matches		No MS2	82390636
	No matches	No matches	No matches	No matches		No MS2	960007.6
	No matches	No matches	Single match	No matches		No MS2	63933326
	No matches	No matches	No matches	No matches		No MS2	58547407
	No matches	Single match	No matches	No matches		DDA for pref	70510545
	No matches	No matches	No matches	No matches		No MS2	69870962
	No matches	No matches	No matches	No matches		DDA for pref	3489843
	No matches	No matches	No matches	No matches		No MS2	60396837
	No matches	No matches	No matches	No matches		DDA for othe	955017.75
	No matches	No matches	No matches	No matches		No MS2	54578188
	No matches	No matches	No matches	No matches		No MS2	38329432
	Multiple ma	No matches	No matches	No matches		No MS2	35495200
	No matches	No matches	Multiple ma	Multiple ma		No MS2	78877304
	No matches	No matches	No matches	No matches		No MS2	70523379
	No matches	No matches	No matches	No matches		No MS2	80881739
	No matches	No matches	No matches	No matches		No MS2	78220437
	Single match	No matches	Single match	Single match		No MS2	55476084
	No matches	No matches	No matches	No matches		No MS2	62430925

	Multiple ma	No matches	Single match	No matches		No MS2	69698288
	No matches	No matches	No matches	No matches		No MS2	31338212
	No matches	No matches	No matches	No matches		No MS2	26585146
	Multiple ma	No matches	No matches	No matches		No MS2	77291919
	No matches	No matches	No matches	No matches		No MS2	58493126
	No matches	No matches	No matches	No matches		No MS2	80046350
	Multiple ma	No matches	No matches	No matches		No MS2	67170450
	No matches	No matches	No matches	No matches		No MS2	62521885
	No matches	No matches	No matches	No matches		No MS2	67643346
	No matches	No matches	No matches	No matches		No MS2	62186564
	No matches	No matches	Multiple ma	No matches		DDA for pref	47582646
	No matches	No matches	Single match	No matches		DDA for pref	77367719
	No matches	Single match	No matches	No matches		No MS2	79621407
	Multiple ma	Single match	No matches	No matches		No MS2	56953652
	No matches	No matches	No matches	No matches		No MS2	3408476.3
	No matches	No matches	No matches	No matches		No MS2	57627483
	No matches	No matches	No matches	No matches		No MS2	79457065
	No matches	No matches	Single match	No matches		No MS2	79401249
	No matches	No matches	Single match	No matches		No MS2	53844674
	No matches	No matches	No matches	No matches		No MS2	65235144
	No matches	No matches	No matches	Single match		No MS2	60333172
	No matches	No matches	Single match	No matches		No MS2	51681087
	No matches	No matches	No matches	No matches		No MS2	43486436
	Single match	Single match	No matches	Single match		No MS2	58098940
	No matches	No matches	No matches	No matches		No MS2	60822371
	No matches	No matches	No matches	No matches		No MS2	53836735
	No matches	No matches	No matches	No matches		No MS2	51605263
	No matches	No matches	No matches	No matches		No MS2	69623575
	No matches	No matches	No matches	No matches		No MS2	2444610.5
	No matches	Single match	Single match	No matches		No MS2	78482969
	No matches	No matches	No matches	No matches		No MS2	54466523
	No matches	No matches	No matches	No matches		No MS2	61222635
	No matches	No matches	No matches	No matches		No MS2	48354593
	No matches	No matches	No matches	No matches		No MS2	59893513
	No matches	No matches	No matches	No matches		No MS2	45033300
	No matches	No matches	No matches	No matches		No MS2	15558351
	No matches	No matches	No matches	No matches		No MS2	19711464
	No matches	No matches	No matches	No matches		No MS2	50061466
	No matches	No matches	No matches	No matches		No MS2	2100727.6
	No matches	No matches	No matches	No matches		DDA for pref	55898132
	No matches	No matches	No matches	No matches		No MS2	77342003
	No matches	Multiple ma	No matches	No matches		No MS2	48240190
	No matches	No matches	No matches	No matches		No MS2	56308941
	No matches	No matches	No matches	No matches		DDA for pref	52689640
	No matches	No matches	No matches	No matches		No MS2	71078742
	No matches	No matches	No matches	No matches		No MS2	1321798.9
	No matches	No matches	No matches	No matches		No MS2	10062863
	No matches	No matches	No matches	No matches		No MS2	53793168
	No matches	No matches	No matches	No matches		No MS2	76534462
	No matches	No matches	No matches	No matches		DDA for pref	61167543

	No matches	No matches	No matches	No matches		No MS2	40919850
	No matches	No matches	No matches	No matches		No MS2	40792047
	No matches	Single match	Single match	No matches		No MS2	45934720
	No matches	No matches	No matches	No matches		No MS2	56072259
	No matches	No matches	No matches	No matches		No MS2	75805422
	No matches	No matches	No matches	No matches		DDA for pref	75782854
	No matches	No matches	No matches	No matches		No MS2	75749606
	No matches	No matches	No matches	No matches		DDA for pref	71455268
	No matches	No matches	Multiple ma	No matches		No MS2	54491046
	No matches	No matches	No matches	No matches		No MS2	41525569
	No matches	No matches	No matches	No matches		DDA for pref	47580163
	No matches	No matches	No matches	No matches		DDA for othe	55121306
	No matches	No matches	No matches	No matches		No MS2	48581722
	No matches	No matches	No matches	No matches		DDA for pref	73709902
	No matches	No matches	Multiple ma	No matches		No MS2	74965280
	No matches	No matches	Multiple ma	No matches		No MS2	74953897
	No matches	Single match	No matches	No matches		DDA for pref	74776509
	No matches	No matches	No matches	No matches		No MS2	74608489
	No matches	No matches	No matches	No matches		No MS2	74603556
	No matches	No matches	No matches	No matches		No MS2	40535675
	No matches	No matches	No matches	No matches		No MS2	53057041
	No matches	Single match	Single match	No matches		No MS2	74337939
	No matches	No matches	No matches	No matches		No MS2	70450351
	No matches	No matches	Single match	No matches		No MS2	17298002
	No matches	No matches	No matches	No matches		No MS2	43452332
	No matches	No matches	No matches	No matches		No MS2	1321937.2
	No matches	No matches	No matches	No matches		DDA for pref	39359633
	No matches	No matches	No matches	No matches		DDA for othe	1894221.1
	No matches	No matches	No matches	No matches		No MS2	71489565
	No matches	No matches	Single match	Single match	60	DDA for othe	53943056
	No matches	No matches	No matches	No matches		No MS2	8106850.7
	No matches	No matches	No matches	No matches		DDA for pref	1067878.2
	No matches	No matches	No matches	No matches		No MS2	3242653.5
	No matches	No matches	No matches	No matches		No MS2	22948249
	No matches	No matches	No matches	No matches		DDA for pref	68553252
	No matches	No matches	No matches	No matches		No MS2	56798992
	Multiple ma	No matches	Single match	No matches		DDA for pref	28780354
	No matches	No matches	No matches	No matches		No MS2	73244511
	No matches	No matches	No matches	No matches		No MS2	60360207
	No matches	No matches	Single match	No matches		No MS2	57484076
	Multiple ma	Single match	Multiple ma	No matches		No MS2	73136273
	Multiple ma	No matches	Single match	No matches		No MS2	39635625
	No matches	No matches	No matches	No matches		DDA for pref	68874740
	No matches	No matches	No matches	No matches		No MS2	73063451
	No matches	No matches	No matches	No matches		DDA for pref	73062789
	No matches	No matches	No matches	No matches		No MS2	45755138
	No matches	No matches	No matches	No matches		DDA for pref	42800829
	No matches	No matches	No matches	Single match		No MS2	72902415
	No matches	No matches	No matches	No matches		No MS2	55429336
	No matches	No matches	No matches	No matches		No MS2	48907822

	No matches	No matches	No matches	No matches		No MS2	43094437
	No matches	No matches	No matches	No matches		DDA for pref	47198926
	No matches	No matches	No matches	No matches		DDA for othe	57403642
	No matches	No matches	No matches	No matches		No MS2	42609339
	No matches	No matches	No matches	No matches		DDA for pref	61160480
	No matches	No matches	No matches	No matches		No MS2	72500350
	No matches	No matches	No matches	Single match		No MS2	61266911
	No matches	No matches	No matches	No matches		No MS2	45675674
	No matches	No matches	No matches	No matches		DDA for pref	56916330
	No matches	No matches	No matches	No matches		No MS2	53197035
	No matches	No matches	No matches	No matches		No MS2	46837129
	No matches	Single match	No matches	No matches		No MS2	72265724
	No matches	No matches	No matches	No matches		No MS2	38464190
	No matches	No matches	Single match	No matches		No MS2	58183372
	No matches	No matches	No matches	No matches		No MS2	40381838
	No matches	No matches	No matches	No matches		No MS2	59602314
	No matches	No matches	No matches	No matches		No MS2	35402625
	No matches	No matches	No matches	No matches		No MS2	63846557
	No matches	No matches	No matches	No matches		No MS2	2220591.5
	No matches	No matches	No matches	No matches		No MS2	71445624
	No matches	No matches	No matches	No matches		No MS2	71436895
	No matches	No matches	Multiple ma	No matches		No MS2	53256717
	No matches	No matches	No matches	No matches		DDA for pref	71306959
	No matches	No matches	No matches	No matches		No MS2	53629011
	No matches	No matches	No matches	No matches		No MS2	71239174
	No matches	No matches	No matches	No matches		No MS2	57157477
	No matches	No matches	No matches	No matches		No MS2	57984827
	No matches	Single match	No matches	No matches		No MS2	61765607
	Multiple ma	No matches	No matches	No matches		No MS2	60579726
	No matches	No matches	Multiple ma	No matches		No MS2	70860433
	No matches	No matches	No matches	No matches		No MS2	54801888
	No matches	No matches	No matches	No matches		No MS2	23924915
	No matches	No matches	No matches	Multiple ma		No MS2	50325723
	No matches	No matches	Multiple ma	No matches		No MS2	39935322
	No matches	No matches	No matches	No matches		No MS2	39810273
	No matches	No matches	No matches	No matches		DDA for pref	70285204
	No matches	Multiple ma	No matches	No matches		No MS2	60840840
	Multiple ma	No matches	No matches	No matches		No MS2	51589286
	No matches	No matches	No matches	No matches		DDA for pref	41015197
	No matches	No matches	No matches	No matches		No MS2	47381333
	No matches	No matches	Multiple ma	No matches		No MS2	67936665
	No matches	No matches	No matches	No matches		No MS2	50848703
	No matches	No matches	No matches	No matches		No MS2	42745220
	No matches	No matches	No matches	No matches		No MS2	62568167
	No matches	No matches	No matches	No matches		No MS2	55005314
	No matches	No matches	No matches	No matches		No MS2	228661.31
	No matches	No matches	No matches	No matches		No MS2	57961575
	No matches	No matches	No matches	No matches		No MS2	26447984
	No matches	No matches	No matches	No matches		DDA for pref	57341312
	No matches	No matches	No matches	No matches		No MS2	69005617

	Multiple ma	Single match	No matches	No matches		No MS2	50403401
	No matches	No matches	No matches	No matches		No MS2	11303618
	No matches	No matches	No matches	No matches		DDA for pref	41870116
	Multiple ma	No matches	No matches	No matches		No MS2	54093624
	No matches	No matches	No matches	No matches		No MS2	52555994
	No matches	No matches	No matches	No matches		DDA for pref	50004459
	No matches	No matches	No matches	No matches		No MS2	68573128
	No matches	No matches	No matches	No matches		No MS2	41012826
	No matches	No matches	No matches	No matches		No MS2	68518732
	No matches	Multiple ma	No matches	No matches		DDA for othe	68290446
	No matches	No matches	No matches	No matches		No MS2	53246023
	No matches	No matches	No matches	No matches		No MS2	52509755
	No matches	No matches	No matches	No matches		No MS2	34579901
	No matches	No matches	No matches	No matches		No MS2	63728481
	No matches	No matches	Single match	No matches		No MS2	58531926
	No matches	No matches	No matches	No matches		No MS2	68179365
	No matches	No matches	Single match	No matches		DDA for pref	32722838
	No matches	No matches	No matches	No matches		No MS2	62927467
	Multiple ma	No matches	No matches	No matches		No MS2	62959947
	Multiple ma	No matches	No matches	No matches		No MS2	67768121
	No matches	No matches	No matches	No matches		No MS2	52934172
	No matches	No matches	No matches	No matches		No MS2	67650879
	No matches	No matches	No matches	No matches		No MS2	67616526
	No matches	No matches	No matches	No matches		No MS2	65486180
	No matches	No matches	No matches	No matches		DDA for pref	30854563
	No matches	No matches	Single match	No matches		No MS2	54968111
	No matches	No matches	No matches	No matches		No MS2	46491770
	No matches	No matches	No matches	No matches		No MS2	44871302
	No matches	No matches	Single match	No matches		DDA for othe	67221509
	No matches	No matches	No matches	No matches		No MS2	67218333
	No matches	Single match	No matches	No matches		No MS2	62299573
	No matches	No matches	No matches	No matches		DDA for pref	22855531
	No matches	No matches	No matches	No matches		No MS2	51466307
	No matches	No matches	No matches	No matches		No MS2	58151556
	No matches	No matches	No matches	No matches		No MS2	52719853
	No matches	No matches	No matches	No matches		DDA for pref	67026637
	No matches	No matches	No matches	No matches		No MS2	67000652
	No matches	No matches	Multiple ma	No matches		DDA for pref	7555703.3
	No matches	No matches	No matches	No matches		No MS2	16837590
	No matches	No matches	Multiple ma	No matches		DDA for pref	66636849
	No matches	No matches	No matches	No matches		No MS2	66584908
	No matches	Single match	No matches	No matches		No MS2	59444461
	No matches	No matches	No matches	No matches		No MS2	2127366.9
	No matches	No matches	No matches	No matches		DDA for pref	48032648
	No matches	Single match	No matches	Single match		No MS2	30622306
	No matches	No matches	Multiple ma	Single match		No MS2	46349031
	No matches	No matches	No matches	No matches	61.9	DDA for pref	66085796
	No matches	No matches	No matches	No matches		No MS2	61201064
	No matches	No matches	No matches	No matches		DDA for pref	65954439
	No matches	No matches	No matches	No matches		DDA for pref	5459667.3

	Multiple ma	No matches	No matches	No matches		DDA for pref	65898715
	No matches	No matches	No matches	No matches		No MS2	54707486
	No matches	No matches	No matches	No matches		No MS2	47165171
	Single match	No matches	No matches	No matches		No MS2	65707585
	No matches	No matches	No matches	No matches		No MS2	46704904
	No matches	No matches	No matches	No matches		DDA for pref	37834523
	No matches	No matches	No matches	Single match		No MS2	47379926
	No matches	Single match	No matches	No matches		No MS2	29801107
	No matches	No matches	No matches	No matches		No MS2	47398313
	No matches	No matches	No matches	No matches		No MS2	64567217
	Multiple ma	No matches	Single match	No matches		No MS2	33419687
	Multiple ma	No matches	Single match	No matches		No MS2	29773525
	Multiple ma	No matches	No matches	No matches		No MS2	65153656
	No matches	No matches	No matches	No matches		No MS2	474434.2
	No matches	No matches	No matches	No matches		No MS2	62741890
	Multiple ma	No matches	No matches	No matches		No MS2	49005005
	No matches	No matches	No matches	No matches		No MS2	43907671
	Multiple ma	No matches	No matches	No matches		No MS2	44106940
	No matches	Single match	No matches	No matches		DDA for pref	64807702
	No matches	No matches	No matches	No matches		No MS2	64620512
	No matches	No matches	No matches	No matches		No MS2	45625075
	No matches	No matches	No matches	No matches		No MS2	44651693
	No matches	No matches	No matches	No matches		No MS2	47263016
	No matches	No matches	No matches	No matches		No MS2	64180948
	No matches	No matches	No matches	No matches		No MS2	43108153
	No matches	No matches	No matches	No matches		No MS2	55884937
	No matches	No matches	No matches	No matches	92.3	DDA for pref	63743319
	No matches	No matches	No matches	No matches		No MS2	51421406
	No matches	No matches	No matches	No matches		DDA for pref	55198759
	No matches	No matches	No matches	No matches		No MS2	54827284
	No matches	No matches	No matches	No matches		No MS2	37366280
	No matches	No matches	No matches	No matches		No MS2	50593300
	No matches	No matches	No matches	No matches		DDA for othe	45917940
	No matches	No matches	No matches	No matches		No MS2	37539023
	No matches	No matches	No matches	No matches		DDA for pref	48528505
	No matches	No matches	No matches	No matches		DDA for pref	43558209
	No matches	No matches	Multiple ma	Multiple ma		No MS2	47920481
	No matches	No matches	No matches	No matches		DDA for pref	57815495
	No matches	No matches	No matches	No matches		No MS2	63115474
	No matches	No matches	Single match	No matches		No MS2	49201663
	No matches	No matches	No matches	No matches		No MS2	29327677
	No matches	No matches	No matches	No matches		No MS2	993752
	Multiple ma	No matches	No matches	No matches		No MS2	63041563
	No matches	No matches	No matches	No matches		No MS2	48936780
	No matches	No matches	No matches	Single match		No MS2	43764589
	No matches	No matches	No matches	No matches		No MS2	58554223
	Multiple ma	No matches	Multiple ma	No matches		No MS2	54388226
	No matches	No matches	No matches	No matches		No MS2	62836021
	No matches	No matches	Single match	No matches		No MS2	40498786
	Multiple ma	No matches	Multiple ma	No matches	85.1	DDA for pref	50011642

	No matches	No matches	No matches	No matches		No MS2	29377422
	Single match	No matches	No matches	No matches		No MS2	45711046
	No matches	No matches	No matches	No matches		No MS2	62204458
	No matches	No matches	No matches	No matches		No MS2	62055747
	No matches	No matches	No matches	No matches		DDA for pref	61966740
	No matches	No matches	No matches	No matches		No MS2	49173697
	No matches	No matches	No matches	No matches		No MS2	2473204.7
	No matches	No matches	Multiple ma	No matches	89.2	DDA for pref	61881641
	No matches	No matches	No matches	No matches		No MS2	61856673
	No matches	No matches	No matches	No matches		No MS2	58598752
	No matches	No matches	No matches	No matches		No MS2	48945852
	No matches	Single match	Single match	No matches		No MS2	32673305
	No matches	No matches	Single match	No matches		DDA for othe	24974489
	No matches	No matches	No matches	No matches		No MS2	35154626
	No matches	No matches	No matches	No matches		No MS2	33312917
	No matches	No matches	No matches	No matches		No MS2	49827590
	No matches	No matches	No matches	No matches		No MS2	26454707
	No matches	No matches	No matches	No matches		DDA for pref	37021875
	No matches	No matches	No matches	No matches		No MS2	61286158
	No matches	No matches	No matches	No matches		No MS2	19899516
	Multiple ma	No matches	Multiple ma	No matches		No MS2	43137250
	No matches	No matches	No matches	No matches		No MS2	61105893
	No matches	No matches	No matches	No matches		No MS2	58111997
	No matches	No matches	No matches	No matches		No MS2	60927741
	No matches	No matches	No matches	No matches		No MS2	50764398
	No matches	No matches	No matches	No matches		No MS2	42299282
	No matches	No matches	Single match	No matches		No MS2	1488765.2
	No matches	No matches	No matches	No matches		No MS2	60755406
	No matches	No matches	No matches	No matches		No MS2	1782172.5
	No matches	No matches	No matches	No matches		No MS2	60727482
	No matches	No matches	No matches	No matches		No MS2	51521422
	Single match	No matches	No matches	No matches		No MS2	44390583
	No matches	No matches	No matches	No matches		No MS2	60633772
	No matches	No matches	No matches	No matches		No MS2	3346204
	Single match	No matches	No matches	No matches		No MS2	60464709
	No matches	No matches	No matches	No matches		No MS2	41047532
	No matches	No matches	No matches	No matches		No MS2	60293802
	No matches	No matches	Single match	No matches		No MS2	53904687
	No matches	No matches	No matches	No matches		No MS2	32427090
	No matches	No matches	No matches	No matches		No MS2	60196265
	No matches	No matches	Single match	No matches		DDA for pref	39672303
	No matches	No matches	Single match	No matches		No MS2	37796995
	No matches	No matches	No matches	No matches		No MS2	37272706
	No matches	No matches	Single match	No matches		No MS2	47822196
	Multiple ma	No matches	No matches	No matches		No MS2	48842120
	No matches	No matches	No matches	No matches		No MS2	5199407.2
	No matches	No matches	No matches	No matches		No MS2	59891084
	No matches	No matches	No matches	No matches		No MS2	440637.73
	No matches	No matches	No matches	No matches		No MS2	47474580
	Multiple ma	No matches	No matches	No matches		No MS2	41392168

	No matches	No matches	Single match	No matches		No MS2	33312200
	No matches	No matches	No matches	Single match		No MS2	2835558.7
	No matches	No matches	No matches	No matches		DDA for pref	40202808
	No matches	No matches	Single match	No matches		No MS2	59633586
	No matches	Single match	Single match	No matches		No MS2	52759179
	No matches	No matches	No matches	No matches		No MS2	37826139
	No matches	No matches	No matches	No matches		DDA for pref	48195449
	No matches	No matches	No matches	No matches		No MS2	59381990
	No matches	No matches	No matches	No matches		No MS2	59374562
	No matches	No matches	No matches	No matches		No MS2	1019730.6
	No matches	No matches	No matches	No matches		No MS2	38912359
	No matches	No matches	No matches	No matches		No MS2	59183203
	Multiple ma	No matches	No matches	No matches		No MS2	44197983
	No matches	No matches	Multiple ma	No matches		No MS2	39032618
	No matches	No matches	No matches	No matches		No MS2	16807091
	Multiple ma	No matches	No matches	No matches		No MS2	59039697
	No matches	No matches	No matches	No matches		No MS2	36431898
	No matches	No matches	No matches	No matches		No MS2	22966917
	No matches	No matches	Single match	No matches		No MS2	35726321
	No matches	No matches	No matches	No matches		No MS2	47907251
	No matches	No matches	No matches	No matches		No MS2	52606148
	No matches	No matches	No matches	No matches		No MS2	58561333
	No matches	No matches	No matches	No matches		DDA for pref	58536556
	No matches	Single match	No matches	No matches		DDA for pref	44261298
	No matches	No matches	No matches	No matches		No MS2	11377929
	No matches	No matches	No matches	No matches		No MS2	58456532
	No matches	Single match	No matches	No matches		No MS2	18095932
	No matches	No matches	No matches	No matches		No MS2	58427020
	No matches	No matches	No matches	No matches		No MS2	8227956.5
	No matches	Multiple ma	No matches	No matches		DDA for pref	30203304
	No matches	No matches	No matches	No matches		No MS2	40572531
	No matches	No matches	No matches	No matches		No MS2	57904129
	No matches	No matches	No matches	No matches		No MS2	57844704
	Multiple ma	No matches	No matches	No matches		No MS2	57076843
	No matches	Single match	No matches	No matches		No MS2	50881826
	No matches	No matches	No matches	No matches		DDA for othe	42989284
	No matches	No matches	No matches	No matches		DDA for pref	330175.75
	Multiple ma	No matches	Single match	No matches		DDA for pref	51140603
	No matches	No matches	Multiple ma	No matches		DDA for othe	57330476
	No matches	No matches	No matches	No matches		No MS2	41670069
	No matches	No matches	Single match	No matches		No MS2	57445827
	No matches	No matches	No matches	No matches		No MS2	57422193
	No matches	No matches	No matches	No matches		No MS2	39239046
	No matches	No matches	Single match	No matches		No MS2	46337216
	No matches	No matches	No matches	No matches		No MS2	30224913
	No matches	No matches	Single match	No matches		No MS2	57230036
	No matches	No matches	No matches	No matches		No MS2	57192275
	No matches	No matches	No matches	No matches		No MS2	57157780
	No matches	No matches	Single match	No matches		DDA for othe	48330618
	No matches	No matches	No matches	No matches		DDA for pref	48599916

	No matches	No matches	No matches	No matches		No MS2	56911430
	No matches	No matches	No matches	No matches		No MS2	47798444
	No matches	No matches	No matches	No matches		No MS2	56833791
	No matches	No matches	Single match	No matches	81.3	DDA for pref	41280143
	No matches	No matches	No matches	No matches		DDA for pref	929686.78
	No matches	Single match	Single match	No matches		No MS2	50071413
	No matches	No matches	No matches	No matches		No MS2	36772590
	Multiple ma	No matches	No matches	Single match		No MS2	56663098
	No matches	No matches	No matches	No matches		No MS2	1190050.3
	No matches	No matches	No matches	No matches		No MS2	50461800
	No matches	No matches	Single match	Single match		No MS2	17040084
	No matches	No matches	No matches	No matches		No MS2	41651513
	No matches	No matches	No matches	No matches		No MS2	44439645
	No matches	No matches	No matches	No matches		No MS2	40883049
	No matches	No matches	No matches	No matches		DDA for pref	25185095
	No matches	No matches	No matches	No matches		No MS2	34085094
	No matches	No matches	No matches	No matches		No MS2	34897209
	No matches	No matches	No matches	No matches		No MS2	56166261
	No matches	No matches	No matches	No matches		No MS2	23364559
	No matches	No matches	No matches	No matches		DDA for pref	31842885
	No matches	No matches	No matches	No matches		No MS2	32585836
	No matches	No matches	No matches	No matches		No MS2	13911679
	Multiple ma	No matches	Single match	No matches		No MS2	31545334
	No matches	Single match	No matches	No matches		No MS2	35827264
	No matches	No matches	No matches	No matches		No MS2	52389792
	No matches	No matches	No matches	No matches		No MS2	2690909.1
	No matches	No matches	No matches	No matches		No MS2	36758162
	No matches	No matches	Single match	No matches		No MS2	38167671
	No matches	No matches	No matches	No matches		No MS2	33096444
	No matches	No matches	No matches	No matches		No MS2	52885541
	No matches	No matches	No matches	No matches		DDA for pref	53789049
	No matches	No matches	Single match	No matches		No MS2	30272244
	No matches	No matches	No matches	No matches		No MS2	45313343
	No matches	No matches	Single match	Single match		DDA for pref	48556297
	No matches	No matches	Multiple ma	No matches		No MS2	38301711
	No matches	No matches	No matches	No matches		No MS2	36072828
	No matches	No matches	No matches	No matches		No MS2	54981461
	No matches	No matches	No matches	No matches		No MS2	52807018
	No matches	No matches	No matches	No matches		No MS2	54917126
	No matches	No matches	No matches	No matches		No MS2	45155771
	No matches	No matches	No matches	No matches		No MS2	54814938
	No matches	No matches	No matches	No matches		DDA for pref	41035761
	No matches	No matches	No matches	No matches		No MS2	29173468
	No matches	No matches	No matches	No matches		No MS2	54683699
	No matches	No matches	Multiple ma	No matches		No MS2	26787869
	No matches	No matches	No matches	No matches		No MS2	35445402
	No matches	No matches	No matches	No matches		DDA for pref	52236929
	No matches	No matches	No matches	No matches		No MS2	9809431.2
	No matches	No matches	No matches	No matches		No MS2	47696384
	No matches	No matches	No matches	No matches		No MS2	54330263

	No matches	No matches	No matches	No matches		DDA for pref	12722829
	No matches	No matches	Multiple ma	No matches		No MS2	52745770
	No matches	No matches	No matches	No matches		DDA for pref	27782046
	No matches	No matches	No matches	No matches		No MS2	33780257
	No matches	No matches	No matches	No matches		DDA for pref	48925191
	No matches	No matches	No matches	No matches		No MS2	53353190
	No matches	No matches	No matches	No matches		No MS2	29005926
	No matches	No matches	No matches	No matches		No MS2	884653.82
	No matches	No matches	Multiple ma	Multiple ma		No MS2	53514987
	No matches	Single match	No matches	No matches		No MS2	46188575
	No matches	Single match	No matches	No matches		No MS2	53431648
	No matches	No matches	No matches	No matches		No MS2	12105090
	No matches	No matches	No matches	No matches		No MS2	40580550
	No matches	No matches	No matches	No matches		No MS2	46781811
	No matches	No matches	Multiple ma	No matches		No MS2	22309805
	No matches	No matches	No matches	No matches		No MS2	6688599.3
	No matches	No matches	No matches	No matches		No MS2	21247401
	No matches	No matches	No matches	No matches		No MS2	53059358
	No matches	No matches	No matches	No matches		No MS2	38866000
	No matches	No matches	No matches	Single match		No MS2	22073562
	No matches	No matches	No matches	No matches		No MS2	51839038
	No matches	No matches	No matches	No matches		No MS2	3165822.7
	No matches	No matches	No matches	No matches		No MS2	33151356
	No matches	No matches	No matches	No matches		DDA for pref	44732413
	No matches	No matches	No matches	No matches		No MS2	45623705
	No matches	No matches	No matches	No matches		No MS2	36677356
	No matches	No matches	No matches	No matches		No MS2	17521668
	Single match	No matches	No matches	No matches		DDA for othe	31423465
	No matches	No matches	No matches	No matches		No MS2	35031939
	No matches	No matches	No matches	No matches		No MS2	52410277
	No matches	No matches	No matches	No matches		No MS2	12835105
	No matches	No matches	No matches	No matches		No MS2	39400503
	No matches	No matches	No matches	No matches		No MS2	972592.42
	No matches	No matches	No matches	No matches		No MS2	52277168
	No matches	No matches	No matches	No matches		No MS2	39412711
	Multiple ma	No matches	No matches	No matches		No MS2	52217205
	No matches	No matches	No matches	No matches		DDA for pref	16064851
	Multiple ma	No matches	No matches	No matches		No MS2	34115072
	No matches	No matches	Single match	No matches		No MS2	5688948.8
	No matches	No matches	No matches	No matches		No MS2	29051449
	No matches	No matches	No matches	No matches		No MS2	14375593
	No matches	No matches	No matches	No matches		DDA for pref	44152828
	No matches	No matches	No matches	No matches		No MS2	10407930
	No matches	No matches	Multiple ma	No matches		No MS2	48455105
	No matches	No matches	No matches	No matches		No MS2	27191119
	No matches	No matches	No matches	No matches		No MS2	32101102
	No matches	No matches	No matches	No matches		No MS2	51522348
	No matches	No matches	No matches	No matches		No MS2	31896710
	No matches	No matches	Single match	No matches		DDA for pref	51299396
	No matches	No matches	Single match	No matches		DDA for pref	51247286

	No matches	No matches	No matches	No matches		No MS2	9675385.7
	No matches	No matches	No matches	No matches		No MS2	18921775
	No matches	No matches	No matches	No matches		DDA for pref	51089690
	No matches	No matches	No matches	No matches		No MS2	2885714.7
	No matches	Single match	No matches	No matches		No MS2	15456631
	No matches	No matches	No matches	No matches		No MS2	18773917
	No matches	No matches	No matches	No matches		No MS2	43796226
	No matches	No matches	Multiple ma	No matches		No MS2	41593501
	No matches	No matches	No matches	No matches		No MS2	37642049
	No matches	No matches	No matches	No matches		No MS2	50736825
	No matches	No matches	No matches	No matches		DDA for pref	36285818
	No matches	No matches	No matches	No matches		No MS2	5068895.4
	No matches	No matches	No matches	No matches		No MS2	50704808
	No matches	No matches	Multiple ma	No matches		No MS2	43690436
	No matches	No matches	No matches	No matches		No MS2	5741866.7
	No matches	No matches	No matches	No matches		No MS2	36554482
	No matches	No matches	No matches	No matches		No MS2	28527385
	No matches	No matches	No matches	No matches		No MS2	28929500
	No matches	No matches	Multiple ma	No matches		No MS2	40433004
	No matches	No matches	Multiple ma	No matches		No MS2	50541888
	No matches	No matches	No matches	No matches		DDA for pref	50457802
	No matches	No matches	No matches	No matches		No MS2	50450116
	Multiple ma	No matches	No matches	No matches		No MS2	43874533
	No matches	No matches	No matches	No matches		No MS2	50391642
	No matches	No matches	No matches	No matches		No MS2	50386259
	No matches	No matches	Single match	No matches		No MS2	23424374
	Multiple ma	No matches	Single match	No matches		No MS2	38130218
	Single match	No matches	No matches	No matches		No MS2	44138210
	No matches	No matches	No matches	No matches		No MS2	29583063
	Multiple ma	No matches	No matches	No matches		No MS2	42135580
	No matches	No matches	No matches	No matches		No MS2	307056.06
	No matches	No matches	No matches	No matches		No MS2	9869225.5
	No matches	No matches	No matches	No matches		No MS2	28090629
	No matches	No matches	No matches	No matches		No MS2	14238203
	No matches	No matches	No matches	No matches		No MS2	36670888
	No matches	No matches	No matches	No matches		No MS2	44976961
	No matches	No matches	No matches	No matches		DDA for pref	47736258
	No matches	No matches	No matches	No matches		No MS2	49994677
	No matches	No matches	No matches	No matches		No MS2	49994677
	No matches	No matches	Single match	Single match		No MS2	49994677
	No matches	No matches	No matches	No matches		No MS2	25049337
	No matches	No matches	No matches	Multiple ma		No MS2	46297998
	Single match	No matches	No matches	No matches		No MS2	37872247
	No matches	No matches	No matches	No matches		No MS2	40314931
	Multiple ma	No matches	No matches	No matches		No MS2	36321960
	No matches	No matches	No matches	No matches		No MS2	44609673
	No matches	No matches	No matches	No matches		No MS2	42818261
	No matches	No matches	No matches	No matches		No MS2	42614900
	No matches	No matches	No matches	No matches		No MS2	40844064
	No matches	No matches	No matches	No matches		No MS2	13466409

	No matches	No matches	No matches	No matches		No MS2	1888849.7
	No matches	Single match	Single match	No matches		DDA for pref	39758931
	Single match	No matches	Multiple ma	No matches		No MS2	28212818
	No matches	No matches	No matches	No matches		No MS2	44310624
	No matches	No matches	No matches	No matches		No MS2	36846759
	No matches	No matches	No matches	No matches		No MS2	49460288
	No matches	No matches	No matches	No matches		No MS2	33581052
	Multiple ma	No matches	No matches	No matches		No MS2	43135804
	Multiple ma	No matches	No matches	No matches		No MS2	14205801
	No matches	No matches	No matches	No matches		No MS2	49297332
	No matches	Single match	Single match	No matches		No MS2	36736201
	Multiple ma	No matches	Multiple ma	No matches		No MS2	4967493.2
	No matches	No matches	No matches	No matches		No MS2	14650683
	No matches	No matches	No matches	No matches		DDA for pref	40380266
	No matches	No matches	No matches	No matches		No MS2	31646504
	No matches	No matches	No matches	No matches		No MS2	42796142
	No matches	No matches	No matches	No matches		No MS2	45885878
	No matches	Single match	Multiple ma	Single match		No MS2	36318440
	Multiple ma	No matches	Multiple ma	No matches		No MS2	37173129
	Multiple ma	No matches	No matches	Single match		DDA for pref	49068944
	No matches	No matches	No matches	No matches		No MS2	49065254
	No matches	No matches	No matches	No matches		No MS2	19346488
	No matches	No matches	No matches	No matches		No MS2	499201.44
	Multiple ma	No matches	No matches	No matches		No MS2	34054617
	No matches	No matches	No matches	No matches		No MS2	48974095
	No matches	No matches	No matches	No matches		No MS2	37753215
	No matches	No matches	No matches	Multiple ma		No MS2	29832064
	No matches	No matches	No matches	No matches		DDA for pref	38246920
	No matches	No matches	No matches	No matches		No MS2	357791.79
	Single match	No matches	No matches	No matches		No MS2	20481168
	No matches	No matches	No matches	No matches		No MS2	41218100
	No matches	No matches	No matches	No matches		No MS2	44765797
	No matches	No matches	No matches	No matches		No MS2	14237934
	No matches	No matches	No matches	No matches		No MS2	28545355
	No matches	No matches	No matches	No matches		No MS2	48386065
	No matches	No matches	No matches	No matches		No MS2	41329959
	No matches	No matches	No matches	No matches		No MS2	831286.27
	No matches	No matches	No matches	No matches		No MS2	48217613
	No matches	No matches	No matches	No matches		No MS2	1473378.4
	No matches	No matches	No matches	No matches		No MS2	41629532
	No matches	No matches	No matches	No matches		No MS2	38768346
	No matches	No matches	No matches	No matches		No MS2	33853942
	No matches	No matches	No matches	No matches		No MS2	41051070
	No matches	Single match	No matches	Single match		No MS2	37147521
	No matches	Single match	No matches	No matches		No MS2	41689479
	No matches	No matches	No matches	No matches		No MS2	40403346
	No matches	No matches	No matches	No matches		No MS2	733749.79
	Multiple ma	No matches	No matches	Single match		No MS2	34150624
	No matches	No matches	Single match	No matches		DDA for pref	8603014.9
	No matches	No matches	No matches	No matches		No MS2	47833159

	No matches	No matches	No matches	No matches		No MS2	40662620
	No matches	No matches	No matches	No matches		No MS2	46944854
	No matches	No matches	No matches	No matches		No MS2	47727176
	No matches	No matches	No matches	No matches		DDA for othe	37070353
	No matches	No matches	No matches	No matches		No MS2	30688005
	No matches	No matches	No matches	No matches		No MS2	1064331.2
	No matches	No matches	Single match	No matches		No MS2	23108278
	No matches	No matches	No matches	No matches		No MS2	29705619
	No matches	No matches	No matches	No matches		No MS2	47535068
	No matches	No matches	Single match	No matches		No MS2	26554633
	No matches	Single match	No matches	Single match		No MS2	47498411
	No matches	Single match	No matches	No matches		No MS2	47498411
	No matches	No matches	No matches	No matches		No MS2	28726540
	No matches	No matches	No matches	No matches		DDA for pref	28514246
	No matches	No matches	No matches	No matches		DDA for pref	38892337
	No matches	No matches	No matches	No matches		No MS2	47396901
	No matches	No matches	No matches	No matches		No MS2	46056828
	No matches	No matches	No matches	No matches		No MS2	585016.32
	No matches	No matches	No matches	No matches		No MS2	33161688
	No matches	No matches	No matches	No matches		DDA for pref	35937597
	No matches	No matches	No matches	No matches		No MS2	43814037
	No matches	No matches	No matches	No matches		No MS2	47256919
	Single match	No matches	No matches	No matches		No MS2	18569676
	No matches	No matches	Single match	No matches		No MS2	41106810
	No matches	No matches	No matches	No matches		No MS2	18406803
	No matches	No matches	No matches	No matches		DDA for pref	20241399
	No matches	No matches	No matches	No matches		No MS2	47116794
	No matches	Multiple ma	Single match	No matches		DDA for pref	18494285
	No matches	No matches	No matches	No matches		No MS2	16105976
	No matches	No matches	No matches	No matches		No MS2	47026872
	No matches	No matches	No matches	Single match		No MS2	1420495.3
	Multiple ma	Single match	Multiple ma	No matches		No MS2	47013456
	No matches	No matches	No matches	No matches		No MS2	28912652
	No matches	No matches	No matches	No matches		No MS2	46846730
	No matches	No matches	No matches	No matches		No MS2	21842275
	No matches	No matches	Multiple ma	Single match		No MS2	6984988.4
	Multiple ma	No matches	Single match	No matches		No MS2	6984988.4
	Multiple ma	No matches	No matches	No matches		No MS2	29196700
	No matches	No matches	No matches	No matches		No MS2	19836713
	Multiple ma	No matches	Multiple ma	No matches	91.8	DDA for pref	36652946
	No matches	No matches	Single match	Single match		No MS2	41713461
	No matches	No matches	No matches	Single match		No MS2	30184755
	No matches	No matches	Multiple ma	No matches		No MS2	46649197
	No matches	Single match	Single match	No matches	60.1	DDA for othe	39855290
	No matches	No matches	No matches	No matches		No MS2	24558844
	No matches	No matches	Single match	No matches		No MS2	46557079
	No matches	No matches	Single match	No matches		No MS2	18206514
	No matches	No matches	No matches	No matches		No MS2	46450174
	No matches	No matches	Multiple ma	No matches		No MS2	16518161
	No matches	No matches	No matches	No matches		No MS2	921214.34

	No matches	No matches	No matches	No matches		No MS2	46371330
	No matches	No matches	No matches	No matches		DDA for pref	28908704
	No matches	No matches	No matches	No matches		No MS2	594215.44
	No matches	No matches	No matches	No matches		No MS2	32800559
	No matches	No matches	No matches	No matches		No MS2	24098460
	No matches	No matches	Multiple ma	No matches		No MS2	46210167
	No matches	No matches	No matches	No matches		No MS2	22913207
	No matches	No matches	No matches	Multiple ma		DDA for pref	26463218
	No matches	No matches	No matches	No matches		No MS2	30060001
	No matches	No matches	No matches	No matches		No MS2	17043635
	No matches	No matches	No matches	No matches		No MS2	3485888.5
	No matches	No matches	No matches	No matches		No MS2	34949878
	No matches	No matches	No matches	No matches		No MS2	18769168
	No matches	No matches	No matches	No matches		No MS2	46018506
	No matches	No matches	No matches	No matches		No MS2	32687967
	No matches	No matches	No matches	No matches		No MS2	42965540
	No matches	No matches	No matches	No matches		No MS2	43374521
	No matches	No matches	No matches	No matches		No MS2	29880397
	No matches	No matches	No matches	No matches		No MS2	10935156
	No matches	No matches	No matches	No matches		No MS2	29779956
	No matches	No matches	Single match	No matches		No MS2	31447986
	No matches	Single match	No matches	Single match		No MS2	45522241
	No matches	No matches	No matches	No matches		No MS2	45413236
	No matches	No matches	No matches	No matches		No MS2	34138909
	No matches	No matches	No matches	No matches		No MS2	38718812
	No matches	No matches	No matches	No matches		No MS2	45302854
	No matches	No matches	No matches	No matches		No MS2	300595.77
	No matches	No matches	Multiple ma	No matches		DDA for pref	43996092
	No matches	No matches	No matches	No matches		No MS2	45140841
	Multiple ma	Single match	Multiple ma	Single match	66.3	DDA for pref	28939099
	No matches	No matches	No matches	No matches		DDA for pref	36829637
	No matches	No matches	No matches	No matches		No MS2	45075673
	No matches	No matches	No matches	No matches		DDA for pref	39565500
	No matches	No matches	No matches	No matches		No MS2	31551892
	No matches	No matches	No matches	No matches		No MS2	31157769
	No matches	No matches	No matches	No matches		No MS2	35632960
	No matches	No matches	Single match	No matches		No MS2	28517937
	No matches	No matches	Single match	No matches		No MS2	44881525
	No matches	No matches	No matches	No matches		No MS2	33518619
	No matches	No matches	No matches	No matches		No MS2	25810004
	No matches	No matches	No matches	No matches		No MS2	33235994
	No matches	No matches	No matches	No matches		No MS2	34389242
	No matches	No matches	No matches	No matches		DDA for pref	37910357
	No matches	No matches	No matches	No matches		No MS2	44512411
	No matches	No matches	No matches	No matches		No MS2	36510404
	No matches	No matches	Single match	No matches		No MS2	44467528
	No matches	No matches	Single match	No matches		No MS2	9212797.8
	No matches	No matches	No matches	No matches		No MS2	44415794
	No matches	No matches	No matches	No matches		No MS2	30335232
	No matches	No matches	No matches	No matches		No MS2	1085501.4

	Single match	No matches	No matches	No matches		No MS2	31135378
	No matches	No matches	Single match	No matches		No MS2	13490028
	No matches	No matches	No matches	No matches		No MS2	44272956
	No matches	No matches	Multiple ma	No matches		No MS2	44262366
	Multiple ma	No matches	No matches	No matches		No MS2	35736821
	No matches	Single match	No matches	No matches		No MS2	22859328
	No matches	No matches	No matches	No matches		DDA for pref	38892337
	Multiple ma	No matches	Multiple ma	No matches		DDA for othe	42866741
	No matches	No matches	No matches	No matches		No MS2	43977040
	No matches	No matches	No matches	No matches		No MS2	43959494
	Single match	No matches	No matches	No matches		DDA for pref	22422066
	No matches	No matches	No matches	No matches		No MS2	42847465
	No matches	No matches	No matches	No matches		No MS2	35060973
	No matches	No matches	Multiple ma	Single match		No MS2	20278274
	No matches	No matches	No matches	No matches		No MS2	39803533
	No matches	No matches	No matches	No matches		DDA for othe	43081471
	No matches	No matches	No matches	No matches		No MS2	35257710
	No matches	No matches	No matches	No matches		No MS2	34237119
	No matches	No matches	No matches	No matches		No MS2	43618304
	No matches	No matches	No matches	No matches		No MS2	43618304
	No matches	No matches	No matches	No matches		No MS2	37656717
	Multiple ma	No matches	No matches	No matches		No MS2	35002900
	No matches	No matches	No matches	No matches		No MS2	30018712
	No matches	No matches	No matches	No matches		No MS2	36759536
	Multiple ma	Single match	Multiple ma	No matches		DDA for othe	43361191
	No matches	No matches	No matches	No matches		No MS2	43279131
	No matches	No matches	No matches	No matches		No MS2	37748521
	No matches	No matches	No matches	No matches		No MS2	30501183
	No matches	No matches	No matches	No matches		No MS2	905413.27
	No matches	No matches	No matches	No matches		No MS2	40908647
	No matches	No matches	No matches	No matches		No MS2	936875.17
	No matches	No matches	No matches	No matches		No MS2	37871378
	No matches	Single match	Single match	No matches		No MS2	15222467
	No matches	No matches	No matches	No matches		No MS2	38954490
	Multiple ma	No matches	No matches	No matches		No MS2	13209015
	No matches	No matches	No matches	Single match		No MS2	43085132
	Multiple ma	No matches	No matches	No matches		No MS2	34555388
	No matches	No matches	No matches	No matches		No MS2	42928546
	Multiple ma	No matches	Multiple ma	No matches		No MS2	6443196.1
	Multiple ma	No matches	Single match	No matches		No MS2	34850660
	No matches	No matches	No matches	No matches		No MS2	42909253
	No matches	No matches	No matches	No matches		No MS2	42904305
	No matches	No matches	No matches	No matches		DDA for pref	20403407
	No matches	No matches	No matches	No matches		No MS2	24956193
	No matches	Single match	No matches	No matches		No MS2	42666604
	No matches	No matches	No matches	No matches		No MS2	32630641
	No matches	No matches	No matches	No matches		No MS2	31757107
	No matches	No matches	No matches	No matches		No MS2	40741015
	No matches	No matches	No matches	No matches		No MS2	11384340
	No matches	Single match	Single match	No matches	68.6	DDA for othe	29136542

	Multiple ma	No matches	No matches	No matches		No MS2	26610522
	No matches	No matches	No matches	No matches		No MS2	37723405
	No matches	No matches	No matches	No matches		No MS2	33448400
	No matches	No matches	Single match	No matches		No MS2	42576340
	No matches	No matches	No matches	No matches		No MS2	42562964
	No matches	No matches	No matches	No matches		No MS2	19705461
	No matches	No matches	No matches	No matches		DDA for pref	12451442
	No matches	No matches	No matches	No matches		No MS2	42511797
	No matches	No matches	No matches	No matches		DDA for pref	15329639
	No matches	No matches	No matches	No matches		No MS2	18770321
	No matches	No matches	No matches	No matches		No MS2	18356044
	No matches	No matches	No matches	No matches		No MS2	31901470
	No matches	No matches	No matches	No matches		No MS2	23990319
	No matches	No matches	No matches	No matches		No MS2	2932063.7
	Multiple ma	No matches	No matches	No matches		No MS2	40474832
	No matches	No matches	No matches	No matches		No MS2	42338599
	No matches	No matches	No matches	No matches		No MS2	5652052.4
	No matches	No matches	No matches	No matches		No MS2	4783841.5
	No matches	No matches	No matches	No matches		DDA for othe	32550131
	No matches	No matches	No matches	No matches		DDA for pref	42172990
	No matches	No matches	No matches	No matches		No MS2	33682096
	No matches	No matches	No matches	No matches		No MS2	42062832
	No matches	No matches	No matches	No matches		No MS2	8145361.2
	No matches	No matches	No matches	No matches		No MS2	7694715
	No matches	No matches	Multiple ma	No matches		No MS2	12928583
	No matches	No matches	Single match	No matches		No MS2	32692881
	No matches	No matches	No matches	No matches		No MS2	1450815.7
	No matches	No matches	No matches	No matches		No MS2	34276154
	No matches	Single match	No matches	No matches		No MS2	41827834
	No matches	No matches	No matches	No matches		No MS2	17965719
	No matches	No matches	No matches	No matches		No MS2	20562349
	No matches	No matches	Multiple ma	Single match		No MS2	29415162
	No matches	No matches	No matches	No matches		No MS2	38293397
	No matches	No matches	No matches	No matches		No MS2	24233312
	No matches	No matches	No matches	No matches		No MS2	31485832
	No matches	No matches	No matches	No matches		No MS2	30654009
	No matches	No matches	Multiple ma	No matches		No MS2	29415162
	No matches	No matches	No matches	No matches		No MS2	1861975
	Multiple ma	No matches	Single match	No matches		DDA for pref	489239.13
	No matches	No matches	No matches	No matches		No MS2	41587844
	Multiple ma	No matches	No matches	No matches		No MS2	41574377
	No matches	No matches	Single match	No matches		No MS2	40451233
	No matches	No matches	Single match	No matches		No MS2	24516231
	Single match	No matches	No matches	No matches		No MS2	36047489
	No matches	No matches	No matches	No matches		No MS2	38212986
	No matches	No matches	Single match	No matches		No MS2	2471491.9
	No matches	No matches	No matches	No matches		No MS2	15091257
	No matches	No matches	No matches	No matches		No MS2	41342689
	No matches	No matches	No matches	No matches		No MS2	14128574
	No matches	No matches	No matches	No matches		No MS2	41324287

	Multiple ma	Single match	Multiple ma	No matches		DDA for pref	40419896
	No matches	No matches	No matches	No matches		No MS2	41219884
	No matches	No matches	No matches	No matches		No MS2	3712115.1
	No matches	No matches	No matches	Multiple ma		No MS2	20135405
	No matches	No matches	No matches	No matches		No MS2	30213533
	No matches	No matches	No matches	No matches		DDA for pref	34503569
	No matches	No matches	No matches	No matches		No MS2	20583509
	No matches	No matches	No matches	No matches		No MS2	41011134
	No matches	No matches	Multiple ma	No matches		No MS2	26667170
	No matches	No matches	Single match	No matches		No MS2	40944201
	No matches	No matches	No matches	No matches		No MS2	33578874
	No matches	No matches	No matches	No matches		No MS2	33627580
	No matches	No matches	No matches	No matches		No MS2	21036662
	No matches	No matches	No matches	No matches		No MS2	29341572
	No matches	No matches	No matches	No matches		DDA for pref	33042359
	No matches	No matches	Multiple ma	No matches		No MS2	30085132
	No matches	No matches	No matches	No matches		No MS2	23692498
	No matches	No matches	Single match	No matches		No MS2	28317481
	No matches	No matches	No matches	No matches		No MS2	29961498
	No matches	No matches	No matches	No matches		No MS2	32180828
	Multiple ma	No matches	No matches	No matches		No MS2	37079291
	No matches	No matches	No matches	No matches		No MS2	34788304
	No matches	No matches	No matches	No matches		No MS2	32271145
	No matches	No matches	Multiple ma	Single match		No MS2	22634491
	No matches	No matches	No matches	No matches		No MS2	35580637
	No matches	No matches	No matches	No matches		No MS2	31354152
	No matches	No matches	No matches	No matches		No MS2	31481946
	No matches	No matches	No matches	No matches		No MS2	26280317
	No matches	No matches	No matches	No matches		No MS2	29891547
	No matches	No matches	Single match	No matches		No MS2	40282650
	No matches	No matches	No matches	No matches		No MS2	30232013
	No matches	No matches	No matches	No matches		No MS2	39646584
	No matches	No matches	No matches	No matches		No MS2	19038542
	No matches	No matches	Multiple ma	No matches		No MS2	30325053
	No matches	No matches	No matches	No matches		No MS2	20172946
	No matches	No matches	No matches	No matches		DDA for pref	28209640
	No matches	No matches	No matches	No matches		No MS2	40114516
	No matches	No matches	No matches	No matches		No MS2	26527531
	No matches	No matches	No matches	No matches		No MS2	5297580.7
	No matches	Single match	No matches	No matches		No MS2	36467845
	No matches	No matches	No matches	No matches		No MS2	23465077
	No matches	No matches	No matches	No matches		No MS2	14071984
	No matches	Single match	No matches	No matches		No MS2	23099194
	Multiple ma	Single match	Multiple ma	No matches		No MS2	18268961
	Multiple ma	Single match	No matches	No matches		No MS2	39885908
	No matches	No matches	No matches	No matches		No MS2	10949767
	No matches	No matches	Single match	No matches		No MS2	27117745
	No matches	Single match	Single match	No matches		No MS2	23538292
	No matches	No matches	No matches	No matches		No MS2	39778855
	No matches	No matches	No matches	No matches		No MS2	28561574

	No matches	No matches	No matches	No matches		No MS2	39113389
	No matches	No matches	No matches	No matches		No MS2	1911631.9
	Multiple ma	No matches	Multiple ma	No matches		No MS2	31668816
	No matches	No matches	No matches	No matches		No MS2	39631526
	No matches	No matches	Multiple ma	No matches		No MS2	21390417
	No matches	No matches	No matches	No matches		No MS2	39597097
	No matches	No matches	No matches	No matches		No MS2	32614297
	No matches	No matches	No matches	No matches		No MS2	28650714
	No matches	No matches	Single match	Single match		No MS2	31163962
	No matches	No matches	Single match	No matches		No MS2	31388557
	No matches	No matches	No matches	No matches		DDA for pref	17908162
	No matches	No matches	No matches	No matches		No MS2	39361533
	Multiple ma	No matches	No matches	No matches		No MS2	26562886
	No matches	No matches	No matches	No matches		No MS2	687250.03
	No matches	Single match	Multiple ma	No matches		No MS2	23054688
	No matches	No matches	No matches	No matches		No MS2	29197113
	No matches	No matches	Single match	Single match		No MS2	21624813
	No matches	No matches	No matches	No matches		No MS2	22775770
	No matches	No matches	No matches	No matches		No MS2	39191007
	No matches	No matches	No matches	No matches		No MS2	25721102
	No matches	No matches	No matches	No matches		No MS2	39153924
	No matches	No matches	No matches	No matches		No MS2	20154915
	No matches	No matches	No matches	No matches		No MS2	24400272
	No matches	No matches	No matches	No matches		DDA for othe	32054879
	No matches	No matches	No matches	Single match		No MS2	25843485
	No matches	No matches	No matches	No matches		No MS2	898638.91
	No matches	No matches	No matches	No matches		No MS2	38851768
	No matches	No matches	No matches	No matches		No MS2	30873300
	No matches	No matches	No matches	No matches		No MS2	38435953
	No matches	No matches	No matches	No matches		No MS2	28418223
	No matches	No matches	No matches	No matches		No MS2	38732184
	No matches	No matches	Single match	No matches		No MS2	38721599
	No matches	No matches	No matches	No matches		No MS2	35731804
	No matches	No matches	No matches	No matches		No MS2	14914221
	No matches	No matches	Multiple ma	Multiple ma		DDA for pref	23925684
	No matches	No matches	No matches	No matches		No MS2	31014399
	No matches	No matches	No matches	No matches		No MS2	36326412
	No matches	No matches	No matches	No matches		No MS2	33913743
	No matches	No matches	No matches	No matches		No MS2	20618424
	No matches	No matches	No matches	No matches		No MS2	27524249
	No matches	No matches	No matches	No matches		No MS2	38588845
	No matches	No matches	No matches	No matches		No MS2	31884275
	Multiple ma	No matches	No matches	No matches		DDA for pref	35967301
	No matches	No matches	Multiple ma	Multiple ma		No MS2	36600060
	No matches	No matches	No matches	No matches		No MS2	18981462
	No matches	No matches	No matches	No matches		No MS2	20822089
	No matches	No matches	No matches	No matches		No MS2	31489147
	No matches	No matches	No matches	No matches		No MS2	31640225
	No matches	No matches	No matches	No matches		No MS2	29804184
	No matches	No matches	No matches	No matches		No MS2	20916004

	No matches	No matches	No matches	No matches		No MS2	1025935.6
	No matches	No matches	No matches	No matches		No MS2	38341888
	No matches	No matches	No matches	No matches		No MS2	11295358
	No matches	No matches	No matches	No matches		No MS2	18309392
	No matches	No matches	Single match	No matches		No MS2	15978184
	No matches	No matches	No matches	No matches		No MS2	35074441
	No matches	No matches	No matches	No matches		No MS2	38216036
	No matches	No matches	No matches	No matches		DDA for pref	38197074
	No matches	No matches	No matches	No matches		No MS2	38182932
	No matches	No matches	No matches	No matches		No MS2	38159606
	Single match	No matches	No matches	No matches		No MS2	9301030.5
	Multiple ma	No matches	No matches	No matches		DDA for pref	9349597.5
	Multiple ma	No matches	Multiple ma	No matches		DDA for othe	34684522
	Multiple ma	No matches	Single match	No matches		No MS2	30135900
	No matches	No matches	No matches	No matches		No MS2	23437258
	No matches	No matches	No matches	No matches		No MS2	986732.46
	No matches	No matches	No matches	No matches		No MS2	25206635
	No matches	No matches	No matches	No matches		No MS2	308960.26
	No matches	No matches	No matches	No matches		No MS2	18496664
	No matches	No matches	No matches	No matches		No MS2	29873077
	No matches	No matches	No matches	No matches		No MS2	37898487
	No matches	No matches	No matches	No matches		No MS2	37888303
	No matches	No matches	No matches	No matches		No MS2	1278604.5
	No matches	No matches	No matches	No matches		No MS2	37788292
	No matches	No matches	No matches	No matches		No MS2	34012897
	No matches	No matches	Single match	No matches		No MS2	35529903
	No matches	No matches	No matches	No matches		No MS2	22160257
	No matches	No matches	No matches	No matches		No MS2	30286450
	No matches	No matches	No matches	No matches		No MS2	24081108
	No matches	No matches	Multiple ma	Single match		No MS2	23986693
	No matches	No matches	No matches	Multiple ma		DDA for pref	32285319
	Multiple ma	No matches	No matches	No matches		No MS2	37451162
	No matches	No matches	No matches	No matches		No MS2	25163133
	No matches	No matches	No matches	No matches		No MS2	21556317
	No matches	No matches	Single match	No matches		No MS2	20336148
	No matches	No matches	No matches	No matches		No MS2	33444824
	No matches	No matches	Multiple ma	No matches		No MS2	36744644
	No matches	No matches	No matches	No matches		No MS2	31532349
	No matches	No matches	No matches	No matches		DDA for othe	23998786
	Multiple ma	No matches	No matches	No matches		No MS2	26186257
	No matches	No matches	No matches	No matches		No MS2	25732484
	No matches	No matches	No matches	No matches		No MS2	18411128
	No matches	No matches	No matches	No matches		No MS2	654480.44
	No matches	No matches	No matches	No matches		No MS2	37049077
	No matches	No matches	No matches	No matches		DDA for othe	37028782
	No matches	No matches	No matches	No matches		No MS2	37004379
	No matches	No matches	No matches	No matches		No MS2	16380098
	No matches	No matches	No matches	No matches		No MS2	36929424
	No matches	No matches	No matches	No matches		No MS2	36917605
	No matches	No matches	No matches	No matches		No MS2	36895496

	No matches	No matches	No matches	No matches		No MS2	19341341
	No matches	No matches	Multiple ma	No matches		No MS2	29368604
	No matches	No matches	No matches	No matches		No MS2	36842034
	No matches	No matches	No matches	No matches		No MS2	14797211
	No matches	No matches	No matches	No matches		DDA for othe	32788645
	No matches	No matches	No matches	No matches		No MS2	36768090
	No matches	No matches	No matches	No matches		No MS2	10530413
	No matches	No matches	No matches	No matches		No MS2	31565423
	No matches	No matches	No matches	No matches		No MS2	36727399
	Multiple ma	No matches	Single match	No matches		No MS2	11359541
	No matches	No matches	No matches	No matches		No MS2	21464872
	No matches	No matches	No matches	No matches		No MS2	9283582.8
	No matches	No matches	No matches	No matches		No MS2	29128044
	No matches	No matches	No matches	No matches		No MS2	22850409
	No matches	No matches	Multiple ma	No matches		No MS2	36625034
	No matches	No matches	No matches	No matches		DDA for pref	27466981
	Multiple ma	No matches	No matches	No matches		No MS2	36597019
	No matches	No matches	No matches	No matches		No MS2	31251759
	No matches	No matches	No matches	No matches		No MS2	1302304.7
	No matches	No matches	Single match	No matches		No MS2	36368716
	Multiple ma	No matches	No matches	No matches		No MS2	28993379
	No matches	No matches	No matches	No matches		No MS2	18955381
	No matches	No matches	No matches	No matches		No MS2	30118150
	No matches	No matches	No matches	No matches		No MS2	10550987
	No matches	No matches	No matches	No matches		No MS2	32502546
	No matches	No matches	No matches	No matches		DDA for pref	36391596
	No matches	No matches	No matches	No matches		No MS2	25097642
	No matches	No matches	No matches	No matches		No MS2	1924365.6
	Multiple ma	No matches	Multiple ma	Single match		No MS2	28807905
	No matches	No matches	No matches	No matches		DDA for pref	6589831.7
	No matches	No matches	Multiple ma	Single match		No MS2	29376820
	Multiple ma	No matches	Multiple ma	No matches		No MS2	33610860
	No matches	No matches	No matches	No matches		No MS2	25464652
	No matches	No matches	No matches	No matches		No MS2	11098247
	No matches	No matches	No matches	No matches		DDA for pref	3102830.3
	No matches	No matches	No matches	No matches		No MS2	36207764
	No matches	No matches	No matches	No matches		No MS2	26603717
	No matches	No matches	No matches	No matches		No MS2	2064197.6
	No matches	No matches	No matches	No matches		No MS2	1783139
	No matches	No matches	No matches	No matches		No MS2	17484887
	No matches	No matches	No matches	No matches		No MS2	22970432
	No matches	No matches	No matches	No matches		No MS2	26219217
	No matches	No matches	No matches	No matches		No MS2	33340822
	No matches	No matches	No matches	Single match		No MS2	13541507
	No matches	Single match	Single match	No matches		No MS2	36027162
	No matches	No matches	No matches	No matches		No MS2	23961937
	No matches	No matches	No matches	No matches		No MS2	36022094
	No matches	No matches	Multiple ma	Single match		No MS2	36018442
	No matches	No matches	No matches	No matches		No MS2	19404177
	No matches	No matches	No matches	No matches		No MS2	35985344

	No matches	No matches	No matches	No matches		No MS2	32640476
	No matches	No matches	No matches	No matches		No MS2	822884.65
	No matches	No matches	No matches	No matches		No MS2	29893108
	No matches	No matches	No matches	No matches		No MS2	22674144
	No matches	No matches	No matches	No matches		No MS2	25342080
	No matches	No matches	No matches	No matches		No MS2	1592394.4
	No matches	No matches	No matches	No matches		No MS2	30256952
	No matches	No matches	No matches	No matches		No MS2	35866491
	No matches	No matches	No matches	No matches		No MS2	29328362
	No matches	No matches	Single match	No matches		No MS2	26307257
	No matches	No matches	No matches	No matches		No MS2	22353273
	Multiple ma	No matches	Multiple ma	No matches		No MS2	16292049
	No matches	No matches	Multiple ma	Multiple ma		No MS2	27110739
	Multiple ma	No matches	No matches	No matches		No MS2	31137755
	No matches	No matches	Single match	No matches		DDA for pref	2040960.3
	No matches	No matches	No matches	No matches		DDA for pref	35705741
	No matches	No matches	No matches	Single match		No MS2	25523860
	No matches	No matches	No matches	No matches		No MS2	21338579
	No matches	No matches	No matches	No matches		No MS2	25917385
	No matches	No matches	No matches	Single match		No MS2	23182156
	No matches	No matches	No matches	No matches		DDA for pref	35549702
	No matches	No matches	No matches	No matches		No MS2	29482156
	No matches	No matches	No matches	No matches		No MS2	28340626
	No matches	No matches	No matches	No matches		No MS2	14280288
	No matches	No matches	No matches	No matches		No MS2	18527733
	Multiple ma	No matches	Single match	No matches		DDA for othe	35443802
	No matches	No matches	No matches	No matches		No MS2	35430831
	No matches	No matches	No matches	No matches		No MS2	35412969
	No matches	No matches	No matches	No matches		No MS2	1002051.6
	No matches	No matches	Single match	No matches		No MS2	29383970
	No matches	No matches	No matches	No matches		No MS2	20628504
	No matches	No matches	No matches	No matches		No MS2	31486203
	No matches	No matches	No matches	No matches		No MS2	35313705
	Multiple ma	No matches	No matches	No matches		No MS2	20611630
	No matches	No matches	No matches	No matches		No MS2	35296496
	No matches	No matches	No matches	No matches		No MS2	28276562
	No matches	No matches	No matches	No matches		No MS2	35264290
	No matches	No matches	No matches	No matches		No MS2	10247084
	No matches	No matches	No matches	No matches		DDA for pref	23856924
	No matches	No matches	No matches	No matches		No MS2	32382562
	No matches	No matches	No matches	No matches		No MS2	30951801
	No matches	No matches	No matches	No matches		No MS2	35210786
	No matches	No matches	No matches	No matches		No MS2	33841155
	No matches	No matches	No matches	No matches		No MS2	17792503
	No matches	No matches	No matches	No matches		No MS2	15408772
	No matches	Single match	No matches	No matches		No MS2	25170850
	No matches	No matches	No matches	No matches		No MS2	20674674
	No matches	No matches	No matches	No matches		No MS2	17147596
	No matches	No matches	No matches	No matches		No MS2	22504234
	No matches	No matches	No matches	No matches		No MS2	29472738

	No matches	No matches	No matches	No matches		No MS2	24953449
	No matches	No matches	No matches	No matches		DDA for pref	35063629
	No matches	No matches	Single match	No matches	89.6	DDA for pref	33743196
	No matches	No matches	No matches	No matches		No MS2	22537198
	No matches	No matches	No matches	No matches		No MS2	2409208.4
	No matches	No matches	No matches	No matches		No MS2	3827321.2
	No matches	No matches	No matches	No matches		DDA for pref	34791508
	No matches	No matches	No matches	No matches		No MS2	26010244
	No matches	No matches	No matches	No matches		No MS2	23309974
	No matches	No matches	No matches	No matches		No MS2	22547820
	No matches	No matches	Single match	No matches		No MS2	21563293
	No matches	No matches	No matches	No matches		No MS2	23944759
	No matches	No matches	No matches	No matches		No MS2	29593797
	Multiple ma	No matches	No matches	No matches		No MS2	34628376
	No matches	No matches	No matches	No matches		DDA for pref	34530163
	No matches	No matches	No matches	No matches		No MS2	26441639
	No matches	No matches	No matches	No matches		No MS2	34485394
	No matches	No matches	No matches	No matches		No MS2	21588418
	No matches	No matches	No matches	No matches		No MS2	21758126
	No matches	No matches	No matches	No matches		No MS2	20657283
	Multiple ma	No matches	No matches	No matches		No MS2	24626150
	No matches	No matches	No matches	No matches		No MS2	20447039
	No matches	No matches	No matches	No matches		No MS2	20447039
	No matches	No matches	No matches	No matches		No MS2	25062040
	No matches	No matches	No matches	No matches		No MS2	5804497.8
	No matches	No matches	No matches	No matches		No MS2	34268561
	No matches	No matches	Single match	No matches		No MS2	26333526
	No matches	No matches	No matches	No matches		DDA for pref	10318547
	No matches	No matches	No matches	No matches		No MS2	31255952
	No matches	No matches	No matches	No matches		DDA for pref	31271361
	Multiple ma	No matches	No matches	No matches		No MS2	27718280
	No matches	No matches	No matches	No matches		DDA for othe	23939316
	No matches	No matches	Multiple ma	No matches		No MS2	22262798
	No matches	No matches	No matches	No matches		No MS2	23294987
	No matches	No matches	No matches	No matches		No MS2	25988909
	No matches	No matches	No matches	No matches		No MS2	24016565
	No matches	Single match	No matches	No matches		No MS2	28425320
	No matches	No matches	No matches	No matches		DDA for othe	13420151
	No matches	No matches	No matches	No matches		No MS2	8824181.7
	Multiple ma	No matches	No matches	No matches		No MS2	31235789
	No matches	No matches	No matches	No matches		No MS2	22883972
	No matches	Single match	Single match	No matches		No MS2	24658780
	Multiple ma	No matches	No matches	No matches		No MS2	23664847
	No matches	Single match	Single match	No matches		No MS2	17642267
	No matches	No matches	No matches	No matches		No MS2	27696107
	No matches	No matches	No matches	No matches		No MS2	22023961
	No matches	No matches	No matches	No matches		No MS2	15667313
	No matches	No matches	No matches	No matches		No MS2	21414311
	Multiple ma	No matches	No matches	No matches		No MS2	12825604
	No matches	No matches	No matches	No matches		No MS2	20929180

	No matches	No matches	Multiple ma	No matches		No MS2	33492992
	No matches	No matches	Multiple ma	No matches		No MS2	14264279
	Single match	No matches	No matches	No matches		No MS2	26889358
	No matches	No matches	No matches	No matches		No MS2	33445906
	No matches	No matches	No matches	No matches		No MS2	16716626
	No matches	No matches	No matches	No matches		No MS2	18542812
	No matches	No matches	No matches	No matches		No MS2	30852673
	No matches	No matches	No matches	No matches		No MS2	26974623
	No matches	No matches	No matches	No matches		No MS2	33321231
	No matches	No matches	No matches	No matches		No MS2	33261236
	No matches	No matches	No matches	No matches		No MS2	23935673
	No matches	No matches	No matches	No matches		No MS2	30570341
	Multiple ma	No matches	No matches	No matches		No MS2	33231664
	No matches	No matches	No matches	No matches		No MS2	2865380.1
	Multiple ma	No matches	Multiple ma	No matches		DDA for pref	25824421
	No matches	No matches	No matches	No matches		DDA for pref	17283330
	No matches	No matches	No matches	No matches		No MS2	25912722
	No matches	No matches	No matches	No matches		DDA for pref	2407410.3
	No matches	No matches	No matches	No matches		No MS2	27381491
	No matches	No matches	No matches	No matches		DDA for pref	27220722
	No matches	No matches	No matches	No matches		No MS2	19517171
	No matches	No matches	No matches	No matches		No MS2	23730146
	No matches	No matches	No matches	No matches		No MS2	32879740
	No matches	No matches	No matches	No matches		No MS2	32876873
	No matches	No matches	No matches	No matches		No MS2	32845845
	No matches	No matches	No matches	No matches		DDA for pref	30892320
	No matches	No matches	No matches	No matches		No MS2	19311738
	No matches	No matches	No matches	No matches		No MS2	1866983.5
	No matches	Single match	Multiple ma	No matches		No MS2	2942953.4
	No matches	No matches	No matches	No matches		No MS2	21596716
	No matches	No matches	No matches	No matches		No MS2	1678599.2
	No matches	No matches	No matches	Single match		No MS2	27247638
	No matches	Single match	No matches	Single match		No MS2	32661189
	No matches	No matches	No matches	No matches		DDA for pref	32649313
	Multiple ma	No matches	No matches	No matches		No MS2	15126366
	No matches	No matches	No matches	No matches		No MS2	32625109
	No matches	No matches	No matches	No matches		No MS2	23836596
	No matches	No matches	Single match	No matches		No MS2	32588044
	No matches	No matches	No matches	No matches		No MS2	22976539
	No matches	No matches	No matches	No matches		No MS2	9802890
	No matches	No matches	No matches	No matches		No MS2	29805358
	No matches	No matches	No matches	No matches		No MS2	23966598
	No matches	No matches	No matches	No matches		No MS2	15485631
	No matches	No matches	Multiple ma	No matches		No MS2	11728860
	No matches	No matches	No matches	No matches		No MS2	32444126
	Multiple ma	No matches	No matches	No matches		No MS2	27423953
	Multiple ma	No matches	No matches	No matches		No MS2	32390151
	No matches	No matches	No matches	No matches		No MS2	16305325
	No matches	No matches	No matches	No matches		No MS2	16513940
	No matches	No matches	No matches	Single match		No MS2	16098173

	No matches	No matches	No matches	No matches		No MS2	3918593.2
	No matches	No matches	No matches	No matches		No MS2	32247646
	No matches	No matches	No matches	No matches		No MS2	4702699.1
	No matches	No matches	No matches	No matches		No MS2	25492237
	No matches	No matches	No matches	No matches		No MS2	27295570
	No matches	No matches	No matches	No matches		No MS2	24376682
	No matches	No matches	No matches	No matches		No MS2	28195444
	Multiple ma	No matches	No matches	No matches		No MS2	32085547
	No matches	No matches	Single match	No matches		No MS2	17842553
	No matches	No matches	No matches	No matches		No MS2	31340066
	Multiple ma	No matches	No matches	No matches		No MS2	31966086
	No matches	Single match	No matches	No matches		No MS2	15668932
	No matches	No matches	No matches	No matches		No MS2	7929325.6
	Multiple ma	No matches	Multiple ma	No matches		No MS2	22012890
	No matches	No matches	No matches	No matches		No MS2	21749955
	No matches	No matches	No matches	No matches		No MS2	28105433
	No matches	No matches	No matches	Single match		No MS2	31883982
	No matches	No matches	Single match	No matches		No MS2	4652523.6
	No matches	No matches	No matches	No matches		No MS2	31830661
	No matches	No matches	No matches	No matches		No MS2	31819829
	No matches	No matches	No matches	No matches		No MS2	26828683
	No matches	No matches	No matches	No matches		No MS2	303165.21
	No matches	No matches	Multiple ma	No matches		No MS2	20148518
	No matches	No matches	No matches	No matches		No MS2	22947007
	No matches	No matches	No matches	Single match		No MS2	25260494
	No matches	No matches	Single match	No matches		No MS2	20315271
	No matches	No matches	No matches	No matches		DDA for pref	26054890
	No matches	No matches	No matches	No matches		No MS2	7780127.9
	No matches	No matches	No matches	No matches		No MS2	24849315
	No matches	No matches	No matches	No matches		No MS2	19253509
	No matches	No matches	No matches	No matches		No MS2	31637976
	No matches	No matches	No matches	No matches		No MS2	1797577.1
	No matches	No matches	No matches	No matches		No MS2	1169639
	No matches	No matches	No matches	No matches		No MS2	11808066
	No matches	No matches	No matches	No matches		No MS2	22771358
	No matches	No matches	No matches	No matches		No MS2	2598686.2
	No matches	No matches	No matches	No matches		No MS2	31542195
	No matches	No matches	No matches	No matches		No MS2	16646685
	No matches	No matches	No matches	No matches		No MS2	21168931
	No matches	No matches	No matches	Multiple ma		No MS2	22443874
	No matches	No matches	No matches	No matches		No MS2	31480031
	No matches	No matches	No matches	Single match		No MS2	29585574
	No matches	No matches	Multiple ma	No matches		No MS2	8054680.9
	No matches	No matches	No matches	No matches		No MS2	22365275
	No matches	No matches	No matches	No matches		No MS2	553017.65
	No matches	No matches	Multiple ma	No matches		No MS2	31357833
	No matches	No matches	No matches	No matches		No MS2	18243559
	No matches	No matches	No matches	No matches		No MS2	31285491
	No matches	No matches	No matches	No matches		No MS2	25921170
	No matches	No matches	No matches	No matches		DDA for othe	15561791

	No matches	No matches	No matches	No matches		No MS2	15049128
	No matches	No matches	No matches	No matches		DDA for othe	31190518
	No matches	No matches	No matches	No matches		No MS2	6873227.2
	No matches	No matches	No matches	No matches		No MS2	1871838.2
	No matches	No matches	No matches	No matches		No MS2	24376682
	No matches	No matches	Single match	No matches		No MS2	22689124
	No matches	No matches	No matches	No matches		No MS2	31111909
	No matches	No matches	No matches	No matches		No MS2	879216.53
	Multiple ma	No matches	No matches	No matches		No MS2	27870260
	No matches	No matches	No matches	No matches		No MS2	31056855
	Multiple ma	No matches	Single match	No matches		No MS2	30997927
	No matches	No matches	No matches	No matches		No MS2	18032287
	No matches	No matches	No matches	No matches		No MS2	2957741
	No matches	No matches	No matches	No matches		No MS2	14147501
	No matches	No matches	No matches	No matches		No MS2	29226697
	No matches	No matches	No matches	No matches		No MS2	25385103
	No matches	No matches	No matches	Single match		No MS2	2637718.7
	No matches	No matches	No matches	No matches		No MS2	2780139.1
	No matches	No matches	No matches	No matches		No MS2	30912947
	No matches	No matches	No matches	Multiple ma		No MS2	15893030
	No matches	No matches	No matches	No matches		No MS2	30873592
	No matches	No matches	No matches	No matches		No MS2	30823052
	No matches	No matches	Multiple ma	No matches		No MS2	22761243
	No matches	No matches	No matches	No matches		No MS2	7246462.7
	No matches	No matches	No matches	No matches		DDA for pref	26923412
	No matches	No matches	No matches	No matches		No MS2	19795632
	No matches	No matches	No matches	No matches		No MS2	1249700
	No matches	No matches	No matches	No matches		No MS2	30199925
	No matches	No matches	No matches	No matches		No MS2	18450772
	No matches	No matches	No matches	No matches		No MS2	9514846.7
	No matches	No matches	No matches	No matches		No MS2	26672349
	No matches	No matches	No matches	No matches		No MS2	20677137
	No matches	No matches	No matches	No matches		No MS2	5963274.7
	No matches	No matches	No matches	No matches		DDA for pref	12936482
	No matches	No matches	No matches	No matches		No MS2	906293.61
	No matches	No matches	No matches	No matches		No MS2	29952476
	No matches	No matches	No matches	No matches		No MS2	667230.63
	No matches	No matches	No matches	No matches		No MS2	1042939.5
	No matches	No matches	No matches	No matches		No MS2	26345788
	No matches	No matches	No matches	No matches		No MS2	30452263
	No matches	No matches	No matches	No matches		No MS2	24135437
	No matches	Single match	Multiple ma	Single match		No MS2	28969380
	No matches	No matches	No matches	No matches		No MS2	29815904
	No matches	No matches	No matches	Single match		No MS2	19329227
	No matches	No matches	No matches	No matches		No MS2	2691847.7
	No matches	No matches	No matches	No matches		No MS2	16632803
	No matches	No matches	No matches	No matches		DDA for pref	11609749
	No matches	No matches	No matches	No matches		No MS2	20370201
	No matches	No matches	Multiple ma	Multiple ma		No MS2	25600479
	No matches	No matches	No matches	No matches		No MS2	22412104

	No matches	Single match	No matches	No matches		No MS2	8276501.4
	No matches	No matches	No matches	No matches		DDA for pref	22047470
	No matches	Single match	No matches	No matches		No MS2	28043430
	No matches	No matches	No matches	No matches		No MS2	26157027
	No matches	No matches	No matches	No matches		No MS2	12000650
	No matches	No matches	Single match	Single match		No MS2	20332523
	No matches	No matches	No matches	No matches		No MS2	14850317
	No matches	No matches	No matches	No matches		DDA for pref	29970179
	No matches	No matches	No matches	No matches		No MS2	21245785
	No matches	No matches	No matches	No matches		No MS2	23632723
	No matches	No matches	No matches	No matches		No MS2	14908141
	No matches	No matches	No matches	No matches		No MS2	22357501
	No matches	No matches	No matches	No matches		No MS2	2800312.6
	No matches	No matches	No matches	No matches		No MS2	20295536
	No matches	No matches	No matches	No matches		No MS2	29925015
	No matches	No matches	Multiple ma	No matches		No MS2	28986874
	No matches	No matches	No matches	No matches		No MS2	18267458
	No matches	No matches	No matches	No matches		No MS2	16027751
	No matches	No matches	No matches	No matches		No MS2	12977589
	No matches	No matches	Single match	No matches		No MS2	23162210
	No matches	No matches	No matches	No matches		No MS2	24456798
	No matches	No matches	No matches	No matches		No MS2	25826571
	No matches	No matches	No matches	No matches		DDA for othe	28924225
	No matches	No matches	No matches	No matches		No MS2	11616273
	No matches	No matches	No matches	No matches		No MS2	883413.23
	No matches	No matches	No matches	No matches		No MS2	11363537
	No matches	No matches	No matches	No matches		No MS2	28441510
	No matches	No matches	No matches	No matches		No MS2	19626754
	Multiple ma	No matches	Single match	No matches		No MS2	16946149
	No matches	No matches	No matches	Single match		No MS2	2589969.4
	No matches	No matches	No matches	No matches		No MS2	1185790.5
	No matches	No matches	No matches	No matches		No MS2	29724787
	No matches	Single match	No matches	No matches		No MS2	25758688
	No matches	Single match	No matches	No matches		No MS2	21058371
	No matches	No matches	No matches	No matches		No MS2	21730457
	Multiple ma	Single match	Multiple ma	No matches		No MS2	19179903
	No matches	No matches	No matches	No matches		No MS2	28146100
	No matches	No matches	No matches	No matches		No MS2	888098.23
	No matches	No matches	No matches	No matches		No MS2	21368570
	No matches	No matches	No matches	No matches		No MS2	22472375
	No matches	No matches	No matches	No matches		No MS2	16810111
	No matches	No matches	No matches	No matches		No MS2	21392061
	No matches	No matches	No matches	No matches		No MS2	29551527
	No matches	No matches	Multiple ma	No matches		No MS2	25908862
	No matches	No matches	No matches	No matches		No MS2	26575177
	No matches	No matches	No matches	No matches		No MS2	25101626
	No matches	No matches	No matches	No matches		No MS2	29503042
	No matches	No matches	Multiple ma	No matches		No MS2	12266756
	No matches	No matches	No matches	No matches		No MS2	1090348.5
	No matches	No matches	No matches	No matches		No MS2	28616101

	No matches	No matches	No matches	No matches		No MS2	23408597
	No matches	No matches	No matches	No matches		No MS2	29374206
	No matches	No matches	No matches	No matches		No MS2	29365487
	No matches	No matches	Single match	No matches		No MS2	16084675
	No matches	No matches	No matches	No matches		No MS2	29298785
	No matches	No matches	No matches	No matches		DDA for pref	18812628
	No matches	No matches	No matches	No matches		No MS2	29263567
	No matches	No matches	No matches	No matches		No MS2	24552276
	No matches	No matches	Multiple ma	No matches		DDA for pref	29238786
	No matches	No matches	Multiple ma	No matches		No MS2	16090324
	No matches	No matches	No matches	No matches		No MS2	23157424
	No matches	No matches	Multiple ma	No matches		No MS2	18351662
	No matches	No matches	No matches	No matches		No MS2	12108293
	Single match	No matches	No matches	No matches		DDA for pref	15719826
	No matches	No matches	Single match	No matches		No MS2	11931906
	Multiple ma	No matches	No matches	No matches		No MS2	1973432.2
	No matches	No matches	No matches	No matches		No MS2	19360628
	No matches	Single match	No matches	No matches		No MS2	14332910
	No matches	No matches	Multiple ma	Multiple ma		DDA for pref	29057395
	No matches	No matches	No matches	No matches		No MS2	1283273
	No matches	No matches	No matches	No matches		No MS2	18983328
	No matches	No matches	No matches	No matches		No MS2	22606116
	No matches	No matches	No matches	No matches		No MS2	20740658
	No matches	No matches	Single match	No matches		No MS2	20223099
	No matches	No matches	Multiple ma	Single match		No MS2	21712756
	No matches	No matches	No matches	No matches		No MS2	24160972
	No matches	No matches	No matches	No matches		No MS2	15834569
	No matches	No matches	No matches	No matches		DDA for othe	22713919
	No matches	No matches	No matches	No matches		DDA for pref	23640669
	No matches	No matches	No matches	No matches		No MS2	14558876
	No matches	No matches	No matches	No matches		No MS2	5113279.1
	No matches	No matches	No matches	No matches		No MS2	18658208
	No matches	No matches	No matches	No matches		No MS2	21793538
	No matches	No matches	No matches	No matches		No MS2	25171974
	No matches	No matches	No matches	No matches		No MS2	28903273
	No matches	No matches	Single match	No matches		No MS2	15973536
	No matches	No matches	No matches	No matches		No MS2	23212632
	No matches	No matches	No matches	No matches		No MS2	7801785.2
	No matches	No matches	No matches	No matches		No MS2	14878175
	No matches	No matches	No matches	No matches		No MS2	1680214.9
	No matches	No matches	No matches	No matches		No MS2	27919222
	No matches	No matches	No matches	No matches		DDA for pref	28819167
	No matches	No matches	No matches	No matches		No MS2	18260954
	No matches	No matches	No matches	No matches		No MS2	19462503
	No matches	No matches	No matches	No matches		No MS2	16888352
	No matches	No matches	No matches	No matches		No MS2	25016893
	No matches	No matches	No matches	No matches		No MS2	28726477
	No matches	No matches	No matches	No matches		No MS2	20389703
	No matches	No matches	Multiple ma	No matches		No MS2	22373249
	Multiple ma	No matches	No matches	No matches		No MS2	10718907

	No matches	No matches	No matches	No matches		No MS2	2957408.8
	No matches	No matches	No matches	No matches		No MS2	24442647
	No matches	No matches	No matches	No matches		No MS2	12682013
	No matches	No matches	No matches	No matches		DDA for pref	23991896
	No matches	No matches	No matches	No matches		No MS2	28613829
	No matches	No matches	No matches	No matches		No MS2	12783615
	No matches	No matches	Multiple ma	No matches		No MS2	25771923
	No matches	No matches	No matches	No matches		No MS2	16966744
	No matches	No matches	No matches	No matches		No MS2	24358801
	No matches	No matches	No matches	No matches		No MS2	1247007.2
	No matches	No matches	No matches	No matches		No MS2	28516846
	No matches	No matches	No matches	No matches		No MS2	20226128
	Multiple ma	No matches	No matches	No matches		DDA for pref	26345106
	No matches	No matches	No matches	No matches		No MS2	28478512
	No matches	No matches	No matches	No matches		No MS2	21988298
	Multiple ma	No matches	No matches	No matches		No MS2	28454787
	Multiple ma	No matches	Single match	No matches		No MS2	15072432
	No matches	No matches	No matches	No matches		No MS2	22733684
	No matches	No matches	No matches	No matches		No MS2	24098196
	No matches	No matches	No matches	No matches		No MS2	28384300
	No matches	No matches	No matches	No matches		No MS2	2272891.3
	No matches	No matches	No matches	No matches		No MS2	17104031
	No matches	No matches	No matches	No matches		No MS2	28353060
	No matches	No matches	No matches	No matches		No MS2	28349312
	No matches	No matches	No matches	No matches		No MS2	23780717
	No matches	No matches	No matches	No matches		No MS2	27469306
	No matches	No matches	No matches	No matches		No MS2	1043405.6
	No matches	No matches	No matches	No matches		No MS2	28288363
	No matches	No matches	No matches	No matches		No MS2	19702827
	No matches	No matches	No matches	No matches		No MS2	19924835
	No matches	No matches	No matches	No matches		No MS2	17442859
	No matches	No matches	Single match	No matches		No MS2	21778582
	No matches	No matches	No matches	No matches		No MS2	4758440.9
	No matches	No matches	No matches	No matches		DDA for pref	17887467
	No matches	No matches	No matches	No matches		No MS2	1530234.8
	No matches	No matches	No matches	No matches		No MS2	13634952
	No matches	No matches	No matches	No matches		No MS2	24298933
	No matches	No matches	No matches	No matches		DDA for pref	12640932
	No matches	No matches	No matches	Single match		No MS2	19327700
	Multiple ma	No matches	No matches	No matches		No MS2	22944065
	No matches	No matches	No matches	No matches		No MS2	27936569
	No matches	No matches	No matches	No matches		DDA for pref	311161.59
	No matches	No matches	No matches	No matches		No MS2	27854521
	No matches	No matches	No matches	No matches		No MS2	27838782
	Multiple ma	No matches	No matches	No matches		No MS2	23836724
	No matches	No matches	Single match	No matches		No MS2	24642955
	No matches	No matches	No matches	No matches		No MS2	27711879
	No matches	No matches	No matches	No matches		No MS2	24549653
	No matches	No matches	No matches	No matches		No MS2	18251146
	Multiple ma	No matches	No matches	No matches		No MS2	21679187

	No matches	No matches	Single match	No matches		No MS2	25520771
	No matches	No matches	No matches	No matches		No MS2	20104488
	No matches	No matches	No matches	No matches		No MS2	8343516.4
	Multiple ma	No matches	No matches	No matches		No MS2	27593376
	No matches	No matches	No matches	No matches		No MS2	18477437
	No matches	No matches	No matches	No matches		No MS2	11481555
	Multiple ma	Single match	Multiple ma	No matches		No MS2	484375.71
	No matches	No matches	No matches	No matches		No MS2	22274982
	No matches	No matches	No matches	No matches		No MS2	20769901
	No matches	No matches	No matches	No matches		No MS2	9114970.1
	No matches	No matches	No matches	No matches		No MS2	3360909.4
	No matches	No matches	No matches	No matches		No MS2	1439144.1
	No matches	No matches	Multiple ma	No matches		No MS2	17885142
	No matches	No matches	No matches	No matches		DDA for pref	11254822
	No matches	No matches	No matches	No matches		No MS2	27358650
	No matches	No matches	No matches	No matches		DDA for pref	2916719.7
	No matches	No matches	No matches	No matches		DDA for pref	22923122
	No matches	No matches	No matches	No matches		No MS2	3862972.8
	No matches	No matches	No matches	No matches		No MS2	1137096.9
	No matches	No matches	No matches	No matches		No MS2	16466532
	No matches	No matches	Multiple ma	Multiple ma		No MS2	18507012
	Multiple ma	No matches	No matches	No matches		No MS2	21764028
	No matches	No matches	No matches	No matches		No MS2	27227213
	No matches	No matches	No matches	No matches		No MS2	19663096
	No matches	No matches	No matches	No matches		DDA for pref	11527848
	No matches	No matches	No matches	No matches		No MS2	17101269
	No matches	No matches	Single match	No matches		No MS2	9333280
	No matches	Single match	Multiple ma	No matches		No MS2	9490719.4
	No matches	No matches	No matches	No matches		No MS2	22816230
	Multiple ma	No matches	No matches	No matches		No MS2	5726207.6
	No matches	No matches	No matches	No matches		No MS2	27106661
	No matches	No matches	No matches	No matches		No MS2	27064231
	No matches	No matches	No matches	No matches		No MS2	22542424
	Multiple ma	No matches	Single match	No matches		No MS2	20470330
	No matches	No matches	No matches	No matches		No MS2	15868382
	No matches	No matches	No matches	No matches		No MS2	1675118.7
	No matches	No matches	No matches	No matches		No MS2	14560721
	No matches	No matches	No matches	No matches		No MS2	14232105
	No matches	No matches	No matches	No matches		No MS2	26923568
	No matches	No matches	No matches	No matches		No MS2	12518712
	No matches	No matches	No matches	No matches		No MS2	21727175
	No matches	No matches	No matches	No matches		No MS2	10943282
	No matches	No matches	No matches	No matches		No MS2	16003531
	No matches	No matches	Multiple ma	Single match		DDA for pref	19851914
	No matches	Multiple ma	Single match	No matches		No MS2	9730933.2
	No matches	No matches	No matches	No matches		No MS2	7979260.8
	No matches	No matches	No matches	No matches		No MS2	22808214
	No matches	No matches	Multiple ma	No matches		No MS2	2545883
	No matches	No matches	No matches	No matches		No MS2	18103146
	No matches	No matches	No matches	No matches		No MS2	20030207

	No matches	No matches	No matches	No matches		No MS2	15409588
	No matches	No matches	No matches	No matches		No MS2	25880890
	No matches	No matches	No matches	Single match		No MS2	14519930
	No matches	No matches	No matches	No matches		DDA for othe	19210222
	No matches	No matches	No matches	No matches		No MS2	26614644
	No matches	No matches	No matches	No matches		No MS2	9132353.5
	Multiple ma	No matches	No matches	No matches		No MS2	26490166
	Multiple ma	No matches	Multiple ma	No matches		No MS2	16285765
	No matches	No matches	No matches	No matches		No MS2	26482511
	No matches	No matches	No matches	No matches		No MS2	22544465
	No matches	No matches	No matches	No matches		No MS2	13276808
	No matches	No matches	No matches	Single match		No MS2	15783749
	No matches	No matches	No matches	No matches		DDA for pref	22706650
	No matches	No matches	No matches	No matches		No MS2	3150950.3
	Multiple ma	No matches	No matches	No matches		No MS2	19669393
	No matches	No matches	No matches	No matches		No MS2	7428498.9
	Multiple ma	No matches	Single match	Single match		No MS2	6116597.6
	Multiple ma	No matches	No matches	No matches		No MS2	17920832
	No matches	No matches	No matches	No matches		No MS2	1699690.2
	No matches	No matches	No matches	No matches		DDA for othe	26340872
	No matches	No matches	No matches	No matches		No MS2	21724310
	No matches	No matches	No matches	No matches		No MS2	14620854
	No matches	No matches	No matches	No matches		DDA for pref	19156723
	No matches	No matches	No matches	No matches		No MS2	744946.58
	No matches	No matches	No matches	No matches		No MS2	26298127
	No matches	No matches	No matches	No matches		No MS2	25774577
	Multiple ma	Single match	Multiple ma	No matches		No MS2	15475593
	No matches	No matches	No matches	No matches		No MS2	26201254
	No matches	No matches	No matches	Single match		No MS2	7543739.6
	No matches	No matches	No matches	No matches		No MS2	289236.93
	No matches	No matches	No matches	No matches		No MS2	26186504
	No matches	No matches	No matches	No matches		No MS2	23386135
	No matches	No matches	No matches	No matches		No MS2	22731521
	No matches	No matches	No matches	No matches		No MS2	17963934
	No matches	No matches	No matches	No matches		No MS2	13654920
	No matches	No matches	No matches	No matches		No MS2	2384865
	No matches	No matches	No matches	No matches		No MS2	14583431
	No matches	No matches	No matches	No matches		No MS2	11012599
	Single match	No matches	No matches	No matches		No MS2	17009036
	No matches	No matches	No matches	No matches		No MS2	26061073
	No matches	No matches	No matches	No matches		No MS2	16567773
	Multiple ma	No matches	Multiple ma	Single match		No MS2	14656310
	No matches	No matches	No matches	No matches		No MS2	16751405
	No matches	No matches	No matches	Multiple ma		DDA for pref	26024291
	No matches	No matches	No matches	No matches		No MS2	14430427
	No matches	No matches	No matches	No matches		No MS2	4948534.9
	No matches	No matches	Multiple ma	Single match		No MS2	26002760
	Multiple ma	No matches	No matches	No matches		No MS2	10099173
	No matches	Single match	No matches	No matches		No MS2	25952078
	No matches	No matches	No matches	No matches		No MS2	1048467.6

	No matches	No matches	No matches	No matches		No MS2	25900878
	No matches	No matches	No matches	No matches		No MS2	23113737
	No matches	No matches	No matches	No matches		No MS2	5360716
	No matches	No matches	No matches	No matches		No MS2	25851186
	No matches	No matches	No matches	No matches		No MS2	13585220
	No matches	No matches	No matches	No matches		No MS2	18150183
	No matches	No matches	No matches	No matches		No MS2	21366152
	No matches	No matches	No matches	No matches		No MS2	25808394
	No matches	No matches	No matches	No matches		No MS2	10464562
	No matches	No matches	No matches	No matches		No MS2	20125646
	No matches	No matches	No matches	No matches		No MS2	13843035
	No matches	No matches	No matches	No matches		No MS2	25704387
	No matches	No matches	No matches	No matches		No MS2	19250793
	No matches	No matches	No matches	No matches		No MS2	25680481
	No matches	No matches	No matches	No matches		No MS2	25672520
	No matches	No matches	No matches	No matches		No MS2	2247688.7
	No matches	No matches	Single match	No matches		No MS2	1319806.4
	No matches	No matches	Single match	Single match		No MS2	983645.68
	No matches	No matches	No matches	No matches		No MS2	25618916
	No matches	No matches	No matches	No matches		DDA for othe	22238375
	Multiple ma	No matches	Single match	Single match		No MS2	25604290
	No matches	No matches	No matches	No matches		No MS2	15660584
	No matches	No matches	No matches	No matches		DDA for pref	17458367
	No matches	No matches	No matches	No matches		No MS2	22184468
	No matches	No matches	No matches	No matches		No MS2	225650.94
	No matches	Single match	No matches	No matches		No MS2	23322400
	No matches	No matches	No matches	No matches		No MS2	20874311
	No matches	No matches	No matches	No matches		No MS2	17840665
	No matches	No matches	No matches	No matches		No MS2	25384871
	No matches	No matches	Single match	No matches		No MS2	10333982
	No matches	No matches	Single match	No matches		No MS2	13806169
	No matches	No matches	No matches	No matches		No MS2	20573680
	Multiple ma	No matches	No matches	No matches		No MS2	12778746
	No matches	No matches	No matches	No matches		No MS2	24412219
	No matches	No matches	No matches	No matches		No MS2	16662895
	No matches	No matches	No matches	No matches		No MS2	16574743
	No matches	No matches	Single match	No matches		No MS2	17730714
	No matches	No matches	No matches	No matches		No MS2	21650035
	No matches	No matches	No matches	No matches		No MS2	25262824
	No matches	No matches	No matches	No matches		No MS2	25246338
	No matches	No matches	No matches	No matches		No MS2	20676885
	No matches	No matches	Single match	No matches		No MS2	15295243
	No matches	No matches	No matches	No matches		No MS2	2560985.7
	No matches	No matches	No matches	No matches		No MS2	24915491
	No matches	No matches	No matches	No matches		No MS2	5752166.4
	No matches	No matches	No matches	No matches		No MS2	15438149
	No matches	No matches	No matches	No matches		No MS2	22455364
	No matches	No matches	No matches	No matches		No MS2	16040993
	No matches	No matches	No matches	No matches		No MS2	20216290
	No matches	No matches	No matches	No matches		No MS2	23549525

	No matches	No matches	Single match	No matches		No MS2	13897087
	No matches	No matches	No matches	No matches		No MS2	23929170
	No matches	No matches	No matches	No matches		No MS2	18346527
	No matches	No matches	Multiple ma	No matches		No MS2	13812564
	Multiple ma	No matches	No matches	No matches		No MS2	25058264
	No matches	No matches	No matches	No matches		No MS2	16522915
	No matches	No matches	No matches	No matches		No MS2	16519760
	No matches	No matches	No matches	No matches		No MS2	23024092
	No matches	No matches	No matches	No matches		No MS2	8645759.7
	Multiple ma	No matches	No matches	No matches		No MS2	24997791
	No matches	No matches	No matches	No matches		No MS2	24385602
	No matches	No matches	Multiple ma	No matches		No MS2	20966854
	No matches	No matches	No matches	No matches		No MS2	19263221
	No matches	No matches	No matches	No matches		No MS2	12280903
	No matches	No matches	No matches	No matches		No MS2	4854376.6
	No matches	No matches	No matches	No matches		No MS2	21486555
	No matches	No matches	Multiple ma	No matches		No MS2	24902591
	No matches	No matches	No matches	No matches		No MS2	20914589
	No matches	No matches	No matches	No matches		No MS2	12375419
	No matches	No matches	Single match	No matches		No MS2	13571689
	No matches	No matches	No matches	No matches		No MS2	21225785
	No matches	No matches	No matches	No matches		No MS2	24823646
	No matches	No matches	No matches	Single match		No MS2	13579750
	No matches	No matches	No matches	No matches		No MS2	24802526
	No matches	No matches	No matches	No matches		No MS2	24779926
	No matches	No matches	No matches	No matches		No MS2	8589806.8
	No matches	No matches	No matches	No matches		DDA for pref	16894768
	No matches	No matches	No matches	No matches		No MS2	18982756
	No matches	No matches	No matches	No matches		No MS2	1282679
	No matches	No matches	No matches	Single match		No MS2	24693306
	No matches	No matches	Multiple ma	No matches		No MS2	18778847
	No matches	No matches	No matches	No matches		No MS2	24675306
	No matches	No matches	No matches	Single match		No MS2	16994474
	Multiple ma	No matches	No matches	No matches		No MS2	14682820
	No matches	No matches	No matches	No matches		No MS2	10740912
	No matches	Multiple ma	No matches	Multiple ma		No MS2	15386114
	No matches	No matches	No matches	No matches		No MS2	14894941
	No matches	No matches	No matches	No matches		No MS2	15177501
	No matches	No matches	No matches	No matches		No MS2	3650188.6
	No matches	Single match	No matches	No matches		No MS2	13176096
	No matches	No matches	No matches	No matches		No MS2	3860113.5
	Multiple ma	No matches	No matches	No matches		No MS2	6256521.9
	No matches	No matches	No matches	No matches		No MS2	22335027
	No matches	No matches	Single match	No matches		No MS2	15432715
	No matches	No matches	No matches	No matches		DDA for pref	12884128
	No matches	No matches	No matches	No matches		No MS2	12923804
	No matches	No matches	No matches	No matches		No MS2	24409534
	No matches	No matches	No matches	No matches		No MS2	13924174
	No matches	No matches	No matches	No matches		No MS2	10812128
	Multiple ma	No matches	No matches	No matches		No MS2	10818114

	No matches	No matches	No matches	No matches		No MS2	18962975
	No matches	No matches	No matches	No matches		No MS2	20238423
	No matches	No matches	Multiple ma	No matches		No MS2	3920865
	No matches	No matches	Multiple ma	No matches		No MS2	17429305
	No matches	No matches	No matches	No matches		No MS2	16528381
	No matches	No matches	No matches	No matches		No MS2	11929852
	No matches	No matches	No matches	No matches		No MS2	1983869.6
	No matches	No matches	No matches	No matches		No MS2	24205879
	No matches	No matches	No matches	No matches		No MS2	1923493.1
	No matches	No matches	No matches	No matches		No MS2	451824.32
	No matches	No matches	No matches	Multiple ma		No MS2	24161577
	Multiple ma	No matches	No matches	No matches		No MS2	24156581
	No matches	No matches	Single match	No matches		No MS2	14773135
	No matches	No matches	No matches	No matches		No MS2	11187340
	No matches	No matches	Multiple ma	No matches		No MS2	1078844.6
	No matches	No matches	No matches	Single match		No MS2	18471839
	No matches	No matches	Single match	Single match		No MS2	17909571
	No matches	No matches	No matches	No matches		No MS2	6193102.9
	No matches	Single match	No matches	No matches		No MS2	10459488
	No matches	Single match	No matches	No matches		No MS2	7889316.3
	No matches	No matches	No matches	No matches		DDA for pref	24068285
	No matches	No matches	No matches	No matches		No MS2	12731116
	Single match	No matches	No matches	No matches		No MS2	14162242
	No matches	Multiple ma	No matches	No matches		No MS2	21135310
	No matches	No matches	Single match	No matches		DDA for othe	1968618.6
	No matches	No matches	No matches	No matches		No MS2	22923900
	No matches	No matches	No matches	No matches		No MS2	17953036
	Multiple ma	No matches	No matches	No matches		No MS2	13838122
	No matches	No matches	No matches	No matches		No MS2	6165614.2
	No matches	No matches	Single match	No matches		No MS2	23954690
	No matches	No matches	No matches	No matches		DDA for pref	20311064
	No matches	No matches	Single match	No matches		No MS2	20383275
	No matches	No matches	No matches	No matches		No MS2	23851318
	No matches	No matches	No matches	No matches		No MS2	18765289
	Multiple ma	No matches	No matches	No matches		No MS2	3880535.3
	No matches	No matches	No matches	No matches		No MS2	11365777
	No matches	No matches	No matches	No matches		No MS2	13003886
	No matches	No matches	No matches	No matches		No MS2	6213754.1
	No matches	No matches	No matches	No matches		No MS2	965881.02
	Multiple ma	No matches	No matches	No matches		No MS2	23381502
	No matches	No matches	No matches	No matches		No MS2	23749321
	No matches	Single match	No matches	No matches		DDA for othe	13055411
	No matches	No matches	Single match	No matches		No MS2	21675230
	No matches	No matches	No matches	No matches		No MS2	942640.09
	No matches	No matches	No matches	No matches		No MS2	2080545.8
	No matches	No matches	No matches	No matches		No MS2	17745076
	No matches	No matches	No matches	No matches		No MS2	17745076
	No matches	No matches	No matches	No matches		No MS2	21643993
	No matches	No matches	No matches	No matches		No MS2	23625122
	Multiple ma	No matches	No matches	No matches		No MS2	7429503.2

	No matches	No matches	No matches	No matches		No MS2	20011743
	No matches	No matches	No matches	No matches		No MS2	9395501.9
	No matches	No matches	No matches	No matches		No MS2	9605923.2
	No matches	No matches	No matches	No matches		No MS2	21889553
	No matches	No matches	No matches	No matches		No MS2	13602167
	No matches	No matches	No matches	No matches		DDA for pref	23493336
	No matches	No matches	No matches	No matches		No MS2	23444543
	Multiple ma	No matches	Single match	No matches		DDA for pref	16778141
	No matches	No matches	No matches	No matches		No MS2	17641929
	No matches	No matches	No matches	No matches		No MS2	12061401
	No matches	No matches	Single match	No matches		No MS2	13917392
	Single match	No matches	No matches	No matches		No MS2	12165931
	No matches	No matches	No matches	No matches		No MS2	20845558
	No matches	Single match	Single match	No matches		No MS2	12663761
	No matches	No matches	No matches	No matches		No MS2	10644497
	No matches	No matches	No matches	No matches		No MS2	21359377
	No matches	Single match	No matches	Single match		No MS2	17446374
	Multiple ma	Single match	Multiple ma	No matches		No MS2	18740982
	No matches	No matches	No matches	No matches		No MS2	14478546
	No matches	No matches	No matches	No matches		No MS2	23309451
	No matches	No matches	No matches	No matches		No MS2	9903126.2
	No matches	No matches	No matches	No matches		No MS2	23297261
	No matches	No matches	No matches	No matches		No MS2	20367643
	No matches	No matches	No matches	No matches		No MS2	23262353
	No matches	No matches	No matches	No matches		No MS2	2730346.7
	No matches	No matches	No matches	No matches		No MS2	11672963
	No matches	No matches	No matches	No matches		No MS2	8416951.8
	No matches	No matches	No matches	No matches		No MS2	8411653.9
	No matches	No matches	No matches	No matches		No MS2	608114.99
	No matches	No matches	No matches	No matches		DDA for othe	10125162
	No matches	No matches	No matches	No matches		No MS2	20591728
	No matches	No matches	No matches	No matches		No MS2	18170300
	No matches	No matches	No matches	No matches		No MS2	12447917
	No matches	No matches	No matches	No matches		No MS2	23063726
	No matches	No matches	No matches	No matches		No MS2	22023243
	No matches	No matches	No matches	No matches		No MS2	23059565
	No matches	No matches	Multiple ma	No matches		No MS2	23055912
	No matches	No matches	No matches	No matches		No MS2	23052516
	No matches	No matches	No matches	No matches		No MS2	9303169.8
	No matches	No matches	No matches	No matches		No MS2	16574108
	No matches	No matches	No matches	No matches		No MS2	19404177
	No matches	No matches	No matches	No matches		No MS2	10745732
	No matches	No matches	No matches	No matches		No MS2	22923786
	No matches	No matches	No matches	No matches		No MS2	14655014
	No matches	No matches	No matches	No matches		No MS2	12519816
	No matches	No matches	No matches	No matches		No MS2	15844399
	Single match	No matches	No matches	No matches		No MS2	7212650.2
	No matches	No matches	No matches	No matches		No MS2	13275616
	No matches	No matches	No matches	No matches		No MS2	10654500
	No matches	No matches	No matches	No matches		No MS2	19746065

	Single match	No matches	No matches	No matches		No MS2	21656623
	No matches	No matches	No matches	No matches		DDA for pref	13261113
	No matches	No matches	No matches	No matches		No MS2	1518294.8
	No matches	No matches	Single match	No matches		No MS2	11161870
	No matches	No matches	No matches	No matches		No MS2	11122497
	No matches	No matches	No matches	No matches		No MS2	8204476.6
	No matches	No matches	No matches	No matches		No MS2	10547719
	No matches	Single match	No matches	No matches		No MS2	18690051
	No matches	No matches	Single match	Single match		No MS2	888041.83
	No matches	No matches	No matches	No matches		No MS2	11822133
	No matches	No matches	No matches	No matches		No MS2	19468430
	No matches	No matches	No matches	No matches		No MS2	11246104
	No matches	No matches	Multiple ma	Single match		No MS2	9101552.4
	No matches	No matches	No matches	No matches		No MS2	19048269
	No matches	No matches	No matches	No matches		No MS2	17476121
	No matches	No matches	No matches	No matches		No MS2	12767633
	No matches	No matches	No matches	No matches		No MS2	14606883
	No matches	No matches	No matches	No matches		No MS2	22681152
	No matches	No matches	No matches	No matches		No MS2	19344847
	No matches	No matches	No matches	Single match		No MS2	12954972
	No matches	No matches	No matches	No matches		DDA for pref	15990795
	No matches	No matches	No matches	No matches		No MS2	18600238
	No matches	No matches	No matches	No matches		No MS2	18286635
	No matches	No matches	No matches	No matches		No MS2	3713085.2
	No matches	No matches	No matches	No matches		No MS2	9652523.2
	No matches	No matches	No matches	No matches		No MS2	4477699.2
	Multiple ma	No matches	No matches	No matches		No MS2	12348401
	No matches	No matches	No matches	No matches		No MS2	425713.67
	No matches	No matches	No matches	No matches		No MS2	16138222
	No matches	No matches	Single match	No matches		DDA for pref	16985437
	No matches	No matches	Single match	No matches		No MS2	22395466
	No matches	No matches	Single match	No matches		No MS2	13701013
	No matches	No matches	No matches	No matches		No MS2	6231434.3
	Single match	No matches	No matches	No matches		No MS2	22356261
	No matches	No matches	No matches	No matches		No MS2	9140892.7
	No matches	No matches	No matches	No matches		No MS2	22346354
	No matches	No matches	No matches	No matches		No MS2	21679869
	No matches	No matches	No matches	No matches		No MS2	15307907
	No matches	No matches	No matches	No matches		No MS2	13594158
	No matches	No matches	No matches	No matches		No MS2	14561110
	No matches	No matches	No matches	No matches		No MS2	15839918
	No matches	No matches	No matches	No matches		No MS2	22242674
	No matches	No matches	No matches	No matches		No MS2	13030084
	No matches	No matches	No matches	No matches		No MS2	10893977
	No matches	No matches	No matches	No matches		No MS2	20056005
	No matches	No matches	No matches	No matches		No MS2	14001171
	No matches	Single match	No matches	No matches		No MS2	10308361
	No matches	Single match	No matches	Single match		No MS2	12487891
	No matches	No matches	No matches	No matches		No MS2	18034528
	Multiple ma	No matches	No matches	No matches		No MS2	16188137

	No matches	No matches	No matches	No matches		No MS2	10502994
	No matches	No matches	No matches	No matches		No MS2	14532272
	No matches	No matches	No matches	No matches		No MS2	22102089
	No matches	No matches	No matches	No matches		No MS2	22045358
	No matches	No matches	No matches	No matches		No MS2	21087320
	Multiple ma	No matches	Single match	No matches		No MS2	6450417.9
	No matches	No matches	No matches	No matches		No MS2	22009936
	No matches	No matches	No matches	No matches		No MS2	22006306
	No matches	No matches	No matches	No matches		No MS2	475740.29
	Multiple ma	No matches	No matches	No matches		No MS2	12579728
	No matches	No matches	No matches	No matches		No MS2	21934441
	No matches	No matches	No matches	No matches		No MS2	14880586
	No matches	No matches	No matches	No matches		No MS2	870589.45
	No matches	No matches	No matches	No matches		No MS2	5663072.9
	No matches	No matches	No matches	No matches		DDA for pref	19252608
	Multiple ma	No matches	No matches	No matches		No MS2	20637339
	No matches	No matches	No matches	No matches		No MS2	20338327
	No matches	No matches	No matches	No matches		No MS2	17149694
	No matches	No matches	No matches	No matches		No MS2	12527699
	No matches	No matches	No matches	Single match		No MS2	19396161
	No matches	No matches	No matches	No matches		No MS2	3414574.9
	No matches	No matches	Multiple ma	No matches		No MS2	15460253
	No matches	No matches	No matches	No matches		No MS2	20498562
	No matches	No matches	No matches	No matches		No MS2	19431758
	Single match	No matches	No matches	No matches		No MS2	16535962
	No matches	No matches	No matches	No matches		No MS2	9574411.5
	No matches	No matches	No matches	No matches		No MS2	21720429
	No matches	No matches	No matches	No matches		No MS2	3228635.9
	No matches	No matches	Single match	No matches		No MS2	21701071
	No matches	No matches	No matches	No matches		No MS2	21684770
	No matches	No matches	No matches	No matches		No MS2	4598455.6
	No matches	No matches	No matches	No matches		No MS2	21674392
	No matches	No matches	No matches	Single match		No MS2	8797962
	No matches	No matches	No matches	No matches		No MS2	21399603
	No matches	No matches	No matches	No matches		No MS2	10515534
	No matches	No matches	No matches	No matches		No MS2	15919020
	No matches	No matches	No matches	No matches		No MS2	21621401
	No matches	No matches	No matches	No matches		No MS2	2374381.4
	No matches	No matches	No matches	No matches		No MS2	16024010
	No matches	No matches	Single match	No matches		No MS2	17956471
	No matches	No matches	No matches	No matches		No MS2	21577644
	No matches	No matches	No matches	No matches		No MS2	21543120
	No matches	No matches	No matches	No matches		No MS2	12553794
	No matches	No matches	No matches	No matches		No MS2	21533868
	No matches	No matches	No matches	No matches		No MS2	1243385.8
	No matches	No matches	No matches	No matches		No MS2	11070561
	No matches	No matches	No matches	No matches		No MS2	8360536.9
	No matches	Multiple ma	No matches	No matches		No MS2	13042594
	No matches	No matches	No matches	No matches		No MS2	1370856
	No matches	No matches	No matches	No matches		No MS2	16088784

	No matches	No matches	No matches	No matches		No MS2	15338953
	No matches	No matches	No matches	No matches		No MS2	13626709
	No matches	No matches	No matches	No matches		No MS2	21323493
	No matches	No matches	No matches	No matches		No MS2	21320273
	No matches	No matches	No matches	No matches		No MS2	20306022
	No matches	No matches	Single match	No matches		No MS2	19400046
	No matches	No matches	No matches	No matches		No MS2	21253141
	No matches	No matches	No matches	Single match		No MS2	2287333
	No matches	No matches	No matches	No matches		No MS2	12612451
	No matches	No matches	No matches	No matches		No MS2	11606108
	No matches	No matches	No matches	No matches		No MS2	11590628
	Multiple ma	No matches	No matches	No matches		DDA for pref	21202253
	No matches	No matches	No matches	No matches		No MS2	19962276
	No matches	No matches	No matches	No matches		No MS2	16278063
	No matches	No matches	Single match	Multiple ma		No MS2	8160748
	No matches	No matches	No matches	No matches		No MS2	17200269
	No matches	No matches	No matches	No matches		No MS2	15629859
	No matches	No matches	No matches	No matches		No MS2	20201378
	No matches	No matches	No matches	No matches		No MS2	21147167
	No matches	No matches	Multiple ma	No matches		No MS2	18673840
	No matches	No matches	No matches	No matches		No MS2	21133176
	No matches	No matches	No matches	No matches		No MS2	21117801
	No matches	No matches	No matches	No matches		No MS2	17463294
	No matches	No matches	Single match	No matches		No MS2	21078778
	No matches	No matches	No matches	No matches		No MS2	15132288
	No matches	No matches	No matches	No matches		No MS2	878978.28
	No matches	No matches	No matches	No matches		No MS2	17829933
	No matches	No matches	Single match	No matches		No MS2	18063722
	No matches	No matches	No matches	No matches		No MS2	16319396
	No matches	No matches	No matches	No matches		No MS2	17683498
	No matches	No matches	No matches	No matches		No MS2	13582959
	No matches	No matches	No matches	No matches		No MS2	18038706
	No matches	No matches	No matches	No matches		No MS2	5105335.9
	No matches	No matches	No matches	No matches		No MS2	584298.55
	No matches	No matches	No matches	No matches		No MS2	20690010
	No matches	No matches	No matches	No matches		No MS2	11805974
	No matches	No matches	No matches	No matches		No MS2	17753505
	No matches	No matches	No matches	No matches		No MS2	9280237.8
	No matches	No matches	No matches	No matches		No MS2	7760287.7
	No matches	No matches	No matches	No matches		No MS2	18081171
	Multiple ma	No matches	No matches	No matches		No MS2	20843442
	No matches	No matches	No matches	No matches		No MS2	3684157.3
	No matches	No matches	No matches	No matches		No MS2	15027536
	No matches	No matches	No matches	No matches		No MS2	11654587
	No matches	No matches	No matches	No matches		No MS2	11074348
	No matches	No matches	No matches	No matches		No MS2	976263.19
	No matches	No matches	No matches	No matches		No MS2	8880828
	No matches	No matches	No matches	No matches		No MS2	16191732
	No matches	No matches	No matches	No matches		No MS2	949624.99
	No matches	No matches	No matches	No matches		No MS2	12946180

	No matches	No matches	Multiple ma	No matches			No MS2	20587446
	No matches	No matches	No matches	No matches			No MS2	20586568
	No matches	No matches	No matches	No matches			No MS2	14325216
	No matches	No matches	No matches	No matches			No MS2	16068898
	No matches	No matches	No matches	No matches			No MS2	2019986.9
	No matches	No matches	No matches	No matches			No MS2	10971466
	No matches	No matches	No matches	No matches			No MS2	16091081
	No matches	No matches	No matches	No matches			No MS2	13519797
	No matches	No matches	No matches	No matches			No MS2	20489071
	Multiple ma	No matches	No matches	No matches			No MS2	20083370
	No matches	No matches	No matches	No matches			DDA for pref	5643429.3
	No matches	No matches	No matches	No matches			No MS2	17142409
	No matches	No matches	No matches	No matches			No MS2	16175290
	No matches	No matches	No matches	No matches			DDA for othe	16851276
	No matches	No matches	Single match	No matches			No MS2	16532440
	No matches	No matches	Single match	Single match			No MS2	13961323
	No matches	No matches	No matches	No matches			No MS2	308453.81
	No matches	No matches	No matches	No matches			No MS2	10520794
	No matches	No matches	No matches	No matches			DDA for othe	15182435
	No matches	No matches	No matches	No matches			No MS2	12693917
	No matches	Single match	No matches	No matches			No MS2	20349917
	No matches	No matches	No matches	No matches			No MS2	12434368
	No matches	No matches	No matches	No matches			No MS2	12104553
	No matches	No matches	No matches	No matches			No MS2	14314016
	No matches	No matches	No matches	No matches			No MS2	12482743
	Multiple ma	No matches	No matches	No matches			No MS2	14203013
	No matches	No matches	No matches	No matches			No MS2	8253080
	Multiple ma	No matches	No matches	No matches			No MS2	14431993
	No matches	No matches	Single match	No matches			No MS2	20312520
	No matches	No matches	No matches	No matches			No MS2	16794181
	No matches	No matches	No matches	No matches			No MS2	7712048.5
	No matches	No matches	Single match	Single match			No MS2	14014162
	No matches	No matches	No matches	No matches			No MS2	17488532
	No matches	No matches	Multiple ma	Single match			No MS2	15988040
	Single match	No matches	No matches	No matches			No MS2	20258364
	No matches	No matches	No matches	Multiple ma			No MS2	17720674
	No matches	No matches	No matches	No matches			No MS2	5619609.1
	No matches	No matches	No matches	No matches			No MS2	20003473
	No matches	No matches	Single match	No matches			No MS2	10895484
	No matches	No matches	No matches	No matches			No MS2	4818042.2
	Multiple ma	No matches	Single match	No matches			No MS2	9748074
	No matches	No matches	No matches	No matches			No MS2	20109622
	No matches	No matches	No matches	No matches			No MS2	4950544.2
	No matches	Single match	No matches	Single match			DDA for pref	16440682
	No matches	No matches	No matches	No matches			No MS2	18374309
	No matches	No matches	No matches	No matches			No MS2	5453592.7
	Multiple ma	No matches	No matches	No matches			No MS2	12761747
	No matches	No matches	No matches	No matches			No MS2	16796682
	Multiple ma	No matches	No matches	No matches			No MS2	13508184
	No matches	No matches	No matches	No matches			No MS2	4233216.7

	No matches	No matches	No matches	No matches		No MS2	11368837
	No matches	No matches	No matches	No matches		No MS2	9665535.7
	No matches	No matches	No matches	No matches		No MS2	18255815
	No matches	No matches	No matches	No matches		No MS2	16013889
	No matches	No matches	No matches	No matches		No MS2	14697269
	No matches	No matches	No matches	No matches		No MS2	19955247
	No matches	No matches	No matches	No matches		No MS2	19947320
	No matches	No matches	Single match	No matches		No MS2	7102806.6
	No matches	No matches	Multiple ma	No matches		No MS2	10435152
	No matches	No matches	No matches	No matches		No MS2	19892821
	No matches	No matches	No matches	No matches		No MS2	1623945.9
	No matches	No matches	Single match	Single match		No MS2	11897324
	No matches	No matches	No matches	No matches		No MS2	9296411.5
	No matches	No matches	No matches	No matches		No MS2	14367774
	No matches	No matches	No matches	No matches		No MS2	19794868
	No matches	No matches	No matches	No matches		No MS2	19761056
	No matches	No matches	No matches	No matches		No MS2	7098325.4
	No matches	Single match	No matches	Single match		No MS2	19745963
	No matches	No matches	No matches	No matches		No MS2	9065929.2
	No matches	No matches	No matches	No matches		No MS2	19730845
	No matches	No matches	No matches	No matches		No MS2	9348973.4
	No matches	No matches	No matches	No matches		No MS2	14115979
	No matches	No matches	No matches	No matches		No MS2	18797292
	No matches	No matches	No matches	No matches		No MS2	17570289
	No matches	No matches	No matches	No matches		No MS2	17111116
	No matches	No matches	No matches	No matches		No MS2	8934660.1
	No matches	No matches	No matches	No matches		No MS2	14479721
	No matches	No matches	No matches	No matches		No MS2	17128820
	No matches	No matches	Single match	No matches		No MS2	12673420
	No matches	No matches	No matches	No matches		No MS2	15078184
	No matches	No matches	No matches	No matches		No MS2	7782442.7
	No matches	No matches	No matches	No matches		No MS2	11779211
	No matches	No matches	No matches	No matches		No MS2	11918404
	Multiple ma	Single match	Multiple ma	No matches		No MS2	3604339
	No matches	Single match	No matches	No matches		No MS2	1083776.4
	No matches	No matches	No matches	No matches		No MS2	16175664
	No matches	No matches	No matches	No matches		No MS2	19459444
	No matches	No matches	No matches	No matches		No MS2	12180476
	No matches	No matches	No matches	No matches		No MS2	16331913
	No matches	No matches	No matches	No matches		No MS2	6715353.9
	No matches	No matches	No matches	No matches		No MS2	11113734
	No matches	No matches	No matches	No matches		No MS2	14557446
	No matches	No matches	No matches	No matches		No MS2	19424777
	No matches	No matches	No matches	No matches		No MS2	19402420
	No matches	No matches	Single match	No matches		No MS2	19400571
	No matches	No matches	No matches	No matches		No MS2	19372062
	No matches	No matches	No matches	Single match		No MS2	3094132.2
	No matches	No matches	No matches	No matches		No MS2	15933019
	No matches	No matches	No matches	No matches		No MS2	1336461.3
	No matches	No matches	No matches	No matches		No MS2	16556009

	Multiple ma	No matches	No matches	No matches		No MS2	14436067
	No matches	No matches	No matches	No matches		No MS2	8063404.7
	No matches	No matches	Single match	No matches		No MS2	17988864
	No matches	No matches	Single match	No matches		No MS2	11607601
	No matches	No matches	No matches	No matches		No MS2	19297781
	No matches	No matches	No matches	No matches		No MS2	19291440
	No matches	No matches	No matches	No matches		No MS2	4543309.1
	No matches	No matches	No matches	No matches		No MS2	4543309.1
	Multiple ma	No matches	Single match	No matches		No MS2	19272128
	No matches	No matches	No matches	No matches		No MS2	2065781.1
	No matches	No matches	Single match	No matches		No MS2	8839167.4
	No matches	No matches	No matches	No matches		No MS2	10845274
	No matches	No matches	No matches	No matches		No MS2	10537053
	No matches	No matches	No matches	No matches		No MS2	12668206
	Multiple ma	No matches	Multiple ma	No matches		No MS2	7600406.3
	No matches	No matches	No matches	Single match		No MS2	11608199
	No matches	No matches	No matches	No matches		No MS2	14236658
	No matches	No matches	No matches	No matches		No MS2	8370125.4
	No matches	No matches	No matches	No matches		No MS2	19107717
	No matches	No matches	No matches	No matches		No MS2	3285453.3
	No matches	No matches	Single match	No matches		No MS2	4211645.9
	No matches	No matches	No matches	No matches		No MS2	9721708.7
	No matches	No matches	No matches	No matches		No MS2	16408915
	No matches	No matches	No matches	No matches		No MS2	11118564
	No matches	No matches	No matches	No matches		No MS2	6240540.8
	No matches	No matches	No matches	No matches		No MS2	10638469
	No matches	No matches	No matches	No matches		No MS2	16810560
	No matches	No matches	No matches	No matches		No MS2	11847035
	No matches	No matches	No matches	No matches		No MS2	19001572
	No matches	No matches	No matches	No matches		No MS2	18992161
	No matches	No matches	No matches	No matches		No MS2	18987781
	Multiple ma	No matches	Single match	No matches		No MS2	18980426
	No matches	No matches	No matches	No matches		No MS2	1337417.8
	No matches	Single match	Single match	No matches		DDA for pref	2503529.4
	No matches	No matches	No matches	No matches		No MS2	18021747
	No matches	No matches	No matches	No matches		No MS2	18925145
	Multiple ma	No matches	No matches	No matches		DDA for pref	18924356
	No matches	No matches	No matches	No matches		No MS2	18914766
	No matches	No matches	No matches	No matches		No MS2	14786681
	No matches	No matches	No matches	No matches		DDA for othe	16236833
	No matches	No matches	No matches	No matches		No MS2	17652026
	No matches	No matches	Single match	No matches		No MS2	12892777
	No matches	No matches	No matches	No matches		No MS2	5321119.6
	No matches	No matches	Multiple ma	Single match		No MS2	18854183
	No matches	No matches	No matches	No matches		No MS2	18845691
	No matches	No matches	No matches	No matches		No MS2	15305229
	No matches	No matches	No matches	Single match		No MS2	10431602
	No matches	No matches	No matches	No matches		No MS2	14640139
	No matches	No matches	No matches	No matches		No MS2	11099203
	No matches	No matches	No matches	No matches		No MS2	14990496

	No matches	No matches	No matches	No matches		No MS2	15210414
	No matches	No matches	No matches	No matches		No MS2	12685183
	No matches	No matches	No matches	No matches		No MS2	13078418
	No matches	No matches	No matches	No matches		No MS2	9471107.5
	No matches	No matches	No matches	No matches		No MS2	12395489
	No matches	No matches	No matches	Single match		DDA for pref	14590631
	No matches	No matches	No matches	No matches		No MS2	15147219
	No matches	No matches	No matches	No matches		No MS2	14462092
	No matches	No matches	No matches	No matches		No MS2	14680396
	No matches	No matches	No matches	No matches		No MS2	17616361
	No matches	No matches	No matches	No matches		No MS2	18709487
	No matches	No matches	No matches	No matches		No MS2	10573863
	No matches	No matches	No matches	No matches		No MS2	16434136
	No matches	No matches	No matches	No matches		DDA for pref	11231977
	No matches	No matches	No matches	No matches		No MS2	18678415
	No matches	No matches	No matches	No matches		No MS2	11803813
	Single match	No matches	No matches	No matches		No MS2	18649320
	No matches	No matches	Multiple ma	Multiple ma		No MS2	8343190.6
	No matches	No matches	No matches	No matches		No MS2	18633460
	No matches	No matches	No matches	No matches		No MS2	14037834
	No matches	No matches	No matches	No matches		No MS2	16375523
	No matches	No matches	No matches	No matches		No MS2	10802423
	Single match	No matches	No matches	No matches		No MS2	16617322
	No matches	No matches	No matches	No matches		No MS2	7578904.4
	No matches	No matches	No matches	No matches		No MS2	12085902
	No matches	No matches	No matches	No matches		No MS2	8840817.8
	No matches	No matches	No matches	No matches		No MS2	1299500.4
	No matches	No matches	No matches	No matches		No MS2	18561224
	No matches	No matches	No matches	No matches		No MS2	18432369
	No matches	No matches	No matches	No matches		DDA for pref	18516024
	No matches	No matches	No matches	No matches		No MS2	16942364
	Multiple ma	No matches	No matches	No matches		No MS2	17822488
	No matches	No matches	No matches	No matches		No MS2	6431644.9
	No matches	No matches	No matches	No matches		No MS2	18130984
	Multiple ma	No matches	Single match	No matches		No MS2	11173266
	No matches	No matches	No matches	No matches		No MS2	1215045.2
	No matches	No matches	No matches	No matches		No MS2	18416570
	No matches	No matches	No matches	No matches		No MS2	641329.4
	No matches	No matches	No matches	No matches		No MS2	834543.98
	No matches	No matches	No matches	No matches		No MS2	12806456
	Multiple ma	No matches	No matches	No matches		No MS2	6118318.2
	Multiple ma	No matches	No matches	No matches		No MS2	14160146
	No matches	No matches	No matches	No matches		No MS2	9826741.1
	No matches	Single match	No matches	Single match		No MS2	18368651
	No matches	No matches	No matches	No matches		No MS2	2606081.3
	No matches	No matches	No matches	No matches		No MS2	414772.4
	No matches	No matches	No matches	No matches		No MS2	1720491.9
	No matches	No matches	No matches	No matches		No MS2	18355251
	No matches	No matches	No matches	No matches		No MS2	18333697
	Multiple ma	Single match	Multiple ma	No matches		No MS2	17786011

	No matches	No matches	Multiple ma	No matches		No MS2	5189181.1
	No matches	No matches	No matches	No matches		No MS2	304992.68
	No matches	No matches	No matches	No matches		No MS2	18295366
	No matches	No matches	No matches	No matches		No MS2	13506084
	No matches	No matches	No matches	No matches		No MS2	18272640
	No matches	No matches	No matches	No matches		No MS2	18267165
	Multiple ma	No matches	No matches	No matches		No MS2	18248372
	No matches	No matches	No matches	No matches		No MS2	18227073
	No matches	No matches	No matches	No matches		No MS2	11256255
	No matches	No matches	No matches	No matches		No MS2	10217940
	No matches	No matches	No matches	No matches		No MS2	16303578
	No matches	No matches	No matches	No matches		No MS2	11172635
	No matches	No matches	No matches	No matches		No MS2	8986212.7
	No matches	No matches	No matches	No matches		No MS2	3045549.2
	No matches	No matches	Single match	No matches		DDA for pref	18167469
	Multiple ma	No matches	No matches	No matches		No MS2	15605059
	No matches	No matches	No matches	Single match		No MS2	14102345
	No matches	No matches	No matches	No matches		No MS2	2862231.4
	No matches	No matches	No matches	No matches		No MS2	18141420
	No matches	No matches	No matches	No matches		No MS2	18132479
	No matches	No matches	No matches	No matches		No MS2	12187222
	No matches	No matches	No matches	No matches		No MS2	18104181
	Multiple ma	No matches	No matches	No matches		No MS2	14839330
	No matches	No matches	No matches	No matches		No MS2	1710300.1
	No matches	No matches	No matches	No matches		No MS2	18054267
	No matches	No matches	No matches	No matches		No MS2	14113546
	No matches	No matches	No matches	No matches		No MS2	18022702
	No matches	No matches	No matches	No matches		No MS2	18014302
	No matches	No matches	No matches	No matches		No MS2	9906246.3
	No matches	No matches	No matches	No matches		No MS2	12127442
	No matches	No matches	No matches	No matches		No MS2	10891017
	No matches	No matches	No matches	No matches		No MS2	3716306.3
	No matches	No matches	Multiple ma	No matches		DDA for othe	9214384.9
	No matches	No matches	No matches	No matches		No MS2	17917181
	No matches	No matches	No matches	No matches		No MS2	13176286
	No matches	No matches	No matches	No matches		No MS2	17841165
	No matches	No matches	Single match	No matches		No MS2	15094992
	No matches	No matches	No matches	No matches		No MS2	11957702
	No matches	No matches	No matches	No matches		No MS2	13069641
	No matches	No matches	No matches	No matches		No MS2	13033711
	No matches	No matches	No matches	No matches		No MS2	6611776.7
	No matches	No matches	No matches	No matches		No MS2	14383845
	No matches	No matches	No matches	No matches		No MS2	15643854
	No matches	No matches	No matches	No matches		No MS2	13827749
	No matches	Single match	Multiple ma	No matches		No MS2	14193248
	No matches	No matches	No matches	No matches		No MS2	4379804.3
	No matches	No matches	No matches	No matches		No MS2	10078906
	No matches	No matches	No matches	No matches		No MS2	17674785
	No matches	No matches	No matches	No matches		No MS2	1516937.7
	No matches	No matches	No matches	No matches		No MS2	17655983

	No matches	No matches	Single match	No matches		No MS2	17635612
	No matches	No matches	No matches	No matches		DDA for pref	15365051
	No matches	No matches	No matches	No matches		No MS2	11257250
	No matches	No matches	No matches	No matches		No MS2	8069807.2
	No matches	No matches	No matches	No matches		No MS2	13509551
	No matches	No matches	No matches	No matches		No MS2	17579009
	No matches	No matches	No matches	No matches		No MS2	17569900
	No matches	No matches	No matches	No matches		No MS2	10780439
	No matches	No matches	No matches	No matches		No MS2	17533125
	No matches	No matches	No matches	No matches		No MS2	13811642
	No matches	No matches	No matches	No matches		No MS2	12127894
	No matches	No matches	No matches	No matches		No MS2	17528154
	No matches	No matches	No matches	No matches		No MS2	17498540
	No matches	No matches	No matches	No matches		No MS2	5956154.9
	No matches	No matches	No matches	No matches		No MS2	15454054
	No matches	No matches	No matches	No matches		No MS2	7482019.5
	No matches	No matches	No matches	No matches		No MS2	17440598
	No matches	No matches	No matches	No matches		No MS2	945087.67
	No matches	No matches	No matches	No matches		No MS2	17431035
	No matches	No matches	No matches	No matches		No MS2	17423911
	No matches	No matches	No matches	No matches		No MS2	8295048.7
	No matches	No matches	No matches	No matches		No MS2	3868968.8
	No matches	No matches	No matches	No matches		No MS2	1896553.2
	No matches	No matches	No matches	No matches		No MS2	16946218
	No matches	No matches	No matches	No matches		No MS2	9402790.2
	No matches	No matches	No matches	No matches		No MS2	1407791.8
	No matches	Single match	No matches	No matches		No MS2	13857623
	No matches	No matches	No matches	No matches		No MS2	15661241
	No matches	No matches	Single match	No matches		No MS2	1777817.7
	No matches	No matches	No matches	No matches		No MS2	4263340.4
	No matches	No matches	No matches	No matches		No MS2	560691.81
	No matches	No matches	No matches	No matches		No MS2	12751887
	No matches	No matches	No matches	No matches		No MS2	17226618
	No matches	No matches	No matches	No matches		No MS2	17218288
	No matches	No matches	No matches	No matches		No MS2	8018119.6
	No matches	No matches	No matches	No matches		No MS2	17202166
	No matches	No matches	Single match	No matches		No MS2	17199291
	No matches	No matches	No matches	No matches		No MS2	15326696
	No matches	No matches	No matches	No matches		No MS2	7083794.8
	No matches	No matches	Multiple ma	Multiple ma		No MS2	11981973
	No matches	No matches	No matches	No matches		No MS2	10012908
	No matches	No matches	No matches	Single match		No MS2	7789628.4
	No matches	No matches	No matches	No matches		No MS2	7373755.5
	No matches	No matches	No matches	No matches		No MS2	14855212
	No matches	No matches	No matches	Single match		No MS2	9613078
	No matches	No matches	No matches	No matches		No MS2	17128836
	Multiple ma	No matches	Single match	No matches		No MS2	9734469
	No matches	No matches	No matches	No matches		No MS2	10502819
	No matches	No matches	No matches	No matches		No MS2	2327982.6
	No matches	No matches	No matches	No matches		No MS2	17052843

	No matches	No matches	No matches	No matches		No MS2	8462322.4
	No matches	No matches	No matches	No matches		No MS2	11258086
	No matches	No matches	No matches	No matches		No MS2	17007817
	No matches	No matches	Multiple ma	No matches		No MS2	11909590
	No matches	No matches	No matches	No matches		No MS2	16980882
	No matches	No matches	No matches	No matches		No MS2	16462084
	No matches	No matches	No matches	No matches		No MS2	9955171.4
	No matches	No matches	No matches	No matches		No MS2	10476432
	No matches	No matches	No matches	No matches		No MS2	1041449.7
	No matches	No matches	No matches	No matches		No MS2	11980575
	No matches	No matches	No matches	No matches		No MS2	7672243.2
	No matches	No matches	Single match	No matches		No MS2	10200636
	No matches	No matches	Multiple ma	No matches		No MS2	12202298
	No matches	No matches	No matches	No matches		No MS2	12202298
	No matches	No matches	No matches	No matches		No MS2	7990995.1
	No matches	No matches	No matches	No matches		No MS2	16918155
	Multiple ma	No matches	No matches	No matches		DDA for pref	16809220
	No matches	No matches	No matches	No matches		No MS2	16892043
	No matches	No matches	No matches	No matches		No MS2	8792214.3
	Multiple ma	No matches	No matches	No matches		No MS2	7958342.6
	Multiple ma	No matches	No matches	No matches		No MS2	14054567
	No matches	No matches	No matches	No matches		No MS2	12545155
	No matches	No matches	No matches	No matches		No MS2	5672164.1
	No matches	No matches	No matches	No matches		No MS2	14420652
	Multiple ma	No matches	Single match	No matches		No MS2	13268143
	No matches	Single match	Single match	No matches		No MS2	1413447
	Multiple ma	No matches	Single match	No matches		DDA for pref	13292026
	Multiple ma	No matches	No matches	No matches		No MS2	16755720
	No matches	No matches	No matches	No matches		No MS2	5801476.2
	No matches	No matches	No matches	No matches		No MS2	14776209
	No matches	No matches	No matches	No matches		No MS2	1156449.7
	No matches	No matches	Multiple ma	No matches		No MS2	9404940.5
	Multiple ma	No matches	No matches	No matches		No MS2	8025112.5
	No matches	No matches	No matches	No matches		No MS2	16675840
	No matches	No matches	No matches	No matches		No MS2	16655344
	No matches	No matches	No matches	No matches		No MS2	11772614
	No matches	No matches	No matches	No matches		No MS2	15189190
	No matches	No matches	No matches	No matches		No MS2	338122.24
	No matches	No matches	No matches	No matches		No MS2	6184670.2
	No matches	No matches	No matches	No matches		No MS2	2512118.6
	No matches	No matches	No matches	No matches		No MS2	1061831.1
	No matches	No matches	No matches	No matches		No MS2	16609305
	No matches	No matches	Single match	No matches		No MS2	16595933
	Multiple ma	No matches	Single match	No matches		No MS2	16595933
	No matches	No matches	No matches	No matches		No MS2	16580785
	No matches	No matches	No matches	No matches		No MS2	16575124
	No matches	No matches	No matches	No matches		No MS2	6116534.7
	No matches	No matches	No matches	No matches		No MS2	11901550
	No matches	No matches	No matches	No matches		No MS2	14509074
	No matches	No matches	No matches	No matches		No MS2	2094052.6

	No matches	Multiple ma	No matches	Single match		No MS2	14888279
	No matches	No matches	No matches	No matches		No MS2	15754204
	No matches	No matches	No matches	No matches		No MS2	16499071
	No matches	No matches	No matches	No matches		No MS2	12067134
	No matches	No matches	No matches	No matches		No MS2	13864443
	No matches	No matches	No matches	No matches		No MS2	5572592.3
	No matches	No matches	No matches	No matches		No MS2	10932420
	No matches	No matches	No matches	No matches		No MS2	11087739
	No matches	No matches	No matches	No matches		No MS2	13551705
	No matches	No matches	No matches	No matches		No MS2	8877764.4
	No matches	No matches	No matches	No matches		No MS2	5455885.4
	No matches	No matches	No matches	No matches		No MS2	12355354
	No matches	No matches	Multiple ma	No matches		No MS2	12925628
	No matches	No matches	No matches	No matches		No MS2	7953549.2
	No matches	No matches	No matches	No matches		No MS2	3074803.8
	No matches	No matches	No matches	No matches		No MS2	1078475.5
	No matches	No matches	No matches	No matches		No MS2	16325582
	Multiple ma	Single match	Multiple ma	No matches		DDA for pref	12260892
	No matches	No matches	No matches	No matches		No MS2	11115724
	No matches	No matches	No matches	No matches		No MS2	1360434.8
	No matches	No matches	Single match	Single match		No MS2	13233426
	No matches	No matches	No matches	No matches		No MS2	12319544
	No matches	No matches	No matches	No matches		No MS2	12946137
	No matches	No matches	No matches	No matches		No MS2	7142139.7
	No matches	No matches	No matches	No matches		No MS2	16262457
	No matches	No matches	No matches	No matches		No MS2	11977947
	No matches	No matches	No matches	No matches		No MS2	2069969.4
	No matches	No matches	Single match	Single match		No MS2	8000963.8
	Multiple ma	No matches	No matches	No matches		No MS2	9736020.8
	No matches	No matches	No matches	No matches		No MS2	12279171
	No matches	No matches	No matches	No matches		No MS2	8186658.8
	No matches	Single match	No matches	No matches		No MS2	2265820
	No matches	No matches	No matches	No matches		No MS2	16154741
	No matches	No matches	No matches	No matches		No MS2	7378244.6
	No matches	No matches	No matches	No matches		No MS2	8802930.3
	No matches	No matches	No matches	No matches		No MS2	527496.8
	No matches	No matches	No matches	No matches		No MS2	2235942.5
	Multiple ma	No matches	Single match	No matches		No MS2	12891861
	No matches	No matches	No matches	No matches		No MS2	7829228.1
	No matches	No matches	No matches	No matches		No MS2	274952.72
	No matches	No matches	No matches	No matches		No MS2	12657562
	No matches	No matches	No matches	No matches		No MS2	11380248
	No matches	No matches	No matches	No matches		No MS2	11480740
	No matches	No matches	No matches	No matches		No MS2	13880255
	No matches	No matches	No matches	Single match		No MS2	16021700
	No matches	No matches	No matches	No matches		No MS2	9774480.8
	No matches	No matches	No matches	Single match		No MS2	11365944
	No matches	No matches	No matches	No matches		No MS2	16003789
	No matches	No matches	No matches	No matches		No MS2	8981995.9
	Multiple ma	No matches	No matches	No matches		No MS2	15999694

	No matches	No matches	No matches	No matches		No MS2	15988663
	No matches	No matches	No matches	No matches		No MS2	2711380.5
	No matches	No matches	No matches	No matches		No MS2	15968053
	No matches	No matches	Single match	No matches		No MS2	9031981.1
	No matches	No matches	No matches	No matches		No MS2	14526238
	No matches	No matches	No matches	No matches		No MS2	15900294
	No matches	No matches	No matches	No matches		No MS2	15852961
	No matches	No matches	No matches	No matches		No MS2	15850534
	No matches	No matches	No matches	No matches		No MS2	10236789
	No matches	No matches	No matches	No matches		No MS2	15791967
	No matches	Single match	Multiple ma	No matches		No MS2	15765644
	No matches	No matches	No matches	No matches		No MS2	13708217
	No matches	No matches	No matches	No matches		No MS2	8816475.3
	No matches	No matches	No matches	No matches		No MS2	15753035
	No matches	No matches	No matches	No matches		No MS2	15741562
	No matches	No matches	No matches	Single match		No MS2	6148776.7
	No matches	No matches	No matches	No matches		No MS2	9482923.4
	No matches	No matches	No matches	No matches		No MS2	1238308.7
	No matches	No matches	No matches	No matches		No MS2	9159938.4
	No matches	No matches	No matches	No matches		No MS2	15655105
	No matches	No matches	No matches	No matches		No MS2	3465472.7
	No matches	No matches	No matches	No matches		No MS2	5374009.2
	No matches	No matches	No matches	No matches		No MS2	10829413
	No matches	No matches	No matches	No matches		No MS2	15619157
	No matches	No matches	No matches	No matches		No MS2	12890976
	No matches	No matches	No matches	No matches		No MS2	8955813.1
	No matches	No matches	No matches	No matches		No MS2	2411938.4
	Multiple ma	No matches	No matches	No matches		No MS2	11404552
	No matches	No matches	No matches	No matches		No MS2	2721381
	No matches	No matches	No matches	No matches		No MS2	1031903.6
	No matches	No matches	No matches	No matches		No MS2	9817081.2
	No matches	No matches	No matches	No matches		No MS2	316338.3
	No matches	No matches	No matches	No matches		No MS2	11382046
	No matches	No matches	No matches	No matches		No MS2	11118256
	No matches	No matches	No matches	No matches		No MS2	10239377
	No matches	No matches	No matches	Single match		No MS2	5036723.1
	No matches	No matches	No matches	No matches		No MS2	9475155.3
	No matches	No matches	No matches	No matches		No MS2	9277538
	No matches	No matches	No matches	No matches		No MS2	13428293
	No matches	No matches	No matches	No matches		DDA for pref	7803460.4
	No matches	No matches	No matches	No matches		No MS2	8839062.1
	No matches	No matches	No matches	No matches		No MS2	15426665
	No matches	No matches	No matches	No matches		No MS2	14203013
	No matches	No matches	No matches	No matches		No MS2	4620082.3
	No matches	No matches	Single match	Single match		No MS2	11134985
	No matches	No matches	No matches	No matches		No MS2	12043526
	No matches	No matches	No matches	No matches		No MS2	15392488
	No matches	No matches	No matches	No matches		No MS2	15150789
	No matches	No matches	No matches	No matches		No MS2	11434316
	No matches	No matches	No matches	No matches		No MS2	13754328

	No matches	No matches	Multiple ma	No matches		No MS2	10466924
	No matches	No matches	Single match	No matches		No MS2	10598645
	Single match	No matches	No matches	No matches		No MS2	268646.92
	No matches	No matches	No matches	No matches		No MS2	8387035.3
	No matches	No matches	Multiple ma	No matches		No MS2	2346576.6
	No matches	No matches	No matches	No matches		No MS2	13755727
	No matches	No matches	No matches	No matches		No MS2	15345321
	No matches	No matches	No matches	Single match		No MS2	6378612.9
	No matches	No matches	No matches	No matches		No MS2	10217485
	No matches	No matches	No matches	No matches		No MS2	1845949.1
	No matches	No matches	No matches	No matches		No MS2	2690636.7
	No matches	No matches	No matches	No matches		No MS2	8091416
	No matches	No matches	No matches	No matches		No MS2	15293030
	No matches	No matches	No matches	No matches		No MS2	11794040
	No matches	No matches	No matches	No matches		No MS2	12274095
	No matches	No matches	No matches	No matches		No MS2	371166.92
	No matches	No matches	No matches	No matches		No MS2	3355971.8
	No matches	No matches	No matches	No matches		No MS2	9727849.4
	No matches	No matches	No matches	No matches		No MS2	11110263
	No matches	No matches	No matches	No matches		No MS2	15225010
	No matches	No matches	Multiple ma	No matches		No MS2	10737748
	No matches	No matches	No matches	No matches		DDA for othe	12578660
	No matches	No matches	No matches	No matches		No MS2	10959573
	No matches	No matches	No matches	No matches		No MS2	12740128
	No matches	No matches	No matches	No matches		No MS2	15143181
	Multiple ma	No matches	No matches	No matches		No MS2	15126068
	No matches	No matches	No matches	No matches		No MS2	10977565
	No matches	No matches	No matches	No matches		No MS2	1335534.4
	No matches	No matches	No matches	No matches		No MS2	15100097
	No matches	No matches	No matches	No matches		No MS2	11058074
	Multiple ma	No matches	No matches	No matches		No MS2	5417125.1
	No matches	No matches	No matches	No matches		No MS2	13979519
	No matches	No matches	No matches	No matches		No MS2	7891856.2
	No matches	No matches	No matches	No matches		No MS2	5640470.3
	No matches	No matches	No matches	No matches		No MS2	12216978
	No matches	No matches	No matches	No matches		No MS2	15027904
	No matches	No matches	No matches	No matches		No MS2	4417501.4
	No matches	No matches	No matches	No matches		No MS2	8801390
	No matches	No matches	No matches	No matches		No MS2	15005033
	No matches	No matches	No matches	No matches		No MS2	13170748
	No matches	No matches	No matches	No matches		No MS2	8061617.3
	No matches	No matches	Multiple ma	No matches		DDA for othe	11641163
	No matches	No matches	Single match	No matches		No MS2	10442542
	No matches	No matches	No matches	No matches		No MS2	14571105
	No matches	Single match	No matches	No matches		No MS2	1440918
	No matches	No matches	No matches	No matches		No MS2	10690599
	No matches	No matches	No matches	No matches		No MS2	12780813
	No matches	No matches	No matches	Multiple ma		No MS2	11792567
	No matches	No matches	No matches	No matches		No MS2	3485548.8
	No matches	No matches	No matches	No matches		No MS2	3485548.8

	No matches	No matches	No matches	No matches		No MS2	11366513
	No matches	No matches	No matches	No matches		No MS2	9373425.6
	No matches	No matches	No matches	No matches		No MS2	8856827.8
	Multiple ma	No matches	No matches	No matches		No MS2	14139086
	Multiple ma	No matches	No matches	No matches		No MS2	14144975
	No matches	No matches	No matches	No matches		No MS2	14390527
	No matches	No matches	No matches	No matches		No MS2	8412927.2
	No matches	No matches	No matches	No matches		No MS2	10919308
	No matches	No matches	No matches	No matches		No MS2	2560818.3
	No matches	No matches	Single match	No matches		No MS2	3880457.2
	No matches	No matches	Multiple ma	Multiple ma		No MS2	11302886
	No matches	No matches	Multiple ma	No matches		No MS2	10466924
	No matches	No matches	No matches	No matches		No MS2	14844385
	No matches	Single match	Multiple ma	Single match		No MS2	7144007.1
	No matches	No matches	No matches	No matches		No MS2	14817695
	No matches	No matches	No matches	No matches		No MS2	11986701
	No matches	No matches	No matches	No matches		No MS2	14805378
	No matches	No matches	No matches	No matches		No MS2	13334405
	No matches	No matches	No matches	No matches		No MS2	8412735.9
	No matches	No matches	No matches	No matches		No MS2	14730931
	No matches	No matches	No matches	No matches		No MS2	6260986.9
	No matches	No matches	No matches	No matches		No MS2	14722669
	No matches	No matches	No matches	No matches		No MS2	14424276
	No matches	No matches	No matches	No matches		No MS2	14652737
	Multiple ma	No matches	No matches	No matches		No MS2	14651971
	No matches	No matches	No matches	No matches		No MS2	9340851.7
	No matches	No matches	No matches	No matches		No MS2	7654459.1
	No matches	No matches	No matches	No matches		No MS2	11745457
	No matches	No matches	No matches	No matches		No MS2	8447965.8
	No matches	No matches	No matches	No matches		No MS2	6813056.7
	No matches	No matches	No matches	Single match		No MS2	8384472
	No matches	No matches	No matches	No matches		No MS2	12116655
	No matches	No matches	No matches	No matches		No MS2	8372747.7
	No matches	No matches	No matches	No matches		No MS2	910875.92
	No matches	No matches	No matches	No matches		No MS2	14547993
	No matches	No matches	No matches	No matches		No MS2	12134792
	No matches	No matches	Multiple ma	Single match		No MS2	14533311
	No matches	No matches	No matches	No matches		No MS2	13927971
	No matches	No matches	No matches	No matches		No MS2	11674919
	No matches	No matches	No matches	No matches		No MS2	14515312
	No matches	No matches	No matches	No matches		No MS2	3823259.9
	Multiple ma	No matches	No matches	No matches		No MS2	10584951
	No matches	No matches	No matches	Single match		No MS2	7426580
	Multiple ma	No matches	Multiple ma	No matches		No MS2	13529644
	No matches	No matches	No matches	No matches		No MS2	5971514.3
	No matches	No matches	No matches	No matches		No MS2	8700348.3
	No matches	Single match	Multiple ma	No matches		No MS2	1027210.2
	No matches	No matches	No matches	No matches		No MS2	12628190
	Multiple ma	No matches	No matches	No matches		No MS2	4283406.9
	No matches	Single match	No matches	No matches		No MS2	14443049

	No matches	No matches	No matches	No matches		No MS2	14438619
	No matches	No matches	No matches	No matches		No MS2	1657349.8
	Single match	No matches	No matches	No matches		No MS2	11525504
	No matches	No matches	Single match	No matches		No MS2	744354.67
	No matches	No matches	No matches	No matches		No MS2	9128260.1
	No matches	No matches	No matches	No matches		No MS2	12029787
	No matches	No matches	No matches	No matches		No MS2	9066591.2
	No matches	No matches	No matches	No matches		No MS2	9800109.9
	No matches	No matches	No matches	No matches		No MS2	10251590
	Multiple ma	No matches	No matches	No matches		No MS2	979551.32
	No matches	No matches	No matches	No matches		No MS2	12789823
	No matches	No matches	No matches	No matches		No MS2	509792.43
	Multiple ma	No matches	No matches	No matches		No MS2	5721259.6
	No matches	No matches	No matches	No matches		No MS2	14343095
	No matches	No matches	Single match	No matches		No MS2	14336582
	No matches	No matches	No matches	No matches		No MS2	8857351.8
	Multiple ma	No matches	No matches	No matches		No MS2	13209316
	No matches	No matches	No matches	No matches		No MS2	10486271
	No matches	No matches	No matches	No matches		No MS2	10529207
	No matches	No matches	No matches	No matches		No MS2	7266252.9
	No matches	No matches	No matches	No matches		No MS2	8612144.7
	No matches	No matches	No matches	No matches		No MS2	335163.21
	No matches	No matches	No matches	Single match		No MS2	4016651.5
	No matches	No matches	No matches	No matches		No MS2	7219950.4
	No matches	No matches	No matches	No matches		No MS2	349948.73
	No matches	No matches	No matches	No matches		No MS2	8624516.6
	No matches	No matches	No matches	No matches		No MS2	12519233
	No matches	No matches	No matches	No matches		No MS2	728432.36
	No matches	No matches	No matches	No matches		No MS2	14126510
	No matches	No matches	No matches	No matches		No MS2	7111247.7
	No matches	No matches	No matches	No matches		No MS2	14112940
	No matches	No matches	No matches	No matches		No MS2	8374453.3
	No matches	No matches	No matches	No matches		No MS2	9387584.6
	No matches	No matches	No matches	No matches		No MS2	14061452
	Multiple ma	No matches	Single match	No matches		No MS2	14047364
	No matches	No matches	No matches	No matches		No MS2	14042225
	No matches	No matches	No matches	No matches		No MS2	11548517
	No matches	No matches	No matches	No matches		No MS2	1533237.4
	No matches	No matches	No matches	No matches		No MS2	7503857.5
	No matches	No matches	No matches	No matches		No MS2	12542816
	No matches	No matches	No matches	No matches		No MS2	7666827.7
	No matches	No matches	No matches	No matches		No MS2	1921186
	No matches	No matches	No matches	No matches		No MS2	4854396.4
	No matches	No matches	No matches	No matches		No MS2	12644713
	No matches	No matches	Multiple ma	No matches		No MS2	9148896.7
	No matches	No matches	No matches	Single match		No MS2	13982927
	No matches	No matches	Single match	No matches		No MS2	7856016
	No matches	No matches	No matches	No matches		No MS2	12190466
	No matches	No matches	No matches	No matches		No MS2	8719787.4
	No matches	No matches	No matches	No matches		No MS2	13960997

	No matches	No matches	No matches	No matches		No MS2	10054737
	No matches	No matches	No matches	No matches		No MS2	9487362.2
	No matches	No matches	No matches	No matches		No MS2	8447057.5
	No matches	No matches	No matches	No matches		No MS2	9225198
	No matches	No matches	No matches	No matches		No MS2	1371007.5
	No matches	No matches	No matches	No matches		No MS2	12847488
	No matches	No matches	Single match	No matches		No MS2	9506458.7
	No matches	No matches	No matches	No matches		No MS2	2079832.3
	Single match	No matches	No matches	No matches		No MS2	9110352.7
	No matches	No matches	No matches	No matches		No MS2	5011694.4
	No matches	No matches	No matches	No matches		DDA for pref	7297215.3
	No matches	No matches	No matches	No matches		No MS2	9554121.3
	No matches	No matches	No matches	No matches		No MS2	6190667.7
	Multiple ma	No matches	No matches	No matches		No MS2	8435271.5
	No matches	No matches	No matches	No matches		No MS2	12891615
	No matches	No matches	No matches	No matches		No MS2	9932043.2
	No matches	No matches	No matches	Single match		No MS2	9956236
	No matches	No matches	Multiple ma	No matches		No MS2	7305316.7
	No matches	No matches	No matches	No matches		No MS2	8654521.5
	No matches	No matches	No matches	No matches		No MS2	9030567.6
	No matches	No matches	No matches	No matches		No MS2	1547050.5
	No matches	No matches	No matches	No matches		No MS2	13733686
	Multiple ma	No matches	No matches	No matches		No MS2	963487.1
	No matches	No matches	No matches	No matches		No MS2	13717552
	Multiple ma	No matches	Multiple ma	No matches		No MS2	13717114
	No matches	No matches	No matches	No matches		No MS2	11486951
	No matches	No matches	No matches	No matches		No MS2	2073978.3
	No matches	No matches	No matches	No matches		No MS2	13675273
	No matches	No matches	No matches	No matches		No MS2	7135981.2
	Multiple ma	No matches	No matches	No matches	62.6	DDA for pref	10974081
	No matches	No matches	No matches	No matches		No MS2	956840.49
	No matches	No matches	No matches	No matches		No MS2	13194314
	No matches	No matches	No matches	No matches		No MS2	10155913
	No matches	No matches	No matches	No matches		No MS2	9566589.4
	No matches	No matches	No matches	No matches		No MS2	8742929.6
	No matches	Single match	Multiple ma	No matches		No MS2	806226.27
	No matches	No matches	No matches	No matches		No MS2	1252378.5
	No matches	No matches	No matches	No matches		No MS2	1481426.4
	No matches	No matches	No matches	No matches		No MS2	13632960
	No matches	No matches	No matches	No matches		No MS2	8796837.7
	No matches	No matches	No matches	No matches		No MS2	1811363.4
	No matches	No matches	No matches	No matches		No MS2	7037067.7
	No matches	No matches	No matches	No matches		No MS2	11466745
	No matches	No matches	No matches	No matches		No MS2	6337047.1
	No matches	No matches	No matches	No matches		No MS2	13593768
	No matches	No matches	No matches	No matches		No MS2	10852254
	No matches	No matches	No matches	No matches		No MS2	9570864.7
	No matches	No matches	No matches	No matches		No MS2	8623617.5
	No matches	No matches	No matches	No matches		No MS2	7123761.4
	No matches	No matches	Multiple ma	Multiple ma		No MS2	10409941

	No matches	No matches	No matches	No matches		No MS2	13542016
	No matches	No matches	No matches	No matches		No MS2	13539758
	No matches	No matches	No matches	No matches		No MS2	5399059
	Single match	No matches	No matches	No matches		No MS2	678588.76
	No matches	No matches	No matches	No matches		No MS2	11012569
	No matches	No matches	No matches	No matches		No MS2	4358113.5
	No matches	No matches	No matches	No matches		No MS2	1250463.5
	No matches	No matches	No matches	No matches		No MS2	10885331
	No matches	Single match	No matches	No matches		No MS2	13498867
	No matches	No matches	No matches	No matches		No MS2	13486568
	No matches	No matches	No matches	No matches		No MS2	11829580
	No matches	No matches	No matches	No matches		No MS2	4193047.3
	No matches	No matches	No matches	No matches		No MS2	3239476.3
	No matches	No matches	No matches	No matches		No MS2	13455974
	No matches	No matches	No matches	No matches		No MS2	13088539
	No matches	No matches	No matches	No matches		No MS2	10239228
	No matches	No matches	No matches	No matches		No MS2	7982738.3
	No matches	No matches	Single match	No matches		No MS2	13423084
	No matches	Single match	No matches	No matches		No MS2	13421700
	No matches	No matches	Multiple ma	No matches		No MS2	11713533
	No matches	No matches	No matches	No matches		No MS2	13414825
	No matches	No matches	No matches	Multiple ma		No MS2	9965294.9
	Multiple ma	No matches	Single match	Single match		No MS2	13393779
	Multiple ma	No matches	No matches	No matches		No MS2	13158550
	No matches	No matches	No matches	No matches		No MS2	9791647.6
	No matches	No matches	No matches	No matches		No MS2	1089115
	No matches	No matches	No matches	No matches		No MS2	11667108
	No matches	No matches	No matches	No matches		No MS2	13273139
	No matches	No matches	Single match	No matches		No MS2	13343098
	No matches	No matches	No matches	No matches		No MS2	2761982.3
	No matches	No matches	No matches	No matches		No MS2	1956938.6
	No matches	No matches	No matches	No matches		No MS2	11051171
	No matches	No matches	Single match	No matches		No MS2	9104723.6
	No matches	No matches	No matches	No matches		No MS2	8675914.7
	No matches	No matches	No matches	No matches		No MS2	9391190.4
	No matches	No matches	No matches	No matches		No MS2	10714718
	No matches	No matches	No matches	No matches		No MS2	9095109.2
	No matches	No matches	No matches	No matches		No MS2	13289136
	No matches	No matches	Multiple ma	No matches		No MS2	9865004.8
	No matches	No matches	Multiple ma	No matches		No MS2	1818599.5
	No matches	No matches	No matches	No matches		No MS2	7533145.9
	No matches	No matches	No matches	No matches		No MS2	13231745
	No matches	Multiple ma	No matches	No matches		No MS2	7020826.1
	No matches	No matches	No matches	No matches		No MS2	1312149.3
	No matches	No matches	No matches	No matches		No MS2	5726143.1
	No matches	No matches	No matches	No matches		No MS2	11935115
	No matches	No matches	No matches	No matches		No MS2	8564017.4
	No matches	No matches	No matches	No matches		No MS2	13192046
	No matches	No matches	No matches	No matches		No MS2	13181930
	No matches	No matches	No matches	No matches		No MS2	10012812

	No matches	No matches	No matches	No matches		No MS2	9346635.2
	No matches	No matches	No matches	No matches		No MS2	13149650
	No matches	No matches	No matches	No matches		No MS2	9047759.6
	Multiple ma	No matches	No matches	No matches		No MS2	10629794
	No matches	No matches	Multiple ma	No matches		No MS2	9358076.5
	No matches	No matches	No matches	No matches		DDA for pref	13131160
	No matches	No matches	No matches	No matches		No MS2	9502732.8
	No matches	No matches	No matches	No matches		No MS2	12682605
	No matches	Single match	Single match	No matches		No MS2	9481760.2
	No matches	No matches	Single match	No matches		No MS2	9712906.9
	No matches	No matches	No matches	No matches		No MS2	7816215.3
	No matches	No matches	No matches	No matches		No MS2	10691677
	No matches	No matches	No matches	No matches		No MS2	7146159.4
	No matches	No matches	No matches	No matches		No MS2	8256844.9
	No matches	No matches	No matches	No matches		No MS2	7720917.7
	No matches	Single match	No matches	No matches		No MS2	5202779.7
	No matches	No matches	Single match	No matches		No MS2	10879884
	No matches	No matches	No matches	No matches		No MS2	1486201.1
	No matches	No matches	No matches	No matches		No MS2	9283146.2
	No matches	No matches	No matches	No matches		No MS2	13036755
	No matches	No matches	No matches	No matches		No MS2	9040010.5
	No matches	No matches	No matches	No matches		No MS2	13018168
	No matches	No matches	Multiple ma	No matches		No MS2	10827731
	No matches	No matches	No matches	No matches		DDA for pref	9985674
	No matches	No matches	No matches	No matches		No MS2	10000661
	No matches	No matches	No matches	No matches		No MS2	12986396
	No matches	No matches	No matches	No matches		DDA for othe	9885622.9
	No matches	No matches	No matches	No matches		No MS2	12979458
	No matches	No matches	No matches	No matches		No MS2	12979413
	No matches	No matches	No matches	No matches		No MS2	1402521.2
	No matches	No matches	Multiple ma	No matches		No MS2	11372713
	No matches	No matches	No matches	No matches		No MS2	6811896
	No matches	No matches	No matches	No matches		No MS2	12965703
	No matches	No matches	No matches	No matches		No MS2	10909375
	Multiple ma	No matches	Multiple ma	No matches		No MS2	8288334.1
	No matches	No matches	No matches	No matches		DDA for pref	9853050.1
	No matches	No matches	No matches	No matches		No MS2	9538780.3
	No matches	No matches	No matches	No matches		No MS2	4745282.4
	No matches	No matches	No matches	No matches		No MS2	7059481
	No matches	No matches	No matches	No matches		No MS2	5431856.9
	No matches	No matches	No matches	No matches		No MS2	12589428
	No matches	No matches	No matches	No matches		No MS2	10730071
	No matches	No matches	No matches	No matches		No MS2	11553353
	No matches	No matches	No matches	No matches		No MS2	12775314
	No matches	No matches	No matches	No matches		No MS2	9033660.2
	No matches	No matches	No matches	No matches		No MS2	10598429
	No matches	No matches	No matches	No matches		No MS2	10546325
	No matches	No matches	No matches	No matches		No MS2	3504103.1
	No matches	No matches	No matches	No matches		No MS2	12034231
	No matches	No matches	No matches	No matches		No MS2	12753568

	No matches	No matches	No matches	No matches		No MS2	5255827.8
	No matches	No matches	No matches	No matches		No MS2	5849657.2
	No matches	No matches	No matches	No matches		No MS2	8969599.2
	No matches	No matches	No matches	No matches		No MS2	8576673.9
	Single match	No matches	No matches	No matches		No MS2	10196129
	Single match	No matches	No matches	No matches		No MS2	1607291.9
	No matches	No matches	No matches	No matches		No MS2	12715903
	No matches	No matches	No matches	No matches		No MS2	11737677
	No matches	No matches	No matches	No matches		No MS2	6206483.4
	Single match	No matches	No matches	No matches		No MS2	9582853.9
	Multiple ma	No matches	Single match	Single match		No MS2	7677882.4
	No matches	No matches	No matches	No matches		No MS2	681148.1
	Multiple ma	No matches	Single match	No matches		No MS2	841521.57
	No matches	No matches	No matches	No matches		No MS2	10957239
	No matches	No matches	No matches	No matches		No MS2	8301693.8
	No matches	No matches	Single match	No matches		No MS2	3709019.2
	No matches	No matches	No matches	No matches		No MS2	4558467.2
	No matches	No matches	No matches	No matches		No MS2	2522147.3
	No matches	No matches	No matches	No matches		No MS2	8664496
	No matches	No matches	No matches	No matches		No MS2	12557182
	No matches	No matches	No matches	No matches		No MS2	7799049.1
	No matches	No matches	No matches	No matches		No MS2	486504.9
	No matches	No matches	No matches	No matches		No MS2	8957620.6
	No matches	No matches	No matches	No matches		No MS2	5032143.4
	No matches	No matches	No matches	No matches		No MS2	12530489
	No matches	No matches	No matches	No matches		No MS2	9791271.5
	No matches	No matches	No matches	Single match		No MS2	5816448.9
	No matches	No matches	No matches	No matches		No MS2	7371157.6
	No matches	No matches	No matches	No matches		No MS2	7172521
	No matches	No matches	No matches	No matches		No MS2	12474130
	No matches	No matches	No matches	No matches		No MS2	5416310.5
	No matches	No matches	No matches	No matches		No MS2	11831025
	No matches	No matches	No matches	No matches		No MS2	8923132.9
	No matches	No matches	No matches	No matches		No MS2	12431444
	No matches	No matches	No matches	No matches		No MS2	12426464
	No matches	No matches	No matches	No matches		No MS2	12411928
	No matches	No matches	No matches	No matches		No MS2	10251348
	Single match	No matches	No matches	No matches		No MS2	9489659.3
	No matches	No matches	No matches	No matches		No MS2	1048215.2
	No matches	No matches	Single match	No matches		No MS2	9541058
	No matches	No matches	No matches	No matches		No MS2	8230867.9
	No matches	No matches	No matches	No matches		No MS2	12248181
	No matches	No matches	No matches	No matches		No MS2	12242096
	No matches	No matches	No matches	No matches		No MS2	12225787
	No matches	No matches	No matches	No matches		No MS2	8919444.2
	No matches	No matches	No matches	No matches		No MS2	4749778.8
	No matches	No matches	Multiple ma	No matches		No MS2	10825890
	No matches	Multiple ma	Single match	No matches		No MS2	11472874
	No matches	No matches	No matches	No matches		No MS2	274238.34
	No matches	No matches	No matches	No matches		No MS2	5925032.5

	Multiple ma	No matches	No matches	No matches		No MS2	6290252.4
	No matches	No matches	No matches	No matches		No MS2	12142360
	No matches	No matches	No matches	No matches		No MS2	10072396
	No matches	No matches	No matches	No matches		No MS2	9321615.4
	No matches	No matches	No matches	No matches		No MS2	12065637
	No matches	No matches	No matches	No matches		No MS2	12046544
	No matches	No matches	No matches	No matches		No MS2	2047436.7
	No matches	No matches	No matches	No matches		No MS2	4086248.3
	No matches	No matches	No matches	No matches		No MS2	8069807.2
	No matches	No matches	No matches	No matches		No MS2	8194019.6
	No matches	No matches	No matches	No matches		No MS2	10233461
	No matches	No matches	No matches	No matches		No MS2	1298139.4
	No matches	No matches	No matches	No matches		No MS2	11029972
	No matches	No matches	No matches	No matches		No MS2	10625948
	No matches	No matches	No matches	No matches		No MS2	9586367.3
	No matches	No matches	No matches	No matches		No MS2	11305830
	No matches	Single match	No matches	No matches		No MS2	11973883
	No matches	No matches	No matches	No matches		No MS2	11971458
	No matches	No matches	No matches	No matches		No MS2	8630607.3
	No matches	No matches	No matches	No matches		No MS2	1656802.8
	No matches	No matches	No matches	No matches		DDA for pref	9632817
	No matches	No matches	Single match	No matches		No MS2	9343867.2
	Single match	No matches	No matches	No matches		No MS2	11560613
	No matches	No matches	No matches	No matches		No MS2	9977464.2
	No matches	No matches	No matches	No matches		No MS2	11911299
	No matches	No matches	No matches	No matches		No MS2	10166890
	No matches	No matches	No matches	No matches		No MS2	6611901.8
	No matches	No matches	No matches	No matches		No MS2	8594894.5
	No matches	No matches	No matches	No matches		No MS2	9865703.6
	Multiple ma	Single match	Multiple ma	No matches		No MS2	8414834.2
	No matches	No matches	No matches	No matches		No MS2	7206247.8
	No matches	No matches	No matches	No matches		No MS2	6879409.9
	No matches	Single match	No matches	No matches		No MS2	9643596
	No matches	No matches	No matches	No matches		No MS2	11838091
	No matches	No matches	No matches	No matches		No MS2	4915842.5
	No matches	No matches	No matches	No matches		No MS2	2562472.3
	No matches	No matches	No matches	No matches		No MS2	7833974.4
	No matches	No matches	No matches	No matches		No MS2	11783216
	No matches	No matches	No matches	No matches		No MS2	2172880.9
	No matches	No matches	No matches	No matches		No MS2	11765210
	No matches	No matches	No matches	No matches		No MS2	11739821
	No matches	No matches	No matches	No matches		No MS2	10635826
	No matches	No matches	No matches	No matches		No MS2	11719267
	No matches	No matches	No matches	No matches		No MS2	6668942.3
	No matches	No matches	No matches	No matches		No MS2	7473696.9
	No matches	No matches	No matches	No matches		No MS2	5411003.8
	No matches	No matches	No matches	No matches		No MS2	8590611.6
	No matches	No matches	No matches	No matches		No MS2	11685776
	No matches	No matches	No matches	No matches		No MS2	3871988.1
	No matches	No matches	No matches	No matches		No MS2	319756.81

	No matches	No matches	No matches	No matches		No MS2	10312466
	No matches	No matches	No matches	No matches		No MS2	8205284.8
	No matches	No matches	No matches	No matches		No MS2	2322247.2
	No matches	No matches	No matches	No matches		No MS2	11604454
	Multiple ma	No matches	No matches	No matches		No MS2	11587230
	Multiple ma	No matches	No matches	No matches		No MS2	4971418.8
	No matches	No matches	No matches	No matches		No MS2	295006.1
	No matches	No matches	No matches	No matches		No MS2	5471950.2
	No matches	No matches	Single match	No matches		No MS2	9972074.8
	No matches	No matches	No matches	No matches		No MS2	301795.73
	No matches	No matches	No matches	No matches		No MS2	11554540
	Multiple ma	No matches	Single match	No matches		No MS2	9197054.9
	No matches	No matches	No matches	No matches		No MS2	8362166.7
	No matches	No matches	No matches	No matches		No MS2	9674960.2
	No matches	No matches	No matches	No matches		No MS2	2459955.5
	No matches	No matches	No matches	No matches		No MS2	6884421.7
	No matches	No matches	No matches	No matches		No MS2	885093.39
	No matches	No matches	No matches	No matches		No MS2	10572619
	No matches	No matches	No matches	No matches		No MS2	5105398.2
	No matches	No matches	No matches	No matches		No MS2	11467967
	Multiple ma	No matches	No matches	No matches		No MS2	8231305.6
	No matches	No matches	No matches	No matches		No MS2	6415913.3
	No matches	No matches	No matches	No matches		No MS2	7820424.3
	No matches	No matches	No matches	Single match		No MS2	11431079
	No matches	No matches	No matches	No matches		No MS2	4858638
	Single match	No matches	No matches	No matches		No MS2	5980820.3
	No matches	No matches	No matches	No matches		No MS2	1053468.8
	No matches	Single match	Multiple ma	No matches		DDA for pref	9391312
	Multiple ma	No matches	No matches	No matches		No MS2	6588135.4
	No matches	No matches	No matches	No matches		No MS2	3458796.6
	No matches	No matches	No matches	No matches		No MS2	8574525.9
	No matches	No matches	No matches	No matches		No MS2	6500531.9
	No matches	No matches	No matches	No matches		No MS2	5042265.9
	No matches	No matches	No matches	No matches		No MS2	11355708
	No matches	No matches	No matches	No matches		No MS2	7849947.6
	No matches	No matches	No matches	No matches		No MS2	11344991
	No matches	No matches	No matches	No matches		No MS2	11339715
	No matches	No matches	No matches	No matches		No MS2	11320262
	No matches	No matches	No matches	No matches		No MS2	2409817.3
	No matches	No matches	No matches	No matches		No MS2	3788515.5
	No matches	No matches	No matches	No matches		No MS2	8182903.3
	No matches	No matches	No matches	No matches		No MS2	4857157.7
	No matches	No matches	No matches	No matches		No MS2	7461859.5
	No matches	No matches	No matches	No matches		No MS2	11251366
	No matches	No matches	No matches	No matches		No MS2	11250297
	No matches	No matches	No matches	No matches		No MS2	8462275.9
	No matches	No matches	No matches	No matches		No MS2	697389.86
	No matches	No matches	No matches	No matches		No MS2	990881.83
	No matches	No matches	No matches	No matches		No MS2	7342924.6
	No matches	No matches	No matches	Multiple ma		No MS2	11200252

	No matches	No matches	Single match	No matches		No MS2	6216431.6
	No matches	No matches	No matches	No matches		No MS2	8700404.8
	No matches	No matches	No matches	No matches		No MS2	10128421
	No matches	No matches	No matches	No matches		No MS2	11169612
	No matches	No matches	No matches	No matches		No MS2	1006992
	No matches	Single match	Single match	Single match		No MS2	7802046.5
	No matches	No matches	No matches	No matches		No MS2	11157678
	No matches	No matches	Multiple ma	No matches		No MS2	11151179
	No matches	No matches	Single match	No matches		No MS2	1241747.4
	No matches	No matches	No matches	No matches		No MS2	9279652.1
	No matches	No matches	No matches	No matches		No MS2	6215699.7
	No matches	No matches	No matches	No matches		No MS2	7803695.5
	No matches	No matches	No matches	No matches		No MS2	8165424
	No matches	No matches	No matches	No matches		No MS2	748804.25
	No matches	No matches	No matches	No matches		No MS2	11072820
	No matches	No matches	No matches	No matches		DDA for pref	11064714
	No matches	No matches	No matches	No matches		No MS2	10620762
	No matches	No matches	Single match	No matches		No MS2	8551019.2
	Multiple ma	No matches	No matches	No matches		No MS2	10525255
	No matches	No matches	No matches	No matches		No MS2	11031643
	No matches	No matches	No matches	No matches		No MS2	4408002.1
	Single match	No matches	Single match	Single match		No MS2	4964438.3
	No matches	No matches	No matches	No matches		No MS2	1527593.1
	No matches	No matches	No matches	Multiple ma		No MS2	8276960
	No matches	No matches	No matches	No matches		No MS2	4874247.9
	No matches	No matches	No matches	No matches		No MS2	6607473.8
	No matches	No matches	No matches	No matches		No MS2	8332838.2
	No matches	No matches	No matches	No matches		No MS2	5692239.3
	No matches	No matches	No matches	No matches		No MS2	7209961.6
	No matches	No matches	No matches	No matches		No MS2	10941004
	No matches	No matches	No matches	No matches		No MS2	8196329.6
	No matches	Single match	No matches	No matches		No MS2	5402847.9
	No matches	No matches	No matches	No matches		No MS2	10909522
	No matches	No matches	No matches	No matches		No MS2	8305143.8
	No matches	No matches	Single match	No matches		No MS2	4607722.8
	No matches	No matches	No matches	No matches		No MS2	4711006.5
	No matches	No matches	No matches	No matches		No MS2	10885054
	No matches	Single match	Multiple ma	No matches		No MS2	10871322
	No matches	No matches	No matches	No matches		No MS2	8730527
	No matches	No matches	No matches	No matches		No MS2	8921086.2
	No matches	No matches	No matches	No matches		No MS2	5942772.9
	No matches	No matches	No matches	No matches		No MS2	2881480.1
	No matches	No matches	No matches	No matches		No MS2	4068702
	No matches	No matches	No matches	No matches		No MS2	930860.75
	No matches	No matches	No matches	No matches		No MS2	5347274.2
	Multiple ma	No matches	No matches	No matches		No MS2	4101325.9
	No matches	No matches	No matches	No matches		No MS2	7822036
	No matches	No matches	No matches	No matches		No MS2	2308242.4
	No matches	No matches	Multiple ma	No matches		No MS2	3845685.7
	No matches	No matches	No matches	No matches		No MS2	108972.26

	No matches	No matches	No matches	No matches		No MS2	6219065.4
	Multiple ma	No matches	Single match	No matches		No MS2	5878219.7
	No matches	No matches	No matches	No matches		No MS2	273704.38
	No matches	No matches	No matches	No matches		No MS2	1674398.6
	No matches	No matches	Single match	No matches		No MS2	7932762
	No matches	No matches	No matches	No matches		No MS2	6639720.9
	Multiple ma	No matches	No matches	No matches		No MS2	2911606.7
	No matches	No matches	No matches	No matches		No MS2	1412742.8
	No matches	No matches	No matches	No matches		No MS2	7843427.3
	No matches	No matches	No matches	No matches		No MS2	4709875.1
	No matches	No matches	No matches	No matches		No MS2	5573813.7
	No matches	No matches	No matches	No matches		No MS2	6282714.4
	No matches	No matches	No matches	No matches		No MS2	6303249.7
	No matches	No matches	No matches	No matches		No MS2	5164003.6
	No matches	No matches	No matches	Single match		No MS2	10626690
	No matches	No matches	No matches	No matches		DDA for pref	2786088.2
	No matches	No matches	No matches	No matches		No MS2	6567354.6
	No matches	No matches	No matches	No matches		No MS2	4326986.1
	Multiple ma	No matches	No matches	No matches		DDA for pref	8726543.9
	No matches	No matches	No matches	No matches		No MS2	10551129
	No matches	No matches	No matches	No matches		No MS2	3915518.9
	No matches	No matches	Single match	No matches		No MS2	8831670.3
	No matches	No matches	No matches	No matches		No MS2	6483065
	Multiple ma	No matches	No matches	No matches		No MS2	6638420.7
	Multiple ma	No matches	No matches	No matches		No MS2	3626279.2
	No matches	No matches	Single match	No matches		No MS2	8247941.2
	No matches	No matches	No matches	No matches		No MS2	6968595.8
	Multiple ma	No matches	No matches	No matches		No MS2	5493745
	No matches	No matches	No matches	No matches		No MS2	4255275.8
	No matches	No matches	Single match	Single match		No MS2	3776935.8
	No matches	No matches	No matches	No matches		No MS2	10467221
	No matches	No matches	No matches	No matches		No MS2	3049100.9
	No matches	No matches	No matches	No matches		DDA for othe	10463337
	No matches	No matches	No matches	No matches		No MS2	5720204.9
	No matches	No matches	No matches	No matches		No MS2	8126941.6
	No matches	No matches	No matches	No matches		No MS2	616407.99
	No matches	No matches	No matches	No matches		No MS2	1014763
	No matches	No matches	No matches	No matches		No MS2	2875378.3
	No matches	No matches	No matches	No matches		No MS2	10412750
	Multiple ma	No matches	No matches	No matches		No MS2	4359117.9
	No matches	No matches	No matches	No matches		No MS2	7318970.1
	No matches	No matches	No matches	No matches		No MS2	3748154.7
	No matches	No matches	Single match	Single match		DDA for othe	10071151
	No matches	No matches	No matches	No matches		No MS2	10378969
	No matches	No matches	No matches	No matches		No MS2	10376895
	No matches	No matches	No matches	No matches		No MS2	7080760.6
	No matches	No matches	No matches	No matches		No MS2	5400179.9
	Multiple ma	No matches	Multiple ma	No matches		No MS2	8030252.7
	No matches	No matches	No matches	No matches		No MS2	1782415.8
	No matches	No matches	No matches	No matches		No MS2	10316545

	No matches	No matches	No matches	No matches		No MS2	10304922
	No matches	No matches	Multiple ma	Multiple ma		No MS2	7525580.5
	Multiple ma	No matches	Multiple ma	No matches		No MS2	10278053
	No matches	No matches	No matches	No matches		No MS2	5405638.6
	No matches	No matches	No matches	No matches		No MS2	5464176.8
	Multiple ma	No matches	No matches	No matches		No MS2	10231146
	No matches	No matches	No matches	No matches		No MS2	4482011.6
	No matches	No matches	No matches	No matches		No MS2	2990023.7
	No matches	No matches	No matches	No matches		No MS2	6700341.3
	No matches	No matches	No matches	No matches		No MS2	6761950.1
	Multiple ma	No matches	Single match	No matches		No MS2	8450542.1
	No matches	No matches	No matches	No matches		No MS2	4060129.7
	No matches	No matches	No matches	Single match		No MS2	10132305
	No matches	No matches	No matches	No matches		No MS2	4625480.5
	Single match	No matches	No matches	No matches		No MS2	2653277.7
	No matches	No matches	No matches	No matches		No MS2	8944485.9
	No matches	No matches	No matches	No matches		No MS2	6402394.2
	No matches	No matches	No matches	No matches		No MS2	10094142
	Multiple ma	No matches	Single match	No matches		No MS2	4166892.7
	No matches	No matches	Multiple ma	No matches		No MS2	9800471.3
	No matches	No matches	No matches	No matches		No MS2	6274606.7
	No matches	No matches	No matches	No matches		No MS2	5014374.9
	No matches	No matches	No matches	No matches		No MS2	452529.71
	No matches	No matches	No matches	No matches	85.1	DDA for pref	2476995.3
	No matches	No matches	No matches	No matches		No MS2	9089153.9
	No matches	No matches	No matches	No matches		No MS2	9986984.8
	No matches	No matches	No matches	No matches		No MS2	951329.95
	Multiple ma	No matches	No matches	No matches		No MS2	1954153.1
	No matches	No matches	No matches	No matches		No MS2	4547245.6
	Multiple ma	No matches	No matches	No matches		No MS2	278342.27
	No matches	No matches	No matches	No matches		No MS2	7513127.8
	No matches	No matches	No matches	No matches		No MS2	10004409
	No matches	No matches	No matches	No matches		No MS2	2855739.8
	No matches	Single match	No matches	No matches		No MS2	7176838.2
	No matches	No matches	No matches	No matches		No MS2	6876317.6
	No matches	No matches	No matches	No matches		No MS2	7736137.9
	No matches	No matches	Single match	No matches		No MS2	7074064.5
	No matches	No matches	No matches	No matches		No MS2	3801375.3
	No matches	No matches	No matches	No matches		No MS2	9949516.7
	No matches	No matches	No matches	No matches		No MS2	9931580.3
	No matches	No matches	No matches	No matches		No MS2	7355361
	No matches	No matches	No matches	Single match		No MS2	7284526.5
	No matches	No matches	No matches	No matches		No MS2	9912832.5
	No matches	No matches	No matches	No matches		No MS2	939508.14
	No matches	No matches	No matches	No matches		No MS2	650323.77
	No matches	No matches	Multiple ma	Multiple ma		No MS2	9892811.4
	No matches	No matches	No matches	No matches		No MS2	1208527.5
	No matches	No matches	No matches	No matches		No MS2	339412.53
	No matches	No matches	No matches	No matches		No MS2	5815334.5
	No matches	No matches	No matches	No matches		No MS2	8054735.7

	No matches	No matches	No matches	No matches		No MS2	7861981.6
	No matches	No matches	No matches	No matches		No MS2	5129420.6
	No matches	No matches	No matches	No matches		No MS2	9015906.2
	No matches	No matches	No matches	No matches		No MS2	8325595.4
	No matches	No matches	No matches	No matches		No MS2	7781636.5
	No matches	No matches	No matches	No matches		No MS2	6001771.9
	No matches	No matches	No matches	No matches		No MS2	8359833.4
	No matches	No matches	No matches	No matches		No MS2	5889049.1
	No matches	No matches	No matches	No matches		No MS2	5901113
	No matches	No matches	No matches	No matches		No MS2	6949368.1
	No matches	No matches	No matches	No matches		No MS2	5557458.2
	No matches	No matches	No matches	No matches		No MS2	9808886
	No matches	No matches	No matches	No matches		No MS2	9806821.6
	No matches	No matches	Single match	No matches		No MS2	8809700.4
	No matches	No matches	No matches	No matches		No MS2	9783627.9
	No matches	No matches	No matches	No matches		No MS2	8918344.2
	No matches	No matches	No matches	No matches		No MS2	9778322.2
	No matches	No matches	No matches	No matches		No MS2	1565942
	No matches	No matches	Single match	No matches		No MS2	4228134.8
	No matches	No matches	No matches	No matches		No MS2	9759121.2
	No matches	No matches	No matches	Multiple ma		No MS2	4373525
	No matches	No matches	No matches	No matches		No MS2	7875978.4
	No matches	No matches	No matches	No matches		No MS2	180735.4
	No matches	No matches	No matches	No matches		No MS2	9697336.9
	No matches	No matches	No matches	No matches		No MS2	8420476.4
	No matches	No matches	No matches	No matches		DDA for pref	8198227.7
	No matches	No matches	No matches	No matches		No MS2	6056062.3
	No matches	No matches	No matches	No matches		No MS2	9693644.4
	No matches	No matches	No matches	No matches		No MS2	8376069.9
	No matches	No matches	No matches	No matches		No MS2	4657832.7
	No matches	No matches	No matches	No matches		No MS2	6530035.7
	No matches	No matches	No matches	No matches		No MS2	9674626.5
	Multiple ma	No matches	No matches	No matches		No MS2	8056920.6
	Multiple ma	No matches	No matches	No matches		No MS2	3181017.2
	Multiple ma	No matches	Single match	No matches		No MS2	6391792.8
	No matches	No matches	No matches	No matches		No MS2	524243.26
	No matches	No matches	No matches	No matches		No MS2	7135701.6
	No matches	No matches	No matches	No matches		No MS2	9638354.9
	No matches	No matches	No matches	No matches		No MS2	8572254.5
	Single match	No matches	No matches	No matches		No MS2	7475690.1
	No matches	No matches	No matches	No matches		No MS2	9618403
	No matches	No matches	No matches	No matches		No MS2	3955478.1
	No matches	Multiple ma	No matches	Single match		No MS2	6193103.9
	No matches	No matches	Single match	No matches		No MS2	5480704.5
	No matches	No matches	No matches	No matches		No MS2	3276034.8
	No matches	No matches	No matches	No matches		No MS2	9570861.3
	No matches	No matches	No matches	No matches		No MS2	7523877
	No matches	No matches	No matches	No matches		No MS2	5512130.7
	No matches	No matches	No matches	No matches		No MS2	6155824
	No matches	Single match	No matches	No matches		No MS2	869325.45

	No matches	No matches	No matches	No matches		No MS2	5313278.5
	No matches	No matches	No matches	No matches		No MS2	6476336.3
	No matches	Single match	No matches	Single match		No MS2	1292800.2
	No matches	No matches	No matches	No matches		No MS2	3643839
	No matches	No matches	No matches	No matches		No MS2	5302227.4
	No matches	No matches	No matches	No matches		No MS2	5566716.2
	No matches	No matches	No matches	No matches		No MS2	9237990.6
	No matches	No matches	No matches	No matches		No MS2	3793393.5
	No matches	No matches	No matches	No matches		No MS2	3511983.1
	No matches	No matches	No matches	No matches		No MS2	9489476.5
	No matches	No matches	No matches	No matches		No MS2	9488037
	No matches	No matches	No matches	No matches		No MS2	837241.99
	No matches	No matches	No matches	No matches		No MS2	4001084.9
	No matches	No matches	No matches	No matches		No MS2	2171376.3
	No matches	No matches	No matches	No matches		No MS2	6416181.7
	No matches	No matches	No matches	No matches		No MS2	9109619.7
	No matches	No matches	No matches	No matches		No MS2	5539370.8
	No matches	No matches	No matches	No matches		No MS2	4387895.8
	No matches	No matches	No matches	No matches		No MS2	7708281.8
	No matches	Single match	No matches	No matches		No MS2	8433367.7
	Multiple ma	No matches	No matches	No matches		DDA for pref	8124466.8
	No matches	No matches	No matches	No matches		No MS2	7372286.1
	No matches	No matches	No matches	No matches		No MS2	256175.06
	No matches	No matches	No matches	No matches		No MS2	956789.94
	No matches	No matches	No matches	No matches		No MS2	7730497.2
	No matches	No matches	No matches	No matches		No MS2	6347420.5
	No matches	No matches	No matches	No matches		No MS2	6589167.2
	No matches	No matches	No matches	No matches		No MS2	7738864.1
	No matches	No matches	No matches	No matches		No MS2	9419927.1
	No matches	No matches	No matches	No matches		No MS2	7344016.3
	No matches	No matches	No matches	No matches		No MS2	6103131.5
	No matches	No matches	No matches	No matches		No MS2	9003932.5
	No matches	No matches	No matches	No matches		No MS2	4067767.7
	No matches	No matches	No matches	No matches		No MS2	6648103.4
	No matches	No matches	No matches	No matches		No MS2	6623264.5
	No matches	No matches	No matches	No matches		No MS2	9349809
	No matches	No matches	No matches	No matches		No MS2	9141472.5
	No matches	No matches	Single match	No matches		No MS2	6328900.2
	No matches	No matches	No matches	No matches		No MS2	7456992
	No matches	No matches	No matches	No matches		No MS2	5948135.6
	No matches	No matches	No matches	No matches		No MS2	5863969.8
	No matches	No matches	No matches	No matches		No MS2	7040851.3
	No matches	No matches	No matches	No matches		No MS2	7155582.7
	No matches	No matches	No matches	No matches		No MS2	6563812.9
	No matches	No matches	No matches	No matches		No MS2	5155981.7
	No matches	No matches	No matches	No matches		No MS2	8630855.1
	No matches	No matches	No matches	No matches		No MS2	314364.49
	No matches	No matches	No matches	No matches		No MS2	7513465.2
	No matches	No matches	No matches	No matches		No MS2	1280672
	No matches	No matches	Single match	No matches		No MS2	5501974.6

	No matches	No matches	No matches	Multiple ma		No MS2	7071577.5
	No matches	No matches	No matches	No matches		No MS2	1248444.6
	No matches	No matches	Single match	No matches		No MS2	2546925
	No matches	No matches	No matches	No matches		DDA for pref	4153930.1
	No matches	No matches	No matches	No matches		No MS2	6429229.2
	No matches	No matches	Single match	No matches		No MS2	8313353.9
	No matches	No matches	No matches	No matches		No MS2	2232404.1
	Multiple ma	No matches	Single match	No matches		No MS2	9146293.6
	No matches	No matches	No matches	No matches		No MS2	9142015.8
	No matches	No matches	No matches	No matches		No MS2	631658.48
	No matches	Single match	No matches	No matches		No MS2	9128131.1
	No matches	No matches	No matches	No matches		No MS2	8454209.2
	No matches	No matches	No matches	No matches		No MS2	6334922
	No matches	No matches	No matches	No matches		No MS2	9097615.1
	No matches	No matches	No matches	No matches		No MS2	6773263.4
	No matches	No matches	No matches	No matches		No MS2	3486327
	No matches	No matches	No matches	No matches		No MS2	290398.32
	No matches	No matches	Multiple ma	Multiple ma		No MS2	6609128
	No matches	No matches	No matches	No matches		No MS2	6604135
	Multiple ma	No matches	No matches	No matches		No MS2	6592752.1
	No matches	No matches	No matches	No matches		No MS2	3224320.4
	No matches	No matches	No matches	No matches		No MS2	7266306.2
	No matches	Single match	No matches	No matches		No MS2	5253128
	No matches	No matches	No matches	No matches		No MS2	3901051.3
	No matches	No matches	No matches	No matches		No MS2	7095213.9
	No matches	No matches	No matches	No matches		No MS2	8979560.8
	No matches	No matches	No matches	No matches		No MS2	8012023.3
	No matches	No matches	Single match	No matches		No MS2	594482.76
	No matches	No matches	No matches	No matches		No MS2	7533456.2
	No matches	No matches	No matches	No matches		No MS2	3979316.1
	No matches	No matches	Multiple ma	No matches		No MS2	4729650.9
	No matches	No matches	No matches	No matches		No MS2	4234892
	No matches	No matches	No matches	No matches		No MS2	8911422.8
	No matches	No matches	No matches	Multiple ma		No MS2	8911055
	No matches	No matches	No matches	No matches		No MS2	631904.38
	No matches	No matches	No matches	No matches		No MS2	6637727.4
	No matches	No matches	No matches	No matches		No MS2	317266.56
	No matches	No matches	No matches	No matches		No MS2	5875062.1
	No matches	No matches	No matches	No matches		No MS2	5802299
	No matches	No matches	Single match	No matches		No MS2	8371630.7
	No matches	Single match	No matches	No matches		No MS2	7466979.3
	No matches	No matches	No matches	No matches		No MS2	7555446.1
	No matches	No matches	No matches	No matches		No MS2	8795664.9
	No matches	No matches	No matches	No matches		No MS2	5016711.7
	No matches	No matches	No matches	Multiple ma		No MS2	6797801.7
	No matches	No matches	No matches	No matches		No MS2	6345730.3
	No matches	No matches	No matches	No matches		DDA for othe	3310606.7
	No matches	No matches	No matches	No matches		No MS2	8763883.3
	No matches	No matches	No matches	No matches		No MS2	8762741.2
	Multiple ma	No matches	No matches	No matches		No MS2	1389118

	Multiple ma	No matches	No matches	No matches		No MS2	7643202.5
	Multiple ma	No matches	No matches	No matches		No MS2	6095394.1
	No matches	No matches	No matches	No matches		No MS2	6492851.3
	No matches	Single match	No matches	No matches		No MS2	8495994.7
	No matches	No matches	No matches	No matches		No MS2	329607.31
	No matches	No matches	No matches	No matches		No MS2	6141054.8
	No matches	No matches	No matches	No matches		No MS2	5856637.1
	Multiple ma	No matches	Single match	No matches		No MS2	1966930.8
	No matches	No matches	No matches	No matches		No MS2	6631281.8
	Multiple ma	No matches	No matches	No matches		No MS2	7203683.1
	No matches	No matches	No matches	No matches		DDA for pref	7833444.4
	Multiple ma	No matches	No matches	No matches		No MS2	8682616.5
	No matches	No matches	No matches	No matches		No MS2	7484105.2
	No matches	No matches	No matches	No matches		No MS2	1368002.8
	No matches	No matches	No matches	No matches		No MS2	8665111.9
	No matches	No matches	No matches	No matches		No MS2	1829823.7
	No matches	Multiple ma	No matches	No matches		No MS2	4783510.1
	No matches	No matches	No matches	No matches		No MS2	975084.96
	No matches	No matches	No matches	No matches		No MS2	6878861.3
	No matches	No matches	No matches	Single match		No MS2	8639711.4
	No matches	No matches	No matches	No matches		No MS2	4388538.9
	No matches	No matches	No matches	No matches		No MS2	8629803.4
	No matches	No matches	Multiple ma	No matches		No MS2	6681432.9
	No matches	No matches	No matches	No matches		No MS2	8623617.5
	No matches	No matches	No matches	No matches		No MS2	2322811.9
	No matches	No matches	No matches	No matches		No MS2	387555.98
	No matches	No matches	No matches	No matches		No MS2	6320954.6
	No matches	No matches	No matches	No matches		No MS2	1343683
	No matches	No matches	No matches	No matches		No MS2	8605568.9
	No matches	No matches	No matches	No matches		No MS2	1393618
	No matches	No matches	No matches	No matches		No MS2	7961058.1
	No matches	No matches	No matches	No matches		No MS2	7111366.2
	Multiple ma	No matches	No matches	No matches		No MS2	3411341.1
	No matches	No matches	No matches	No matches		No MS2	8562129.1
	No matches	No matches	No matches	Single match		No MS2	6705354.2
	No matches	No matches	No matches	No matches		No MS2	444076.24
	No matches	No matches	No matches	No matches		No MS2	1851011.7
	No matches	No matches	No matches	No matches		No MS2	672541.87
	No matches	No matches	No matches	No matches		No MS2	6098421.8
	No matches	No matches	No matches	No matches		No MS2	4329880.5
	No matches	No matches	No matches	No matches		No MS2	5662906.1
	No matches	No matches	No matches	No matches		No MS2	6383208.1
	No matches	No matches	No matches	No matches		No MS2	4155793.4
	No matches	No matches	No matches	Single match		No MS2	7565537.9
	No matches	No matches	No matches	No matches		No MS2	207923.48
	No matches	No matches	No matches	No matches		No MS2	4765588.4
	No matches	No matches	No matches	No matches		No MS2	6644947.8
	No matches	No matches	No matches	No matches		No MS2	4001744.6
	Multiple ma	No matches	No matches	No matches		No MS2	7351348.5
	No matches	No matches	No matches	No matches		No MS2	8472308.5

	No matches	No matches	No matches	No matches		No MS2	3105864.3
	Multiple ma	No matches	No matches	No matches		No MS2	6815080.5
	No matches	No matches	Single match	No matches		No MS2	6782987
	No matches	No matches	No matches	No matches		No MS2	5213660.7
	No matches	No matches	No matches	No matches		No MS2	6423808.2
	No matches	No matches	No matches	No matches		No MS2	6353327.3
	No matches	No matches	No matches	No matches		No MS2	7114616.5
	No matches	No matches	No matches	No matches		No MS2	8422160
	No matches	No matches	No matches	No matches		No MS2	6752177.8
	No matches	No matches	No matches	No matches		No MS2	4384995.3
	No matches	Single match	No matches	No matches		No MS2	8398743.4
	Multiple ma	No matches	No matches	No matches		No MS2	3484191
	No matches	No matches	No matches	No matches		No MS2	6053417.5
	No matches	No matches	No matches	No matches		No MS2	8375151.6
	No matches	No matches	No matches	No matches		No MS2	6093640.7
	Multiple ma	No matches	No matches	No matches		No MS2	8359010.6
	No matches	No matches	No matches	No matches		No MS2	6237760.9
	No matches	No matches	No matches	No matches		No MS2	5693840.1
	No matches	No matches	No matches	No matches		No MS2	4799951.3
	No matches	No matches	No matches	No matches		No MS2	785172.16
	No matches	No matches	No matches	No matches		No MS2	479560.43
	No matches	No matches	No matches	No matches		No MS2	5295063.8
	No matches	No matches	No matches	No matches		No MS2	5605087.9
	No matches	No matches	No matches	No matches		DDA for pref	840529.52
	No matches	No matches	No matches	No matches		No MS2	6501981.5
	No matches	No matches	No matches	No matches		No MS2	7262209.4
	No matches	No matches	No matches	No matches		No MS2	4518811.1
	No matches	No matches	No matches	No matches		No MS2	8252749.5
	No matches	No matches	No matches	No matches		No MS2	6497206.1
	No matches	No matches	No matches	No matches		No MS2	8244738.7
	No matches	No matches	No matches	No matches		DDA for pref	5027849
	Multiple ma	No matches	Multiple ma	No matches		No MS2	8233954.8
	No matches	No matches	No matches	No matches		No MS2	1865844.6
	No matches	No matches	No matches	No matches		No MS2	8204304.8
	No matches	No matches	No matches	No matches		No MS2	6780535.3
	No matches	No matches	No matches	No matches		No MS2	3843711.8
	No matches	No matches	No matches	No matches		No MS2	8185550.3
	No matches	No matches	No matches	No matches		No MS2	4845296.8
	No matches	No matches	No matches	No matches		No MS2	7158171.2
	Multiple ma	No matches	Single match	Single match		No MS2	461119.34
	No matches	No matches	No matches	No matches		No MS2	3960663.7
	No matches	No matches	No matches	No matches		No MS2	1345250.6
	No matches	No matches	No matches	No matches		No MS2	4845859.1
	No matches	No matches	No matches	No matches		No MS2	8123274
	No matches	No matches	No matches	No matches		No MS2	3327652.8
	No matches	No matches	No matches	No matches		No MS2	6385907.3
	No matches	No matches	No matches	No matches		No MS2	6167962.7
	No matches	No matches	No matches	No matches		No MS2	1707050.5
	No matches	No matches	No matches	No matches		No MS2	6717984.3
	No matches	No matches	No matches	No matches		No MS2	303526.45

	No matches	No matches	No matches	No matches		No MS2	6092841.8
	No matches	No matches	No matches	No matches		No MS2	8091335.1
	No matches	Single match	No matches	No matches		No MS2	4049512.1
	No matches	Single match	Single match	No matches		No MS2	7952694.2
	No matches	No matches	No matches	No matches		No MS2	2557970.4
	No matches	No matches	No matches	No matches		No MS2	3341476.9
	No matches	No matches	No matches	No matches		No MS2	392645.39
	No matches	No matches	No matches	Single match		No MS2	6537740.2
	No matches	No matches	No matches	No matches		No MS2	1604344.2
	No matches	No matches	No matches	No matches		No MS2	6221497.1
	No matches	No matches	No matches	No matches		No MS2	4426511.3
	No matches	No matches	No matches	No matches		No MS2	3410822.8
	No matches	No matches	No matches	No matches		No MS2	6503769.1
	No matches	No matches	No matches	No matches		No MS2	4503453.1
	No matches	No matches	No matches	No matches		No MS2	6047509.1
	No matches	No matches	No matches	No matches		No MS2	6044731.3
	No matches	No matches	No matches	No matches		No MS2	409395.94
	No matches	No matches	No matches	No matches		No MS2	4758145.9
	No matches	No matches	No matches	No matches		No MS2	7902224.5
	No matches	No matches	No matches	No matches		No MS2	1417901.1
	No matches	No matches	No matches	No matches		No MS2	4450356.3
	No matches	No matches	No matches	No matches		No MS2	3095375.1
	No matches	No matches	No matches	No matches		No MS2	7880129.9
	No matches	No matches	No matches	No matches		No MS2	690586
	No matches	No matches	No matches	No matches		No MS2	6545915.3
	Multiple ma	No matches	No matches	No matches		No MS2	7031189.1
	No matches	No matches	No matches	No matches		No MS2	4923690.3
	No matches	No matches	No matches	No matches		No MS2	952308.83
	No matches	No matches	No matches	No matches		No MS2	4114611.4
	No matches	No matches	No matches	No matches		No MS2	7835616.9
	No matches	No matches	No matches	No matches		No MS2	4752463.2
	No matches	No matches	No matches	No matches		No MS2	3504301.4
	No matches	No matches	No matches	No matches		No MS2	7145640.9
	No matches	No matches	No matches	No matches		No MS2	2804731.7
	No matches	No matches	No matches	No matches		No MS2	6201949.6
	No matches	No matches	No matches	No matches		No MS2	5569447.9
	No matches	No matches	Multiple ma	No matches		No MS2	7577413.7
	No matches	No matches	Single match	Single match		No MS2	4023018
	No matches	No matches	No matches	No matches		No MS2	1878430.3
	No matches	No matches	No matches	No matches		No MS2	6030914.8
	No matches	No matches	No matches	No matches		No MS2	5740877.9
	No matches	No matches	No matches	No matches		No MS2	5856254.8
	No matches	No matches	No matches	Single match		No MS2	3883138.9
	No matches	No matches	No matches	No matches		No MS2	7707135.4
	No matches	No matches	No matches	No matches		No MS2	4396423.4
	No matches	No matches	No matches	No matches		No MS2	1560518
	No matches	No matches	No matches	Single match		No MS2	5764504.6
	No matches	Single match	Single match	No matches		No MS2	4491915.7
	No matches	No matches	No matches	No matches		No MS2	2907420.4
	No matches	No matches	No matches	No matches		No MS2	7625064.2

	No matches	No matches	No matches	No matches		No MS2	408332.97
	No matches	No matches	No matches	No matches		No MS2	6279291.5
	No matches	No matches	No matches	No matches		No MS2	6897667.8
	No matches	No matches	No matches	No matches		No MS2	6476688.5
	No matches	No matches	No matches	No matches		No MS2	4829437
	No matches	No matches	No matches	No matches		No MS2	4432237.7
	No matches	No matches	No matches	No matches		No MS2	7029481.2
	No matches	No matches	No matches	No matches		No MS2	5767344.5
	No matches	No matches	No matches	No matches		No MS2	1728655.9
	No matches	No matches	No matches	No matches		No MS2	7562254.3
	No matches	No matches	No matches	No matches		No MS2	7026724.6
	No matches	No matches	No matches	No matches		No MS2	5366919.2
	No matches	No matches	No matches	No matches		No MS2	5022898.5
	No matches	No matches	No matches	No matches		No MS2	5796288.1
	No matches	No matches	No matches	No matches		No MS2	5671551.3
	No matches	No matches	No matches	No matches		No MS2	7499074.5
	No matches	No matches	No matches	No matches		No MS2	5014877.6
	No matches	No matches	No matches	No matches		No MS2	3480218
	No matches	No matches	No matches	No matches		No MS2	7488056.8
	No matches	No matches	No matches	No matches		No MS2	5375112.5
	No matches	No matches	No matches	No matches		No MS2	7471659.6
	No matches	No matches	No matches	No matches		No MS2	5189744.8
	No matches	No matches	No matches	No matches		No MS2	759168.24
	No matches	No matches	No matches	No matches		No MS2	5548392.1
	Multiple ma	No matches	Multiple ma	No matches		No MS2	5912670.9
	No matches	No matches	No matches	No matches		No MS2	6069291
	No matches	No matches	No matches	No matches		No MS2	988964.91
	No matches	No matches	No matches	No matches		No MS2	363879.92
	No matches	No matches	No matches	No matches		No MS2	6248045.4
	No matches	No matches	No matches	No matches		No MS2	6482295.9
	No matches	No matches	No matches	No matches		No MS2	4936390.2
	No matches	No matches	No matches	No matches		No MS2	4468535
	No matches	No matches	No matches	No matches		No MS2	5050221.8
	No matches	No matches	No matches	No matches		No MS2	3208532.9
	No matches	No matches	No matches	No matches		No MS2	2200520.5
	No matches	No matches	No matches	No matches		No MS2	4172839.1
	No matches	No matches	No matches	No matches		No MS2	2140169.9
	No matches	No matches	No matches	No matches		No MS2	7324019.7
	No matches	Single match	No matches	No matches		No MS2	7316541.3
	No matches	No matches	No matches	No matches		No MS2	4476031.2
	No matches	No matches	Multiple ma	No matches		No MS2	2444620.3
	No matches	No matches	No matches	No matches		No MS2	3861149.6
	No matches	No matches	No matches	No matches		No MS2	7297965
	No matches	No matches	Multiple ma	No matches		DDA for othe	4491448.5
	No matches	No matches	No matches	No matches		No MS2	5100365.3
	No matches	No matches	No matches	No matches		No MS2	7283669
	No matches	No matches	No matches	No matches		No MS2	7277096
	No matches	No matches	Multiple ma	No matches		No MS2	7256580.4
	No matches	No matches	Single match	No matches		No MS2	4138541.6
	No matches	No matches	No matches	No matches		No MS2	4368058.2

	No matches	No matches	No matches	No matches		No MS2	6608721
	No matches	No matches	No matches	No matches		No MS2	1705387.9
	Multiple ma	No matches	Single match	Single match		No MS2	7225894.9
	No matches	No matches	Multiple ma	No matches		No MS2	1230436.5
	No matches	No matches	No matches	No matches		No MS2	4013032.4
	No matches	No matches	No matches	No matches		No MS2	3241612.5
	No matches	No matches	No matches	No matches		No MS2	4277151.9
	No matches	No matches	No matches	No matches		No MS2	474918.73
	No matches	No matches	No matches	No matches		No MS2	7192818.8
	No matches	No matches	Multiple ma	No matches		No MS2	1165270.3
	No matches	No matches	No matches	No matches		No MS2	864997.99
	No matches	No matches	No matches	No matches		No MS2	7192307.3
	No matches	No matches	No matches	No matches		No MS2	3160379.4
	No matches	No matches	No matches	No matches		No MS2	3889954.7
	No matches	No matches	No matches	No matches		No MS2	3786092.3
	No matches	No matches	No matches	No matches		No MS2	6444221.8
	No matches	No matches	No matches	No matches		No MS2	2922035.1
	No matches	No matches	No matches	No matches		No MS2	5535076
	No matches	No matches	No matches	No matches		No MS2	1814205.6
	No matches	No matches	No matches	No matches		No MS2	7144190.1
	No matches	No matches	No matches	No matches		No MS2	3683872.8
	No matches	No matches	Single match	No matches		No MS2	7142018.6
	Multiple ma	No matches	No matches	No matches		No MS2	7140116.7
	No matches	No matches	No matches	No matches		No MS2	883713.29
	No matches	No matches	No matches	No matches		No MS2	4417145.2
	Multiple ma	No matches	No matches	No matches		No MS2	6574756.2
	No matches	No matches	No matches	No matches		No MS2	7128273.2
	No matches	No matches	No matches	No matches		No MS2	4407422.6
	No matches	No matches	No matches	No matches		No MS2	7088416.2
	No matches	No matches	No matches	No matches		No MS2	3814119.3
	No matches	No matches	No matches	No matches		No MS2	294281.72
	No matches	No matches	No matches	No matches		No MS2	3717547.7
	No matches	No matches	No matches	No matches		No MS2	227465.82
	Multiple ma	Multiple ma	No matches	No matches		No MS2	6769648.9
	No matches	No matches	No matches	No matches		No MS2	4547235.4
	No matches	No matches	No matches	No matches		No MS2	5944439.9
	No matches	No matches	No matches	No matches		No MS2	545240.71
	Multiple ma	No matches	No matches	No matches		No MS2	3622247.5
	No matches	No matches	No matches	No matches		No MS2	5097216.7
	No matches	No matches	No matches	No matches		No MS2	7028202.1
	No matches	No matches	No matches	No matches		No MS2	7011341.7
	No matches	No matches	No matches	No matches		DDA for pref	243446.68
	No matches	No matches	No matches	No matches		No MS2	2907263.2
	No matches	No matches	No matches	No matches		No MS2	4070704.6
	No matches	Single match	Single match	No matches		No MS2	6995420
	No matches	No matches	No matches	No matches		No MS2	5959250.7
	No matches	No matches	No matches	No matches		No MS2	4889884.9
	No matches	No matches	No matches	No matches		No MS2	6978841.9
	No matches	No matches	No matches	No matches		No MS2	6974692
	No matches	No matches	No matches	No matches		No MS2	5754262.6

	No matches	No matches	No matches	No matches		No MS2	6962532.5
	No matches	No matches	No matches	No matches		No MS2	1159927.8
	No matches	No matches	No matches	No matches		No MS2	3680810.9
	No matches	No matches	No matches	No matches		No MS2	5819136.3
	No matches	No matches	Multiple ma	No matches		No MS2	6119860.1
	No matches	No matches	No matches	No matches		No MS2	6928568.1
	No matches	No matches	No matches	Single match		No MS2	5045222
	No matches	No matches	No matches	No matches		No MS2	6924979.6
	Multiple ma	No matches	No matches	No matches		No MS2	6224964.1
	No matches	No matches	No matches	No matches		No MS2	4168330.8
	No matches	No matches	No matches	No matches		No MS2	5442373.2
	No matches	No matches	No matches	No matches		No MS2	4783199.6
	No matches	No matches	No matches	No matches		No MS2	6899527.2
	No matches	No matches	Single match	No matches		No MS2	1873591.8
	No matches	No matches	No matches	No matches		No MS2	6884674.3
	No matches	No matches	No matches	No matches		No MS2	432941.71
	No matches	No matches	No matches	No matches		No MS2	2208535.4
	No matches	No matches	No matches	No matches		No MS2	138442.84
	Single match	No matches	No matches	No matches		No MS2	1084636
	No matches	No matches	No matches	No matches		No MS2	6851557.3
	No matches	No matches	No matches	No matches		No MS2	5887848.2
	No matches	No matches	No matches	No matches		No MS2	1204556.2
	No matches	No matches	No matches	No matches		No MS2	6831435.7
	No matches	No matches	No matches	No matches		No MS2	1178971.8
	No matches	No matches	No matches	No matches		No MS2	965625.58
	No matches	No matches	No matches	No matches		No MS2	4205721.2
	No matches	No matches	No matches	No matches		No MS2	5022643.1
	No matches	No matches	No matches	No matches		No MS2	6772995.9
	No matches	No matches	No matches	No matches		No MS2	6456302.9
	No matches	No matches	No matches	No matches		No MS2	3729827.1
	No matches	No matches	No matches	No matches		No MS2	4452888.8
	No matches	No matches	No matches	No matches		No MS2	613568.91
	No matches	No matches	No matches	No matches		No MS2	6558781.7
	No matches	No matches	No matches	No matches		No MS2	6447689
	No matches	No matches	No matches	Single match		No MS2	6219014
	No matches	No matches	No matches	No matches		No MS2	2386312.8
	Multiple ma	No matches	No matches	No matches		No MS2	3931108.9
	No matches	No matches	Multiple ma	No matches		No MS2	5448713.2
	No matches	No matches	No matches	No matches		No MS2	2714362.1
	No matches	No matches	No matches	No matches		No MS2	2622652.7
	No matches	No matches	No matches	No matches		No MS2	4009054.1
	No matches	No matches	Single match	No matches		No MS2	6658129.2
	No matches	No matches	No matches	No matches		No MS2	4388275.5
	No matches	No matches	No matches	No matches		No MS2	5010506.6
	Multiple ma	Single match	Multiple ma	No matches		No MS2	5579891.1
	No matches	No matches	No matches	No matches		No MS2	4300307.1
	No matches	No matches	No matches	No matches		No MS2	4669750.3
	No matches	No matches	No matches	No matches		No MS2	4539813.1
	No matches	No matches	No matches	No matches		No MS2	4635434.8
	No matches	No matches	No matches	No matches		No MS2	3131879

	No matches	No matches	No matches	No matches		No MS2	5182920.9
	No matches	No matches	No matches	No matches		No MS2	6561328.1
	No matches	No matches	No matches	No matches		No MS2	6560322.4
	Multiple ma	No matches	Multiple ma	Single match		No MS2	5572546.5
	No matches	No matches	No matches	No matches		No MS2	4975222.1
	No matches	No matches	No matches	No matches		No MS2	2381783.5
	No matches	No matches	No matches	No matches		No MS2	732565.27
	No matches	No matches	No matches	No matches		No MS2	632339.73
	No matches	No matches	No matches	No matches		No MS2	6528511.8
	No matches	No matches	Multiple ma	No matches		No MS2	6520628.6
	No matches	No matches	No matches	No matches		No MS2	892547.75
	No matches	No matches	No matches	No matches		No MS2	3316862.3
	No matches	No matches	No matches	No matches		No MS2	3626080
	No matches	No matches	No matches	No matches		No MS2	938547.35
	No matches	No matches	No matches	No matches		No MS2	2181504.5
	No matches	No matches	No matches	No matches		No MS2	407398.86
	No matches	No matches	No matches	No matches		No MS2	1511014.7
	No matches	No matches	No matches	No matches		No MS2	2338444.3
	No matches	No matches	No matches	No matches		No MS2	3777459
	No matches	No matches	No matches	No matches		No MS2	738756.87
	No matches	No matches	No matches	No matches		No MS2	6437551.6
	No matches	No matches	No matches	No matches		No MS2	6428636.3
	No matches	No matches	No matches	No matches		DDA for pref	1984645.7
	No matches	No matches	Multiple ma	No matches		No MS2	6399194.9
	No matches	No matches	No matches	No matches		No MS2	6395915.4
	No matches	No matches	No matches	No matches		No MS2	4055025.8
	No matches	No matches	No matches	No matches		No MS2	4221503.9
	No matches	No matches	No matches	Single match		No MS2	2588950.6
	No matches	No matches	No matches	No matches		No MS2	1915746.2
	No matches	No matches	No matches	No matches		No MS2	272216.17
	No matches	No matches	No matches	No matches		No MS2	2128193.4
	No matches	No matches	No matches	No matches		No MS2	4155704.6
	No matches	No matches	No matches	No matches		No MS2	6362375.9
	No matches	No matches	No matches	No matches		No MS2	6362284.8
	No matches	No matches	No matches	No matches		No MS2	4058757.6
	No matches	No matches	No matches	No matches		DDA for othe	3236118.8
	No matches	No matches	No matches	No matches		No MS2	4958365.5
	No matches	No matches	No matches	No matches		No MS2	3097685.8
	No matches	No matches	No matches	No matches		No MS2	6291880.1
	Single match	No matches	No matches	No matches		No MS2	4416644.5
	No matches	No matches	No matches	No matches		No MS2	6262126.7
	No matches	No matches	No matches	No matches		No MS2	5299051.9
	No matches	No matches	No matches	No matches		No MS2	2150097.9
	No matches	No matches	No matches	No matches		No MS2	4786095.2
	No matches	No matches	No matches	No matches		No MS2	1560693.5
	No matches	No matches	No matches	No matches		No MS2	1861713.9
	No matches	No matches	No matches	No matches		No MS2	5918298.1
	No matches	No matches	No matches	No matches		No MS2	4367510.3
	No matches	No matches	No matches	No matches		No MS2	227518.56
	Multiple ma	No matches	No matches	No matches		No MS2	3325056.8

	No matches	No matches	No matches	No matches		No MS2	4810422.6
	Multiple ma	No matches	No matches	No matches		No MS2	4215597.1
	No matches	No matches	No matches	No matches		No MS2	4873878.1
	No matches	No matches	No matches	No matches		No MS2	1189132.9
	No matches	No matches	No matches	No matches		No MS2	4669791.3
	No matches	No matches	No matches	No matches		No MS2	3502308.9
	No matches	No matches	No matches	No matches		No MS2	608578.71
	No matches	No matches	No matches	No matches		No MS2	6175396.9
	Single match	No matches	No matches	No matches		No MS2	3593184.3
	No matches	No matches	No matches	No matches		No MS2	1439748.9
	No matches	No matches	No matches	No matches		No MS2	6156076.2
	No matches	No matches	Single match	Single match		No MS2	3307174.3
	No matches	Single match	No matches	No matches		No MS2	2332078.5
	No matches	No matches	No matches	No matches		No MS2	6138255.8
	No matches	No matches	No matches	No matches		No MS2	3317112.6
	No matches	No matches	No matches	No matches		No MS2	6136516.1
	No matches	No matches	No matches	No matches		No MS2	5792439.2
	No matches	No matches	No matches	No matches		DDA for pref	627446.89
	No matches	No matches	Multiple ma	No matches		No MS2	5927994.1
	No matches	No matches	No matches	No matches		No MS2	2341973.7
	No matches	No matches	No matches	No matches		No MS2	6055893.2
	No matches	No matches	Multiple ma	No matches		No MS2	5809389.3
	No matches	No matches	No matches	No matches		No MS2	3372565.3
	Multiple ma	No matches	No matches	No matches		DDA for pref	1148447.2
	No matches	No matches	No matches	No matches		No MS2	2528371.4
	Multiple ma	No matches	No matches	No matches		No MS2	1628363.7
	No matches	No matches	No matches	No matches		No MS2	3498607.4
	No matches	No matches	Multiple ma	No matches		No MS2	2305285.7
	No matches	No matches	No matches	No matches		No MS2	4852842.8
	No matches	No matches	No matches	No matches		No MS2	5950374.7
	No matches	No matches	No matches	No matches		No MS2	5934470.7
	No matches	No matches	No matches	No matches		No MS2	4504989.1
	No matches	No matches	No matches	No matches		No MS2	4040617.9
	No matches	No matches	No matches	No matches		No MS2	4397420.8
	No matches	No matches	No matches	No matches		No MS2	2292596.6
	Multiple ma	No matches	No matches	No matches		No MS2	2165508.9
	No matches	No matches	No matches	No matches		No MS2	5844493.3
	No matches	No matches	No matches	No matches		No MS2	243248.39
	Multiple ma	No matches	No matches	No matches		No MS2	2671254.1
	No matches	No matches	No matches	No matches		No MS2	2587687.6
	No matches	No matches	No matches	No matches		No MS2	4708704.3
	No matches	No matches	No matches	No matches		No MS2	3215898.8
	No matches	No matches	No matches	No matches		No MS2	4000540.1
	No matches	No matches	No matches	No matches		DDA for othe	5782135.9
	No matches	No matches	No matches	No matches		No MS2	3480218
	No matches	No matches	No matches	No matches		No MS2	4545691.9
	No matches	No matches	No matches	No matches		No MS2	281009.64
	No matches	No matches	No matches	No matches		No MS2	454782.81
	No matches	No matches	Single match	No matches		No MS2	4106573
	Multiple ma	No matches	Multiple ma	No matches		No MS2	3637139.6

	No matches	No matches	No matches	No matches		No MS2	344380.6
	No matches	No matches	No matches	No matches		No MS2	5699800.8
	No matches	No matches	No matches	No matches		No MS2	3717470.3
	No matches	No matches	Multiple ma	No matches		No MS2	3271613
	No matches	No matches	No matches	No matches		No MS2	4034645.1
	No matches	No matches	No matches	No matches		No MS2	4979021.4
	No matches	No matches	No matches	No matches		No MS2	948525.05
	No matches	No matches	No matches	No matches		No MS2	5379579.8
	No matches	No matches	Multiple ma	No matches		No MS2	2594994.7
	No matches	No matches	No matches	No matches		DDA for othe	2196114.7
	No matches	No matches	No matches	No matches		No MS2	3714016.4
	No matches	No matches	No matches	No matches		No MS2	792286.69
	No matches	No matches	No matches	No matches		No MS2	3116685.9
	No matches	No matches	No matches	No matches		No MS2	2436376.8
	No matches	No matches	No matches	No matches		No MS2	5100839
	No matches	No matches	No matches	No matches		No MS2	5592924.7
	No matches	No matches	No matches	No matches		No MS2	5070583.1
	No matches	No matches	No matches	No matches		No MS2	5578168
	No matches	No matches	No matches	No matches		No MS2	3144659.5
	No matches	No matches	No matches	No matches		No MS2	2942477.4
	No matches	No matches	No matches	No matches		No MS2	4498090.5
	No matches	No matches	No matches	No matches		No MS2	4155272.3
	No matches	No matches	No matches	No matches		No MS2	5037086.8
	No matches	No matches	No matches	No matches		No MS2	5282292
	No matches	No matches	No matches	No matches		No MS2	1002546
	No matches	No matches	Single match	No matches		No MS2	3495871.4
	No matches	No matches	No matches	No matches		No MS2	5259059.5
	Multiple ma	No matches	No matches	No matches		No MS2	2328744.6
	No matches	No matches	No matches	No matches		No MS2	2933087.5
	No matches	No matches	Multiple ma	No matches		No MS2	4035873.1
	No matches	No matches	No matches	No matches		No MS2	313971.68
	No matches	No matches	No matches	No matches		No MS2	5504670.2
	No matches	No matches	No matches	No matches		No MS2	5486539.6
	No matches	No matches	No matches	No matches		No MS2	2342240.8
	No matches	No matches	Multiple ma	No matches		No MS2	2916806
	No matches	No matches	No matches	No matches		No MS2	4140120.2
	No matches	No matches	No matches	No matches		No MS2	3337155.4
	No matches	No matches	No matches	No matches		No MS2	5472914.9
	No matches	No matches	No matches	Single match		No MS2	4315228.8
	No matches	No matches	Multiple ma	No matches		No MS2	4964038.1
	No matches	No matches	No matches	No matches		No MS2	5451267.9
	No matches	No matches	No matches	No matches		No MS2	3275701.6
	No matches	No matches	No matches	No matches		No MS2	2875569.2
	No matches	No matches	Multiple ma	Multiple ma		No MS2	4767515.3
	No matches	No matches	No matches	No matches		No MS2	2680464
	Multiple ma	No matches	Multiple ma	No matches		No MS2	5417739.9
	No matches	No matches	No matches	No matches		No MS2	4958771.2
	Multiple ma	No matches	No matches	No matches		No MS2	2879746.2
	No matches	No matches	No matches	No matches		No MS2	5381094.6
	No matches	No matches	No matches	No matches		No MS2	4518094.8

	No matches	No matches	No matches	No matches		No MS2	5063370.2
	No matches	No matches	No matches	No matches		No MS2	850695.23
	No matches	No matches	No matches	No matches		No MS2	5346010.2
	No matches	No matches	No matches	No matches		No MS2	4145170.8
	No matches	No matches	No matches	No matches		No MS2	5331336
	No matches	No matches	No matches	No matches		No MS2	3361543.9
	No matches	No matches	No matches	No matches		No MS2	4133664.5
	No matches	No matches	No matches	No matches		No MS2	5177823.4
	No matches	No matches	No matches	No matches		No MS2	490551.5
	No matches	Single match	No matches	No matches		No MS2	2644473.7
	No matches	No matches	No matches	No matches		DDA for pref	3294484.8
	No matches	No matches	No matches	No matches		No MS2	4208890.2
	No matches	No matches	No matches	No matches		No MS2	2949255.3
	No matches	Single match	No matches	No matches		No MS2	3867643.8
	No matches	No matches	No matches	No matches		No MS2	5281370.6
	No matches	No matches	No matches	No matches		No MS2	5278910.6
	No matches	No matches	No matches	No matches		No MS2	2111988.5
	No matches	No matches	No matches	No matches		No MS2	3044145.2
	No matches	No matches	No matches	No matches		No MS2	2145370.2
	No matches	No matches	No matches	No matches		No MS2	1312677.3
	No matches	No matches	No matches	No matches		No MS2	3085683.9
	No matches	No matches	No matches	No matches		No MS2	453159.76
	No matches	No matches	No matches	No matches		No MS2	3328726.8
	No matches	No matches	No matches	No matches		No MS2	5218059.9
	No matches	No matches	No matches	No matches		No MS2	1243264.7
	No matches	No matches	No matches	No matches		No MS2	1018721.5
	No matches	No matches	No matches	No matches		No MS2	468802.79
	No matches	No matches	No matches	No matches		No MS2	1184378.5
	No matches	No matches	No matches	No matches		No MS2	1114179.5
	No matches	No matches	No matches	No matches		No MS2	1877775.5
	No matches	No matches	No matches	Single match		No MS2	3644189.9
	No matches	No matches	No matches	No matches		No MS2	2647560.4
	No matches	No matches	No matches	No matches		No MS2	536025.47
	No matches	No matches	No matches	No matches		No MS2	2978482.6
	No matches	No matches	No matches	No matches		No MS2	5171556.7
	No matches	No matches	Single match	No matches		No MS2	5166266.8
	No matches	No matches	No matches	No matches		No MS2	3588002.1
	No matches	No matches	No matches	No matches		No MS2	2486516.5
	No matches	No matches	Multiple ma	No matches		No MS2	3430153.5
	No matches	No matches	No matches	No matches		No MS2	1466973.1
	No matches	No matches	No matches	No matches		No MS2	3471350
	No matches	No matches	No matches	No matches		No MS2	5126700.7
	No matches	No matches	No matches	No matches		No MS2	1979541.6
	No matches	No matches	No matches	No matches		No MS2	877598.18
	No matches	No matches	No matches	No matches		No MS2	4854222.3
	No matches	No matches	No matches	No matches		No MS2	1567187
	No matches	No matches	No matches	No matches		No MS2	5097469.9
	No matches	No matches	No matches	No matches		No MS2	1571223.2
	No matches	No matches	No matches	No matches		No MS2	2476020.9
	No matches	No matches	No matches	No matches		No MS2	3098852

	No matches	No matches	No matches	No matches		No MS2	5079357.2
	No matches	No matches	No matches	No matches		No MS2	4781926.1
	No matches	No matches	No matches	No matches		No MS2	2642314.6
	No matches	No matches	No matches	No matches		No MS2	3633874.6
	No matches	No matches	No matches	No matches		No MS2	463943.57
	No matches	No matches	No matches	No matches		No MS2	1155730.7
	No matches	No matches	No matches	No matches		No MS2	4893410.7
	No matches	No matches	No matches	No matches		No MS2	5019636
	No matches	No matches	No matches	No matches		No MS2	5013960.5
	No matches	No matches	No matches	No matches		No MS2	4907325.5
	No matches	No matches	No matches	No matches		No MS2	1864526.1
	No matches	No matches	No matches	No matches		No MS2	997837.97
	No matches	No matches	No matches	No matches		No MS2	4984767.9
	Multiple ma	No matches	No matches	No matches		No MS2	3004119.1
	No matches	No matches	No matches	No matches		No MS2	3546737.5
	No matches	No matches	No matches	No matches		No MS2	4963953.7
	Multiple ma	Multiple ma	Single match	No matches		No MS2	3553158.9
	Multiple ma	No matches	No matches	No matches		No MS2	4959175.4
	No matches	No matches	No matches	No matches		No MS2	3532388
	No matches	No matches	No matches	No matches		No MS2	3443992.7
	No matches	No matches	No matches	No matches		No MS2	3382762
	No matches	No matches	No matches	No matches		No MS2	3388600.1
	No matches	No matches	No matches	No matches		No MS2	854070.33
	No matches	No matches	No matches	No matches		No MS2	307832.83
	No matches	No matches	No matches	No matches		No MS2	3610313.1
	No matches	No matches	No matches	No matches		No MS2	505495.54
	No matches	No matches	No matches	No matches		No MS2	1111117.8
	No matches	No matches	No matches	No matches		No MS2	4871136.1
	No matches	No matches	No matches	No matches		No MS2	4865565.5
	No matches	No matches	No matches	No matches		No MS2	3563714.5
	No matches	No matches	No matches	No matches		No MS2	2243857.7
	No matches	No matches	No matches	Multiple ma		No MS2	4224301.6
	No matches	No matches	No matches	No matches		No MS2	2634279.8
	No matches	No matches	No matches	No matches		No MS2	1008780.4
	No matches	No matches	No matches	No matches		No MS2	4836677.1
	No matches	No matches	No matches	No matches		No MS2	2415886.8
	No matches	No matches	No matches	No matches		No MS2	4821868.4
	No matches	No matches	No matches	No matches		No MS2	389007.92
	No matches	No matches	No matches	No matches		No MS2	1211869.1
	No matches	No matches	No matches	No matches		No MS2	4783097.2
	No matches	No matches	No matches	No matches		No MS2	3696302.4
	No matches	No matches	Multiple ma	Single match		No MS2	4364578.8
	No matches	No matches	No matches	Single match		No MS2	3738796.6
	No matches	No matches	No matches	No matches		No MS2	2723314.4
	No matches	No matches	No matches	No matches		No MS2	3776389
	No matches	No matches	No matches	No matches		No MS2	3154086.7
	Multiple ma	No matches	No matches	No matches		No MS2	2968818.8
	No matches	No matches	No matches	No matches		No MS2	533893.11
	No matches	No matches	No matches	No matches		No MS2	329193.89
	No matches	No matches	No matches	No matches		No MS2	4103403.2

	No matches	No matches	No matches	No matches		No MS2	1158338.3
	No matches	No matches	No matches	No matches		No MS2	2201223
	No matches	No matches	No matches	No matches		No MS2	3028550.7
	No matches	No matches	No matches	No matches		No MS2	4540853.6
	No matches	No matches	No matches	No matches		No MS2	3447169.5
	Multiple ma	No matches	No matches	No matches		No MS2	4209476.1
	No matches	No matches	No matches	No matches		No MS2	492179.6
	No matches	No matches	No matches	No matches		No MS2	3340947.3
	No matches	No matches	No matches	No matches		No MS2	2637852.6
	No matches	No matches	No matches	No matches		No MS2	811677.42
	No matches	No matches	No matches	No matches		No MS2	3080411.6
	No matches	No matches	No matches	No matches		No MS2	1736401.3
	No matches	No matches	No matches	No matches		No MS2	2822760.9
	No matches	No matches	No matches	No matches		No MS2	4696378.5
	No matches	No matches	No matches	No matches		No MS2	3025389.4
	Multiple ma	No matches	Single match	No matches		No MS2	3587278.4
	Multiple ma	No matches	No matches	No matches		No MS2	601926.72
	No matches	No matches	No matches	No matches		No MS2	4653788
	No matches	No matches	No matches	No matches		No MS2	1109476.4
	No matches	No matches	No matches	No matches		No MS2	4633854.4
	Multiple ma	Single match	Multiple ma	No matches		No MS2	4632601.5
	No matches	No matches	No matches	No matches		No MS2	3889682.7
	No matches	No matches	No matches	No matches		No MS2	3029467.8
	No matches	No matches	No matches	No matches		No MS2	2726001.3
	No matches	No matches	No matches	No matches		No MS2	3769061.3
	No matches	No matches	No matches	No matches		No MS2	773793.81
	No matches	No matches	No matches	No matches		No MS2	2951080.6
	Multiple ma	No matches	Multiple ma	No matches		No MS2	3416888.3
	No matches	No matches	No matches	No matches		No MS2	3638018.1
	No matches	No matches	No matches	No matches		No MS2	2826635.5
	No matches	No matches	No matches	No matches		No MS2	3504929.5
	No matches	No matches	No matches	No matches		No MS2	3570897.3
	No matches	No matches	No matches	No matches		DDA for pref	3207132.8
	No matches	No matches	No matches	No matches		No MS2	3582503.5
	No matches	No matches	No matches	No matches		No MS2	2682596
	No matches	No matches	No matches	No matches		No MS2	2334306
	No matches	No matches	No matches	No matches		No MS2	312110.33
	No matches	No matches	No matches	No matches		No MS2	3866891.7
	No matches	No matches	No matches	No matches		No MS2	271314.66
	No matches	No matches	No matches	No matches		No MS2	1989481.1
	No matches	No matches	No matches	No matches		No MS2	2623843
	No matches	No matches	No matches	No matches		No MS2	3380827.4
	No matches	No matches	No matches	No matches		No MS2	3771129.8
	No matches	No matches	No matches	No matches		No MS2	361927.74
	No matches	No matches	No matches	No matches		No MS2	2573471.9
	No matches	No matches	No matches	No matches		No MS2	3282924.2
	No matches	No matches	No matches	No matches		No MS2	3247555.9
	Single match	No matches	Single match	No matches		No MS2	2850812.3
	No matches	No matches	No matches	No matches		No MS2	3452961.7
	Multiple ma	No matches	No matches	No matches		No MS2	2257990.2

	No matches	No matches	No matches	No matches		No MS2	4446299.1
	No matches	No matches	No matches	No matches		No MS2	1523151.2
	No matches	No matches	No matches	No matches		No MS2	1553564.9
	No matches	No matches	No matches	No matches		No MS2	2720552.4
	No matches	No matches	No matches	No matches		No MS2	4102868
	No matches	No matches	No matches	No matches		No MS2	791783.44
	No matches	No matches	No matches	No matches		No MS2	2402151.1
	No matches	No matches	No matches	No matches		No MS2	3730341.5
	No matches	No matches	No matches	No matches		No MS2	1989566.5
	No matches	No matches	No matches	No matches		No MS2	291803.88
	No matches	No matches	No matches	No matches		No MS2	3513188.5
	No matches	No matches	No matches	No matches		No MS2	3919918.5
	No matches	No matches	No matches	No matches		No MS2	1240990.4
	No matches	No matches	No matches	No matches		No MS2	3597494.4
	No matches	No matches	No matches	No matches		No MS2	2596684.2
	No matches	No matches	No matches	Single match		No MS2	3044218.6
	No matches	No matches	No matches	No matches		No MS2	3874287.3
	No matches	No matches	No matches	Single match		No MS2	2831920.2
	No matches	No matches	Multiple ma	No matches		No MS2	3385856.3
	No matches	No matches	No matches	No matches		No MS2	4279265.8
	No matches	No matches	No matches	No matches		No MS2	4271115.2
	No matches	No matches	No matches	No matches		No MS2	1313582.5
	No matches	No matches	No matches	No matches		No MS2	2737626.3
	No matches	No matches	No matches	No matches		No MS2	2829807.7
	No matches	No matches	No matches	No matches		No MS2	368681.93
	No matches	No matches	No matches	No matches		No MS2	4245260.5
	No matches	No matches	No matches	No matches		No MS2	1327313.4
	No matches	No matches	No matches	No matches		No MS2	1343980.5
	No matches	No matches	No matches	No matches		No MS2	2793650.5
	No matches	No matches	No matches	No matches		No MS2	2552009.1
	Multiple ma	No matches	No matches	No matches		No MS2	2082510.8
	No matches	No matches	No matches	No matches		No MS2	2560323.1
	No matches	No matches	No matches	No matches		No MS2	3171840.3
	No matches	No matches	No matches	No matches		No MS2	3563099.6
	No matches	No matches	No matches	No matches		No MS2	2674723.8
	No matches	No matches	No matches	No matches		No MS2	845224.01
	No matches	No matches	No matches	No matches		No MS2	3376152.9
	No matches	No matches	No matches	No matches		No MS2	3269105.9
	No matches	No matches	No matches	No matches		No MS2	4171991.6
	No matches	No matches	No matches	No matches		No MS2	2883842.2
	Multiple ma	No matches	No matches	No matches		No MS2	778560.91
	No matches	No matches	No matches	No matches		No MS2	1775140
	No matches	No matches	No matches	No matches		No MS2	2282518.5
	Multiple ma	No matches	Single match	No matches		No MS2	1247483.8
	No matches	No matches	No matches	No matches		No MS2	1457744.9
	No matches	No matches	No matches	No matches		No MS2	1841025.3
	No matches	No matches	No matches	No matches		No MS2	465515.07
	No matches	No matches	No matches	No matches		No MS2	2778654.9
	No matches	No matches	No matches	No matches		No MS2	3236726.2
	No matches	No matches	No matches	No matches		No MS2	1179955.4

	No matches	No matches	No matches	No matches		No MS2	3991302.3
	No matches	No matches	Multiple ma	No matches		No MS2	2353924.8
	No matches	No matches	No matches	No matches		No MS2	2454221.6
	No matches	No matches	Multiple ma	No matches		No MS2	3476304.1
	No matches	No matches	No matches	No matches		No MS2	3050276.7
	No matches	No matches	No matches	No matches		No MS2	2748447
	No matches	No matches	No matches	Single match		No MS2	3991940.3
	No matches	No matches	No matches	No matches		No MS2	1435373.9
	No matches	No matches	No matches	No matches		No MS2	1570276.5
	No matches	Single match	No matches	No matches		No MS2	1478224.8
	No matches	No matches	No matches	No matches		No MS2	2866050.5
	No matches	Single match	No matches	No matches		No MS2	3326782.6
	No matches	No matches	No matches	No matches		No MS2	2096253.3
	No matches	No matches	No matches	No matches		No MS2	2898048.8
	No matches	No matches	No matches	No matches		No MS2	3589387.6
	No matches	No matches	No matches	No matches		No MS2	1720093
	No matches	No matches	No matches	No matches		No MS2	2959588.2
	No matches	No matches	No matches	No matches		No MS2	3295072.7
	No matches	No matches	No matches	No matches		No MS2	353021.4
	No matches	No matches	No matches	No matches		No MS2	449911.77
	No matches	No matches	No matches	No matches		No MS2	2886639.8
	Multiple ma	No matches	No matches	No matches		No MS2	2305941.8
	No matches	No matches	No matches	No matches		No MS2	2049614.1
	No matches	No matches	No matches	No matches		No MS2	3117550.1
	No matches	No matches	No matches	No matches		No MS2	244096.38
	No matches	No matches	No matches	No matches		No MS2	2530661.7
	No matches	No matches	No matches	No matches		No MS2	2063388.5
	No matches	No matches	No matches	No matches		No MS2	1301262.1
	No matches	No matches	No matches	No matches		No MS2	791076.81
	No matches	No matches	No matches	No matches		No MS2	3809380.7
	No matches	No matches	No matches	No matches		No MS2	1893786.9
	No matches	No matches	Single match	No matches		DDA for pref	2172944.3
	No matches	No matches	No matches	No matches		No MS2	810263.25
	No matches	No matches	No matches	No matches		DDA for pref	834216.36
	No matches	No matches	No matches	No matches		No MS2	2651547.4
	No matches	No matches	Multiple ma	Single match		No MS2	3332827.9
	No matches	No matches	No matches	No matches		No MS2	3652068.8
	No matches	No matches	No matches	No matches		No MS2	2315375.9
	No matches	No matches	No matches	No matches		No MS2	98035.501
	No matches	No matches	No matches	No matches		No MS2	2693963
	No matches	No matches	No matches	No matches		No MS2	1766256.9
	No matches	No matches	No matches	No matches		No MS2	1514032.8
	No matches	No matches	No matches	No matches		No MS2	438835.08
	No matches	No matches	No matches	No matches		No MS2	1737647.8
	No matches	No matches	No matches	No matches		No MS2	2726256.5
	No matches	No matches	No matches	No matches		No MS2	1427216.2
	No matches	No matches	No matches	No matches		No MS2	2029450.3
	No matches	No matches	No matches	No matches		No MS2	3019779.5
	No matches	No matches	No matches	No matches		No MS2	927539.89
	No matches	No matches	No matches	No matches		No MS2	1797670.2

	Multiple ma	No matches	Multiple ma	No matches		No MS2	2301016.2
	No matches	No matches	No matches	No matches		No MS2	3656184.8
	No matches	No matches	No matches	No matches		No MS2	1621325.4
	No matches	No matches	No matches	No matches		No MS2	2310987.5
	No matches	No matches	No matches	No matches		No MS2	3624545.8
	No matches	No matches	No matches	No matches		No MS2	2275520.8
	No matches	No matches	No matches	No matches		No MS2	2678686.8
	Multiple ma	Single match	Multiple ma	No matches		No MS2	2656705.4
	No matches	No matches	No matches	No matches		No MS2	3610628.6
	No matches	No matches	No matches	No matches		No MS2	1621763.8
	No matches	No matches	No matches	No matches		No MS2	2803651.7
	No matches	No matches	No matches	No matches		No MS2	2680652.8
	No matches	No matches	No matches	No matches		No MS2	1320702.9
	Multiple ma	No matches	Single match	No matches		No MS2	2315828.5
	No matches	No matches	No matches	No matches		No MS2	709074.71
	No matches	No matches	No matches	No matches		No MS2	2647692.5
	No matches	No matches	No matches	No matches		No MS2	1944338.4
	No matches	No matches	No matches	No matches		No MS2	1207368.1
	No matches	No matches	No matches	No matches		No MS2	1675833.3
	No matches	No matches	No matches	No matches		No MS2	1079050.8
	No matches	No matches	No matches	No matches		No MS2	3504929.5
	No matches	No matches	No matches	No matches		No MS2	2378468.2
	No matches	No matches	No matches	No matches		No MS2	2558209.7
	No matches	No matches	No matches	No matches		No MS2	2702807
	No matches	No matches	No matches	No matches		No MS2	319991.09
	No matches	No matches	No matches	No matches		No MS2	2668123.3
	No matches	No matches	No matches	No matches		No MS2	860390.83
	No matches	No matches	No matches	No matches		No MS2	2074146
	No matches	No matches	No matches	No matches		No MS2	2305309.5
	Multiple ma	No matches	No matches	No matches		No MS2	731098.06
	No matches	No matches	No matches	No matches		No MS2	1037474.8
	No matches	Single match	No matches	No matches		No MS2	2018473.6
	No matches	No matches	No matches	No matches		No MS2	3454037.3
	No matches	No matches	No matches	No matches		No MS2	3351744.2
	No matches	No matches	No matches	No matches		No MS2	3434121.8
	No matches	No matches	No matches	No matches		No MS2	775967.83
	No matches	No matches	No matches	No matches		No MS2	1998904.5
	No matches	No matches	No matches	No matches		No MS2	3299838.8
	No matches	No matches	No matches	No matches		No MS2	3416642.1
	No matches	No matches	No matches	No matches		No MS2	3404780.2
	No matches	Multiple ma	No matches	No matches		No MS2	1083647.9
	No matches	No matches	No matches	No matches		No MS2	1109161.8
	No matches	No matches	No matches	No matches		No MS2	1807354
	No matches	No matches	No matches	No matches		No MS2	1516213.3
	No matches	No matches	No matches	No matches		No MS2	1652451.8
	No matches	No matches	No matches	No matches		No MS2	1772141
	No matches	No matches	No matches	No matches		No MS2	3360736.2
	No matches	No matches	No matches	No matches		No MS2	2543957
	No matches	Single match	No matches	No matches		No MS2	2443020.8
	No matches	No matches	Single match	No matches		No MS2	2230547

	No matches	No matches	No matches	No matches		No MS2	972200.9
	No matches	No matches	No matches	No matches		No MS2	2498069.7
	No matches	No matches	No matches	No matches		No MS2	309927.99
	No matches	No matches	No matches	No matches		No MS2	3288283.2
	No matches	No matches	No matches	No matches		No MS2	2173393.8
	No matches	No matches	No matches	No matches		No MS2	262942.92
	No matches	No matches	No matches	No matches		No MS2	1067066.8
	Single match	No matches	No matches	No matches		No MS2	3254483.2
	No matches	No matches	No matches	No matches		No MS2	2276170.9
	No matches	No matches	No matches	No matches		No MS2	2303981.7
	No matches	No matches	No matches	No matches		No MS2	1088465.7
	No matches	No matches	No matches	No matches		No MS2	1498794.3
	No matches	No matches	No matches	No matches		No MS2	2426181.8
	No matches	No matches	No matches	No matches		No MS2	2492850.7
	No matches	No matches	No matches	No matches		No MS2	172867.08
	No matches	No matches	No matches	No matches		No MS2	521178.67
	No matches	No matches	No matches	No matches		No MS2	998867.94
	No matches	No matches	No matches	No matches		No MS2	1728419.9
	No matches	No matches	No matches	No matches		No MS2	1248344.4
	No matches	No matches	No matches	No matches		No MS2	967810.95
	No matches	No matches	No matches	No matches		No MS2	2221901.3
	No matches	No matches	No matches	No matches		No MS2	445298.29
	No matches	No matches	No matches	No matches		No MS2	2931030.1
	No matches	No matches	No matches	No matches		No MS2	1316297.1
	No matches	No matches	No matches	No matches		No MS2	2748566.1
	No matches	No matches	No matches	No matches		No MS2	292196.08
	No matches	No matches	No matches	No matches		No MS2	506899.78
	No matches	No matches	No matches	No matches		No MS2	881026.5
	No matches	No matches	No matches	No matches		No MS2	346228.75
	No matches	No matches	No matches	No matches		No MS2	3035587.7
	No matches	No matches	No matches	No matches		No MS2	1895469.1
	No matches	No matches	No matches	No matches		No MS2	243112.12
	No matches	No matches	No matches	No matches		No MS2	2154587.1
	No matches	No matches	No matches	No matches		No MS2	611431.03
	No matches	No matches	No matches	No matches		No MS2	1197073.5
	No matches	No matches	No matches	No matches		No MS2	500049.87
	No matches	No matches	No matches	No matches		DDA for pref	947028.91
	No matches	No matches	No matches	No matches		No MS2	2938347.8
	No matches	No matches	No matches	No matches		No MS2	1188132.9
	No matches	No matches	No matches	No matches		No MS2	461385.4
	Multiple ma	No matches	Single match	No matches		No MS2	1796721.8
	No matches	No matches	No matches	No matches		No MS2	2931046
	No matches	No matches	No matches	No matches		No MS2	2768482.3
	No matches	No matches	No matches	No matches		No MS2	2915537.3
	Single match	No matches	No matches	No matches		No MS2	576618.77
	No matches	No matches	No matches	No matches		No MS2	2741275.2
	No matches	No matches	No matches	No matches		No MS2	1794625
	No matches	No matches	No matches	No matches		DDA for pref	697206.05
	No matches	No matches	No matches	No matches		No MS2	1648194
	No matches	No matches	No matches	No matches		No MS2	754243.49

	No matches	No matches	No matches	No matches		No MS2	1458261.5
	No matches	No matches	No matches	No matches		No MS2	525899.82
	No matches	No matches	No matches	No matches		No MS2	355035.11
	No matches	No matches	No matches	No matches		No MS2	2853439.9
	No matches	No matches	No matches	No matches		No MS2	517982.41
	No matches	No matches	No matches	No matches		No MS2	1607653.4
	No matches	No matches	Single match	No matches		No MS2	673567.88
	No matches	No matches	Single match	Single match		No MS2	1796920.5
	No matches	No matches	No matches	No matches		No MS2	1794590.8
	No matches	No matches	No matches	No matches		No MS2	1762684.9
	No matches	No matches	No matches	No matches		No MS2	267973.82
	No matches	No matches	No matches	No matches		No MS2	1093121.6
	No matches	No matches	No matches	No matches		No MS2	296402.54
	No matches	No matches	No matches	No matches		No MS2	733318.87
	No matches	No matches	No matches	No matches		No MS2	1687079.5
	No matches	No matches	No matches	No matches		No MS2	2176719.9
	No matches	Single match	No matches	No matches		No MS2	473241.87
	No matches	No matches	No matches	No matches		No MS2	1715552
	No matches	No matches	No matches	No matches		No MS2	274913.46
	No matches	No matches	No matches	No matches		No MS2	2435835.4
	No matches	No matches	No matches	No matches		No MS2	2578831.1
	Multiple ma	No matches	Single match	No matches		No MS2	287121.58
	No matches	No matches	No matches	No matches		No MS2	1930948.2
	No matches	No matches	No matches	No matches		No MS2	2673712.4
	No matches	No matches	No matches	No matches		No MS2	1929026.3
	Single match	No matches	No matches	No matches		No MS2	1053596.5
	No matches	No matches	No matches	No matches		No MS2	1028183
	No matches	Single match	No matches	No matches		No MS2	2314893.6
	No matches	No matches	No matches	No matches		No MS2	429025.81
	No matches	No matches	No matches	No matches		No MS2	2086961
	No matches	No matches	No matches	No matches		No MS2	2157862.1
	No matches	No matches	No matches	No matches		No MS2	788914.83
	No matches	No matches	No matches	No matches		No MS2	1243697.5
	No matches	No matches	No matches	No matches		No MS2	219203.13
	No matches	No matches	No matches	No matches		No MS2	1440806.1
	No matches	No matches	No matches	No matches		No MS2	289078.75
	No matches	No matches	No matches	No matches		No MS2	1747979.7
	No matches	No matches	No matches	No matches		No MS2	1717289.4
	No matches	No matches	No matches	No matches		No MS2	1887427.7
	No matches	No matches	No matches	No matches		No MS2	1658809.9
	No matches	No matches	No matches	No matches		No MS2	627105.42
	No matches	No matches	No matches	No matches		No MS2	290346.41
	No matches	No matches	No matches	No matches		No MS2	1694027.8
	No matches	No matches	No matches	No matches		No MS2	538529.85
	No matches	No matches	No matches	No matches		No MS2	2403934.3
	No matches	No matches	No matches	No matches		DDA for othe	2399305.7
	No matches	No matches	No matches	No matches		No MS2	1894370.7
	No matches	No matches	No matches	No matches		No MS2	2382665.3
	No matches	No matches	No matches	No matches		No MS2	1524646.5
	No matches	No matches	No matches	No matches		No MS2	2343329.4

	No matches	No matches	No matches	No matches		No MS2	228295.11
	No matches	No matches	No matches	No matches		No MS2	1263784.7
	No matches	No matches	No matches	No matches		No MS2	2257700.4
	No matches	No matches	No matches	No matches		No MS2	128349.04
	No matches	No matches	No matches	No matches		No MS2	2131469.5
	No matches	No matches	Multiple ma	No matches		No MS2	717851.51
	No matches	No matches	No matches	No matches		No MS2	1291287.6
	No matches	No matches	No matches	No matches		No MS2	230977.76
	No matches	Single match	No matches	No matches		No MS2	1604639.3
	No matches	No matches	No matches	No matches		No MS2	1222474.4
	No matches	No matches	Single match	Multiple ma		No MS2	1351196.2
	No matches	No matches	No matches	No matches		No MS2	1681845.6
	No matches	No matches	No matches	No matches		No MS2	1685878.9
	No matches	No matches	No matches	No matches		No MS2	1048775.6
	No matches	No matches	No matches	No matches		No MS2	543719.74
	No matches	No matches	No matches	No matches		No MS2	1028179.3
	No matches	No matches	No matches	No matches		No MS2	204019.3
	No matches	No matches	No matches	No matches		No MS2	117352.37
	Multiple ma	No matches	No matches	No matches		No MS2	1916821.1
	No matches	No matches	No matches	No matches		No MS2	264622.45
	No matches	No matches	No matches	No matches		No MS2	909304.74
	No matches	No matches	No matches	No matches		No MS2	1426918.7
	No matches	No matches	No matches	No matches		No MS2	688813.94
	No matches	No matches	No matches	No matches		No MS2	331712.55
	No matches	No matches	No matches	No matches		No MS2	911698.91
	No matches	No matches	No matches	No matches		No MS2	1229063.6
	No matches	No matches	No matches	No matches		No MS2	202642.44
	No matches	No matches	No matches	No matches		No MS2	1079612
	No matches	No matches	No matches	No matches		No MS2	253324.1
	No matches	No matches	No matches	No matches		No MS2	552229.79
	No matches	No matches	No matches	No matches		No MS2	1305772.9
	No matches	No matches	No matches	No matches		No MS2	194895.57
	No matches	No matches	No matches	No matches		No MS2	863335.15
	No matches	No matches	No matches	Multiple ma		DDA for othe	1585669.7
	No matches	No matches	No matches	No matches		No MS2	434697.1
	No matches	No matches	No matches	No matches		No MS2	791661.32
	No matches	No matches	No matches	No matches		No MS2	389868.46
	No matches	No matches	No matches	No matches		No MS2	1566082.5
	No matches	No matches	No matches	No matches		No MS2	1338754.7
	No matches	No matches	No matches	No matches		No MS2	904625.53
	No matches	No matches	No matches	No matches		No MS2	237897.52
	No matches	No matches	No matches	No matches		No MS2	806570.22
	No matches	No matches	No matches	No matches		No MS2	165405.89
	No matches	No matches	No matches	No matches		No MS2	203655.32

Group Area:	Group CV [%	Group CV [%	Ratio: (b) / (a	Log2 Fold Change: (b) / (a)
1.191E+11		11.370533	0.88	-0.18
3.75E+10		2.800465	8.357	3.06
3.741E+10		2.770785	0.865	-0.21
1.879E+10		6.6031976	1.031	0.04
1.596E+10		53.848301	0.932	-0.1
8.446E+09		15.474188	2.911	1.54
8.245E+09		4.2895119	0.93	-0.1
6.96E+09		50.590189	1.062	0.09
7.219E+09		8.2264749	31.924	5
6.524E+09		9.1710555	0.924	-0.11
5.872E+09		5.116721	1.102	0.14
5.3E+09		11.939139	0.779	-0.36
5.383E+09		6.006876	13.173	3.72
5.308E+09		7.1595984	1.063	0.09
3.706E+09		47.914534	0.744	-0.43
3.507E+09		40.827722	1.048	0.07
3.567E+09		11.665428	0.883	-0.18
3.963E+09		5.6339154	1.022	0.03
3.724E+09		19.064145	0.817	-0.29
3.899E+09		3.3521931	0.96	-0.06
3.815E+09		3.1128433	0.967	-0.05
3.712E+09		2.7537027	0.916	-0.13
3.263E+09		14.100552	0.754	-0.41
3.278E+09		5.2969515	0.877	-0.19
3.265E+09		3.2325684	1.076	0.11
3.269E+09		11.73606	0.93	-0.11
2.923E+09		3.8438614	0.907	-0.14
3.003E+09		3.8416561	1.22	0.29
2.899E+09		54.170893	0.945	-0.08
2.568E+09		10.587152	0.725	-0.46
2.569E+09		7.6675663	0.727	-0.46
2.864E+09		3.1701648	0.957	-0.06
2.788E+09		1.9941458	0.95	-0.07
2.699E+09		2.8578448	0.862	-0.21
2.259E+09		6.6901097	0.715	-0.48
1.985E+09		54.333359	0.898	-0.16
1.95E+09		54.45937	0.893	-0.16
2.228E+09		7.9216615	0.953	-0.07
2.183E+09		11.781672	0.824	-0.28
2.254E+09		6.3789188	0.958	-0.06
2.308E+09		1.2600758	0.884	-0.18
1.975E+09		49.228728	1.201	0.26
2.191E+09		7.5696777	3.581	1.84
2.024E+09		10.204446	0.813	-0.3
1.953E+09		59.136743	0.909	-0.14
1.974E+09		11.126216	0.781	-0.36
1.777E+09		27.177773	1.502	0.59
1.905E+09		8.0147627	1.09	0.12

Only

1.124E+09		20.414717	0.485	-1.04
1.112E+09		14.844393	0.487	-1.04
1.53E+09		42.04022	1.236	0.31
1.824E+09		2.7992037	0.896	-0.16
983902127		27.822338	0.448	-1.16
1.745E+09		11.804486	0.798	-0.33
1.687E+09		8.2906541	0.879	-0.19
1.467E+09		18.972774	3.605	1.85
1.644E+09		8.5257621	0.798	-0.33
1.717E+09		16.115301	1.034	0.05
1.473E+09		8.6450685	1.131	0.18
1.452E+09		57.758472	1.057	0.08
1.283E+09		10.800391	0.873	-0.2
1.498E+09		66.456757	111.601	6.8
1.467E+09		3.5260892	0.925	-0.11
1.46E+09		2.349067	0.963	-0.05
1.154E+09		35.949462	1.176	0.23
1.467E+09		4.2646775	0.846	-0.24
1.362E+09		7.0278647	0.991	-0.01
734617016		114.26731	49.74	5.64
1.343E+09		10.444599	0.759	-0.4
1.405E+09		10.993206	0.969	-0.04
1.402E+09		3.7532381	0.87	-0.2
1.359E+09		5.6991409	1.013	0.02
1.378E+09		5.7041559	0.99	-0.01
1.375E+09		19.71588	1332.48	10.38
1.345E+09		59.645215	0.933	-0.1
1.203E+09		6.2044497	1.081	0.11
902288583		23.316215	208.615	7.7
1.2E+09		7.9132841	0.935	-0.1
96321913		66.486173	0.063	-3.99
1.173E+09		12.86172	1.127	0.17
880267615		3.3505882	0.596	-0.75
911166157		11.314911	0.619	-0.69
712996993		63.922846	0.729	-0.46
1.087E+09		66.602566	69.649	6.12
1.193E+09		3.9277399	0.934	-0.1
1.098E+09		31.043752	0.832	-0.26
1.121E+09		1.147368	1.034	0.05
1.069E+09		59.813316	0.936	-0.09
1.08E+09		3.9153827	1.063	0.09
1.003E+09		6.8335691	1.001	0
906629477		11.423303	1.094	0.13
980764065		6.5711157	1.058	0.08
965660443		11.043259	1.034	0.05
959524289		11.25737	1.039	0.06
953294410		11.453202	1.038	0.05
949647950		11.388019	37.537	5.23
927067836		8.8479993	0.742	-0.43
924602634		8.9451947	0.742	-0.43

Only

913256977		16.143699	0.985	-0.02
1.019E+09		4.1167308	0.922	-0.12
736232703		40.825192	1.885	0.91
963368384		6.5012365	0.894	-0.16
964775632		2.7766507	0.939	-0.09
927343520		6.0726977	433.211	8.76
846709156		9.8791303	0.88	-0.18
21396240		8.5274509	0.019	-5.68
904506186		3.1174338	1.033	0.05
564473092		28.463868	0.515	-0.96
880463957		3.9753352	1	0
816363571		12.437117	0.826	-0.28
741934803		10.26849	0.908	-0.14
814814213		6.881563	0.778	-0.36
604178268		27.842856	0.994	-0.01
854342990		3.3162694	1.002	0
223985283		6.8774717	0.215	-2.22
804152249		34.948086	1.514	0.6
785907232		3.8406665	0.878	-0.19
567846844		5.1756573	0.556	-0.85
648960276		5.2870731	0.646	-0.63
603508233		36.460022	2.034	1.02
771962620		6.8052101	1.631	0.71
523804062		34.528939	1.131	0.18
779277729		4.0030318	1.15	0.2
776905091		2.7831583	1.193	0.25
335541.11		22.728977	0	-11.49
650457592		4.7447152	0.679	-0.56
765219510		1.1965624	0.884	-0.18
746835383		8.7313533	0.888	-0.17
748583681		5.1071107	7.82	2.97
746420088		2.4053893	0.878	-0.19
470773989		40.528078	1.398	0.48
736831114		5.5857545	1.042	0.06
713396304		4.2757583	1.022	0.03
699316412		4.0828601	0.77	-0.38
591311852		24.014004	0.946	-0.08
699019882		14.329654	1.085	0.12
571786400		26.116757	0.829	-0.27
731280684		3.048149	1.157	0.21
217401484		10.547344	0.244	-2.03
96035842		43.029355	0.109	-3.2
585573299		14.72939	1.046	0.07
722505724		1.5861425	0.93	-0.1
671433422		3.5170136	0.913	-0.13
640741650		8.9107197	1.376	0.46
244281896		75.275999	0.289	-1.79
662507598		6.109114	0.959	-0.06
487262322		63.151814	0.746	-0.42
663289230		4.2158604	0.882	-0.18

Only

668583045		7.2965	1.022	0.03
626624388		17.241002	1.127	0.17
476380227		36.654432	0.957	-0.06
647610505		12.807718	0.783	-0.35
642115919		3.1213269	0.912	-0.13
665664550		2.8931646	0.95	-0.07
634324053		3.1636652	0.911	-0.13
548821692		7.271867	0.681	-0.55
490214243		56.335194	0.958	-0.06
611974293		6.2243044	1	0
541578544		7.3570199	0.681	-0.55
539542786		3.6862393	0.684	-0.55
567423836		19.666755	0.786	-0.35
575236628		4.0110915	0.739	-0.44
600649820		4.6926115	0.859	-0.22
626588458		2.7657337	0.93	-0.1
346430341		24.422387	0.449	-1.15
589966846		43.873692	1.357	0.44
354086006		16.315151	0.465	-1.11
549309021		3.875288	0.732	-0.45
560969889		8.1952611	1.543	0.63
562674864		7.0583198	1.131	0.18
362108947		36.337347	2.092	1.06
589858766		2.556661	0.986	-0.02
580032842		9.3762444	0.885	-0.18
448259566		68.199548	64.935	6.02
3550457.3		15.917185	0.005	-7.67
514903239		7.9779229	0.713	-0.49
547150661		12.816863	0.9	-0.15
585981387		3.5149817	0.93	-0.1
476701036		16.410514	0.665	-0.59
543470245		2.9159137	0.762	-0.39
409531604		6.5438715	0.58	-0.78
547370240		6.2426823	1.036	0.05
517588499		6.3485125	0.745	-0.42
303198519		12.138028	0.439	-1.19
534667396		7.6389165	1.246	0.32
514133455		7.5679162	0.81	-0.3
527877963		6.2487347	0.888	-0.17
291362778		11.796478	0.437	-1.19
518813883		7.7822692	1.071	0.1
240875890		30.377865	0.366	-1.45
521015409		50.992568	1.388	0.47
233278831		106.00168	0.431	-1.22
526670338		2.7498927	2.912	1.54
232071711		50.211411	1.26	0.33
285256326		42.566915	0.445	-1.17
484821040		17.499513	1.008	0.01
474196882		7.0015607	1.098	0.14
502786983		11.128779	1.413	0.5

482236694		17.64245	1.117	0.16
451616328		49.750617	1.183	0.24
369163208		17.339763	0.736	-0.44
431298914		54.025134	1.107	0.15
437966742		7.1961389	1.159	0.21
388534928		17.46652	1.202	0.27
463721974		15.351597	1.106	0.15
464251564		4.4171427	1.079	0.11
24026110		173.40875	5.499	2.46
364972933		7.0335824	0.633	-0.66
457510535		1.8616799	1.1	0.14
443560882		5.6547385	1.235	0.3
336887179		15.61344	0.599	-0.74
442699763		6.5979546	1.392	0.48
428473529		10.364967	0.804	-0.31
440133228		5.3823798	1.087	0.12
434272717		10.919786	0.951	-0.07
355291958		8.7233154	0.638	-0.65
393501034		10.508626	0.865	-0.21
73932059		119.03733	1.531	0.61
318955546		7.884346	0.577	-0.79
372428124		29.118677	2.125	1.09
446716237		2.1086135	1.003	0.01
397191128		22.172674	0.976	-0.03
409829958		5.0687746	0.89	-0.17
404689840		8.4222854	1.164	0.22
419705477		10.103381	0.99	-0.01
441049892		6.8327858	1.085	0.12
413960976		6.1672768	0.992	-0.01
420207010		5.5042667	0.93	-0.1
436104936		1.1555122	0.913	-0.13
435688898		1.3974844	0.896	-0.16
129943434		13.518389	0.246	-2.03
421220037		5.0067451	0.906	-0.14
426701811		1.3292185	0.916	-0.13
411227163		4.7497726	0.858	-0.22
417407962		4.6980217	0.812	-0.3
403793234		4.199814	0.808	-0.31
367718490		12.499544	1.371	0.46
398383651		5.7111067	1.294	0.37
394982568		4.9102903	0.94	-0.09
324361307		41.35599	12.223	3.61
400068916		3.4320395	1.496	0.58
298648630		13.290572	0.6	-0.74
350918034		15.34275	0.902	-0.15
336495219		27.68769	0.716	-0.48
248233759		39.648989	128.744	7.01
166431321		40.132314	0.336	-1.58
393602573		10.587245	0.996	-0.01
294270402		20.718153	3.424	1.78

Only

380492965		11.671399	1.184	0.24
109485843		40.629353	0.225	-2.15
374580721		8.9682604	1.1	0.14
359358283		7.3847165	2.863	1.52
370229457		4.2174537	0.926	-0.11
369971900		4.760766	0.807	-0.31
65650195		135.37599	59.41	5.89
382031753		3.3689996	1.294	0.37
195638459		15.038172	0.422	-1.24
342640218		10.548535	0.99	-0.01
285327000		33.554887	2.628	1.39
313382146		54.185852	0.855	-0.23
362729318		4.4892847	0.92	-0.12
368119412		2.4921996	1.027	0.04
370181780		2.3306244	0.973	-0.04
362059700		5.0149193	1.004	0.01
361953267		3.6951795	69.892	6.13
278341958		12.90613	0.625	-0.68
352730256		4.6963025	1.001	0
345735119		9.6345975	1.278	0.35
362500548		2.7868975	0.976	-0.03
352802549		5.1325288	1.137	0.19
350577102		5.3923324	0.826	-0.28
303677644		69.068778	89.456	6.48
359171964		4.9984703	0.895	-0.16
179021222		114.43156	0.486	-1.04
350008962		9.6515539	0.804	-0.31
345177958		4.9676007	1.152	0.2
317974763		14.233495	0.737	-0.44
315793857		10.164548	0.976	-0.03
168385924		74.914275	1.222	0.29
321913534		7.1604723	0.897	-0.16
334075049		8.6232683	0.876	-0.19
333013928		6.4363395	1.009	0.01
313539799		13.00096	0.822	-0.28
332160327		6.7634826	0.921	-0.12
185481369		14.439966	0.446	-1.17
159263027		28.458962	0.383	-1.38
341670216		4.5581567	0.893	-0.16
159054400		27.534638	0.385	-1.38
258031989		32.132222	0.783	-0.35
326622164		46.72518	0.982	-0.03
331109875		1.052024	1.115	0.16
340821660		4.2631494	0.908	-0.14
228124038		7.2838786	0.56	-0.84
328697384		1.0531973	1.112	0.15
299544814		13.411197	0.922	-0.12
327159664		3.5236193	1.08	0.11
298951806		9.553434	1.417	0.5
282986792		12.65887	0.731	-0.45

Only

328752737		4.3510117	1.206	0.27
316839931		7.7707375	1.081	0.11
298278150		6.0821503	0.752	-0.41
2912482.3		18.575657	0.007	-7.08
302747187		6.9151126	19.599	4.29
315191353		1.8302627	1.33	0.41
184026461		58.399772	0.711	-0.49
239668683		31.674965	8.911	3.16
311770712		2.6950538	0.857	-0.22
308279227		1.53575	0.959	-0.06
305336562		4.6078364	0.853	-0.23
261604470		10.420935	0.7	-0.51
291241005		5.3995611	0.798	-0.33
283631489		13.279679	1.347	0.43
284930891		12.112086	0.823	-0.28
264618754		10.089876	0.763	-0.39
298779692		5.7053291	0.83	-0.27
293486176		1.9151276	0.879	-0.19
130303210		9.0183626	0.364	-1.46
222470954		23.518716	0.623	-0.68
283499609		6.8118298	0.859	-0.22
282514602		10.432007	0.795	-0.33
261310557		9.3949897	0.846	-0.24
250559099		27.576204	1.123	0.17
93468909		26.088168	0.264	-1.92
253286152		9.6894607	0.969	-0.05
287452815		4.6517824	0.903	-0.15
259953453		13.533646	1.404	0.49
253025863		12.022364	0.96	-0.06
274441787		28.412201	1.328	0.41
269735643		6.3902118	0.923	-0.12
271490315		3.4250857	0.988	-0.02
247241004		22.272175	0.732	-0.45
200890376		29.607886	0.779	-0.36
262627775		60.867017	1.201	0.26
273830760		13.901068	1.15	0.2
86768529		39.929927	0.252	-1.99
280829537		2.1129899	0.902	-0.15
287095055		4.6706651	0.858	-0.22
277385381		3.1464659	1.057	0.08
232703811		11.819356	0.735	-0.44
270748568		4.515888	0.919	-0.12
264871876		7.5116269	0.835	-0.26
167631800		28.056689	0.498	-1.01
255195844		8.1772348	1.165	0.22
21814089		34.324064	0.065	-3.94
264830196		7.4969658	0.898	-0.16
260470709		6.6490035	0.925	-0.11
266425686		3.8333423	1.092	0.13
261940864		5.9669123	0.899	-0.15

Only

262499267		17.199611	1.043	0.06
265105653		4.8403924	0.912	-0.13
262045250		9.0865199	1.016	0.02
250176936		7.9647672	0.969	-0.05
209037525		24.719214	0.641	-0.64
217612754		16.707303	0.925	-0.11
246050326		9.8791175	0.892	-0.17
228520877		6.9125069	0.702	-0.51
258344735		19.430294	1.031	0.04
253895081		4.4429084	1.401	0.49
244113758		6.2491527	1.078	0.11
248097043		6.309485	0.993	-0.01
262575423		3.7309277	1.35	0.43
249320369		6.2743803	0.942	-0.09
217820550		11.150184	1.013	0.02
246168003		5.4591118	1.074	0.1
238969576		7.7405941	1.193	0.25
208162885		27.018208	1.137	0.18
233746703		7.0268166	0.953	-0.07
255978803		43.297522	1.001	0
126257170		17.326542	0.405	-1.3
217657748		9.7329827	1.246	0.32
246462668		4.9420678	1.014	0.02
249731071		4.8741231	0.873	-0.2
201173215		37.413888	0.783	-0.35
233946957		9.3688544	0.855	-0.23
209372218		18.04278	0.877	-0.19
40315962		135.94653	18.045	4.17
189580988		21.566161	68.31	6.09
211016515		61.29326	0.688	-0.54
224988654		7.7884396	1.441	0.53
200528806		14.868745	0.726	-0.46
109239871		49.38873	0.364	-1.46
166439987		35.859289	1.078	0.11
234310135		2.7351898	1.239	0.31
215401760		18.810313	0.841	-0.25
192933423		19.157095	1.027	0.04
145249617		20.858118	0.492	-1.02
163517881		22.910449	0.998	0
211835637		5.2582618	0.722	-0.47
222381848		59.847846	1.102	0.14
72018213		121.97943	22.847	4.51
208580183		17.001179	1.224	0.29
230904650		5.2052699	0.79	-0.34
226086284		3.0136544	0.793	-0.33
226184183		19.190976	1.294	0.37
215952911		14.869269	0.871	-0.2
233522482		2.7036036	1.242	0.31
231322213		4.9931139	0.805	-0.31
226012167		5.5590362	0.958	-0.06

Only

237627256		1.9835301	1.032	0.05
197507757		12.070204	0.989	-0.02
229946780		2.53666	0.933	-0.1
219267848		3.2270885	1.023	0.03
107174790		41.836848	0.378	-1.4
172540344		30.73745	0.921	-0.12
228508402		5.7495902	0.904	-0.15
95225916		27.007012	0.342	-1.55
173771937		39.833517	1.036	0.05
83932092		18.520543	0.307	-1.7
213217633		6.6249913	0.896	-0.16
205773837		9.5360433	0.869	-0.2
172055798		39.873921	1.035	0.05
212962536		2.4276623	0.787	-0.35
192699620		21.97307	141.433	7.14
161642136		7.4731374	0.6	-0.74
215912402		59.436355	0.95	-0.07
209859128		7.8122737	1.168	0.22
92491918		28.092254	0.346	-1.53
167080740		20.924074	1.728	0.79
166424957		16.586507	187.382	7.55
214323271		5.8261112	0.884	-0.18
218287459		6.91084	1.072	0.1
203877799		10.349057	1.312	0.39
203877799		10.349057	1.312	0.39
186330647		60.033305	1.204	0.27
203324818		9.455457	3.487	1.8
204725541		7.3365329	1.227	0.3
135941272		6.0159643	0.528	-0.92
174035635		18.361304	1.021	0.03
1486993.9		19.247114	0.006	-7.43
202614793		5.9590682	0.84	-0.25
203961648		1.9714335	1.018	0.03
200571510		6.7940322	0.803	-0.32
195276385		7.0721412	1.084	0.12
189533144		5.3624994	0.887	-0.17
179650735		35.18037	1.057	0.08
190514328		4.5578274	0.938	-0.09
199570276		8.94649	0.899	-0.15
35396907		66.261675	0.142	-2.82
92459068		32.843857	0.371	-1.43
190820223		5.9357049	1.218	0.28
155437971		26.256604	0.866	-0.21
8744062.2		177.89023	0.533	-0.91
74754702		23.214288	0.304	-1.72
191929109		4.0322118	0.901	-0.15
166902741		18.469788	0.827	-0.27
167712912		20.95095	0.769	-0.38
3103151.3		190.0766	1.381	0.47
186197061		6.6286366	1.01	0.02

Only

145776184		19.080583	0.987	-0.02
188571950		2.4527331	1.039	0.05
185226773		3.1787964	0.78	-0.36
14400854		13.887701	0.061	-4.04
89991831		109.82988	0.582	-0.78
149732523		37.58494	2.23	1.16
195473570		2.7230619	0.917	-0.13
144711441		37.101217	1.137	0.18
136677071		45.642057	1.383	0.47
187830759		7.4127429	0.992	-0.01
170309805		15.586321	0.895	-0.16
158836803		10.900614	0.882	-0.18
12396697		126.38463	0.053	-4.24
163170554		5.769606	0.697	-0.52
147580739		7.7545832	0.635	-0.66
180173720		5.3551026	0.897	-0.16
173611453		9.5546673	0.993	-0.01
95006825		114.75361	0.446	-1.16
137140195		71.969206	46.774	5.55
140225400		8.4767522	0.614	-0.7
180774770		5.236152	1.065	0.09
182671415		3.2034484	0.951	-0.07
126096535		5.8949871	0.554	-0.85
184166062		5.4726225	1.068	0.09
166831716		9.9432653	0.802	-0.32
54851545		82.194	0.901	-0.15
180129977		7.5006582	1.055	0.08
178905440		9.5901577	0.809	-0.31
183286012		2.1123266	0.958	-0.06
90243812		54.33848	0.402	-1.31
171210532		8.6363871	0.775	-0.37
166055080		5.6957438	0.874	-0.19
179479630		9.1189474	0.901	-0.15
152129360		15.976481	2.492	1.32
158157667		8.627505	0.831	-0.27
158282427		14.097299	0.985	-0.02
179400075		13.619377	1.511	0.6
163853441		17.79523	1.581	0.66
85943698		101.49141	0.39	-1.36
114231389		32.5306	2.092	1.06
168919074		5.4564162	0.888	-0.17
112946987		34.510408	1.29	0.37
180819984		8.7480008	0.833	-0.26
167408269		10.138806	0.959	-0.06
169927596		7.4719808	1.284	0.36
146592311		16.958772	1.165	0.22
136539131		20.825286	0.665	-0.59
158392911		12.385343	0.729	-0.46
160429903		18.11069	1.465	0.55
158532805		7.0601989	0.808	-0.31

Only

174233621		6.3660709	0.82	-0.29
172510820		7.9325055	0.954	-0.07
174220914		6.0929505	0.83	-0.27
121436744		6.9175551	0.568	-0.82
126431675		60.901105	90.829	6.51
161783822		5.6784056	0.869	-0.2
88937572		14.338659	0.42	-1.25
155236844		13.462861	0.89	-0.17
171370741		3.5861269	0.875	-0.19
162502594		5.0453766	0.891	-0.17
158350843		21.824311	0.911	-0.14
165014821		7.5200625	1.366	0.45
156559689		7.9095995	1.241	0.31
130400078		23.796953	0.744	-0.43
127234424		47.188807	1.178	0.24
142982774		21.78952	0.746	-0.42
147370036		53.999639	1.229	0.3
129418427		16.66691	1.149	0.2
71307961		36.138318	0.346	-1.53
156926568		6.3349242	0.894	-0.16
145921075		9.5739614	0.959	-0.06
165210363		2.1452022	0.84	-0.25
148658297		14.683547	0.934	-0.1
30403800		54.451244	0.149	-2.74
154479321		5.811651	0.897	-0.16
154479321		5.811651	0.897	-0.16
160033896		6.7475518	0.85	-0.23
158568961		8.6821032	0.866	-0.21
142743496		11.315252	0.904	-0.15
150469069		7.2585702	1.212	0.28
99638559		79.224868	20.662	4.37
156087546		5.5972732	1.647	0.72
103900537		9.5758032	0.527	-0.92
161391489		0.4148088	1.231	0.3
149179614		8.268508	1.267	0.34
156964662		11.827999	1.098	0.14
142188613		13.785272	0.726	-0.46
155187246		3.7582013	1.01	0.01
97940662		41.849516	0.502	-0.99
156078264		3.4845579	0.817	-0.29
158218061		5.6826936	1.047	0.07
136839193		17.032996	0.742	-0.43
140829963		17.878178	0.886	-0.18
145180029		12.620095	0.892	-0.17
155583421		7.4510106	0.881	-0.18
153758995		7.7005104	3.645	1.87
92360492		33.827099	0.535	-0.9
146059881		7.8295515	1.4	0.49
144288654		5.575	1.214	0.28
139230526		8.7246804	1.066	0.09

Only

145570242		9.1543942	0.915	-0.13
89286102		24.514007	0.47	-1.09
145565815		4.3284097	1.017	0.02
125291533		28.962894	2.832	1.5
97437957		10.365533	0.515	-0.96
26232380		131.84963	10.243	3.36
148709515		7.4606839	27.682	4.79
153471463		2.2109875	1.018	0.03
128305235		69.354621	21.71	4.44
102842643		10.381866	0.548	-0.87
116591357		27.487675	0.936	-0.1
149471530		7.126353	0.85	-0.24
151300242		7.4266859	1.074	0.1
144867125		7.8175977	1.085	0.12
7430623.4		174.80172	5.15	2.36
134101679		7.6318608	0.786	-0.35
8915669.8		64.615867	0.049	-4.36
27026556		115.31378	0.148	-2.76
89867150		71.34889	0.718	-0.48
141456707		9.4681593	1.218	0.28
114061248		18.296611	0.747	-0.42
101842169		34.768645	0.755	-0.4
140933138		5.3901595	0.781	-0.36
144968469		2.3248768	0.971	-0.04
123955351		13.628989	1.035	0.05
137364060		6.8005079	0.764	-0.39
143829548		9.0444614	0.81	-0.3
141020054		6.0257569	0.949	-0.08
141679455		59.404486	1.083	0.11
147065354		7.8787682	0.887	-0.17
127103788		8.5404843	0.976	-0.04
143263230		5.7149832	1.133	0.18
87478396		27.596448	0.491	-1.03
44777586		118.05249	4.076	2.03
134330145		9.8416951	1.173	0.23
125781778		10.108426	1.062	0.09
142534161		8.1945609	1.169	0.23
126050041		17.08018	1.012	0.02
139064201		6.1043874	0.813	-0.3
88294516		32.255776	0.504	-0.99
132926595		16.008553	0.946	-0.08
132926595		16.008553	0.946	-0.08
138928102		4.4511607	1.147	0.2
133044225		5.5067281	0.981	-0.03
132243695		9.363014	1.369	0.45
132136941		14.571498	1.065	0.09
139336083		8.8081291	1.255	0.33
117259858		7.1604783	0.679	-0.56
130732106		20.093441	0.853	-0.23
130362216		12.009845	1.057	0.08

Only

126282431		12.693983	1.238	0.31
113602929		57.843543	0.84	-0.25
108313568		9.7182713	0.633	-0.66
140204136		0.4871253	0.867	-0.21
136191289		10.04188	0.879	-0.19
132670995		8.438303	1.361	0.44
61615127		35.062303	0.361	-1.47
136034855		4.1208873	1.161	0.22
111087883		28.606133	0.673	-0.57
131889229		6.7781088	1.041	0.06
122962630		9.1068883	0.724	-0.47
130083484		6.3266154	0.875	-0.19
130241327		12.921024	0.771	-0.38
30992998		114.91022	0.29	-1.79
98542924		38.544487	0.655	-0.61
60038981		29.747822	0.357	-1.48
3518932.4		15.096248	0.021	-5.57
127418984		6.6172144	0.761	-0.39
134707589		3.1758575	9.597	3.26
121094424		11.33156	1.187	0.25
116188244		18.525092	1.225	0.29
130151595		7.8040434	0.836	-0.26
95771541		20.313349	0.767	-0.38
71216136		35.23392	0.429	-1.22
75255987		13.120609	0.453	-1.14
131198573		5.3151277	86.122	6.43
13600644		8.9093266	0.083	-3.6
106735902		55.94536	0.742	-0.43
65585273		18.090936	0.399	-1.33
134913929		4.8922844	0.876	-0.19
122532601		6.0111673	0.819	-0.29
87498260		34.816299	1.172	0.23
117196119		35.033594	0.792	-0.34
128370286		5.3819452	1.089	0.12
115186524		9.6605388	0.96	-0.06
131622496		5.89535	1.214	0.28
120684313		8.9608696	1.225	0.29
127875659		5.2092889	0.957	-0.06
4216466.1		30.980566	0.026	-5.25
103221086		7.1008015	0.641	-0.64
72150763		61.751715	0.449	-1.15
92540933		11.994196	0.577	-0.79
47078887		19.132769	0.294	-1.77
123197853		10.890571	1.119	0.16
62845049		106.87731	26.763	4.74
92540521		46.592445	0.878	-0.19
118948094		9.0334475	0.823	-0.28
95648241		16.562057	0.805	-0.31
116279416		9.9171157	0.761	-0.39
103502928		22.384127	1.849	0.89

Only

110638220		25.579499	0.93	-0.1
116933155		7.5123233	0.891	-0.17
118386695		5.863445	1.637	0.71
92802703		3.403218	0.591	-0.76
113633442		9.8989743	2.531	1.34
63876175		4.6666504	0.409	-1.29
104347738		22.503955	0.669	-0.58
111686188		10.444696	1.108	0.15
87225783		9.0000332	0.562	-0.83
118050775		6.0996765	0.806	-0.31
123347913		2.9557651	1.24	0.31
100528178		18.131353	0.833	-0.26
75387976		14.911491	0.488	-1.03
120149712		5.4247455	1.271	0.35
91441729		23.101769	0.923	-0.12
115256767		8.5805637	1.551	0.63
125660005		4.4891919	0.95	-0.07
117563124		7.8251319	1.286	0.36
123945231		4.4598295	0.841	-0.25
95407626		15.58561	0.879	-0.19
114082324		5.3070811	1.194	0.26
107949513		22.561384	0.711	-0.49
74308850		15.457162	0.49	-1.03
72888974		4.7599788	0.482	-1.05
108609556		10.099845	0.718	-0.48
117693274		8.3827053	8.214	3.04
93934034		18.705813	0.621	-0.69
102650474		8.5460817	0.679	-0.56
118674010		6.61252	1.453	0.54
116981324		12.116624	0.932	-0.1
105766197		17.995331	1.027	0.04
74366673		36.3192	1.042	0.06
54392300		55.033271	1.644	0.72
119590928		7.0008427	1.025	0.04
85874705		26.180158	1.457	0.54
115612668		7.344435	0.894	-0.16
121115288		11.693267	1.011	0.02
107208495		66.027067	51.344	5.68
45038603		22.797678	0.304	-1.72
23314694		2.0618556	0.157	-2.67
113653651		9.5993637	0.836	-0.26
119997781		4.4863131	1.566	0.65
112241913		5.1422055	1.056	0.08
117583784		3.9055174	0.863	-0.21
106450135		1.4053768	0.723	-0.47
116293920		5.8385577	1.047	0.07
104872716		20.296917	1.481	0.57
98593650		3.7402614	0.67	-0.58
118964483		7.5477714	0.97	-0.04
85197062		24.391691	0.604	-0.73

Only

113768514		4.6823996	1.048	0.07
115834048		11.072135	0.853	-0.23
101133348		10.3725	1.211	0.28
57589047		35.429355	0.394	-1.34
97816808		7.9539404	0.671	-0.58
75531131		18.828506	0.518	-0.95
105853774		9.0141064	1.106	0.15
96439924		12.151434	0.664	-0.59
116000437		18.281045	0.888	-0.17
92659941		19.557011	0.784	-0.35
104372595		7.9652677	0.733	-0.45
80053279		26.330054	1.369	0.45
107665501		10.867737	0.814	-0.3
103244174		10.091959	0.928	-0.11
96693989		12.223263	1.066	0.09
103441319		66.34977	72.134	6.17
106509010		11.978576	8.179	3.03
68179649		75.377729	0.786	-0.35
114213346		6.4049867	0.885	-0.18
99759680		10.954417	2.362	1.24
80083914		23.366172	0.899	-0.15
110315232		7.8819786	1.147	0.2
93563281		13.84793	0.943	-0.08
109061129		39.195406	1.066	0.09
8223027.4		147.62666	0.059	-4.09
62219847		7.6335734	0.446	-1.16
110918857		5.1508226	1.071	0.1
97945312		10.791495	0.703	-0.51
103791892		8.181214	1.043	0.06
110841543		3.6313279	1.003	0
51983349		108.31035	0.508	-0.98
104627257		5.7561937	1.08	0.11
87518739		19.852018	0.966	-0.05
78198438		49.948133	0.565	-0.82
83481047		4.7758257	0.604	-0.73
85774551		34.727374	0.7	-0.51
108871820		4.0770554	1.235	0.31
48505384		31.507186	0.353	-1.5
96744714		8.9659826	0.705	-0.5
106880880		6.1286394	1.006	0.01
91114652		11.939166	2.048	1.03
73069737		39.210367	1.721	0.78
7162253.1		10.128053	0.052	-4.26
89140794		19.461195	0.653	-0.62
104672421		4.1368372	0.768	-0.38
107652632		16.358705	0.824	-0.28
26286524		23.767216	0.194	-2.37
109727136		4.8273525	1.022	0.03
93110315		15.660404	1.133	0.18
86431155		15.340842	15.979	4

Only

108099702		1.8140089	0.941	-0.09
40764454		35.384039	0.305	-1.71
101917356		11.012104	1.313	0.39
104843862		7.4248015	27.644	4.79
87034150		59.540361	0.858	-0.22
104950335		52.536589	0.86	-0.22
106468820		6.2625842	0.8	-0.32
107605533		3.6633884	1.001	0
101659061		5.4439982	0.765	-0.39
45134839		112.96901	6.928	2.79
63675907		40.131829	0.48	-1.06
75673667		38.306089	0.572	-0.81
108169085		6.6639517	0.925	-0.11
96867936		9.9888457	1.168	0.22
94641028		20.551758	1.12	0.16
97002302		5.5062055	1	0
96805773		11.337196	0.856	-0.22
19564094		45.140686	0.15	-2.74
90331836		16.58421	1.813	0.86
96568497		19.655587	1.45	0.54
100413610		6.2053405	1.04	0.06
90117342		5.7723456	0.695	-0.52
103103499		6.0072865	0.857	-0.22
83014138		70.173656	19.138	4.26
105801930		1.8244206	1.09	0.12
102424360		6.5977212	0.899	-0.15
97692149		5.9312078	0.923	-0.12
102353868		7.8422128	1.789	0.84
105500501		3.5192407	0.885	-0.18
102291836		10.236887	0.956	-0.06
91559019		22.019923	0.773	-0.37
86383711		39.085266	2.476	1.31
55984135		39.31275	1.131	0.18
86443679		3.0308247	0.675	-0.57
102484820		4.6888008	2.575	1.36
2290780.7		185.1093	0.018	-5.8
96956004		9.4897945	1.612	0.69
101540632		4.8346462	0.885	-0.18
95108398		28.428589	1.218	0.28
79780185		28.958471	0.72	-0.47
95056599		14.735426	0.998	0
96558005		15.334864	5.018	2.33
67373248		47.419826	0.532	-0.91
99022801		5.8968125	0.97	-0.04
101694579		4.7631381	1.258	0.33
97744174		7.4025719	0.973	-0.04
96705929		7.6997202	1.094	0.13
101481302		12.062732	3.497	1.81
96957332		64.612651	41.148	5.36
99058632		5.637735	0.925	-0.11

Only

95872278		8.356485	27.292	4.77
78674507		7.7548755	0.629	-0.67
52672116		109.99548	6.434	2.69
98798657		6.8806333	0.797	-0.33
63474047		53.783757	1.209	0.27
52227429		23.747054	0.42	-1.25
97769525		2.9623612	0.893	-0.16
85701655		28.241678	1.169	0.22
97817829		5.0951014	1.131	0.18
98339026		1.6500372	1.078	0.11
55233676		16.366896	0.449	-1.16
2024628.6		187.81533	2.268	1.18
58433847		22.261699	0.476	-1.07
99462454		4.4684572	95.62	6.58
91028545		15.670118	4.91	2.3
54506022		9.0691274	0.445	-1.17
86819702		10.642816	1.205	0.27
99828299		3.5956307	0.995	-0.01
84554985		21.342879	72.112	6.17
91365460		4.4757878	0.753	-0.41
72676450		13.69183	0.599	-0.74
95558387		3.534683	1.127	0.17
86058030		8.3866122	0.944	-0.08
100868880		6.4439641	0.93	-0.1
93539592		9.1403969	0.777	-0.36
97675676		10.430834	0.864	-0.21
97085032		2.997127	0.933	-0.1
96911378		3.3557069	1.077	0.11
97128702		6.5736935	0.891	-0.17
84570282		12.998375	0.856	-0.22
90610984		12.877271	0.859	-0.22
90930913		7.0772049	1.514	0.6
89948976		9.9285366	1.085	0.12
94045339		7.758492	0.808	-0.31
91777543		9.3687725	1.094	0.13
91752968		7.6547391	0.993	-0.01
97919826		2.8361854	1.026	0.04
96155687		5.0608775	1.285	0.36
92405403		6.9201465	0.996	-0.01
70958145		23.905173	0.982	-0.03
89003172		9.4209417	1.084	0.12
92930135		3.8516239	1.051	0.07
92796418		9.8443042	0.901	-0.15
33782145		27.93002	0.29	-1.79
87132530		8.2865402	0.943	-0.09
85530239		15.339053	2.249	1.17
70966769		22.382826	0.613	-0.71
9465974.9		146.52329	0.814	-0.3
84308456		17.844581	1.977	0.98
64885417		36.135521	0.563	-0.83

Only

91868768		3.4385457	1.09	0.12
82421180		10.498368	0.91	-0.14
81909550		14.618235	1.422	0.51
85689449		10.240727	0.808	-0.31
83411480		9.2181551	0.879	-0.19
69971029		32.600245	0.667	-0.58
88732904		3.6604176	0.972	-0.04
81075512		12.862901	1.176	0.23
90793788		4.4428363	1.028	0.04
92587676		1.4024252	0.811	-0.3
2288602.3		18.053484	0.02	-5.64
82344239		15.004971	1.04	0.06
93702346		3.5759173	0.929	-0.11
78183484		12.386108	0.977	-0.03
92128176		2.6278253	0.967	-0.05
88590770		6.5507567	1.339	0.42
87274712		12.235922	0.826	-0.28
71954416		2.4490499	0.637	-0.65
89418534		5.4271071	1.193	0.25
82934206		18.000382	1.259	0.33
87656989		8.2955131	1.07	0.1
15571752		103.15602	0.138	-2.86
67141298		4.3046142	0.596	-0.75
83189577		15.840296	2.438	1.29
75163492		70.37534	66.403	6.05
79940267		16.369275	1.223	0.29
92759187		2.4134306	1.068	0.09
88974110		4.2154848	0.86	-0.22
58692169		5.1343424	0.525	-0.93
85178749		7.0410378	1.171	0.23
76078236		61.299935	1.054	0.08
90162287		5.7979137	0.964	-0.05
84298155		2.3648237	0.758	-0.4
81860690		13.074973	1.189	0.25
85169152		6.0937748	0.899	-0.15
89605194		9.4536334	0.919	-0.12
85946594		12.193843	0.904	-0.15
41276895		12.873799	0.374	-1.42
79317168		14.160019	0.992	-0.01
86227600		5.7719783	0.811	-0.3
84791844		9.8258376	3.529	1.82
64483092		18.043291	1.719	0.78
83321900		11.611174	1.72	0.78
59876080		22.852211	0.549	-0.87
80895921		17.995729	2.278	1.19
83394615		9.7084524	1.794	0.84
61245924		10.720595	0.564	-0.83
85738314		4.747899	0.908	-0.14
54131415		5.68449	0.499	-1
82043674		11.431726	0.983	-0.03

Only

77347657		12.886982	1.115	0.16
75680777		29.76101	52.959	5.73
63627521		27.684726	1.963	0.97
86034521		10.327083	0.996	-0.01
61639961		8.0666931	0.572	-0.81
48455694		52.871781	0.45	-1.15
72980539		11.335101	1.008	0.01
66311613		49.465991	3.555	1.83
54542619		37.676071	0.745	-0.42
83360199		11.048399	0.873	-0.2
44219828		104.14874	86.56	6.44
82267268		6.0556972	1.01	0.01
16590780		38.602939	0.155	-2.69
55097862		15.084832	0.516	-0.96
59486688		41.832049	0.862	-0.22
40652960		64.94575	0.382	-1.39
78932503		7.9085021	0.926	-0.11
78334075		6.3163027	0.781	-0.36
76184259		2.7836165	0.717	-0.48
81890423		8.0929149	1.048	0.07
83163489		16.800046	1	0
68802974		33.245078	27.621	4.79
33852404		65.77437	0.32	-1.64
81636556		25.798038	4.54	2.18
79151404		13.279447	2.474	1.31
66106758		35.78759	1.71	0.77
74267455		15.37149	1.707	0.77
76561380		12.070345	1.674	0.74
77460823		13.690107	3.001	1.59
83556252		5.6454859	1.19	0.25
77814531		10.078148	0.859	-0.22
79342201		6.3956438	0.868	-0.2
59734324		23.045524	0.819	-0.29
80037487		7.0736593	1.009	0.01
85091451		5.3226273	0.84	-0.25
77624026		15.171399	1.275	0.35
79856094		4.7081391	1.038	0.05
80576331		6.9607134	1.02	0.03
80317619		10.192399	1.036	0.05
51451783		19.92565	0.497	-1.01
77121092		12.093266	1.019	0.03
59028642		27.87925	0.574	-0.8
24920934		119.74116	3.698	1.89
79719692		8.48285	1.074	0.1
47814333		98.559759	0.55	-0.86
52590083		30.391503	0.938	-0.09
67781452		8.5244939	0.66	-0.6
50541154		68.380596	0.494	-1.02
8009361.2		18.724129	0.078	-3.68
66758094		62.453258	8.699	3.12

Only

78324700		11.357431	1.159	0.21
77674757		7.2086431	1.657	0.73
81945300		3.5627672	0.936	-0.1
12589220		15.769388	0.124	-3.01
79172194		15.239938	1.194	0.26
75387148		13.066424	3.749	1.91
68773435		19.307265	1.054	0.08
63907971		21.907386	1.371	0.45
78542347		3.9934018	0.775	-0.37
77179500		3.5902935	1.398	0.48
77849651		10.368688	1.018	0.03
76741976		10.176979	1.006	0.01
26156311		56.020265	0.261	-1.94
80190687		3.8662048	0.892	-0.17
27370043		89.063546	0.273	-1.87
75846873		19.550166	1.848	0.89
64141366		23.982654	0.814	-0.3
42931276		37.429181	0.43	-1.22
80569633		1.4241668	0.954	-0.07
699896.85		29.317795	0.007	-7.15
61742437		49.286497	0.787	-0.35
61680227		46.318804	1.22	0.29
72515815		16.569781	1.391	0.48
76644981		5.3766428	4.432	2.15
54214994		33.625571	23.98	4.58
81254129		5.0650512	0.907	-0.14
63689078		40.147416	0.945	-0.08
79222876		2.2174367	0.926	-0.11
39286694		28.855715	0.397	-1.33
61695190		68.664761	140.668	7.14
79270847		4.5200748	1.194	0.26
70416851		52.604815	0.964	-0.05
74132612		6.5532363	1.668	0.74
49210233		43.09391	0.553	-0.85
75151942		4.4587723	1.231	0.3
76775956		45.628243	10.175	3.35
80369366		4.3581613	0.866	-0.21
76635704		5.7961913	0.781	-0.36
78088995		3.4686866	1.095	0.13
56995092		23.366391	1.017	0.02
66008685		11.892813	1.227	0.3
70674820		18.276102	1.288	0.36
73822407		13.933622	1.105	0.14
44334386		25.056875	0.454	-1.14
56927935		7.3071723	0.584	-0.78
68502962		18.493059	1.233	0.3
69359018		9.4956007	0.9	-0.15
77162206		5.1213162	0.939	-0.09
72575061		22.132695	1.53	0.61
38161030		12.165404	0.393	-1.35

Only

72080258		8.6777219	1.429	0.51
71983446		18.76795	1.043	0.06
74463589		5.7828975	0.848	-0.24
22724264		42.63415	0.236	-2.08
65220457		30.828379	36.487	5.19
71775885		19.003531	1.494	0.58
54658566		9.934868	0.568	-0.82
76597950		13.535935	1.065	0.09
60092361		46.803161	1.013	0.02
74564689		6.7034077	1.297	0.37
76498636		3.3665636	1.32	0.4
75140752		4.5431849	0.978	-0.03
6265649.8		152.53325	0.965	-0.05
66639459		14.037801	26.36	4.72
71424756		7.4526876	1.595	0.67
61078688		8.1580009	0.645	-0.63
77437062		2.172688	0.899	-0.15
71957343		10.32092	0.963	-0.05
67162674		14.596143	0.758	-0.4
72187918		9.4514657	2.431	1.28
69587456		16.038075	0.938	-0.09
57608409		55.031745	1.259	0.33
4422874.5		58.427919	0.047	-4.41
5669798.5		163.20372	3.901	1.96
73953874		19.145429	1.02	0.03
28258335		41.822185	0.302	-1.73
73224392		8.5079252	1.276	0.35
74526129		4.6300143	0.856	-0.22
61410968		2.1766705	0.661	-0.6
28124874		11.360853	0.303	-1.72
71391247		5.9995674	1.03	0.04
77468604		3.0023265	0.901	-0.15
66210913		12.112175	1.195	0.26
76103307		3.0781418	0.933	-0.1
56761732		28.411618	0.942	-0.09
68457575		28.051171	2.409	1.27
73437861		3.8133103	0.962	-0.06
71311087		7.7567092	1.069	0.1
65653691		14.709041	0.992	-0.01
22058869		100.13091	0.24	-2.06
66223701		20.448518	5.238	2.39
66267883		14.612243	0.836	-0.26
61011930		50.728606	0.769	-0.38
74100605		4.0125918	0.931	-0.1
68568048		61.171809	0.989	-0.02
37606012		22.96521	0.411	-1.28
68466380		11.106753	0.943	-0.08
73555665		5.4666584	0.811	-0.3
73871088		2.566952	0.971	-0.04
45705002		47.480612	1.1	0.14

Only

72709999		6.6907978	0.893	-0.16
58652102		15.957779	1.036	0.05
59347644		28.698731	46.177	5.53
73281534		4.1980607	1.424	0.51
34280305		46.657525	0.378	-1.4
65661310		65.849242	58.884	5.88
62498618		27.464496	0.961	-0.06
8826906.6		49.596393	0.098	-3.35
64744098		7.9171738	0.887	-0.17
51884188		28.5094	2.338	1.23
71717275		6.2644458	1.055	0.08
58768516		15.497195	1.742	0.8
66842160		19.099247	0.871	-0.2
74021938		2.4386632	1.044	0.06
71601757		5.8524673	1.104	0.14
52321348		21.270178	0.743	-0.43
65294253		20.835912	1.604	0.68
64099678		26.53954	1.252	0.32
68155030		5.6543634	0.991	-0.01
37732899		27.152738	0.425	-1.23
61121443		26.780255	1.022	0.03
72483609		3.3916696	1.088	0.12
70084046		3.9635951	1.815	0.86
61276876		13.420618	0.751	-0.41
56265637		22.208869	1.44	0.53
68482060		9.0671401	0.894	-0.16
52366644		5.5974672	0.599	-0.74
68923124		6.7383551	1.192	0.25
66849140		8.8252108	0.875	-0.19
58493033		10.771012	1.206	0.27
64675759		11.890024	0.861	-0.22
69697915		3.1268498	0.843	-0.25
33758507		13.543116	0.389	-1.36
64656159		14.821313	0.823	-0.28
56145189		15.338115	1.269	0.34
67152315		8.1139759	1.072	0.1
62431989		14.44667	1.084	0.12
68415474		7.0270995	1.234	0.3
53549992		17.96963	0.985	-0.02
68251962		6.969854	0.815	-0.3
55526558		18.649021	1.534	0.62
63338921		12.761033	0.735	-0.44
60418080		15.999757	0.792	-0.34
2555120.6		76.651562	0.03	-5.07
67205273		6.5393409	0.869	-0.2
68731933		6.0653675	0.917	-0.13
71225559		8.4883629	0.963	-0.05
62510545		6.6311447	1.132	0.18
64924068		6.6978547	0.847	-0.24
63374339		17.38118	1.978	0.98

Only

68015781		6.7786303	1.126	0.17
10403533		137.7802	2.112	1.08
68085540		7.0788223	1.208	0.27
68265720		11.150174	1.031	0.04
65746377		16.093467	1.319	0.4
69026046		7.1630885	0.843	-0.25
60364627		16.398948	0.713	-0.49
21797399		22.219351	0.257	-1.96
62841066		9.047448	15.752	3.98
1022311.1		189.36933	0.867	-0.21
56758546		11.861778	4.779	2.26
57964902		18.760189	0.784	-0.35
39036092		39.131578	0.828	-0.27
68235650		7.4964207	0.914	-0.13
66756791		6.3737987	1.003	0
67001538		3.3978742	0.977	-0.03
25822872		30.213551	0.308	-1.7
53987773		21.741296	1.921	0.94
64880215		7.5356036	0.945	-0.08
67335928		4.045237	1.114	0.16
63996755		33.915456	1.108	0.15
57987879		10.096518	0.832	-0.27
44408840		31.281035	1.603	0.68
47238056		41.955731	1.224	0.29
65153268		5.6417646	0.913	-0.13
66042839		3.5330773	1.083	0.11
46887770		63.220504	0.835	-0.26
48563629		68.448063	29.131	4.86
66672358		5.4352203	1.353	0.44
66328505		3.4386316	1.058	0.08
52412938		34.397634	40.649	5.35
68338375		1.7856436	0.85	-0.23
1107353.9		15.054188	0.013	-6.22
54270624		35.574659	56.531	5.82
59878446		11.166439	0.937	-0.09
68489418		11.457876	1.17	0.23
64251552		5.5893378	0.911	-0.13
64509357		5.5089643	0.923	-0.12
44318132		42.909822	12.699	3.67
41370030		37.854546	0.685	-0.55
59169870		27.78546	61.957	5.95
68243500		26.056163	1.25	0.32
50580239		26.264283	1.32	0.4
39887886		37.181307	1.124	0.17
66025114		8.3196742	0.837	-0.26
59519150		42.208497	0.844	-0.24
43354394		18.667759	0.536	-0.9
61149394		7.9051519	0.782	-0.36
52809323		16.091522	0.952	-0.07
60899588		18.660979	0.975	-0.04

Only

60846369		6.7185303	0.873	-0.2
52155145		23.57688	1.664	0.73
56409924		10.265034	2.122	1.09
63008434		7.3822213	0.815	-0.29
62794265		3.205113	1.074	0.1
36210799		10.677722	0.452	-1.14
59041976		8.3315749	0.879	-0.19
58590287		14.50447	0.937	-0.09
61427342		6.8864442	0.908	-0.14
60934424		13.183394	0.98	-0.03
53819251		21.44982	1.131	0.18
57553474		11.049091	0.744	-0.43
43985815		46.486605	0.552	-0.86
58155366		11.998926	1.021	0.03
63824287		5.7173514	18.725	4.23
65092319		3.3222362	1.13	0.18
40453671		9.5328965	0.509	-0.97
27540974		16.111749	0.347	-1.53
58007333		15.446342	1.077	0.11
65498415		2.0604529	1.004	0.01
57706559		10.898776	0.956	-0.06
62491314		4.7052787	1.209	0.27
46246532		27.65651	1.063	0.09
61567334		6.6475565	1.06	0.08
54503880		25.171305	0.896	-0.16
40587132		27.553422	0.754	-0.41
64656654		6.6709083	1.253	0.33
53839979		12.256776	0.773	-0.37
22429023		96.592139	9.175	3.2
52378250		3.5486189	0.667	-0.58
59546911		10.877054	1.093	0.13
61238354		6.2438513	1	0
50941511		20.213378	1.053	0.08
63985597		8.1524225	1.068	0.1
64954406		18.471866	1.442	0.53
62953862		5.718022	4.046	2.02
56190642		9.2255732	2.851	1.51
58560953		10.672269	1.17	0.23
56111574		27.009414	26.711	4.74
62220066		59.451287	1.113	0.15
26297093		8.7653814	0.34	-1.56
49839410		13.38942	1.033	0.05
59699187		12.149446	1.06	0.08
59877179		8.2993991	1.136	0.18
58473606		7.0202723	0.823	-0.28
53155023		12.154637	40.214	5.33
764592.9		191.71141	0.076	-3.72
58809575		10.933015	1.093	0.13
49882798		17.010006	0.652	-0.62
62855419		4.2673174	1.028	0.04

Only

49557058		27.895687	1.211	0.28
47216650		20.221011	1.157	0.21
54338624		12.357737	1.183	0.24
57172895		11.714942	1.02	0.03
56012253		8.4296267	0.739	-0.44
52853423		11.765136	0.697	-0.52
41720587		8.8046544	0.551	-0.86
58580382		13.325566	0.82	-0.29
59866741		12.519785	1.099	0.14
41765990		23.828684	1.006	0.01
58119657		7.5928757	1.222	0.29
51104603		17.154238	0.927	-0.11
49133557		12.369505	1.011	0.02
57797656		16.944688	0.784	-0.35
36841773		68.571557	0.491	-1.02
34818484		23.197846	0.465	-1.11
49231538		9.8656593	0.658	-0.6
57466205		2.6209627	0.77	-0.38
30736307		15.612605	0.412	-1.28
49135550		55.946741	1.212	0.28
50837423		32.710027	0.958	-0.06
48303770		11.298248	0.65	-0.62
59054452		5.2408787	0.838	-0.25
50830003		11.871743	2.938	1.56
51556848		11.335097	1.187	0.25
57517991		16.833618	43.51	5.44
53240295		9.6121634	1.353	0.44
54462741		65.30119	28.752	4.85
60741475		5.6060812	0.85	-0.24
51525999		14.871278	0.955	-0.07
48656252		48.717728	6.002	2.59
53193180		29.58715	49.812	5.64
59487270		4.6954886	18.345	4.2
53024974		23.117285	2.311	1.21
58295541		4.4770359	0.85	-0.23
56011776		17.044292	0.986	-0.02
55710298		10.296369	1.936	0.95
595251.18		28.689731	0.008	-6.94
53216968		17.938736	0.882	-0.18
59002376		1.7640795	1.026	0.04
56445733		3.9426071	0.772	-0.37
44721388		18.472505	1.128	0.17
52847572		9.1836311	0.767	-0.38
38819639		19.534413	0.531	-0.91
1840393.9		106.10467	0.025	-5.31
56598856		7.7420098	1.237	0.31
59971054		6.7034577	1.401	0.49
32417794		24.314564	0.445	-1.17
56322474		5.6003006	1.016	0.02
57207567		6.459023	1.17	0.23

Only

57054653		11.96576	1.324	0.4
56031905		7.556973	1.187	0.25
56235829		13.868016	0.98	-0.03
55864462		21.186959	1.311	0.39
58784198		2.8867409	0.961	-0.06
26330355		43.102008	0.363	-1.46
51113177		16.079964	0.834	-0.26
52752855		13.279186	1.155	0.21
58558438		5.0873204	1.029	0.04
58070921		4.0696313	1.092	0.13
42932421		20.677368	0.917	-0.13
14244032		18.604242	0.197	-2.34
52740308		15.613972	1.371	0.46
36940815		27.599613	0.635	-0.66
50828351		20.829934	1.259	0.33
58734226		5.6910217	0.985	-0.02
44998548		32.647938	1.271	0.35
51607059		7.7217039	0.808	-0.31
44537630		65.415983	20.057	4.33
16080944		16.99513	0.225	-2.15
42970523		19.73365	0.602	-0.73
54717215		10.5115	1.027	0.04
41090217		14.450222	0.576	-0.8
55441348		3.2769448	1.034	0.05
52511099		8.9871281	0.737	-0.44
55788010		6.2467823	0.976	-0.03
57723757		6.4742539	0.995	-0.01
54955426		11.985046	0.89	-0.17
55855497		7.9310701	0.922	-0.12
57888450		6.3233726	0.817	-0.29
46089968		15.590617	0.841	-0.25
53684684		10.806578	2.244	1.17
55074470		12.962861	1.094	0.13
51768990		8.8547486	1.296	0.37
51768990		8.8547486	1.3	0.38
49164798		4.5462425	0.7	-0.52
49205027		12.854889	0.809	-0.31
54287804		3.7768519	1.052	0.07
53932090		5.9848399	1.315	0.39
55714730		6.9577666	1.176	0.23
57078902		58.440245	0.84	-0.25
55348465		8.2032148	1.088	0.12
53105973		14.944472	1.242	0.31
53305647		10.144533	0.852	-0.23
51771320		4.9814396	0.941	-0.09
39169699		69.786797	171.3	7.42
48278096		9.3220187	0.833	-0.26
55286314		1.2420286	2.09	1.06
53214978		10.935266	0.928	-0.11
52985447		2.9379485	0.768	-0.38

Only

57394768		5.3341714	1.139	0.19
3486841.9		168.80304	0.308	-1.7
52529987		11.898145	1.255	0.33
54019153		19.796298	0.999	0
55997048		4.0688337	1.065	0.09
52030793		7.5494469	1.041	0.06
1448187.2		32.694768	0.021	-5.57
51240407		8.95066	1.249	0.32
52032248		5.9063191	0.759	-0.4
46192970		5.6203985	0.676	-0.56
46950154		14.780086	0.882	-0.18
47054676		17.42199	0.896	-0.16
51928333		17.742991	1.502	0.59
56185215		7.8805299	0.882	-0.18
53264736		3.6149048	0.91	-0.14
38379804		2.2769486	0.563	-0.83
50380782		60.02935	1.54	0.62
53497801		4.3407256	0.85	-0.23
51942014		10.308916	0.825	-0.28
43992475		16.952909	0.649	-0.62
53953721		5.859503	1.019	0.03
13685146		28.197045	0.202	-2.31
36458921		24.281562	0.539	-0.89
45978311		11.66895	0.702	-0.51
45903215		18.348427	1.488	0.57
52232546		8.5671774	0.95	-0.07
52394096		3.3535178	1.127	0.17
51360305		4.6984823	1.145	0.19
46360057		13.980506	0.69	-0.54
31883067		12.401856	0.474	-1.08
46781755		27.533671	0.751	-0.41
48131909		17.904987	2.106	1.07
43882179		11.803559	0.853	-0.23
49776987		9.0907962	0.856	-0.22
52173006		8.5801109	0.99	-0.02
8272144		42.609805	0.123	-3.02
34770073		27.2607	0.519	-0.95
52574468		6.9673562	6.958	2.8
51426410		5.7466959	3.054	1.61
4201613		35.257723	0.063	-3.99
769895.4		67.522932	0.012	-6.43
50779697		14.422993	0.854	-0.23
50085861		6.0443516	23.544	4.56
55092418		6.5388856	1.147	0.2
54483967		1.799654	1.779	0.83
53787470		2.7111354	1.16	0.21
39322779		13.703197	0.595	-0.75
50014196		12.361619	0.817	-0.29
28939664		8.6729344	0.439	-1.19
26777558		97.800161	4.905	2.29

Only

1460744.9		39.464022	0.022	-5.5
54255280		4.3137405	0.992	-0.01
47985631		15.091097	1.017	0.02
30303218		27.440279	0.461	-1.12
44318890		15.491187	0.949	-0.08
52037061		5.1840071	1.375	0.46
42226895		22.236407	0.891	-0.17
40796633		32.51251	1.369	0.45
44800723		11.608563	0.945	-0.08
37876631		22.610342	0.587	-0.77
31696077		30.839476	0.948	-0.08
41818643		36.052187	1.405	0.49
49005025		6.9611264	0.752	-0.41
52272333		11.474833	110.178	6.78
51625607		9.8168229	0.823	-0.28
40017291		27.204991	0.817	-0.29
48420207		19.148272	1.103	0.14
47573680		12.026616	1.079	0.11
39548245		11.995288	0.61	-0.71
31659778		25.875449	0.49	-1.03
48301357		12.166491	1.059	0.08
46052072		11.810215	1.031	0.04
52368205		1.9622229	1.108	0.15
47801485		7.1536419	0.745	-0.43
43700901		60.28305	1.014	0.02
47034057		22.79216	0.842	-0.25
50842335		3.9510209	0.798	-0.33
42224319		15.828736	0.821	-0.28
51498220		9.1813032	0.933	-0.1
49004666		5.6072742	0.894	-0.16
41325946		16.534953	1.106	0.15
50532340		11.049135	0.999	0
51249318		5.9041739	1.116	0.16
49535384		5.7978117	1.32	0.4
22532901		101.86864	0.464	-1.11
24062403		110.28707	0.552	-0.86
52136818		3.5090502	1.088	0.12
49157725		5.8673666	0.85	-0.23
458662.57		63.27622	0.007	-7.1
50002230		6.4648165	1.016	0.02
44974942		26.045598	1.534	0.62
51190379		3.294083	51.512	5.69
201254.35		30.461331	0.003	-8.29
46672280		7.847998	0.954	-0.07
33858817		25.777141	0.774	-0.37
36812632		37.500172	0.629	-0.67
46833366		6.2118043	0.861	-0.22
25258633		19.773729	0.402	-1.31
48161025		4.2210856	1.189	0.25
44987492		12.396422	0.9	-0.15

Only

36193004		26.712605	1.232	0.3
41445300		16.190053	0.907	-0.14
26666230		18.809333	0.429	-1.22
35537289		29.222536	0.573	-0.8
41321760		5.341885	0.667	-0.58
44776049		13.0502	0.911	-0.14
51390257		7.8602706	20.779	4.38
30987489		11.077352	0.501	-1
24335985		13.324531	0.393	-1.35
49105771		6.9518572	0.838	-0.25
46733637		5.0118262	0.955	-0.07
36329628		31.038205	1.112	0.15
20529500		65.573819	0.822	-0.28
49251114		39.691472	1.401	0.49
41008738		21.278185	1.231	0.3
39968070		19.911659	0.802	-0.32
41092387		19.008931	1.553	0.64
46557812		9.7459819	1.258	0.33
26794447		12.165099	0.437	-1.19
47184641		27.531952	2.371	1.25
45585633		7.9431415	1.057	0.08
47481936		6.1118071	0.777	-0.36
32368277		35.333221	0.557	-0.84
18322412		22.865272	0.301	-1.73
41907371		45.084294	0.826	-0.28
45688457		15.753564	1.08	0.11
41730556		59.925841	28.03	4.81
346051.33		29.634122	0.006	-7.46
47260893		6.7351318	26.519	4.73
2784001.8		24.413156	0.046	-4.45
49041364		4.5766327	0.952	-0.07
36228938		22.627315	0.816	-0.29
2225907.8		152.61744	0.037	-4.77
37366091		29.887718	11.167	3.48
41594291		11.10379	0.688	-0.54
41492616		12.844285	1.011	0.02
14602674		44.529928	0.242	-2.05
47339398		10.658153	0.878	-0.19
26078727		39.189848	0.804	-0.31
38931713		6.1312063	0.647	-0.63
47332626		23.614499	1.193	0.25
46452359		9.7397398	1.229	0.3
36324868		41.072522	0.975	-0.04
44942700		14.58864	0.94	-0.09
45858147		7.6264228	0.939	-0.09
1119645.9		184.68675	0.215	-2.22
18068453		41.175408	0.302	-1.73
21586429		113.53347	48.989	5.61
34860759		20.429633	0.734	-0.45
46107485		9.064278	1.114	0.16

Only

44859566		24.672292	1.347	0.43
41915522		66.269732	14.782	3.89
45938952		7.8758418	1.143	0.19
27885995		16.115033	0.468	-1.1
45481498		8.8152813	0.862	-0.21
42291205		12.282738	1.118	0.16
47141023		9.9742573	0.978	-0.03
44106190		4.3931356	0.743	-0.43
4028802.3		22.679577	0.068	-3.88
43971991		40.04225	43.121	5.43
37370641		27.769279	0.96	-0.06
28364497		22.981095	0.479	-1.06
48628244		4.0985088	1.1	0.14
47606221		3.5323095	1.22	0.29
22848295		48.085397	1.359	0.44
26276570		36.057537	0.445	-1.17
41342478		54.862012	1.135	0.18
27686807		48.08223	1.206	0.27
35866144		24.305369	1.004	0.01
45110485		5.0813995	0.942	-0.09
47905547		1.1873426	0.911	-0.14
28453542		46.018978	0.486	-1.04
929121.77		19.570949	0.016	-5.98
38417240		44.309338	0.868	-0.2
44306972		14.159475	3.894	1.96
43108485		9.6088275	0.737	-0.44
45587173		4.7787935	2.519	1.33
35530785		5.6743074	0.608	-0.72
34482624		37.415222	4.191	2.07
37842095		33.62574	1.253	0.33
44840562		13.195173	1.105	0.14
9373790.6		99.321052	0.162	-2.63
41698924		3.5804516	0.721	-0.47
46656811		7.1764936	0.817	-0.29
43872780		17.216021	0.862	-0.21
47043764		2.6542468	1.094	0.13
41426398		66.550395	125.468	6.97
2886402.1		162.52381	0.056	-4.15
46686639		9.1441543	0.814	-0.3
41150130		12.418377	0.988	-0.02
30007697		25.770277	0.522	-0.94
5851704		55.058803	0.102	-3.29
45434885		4.6090013	1.158	0.21
39053286		13.054371	0.843	-0.25
36744410		18.557507	1.216	0.28
35344963		13.364125	0.618	-0.7
976606.26		180.9198	0.017	-5.87
35401744		6.8763927	0.619	-0.69
44716311		7.9779308	0.925	-0.11
45013620		6.0877316	0.926	-0.11

Only

498289.57		33.186741	0.009	-6.84
44983280		4.957975	0.941	-0.09
30521909		19.542789	0.537	-0.9
42546654		19.701787	1.031	0.04
15896418		118.16102	17.099	4.1
40573979		19.026334	0.81	-0.3
39408517		14.164981	1.072	0.1
24648790		20.962939	0.435	-1.2
45233766		12.727481	38.01	5.25
44582035		6.5054865	0.883	-0.18
33681954		26.288045	1.977	0.98
43539129		8.323404	1.045	0.06
43251493		12.340591	0.973	-0.04
39216070		11.357132	0.959	-0.06
31855554		26.792228	1.265	0.34
42720407		9.3991307	1.253	0.33
41006577		13.925812	1.175	0.23
7885070.7		38.76336	0.14	-2.83
45712285		11.86564	1.956	0.97
43690837		12.536003	1.372	0.46
43985735		3.1164333	1.35	0.43
41942330		64.651722	3.015	1.59
43292751		14.087175	1.372	0.46
45454825		2.7962035	1.269	0.34
43607835		8.1105727	0.832	-0.26
45051104		2.3073073	16.742	4.07
34747966		26.906087	0.945	-0.08
42461138		9.4681728	1.112	0.15
44618449		3.927388	1.348	0.43
28222873		36.692104	0.534	-0.91
43839137		3.1296714	0.815	-0.3
40274703		8.6979521	1.33	0.41
44929034		5.290923	0.992	-0.01
44276311		4.2957219	0.912	-0.13
36891293		16.959399	0.963	-0.05
35222667		15.387585	0.976	-0.03
465277.67		7.3039529	0.008	-6.88
44027472		8.2697031	0.834	-0.26
25649524		55.519364	0.467	-1.1
41535584		8.5706836	0.92	-0.12
40347322		3.3310861	0.736	-0.44
43545264		11.295369	1.061	0.09
39801955		15.757698	1.364	0.45
24326747		17.343035	0.445	-1.17
37018614		16.678407	1.382	0.47
44691319		1.6185732	1.261	0.33
28603789		33.497669	0.548	-0.87
38683073		13.461071	3.943	1.98
43297245		6.4698717	0.908	-0.14
37574037		8.4651204	0.692	-0.53

Only

18882532		56.024085	1.484	0.57
41063286		12.893947	0.779	-0.36
37956661		23.439615	1.366	0.45
41380252		6.3594453	1.225	0.29
37938038		19.379172	0.775	-0.37
42644984		8.1967931	0.799	-0.32
32556498		35.766147	1.122	0.17
38501759		65.78076	43.522	5.44
35160061		7.3641376	0.657	-0.61
41697821		3.4934149	0.903	-0.15
39617793		6.6924804	0.741	-0.43
17965802		111.83328	1.484	0.57
42049609		44.824878	1.036	0.05
41767778		2.2526847	0.893	-0.16
38866891		10.911774	1.742	0.8
2305624.4		166.90411	0.345	-1.54
33606956		17.942183	1.582	0.66
29310124		23.606319	0.552	-0.86
35267162		13.74836	0.907	-0.14
38312893		17.919357	1.736	0.8
33674910		19.879557	0.65	-0.62
30693147		67.770487	9.695	3.28
11148500		101.75439	0.336	-1.57
43281106		9.8096097	0.968	-0.05
42876794		2.4847083	0.94	-0.09
32988921		38.441141	0.899	-0.15
39295147		36.643808	2.243	1.17
37341433		10.812503	1.188	0.25
41097208		5.5994339	1.173	0.23
32711460		3.3531562	0.624	-0.68
28658930		44.104587	2.233	1.16
20284295		110.82862	0.515	-0.96
42731732		4.1808274	43.936	5.46
38039985		16.530394	0.728	-0.46
39123415		12.255269	0.993	-0.01
22991696		17.511732	0.44	-1.18
26413121		46.156835	1.644	0.72
39342576		7.100612	1.153	0.21
33394945		19.752827	5.87	2.55
20125258		73.306377	0.693	-0.53
38165476		9.6804689	2.655	1.41
41122329		7.7398436	0.931	-0.1
20311925		70.906864	1.952	0.96
42292287		11.02373	0.873	-0.2
35537123		20.946224	1.307	0.39
40673337		14.627592	1.267	0.34
30352762		6.4843686	0.589	-0.76
39186066		11.922975	1.229	0.3
40088937		7.7968906	0.781	-0.36
36677727		6.1240631	0.716	-0.48

Only

35839376		40.545936	3.704	1.89
35810800		15.997369	1.893	0.92
26733069		31.443372	0.523	-0.93
7961287.7		124.27175	2.759	1.46
37210161		17.338132	2.407	1.27
36032419		33.101265	1.919	0.94
37880034		16.692812	0.865	-0.21
41339108		2.5847375	0.994	-0.01
27908161		31.002998	0.741	-0.43
34462343		10.311614	0.679	-0.56
37147355		6.9850033	1.024	0.03
38577010		21.617074	7.611	2.93
36602957		9.9265146	0.722	-0.47
40623465		3.9684009	0.93	-0.11
39760152		6.4790611	6.925	2.79
32788468		12.806178	0.897	-0.16
39382876		6.482973	1.381	0.47
24964411		35.05904	0.863	-0.21
40943243		2.8201744	1.013	0.02
34725603		8.9217129	0.687	-0.54
30213394		18.240603	0.599	-0.74
1679392.9		36.057169	0.033	-4.91
38668271		7.2449794	0.881	-0.18
1210351.4		7.4215492	0.024	-5.38
30104324		25.215351	0.597	-0.74
34850283		17.174114	1.488	0.57
41221315		6.9841617	1.081	0.11
37906914		5.2793334	0.859	-0.22
38770596		9.7555252	1.311	0.39
37557464		11.374229	0.891	-0.17
17742172		90.930588	57.782	5.85
31278407		24.747153	3.169	1.66
29943147		38.568082	1.066	0.09
40164510		4.4430546	2.821	1.5
33917599		24.380601	0.925	-0.11
39309183		3.8244314	0.874	-0.19
41288374		6.8478061	0.865	-0.21
23073156		12.448595	0.462	-1.12
23073156		12.448595	0.462	-1.12
23073156		12.448595	0.462	-1.12
35369125		15.7511	1.412	0.5
37535706		8.4887667	0.811	-0.3
39232186		7.0972061	1.036	0.05
39273062		6.0773875	0.974	-0.04
32910927		13.98082	0.906	-0.14
37278865		8.8973189	0.836	-0.26
1908377.4		169.52636	0.045	-4.49
39747218		5.5719801	0.933	-0.1
36243254		8.8408311	0.887	-0.17
23007355		49.0016	1.708	0.77

Only

29002148		33.273147	15.354	3.94
30069815		21.890286	0.756	-0.4
34947027		11.992371	1.239	0.31
31118589		24.029229	0.702	-0.51
38729172		6.3315118	1.051	0.07
38308121		20.518933	0.775	-0.37
35517874		19.747215	1.058	0.08
38400275		10.465408	0.89	-0.17
23067219		40.615667	1.624	0.7
11595676		12.966376	0.235	-2.09
39903178		4.6599026	1.086	0.12
19743965		44.742843	3.975	1.99
38930766		11.764815	2.657	1.41
36758689		7.2352119	0.91	-0.14
40197069		12.240479	1.27	0.35
35302230		12.200064	0.825	-0.28
39550046		5.5390094	0.862	-0.21
36914445		5.5206054	1.016	0.02
36289517		14.12638	0.976	-0.03
39336734		4.6370926	0.802	-0.32
14145313		22.22971	0.288	-1.79
30329698		19.229549	1.568	0.65
35526939		12.651462	71.168	6.15
36374167		10.563124	1.068	0.1
1730175.6		10.170214	0.035	-4.82
37046736		5.7515334	0.981	-0.03
37633665		8.533167	1.262	0.34
38192234		57.834814	0.999	0
6167647.2		141.08395	17.238	4.11
22428956		35.841896	1.095	0.13
33519405		11.273228	0.813	-0.3
33407852		27.271415	0.746	-0.42
32906378		21.62642	2.311	1.21
36432260		6.1990886	1.276	0.35
36490656		9.5463544	0.754	-0.41
38612276		5.2186546	0.934	-0.1
25507948		42.327148	30.685	4.94
22553509		7.4573126	0.468	-1.1
35745291		16.065914	24.261	4.6
32045764		38.424642	0.77	-0.38
39016883		3.8817388	1.006	0.01
35463851		11.06697	1.048	0.07
36573479		8.9707631	0.891	-0.17
36881462		6.8819834	0.993	-0.01
30185427		18.108972	0.724	-0.47
35791510		17.508167	0.886	-0.17
27116601		38.207905	36.956	5.21
29158363		35.883098	0.854	-0.23
30565500		19.67831	3.553	1.83
16346790		24.400907	0.342	-1.55

Only

35688669		8.5714787	0.878	-0.19
38979111		8.4894103	0.83	-0.27
16684182		29.248251	0.35	-1.52
34744745		13.502126	0.937	-0.09
38278971		6.2128732	1.247	0.32
34895163		15.78394	32.786	5.04
33288873		21.870023	1.441	0.53
36058218		7.7787942	1.214	0.28
27572246		9.2428773	0.58	-0.79
36012069		14.083656	1.356	0.44
33180595		9.9984033	0.699	-0.52
33180595		9.9984033	0.699	-0.52
34940380		10.419817	1.216	0.28
36871911		7.6891735	1.293	0.37
37259804		4.9771116	0.958	-0.06
32089547		14.559625	0.677	-0.56
38758224		2.5981548	0.842	-0.25
28668592		68.291311	49.005	5.61
35753248		11.934241	1.078	0.11
38562590		5.9634154	1.073	0.1
31291589		19.073288	0.714	-0.49
27525257		6.8334279	0.582	-0.78
20548011		54.491616	1.107	0.15
35257541		14.050253	0.858	-0.22
35852825		23.670689	1.948	0.96
31361819		30.632752	1.549	0.63
37173891		1.8168924	0.789	-0.34
27414066		20.675444	1.482	0.57
30904682		35.978553	1.919	0.94
26633162		24.304642	0.566	-0.82
37179616		4.1406859	26.174	4.71
34198922		9.9926199	0.727	-0.46
35742680		13.766516	1.236	0.31
4095033.9		128.81174	0.087	-3.52
37454790		6.2562129	1.715	0.78
37127992		49.506805	5.315	2.41
37396870		4.8507	5.354	2.42
36283265		9.1980578	1.243	0.31
30185950		18.24766	1.522	0.61
33150163		10.890685	0.904	-0.14
34885413		7.3473447	0.836	-0.26
37669191		5.2240232	1.248	0.32
30668067		6.3243158	0.657	-0.61
38039336		2.3895918	0.954	-0.07
33138099		12.516853	1.349	0.43
33152158		9.7905971	0.712	-0.49
17734940		67.157817	0.974	-0.04
18953992		26.037713	0.408	-1.29
34880542		7.7149726	2.112	1.08
31251620		27.142411	33.924	5.08

Only

33242620		14.324827	0.717	-0.48
34846441		9.959434	1.205	0.27
35468744		11.827123	59.69	5.9
32011955		16.347308	0.976	-0.04
26835036		32.744798	1.114	0.16
34144960		10.325694	0.739	-0.44
35390955		11.124896	1.545	0.63
31915428		17.133439	1.206	0.27
33555205		15.638961	1.116	0.16
31021424		14.984615	1.82	0.86
1097068.8		181.16853	0.315	-1.67
30874492		16.918263	0.883	-0.18
27601668		18.394248	1.471	0.56
29892212		14.047947	0.65	-0.62
32114132		13.206234	0.982	-0.03
37230636		9.6250003	0.867	-0.21
35558385		5.6444743	0.82	-0.29
37795481		3.3962477	1.265	0.34
35202021		17.281237	3.219	1.69
37732617		5.322036	1.267	0.34
32639762		11.317123	1.038	0.05
2957812.5		9.4252502	0.065	-3.94
18512211		3.236226	0.408	-1.29
33356006		10.915302	0.977	-0.03
33463126		7.865939	0.864	-0.21
404065.07		26.990083	0.009	-6.81
37177001		2.6762169	123.678	6.95
36901911		4.15997	0.839	-0.25
21746826		13.561418	0.482	-1.05
34657654		5.9722042	1.198	0.26
34145809		13.608598	0.927	-0.11
607935.88		176.36838	0.013	-6.21
31415628		22.684527	0.794	-0.33
32901534		58.929537	1.043	0.06
31620903		24.306727	1.015	0.02
35737417		3.2085885	1.003	0
27663562		19.080401	0.97	-0.04
20687863		4.0209874	0.461	-1.12
34891030		24.659361	1.041	0.06
34481293		7.5645427	1.336	0.42
36922184		4.5398773	1.111	0.15
36553432		3.2865423	1.063	0.09
34734276		8.762509	0.916	-0.13
4862956.2		113.62045	0.109	-3.19
30619880		14.201526	0.839	-0.25
32952854		19.181681	0.741	-0.43
31588657		15.166596	3.429	1.78
26215403		16.934016	0.59	-0.76
30158885		19.876495	0.994	-0.01
33531446		60.4306	30.89	4.95

Only

30505560		23.631151	0.98	-0.03
32356440		9.3177192	2.399	1.26
26863921		23.778977	0.607	-0.72
32353392		6.882147	0.731	-0.45
34657604		13.1215	0.97	-0.04
30155061		14.645233	1.319	0.4
18335947		113.1561	0.471	-1.08
36216165		1.5253507	0.845	-0.24
29570208		6.5702585	0.672	-0.57
14687865		31.99365	0.334	-1.58
26406706		19.374807	1.178	0.24
36152043		4.1307801	0.844	-0.25
8876122.2		92.026152	0.253	-1.98
33803309		9.9118438	1.667	0.74
32911905		21.792641	0.827	-0.27
33207602		8.1233302	0.771	-0.38
18132935		113.69984	0.514	-0.96
34808948		4.2297035	1.017	0.02
32522773		11.350992	0.746	-0.42
32522773		11.350992	0.746	-0.42
34041633		5.0103932	0.904	-0.15
31690842		16.969988	0.905	-0.14
28628965		37.186813	0.954	-0.07
27978865		17.003777	0.761	-0.39
32669977		6.5009012	0.753	-0.41
15842016		28.215755	0.366	-1.45
35842727		3.0330885	0.95	-0.07
27176990		43.21749	0.891	-0.17
6945319.8		129.46006	7.671	2.94
32483232		15.654557	0.794	-0.33
33956355		6.6864977	36.244	5.18
33296341		4.0721534	0.879	-0.19
21686404		44.93413	1.425	0.51
23896695		24.529485	0.613	-0.7
30806683		26.848936	2.332	1.22
31388574		18.940023	0.729	-0.46
34705257		15.165919	1.004	0.01
5507860.8		7.5633822	0.128	-2.96
28301326		26.91816	4.392	2.14
33010031		4.7005125	0.947	-0.08
31289522		13.954036	0.729	-0.46
6898356.8		32.093431	0.161	-2.64
31913798		12.739073	1.564	0.65
35034892		10.863621	1.404	0.49
34956450		8.6598357	0.819	-0.29
31753243		48.755513	0.973	-0.04
23767901		30.098562	0.748	-0.42
29872364		12.39769	0.733	-0.45
35006986		4.7099675	3.075	1.62
32107496		5.3302429	1.102	0.14

Only

33160801		20.139453	1.246	0.32
33904047		10.980837	0.899	-0.15
32954710		9.4186201	0.985	-0.02
29396995		7.7424493	0.69	-0.53
1892685.1		72.019138	0.044	-4.49
26451846		20.553338	1.342	0.42
31583401		13.688957	2.537	1.34
26773995		7.1354904	0.63	-0.67
31307081		24.87855	2.042	1.03
25589958		32.544302	1.363	0.45
30002128		13.111619	1.634	0.71
33850628		4.3222886	1.061	0.09
33335592		13.485266	1.39	0.47
30097653		17.724945	10.265	3.36
31670264		7.9279323	0.782	-0.35
21702617		11.171234	0.513	-0.96
25437002		25.096628	4.5	2.17
11635467		74.355715	2.432	1.28
33895192		9.5665109	1.041	0.06
15131393		49.618124	0.359	-1.48
29496101		18.593925	0.876	-0.19
28835771		3.8580631	0.686	-0.54
19594617		70.470059	2.406	1.27
23690424		24.698836	3.079	1.62
34137780		3.157704	2.64	1.4
32208088		11.17529	0.985	-0.02
4334680.1		140.39901	2.988	1.58
33013160		5.9408061	0.963	-0.05
21908374		11.906743	0.524	-0.93
30611755		9.0449045	1.704	0.77
29877794		11.705179	1.453	0.54
33537733		8.2419698	1.14	0.19
29978813		10.286204	0.783	-0.35
31433008		8.7670876	1.297	0.38
31440772		9.4228787	0.999	0
30452325		12.92614	0.993	-0.01
33537733		8.1782869	1.14	0.19
30633991		7.6644361	16.452	4.04
23635528		25.634911	48.311	5.59
24452630		22.695458	0.588	-0.77
29722704		11.807794	0.715	-0.48
16057814		60.333415	0.397	-1.33
23094238		38.556167	0.942	-0.09
31893226		6.6048968	0.885	-0.18
32623840		5.9318589	0.854	-0.23
6307260.9		131.60965	2.552	1.35
31337620		15.387244	2.077	1.05
19786701		5.5562795	0.479	-1.06
19178252		36.774212	1.357	0.44
18040282		3.7197486	0.437	-1.2

Only

33804194		2.5328754	0.836	-0.26
17144745		16.469897	0.416	-1.27
28511959		12.190581	7.681	2.94
27488696		14.406745	1.365	0.45
32479212		5.2982439	1.075	0.1
31602891		7.4129988	0.916	-0.13
27160544		21.747396	1.32	0.4
16700865		5.1315016	0.407	-1.3
30635429		6.9849367	1.149	0.2
1848876.2		5.8656018	0.045	-4.47
19832656		43.801231	0.591	-0.76
27499277		13.544081	0.818	-0.29
30190874		13.165164	1.435	0.52
23086277		27.336514	0.787	-0.35
30102662		10.574398	0.911	-0.13
25976344		27.455685	0.863	-0.21
25231710		23.085368	1.065	0.09
31437429		10.908925	1.11	0.15
32988808		5.2836556	1.101	0.14
30986987		7.0998316	0.963	-0.05
30205339		13.889867	0.815	-0.3
27184628		13.233223	0.781	-0.36
25688378		25.976202	0.796	-0.33
22237126		24.555114	0.982	-0.03
30350437		7.5395434	0.853	-0.23
27867034		15.005118	0.889	-0.17
30734152		14.959968	0.976	-0.03
31985297		14.461686	1.217	0.28
29707367		24.674069	0.994	-0.01
29067892		5.3939324	0.722	-0.47
24651000		16.805527	0.815	-0.29
31757638		7.2972223	0.801	-0.32
25841338		16.615725	1.357	0.44
28407896		12.797876	0.937	-0.09
21994090		31.893918	1.09	0.12
30185641		11.429444	1.07	0.1
16012988		16.225149	0.399	-1.32
22525252		31.245924	0.849	-0.24
24960644		20.144467	4.712	2.24
32337210		4.3376301	0.887	-0.17
32780385		10.988153	1.397	0.48
30765900		10.054291	2.186	1.13
23096334		39.730187	1	0
23421485		29.884739	1.282	0.36
29040482		8.569296	0.728	-0.46
30425907		11.574881	2.779	1.47
30639385		8.7285273	1.13	0.18
27821804		13.335634	1.182	0.24
2338119		15.124298	0.059	-4.09
29025500		13.206073	1.016	0.02

Only

31116107		5.9641428	0.796	-0.33
29508473		14.513509	15.436	3.95
28311317		18.410574	0.894	-0.16
29008640		55.503805	0.732	-0.45
22972858		32.21196	1.074	0.1
16433648		49.768249	0.415	-1.27
27550069		53.863681	0.845	-0.24
28727322		11.192417	1.003	0
30412949		7.6385034	0.976	-0.04
30648418		11.09762	0.976	-0.03
28838359		60.388603	1.61	0.69
22536085		28.076668	0.573	-0.8
29593752		9.4213539	1.114	0.16
30676622		4.9683483	44.637	5.48
32625201		2.406531	1.415	0.5
30346590		11.626369	1.039	0.06
28539318		10.149946	1.32	0.4
29598759		12.205423	1.3	0.38
20178433		20.775515	0.515	-0.96
32013019		4.6660851	1.245	0.32
31394507		9.1202953	0.802	-0.32
25940716		17.684872	1.287	0.36
19783153		37.871774	0.811	-0.3
29578592		11.002771	0.923	-0.12
23508053		36.341075	0.91	-0.14
25286757		13.286704	28.139	4.81
25265035		6.2275925	0.65	-0.62
29696742		7.2337952	0.962	-0.06
30811342		5.2149598	0.802	-0.32
26953169		24.713895	0.948	-0.08
24215996		6.5422897	0.625	-0.68
29517031		9.4314293	0.762	-0.39
28970196		7.3947617	0.811	-0.3
27913062		13.839016	1.872	0.9
27037892		9.40698	1.13	0.18
18478337		50.11563	0.596	-0.75
27696142		12.081221	0.762	-0.39
31839465		6.0767437	0.939	-0.09
22915567		24.741456	1.111	0.15
26189740		13.428208	0.952	-0.07
979527.73		6.0724513	0.025	-5.3
31248303		2.4035991	0.98	-0.03
30984934		4.3941937	0.861	-0.22
30276880		10.025348	0.827	-0.27
27418610		16.496725	1.444	0.53
25272683		22.034267	1.214	0.28
26249238		18.603023	0.834	-0.26
31309613		5.7923165	0.99	-0.02
26100889		24.876669	0.876	-0.19
27816472		18.759141	1.33	0.41

Only

22026086		46.470535	21.469	4.42
9904252.3		7.8462242	0.258	-1.95
30295834		10.697828	2.682	1.42
24536142		15.489665	1.34	0.42
31021651		18.499639	1.942	0.96
28956591		6.5923631	0.826	-0.28
27874182		19.776285	0.729	-0.46
10848534		36.982796	0.284	-1.82
19386644		10.72146	0.508	-0.98
491954.88		28.097299	0.013	-6.28
16844259		43.378508	1.811	0.86
27779267		14.033436	2.971	1.57
29253950		13.468767	0.843	-0.25
27065328		14.241381	0.898	-0.16
28525283		9.0116251	1.217	0.28
926945.35		178.53308	0.939	-0.09
29972343		4.4444885	1.189	0.25
25371219		68.120632	82.118	6.36
29148812		9.0403333	1.576	0.66
30341266		11.464006	1.016	0.02
22165974		17.322436	0.585	-0.77
12225839		18.118063	0.323	-1.63
28167579		8.8025194	22.03	4.46
3518092.5		104.57795	0.093	-3.43
30606279		13.206804	0.9	-0.15
29474998		5.1199028	0.83	-0.27
25647057		13.497603	1.157	0.21
25919059		20.345808	0.856	-0.22
30677608		3.3374006	1.274	0.35
28918252		14.995676	1.206	0.27
29719696		7.2518316	0.921	-0.12
23727730		14.611722	0.634	-0.66
15975007		46.871057	0.635	-0.66
26081667		13.549327	1.21	0.27
22258373		52.034599	1.095	0.13
24501269		24.332139	0.733	-0.45
26747803		17.325674	0.728	-0.46
30039295		3.783092	0.953	-0.07
27577937		7.471152	1.149	0.2
21548104		53.535854	0.823	-0.28
27448761		13.158499	1.067	0.09
26196229		14.00275	1.423	0.51
28383455		11.252021	43.368	5.44
26755236		14.235224	0.722	-0.47
23429783		5.1316652	0.633	-0.66
25519230		13.519995	0.69	-0.54
22383700		37.53155	1.367	0.45
4091776.8		26.029844	0.111	-3.17
12032259		22.781918	0.326	-1.62
16615228		30.421911	0.45	-1.15

Only

28384330		6.0726983	1.468	0.55
27647285		27.375871	0.941	-0.09
28724311		11.553641	0.78	-0.36
19405480		26.711299	1.311	0.39
27122364		11.272249	0.827	-0.27
13288436		10.238072	0.361	-1.47
29528713		1.0472997	2.804	1.49
27545529		9.6922017	0.873	-0.2
20219854		15.555399	0.551	-0.86
17742487		32.68087	1.562	0.64
19117485		33.270521	0.891	-0.17
23206757		24.703167	2.5	1.32
28147731		5.5648472	0.966	-0.05
25055191		15.933464	1.096	0.13
28319325		4.8272899	0.773	-0.37
24123537		12.918579	0.878	-0.19
21284820		5.374644	0.582	-0.78
4288649		125.29376	0.137	-2.87
24539241		16.047122	18.843	4.24
29898828		4.7749932	0.822	-0.28
23529823		13.077899	0.812	-0.3
20486783		24.357995	1.081	0.11
25695781		8.7936503	0.853	-0.23
17188807		41.45183	1.629	0.7
28600049		7.6268869	0.88	-0.18
13837227		5.5784461	0.38	-1.4
22262922		17.205687	0.887	-0.17
14328849		47.644762	7.446	2.9
27999782		9.3482107	0.972	-0.04
26280464		8.2135935	3.988	2
26670939		14.42536	0.908	-0.14
27737551		8.0741383	0.825	-0.28
24346958		20.900193	0.956	-0.06
27411361		7.1974187	2.47	1.3
24372673		63.584394	7.855	2.97
22553853		35.869398	0.623	-0.68
28742185		14.346325	1.08	0.11
15938072		99.048402	7.721	2.95
11100113		90.254784	6.225	2.64
24725264		11.997691	1.414	0.5
19245781		26.853628	0.838	-0.26
22637394		16.058313	0.863	-0.21
29680271		5.776385	0.89	-0.17
29203566		4.0052265	2.157	1.11
25155329		28.883743	0.698	-0.52
23934044		16.50589	0.999	0
7294412.6		50.721952	0.202	-2.3
17653043		16.926133	0.49	-1.03
23622241		18.160101	1.217	0.28
24166457		13.345018	0.672	-0.57

Only

25455388		14.088518	0.78	-0.36
22019363		43.532328	26.759	4.74
17009150		36.429099	0.569	-0.81
22555704		20.051979	0.995	-0.01
26557161		9.3256229	1.048	0.07
3287312.8		143.63437	2.064	1.05
28627457		9.2534634	0.946	-0.08
26025033		41.710304	0.726	-0.46
27701958		8.1720942	0.945	-0.08
27747921		10.883828	1.055	0.08
26968814		9.2777116	1.206	0.27
20672077		37.145219	1.269	0.34
26794723		10.233742	0.988	-0.02
22754721		23.355726	0.731	-0.45
23238455		18.851241	11.386	3.51
8956774		14.023934	0.251	-2
29238879		21.702656	1.146	0.2
28137396		5.2042419	1.319	0.4
27982517		7.3098696	1.08	0.11
25214556		12.423565	1.088	0.12
20922331		19.482389	0.589	-0.76
27740271		5.5698249	0.941	-0.09
27458172		4.536451	0.969	-0.05
20465326		21.612224	1.433	0.52
27866433		10.346519	1.504	0.59
21196331		19.090877	0.598	-0.74
18174229		23.584988	0.513	-0.96
24451353		14.323626	0.69	-0.53
19737855		26.906055	19.697	4.3
28199527		5.9128178	0.96	-0.06
26361761		8.0991528	1.278	0.35
28949632		2.8829843	0.919	-0.12
24469633		15.81341	0.693	-0.53
24160545		20.174409	1.172	0.23
16388592		25.492392	0.464	-1.11
26380994		14.612524	0.933	-0.1
26774529		9.9448692	0.759	-0.4
28165628		4.2696577	2.749	1.46
28108629		1.5707061	1.178	0.24
23656434		13.901131	0.731	-0.45
26173629		12.59562	0.846	-0.24
15831396		26.289898	0.45	-1.15
21592052		28.968994	0.638	-0.65
18553714		29.68942	1.043	0.06
22437773		17.56328	1.456	0.54
26857938		13.967735	1.067	0.09
25384204		23.387803	1.228	0.3
28497502		5.7624877	1.662	0.73
25539165		15.42577	1.135	0.18
22966930		43.96557	0.779	-0.36

Only

26081167		10.390969	1.045	0.06
11972255		7.7410284	0.341	-1.55
28871673		2.9054887	0.856	-0.22
26986677		9.9343489	1.197	0.26
23828752		16.030024	9.891	3.31
25507227		12.405285	6.665	2.74
773723.42		25.865233	0.022	-5.49
28457143		11.175826	1.094	0.13
27116821		6.4516498	1.163	0.22
25998562		6.5549723	1.153	0.21
28381698		3.3430035	1.316	0.4
27194713		6.3025212	1.136	0.18
24014117		14.618123	0.811	-0.3
18879308		23.833217	0.545	-0.88
16300110		18.99787	0.472	-1.08
25520666		6.5993061	0.965	-0.05
16836830		9.5145133	0.488	-1.03
20672499		47.947406	0.958	-0.06
25001790		55.117732	1.149	0.2
23187401		40.017954	1.122	0.17
22862097		18.909111	0.928	-0.11
24958534		10.334926	1.221	0.29
24958534		11.627897	1.221	0.29
22000429		16.406353	0.878	-0.19
27980157		6.7789142	4.82	2.27
382554.43		16.365298	0.011	-6.49
24251434		18.774775	0.921	-0.12
8347899.6		107.02283	0.809	-0.31
26460490		6.7803218	0.847	-0.24
26008738		6.5592265	0.832	-0.27
25336614		10.726374	0.914	-0.13
27966219		4.5642545	1.168	0.22
27651407		4.5204188	1.242	0.31
27338597		7.1490275	1.174	0.23
25642141		12.606334	0.987	-0.02
22104072		21.828346	0.92	-0.12
26797827		11.002825	0.943	-0.09
25089675		16.117125	1.87	0.9
23658781		13.728273	2.681	1.42
27249934		4.9394218	0.872	-0.2
26275744		8.9799925	1.148	0.2
25639983		7.2400068	1.04	0.06
24281088		7.1802845	1.026	0.04
26077962		10.224959	1.478	0.56
25418840		29.854544	0.918	-0.12
26455864		5.8036425	1.201	0.26
25837378		9.7455417	1.649	0.72
27012527		18.400711	1.261	0.34
26765688		6.1660335	2.087	1.06
23222708		21.974536	1.11	0.15

Only

14280848		26.522387	0.426	-1.23
26543706		6.5547561	1.861	0.9
19896631		31.737453	0.74	-0.43
22635399		9.6227668	0.677	-0.56
22896928		16.893996	1.37	0.45
24454754		24.888957	1.319	0.4
26236461		6.6088068	0.85	-0.23
27268919		4.3511523	1.011	0.02
5884165.6		61.5731	0.177	-2.5
225267.27		29.68779	0.007	-7.21
26572448		60.054199	1.11	0.15
25665168		12.551648	0.84	-0.25
9893376.9		4.1628282	0.298	-1.75
9363192.6		90.475307	3.268	1.71
23871812		52.800602	0.924	-0.11
19967763		64.292631	1.155	0.21
26282392		8.2371253	1.014	0.02
25765798		8.9994546	10.703	3.42
19420968		24.494827	0.709	-0.5
26804375		3.3153684	0.985	-0.02
22806900		9.7646251	1.169	0.22
25336083		8.5570954	1.068	0.09
10769104		45.890835	0.328	-1.61
843390.62		6.7491426	0.026	-5.28
22999174		8.2680415	0.7	-0.51
25251932		58.684596	0.817	-0.29
21347715		15.742659	1.105	0.14
22262292		18.86439	11.924	3.58
23535171		10.736125	7.997	3
23659489		29.590458	1.096	0.13
25153553		8.2625253	14.985	3.91
26027254		4.7035984	0.955	-0.07
23973438		12.47929	0.734	-0.45
11104095		16.858105	0.34	-1.56
13928263		52.589369	0.921	-0.12
20831736		6.645128	0.639	-0.65
19211949		67.044834	0.806	-0.31
24428951		23.572245	0.75	-0.42
23644264		7.7515138	1.029	0.04
19912640		28.920324	2.031	1.02
22230294		18.182457	0.746	-0.42
26597763		12.574259	1.11	0.15
24637718		12.373465	1.591	0.67
20947953		19.594485	1.786	0.84
1694821.1		73.019998	0.052	-4.26
22018965		18.162483	0.803	-0.32
1924683.8		16.038062	0.059	-4.07
22074909		15.271115	1.354	0.44
21155837		14.090129	1.281	0.36
21057931		44.735555	1.308	0.39

22896741		8.8562349	5.843	2.55
11056782		39.861747	0.343	-1.54
24307865		31.982833	5.169	2.37
23429553		9.175194	0.919	-0.12
23789164		11.825334	0.872	-0.2
25911982		6.0895101	1.063	0.09
25911982		6.0895101	0.919	-0.12
15148496		2.6741375	0.472	-1.08
18552446		20.918322	1.04	0.06
20402251		26.401723	0.651	-0.62
21931246		13.194168	0.686	-0.54
22345510		14.748411	1.426	0.51
16896599		33.362765	2.131	1.09
21463709		28.70091	0.975	-0.04
21271079		22.48204	0.978	-0.03
25533777		6.465517	0.908	-0.14
21770778		8.4023426	0.683	-0.55
17260788		37.456985	3.71	1.89
14983289		16.811343	0.471	-1.09
2882926		126.2227	0.091	-3.46
25438785		13.323701	0.948	-0.08
23628855		20.912454	77.941	6.28
20507293		13.937799	1.018	0.03
21998792		51.475581	0.959	-0.06
23576300		12.212171	0.933	-0.1
18661089		20.040642	0.919	-0.12
26055877		3.5732992	1	0
23844152		18.099992	3.065	1.62
22863960		7.3463163	0.92	-0.12
23757288		9.3337206	1.234	0.3
18410200		16.727223	0.582	-0.78
23698574		62.218122	13.184	3.72
25640577		2.3914914	21.922	4.45
15721037		37.520895	1.331	0.41
20337003		20.401211	0.893	-0.16
23886678		7.3662434	9.192	3.2
25271633		16.213378	0.801	-0.32
22742080		7.8883605	1.366	0.45
23863986		5.1752144	1.127	0.17
25589146		1.9779116	1.14	0.19
660595.52		23.286431	0.021	-5.57
14679200		38.074418	0.496	-1.01
24535805		12.620553	3.046	1.61
20169826		43.672725	0.902	-0.15
23043220		63.033839	41.668	5.38
5045065.3		8.935424	0.161	-2.64
21293024		10.584418	1.167	0.22
7106138.4		48.761669	0.227	-2.14
22834399		22.776329	0.881	-0.18
17446110		27.014217	1.121	0.16

Only

21081205		20.481377	1.401	0.49
14846237		14.418062	0.476	-1.07
10283619		90.036311	1.496	0.58
3001767.3		138.12378	1.604	0.68
25539837		6.4632647	1.048	0.07
25592615		5.3506204	1.128	0.17
16243021		31.980698	0.522	-0.94
1812445.5		157.5052	2.061	1.04
21640735		19.959599	0.776	-0.36
671484.99		4.7675843	0.022	-5.53
20905002		11.319833	0.674	-0.57
18154628		19.83484	1.007	0.01
22296977		12.43445	7.539	2.91
18133549		25.377219	1.282	0.36
25068846		6.6062164	0.858	-0.22
23525510		6.7248454	0.927	-0.11
1097727.4		172.5001	0.416	-1.26
23470118		11.800562	8.442	3.08
21540094		24.423561	0.697	-0.52
20406520		14.270398	1.284	0.36
16649142		30.075169	0.539	-0.89
15028743		13.003418	0.488	-1.04
23395174		11.05936	1.028	0.04
16602126		34.725388	2.291	1.2
20399387		14.091534	0.758	-0.4
21578013		25.931431	1.09	0.12
20654000		37.50654	16.527	4.05
17990442		23.234466	0.596	-0.75
23586062		13.744687	1.278	0.35
24293863		8.7571791	2.553	1.35
21644956		15.071309	0.812	-0.3
23416482		13.968051	1.132	0.18
22299113		15.190911	3.739	1.9
12055035		112.96332	0.932	-0.1
15940962		39.964829	17.589	4.14
24403748		5.2869825	0.815	-0.3
16212366		45.398141	24.298	4.6
17191936		22.193732	16.484	4.04
23523435		2.9602443	0.893	-0.16
494871.42		14.432411	0.016	-5.94
21551308		7.8355049	0.893	-0.16
25211890		6.1406988	0.87	-0.2
23930044		6.8133744	0.803	-0.32
20523918		17.585316	1.062	0.09
21982312		13.590554	8.166	3.03
15255473		37.02311	0.917	-0.12
12804607		38.518155	1.103	0.14
22605182		8.6499757	1.11	0.15
20651438		12.888663	0.807	-0.31
22769565		14.091947	1.016	0.02

22598821		15.029471	2.73	1.45
24241170		7.5972256	1.099	0.14
24057895		5.9070044	0.858	-0.22
24431958		3.4861503	0.934	-0.1
21646782		11.422267	1.804	0.85
22371442		14.433443	1.1	0.14
20410108		16.685763	1.374	0.46
16312377		28.664593	0.544	-0.88
23432313		10.388269	1.103	0.14
22567007		10.775255	0.955	-0.07
22158933		16.150608	1.486	0.57
23245301		10.432996	1.04	0.06
1897326.6		151.37296	0.678	-0.56
22375806		16.124792	1.102	0.14
21224827		8.8737164	0.709	-0.5
24149581		9.7713051	0.833	-0.26
22678115		11.278626	1.241	0.31
17253268		30.342663	1.076	0.11
23082375		6.4531775	1.779	0.83
24480479		9.4573514	1.057	0.08
23476396		6.8135135	0.96	-0.06
23305778		4.7388195	0.902	-0.15
23028114		23.384949	0.796	-0.33
21476320		30.475814	1.849	0.89
20001704		32.965828	22.641	4.5
19273149		15.80722	1.696	0.76
24646290		5.4754465	0.867	-0.21
21256158		7.9274839	1.083	0.12
20647743		19.234408	1.218	0.29
22603168		4.8132446	8.727	3.13
23255669		14.988246	19.612	4.29
4478017.9		7.6282329	0.151	-2.73
24072542		5.6891576	0.935	-0.1
21368101		11.904426	1.015	0.02
22769713		11.333155	1.048	0.07
23158068		19.054236	1.207	0.27
17847902		27.133112	0.634	-0.66
22687532		14.349809	25.546	4.68
23217583		17.492829	1.087	0.12
21843585		11.293511	0.972	-0.04
16508906		36.46302	0.982	-0.03
23641936		9.3316159	1.105	0.14
14926842		14.998862	0.505	-0.99
23671507		6.6594362	0.914	-0.13
23883411		3.5589429	0.899	-0.15
20854072		18.631916	0.831	-0.27
22726717		5.5988059	0.77	-0.38
20132851		25.190298	1.641	0.71
16744647		30.304085	15.357	3.94
23759022		2.8197713	0.83	-0.27

Only

16261470		22.094664	0.695	-0.53
22258250		4.7374118	0.758	-0.4
1636182.4		52.021487	0.056	-4.17
22986829		7.2529529	1.429	0.52
14588718		11.909655	0.498	-1.01
18416023		25.284452	0.979	-0.03
13159284		11.135058	0.45	-1.15
20873301		32.308568	0.85	-0.23
7533635.4		22.495352	0.258	-1.96
16281352		29.515362	1.012	0.02
22518989		8.305673	0.972	-0.04
19258759		18.471798	1.049	0.07
17472590		19.611647	1.443	0.53
23944298		2.2385529	1.523	0.61
22832489		15.173381	1.914	0.94
23739545		4.2925276	12.03	3.59
22606915		7.0870678	1.168	0.22
22777174		9.6221885	1.589	0.67
1237481.5		19.854306	0.043	-4.55
20842229		28.693768	16.241	4.02
21473885		10.948008	1.131	0.18
21015573		8.4321204	0.93	-0.11
18316354		24.69449	0.883	-0.18
21456411		8.4404294	1.061	0.09
20673567		19.646049	0.952	-0.07
21967554		5.6580369	0.909	-0.14
20182955		11.050814	1.275	0.35
23781684		5.7954343	1.047	0.07
19700217		17.619861	0.833	-0.26
18177539		18.85343	1.249	0.32
19671074		13.570416	3.847	1.94
17746415		24.176615	0.951	-0.07
22976350		3.7696519	1.054	0.08
15415196		30.335734	0.612	-0.71
667601.4		51.479994	0.023	-5.44
23299638		4.4579933	1.459	0.54
21574696		8.2905817	0.929	-0.11
14473692		35.519929	1.855	0.89
22956373		4.9199212	1.543	0.63
17433405		41.637418	10.376	3.38
23044458		5.5995272	0.825	-0.28
12692390		25.699756	0.44	-1.18
20492132		14.637123	1.122	0.17
23149272		3.9979522	1.189	0.25
22373040		4.5815662	1.325	0.41
21624488		5.7072541	0.864	-0.21
4737303.9		3.9457315	0.165	-2.6
20363055		8.2836081	0.999	0
23513497		3.9348555	1.051	0.07
10587112		64.937134	0.988	-0.02

Only

14479875		64.447361	4.896	2.29
17649804		35.715104	0.722	-0.47
14565337		35.057739	1.149	0.2
19017239		12.677689	0.793	-0.34
17762882		29.185623	0.621	-0.69
21103613		40.031725	1.651	0.72
23650953		5.9953893	0.918	-0.12
20676334		9.9046957	1.219	0.29
21494566		5.997214	0.882	-0.18
21438555		12.533177	17.192	4.1
12803573		12.492713	0.449	-1.16
20560314		15.942449	1.017	0.02
23631845		4.0146124	0.897	-0.16
18772444		50.989658	0.659	-0.6
19284247		24.571784	0.877	-0.19
1249270.1		23.915865	0.044	-4.51
16045554		36.220948	1.065	0.09
21119325		45.059512	0.929	-0.11
22419842		4.8265561	0.93	-0.1
15170228		16.744383	0.534	-0.9
19210938		17.379827	8.452	3.08
22885885		6.8495239	1.338	0.42
1835114.8		82.598936	0.065	-3.95
15748860		11.694835	0.556	-0.85
21113771		6.6675858	0.888	-0.17
20208383		16.524171	0.736	-0.44
1134820.4		168.8811	1.088	0.12
13739899		14.419549	0.486	-1.04
19148934		16.125664	0.972	-0.04
21105682		11.491062	1.059	0.08
18471867		13.317625	1.059	0.08
21520461		4.5441046	0.988	-0.02
18345984		15.978792	3.855	1.95
18646043		46.410063	1.042	0.06
13918018		83.46738	9.095	3.19
21407608		13.031781	1.57	0.65
21140652		7.654151	0.87	-0.2
20049384		17.373543	1.586	0.67
18850931		39.159583	0.975	-0.04
22332088		4.5285538	0.973	-0.04
2931908.5		13.424223	0.105	-3.25
21657660		66.115077	69.603	6.12
13553018		3.9921737	0.487	-1.04
11943901		14.848401	0.429	-1.22
22894121		7.0815562	0.96	-0.06
17611750		18.629121	0.715	-0.48
14400267		21.201011	0.52	-0.94
21153028		11.484316	0.862	-0.21
14731353		27.629679	0.807	-0.31
16519732		17.733068	0.762	-0.39

Only

22733479		2.3294995	0.891	-0.17
17091727		26.331293	0.85	-0.23
16110784		27.67114	1.931	0.95
19666481		6.8468	0.713	-0.49
17077977		25.518758	0.924	-0.11
22342503		4.9899782	1.946	0.96
21298884		6.6218344	43.972	5.46
18714543		25.51893	0.84	-0.25
18581815		30.23106	0.895	-0.16
17350423		17.849089	1.904	0.93
18973581		13.97007	5.645	2.5
20630249		10.589582	14.335	3.84
19978820		21.779607	1.117	0.16
17360291		25.576924	1.542	0.63
13973445		12.010388	0.511	-0.97
16938310		28.566216	5.807	2.54
21456463		5.344956	0.936	-0.1
16744779		17.869894	4.335	2.12
11910760		102.07797	10.475	3.39
13440938		34.918147	0.816	-0.29
21014468		3.4537926	1.135	0.18
20146412		8.3385993	0.926	-0.11
14865463		26.402049	0.546	-0.87
20921319		7.9302449	1.064	0.09
9738852.2		55.135066	0.845	-0.24
20838504		24.235478	1.219	0.29
22713028		7.2334365	2.434	1.28
20916535		6.2238911	2.204	1.14
20789055		9.2425429	0.911	-0.13
20969210		10.695694	3.662	1.87
10896799		16.360745	0.402	-1.31
19421947		5.163412	0.718	-0.48
21504432		1.6026492	0.954	-0.07
17732470		14.999879	0.866	-0.21
20719114		13.658644	1.306	0.38
21659982		6.6159645	12.93	3.69
19219984		12.466156	1.32	0.4
21168647		10.172573	1.487	0.57
7485738.1		30.48171	0.278	-1.85
17210780		16.780196	1.375	0.46
21219689		31.577922	0.977	-0.03
18315909		24.956919	1.674	0.74
18421159		12.819647	1.151	0.2
21601281		6.8742294	1.088	0.12
18026924		18.31218	1.853	0.89
16026312		55.659626	2.008	1.01
16276464		65.106264	0.714	-0.49
19337553		11.935596	7.596	2.93
17646952		25.968025	0.975	-0.04
17172166		15.556972	0.857	-0.22

Only

12785687		34.073428	0.83	-0.27
19796201		13.765726	0.765	-0.39
22134334		6.4868966	1.524	0.61
16498073		15.592986	0.859	-0.22
14135027		27.870901	0.531	-0.91
283169.85		191.15906	0.031	-5.01
4142741.3		13.772031	0.156	-2.68
15638757		28.855626	0.96	-0.06
15002499		13.92402	0.567	-0.82
21895393		5.2898051	0.971	-0.04
20836161		7.6979241	1.569	0.65
19893527		7.6217589	1.26	0.33
18368634		44.469028	0.809	-0.31
6531594.7		117.44984	2.073	1.05
19299780		25.236084	0.981	-0.03
8774946.3		109.73325	1.181	0.24
12128252		48.495515	1.983	0.99
20510740		7.0346023	1.145	0.19
20464546		7.786982	12.04	3.59
1214049.5		23.430866	0.046	-4.44
19657843		10.667428	0.905	-0.14
17384914		12.679193	1.189	0.25
20951154		8.977396	1.094	0.13
16876495		12.824305	22.655	4.5
10070913		31.396653	0.383	-1.38
21245949		4.7664834	0.824	-0.28
18638260		13.705119	1.204	0.27
15612334		20.701864	0.596	-0.75
15082069		48.287409	1.999	1
19544354		66.818624	67.572	6.08
19375616		11.282073	0.74	-0.43
12325642		53.503571	0.527	-0.92
21321298		6.3741946	0.938	-0.09
20881897		8.1593447	1.162	0.22
19827751		5.9714587	1.452	0.54
18579477		10.379052	7.791	2.96
16684754		31.457685	1.144	0.19
17993113		22.945708	1.634	0.71
20525345		10.69232	1.207	0.27
12571647		12.931198	0.482	-1.05
21099730		7.9137283	1.274	0.35
21153037		5.5364371	1.443	0.53
20489666		13.675726	1.223	0.29
17136664		14.11389	0.658	-0.6
5287968.7		102.37687	0.366	-1.45
19892595		12.774702	4.02	2.01
17785850		4.4038792	0.684	-0.55
16695880		16.435946	1.653	0.73
19388767		3.7555183	0.747	-0.42
15077463		33.450592	14.38	3.85

Only

17517950		18.136204	0.676	-0.56
19042610		14.946706	0.824	-0.28
15269986		22.40263	2.848	1.51
14393933		27.456015	0.557	-0.84
11308368		46.778971	0.832	-0.26
18531747		14.089805	1.021	0.03
420144.85		186.25675	0.02	-5.67
8475622.8		66.754044	0.328	-1.61
16164510		28.078887	1.545	0.63
19533420		12.087201	0.971	-0.04
16823510		16.811388	1.215	0.28
13656061		17.777256	0.531	-0.91
12889931		41.794064	0.67	-0.58
831976.35		85.264677	0.032	-4.95
15802132		9.5534196	0.616	-0.7
17666134		53.492021	7.86	2.97
15022255		69.474674	11.382	3.51
19447994		10.449635	19.771	4.31
19141390		7.2716737	0.747	-0.42
20423644		5.4271746	0.918	-0.12
17869937		8.0249593	0.698	-0.52
19041420		6.0925378	1.216	0.28
18410531		17.927227	1.055	0.08
15650765		40.111351	0.705	-0.5
233394.28		193.26131	1.034	0.05
18915782		15.271055	0.811	-0.3
20960877		7.5137747	1.004	0.01
20112348		5.3510914	1.127	0.17
15194508		4.1767057	0.599	-0.74
19306879		17.791245	1.868	0.9
20734051		19.736101	1.502	0.59
20169544		14.741818	0.98	-0.03
18863996		12.775686	1.476	0.56
17421244		14.856923	0.714	-0.49
18144231		7.4926213	1.089	0.12
15802683		20.725897	0.953	-0.07
20347357		3.3764121	1.148	0.2
14659824		48.465871	0.677	-0.56
14360070		12.848515	0.568	-0.81
18608402		26.314525	0.737	-0.44
19856139		9.6897819	0.96	-0.06
17411034		28.61188	1.138	0.19
20563311		3.8594922	8.029	3.01
18887721		8.9662772	0.758	-0.4
18770218		15.05907	3.263	1.71
16421387		27.71299	1.064	0.09
18484751		19.902704	0.823	-0.28
17866971		13.092104	1.114	0.16
15106719		30.274268	0.747	-0.42
17205844		20.402873	0.731	-0.45

Only

14924775		46.571146	1.074	0.1
20224488		3.7840873	0.845	-0.24
16208923		30.486185	0.883	-0.18
20327444		2.8766086	1.472	0.56
1896464.6		19.348852	0.076	-3.72
14819961		46.848015	0.897	-0.16
17950029		12.344467	1.087	0.12
18666097		28.72661	0.811	-0.3
13028760		38.197299	1.507	0.59
12262407		31.293064	0.491	-1.03
16865207		23.706527	0.692	-0.53
19811591		4.8153961	0.945	-0.08
8664976.7		98.243594	0.45	-1.15
19431074		7.9580744	1.582	0.66
18098459		15.760689	3.728	1.9
18008888		8.6759724	0.838	-0.25
1123604.1		5.9321279	0.045	-4.47
16842693		60.211413	0.805	-0.31
9741529.4		103.95083	0.787	-0.35
15290366		18.617798	1.127	0.17
18664422		11.883453	0.879	-0.19
11516102		4.0091187	0.464	-1.11
16412989		18.822777	1.209	0.27
1676727.8		52.043191	0.068	-3.89
14970592		12.958158	0.604	-0.73
10432652		44.9414	1.215	0.28
17252288		22.368755	1.021	0.03
18294293		24.50752	0.964	-0.05
15444387		62.704713	12.041	3.59
20220910		5.3843658	0.819	-0.29
17819249		12.529946	0.949	-0.08
721958.78		10.168361	0.029	-5.1
17138073		41.55994	1.008	0.01
14976915		21.502265	1.02	0.03
12403372		34.770192	1.155	0.21
16067853		20.674192	1.044	0.06
18481822		11.111793	1.241	0.31
16868457		12.406308	1.111	0.15
18963754		3.5527585	5.195	2.38
16973386		33.688526	1.288	0.37
10890212		46.711197	2.821	1.5
14659339		21.482993	2.343	1.23
19341239		16.525815	0.866	-0.21
18415444		14.219321	1.193	0.25
6243186.1		79.844546	0.485	-1.05
17761011		24.610432	1.374	0.46
16438826		16.528023	0.673	-0.57
15981245		15.026367	1.148	0.2
18362995		6.8383147	1.698	0.76
16662212		17.975208	1.54	0.62

Only

18591339		12.856736	0.98	-0.03
18080030		12.992085	0.893	-0.16
19781821		4.471532	5.045	2.33
15667145		18.245799	0.899	-0.15
17572457		15.27994	1.063	0.09
16164369		40.854908	1.355	0.44
425782.49		184.66155	0.215	-2.22
17362495		9.7770348	0.717	-0.48
19667065		4.8966582	10.225	3.35
1824980.6		154.78686	4.039	2.01
6682607.1		21.922849	0.277	-1.85
19382768		3.1646247	0.802	-0.32
18483970		11.108926	1.251	0.32
17279497		24.259495	1.545	0.63
19089506		2.0905336	17.694	4.15
19065084		2.056607	1.032	0.05
17583472		57.854823	0.982	-0.03
9943723.6		110.45547	1.606	0.68
17610774		10.484658	1.684	0.75
17935461		14.336296	2.273	1.18
15986162		10.879401	0.664	-0.59
16923126		19.855041	1.329	0.41
12835246		44.924761	0.906	-0.14
18047062		7.7770842	0.854	-0.23
19643201		4.6779938	9.978	3.32
18657489		40.888104	0.814	-0.3
19186760		23.895388	1.069	0.1
17441644		8.3158828	1.26	0.33
9619907.7		59.041905	1.56	0.64
673169.56		14.229624	0.028	-5.15
18390218		9.195493	0.905	-0.14
18703195		9.4557974	0.918	-0.12
18361152		7.5040725	0.77	-0.38
17553761		6.9062691	0.935	-0.1
17314042		29.073116	4.462	2.16
16112099		16.480681	1.418	0.5
18501856		10.027441	1.423	0.51
14952280		37.161782	2.406	1.27
19208008		4.8712726	19.887	4.31
18495537		17.96558	0.791	-0.34
15862657		7.5831964	0.668	-0.58
12765115		38.127255	0.978	-0.03
18978494		8.2267043	0.876	-0.19
14635854		19.304934	15.526	3.96
11149351		70.93002	5.359	2.42
12628713		29.489081	0.712	-0.49
15318751		17.830098	0.863	-0.21
18126782		7.3562484	0.837	-0.26
14749544		63.109379	0.624	-0.68
16345667		18.295815	2.2	1.14

Only

16910107		15.21747	0.845	-0.24
17722277		21.366586	1.886	0.92
15306463		17.21169	1.593	0.67
15600972		22.227527	0.713	-0.49
16630442		17.566692	1.223	0.29
517043.63		15.874685	0.022	-5.51
970305.08		9.2224178	0.041	-4.59
18210933		43.715819	1.085	0.12
3239651.5		120.61441	0.184	-2.45
13278105		23.042875	1.101	0.14
16412904		31.299512	1.179	0.24
15313749		18.320714	1.259	0.33
17352003		10.888093	0.832	-0.26
17383595		8.0267755	1.373	0.46
17466613		9.9446355	1.641	0.71
19137170		6.679328	0.896	-0.16
18765680		5.173955	1.076	0.11
15120194		20.070085	0.807	-0.31
17739024		9.6850717	1.225	0.29
16197526		20.865794	0.695	-0.53
13304374		22.058228	1.343	0.43
11562701		13.617734	0.496	-1.01
15943271		58.876407	0.783	-0.35
13982268		24.695349	0.601	-0.73
5696835.2		82.229126	2.086	1.06
15646222		20.16716	1.34	0.42
12600941		63.607025	1.497	0.58
17534493		5.7842452	2.085	1.06
816007.87		173.19366	1.342	0.42
16823035		12.905434	1.662	0.73
14007498		44.346885	0.68	-0.56
17272407		8.1507847	0.951	-0.07
15723370		11.314275	1.263	0.34
12474451		13.728756	0.541	-0.89
17695531		6.3919239	0.803	-0.32
955294.88		6.6172776	0.041	-4.59
16091792		9.3332051	0.698	-0.52
7520827.8		34.396973	0.326	-1.62
17326317		13.817612	1.862	0.9
17573582		8.1622084	1.06	0.08
17379402		12.43697	0.896	-0.16
17807173		5.0705139	1.657	0.73
1862041.5		47.622186	0.081	-3.62
17566978		9.5177364	1.199	0.26
13992713		31.252616	1.118	0.16
18023248		8.8684065	1.138	0.19
15233522		33.993514	2.112	1.08
17101414		12.698652	1.288	0.37
13881044		17.604098	1.303	0.38
17069587		14.425108	0.864	-0.21

Only

18581122		19.844336	0.858	-0.22
17647894		12.622627	1.331	0.41
17347921		4.5444753	11.426	3.51
17216783		9.2109758	1.542	0.63
15563959		12.029326	1.399	0.48
13506427		20.54692	1.646	0.72
17038159		45.415431	1.615	0.69
14670644		17.418324	0.785	-0.35
4334684.2		119.87938	4.881	2.29
13119217		30.749949	1.11	0.15
17392684		16.016662	0.893	-0.16
15896106		26.818491	1.413	0.5
13669307		21.131466	1.502	0.59
15843589		8.8358073	0.832	-0.27
18074374		5.9837823	1.034	0.05
12619487		34.746951	0.988	-0.02
11832865		30.488507	0.81	-0.3
9173521.2		13.636558	0.404	-1.31
17348742		6.767958	0.897	-0.16
15533561		11.960759	1.199	0.26
16662776		30.637685	1.042	0.06
18027001		3.7448347	0.969	-0.05
17386858		16.07283	0.951	-0.07
13056619		30.370272	3.516	1.81
15925623		17.911102	1.65	0.72
15616019		25.113309	3.488	1.8
13083013		38.965145	1.059	0.08
17732242		7.2597421	41.653	5.38
15137521		16.247549	0.938	-0.09
17894399		13.159121	1.054	0.08
2288008.4		45.915151	0.102	-3.29
16307040		21.67048	1.19	0.25
1950660.4		145.38936	0.313	-1.68
11811407		18.232122	0.528	-0.92
18196799		5.4516928	1.991	0.99
14147706		7.810841	0.633	-0.66
17979382		6.12421	0.829	-0.27
16543682		10.69988	1.081	0.11
14898674		13.843801	1.096	0.13
16278085		16.073617	1.118	0.16
16824454		25.934284	1.062	0.09
402280.06		36.191773	0.018	-5.79
17574790		7.4379239	1.349	0.43
15341733		22.018316	1.408	0.49
16121833		15.44288	0.804	-0.32
15574612		13.516826	1.112	0.15
14932523		17.220506	1.449	0.53
16881356		9.5956775	1.352	0.43
17986269		15.25012	0.997	0
17450458		4.4111874	1.078	0.11

Only

12304473		37.207478	1.172	0.23
17645572		11.629598	1.214	0.28
8376630.6		18.350106	0.379	-1.4
8351896.3		14.583475	0.379	-1.4
14781265		15.693695	0.701	-0.51
16964044		9.9716992	2.63	1.4
9614151.9		26.710552	0.437	-1.19
6623951		10.452917	0.301	-1.73
8109357.8		109.98704	17.046	4.09
12850643		25.396589	1.022	0.03
13127888		9.7194869	0.599	-0.74
16695167		12.328952	1.122	0.17
16270043		14.189752	18.689	4.22
16336674		25.987238	2.885	1.53
16334638		12.128858	0.848	-0.24
15899545		19.765589	0.77	-0.38
17160346		5.981974	0.844	-0.25
16416232		13.055813	0.957	-0.06
8703136		55.346261	0.695	-0.53
11070278		47.544454	0.571	-0.81
17184741		5.6980525	5.033	2.33
17050924		7.300946	1.103	0.14
16260472		9.9687949	0.793	-0.33
16882530		14.563555	0.869	-0.2
7509318.5		108.84197	0.454	-1.14
15898356		6.5633811	1.661	0.73
12969370		24.556383	0.597	-0.74
9005666.3		41.140308	2.789	1.48
2207165.9		38.628325	0.102	-3.3
15957283		5.1452249	0.736	-0.44
15344191		14.991983	3.337	1.74
7136489.2		30.031631	0.329	-1.6
5140646.5		91.139139	0.584	-0.78
17315227		3.4605769	0.809	-0.31
14535493		15.715923	1.382	0.47
15993128		13.36739	1.005	0.01
408095.3		25.400867	0.019	-5.73
16873647		8.7158938	7.107	2.83
14033470		26.025627	0.876	-0.19
17280280		4.8723113	0.962	-0.06
15708056		2.5359603	0.728	-0.46
17403849		2.7516495	0.808	-0.31
14722576		23.523605	1.173	0.23
7768410.9		40.687613	0.361	-1.47
14053025		46.158039	11.302	3.5
16264200		6.5268278	1.469	0.55
15785634		10.570017	1.888	0.92
16987937		9.5443116	1.302	0.38
15808864		59.612472	11.532	3.53
15703773		10.284353	0.976	-0.03

Only

15845605		13.226445	1.033	0.05
11937761		32.18723	0.876	-0.19
14201610		6.2031361	0.666	-0.59
15144439		6.2539276	0.71	-0.49
15342038		18.758865	0.756	-0.4
17393859		4.0743215	0.897	-0.16
12888810		28.277195	0.606	-0.72
13331168		26.847778	5.828	2.54
13456487		20.420375	1.067	0.09
12600763		17.578162	1.086	0.12
16338947		5.8297572	1.41	0.5
225944.99		39.430294	0.011	-6.55
6981191.5		97.728952	0.35	-1.52
16994842		5.7160981	1.044	0.06
15728677		7.678073	1.927	0.95
13389378		19.284426	0.778	-0.36
14686793		14.824782	0.94	-0.09
10588546		34.520635	0.524	-0.93
10748511		19.25301	0.508	-0.98
16197556		7.9144397	0.867	-0.21
14938035		2.6693494	0.707	-0.5
13147762		37.939889	0.623	-0.68
14214947		19.10515	0.814	-0.3
1172880.3		23.441458	0.056	-4.17
13905561		19.321963	0.919	-0.12
16748564		6.6294348	19.055	4.25
17086287		7.3110976	0.958	-0.06
16881388		2.6044222	0.935	-0.1
14246683		20.389426	0.873	-0.2
15711170		11.08079	0.888	-0.17
16393037		9.9756527	1.207	0.27
16730375		5.5321565	0.927	-0.11
15036044		23.00108	2.945	1.56
12838643		22.362073	21.973	4.46
16475143		18.937487	0.796	-0.33
16323450		11.217152	1.383	0.47
17250494		11.432707	0.972	-0.04
13569084		34.987719	1.462	0.55
16401321		10.359022	2.113	1.08
17295431		3.6337242	0.957	-0.06
15792334		10.196765	0.758	-0.4
14247617		14.586339	3.867	1.95
14444696		13.521823	0.961	-0.06
17233889		5.0670148	1.479	0.56
14060060		21.943944	1.27	0.34
13079516		40.088136	13.398	3.74
11824055		35.825687	1.331	0.41
15795222		3.9550194	0.976	-0.04
16122719		7.4031336	16.978	4.09
13232411		14.979346	1.022	0.03

Only

11591843		16.712686	0.563	-0.83
6490117.3		24.759257	0.315	-1.67
15758786		10.111055	1.1	0.14
16014757		11.094936	0.997	0
12895766		36.634983	6.384	2.67
14816050		13.863169	1.35	0.43
16274469		10.044625	1.011	0.02
13131899		41.779255	0.971	-0.04
12309647		14.384898	0.601	-0.74
10878470		24.97102	0.542	-0.88
10123438		57.355516	1.794	0.84
16055901		4.7266428	0.937	-0.09
14250712		9.2289263	0.881	-0.18
15317107		16.072087	0.909	-0.14
16282335		4.1055265	0.985	-0.02
16034247		8.4256501	1.148	0.2
6618149.7		115.1433	21.456	4.42
15372989		13.971179	1.461	0.55
13041891		15.551581	0.859	-0.22
13217584		18.370872	1.041	0.06
8450630.9		31.675744	0.415	-1.27
12577483		18.804047	1.012	0.02
10479196		43.673978	0.866	-0.21
12667048		45.94495	0.885	-0.18
16230305		9.0485027	1.3	0.38
12794832		27.3507	0.901	-0.15
15269048		4.6956481	1.85	0.89
16079427		16.464621	1.114	0.16
5059032.7		40.999975	0.249	-2.01
11684927		19.445992	0.696	-0.52
11683400		26.125224	1.515	0.6
16507398		2.8975118	1.178	0.24
14724715		7.2346787	0.842	-0.25
11046337		28.772543	0.691	-0.53
229907.29		29.558624	0.011	-6.46
16607494		3.6850391	0.937	-0.09
16245807		15.419593	2.891	1.53
14728542		13.606386	0.736	-0.44
13493879		16.937428	1.238	0.31
8395575.4		50.688458	1.743	0.8
10046655		34.226013	1.031	0.04
10695359		18.28816	0.532	-0.91
14155138		22.684032	2.859	1.52
13739301		15.445056	0.836	-0.26
2889461.9		128.41914	0.157	-2.67
14258413		58.940496	2.614	1.39
14305014		9.6190181	1.121	0.16
15319550		6.0993679	0.912	-0.13
11234856		52.2211	0.832	-0.27
15905233		4.2431133	3.757	1.91

Only

7559819.4		57.574422	0.665	-0.59
14502358		17.799942	1.5	0.59
7479300.1		81.674973	0.41	-1.29
15215530		8.5784853	0.95	-0.07
14189425		13.500688	0.965	-0.05
7489377.3		55.04569	0.375	-1.41
6993652.9		40.788938	0.351	-1.51
15331496		15.14836	2.159	1.11
13966857		12.865637	1.338	0.42
7493787.8		11.66379	0.377	-1.41
1509218.6		142.98376	0.929	-0.11
14913688		11.779039	1.254	0.33
14501452		11.090199	1.56	0.64
11184602		24.155281	0.778	-0.36
6360145.5		61.322258	0.321	-1.64
10830480		22.789193	0.548	-0.87
10479670		29.195315	1.476	0.56
723601.95		69.305929	0.037	-4.77
9190266.6		36.379304	1.014	0.02
453478.27		56.186173	0.023	-5.44
14785756		13.237821	1.582	0.66
16271529		8.8758853	1.153	0.21
15843421		9.2433057	0.843	-0.25
15381777		7.1052806	0.875	-0.19
14472919		16.142153	0.846	-0.24
14919192		8.7765604	1.67	0.74
15393909		10.871626	1.063	0.09
12099431		21.820256	0.706	-0.5
13468539		15.429388	1.063	0.09
15858164		2.2882126	1.052	0.07
12057629		20.674512	1.549	0.63
14405826		12.371567	1.223	0.29
15538997		44.977153	1.304	0.38
15187465		6.7997028	4.214	2.08
14454122		6.3105364	13.337	3.74
12859087		21.070003	0.795	-0.33
10038023		44.358845	0.516	-0.95
13684621		24.233849	1.123	0.17
15560258		13.958708	0.953	-0.07
14478604		25.40634	2.156	1.11
12283689		41.98508	1.105	0.14
14893075		6.0798466	1.023	0.03
7161160.6		27.941992	0.369	-1.44
10143008		26.872952	0.523	-0.94
1264200.9		9.6172648	0.065	-3.94
14237743		23.2588	0.735	-0.44
15902298		7.2449147	5.14	2.36
15985811		4.5883416	1.003	0
13764812		17.05882	10.299	3.36
14782336		4.4912836	0.893	-0.16

Only

14231989		14.171806	0.986	-0.02
11141717		40.348549	1.382	0.47
15056363		6.7235533	0.837	-0.26
13426308		11.727264	1.157	0.21
3947718.1		38.241083	0.205	-2.29
661507.23		41.064106	0.034	-4.87
15538927		5.742509	3.42	1.77
15538927		5.742509	3.42	1.77
12221923		12.270731	0.634	-0.66
13623666		21.499928	6.595	2.72
14067918		14.892496	1.592	0.67
14373510		58.054225	1.325	0.41
12312274		34.149967	1.168	0.22
10819970		27.043149	0.854	-0.23
13529543		25.305503	1.78	0.83
13183247		14.368603	1.136	0.18
14677406		5.1658726	1.031	0.04
8514163.9		45.626425	1.017	0.02
2633610.2		42.50578	0.138	-2.86
3067473.5		102.41415	0.934	-0.1
14368337		6.9315163	3.412	1.77
11583414		25.245747	1.191	0.25
14744674		5.4601547	0.899	-0.15
15204591		11.842822	1.367	0.45
11901486		40.521476	1.907	0.93
14689485		17.537742	1.381	0.47
13175433		12.576523	0.784	-0.35
14541058		6.5170877	1.227	0.3
10521437		11.2932	0.554	-0.85
7612781.5		15.985546	0.401	-1.32
4902221.2		34.68997	0.258	-1.95
12201925		10.455257	0.643	-0.64
14776225		9.9441007	11.048	3.47
14009519		11.36094	5.596	2.48
13799069		9.2149896	0.766	-0.39
3235529.8		58.222507	0.171	-2.55
5647298.3		21.786466	0.298	-1.74
13391335		9.2501826	0.708	-0.5
11568550		23.034648	0.782	-0.35
14392162		8.7641328	0.886	-0.17
13396649		8.3427571	0.759	-0.4
12312614		14.903861	0.955	-0.07
13086503		11.60616	2.459	1.3
13749597		13.950643	0.729	-0.46
5499551.7		65.921882	0.292	-1.78
13077443		17.956222	0.854	-0.23
13915568		11.220678	1.334	0.42
12808711		14.719723	0.875	-0.19
14998855		3.3843119	1.351	0.43
13056139		11.720656	0.871	-0.2

Only

8035439		43.857534	0.528	-0.92
11705104		22.104908	0.923	-0.12
15430812		6.6016465	1.18	0.24
11570456		29.760887	1.222	0.29
14265579		3.5816337	1.151	0.2
14779371		48.066933	1.013	0.02
14702935		6.8372205	0.971	-0.04
10913292		58.205554	0.755	-0.41
13187916		29.764301	0.898	-0.15
12993705		15.551875	0.738	-0.44
2164956.8		7.4288453	0.116	-3.11
11540674		23.160824	1.091	0.13
11508880		35.53519	0.7	-0.51
12621611		12.896961	1.124	0.17
6578464.5		51.191669	0.352	-1.51
14142210		24.972072	1.198	0.26
12137663		36.175509	0.651	-0.62
14467086		3.4726143	1.734	0.79
4407121.8		61.582309	0.237	-2.08
15037079		5.6822274	1.071	0.1
14002662		17.466765	0.855	-0.23
12676896		16.428037	1.174	0.23
15185208		6.577141	0.914	-0.13
13479651		9.5569927	1.779	0.83
9455333		32.367557	0.782	-0.35
15222553		9.8566352	1.722	0.78
6214770.4		112.20633	4.782	2.26
8744078		29.720203	0.471	-1.09
14508658		9.3463794	0.787	-0.35
13344451		5.4453052	0.721	-0.47
11219483		25.164764	0.662	-0.59
13829056		6.3888261	0.776	-0.37
10901238		40.307768	1.695	0.76
13219469		18.228035	0.729	-0.46
13831651		11.695707	1.238	0.31
12808001		10.480907	10.541	3.4
11908253		62.113627	0.647	-0.63
14460575		9.1763947	22.548	4.49
14546746		39.90718	17.431	4.12
14801489		10.574754	1.156	0.21
11746467		20.898945	1.92	0.94
13488718		14.808944	0.953	-0.07
14322861		16.113226	1.458	0.54
10401022		11.983235	0.566	-0.82
14386947		18.093037	5.521	2.46
10267014		32.870461	24.753	4.63
14139428		11.045177	8.218	3.04
14381531		6.0420143	0.784	-0.35
7344725.4		46.985585	0.401	-1.32
15195985		4.9311737	0.854	-0.23

Only

13020322		13.383304	2.509	1.33
3104111.3		128.24297	10.178	3.35
14104141		8.5169238	0.771	-0.38
12537007		13.924958	0.928	-0.11
5914408.8		10.296246	0.324	-1.63
651140.76		135.21637	0.036	-4.81
2416195.9		95.087252	0.132	-2.92
1249632		28.023508	0.069	-3.87
12864147		21.689306	1.143	0.19
14894582		4.344109	1.458	0.54
13602688		10.310254	0.834	-0.26
14233028		16.928431	1.274	0.35
11842040		22.498218	1.318	0.4
8172645.4		75.210122	2.683	1.42
2837052.6		19.9356	0.156	-2.68
13431687		9.177351	0.861	-0.22
14583839		5.4559552	1.034	0.05
11954300		26.464762	4.177	2.06
11546944		8.4231899	0.636	-0.65
1193070.6		17.942431	0.066	-3.93
13467873		7.0703993	1.105	0.14
12404851		12.646456	0.685	-0.55
12716477		13.143446	0.857	-0.22
11430085		21.351995	6.683	2.74
5860738.9		18.557146	0.325	-1.62
8765380.5		53.119729	0.621	-0.69
4339969.1		34.880602	0.241	-2.05
2443805.6		64.302594	0.136	-2.88
14137352		15.385546	1.427	0.51
13599112		19.835962	1.121	0.17
14472652		2.7695866	1.329	0.41
14081425		15.451725	3.789	1.92
13432722		27.030238	1.458	0.54
4645615.7		45.972239	0.259	-1.95
13245828		8.8431877	1.005	0.01
6438138.9		51.720503	0.361	-1.47
13023282		12.150562	0.863	-0.21
11880244		17.736246	0.994	-0.01
14184533		7.3965506	1.085	0.12
12456355		19.135724	0.956	-0.07
11332048		25.562989	1.714	0.78
11486846		24.680151	0.799	-0.32
13596473		7.7775103	0.869	-0.2
11493765		18.41466	0.831	-0.27
14080568		8.6346725	0.992	-0.01
12677226		10.743597	2.894	1.53
14122446		11.381108	1.401	0.49
499999.7		7.3180685	0.028	-5.14
9994947.3		28.037923	6.589	2.72
1218903.9		6.7732641	0.069	-3.86

Only

9812439.8		10.760102	0.556	-0.85
11296925		16.280117	0.735	-0.44
11492494		18.873761	1.021	0.03
10098854		21.032241	1.251	0.32
12965239		23.493769	0.96	-0.06
8157789.4		37.898153	0.464	-1.11
4360847.6		21.557129	0.248	-2.01
10921709		44.509298	1.013	0.02
10400075		14.490792	0.593	-0.75
12050366		10.275135	0.872	-0.2
13538046		5.2260245	1.116	0.16
2865407.4		43.802103	0.163	-2.61
9309262.1		9.4926619	0.532	-0.91
10513934		20.852645	1.765	0.82
14307059		4.7967305	0.926	-0.11
8913567.9		35.145411	1.191	0.25
2500139.5		33.401573	0.143	-2.8
12490563		17.475037	13.216	3.72
11536325		16.501569	0.662	-0.6
473549.43		7.3039529	0.027	-5.2
10159447		24.25553	1.225	0.29
14239541		6.3633775	3.68	1.88
3024456.9		109.49756	1.595	0.67
11749384		25.703826	0.693	-0.53
12469035		11.706206	1.326	0.41
12441010		30.393457	8.837	3.14
12701308		11.246861	0.917	-0.13
10826723		27.757166	0.691	-0.53
13035019		23.059743	7.332	2.87
12377353		19.510444	2.903	1.54
12352309		14.955392	22.03	4.46
14152131		11.100197	1.11	0.15
6988857.6		18.164259	0.406	-1.3
2521821.8		5.5296713	0.146	-2.77
10700385		21.477355	1.335	0.42
1088001.3		15.182034	0.063	-3.98
513604.86		17.805146	0.03	-5.07
9859108.4		32.918397	0.643	-0.64
9513209.6		25.361307	1.343	0.43
12081628		9.6016599	1.008	0.01
13114125		10.469235	1.31	0.39
12015865		19.396746	1.543	0.63
13162686		10.199284	1.785	0.84
13697327		10.168548	0.922	-0.12
9560979.8		26.120107	0.995	-0.01
269768.34		29.558624	0.016	-5.99
10930393		18.145949	1.123	0.17
10870303		26.271852	1.035	0.05
14268010		3.6893443	6.129	2.62
5354317.8		64.375363	0.314	-1.67

Only

13257071		5.5100843	1.567	0.65
11280345		15.445145	1.002	0
9231000.3		9.2999938	0.543	-0.88
13041182		6.7158704	1.095	0.13
12634458		9.4604888	0.744	-0.43
14000939		5.9782096	0.85	-0.23
13618147		8.118319	1.368	0.45
9229443		26.965226	0.881	-0.18
12681075		17.036269	12.176	3.61
13246229		6.0465152	1.106	0.14
13253104		7.640374	1.727	0.79
10575046		25.138089	1.037	0.05
13215958		7.7501254	1.083	0.12
13215958		7.7501254	1.083	0.12
11742909		14.035536	1.47	0.56
5645006.3		44.757397	0.334	-1.58
12855656		10.419809	0.765	-0.39
8092062.3		16.337236	0.479	-1.06
9172279.8		35.807608	1.043	0.06
9610032		37.569084	1.208	0.27
12887945		4.8455641	0.917	-0.13
12381375		9.2081559	0.987	-0.02
12236976		16.088421	2.157	1.11
12334340		16.288518	0.855	-0.23
13640219		7.6970567	1.028	0.04
13611170		12.850702	9.63	3.27
12380694		15.7323	0.931	-0.1
7141557.5		8.9639647	0.426	-1.23
10644839		17.047357	1.835	0.88
13506066		3.5853091	0.914	-0.13
11302783		62.722712	9.774	3.29
8792105.6		32.862466	0.935	-0.1
7439948.1		36.715416	0.927	-0.11
7055898		35.976548	0.423	-1.24
11254155		14.20018	0.676	-0.57
11109898		16.781743	0.944	-0.08
11032867		12.532488	0.726	-0.46
9404113.3		26.129675	27.813	4.8
12392083		15.410512	2.004	1
12271343		10.820777	4.885	2.29
10830520		40.477172	10.2	3.35
10274394		20.240387	0.619	-0.69
3275855.3		49.156073	0.197	-2.34
3436754.4		62.807326	0.207	-2.27
1817237.7		29.620721	0.11	-3.19
5206160.9		58.511507	0.314	-1.67
9086201		40.569686	1.486	0.57
11901873		7.2435681	1	0
12800373		5.9525698	0.882	-0.18
1931427.3		121.00637	0.922	-0.12

Only

12209440		55.911877	0.82	-0.29
12585308		4.3833103	0.799	-0.32
3327688.6		51.664163	0.202	-2.31
13115461		5.3438437	1.087	0.12
10983071		24.760559	0.792	-0.34
10990245		17.609466	1.972	0.98
12624882		9.8547734	1.155	0.21
11631065		21.665325	1.049	0.07
11547818		21.038964	0.852	-0.23
11602738		14.852028	1.307	0.39
11435620		15.806146	2.096	1.07
13156062		9.7843223	1.065	0.09
12359157		9.3057871	0.956	-0.06
10714725		17.507124	1.347	0.43
12445733		15.162167	4.048	2.02
12652354		15.884011	11.732	3.55
8430386.7		7.6915367	0.516	-0.95
11496789		9.6709851	0.938	-0.09
11623307		17.146352	1.046	0.06
11555656		22.260372	8.494	3.09
12233608		7.3200931	0.924	-0.11
12591499		8.5384813	1.022	0.03
12167614		22.060347	0.94	-0.09
12202929		5.8504796	1.709	0.77
6296187		82.004835	0.387	-1.37
12845641		6.5456831	1.072	0.1
12795702		8.3392997	6.182	2.63
10866774		10.714777	1.358	0.44
12857921		8.1306641	1.321	0.4
10724265		20.504086	0.873	-0.2
12122223		8.1820929	1.481	0.57
6920361.9		86.204039	3.054	1.61
10604710		17.283603	0.656	-0.61
10714435		22.81365	1.452	0.54
8988337.9		23.688834	1.021	0.03
10775908		18.982862	20.428	4.35
12155360		15.068586	5.436	2.44
11072731		13.93932	0.859	-0.22
11866625		15.995358	1.516	0.6
12619996		7.26597	45.899	5.52
13230756		6.2423417	1.045	0.06
12256476		8.8419863	1.077	0.11
11112843		15.790826	0.968	-0.05
12440125		9.508796	0.896	-0.16
12060193		10.128918	0.753	-0.41
12666294		3.6243681	1.296	0.37
11267848		21.135123	0.991	-0.01
8475074.8		19.518921	0.53	-0.92
12762047		9.1019526	1.421	0.51
2471874.8		8.1965318	0.154	-2.69

Only

593490.02		7.4390465	0.037	-4.75
6041335		59.675849	2.228	1.16
8787225		10.909571	0.55	-0.86
9722717.9		20.569921	1.076	0.11
7321576.2		34.120467	0.504	-0.99
6991977.2		34.302998	0.44	-1.19
6668884.4		29.32715	0.421	-1.25
8017506.4		13.302779	0.506	-0.98
12905545		6.0681566	1.261	0.33
6513252.7		37.635634	0.412	-1.28
319376.42		25.400867	0.02	-5.63
10687840		13.566342	0.78	-0.36
12439108		9.6106659	1.411	0.5
499400.84		27.783687	0.032	-4.98
850002.49		18.8317	0.054	-4.21
10251016		14.539205	1.667	0.74
10520578		13.439685	1.109	0.15
11837248		14.413421	9.559	3.26
10476321		16.101552	1.144	0.19
10081716		13.222122	0.644	-0.63
10402561		15.805042	3.002	1.59
9116057.7		19.8704	1.696	0.76
12772642		0.5284612	1.179	0.24
9252482.1		35.53529	0.592	-0.76
11344570		11.579522	0.88	-0.18
12412852		6.3361912	1.386	0.47
9843968.9		65.940544	4.081	2.03
11919047		11.702604	1.045	0.06
12647261		6.8529372	4.647	2.22
12332424		9.1837612	11.951	3.58
10579871		24.731708	1.078	0.11
11674654		65.326186	36.906	5.21
11289990		10.575149	0.992	-0.01
10548626		14.342597	0.949	-0.08
11861032		12.840738	1.158	0.21
11480523		15.225893	2.279	1.19
12160715		15.34277	1.283	0.36
11968733		5.9124827	1.29	0.37
9716810		20.089506	0.724	-0.47
11093330		20.939624	1.422	0.51
11834218		13.470085	1.339	0.42
6758187.8		20.090282	0.438	-1.19
10032796		22.215254	0.706	-0.5
11429391		10.537435	2.474	1.31
11204170		15.826208	1.006	0.01
11366038		10.668479	0.944	-0.08
638140.17		104.92093	0.041	-4.59
8881474.9		26.396069	0.586	-0.77
12515349		7.1122674	1.095	0.13
6908945		66.632521	0.502	-0.99

Only

11954379		7.849505	1.142	0.19
11636871		18.026351	1.098	0.13
12782844		5.6967062	47.582	5.57
5224444.1		113.61335	0.623	-0.68
7885774.8		32.161797	3.361	1.75
12161694		10.016349	0.884	-0.18
5250720		7.6390119	0.342	-1.55
7940245.9		49.192915	1.245	0.32
12051187		7.389153	1.179	0.24
10840681		56.031149	5.873	2.55
9761710		36.7906	3.628	1.86
11413526		19.168421	1.411	0.5
6471995.4		10.40211	0.423	-1.24
10204210		15.120756	0.865	-0.21
11360681		8.0985532	0.926	-0.11
5260435.5		112.40732	14.173	3.83
11502599		10.587417	3.428	1.78
12097408		14.609161	1.244	0.31
11296515		14.817414	1.017	0.02
9861735.1		17.404661	0.648	-0.63
10830495		20.527937	1.009	0.01
10614307		14.008547	0.844	-0.24
11052143		36.703846	1.008	0.01
9367540.6		43.934858	0.735	-0.44
8586809.3		21.783294	0.567	-0.82
6652982.4		37.15316	0.44	-1.18
11467317		17.153164	1.045	0.06
10952582		12.006763	8.201	3.04
8714726.6		11.186679	0.577	-0.79
11968881		57.226608	1.082	0.11
10055472		24.908764	1.856	0.89
12249277		6.0583687	0.876	-0.19
10527490		15.953628	1.334	0.42
10524320		18.110351	1.866	0.9
10635899		12.538166	0.871	-0.2
1015275.5		3.8687231	0.068	-3.89
10322404		22.923927	2.337	1.22
9592694.1		17.093831	1.09	0.12
890118.74		6.0772714	0.059	-4.08
12052982		8.0692596	0.915	-0.13
11065548		8.0095336	1.373	0.46
12138390		6.755334	1.043	0.06
11549247		13.33692	1.106	0.15
8676761.1		40.079623	0.595	-0.75
12229470		2.201899	8.487	3.09
11731509		4.7963766	1.097	0.13
8825977.5		23.622225	0.691	-0.53
9250728.3		16.540832	0.784	-0.35
7705578.2		59.903229	2.211	1.14
9138509.4		26.645205	2.622	1.39

Only

11189230		12.093862	0.984	-0.02
9649713.7		15.149806	1.029	0.04
11591220		5.2245727	1.309	0.39
9625170.2		29.060927	0.681	-0.55
12193442		12.412413	0.862	-0.21
11374835		5.9997962	0.79	-0.34
11170200		9.1344722	1.328	0.41
10138441		12.011652	0.928	-0.11
11326343		6.0170892	4.423	2.15
9980929		14.186156	2.572	1.36
10543768		21.699856	0.933	-0.1
8450976.5		73.725036	0.807	-0.31
10680891		11.481728	0.72	-0.47
8193396.6		26.692014	1.147	0.2
7009008.6		18.900616	0.473	-1.08
11456551		17.825614	0.956	-0.07
7208917.7		30.685542	0.487	-1.04
11654511		11.84629	0.874	-0.19
11184513		16.12576	1.329	0.41
9719984.3		18.310129	0.66	-0.6
8136180.2		33.13422	1.3	0.38
7682996.1		40.650072	0.522	-0.94
11118789		9.4076018	0.771	-0.38
6372295.7		42.975766	0.435	-1.2
1081275.4		27.702914	0.074	-3.76
10060107		34.572953	1.077	0.11
10568149		10.579886	1.381	0.47
11542494		6.8827613	0.983	-0.03
12138970		24.422194	1.437	0.52
10655515		14.567222	1.564	0.65
9608710		21.994534	1.146	0.2
9028422.5		34.891713	0.745	-0.42
9889964.3		13.482089	1.181	0.24
10520811		13.307024	11.55	3.53
3853416.9		12.174528	0.265	-1.92
11552511		6.0184228	0.952	-0.07
3139422.7		25.373377	0.216	-2.21
11276908		10.821169	0.81	-0.3
8809136.2		17.012399	0.755	-0.41
422948.64		5.9190416	0.029	-5.1
2399856		114.35979	0.628	-0.67
10088518		14.52249	0.953	-0.07
11576150		14.098385	1.559	0.64
11233189		11.037852	0.83	-0.27
10768729		6.4350606	1.803	0.85
9521002.1		17.82262	1.094	0.13
11226119		12.536716	10.929	3.45
11398427		20.090093	0.903	-0.15
10295769		29.137078	2.404	1.27
6565712.9		31.504043	0.455	-1.14

Only

2028224.7		31.416836	0.14	-2.83
1224406.3		141.73514	0.739	-0.44
11039104		7.3659241	0.958	-0.06
7460099.6		53.288183	10.022	3.33
8697523.5		47.654016	0.953	-0.07
11628392		19.612443	0.967	-0.05
10551320		11.290764	1.164	0.22
10100951		12.918632	1.031	0.04
10368476		6.8375661	1.011	0.02
9205280.4		17.776686	9.397	3.23
5352363.8		53.662253	0.418	-1.26
10555875		24.149205	20.706	4.37
7516596.2		28.666985	1.314	0.39
1210276.5		6.6129027	0.084	-3.57
9218013.3		6.1035085	0.643	-0.64
9461110.2		18.984406	1.068	0.1
5792208		50.160121	0.438	-1.19
10681000		11.086951	1.019	0.03
10928760		12.909028	1.038	0.05
8941394.2		40.87356	1.231	0.3
11053918		5.8945778	1.284	0.36
11235104		6.7556613	33.521	5.07
5593757.2		52.724445	1.393	0.48
8956948.1		20.886502	1.241	0.31
8133859		66.88355	23.243	4.54
9113456.9		34.88461	1.057	0.08
11744497		2.67913	0.938	-0.09
10747375		32.126188	14.754	3.88
10560724		17.58295	0.748	-0.42
9155249.6		23.668303	1.287	0.36
2761138.4		85.748077	0.196	-2.35
9550618.1		15.334399	1.14	0.19
9270439.8		17.352829	0.988	-0.02
1022599.3		6.6327578	0.073	-3.78
7937545.7		13.959617	0.565	-0.82
8483879.9		21.18618	0.604	-0.73
10472245		12.124273	0.907	-0.14
1396382.8		131.30247	0.911	-0.13
10488564		5.0982412	1.398	0.48
11568927		4.295956	0.922	-0.12
6794663.4		40.681969	0.886	-0.17
10866047		9.4417491	5.656	2.5
5620472.6		49.196047	1.158	0.21
11011513		8.578477	0.871	-0.2
9545713.3		16.649685	1.043	0.06
9695913.1		6.6007697	0.693	-0.53
9850013.3		16.394744	1.254	0.33
11244248		7.1827597	0.922	-0.12
7943163		25.531115	0.911	-0.13
3352965.8		19.901533	0.24	-2.06

Only

9802304.8		17.139803	0.975	-0.04
9406490.7		14.245303	0.991	-0.01
10189048		17.625526	1.206	0.27
9459328.7		21.550025	1.025	0.04
1745807.1		122.19313	1.273	0.35
11046874		6.2586644	0.86	-0.22
10611373		13.130274	1.116	0.16
10472557		7.5941609	5.035	2.33
10147547		11.671006	1.114	0.16
9751471.3		14.293941	1.946	0.96
5038634.2		67.222722	0.69	-0.53
10527699		6.0899074	1.102	0.14
10418646		20.457719	1.683	0.75
9098178.5		15.567245	1.079	0.11
10122035		21.642955	0.785	-0.35
11056435		3.9848056	1.113	0.15
9996981.9		9.7266478	1.004	0.01
7150875.4		29.971904	0.979	-0.03
9730249.8		12.151095	1.124	0.17
9492267.7		12.545232	1.051	0.07
11134680		4.5040106	7.197	2.85
9176029.6		23.915498	0.668	-0.58
8685120.3		22.730223	9.014	3.17
1041521.7		34.57025	0.076	-3.72
8538045.9		20.188798	0.622	-0.68
11105012		5.0939228	0.967	-0.05
9168293.9		19.596229	4.421	2.14
5503078.7		11.622934	0.402	-1.31
7768916.1		40.145018	1.089	0.12
5384320.6		44.182842	0.491	-1.03
10337619		9.238702	10.804	3.43
10971791		8.6640227	0.832	-0.27
7329378.1		29.528331	0.722	-0.47
10140918		9.3935411	1.06	0.08
7841325.6		25.303768	0.897	-0.16
9015349		22.115622	11.182	3.48
9015349		22.115622	7.199	2.85
10677478		4.9777122	7.208	2.85
4354265.4		12.732612	0.319	-1.65
10534999		10.665431	1.198	0.26
10216683		8.4373093	5.64	2.5
9048563.6		24.315428	1.286	0.36
6104039.1		75.440052	0.532	-0.91
7931971.9		28.307249	1.252	0.32
831070.81		23.57234	0.061	-4.03
10310103		6.5817104	0.95	-0.07
9778568.7		7.1613905	1.022	0.03
10551145		4.5778777	1.224	0.29
9775624.1		15.670886	1.372	0.46
10744088		8.9715136	1.032	0.05

Only

8395787.4		23.197159	0.62	-0.69
3442832		39.330282	0.254	-1.98
7674659		28.62992	1.421	0.51
10353576		11.15326	15.258	3.93
10854765		1.3946017	0.986	-0.02
7568063.8		39.147267	1.737	0.8
10598913		41.539561	8.476	3.08
10749606		6.5242805	0.988	-0.02
8884401		16.533905	0.658	-0.6
3733090		34.778218	0.277	-1.85
10582530		5.7349145	0.895	-0.16
10880332		22.568136	2.595	1.38
9640000.2		19.99955	2.976	1.57
8589067.1		18.43162	0.638	-0.65
9549562.4		43.689144	0.73	-0.45
10944503		1.5674914	1.069	0.1
10051979		14.617065	1.259	0.33
9098581.1		13.797183	0.678	-0.56
4289038.4		9.3064328	0.32	-1.65
9824626		11.910738	0.839	-0.25
10169329		6.2969901	0.758	-0.4
10539725		6.1426061	1.058	0.08
8386723		4.0547059	0.626	-0.68
10764368		6.1237824	0.818	-0.29
9448244.7		15.339437	0.965	-0.05
7194339.6		39.740908	6.606	2.72
10316636		60.763526	0.884	-0.18
10065309		13.926739	0.758	-0.4
8098035.8		7.8368111	0.607	-0.72
8627782.1		29.231631	3.124	1.64
11066464		8.0229373	5.655	2.5
10359954		9.1122664	0.937	-0.09
9231230.3		20.636356	1.014	0.02
10434398		4.8670142	1.203	0.27
10756243		6.0970372	1.145	0.2
10001520		17.073161	0.933	-0.1
8791956.3		16.416532	0.967	-0.05
8972802.6		20.494595	0.675	-0.57
10159586		10.001585	1.03	0.04
10452459		3.1491518	5.748	2.52
10713578		3.9049734	1.422	0.51
4458585.7		19.385651	0.337	-1.57
7249088		26.949858	1.033	0.05
6245895.2		45.250909	4.76	2.25
9622908.5		23.87636	1.681	0.75
8756006.3		22.162344	0.734	-0.45
8116987.5		18.863744	0.948	-0.08
9399231.1		18.750668	0.712	-0.49
4688512.7		12.288541	0.356	-1.49
10138616		19.725854	1.013	0.02

Only

8948118.2		20.8028	0.957	-0.06
6813197.6		20.866839	0.518	-0.95
10527635		4.0754978	1.164	0.22
9628644.9		23.346686	0.906	-0.14
10395660		24.379292	1.111	0.15
8209117.4		29.252187	0.625	-0.68
9923847.1		8.906399	1.044	0.06
9760322.3		17.757463	0.77	-0.38
9072849.7		14.527674	0.957	-0.06
10343628		5.855881	1.065	0.09
8447415.2		25.535493	1.081	0.11
10352916		6.150281	0.968	-0.05
10237628		12.380701	1.433	0.52
6911514.7		59.897356	0.837	-0.26
9989253.8		8.5761898	1.294	0.37
9608781.1		12.109991	1.847	0.89
9953156.9		12.716912	0.915	-0.13
10459382		4.0109283	7.038	2.82
10466809		11.718695	1.128	0.17
6084654		7.9793848	0.467	-1.1
9074734.6		16.224541	1.004	0.01
3568907.5		18.720418	0.274	-1.87
10509604		6.1025288	0.971	-0.04
10778663		32.838291	1.079	0.11
9237734.8		10.61508	0.924	-0.11
7788863.8		6.6809257	0.6	-0.74
10341106		7.876275	1.046	0.06
300534.44		7.4390465	0.023	-5.43
9608627.8		8.0999397	0.74	-0.43
9661448.6		55.698304	6.889	2.78
10659266		10.953652	0.937	-0.09
8480038.2		25.634449	1.245	0.32
743982.36		119.36368	0.057	-4.12
6503241.6		59.265512	0.596	-0.75
9861783.1		7.1573825	1.19	0.25
10468886		4.6362363	1.063	0.09
8402804.4		12.66189	0.881	-0.18
9907556.9		5.3526617	2.088	1.06
9800682.3		10.186064	1.388	0.47
9154881.3		19.841006	1.685	0.75
9344592.8		13.814602	0.742	-0.43
5392305.7		110.01303	0.503	-0.99
9176140.1		22.612727	0.794	-0.33
9427967.9		4.4580225	0.738	-0.44
9907936.8		12.929337	1.097	0.13
8604398		15.325441	0.812	-0.3
10448248		1.3806385	0.991	-0.01
9478795.5		19.758934	2.705	1.44
9295898.5		11.788365	0.772	-0.37
3481611.3		75.886791	0.273	-1.87

Only

8781370.4		20.200032	1.671	0.74
8243412.5		26.087266	1.409	0.49
10386622		12.83962	1.158	0.21
9104680.1		11.651562	1.062	0.09
10248733		4.6101485	1.005	0.01
9443238.5		13.243007	5.875	2.55
3107109.4		21.114076	0.244	-2.03
9371442.9		9.7624324	0.798	-0.32
9239050.1		12.459006	1.489	0.57
8872435.9		13.885309	0.926	-0.11
8820468.1		17.372048	1.149	0.2
9113840.5		32.962191	13.38	3.74
8533186.3		18.31483	10.14	3.34
7825196.7		40.249227	0.714	-0.49
10113962		7.7176457	1.218	0.28
9396203.4		6.0569531	2.533	1.34
9439572.8		7.764341	2.071	1.05
9422126.8		13.635698	3.736	1.9
5902788.3		39.816884	0.681	-0.55
7111960		16.575987	0.566	-0.82
8629919.6		14.195493	1.107	0.15
2151867.8		123.48058	4.423	2.15
9048739.5		18.358755	1.01	0.01
9379219.6		25.85054	1.864	0.9
4403446.2		37.136987	0.351	-1.51
8439241.4		29.564401	0.862	-0.21
9351108.3		15.309262	1.608	0.68
10229470		4.1487802	1.388	0.47
8811707.1		9.0533643	1.229	0.3
3196877.2		101.11537	0.256	-1.96
4373780.7		84.082389	0.808	-0.31
9251868.3		14.456811	0.782	-0.35
8762767.3		12.084788	0.982	-0.03
3334614.5		36.891291	0.268	-1.9
946846.23		7.4663583	0.076	-3.71
3046232		29.665302	0.245	-2.03
9491600.1		17.849334	0.926	-0.11
9088199.7		15.137995	0.958	-0.06
9290549.4		35.343327	8.863	3.15
9163658.3		6.905537	0.96	-0.06
8467902.4		26.308853	1.029	0.04
454451.43		42.001145	0.037	-4.75
519649.38		130.12101	0.042	-4.56
9068066.3		5.4035386	0.742	-0.43
9673061.4		9.0284313	1.084	0.12
7691114.8		21.78154	1.619	0.7
7421485.3		27.084613	0.686	-0.54
7550891.2		27.505305	0.658	-0.6
9662561.4		4.1428489	35.234	5.14
6155787.7		36.438611	1.039	0.06

Only

8158707.8		28.152113	1.297	0.38
8976395		10.437732	0.739	-0.44
9499583.5		7.2915945	0.943	-0.08
7108142.4		56.31791	0.763	-0.39
5684557.9		11.042764	0.471	-1.09
6118577.9		23.514112	0.508	-0.98
9727347.3		13.680189	4.751	2.25
7199001.3		28.644705	1.762	0.82
7737035.4		23.117477	0.959	-0.06
7670507.6		19.432254	0.936	-0.1
9174185.8		9.7623826	0.896	-0.16
9425435.3		16.685661	7.261	2.86
9574855.4		8.9421337	0.868	-0.2
8451425.7		21.876494	0.795	-0.33
9219833.9		9.1746036	0.962	-0.06
8747116.6		26.723596	0.774	-0.37
7634038.4		16.839579	0.638	-0.65
9002208.9		5.9285056	0.752	-0.41
8901913.9		10.267883	1.031	0.04
9512847.8		12.441936	5.742	2.52
6594330.3		40.286159	0.685	-0.55
9576122.6		4.6479133	1.025	0.04
9457650.4		4.6093199	0.818	-0.29
9774865.3		4.1269759	0.98	-0.03
1438332.3		16.684485	0.121	-3.05
5475419.3		48.564614	0.539	-0.89
8534045.5		15.542381	1.291	0.37
9357688.6		14.947777	1.089	0.12
6698261.3		28.330029	0.679	-0.56
8767235.9		7.3791294	1.042	0.06
8100565.4		18.82536	1.124	0.17
9601781.3		4.2873426	1.396	0.48
9015478.2		10.707961	0.935	-0.1
3157828.7		38.728277	0.267	-1.91
6083301.1		51.186665	1.237	0.31
8160874.2		22.540027	3.185	1.67
7524511.5		25.696092	0.96	-0.06
8011457.7		9.4217859	0.68	-0.56
1510147		123.20452	0.695	-0.52
8217886.1		15.460902	0.698	-0.52
1360687		62.054552	0.116	-3.11
8969258		14.072824	0.843	-0.25
6239953.7		26.923901	0.532	-0.91
8829534.2		38.105618	1.324	0.4
7273122.2		18.835628	0.973	-0.04
9269692		5.689308	1.713	0.78
9446345.9		5.3079649	1.1	0.14
917395.1		2.2748416	0.079	-3.67
8408038		21.946354	2.172	1.12
7456911.2		20.639433	23.321	4.54

Only

9070361.7		7.5104773	0.88	-0.19
8681474.5		23.172094	1.058	0.08
7864775.9		20.786418	3.387	1.76
3327221		80.897826	0.287	-1.8
6627460.2		4.1702036	0.572	-0.81
7486685.6		19.961668	1.506	0.59
8955924.2		59.280809	30.358	4.92
8539726.1		8.8473241	1.561	0.64
9054605		61.161286	0.908	-0.14
2627936.6		85.829945	8.708	3.12
1221142.5		70.332584	0.106	-3.24
8062550.9		56.219101	0.877	-0.19
9230014.5		6.5164895	1.104	0.14
8070938		16.759607	0.834	-0.26
7719037.3		28.796344	3.138	1.65
8860402.8		9.7775385	1.287	0.36
8599094.7		14.072283	9.715	3.28
9075511.4		6.7137295	0.858	-0.22
5787442.6		62.163608	1.134	0.18
466875.29		121.63433	0.041	-4.62
8303374.2		10.845232	1.009	0.01
7423757.5		25.406333	1.157	0.21
9074742.3		3.144609	1.16	0.21
1918937.8		29.084493	0.168	-2.57
7072696		23.004859	1.456	0.54
7819100.4		13.425632	1.307	0.39
9338622.7		2.4054257	8.865	3.15
7551572		19.209126	0.804	-0.31
5844209		34.473414	0.887	-0.17
7573001.7		13.21699	2.189	1.13
8504190.7		34.662039	0.992	-0.01
8561649.8		25.584284	1.317	0.4
8014542.8		13.888479	1.589	0.67
6848287.1		9.8499379	0.603	-0.73
8674193.1		6.6194884	1.105	0.14
319825.4		59.356899	0.028	-5.15
594437.78		4.7675843	0.052	-4.25
5761390		33.383541	0.509	-0.97
8815226.2		12.702867	3.658	1.87
7917882.9		21.672342	2.09	1.06
9077302.5		4.6838722	1.109	0.15
5370638.8		32.409368	1.106	0.14
9024757.8		7.3772769	1.209	0.27
4551195.5		17.664666	0.405	-1.31
8009032.1		13.38034	0.712	-0.49
8155868.8		9.6035389	0.964	-0.05
3272274.3		83.064851	4.692	2.23
985267.6		150.01619	0.994	-0.01
7464474.4		14.308596	1.017	0.02
7404404.3		10.116376	0.661	-0.6

Only

6994624.8		23.353444	1.125	0.17
6919173.5		19.379629	0.795	-0.33
9200157.1		5.5496109	0.908	-0.14
6802033.6		27.490795	0.609	-0.72
8087260.4		57.390806	8.031	3.01
6841029.7		31.14599	0.877	-0.19
7233056.8		8.5228248	0.648	-0.63
1425612.5		11.923651	0.128	-2.97
5847778.5		60.654288	4.709	2.24
9062169.4		3.5375192	0.977	-0.03
4990868.7		43.072476	0.803	-0.32
8487986.1		9.7212265	1.088	0.12
8930972.4		11.285449	1.094	0.13
8660607.2		7.7306189	11.566	3.53
1135525.1		45.550266	0.103	-3.29
5555156.2		21.990172	0.502	-0.99
8415565.8		15.910333	0.792	-0.34
8284632.2		14.72446	0.969	-0.05
8424814		9.0536448	0.8	-0.32
8502901.5		20.219393	0.771	-0.38
4695206.3		48.823776	1.065	0.09
7250776.2		18.660579	1.461	0.55
7356709.7		31.938047	4.816	2.27
8988446		4.1807899	1.086	0.12
9186789.5		5.5364687	1.885	0.91
8158213.6		7.5189059	1.235	0.3
8547031.7		8.7123945	1.026	0.04
6716973.6		27.662569	1.18	0.24
8671015.7		8.0889427	1.203	0.27
6574167.4		27.237764	0.601	-0.73
8813170.5		22.349384	1.075	0.1
8145173.4		11.473106	1.508	0.59
861125.49		6.2864416	0.079	-3.66
8401152.1		8.2021289	1.012	0.02
3221612.7		69.804713	0.699	-0.52
7566219.9		24.535709	1.606	0.68
8585220.2		9.481921	0.789	-0.34
5654415.8		26.351729	0.52	-0.94
7183543.4		24.615118	0.823	-0.28
7776022.7		10.259835	0.872	-0.2
6330856.6		19.523803	1.065	0.09
6582821		21.217672	2.285	1.19
6879176		17.651029	1.691	0.76
6062536.1		63.331209	6.513	2.7
5971175.2		29.213323	1.117	0.16
6844230.3		16.400297	1.669	0.74
8901241.9		12.13608	1.138	0.19
7811110.9		15.586059	3.384	1.76
7754618.2		25.879479	2.016	1.01
8427499.8		59.155434	77.336	6.27

Only

7543866.5		17.584017	1.213	0.28
8586255.7		1.6049601	1.461	0.55
8315111.3		9.9993193	30.38	4.93
7304111		14.635087	4.362	2.13
7147420.6		16.700474	0.901	-0.15
6571567.4		36.586299	0.99	-0.01
6247491.5		31.790539	2.146	1.1
8357422.6		9.5184677	5.916	2.56
7168177.9		22.162946	0.914	-0.13
7579199.4		11.344352	1.609	0.69
8281283.5		11.758983	1.486	0.57
7269955.8		18.626854	1.157	0.21
4497023.8		47.288077	0.713	-0.49
7249314.2		17.62403	1.404	0.49
5758225.4		2.2655654	0.542	-0.88
6789345		22.319156	2.437	1.29
7535945.2		11.687916	1.147	0.2
7536074.7		20.211364	1.742	0.8
8055504		14.353	0.923	-0.12
3058432.9		55.556206	0.29	-1.79
426202.84		168.92435	0.109	-3.2
8543373.9		8.6011544	0.967	-0.05
7870475.9		21.726321	1.214	0.28
8067313.5		6.9334081	1.215	0.28
7782847.4		12.60162	2.146	1.1
7755212.5		17.674387	0.94	-0.09
6194295.5		32.640093	0.889	-0.17
6419868.4		48.723576	1.169	0.22
6179136.8		32.24052	1.452	0.54
8297402.4		14.898416	2.197	1.14
1065249.5		27.571499	0.102	-3.3
6184210.8		19.122063	2.028	1.02
6733217.7		10.093053	0.644	-0.64
8115890.6		3.0139077	1.419	0.5
8155530.1		7.4613836	1.004	0.01
7858695.9		51.563582	12.749	3.67
7285637.4		21.409513	7.18	2.84
5619569.4		28.351556	1.954	0.97
7486696.4		14.240176	0.719	-0.48
5895040.3		44.550631	1.352	0.44
7555532.5		13.657273	1.032	0.05
8054948.5		7.3121485	2.149	1.1
7523883.5		21.906098	0.747	-0.42
6241660.1		10.843139	0.601	-0.73
368876.6		7.4390465	0.036	-4.81
7087547.5		11.252914	1.001	0
8392095.9		18.28664	1.554	0.64
7607472.2		10.754127	0.947	-0.08
5468179.8		63.979302	3.068	1.62
2829362.6		27.767261	0.274	-1.87

Only

7270859.4		15.218687	0.706	-0.5
6720761.8		14.353538	0.893	-0.16
7121493.2		3.951096	0.693	-0.53
7623975.6		8.9275358	1.41	0.5
7768390.1		11.306035	1.422	0.51
4049601.8		17.961085	0.396	-1.34
5642355.7		22.807048	1.259	0.33
5729509.9		52.36103	1.916	0.94
6985712.7		17.302103	1.043	0.06
6902463.8		17.027263	1.021	0.03
7396252.6		12.114882	0.875	-0.19
6860132.6		21.610951	1.69	0.76
8166361.5		6.0306211	0.806	-0.31
6193465.9		23.415211	1.339	0.42
7958243.4		10.007655	2.999	1.58
7939375.5		9.6322928	0.888	-0.17
6183040.2		17.0568	0.966	-0.05
5945804.1		15.850129	0.589	-0.76
3916234		47.875816	0.94	-0.09
7973357.3		13.20005	0.814	-0.3
7097183.2		15.776408	1.131	0.18
6674951.8		12.270869	1.331	0.41
543333.72		167.35864	1.201	0.26
8059615.8		6.9774597	3.254	1.7
8181368.3		7.1727969	0.9	-0.15
6310266.4		24.800887	0.632	-0.66
3949323.3		78.320506	4.151	2.05
6146421.3		19.27851	3.145	1.65
6470013.7		16.93016	1.423	0.51
7386349.5		11.552033	26.537	4.73
6945303.6		16.864602	0.924	-0.11
5216618.5		65.15531	0.521	-0.94
7421634.2		10.075251	2.599	1.38
8234730.4		9.4357823	1.147	0.2
5752449.5		51.991958	0.837	-0.26
5842768.1		25.086346	0.755	-0.4
3953557.2		61.286738	0.559	-0.84
5765692.9		65.092964	1.517	0.6
6714354		19.003246	0.675	-0.57
3815567		29.672271	0.384	-1.38
7023247.7		10.701994	0.955	-0.07
6408475.9		24.794749	0.88	-0.18
724926.41		4.5441406	0.073	-3.77
7835987		11.321041	8.341	3.06
7706504.4		12.590075	11.85	3.57
1299072.9		10.946445	0.131	-2.93
4784075.7		66.62348	3.959	1.98
3361085		110.28831	9.903	3.31
7206108.6		9.0053755	1.239	0.31
4586889.8		41.477217	0.569	-0.81

Only

7526746.5		4.0609367	0.957	-0.06
7037368.5		12.657804	1.372	0.46
7906212.6		16.321206	0.877	-0.19
7639240.9		22.29931	0.918	-0.12
7968295.3		7.1430632	1.024	0.03
5636348.2		22.731722	0.939	-0.09
7006295.9		15.859586	0.838	-0.25
6794968		14.490961	1.154	0.21
7221631.5		8.7113038	1.224	0.29
3940959.9		49.850713	0.567	-0.82
5764538.1		46.645929	1.037	0.05
6749679.2		11.656241	0.688	-0.54
6139503.2		21.008534	0.626	-0.68
7928434.5		45.079825	0.9	-0.15
2842164.1		11.293553	0.291	-1.78
7085049.2		12.944945	0.794	-0.33
5960686.5		19.904914	0.61	-0.71
6960063.8		12.489256	4.445	2.15
7847625.2		11.72457	1.856	0.89
4709708.9		37.619111	0.483	-1.05
6792721.1		9.8217094	1.553	0.64
6804117.7		36.766991	0.864	-0.21
2002074.5		121.80315	11.077	3.47
2683466.3		36.00408	0.277	-1.85
7770716.8		9.1501402	0.923	-0.12
7923187.2		7.7332888	0.966	-0.05
6275118.6		31.346071	1.036	0.05
242735.83		12.705467	0.025	-5.32
7890624.3		3.0871124	0.942	-0.09
7333587.6		5.5830783	1.574	0.65
7311092.2		11.599555	1.12	0.16
5311037.1		30.916	0.549	-0.87
7818313		4.2150967	0.97	-0.04
5904854.1		43.904493	1.856	0.89
6654946.3		25.587476	1.041	0.06
5574856.5		30.349555	10.634	3.41
6395900.6		31.597729	0.896	-0.16
571987.52		9.1845455	0.059	-4.07
7079557.6		12.510105	0.826	-0.28
7974868.3		2.848101	1.067	0.09
6465366.7		13.116334	0.672	-0.57
7203305		10.806287	1.821	0.86
7726663.5		11.61018	1.248	0.32
5814831.4		33.137545	1.061	0.09
4239663.2		41.209544	1.294	0.37
6612341.3		22.793525	0.691	-0.53
7386934.3		12.378737	0.982	-0.03
7112246.3		22.682913	1.29	0.37
7249766.7		13.5874	1.178	0.24
7543039.3		5.4928125	8.677	3.12

Only

6574311.1		17.242363	1.237	0.31
7883302		5.9999808	1.217	0.28
7659037.4		8.5675469	5.924	2.57
5617542.3		25.267673	1.542	0.62
5336863.3		23.598574	1.007	0.01
5956171		23.482226	1.07	0.1
7350645.8		9.0128952	0.796	-0.33
6545668.2		12.959158	1.726	0.79
7122024.2		14.721469	2.028	1.02
6346343.5		5.239174	0.669	-0.58
5521526.8		53.226483	0.582	-0.78
7661202.6		4.5457391	9.151	3.19
5681807.2		24.424843	1.42	0.51
6880474.2		18.591777	3.169	1.66
7753463.9		3.4552218	1.208	0.27
6089085.2		31.601375	0.668	-0.58
7721057.5		2.3621922	1.394	0.48
6519729.3		17.401838	1.486	0.57
7092910.2		22.36701	0.92	-0.12
5920797.3		27.551529	0.702	-0.51
7357783		10.931477	0.906	-0.14
7662844.8		21.982946	1.039	0.06
7477292.8		14.319917	29.188	4.87
6365551.8		11.372732	6.653	2.73
7283637.2		3.7054406	0.942	-0.09
7596267.4		15.691634	1.197	0.26
7398710.8		11.649721	1.123	0.17
6400623.1		14.654596	0.827	-0.27
7430947.7		12.970551	0.789	-0.34
7097405.7		14.162628	0.966	-0.05
5971835.9		25.509626	0.978	-0.03
7197862.2		6.6341462	0.799	-0.32
4419910.4		45.558447	1.087	0.12
7021951		11.935495	1.056	0.08
7090355.2		4.5645522	1.071	0.1
455431.03		4.8728604	0.049	-4.36
7026753.3		9.0457042	0.769	-0.38
6033873		22.037888	0.953	-0.07
5214079.4		28.711031	0.699	-0.52
6401188.3		19.348257	1.076	0.11
6197740.1		19.477844	1.057	0.08
7304549.2		7.9754718	1.037	0.05
7227977.9		5.4223812	1.01	0.01
6785538.3		20.311937	1.034	0.05
3956418.1		98.412465	0.767	-0.38
7690823.7		8.8027778	0.891	-0.17
7190806.4		64.195054	22.874	4.52
5608541.5		21.167185	0.746	-0.42
7141630.8		18.130062	5.576	2.48
6938238.1		9.4022096	1.261	0.33

Only

7250383.6		3.7206108	1.025	0.04
7436021.3		12.299453	5.956	2.57
7146387.8		14.95424	2.806	1.49
6857866.2		18.502711	1.651	0.72
5623361.1		21.413131	0.875	-0.19
7348869		5.450937	0.884	-0.18
7029115.6		12.482879	3.149	1.65
6149103.6		11.384357	0.672	-0.57
4589268.8		13.734178	0.502	-0.99
6268904		24.509837	9.925	3.31
4309732.3		16.709775	0.472	-1.08
3175365.4		59.9206	0.376	-1.41
6970460.3		6.0222022	1.1	0.14
5468129.9		4.894492	0.601	-0.73
5776587.4		17.786759	0.853	-0.23
6396433.4		25.946823	1.835	0.88
5623738		31.893335	19.366	4.28
7020404.7		7.126332	1.062	0.09
6562634.4		14.156569	0.994	-0.01
6084867.5		15.295034	0.923	-0.12
6621173.4		13.567635	2.054	1.04
6919528.2		16.186646	0.952	-0.07
6043650.8		23.341105	1.15	0.2
6200276.8		19.065433	1.589	0.67
7161206.8		7.851058	1.009	0.01
5448127.7		14.687462	0.607	-0.72
7371121.4		1.5226399	0.92	-0.12
7044951.8		7.268929	11.851	3.57
7109361.8		9.6622221	0.944	-0.08
6333353.4		13.173365	1.592	0.67
5759867		19.13908	1.218	0.28
7012693.3		8.6584118	1.656	0.73
2806815.9		18.90427	0.315	-1.67
4900240.7		17.594531	0.55	-0.86
6101161.7		20.107863	9.655	3.27
6107745.4		20.085929	0.92	-0.12
6034508.4		16.114112	19.02	4.25
6008672.4		16.038151	1.023	0.03
6489693.3		16.572591	1.118	0.16
6895457.4		7.2709607	0.824	-0.28
7162142.7		7.738585	0.959	-0.06
5945013.1		44.376244	0.787	-0.35
657903.79		5.3432754	0.075	-3.74
6920839.8		5.5808279	1.38	0.46
6955753.6		12.640558	1.023	0.03
7193727.7		2.6004893	1.134	0.18
7149637.6		16.753701	2.16	1.11
2338786.8		39.753148	0.267	-1.91
6618770.8		13.401103	0.755	-0.4
4707702.8		30.438399	3.389	1.76

Only

4396229.3		63.148169	0.575	-0.8
5239161.7		28.954982	0.86	-0.22
6596139.7		7.5029166	1.016	0.02
7154096		65.597669	0.842	-0.25
6557010.8		64.487833	19.893	4.31
5588407.6		65.794775	0.91	-0.14
5766776.7		21.734615	0.985	-0.02
3726561.3		49.578172	1.895	0.92
6365759.7		9.1119737	0.96	-0.06
5588188.5		17.797089	0.776	-0.37
7048162.6		3.374392	0.9	-0.15
1889188.7		72.888295	0.218	-2.2
7037397.3		3.6484189	0.94	-0.09
6375986.7		9.9799541	4.661	2.22
383532.49		7.4390465	0.044	-4.5
5483175.7		18.547716	2.997	1.58
6024338.9		16.388414	1.259	0.33
6517206.1		11.841658	6.684	2.74
6136562.4		22.505587	0.892	-0.16
1262689		35.824428	0.146	-2.77
6138662.5		19.838922	1.399	0.48
4551608.5		4.518006	0.527	-0.92
6723400.1		6.8019975	1.006	0.01
6102897.5		12.301337	0.708	-0.5
3276052.8		52.227843	1.41	0.5
3750489.6		89.330523	9.677	3.27
6251398.1		9.4478832	0.989	-0.02
5016543.8		24.180481	3.733	1.9
5105295.6		18.24686	0.593	-0.75
6472504.4		11.245289	4.644	2.22
6819531.2		20.083811	0.857	-0.22
3716700.5		46.675756	0.523	-0.94
4586360.2		46.148119	1.344	0.43
6819544.6		2.4332271	0.796	-0.33
6688649.5		8.037842	0.998	0
3330586.9		98.210409	7.5	2.91
5422457.7		21.894546	2.929	1.55
6628529.6		9.6966656	9.856	3.3
6336042.5		11.237944	1.039	0.06
3928164.8		37.866294	0.907	-0.14
4139331.1		44.491409	0.731	-0.45
5221025.8		41.129885	0.818	-0.29
5335965.3		31.736281	1.284	0.36
6659956.9		8.4253193	0.88	-0.18
134116.43		187.20174	0.645	-0.63
5334914.8		24.523265	1.119	0.16
7022788		2.7747612	1.057	0.08
5762984		30.220832	1.44	0.53
6900760.6		4.7217326	0.939	-0.09
5397719.6		6.5285004	0.637	-0.65

Only

6407332.4		12.807456	2.063	1.04
6805818.6		6.77454	0.999	0
6573655		6.0615104	0.969	-0.05
6349880.7		13.263615	1.218	0.28
5959955.8		9.1976355	0.928	-0.11
6716691.7		13.882438	1.057	0.08
6772384.6		8.8285032	0.952	-0.07
4141233.9		46.070575	0.492	-1.02
5219306.1		29.80248	0.773	-0.37
2553203.5		97.64042	0.582	-0.78
2521214.1		32.495891	0.3	-1.74
4962874.1		22.065491	1.424	0.51
6506201.5		6.6205661	1.075	0.1
3784774.1		27.424166	0.452	-1.15
6213352.5		5.9879306	1.02	0.03
5852051.7		11.306182	0.7	-0.51
6421729		9.8647648	1.029	0.04
6035506.5		10.948345	1.06	0.08
4662257.8		31.396501	0.971	-0.04
6559254.4		7.3670933	8.354	3.06
6431492.5		7.4164197	13.411	3.75
6473453.3		7.7356623	1.223	0.29
5744697		21.163165	1.025	0.04
5638125.4		11.601027	6.708	2.75
6303011.3		15.062308	0.969	-0.04
3776982.4		37.555897	0.52	-0.94
208228.75		180.36989	0.046	-4.44
2871926.7		35.332	0.348	-1.52
5848820.3		9.2010966	0.9	-0.15
4209904.3		11.115357	0.511	-0.97
5580964.3		13.004097	1.11	0.15
5618274.8		5.4725636	0.682	-0.55
4821016.7		24.151164	2.584	1.37
1693007.7		38.595876	0.206	-2.28
5246814.2		45.309049	0.774	-0.37
5496886.5		28.208996	1.43	0.52
5708267.9		12.636365	0.697	-0.52
6177057.9		11.25749	1.275	0.35
6333805.2		15.126199	0.885	-0.18
5812334.9		16.818314	12.605	3.66
5782929.8		15.145904	1.46	0.55
6106486.6		26.579108	4.539	2.18
5882656.6		11.93801	1.214	0.28
3070229		7.5037803	0.378	-1.4
6274269.4		13.651948	1.885	0.91
5948683.5		9.4778131	0.932	-0.1
6061418.7		11.714184	0.983	-0.03
5809755.3		27.105656	3.403	1.77
4524929.2		60.8751	0.674	-0.57
4403574.3		27.882747	14.508	3.86

Only

5788839.1		43.224596	0.95	-0.07
5345543.1		21.621097	0.661	-0.6
5965592.9		6.6098055	1.473	0.56
6037494.6		11.432754	0.759	-0.4
4064434.4		37.662695	1.589	0.67
6092605		15.881945	1.823	0.87
6625082.7		5.6262791	16.873	4.08
6260967.6		45.359853	0.958	-0.06
5835460.1		7.1536213	3.637	1.86
6243225.6		5.9090564	1.003	0.01
5428432.8		14.395602	1.226	0.29
4210127		33.473732	1.234	0.3
6074961.5		8.1052931	0.934	-0.1
6226050		3.3587137	1.383	0.47
5940097.9		42.133935	0.982	-0.03
6608729.3		13.1488	1.093	0.13
5129759.3		24.677778	12.53	3.65
4198196.7		52.709395	0.882	-0.18
3756627.1		66.354614	0.475	-1.07
6364554.2		11.114905	4.489	2.17
5406126		35.980374	1.215	0.28
5814866.4		10.554478	1.879	0.91
5161432.3		17.60588	0.655	-0.61
5975738.4		11.746034	8.653	3.11
5732327.8		13.375167	0.876	-0.19
5901488		16.185498	0.839	-0.25
5956886.3		15.644504	1.21	0.27
2514967.4		110.91385	2.641	1.4
4349161		34.953702	1.057	0.08
451899.64		4.8728604	0.058	-4.12
4990047.1		18.285188	1.05	0.07
6354357.5		4.6815131	1.813	0.86
6291378.5		3.5147293	0.88	-0.18
3117325.8		59.68306	1.111	0.15
5985646.2		7.1816561	0.965	-0.05
6022907.3		2.7988698	1.081	0.11
6111172.6		14.442493	0.806	-0.31
4459300.7		31.839132	1.108	0.15
5627922.7		11.434145	2.996	1.58
5605035.4		25.326872	0.929	-0.11
5457324.8		16.1319	0.951	-0.07
6136439.4		11.313908	1.048	0.07
4708087.5		19.545644	1.212	0.28
489949.45		9.5325478	0.064	-3.98
6039294		5.5939178	1.374	0.46
5020336.5		31.282615	3.217	1.69
4631528.8		41.063751	0.803	-0.32
6227862.2		43.581344	1.386	0.47
4647670.1		23.979048	1.599	0.68
2119105.1		22.780982	0.278	-1.85

Only

5446687.9		64.562491	13.339	3.74
4675896.6		17.848395	0.745	-0.43
3368264		46.808536	0.488	-1.03
5353743.2		10.761806	0.827	-0.27
5894821		15.885808	1.221	0.29
6003454.1		9.1941984	1.354	0.44
5626987.6		14.533954	0.8	-0.32
5992021.3		6.5441807	1.039	0.06
4613020.7		25.775451	2.669	1.42
380058.64		154.8678	0.05	-4.31
5758956.7		11.520413	0.82	-0.29
5923826.2		10.956239	1.104	0.14
6128832.9		7.2408165	1.22	0.29
5187659		14.221867	0.895	-0.16
5323704.2		26.922162	0.939	-0.09
4388568.1		6.3322888	0.585	-0.77
5452221.3		16.458379	1.087	0.12
4951372.9		24.203583	1.423	0.51
1565002.3		96.715966	0.209	-2.26
5813736.4		41.361204	1.082	0.11
5182713.4		14.463606	0.694	-0.53
4771924.6		23.304876	0.919	-0.12
4683139.9		17.361004	6.169	2.62
5311176.1		9.4442387	0.957	-0.06
3789235.9		33.924117	0.641	-0.64
5818395.7		9.827877	0.959	-0.06
5812284.2		3.8279006	5.877	2.56
4695220.2		59.272304	12.903	3.69
5875497.4		19.086689	0.94	-0.09
5579672.2		10.422833	0.861	-0.22
6108014		23.811608	1.237	0.31
5381103.1		17.936785	1.204	0.27
3667127.8		50.030184	0.726	-0.46
4144935.6		61.848088	1.292	0.37
5618146.7		11.088236	2.553	1.35
5129465.7		8.8974516	1.229	0.3
5360767.4		15.148827	2.505	1.32
5117592.6		19.378355	0.699	-0.52
2078896.6		15.084738	0.284	-1.82
5281831.2		9.5974871	1.18	0.24
3412168.5		53.170892	1.396	0.48
4663369.2		14.06398	1.208	0.27
1388646.8		80.208096	0.19	-2.39
5086739.7		13.743043	1.133	0.18
4786471.8		19.139137	0.938	-0.09
5135008		1.7659313	0.705	-0.5
3256213.1		32.71352	0.447	-1.16
5679662.2		9.150816	0.783	-0.35
5622172.6		6.2674624	1.358	0.44
5641793.8		9.0447468	1.292	0.37

Only

5873243.5		5.3680272	0.889	-0.17
4965665.4		18.959401	2.912	1.54
4576284		19.592631	0.633	-0.66
5714023		3.3201508	4.644	2.22
4862995.6		11.916814	1.212	0.28
5328525.3		7.6140299	1.644	0.72
4219837.2		29.844602	0.987	-0.02
1908368.2		98.781369	4.018	2.01
3566692.4		49.685016	0.496	-1.01
4840598.7		21.58957	4.154	2.05
5690404.2		5.4166942	6.579	2.72
4594922.6		14.541393	0.639	-0.65
5202470		10.619602	1.646	0.72
5016303		20.841737	1.29	0.37
4977763.7		24.714215	1.315	0.39
4340271.9		21.706102	0.674	-0.57
4853160.3		15.931131	1.661	0.73
2932812.6		54.994764	0.53	-0.92
5501870.7		9.4449178	3.033	1.6
3825289.2		34.066241	0.535	-0.9
4400197.1		33.176668	1.194	0.26
4829982.5		6.9719007	0.676	-0.56
659903.69		6.610894	0.092	-3.44
5498453.4		14.178039	6.222	2.64
4476763		16.477065	1.013	0.02
5027020.7		18.036836	0.765	-0.39
4173780.5		31.772067	0.586	-0.77
5209214.2		19.524536	1.182	0.24
4655812.8		22.072006	0.657	-0.61
4542241.9		31.461591	1.191	0.25
5120398.9		64.335961	17.4	4.12
5099277.5		9.2635931	1.372	0.46
146721.78		183.41256	0.645	-0.63
5827406.1		21.115788	0.861	-0.22
4327897.4		18.691157	0.952	-0.07
5834234.6		9.1968393	0.981	-0.03
3067767		91.167016	5.626	2.49
3229843		40.522763	0.892	-0.17
5776180.5		4.3792119	1.133	0.18
4235796.1		6.3570005	0.603	-0.73
1869111.3		58.684341	0.267	-1.91
4679522.8		66.91309	19.222	4.26
4936360.4		11.773125	1.698	0.76
4890180.4		22.024467	1.201	0.26
1914373		52.584935	0.274	-1.87
4936704.4		30.896413	0.828	-0.27
5143671.4		20.339928	1.052	0.07
1482828.1		15.950435	0.212	-2.23
5257590.4		10.605395	0.754	-0.41
4463801.1		17.237534	0.776	-0.37

Only

805836.49		7.3039529	0.116	-3.11
5152581.5		10.299165	4.442	2.15
5395314.3		7.1354206	1.466	0.55
5072219.4		13.968469	0.872	-0.2
4731493.9		21.774832	0.773	-0.37
1292780.6		6.7732641	0.187	-2.42
5581438.4		7.6487534	1.106	0.15
4518150.7		28.072862	0.652	-0.62
5184449.1		12.611088	0.833	-0.26
4239851.6		21.393737	1.017	0.02
5660197.5		5.2050196	1.04	0.06
5387240.4		15.002591	1.126	0.17
2555990.6		49.225262	0.37	-1.43
3359332.2		54.160721	1.793	0.84
288940		7.4390465	0.042	-4.57
3070252.3		95.977037	7.092	2.83
3819075		28.427248	1.729	0.79
5142609.9		19.394835	37.146	5.22
5202179.6		9.412049	4.796	2.26
989951.39		84.051993	0.144	-2.79
4954238.5		13.189502	0.841	-0.25
3898248.2		21.611912	3.236	1.69
2361298.5		18.780284	0.346	-1.53
5228919.7		12.174033	4.435	2.15
5527555.6		4.8440034	5.724	2.52
5278981.1		9.0349981	1.255	0.33
3827449.8		38.126699	0.762	-0.39
1006266.7		47.549921	0.149	-2.75
5148585.1		18.947735	0.797	-0.33
5574585		12.268745	1.495	0.58
4564855.6		12.760338	1.025	0.04
4718451.1		14.5992	7.69	2.94
5009201.2		15.82321	0.764	-0.39
3589450.7		46.642682	0.557	-0.85
4900629.2		11.118271	0.788	-0.34
4921991		16.721136	2.063	1.04
4765272.2		23.657124	1.212	0.28
5117539.1		12.129574	0.939	-0.09
3423186.6		33.120826	1.261	0.33
4883559.5		19.267257	1.862	0.9
4753849.6		20.640174	1.186	0.25
3197072.7		62.367171	0.48	-1.06
4982848.4		14.287514	1.135	0.18
4948657.7		5.6399193	0.988	-0.02
5467115.5		7.8949528	0.98	-0.03
4610050.2		13.488169	1.072	0.1
4718662.8		13.748045	1.01	0.02
4103979.9		27.187008	0.904	-0.15
5022481.7		9.2339372	1.083	0.12
4690901.2		12.960136	1.498	0.58

Only

5222505.9		22.252507	1.008	0.01
2005564.4		23.366	0.306	-1.71
1022633.7		66.906357	0.156	-2.68
5274277.5		5.2887897	0.946	-0.08
4414231.3		24.872011	0.887	-0.17
4886716.5		25.129381	2.052	1.04
4982315.4		12.037961	6.801	2.77
4622466.2		17.711344	7.31	2.87
2284826.1		104.04511	0.35	-1.51
3435491.9		20.504807	0.527	-0.92
4708465.6		22.378048	5.275	2.4
3965989.5		19.720712	1.196	0.26
4490068.5		11.316185	1.238	0.31
4977281.4		6.4102984	5.303	2.41
5150403.7		13.456895	2.361	1.24
2787661.1		105.67075	6.843	2.77
4507207.2		15.653208	2.983	1.58
4265047.7		17.366813	1.824	0.87
4987204.8		11.947334	1.32	0.4
3857588		34.469125	5.222	2.38
3392508.2		29.560439	0.527	-0.92
1950672.4		25.192231	0.303	-1.72
2210920		80.959994	1.114	0.16
4846742.7		8.1199114	0.757	-0.4
3107953.5		11.412695	0.486	-1.04
4213292.3		20.174576	1.039	0.06
4568285.6		9.8992416	1.082	0.11
3171839.1		34.750214	1.225	0.29
841866.62		129.18012	0.439	-1.19
5010538		4.0765745	18.406	4.2
4199918.2		24.452056	1.973	0.98
4574099.8		17.062917	1.101	0.14
2318459		17.339021	0.364	-1.46
172752.47		5.5344583	0.027	-5.2
5141591.8		28.617025	1.267	0.34
5062221		7.975906	1.564	0.65
4659377		10.854898	0.94	-0.09
3926658		20.734951	1.268	0.34
4919961.3		7.4540012	0.782	-0.35
4193201.7		31.649117	0.949	-0.07
2530082.4		21.681151	0.404	-1.31
4227669.1		24.458826	0.798	-0.33
4574692.7		11.986716	2.128	1.09
4289420.9		16.671138	0.896	-0.16
4221994.2		20.037195	2.705	1.44
2487645.7		57.076268	1.336	0.42
3786413.3		24.585853	0.64	-0.64
3301584		48.352472	0.756	-0.4
2536328.8		46.875097	11.148	3.48
3374283.3		26.928797	1.015	0.02

Only

4927915.6		10.713593	1.024	0.03
3288997.3		26.809056	0.78	-0.36
3972112.4		19.183936	0.815	-0.3
4743121.1		9.560234	3.989	2
4960422.6		10.805454	1.062	0.09
4652418.4		9.3198207	1.328	0.41
4479953.1		10.217516	7.361	2.88
4932524.1		26.909238	0.799	-0.32
5001616.2		8.8859055	1.392	0.48
1383780.4		92.904973	0.961	-0.06
843233.96		4.8728604	0.137	-2.87
3598585.4		36.734593	1.088	0.12
3889843.8		34.439274	1.668	0.74
4180687.6		9.4759527	0.681	-0.55
4609034.3		8.785619	1.389	0.47
3400242.4		49.826042	0.554	-0.85
3964955		26.544235	0.685	-0.55
4389938.3		60.530489	6.997	2.81
4147403.6		29.358116	0.7	-0.52
3091032.6		44.356967	1.32	0.4
581795.79		8.1817307	0.096	-3.38
4726385.6		8.2971836	0.814	-0.3
3903442.1		15.382944	1.157	0.21
4446848.7		30.752793	3.872	1.95
4396892.4		27.590181	1.739	0.8
3295312.2		59.01482	2.024	1.02
4484306.2		7.9662774	1.282	0.36
4303444.6		25.333696	1.867	0.9
4235892.7		11.168629	0.873	-0.2
1712074.6		38.53705	0.288	-1.8
628007.67		8.0402043	0.106	-3.24
3236819.2		55.662969	0.718	-0.48
4508739.4		18.42446	1.116	0.16
3935343		23.660866	0.895	-0.16
4563598.6		5.4027103	1.991	0.99
3355926.8		52.703764	1.55	0.63
1597690.4		35.813434	0.273	-1.87
156901.98		178.90932	0.645	-0.63
4425262.6		31.779868	1.657	0.73
4147698.9		13.134353	1.603	0.68
4482999.5		7.5701904	0.952	-0.07
4352065.3		11.116969	1.353	0.44
4161485.9		18.463681	1.04	0.06
2138557.4		27.966035	0.37	-1.43
4522087.4		9.1390808	1.299	0.38
3805264.4		55.756309	0.837	-0.26
3697946		18.821014	13.159	3.72
985173.04		117.03648	2.166	1.12
4287897.3		6.2061838	1.044	0.06
3281420.9		31.799163	0.902	-0.15

Only

2250047.8		100.56014	6.534	2.71
2691664.7		37.117056	0.472	-1.08
4371698.1		9.8668197	1.176	0.23
2139530.3		51.90395	0.654	-0.61
4292873.2		38.985081	1.064	0.09
4516270.6		12.871452	0.907	-0.14
4483041.6		12.296674	4.726	2.24
3414235.9		30.244927	0.635	-0.66
3951061.8		18.133531	1.523	0.61
2806781.7		29.580393	1.278	0.35
2658783		59.957	0.716	-0.48
2470975.3		84.281866	3.119	1.64
4553764.4		8.8731536	1.461	0.55
3801427.2		39.008806	1.56	0.64
3872567.3		30.814527	0.759	-0.4
514384.59		71.785927	0.092	-3.44
3949442.6		17.753337	0.779	-0.36
946004.6		5.0984407	0.17	-2.56
2879799.6		36.292134	0.916	-0.13
4517991.5		9.0114929	1.535	0.62
3901331.9		21.967551	0.867	-0.21
3763045.7		10.875549	0.906	-0.14
4477073.1		3.4330433	0.889	-0.17
1348897.9		87.9681	0.255	-1.97
3794269		24.707676	3.785	1.92
3676514.1		21.156278	1.052	0.07
4308475.5		5.084986	0.819	-0.29
2949696.4		30.030061	1.267	0.34
4226419.9		9.5110359	1.441	0.53
3726749.4		25.094119	0.923	-0.11
3945221.6		17.146136	12.566	3.65
4106440		11.525328	0.746	-0.42
1807668.2		20.710369	0.329	-1.6
3351623.4		24.268969	1.431	0.52
3880099.4		22.826448	1.33	0.41
3703995.5		16.937732	0.895	-0.16
4180977		6.1076919	1.253	0.33
1575259.6		98.619225	0.288	-1.8
4228361.9		5.3959969	0.98	-0.03
4329240.9		2.2294315	0.872	-0.2
848167.5		41.88896	0.156	-2.68
4492434.1		10.006874	1.371	0.46
3919080		26.09555	1.363	0.45
3744617.9		21.34739	0.785	-0.35
3581906.4		13.119177	1.336	0.42
3580824.8		17.085573	0.661	-0.6
4257863.4		3.7427753	0.859	-0.22
2801067.2		32.615417	0.973	-0.04
2675817.2		13.052176	0.497	-1.01
2947401.4		31.168246	0.652	-0.62

Only

3749555.6		13.215354	0.741	-0.43
2697469.2		52.215837	3.171	1.66
3432194.7		7.9619088	0.642	-0.64
3928464.5		10.096522	0.948	-0.08
3972067.8		8.8665918	0.745	-0.42
3562124.5		63.18342	1.06	0.08
4073250.3		3.6899726	0.985	-0.02
2009580.3		69.43496	0.388	-1.37
3954766.3		10.333423	8.062	3.01
4377320.8		8.7886026	1.655	0.73
4391357.2		4.454785	1.333	0.41
3792450.7		12.280839	0.901	-0.15
3957962.6		14.525341	1.342	0.42
4113053.3		27.827008	1.063	0.09
1299568.6		48.975386	0.246	-2.02
2897873.6		13.076905	0.549	-0.87
3151629.9		39.946682	1.492	0.58
3427141.3		25.227867	1.126	0.17
3214330.7		32.964403	1.498	0.58
3238962.6		28.665879	2.467	1.3
3562009.4		15.021173	1.154	0.21
390590.48		147.7161	0.862	-0.21
4100728.8		3.9472507	1.232	0.3
2151038.9		32.265543	0.412	-1.28
2395840.6		50.031513	1.927	0.95
3864783.7		11.888126	3.794	1.92
1215457.1		107.99914	2.593	1.37
3359800.5		20.215639	2.837	1.5
670683.38		117.55352	0.602	-0.73
3593374.5		22.709161	1.914	0.94
3840741.3		13.357692	1.054	0.08
4177798.4		5.838762	1.578	0.66
3636946.8		59.576721	6.785	2.76
2692055.1		39.001558	0.904	-0.15
3395345.4		13.688575	0.657	-0.61
3081994.1		7.2682283	0.597	-0.75
3715639.4		10.956736	1.036	0.05
3928581.8		6.1883374	1.58	0.66
4234433.6		0.9461781	1.234	0.3
3774795		12.990862	2.573	1.36
3194643		19.773061	0.92	-0.12
3380407.1		21.373148	0.659	-0.6
3294476.9		30.081964	1.664	0.73
807889.34		110.82333	0.921	-0.12
4111561.5		10.569414	0.847	-0.24
3027765.6		32.068778	1.932	0.95
485225.71		11.430977	0.095	-3.39
3896744.7		47.817699	2.48	1.31
3512140.5		19.494163	1.418	0.5
583864.75		131.19977	0.188	-2.41

Only

2634972.1		24.537482	0.519	-0.95
4081687.8		8.1254596	0.854	-0.23
2826678		23.77088	1.07	0.1
2368933.1		46.927419	0.652	-0.62
1677164.1		93.484642	3.615	1.85
3613408.7		12.331578	3.127	1.64
3711663.1		11.773963	0.759	-0.4
2082223.5		43.179247	0.415	-1.27
2691162		11.519354	0.537	-0.9
3628662.3		8.0416058	0.739	-0.44
3940515		21.255543	2.113	1.08
653325.82		116.53924	0.655	-0.61
852893.69		11.519673	0.171	-2.55
2509700.6		28.923818	0.835	-0.26
3313289.5		16.748747	0.934	-0.1
947621.13		41.007434	0.191	-2.39
3573822.4		9.2248305	1.006	0.01
3518646.5		14.749497	0.71	-0.5
2111100.1		79.573688	0.598	-0.74
3784197.3		5.1118332	1.099	0.14
3348039.4		40.80782	0.99	-0.01
2302863.5		39.155214	0.68	-0.56
3852262.4		3.3098108	4.51	2.17
1288696.6		107.90681	4.186	2.07
3594637.2		6.8417215	0.996	-0.01
1727840.7		62.218579	3.418	1.77
3754702.2		9.466761	3.379	1.76
4020724.1		5.7180436	0.825	-0.28
1772752.7		6.4333234	0.364	-1.46
3810342.1		11.923602	1.069	0.1
3491433.9		8.7186067	1.556	0.64
3811251.2		14.386026	0.902	-0.15
3064417.8		18.450351	1.163	0.22
3904788.3		14.735301	3.871	1.95
2024931.3		7.1371786	0.419	-1.26
3629274.2		12.102012	1.502	0.59
1969355.2		48.851409	0.408	-1.29
3881387.8		4.2094518	9.978	3.32
2069589.9		63.792255	1.708	0.77
955406.41		48.597318	0.2	-2.32
3983932.4		6.3208929	1.078	0.11
3243623		42.650063	0.743	-0.43
3401271.9		14.526288	0.91	-0.14
3498193.1		7.2964009	1.285	0.36
3454620.8		10.11017	0.915	-0.13
3379428.8		16.341481	1.071	0.1
3673358.2		7.7122549	1.237	0.31
3110337.9		17.461725	5.826	2.54
2468839.1		66.903939	7.5	2.91
3432831.2		12.421774	0.837	-0.26

Only

3639765.7		5.4321039	3.142	1.65
2927865.6		23.264983	1.33	0.41
3348829.6		12.611779	1.106	0.15
3837869		5.1385925	0.845	-0.24
3823285.3		4.4639432	1.109	0.15
3075094.9		20.055524	0.731	-0.45
2671656.3		59.580987	5.428	2.44
3573603.6		13.381098	1.07	0.1
2461135.4		77.60857	0.933	-0.1
3842871.7		3.4447903	4.734	2.24
3419648.9		11.662411	1.11	0.15
3076589.8		27.248496	1.772	0.83
2672005.2		25.569047	0.947	-0.08
499817.37		4.8728604	0.106	-3.23
3223204.3		15.264937	1.065	0.09
3536975.4		11.987188	0.986	-0.02
3301494.4		9.666344	5.485	2.46
795071.26		88.368563	0.171	-2.55
3459796.8		5.7385075	3.118	1.64
2217347.9		17.805236	0.479	-1.06
3331576.3		8.1733019	0.719	-0.48
3657486.2		13.778135	0.94	-0.09
3381997		20.94171	1.116	0.16
2055229.8		38.624853	0.754	-0.41
3549137.3		9.5711734	0.942	-0.09
3177486		28.147382	4.106	2.04
2834130.7		21.336327	0.96	-0.06
2999690.6		35.360618	0.878	-0.19
3422760.2		12.906937	0.941	-0.09
3386381.2		13.43199	1.198	0.26
3359644.1		16.431807	0.959	-0.06
3080249.3		14.890749	0.863	-0.21
3443868.8		11.679524	1.074	0.1
3584092.2		6.7333905	1	0
2949248.4		16.875977	1.099	0.14
3741527.6		5.0798964	1.603	0.68
1176994.1		77.611703	3.771	1.91
444858.92		140.84384	0.115	-3.12
3529821.7		8.8361802	13.01	3.7
2848664.8		19.681057	1.432	0.52
2956448.3		24.471648	1.127	0.17
3401890.5		7.3183897	1.006	0.01
3572245.5		11.500113	0.947	-0.08
1980435.6		98.125343	5.472	2.45
2593277.5		75.337157	1.008	0.01
3079799.4		12.518378	0.938	-0.09
3110263.1		19.953315	0.958	-0.06
3286434.6		8.4758378	1.153	0.21
3138950.4		34.082673	0.909	-0.14
3511385.6		29.249973	1.555	0.64

Only

2270658.5		56.429067	0.511	-0.97
3159998.2		13.253835	2.075	1.05
3220904.7		10.161605	2.073	1.05
3186124.3		24.209203	1.171	0.23
1806061.8		98.090574	0.44	-1.18
3370838.7		17.423452	4.257	2.09
1991988.9		46.638767	0.829	-0.27
2167338.1		35.199104	0.581	-0.78
2513827.2		27.250045	1.264	0.34
3011981.1		38.232639	10.322	3.37
3025048.6		10.012139	0.861	-0.22
3513709.9		3.753145	0.896	-0.16
2905845.2		13.17415	2.342	1.23
3459334.1		8.8554041	0.962	-0.06
3073694		9.8875788	1.184	0.24
3128119.1		6.5359447	1.028	0.04
2882782.6		17.469836	0.744	-0.43
2943007.2		25.741435	1.039	0.06
2445315.8		25.816546	0.722	-0.47
2904909.6		16.932567	0.679	-0.56
3113642.6		11.133676	0.729	-0.46
1752788.3		92.850931	1.334	0.42
2770283.5		19.687502	1.012	0.02
3170705		9.0691602	1.12	0.16
2615760.1		30.936566	7.095	2.83
1033289.7		60.115671	0.243	-2.04
2883067.4		38.534525	2.172	1.12
3009410.7		24.363531	2.239	1.16
3302074.9		11.216364	1.182	0.24
3033824.1		22.338791	1.189	0.25
2513117.2		25.603989	1.207	0.27
2942785		34.00684	1.149	0.2
3315573.4		9.5217719	1.045	0.06
3014966.8		43.833461	0.846	-0.24
3248746.2		15.242039	1.215	0.28
2569720.9		22.519884	3.04	1.6
3305349.2		5.7689834	0.979	-0.03
2982700.3		13.188283	0.912	-0.13
3024051.6		9.7830147	0.725	-0.46
3067702.9		12.084327	1.064	0.09
3016450.5		14.335247	3.874	1.95
2978755.4		18.275658	1.678	0.75
2527893.7		23.93641	1.108	0.15
3179565.6		35.732659	2.549	1.35
3114246.6		27.930088	2.136	1.1
3327804.5		19.950382	1.808	0.85
479199.21		129.4651	1.029	0.04
2109670.1		35.049394	0.759	-0.4
2937844.7		12.768241	0.908	-0.14
2873806.1		25.962692	2.436	1.28

Only

2551025.7		20.723281	0.639	-0.65
3145808.7		18.834497	1.336	0.42
3110008		9.6965422	1.267	0.34
2068978.6		30.225766	0.595	-0.75
3141537.4		11.838984	1.03	0.04
2954499.9		12.120087	1.075	0.1
2826411.9		38.767804	0.708	-0.5
2657592.6		18.318759	1.851	0.89
3054377.8		8.690474	1.945	0.96
1771690.6		43.373285	1.199	0.26
2437900.2		25.570765	0.851	-0.23
2868821		10.738089	0.862	-0.21
2412885.4		16.022463	1.151	0.2
3195046		5.5271821	1.102	0.14
3069876.6		7.6952515	0.855	-0.23
2645194.6		24.022257	1.538	0.62
3091319.7		12.756606	1.045	0.06
3063045.2		22.651572	0.93	-0.11
2846369.5		8.2913718	8.063	3.01
2155953.6		27.324566	4.792	2.26
3100313		8.5810996	1.074	0.1
2700829		12.851615	1.171	0.23
3105157.7		12.188739	1.515	0.6
2939804.3		55.170253	0.943	-0.08
2970829.9		12.10727	12.171	3.61
3054626.6		9.2672239	1.207	0.27
2309583.4		30.082887	1.119	0.16
2561191.4		20.405177	1.968	0.98
2484753		13.580371	3.141	1.65
415600.47		110.20275	0.109	-3.2
2930077.4		11.19759	1.547	0.63
3065958		17.539193	1.411	0.5
2360934.2		40.67301	2.914	1.54
2576450.5		12.545684	3.088	1.63
2774839.7		10.006752	1.046	0.07
2495582.1		17.293948	0.749	-0.42
2758884.9		58.999238	0.755	-0.4
2682688.8		16.95493	1.159	0.21
1363512.3		85.459802	13.908	3.8
2820046.5		6.0974753	1.047	0.07
1628398.6		74.310311	0.922	-0.12
2119734		29.266853	1.4	0.49
2653891.8		57.305077	6.048	2.6
2252377.8		25.520765	1.296	0.37
2163228.1		32.787847	0.793	-0.33
2918409.4		23.197525	2.045	1.03
3005990.4		15.907901	1.481	0.57
2569757		32.843843	0.851	-0.23
1981325.1		29.250747	2.136	1.09
2819090.6		13.640974	1.568	0.65

Only

2649946.5		16.281269	1.152	0.2
208897.08		91.014151	0.057	-4.13
2042791.9		37.17492	1.26	0.33
2643589.6		14.014316	1.144	0.19
2156396.6		32.063838	0.595	-0.75
2670296.9		31.462212	1.173	0.23
2880719.9		4.3287027	1.075	0.1
2545164.9		18.020657	0.958	-0.06
2507391.6		21.184737	0.694	-0.53
2409995.8		19.973975	1.486	0.57
2643954.8		19.627556	0.943	-0.08
2570363.6		11.732045	0.959	-0.06
2856736.7		7.8138619	2.163	1.11
1856642.4		33.622467	0.802	-0.32
2305128.9		31.717394	3.251	1.7
2500521.8		19.524687	0.944	-0.08
2826193.4		13.065758	1.454	0.54
1944444		48.569994	1.61	0.69
2571380.4		16.779432	1.534	0.62
2606238		24.770181	2.415	1.27
1959852.4		15.663595	0.559	-0.84
2718707.4		9.3364678	1.143	0.19
1998966.2		23.317479	0.781	-0.36
2346325.4		18.624361	0.868	-0.2
1760379.6		73.288641	5.501	2.46
2799291.8		16.94372	1.049	0.07
2453971		63.297084	2.852	1.51
2383003.8		11.517144	1.149	0.2
2028700.4		27.44412	0.88	-0.18
2384563.2		27.268221	3.262	1.71
2073842.8		32.876127	1.999	1
2446922.4		22.608702	1.212	0.28
368499.98		33.630964	0.107	-3.23
2458886.1		9.7184286	0.734	-0.45
1126265.5		31.049963	0.328	-1.61
2393331.8		31.876945	3.084	1.62
2759425.8		11.333549	1.38	0.47
2710581		8.2424269	0.821	-0.28
1732823.9		11.131957	0.507	-0.98
1568833.7		22.435652	0.461	-1.12
2204194.1		40.228737	2.034	1.02
2292057.7		16.615335	2.066	1.05
2326933.8		50.788508	1.287	0.36
2614896		25.667982	1.725	0.79
1802368.7		26.110473	1.091	0.13
2648170.4		12.682548	1.494	0.58
260056.66		122.33981	0.077	-3.69
2777692.9		12.255552	1.092	0.13
2442704.4		31.590069	1	0
2205065.2		16.945958	0.989	-0.02

Only

1849542.9		63.477357	1.902	0.93
2243899.5		14.56724	0.898	-0.15
2150392.7		24.173979	6.938	2.79
2044033		34.695752	0.622	-0.69
2604700.3		5.9127654	1.198	0.26
2551899.3		4.2248322	9.705	3.28
2034910		39.792837	1.907	0.93
1915460.8		7.4481898	0.589	-0.76
2550260.4		9.5561088	1.12	0.16
2591332.9		9.883389	1.125	0.17
1896108		23.408171	1.742	0.8
2049986		24.496993	1.368	0.45
2553073.6		9.8137645	1.052	0.07
1404612.9		49.32154	0.563	-0.83
1983039.7		43.043803	11.471	3.52
2076037.3		62.483762	3.983	1.99
1100749.1		72.48483	1.102	0.14
2353473.6		12.831919	1.362	0.45
2019077.1		22.768368	1.617	0.69
1679483.7		57.075699	1.735	0.8
2374901.5		10.97047	1.069	0.1
874882.13		95.893022	1.965	0.97
1799854.7		48.51934	0.614	-0.7
2035448.3		42.023888	1.546	0.63
2442088.5		8.2140536	0.888	-0.17
2024097.1		27.404118	6.927	2.79
2291021.5		8.7846058	4.52	2.18
2238294.9		36.25327	2.541	1.35
1097113.5		97.126669	3.169	1.66
1836622.9		42.218774	0.605	-0.72
1843081.1		32.636055	0.972	-0.04
1157150.9		56.143327	4.76	2.25
1995262.8		33.902953	0.926	-0.11
1349017.5		50.430419	2.206	1.14
1079877.6		69.063935	0.902	-0.15
1289258.8		80.830956	2.578	1.37
1870354.5		39.942188	1.975	0.98
583427.33		30.355543	0.199	-2.33
1935142.7		26.941971	1.629	0.7
1206734.9		84.39409	2.615	1.39
2345266.2		12.663554	1.305	0.38
1945331.2		36.404094	0.664	-0.59
2395651.1		2.4093461	0.865	-0.21
261244.62		9.9325541	0.09	-3.48
1948572.3		20.002716	3.379	1.76
1345021.6		53.077451	0.491	-1.03
2219221.9		11.802741	1.237	0.31
1986744.4		41.391039	2.85	1.51
2107021.6		18.433687	1.278	0.35
1240709.1		57.316066	1.645	0.72

Only

1685892.8		25.682213	1.156	0.21
2047517.7		14.467719	3.893	1.96
1604980.6		22.823488	4.521	2.18
2309428.4		8.9233044	0.809	-0.31
916184.27		97.744337	1.769	0.82
2287631.8		11.551885	1.423	0.51
1609212.8		36.325388	2.389	1.26
1799457.4		26.691148	1.001	0
2265657.8		3.2648687	1.262	0.34
1000765.5		59.728683	0.568	-0.82
1528676.1		29.52501	5.705	2.51
2098066.1		5.6131001	1.919	0.94
1497583.7		41.994327	5.053	2.34
1500580.6		41.808948	2.046	1.03
2046142.7		22.451606	1.213	0.28
2021032.7		13.223276	0.928	-0.11
2108242.6		14.129421	4.455	2.16
1866588.5		22.776598	1.088	0.12
799993.22		91.972649	2.91	1.54
2193975		10.377045	0.901	-0.15
1265659.4		54.888742	0.491	-1.03
2153059.7		9.6118325	7.499	2.91
1975489.8		10.484368	1.023	0.03
1531000		24.928298	0.573	-0.8
2052968.6		11.63409	1.064	0.09
1563466.7		21.811575	1.484	0.57
2133836		4.8482476	2.075	1.05
1848004.6		7.5026086	0.798	-0.32
1716481		36.474593	4.001	2
1788776.3		36.972741	0.857	-0.22
1392444.5		44.915798	0.645	-0.63
1057671.1		46.134992	1.341	0.42
1695694.3		15.684881	1.363	0.45
1485467.8		31.310771	6.777	2.76
1473534.5		28.606111	1.023	0.03
1560415.8		59.968947	5.398	2.43
1757717.9		27.998083	1.006	0.01
1946698.4		11.815677	1.134	0.18
1779529.2		13.905656	0.943	-0.08
1705362.7		22.368471	1.028	0.04
1499460.6		26.177441	2.391	1.26
1060299.7		55.803759	3.652	1.87
1604655.1		19.201437	0.947	-0.08
1624666.8		50.37878	3.017	1.59
980015.89		16.913064	0.408	-1.29
1537223.9		13.478829	0.641	-0.64
1555504.1		25.46439	0.821	-0.28
748186.43		33.596771	0.314	-1.67
1621435.2		37.071289	1.063	0.09
1408549		27.047962	0.601	-0.73

Only

1427653		19.050565	6.254	2.64
1686926.1		39.622356	1.335	0.42
1184367.8		34.654744	0.525	-0.93
1132896.9		59.884835	8.827	3.14
1078934.7		71.932971	0.506	-0.98
884239.91		69.750683	1.232	0.3
1682853.6		15.98862	1.303	0.38
148987.08		150.56923	0.645	-0.63
1515704.1		14.478134	0.945	-0.08
1475162.2		9.9475184	1.207	0.27
1673279.3		14.943699	1.238	0.31
1345904		15.285157	0.8	-0.32
1201802.9		29.284681	0.713	-0.49
1498528.7		13.684118	1.429	0.51
1354484.5		12.583643	2.491	1.32
1187373.9		17.118925	1.155	0.21
131598.12		152.71023	0.645	-0.63
609708.1		84.920094	5.196	2.38
1382790.3		25.274263	0.721	-0.47
1504523.4		7.9527318	5.686	2.51
1481450.4		12.81737	1.629	0.7
1417816		8.870717	0.994	-0.01
1112408.3		47.615934	1.615	0.69
1499438.2		52.904303	4.52	2.18
1178016.9		41.04725	1.292	0.37
1161650.4		30.34745	0.945	-0.08
1260185.8		42.497335	6.219	2.64
1117710.6		28.153221	1.035	0.05
1240795.9		9.308094	4.898	2.29
863466.82		59.709078	1.564	0.64
1088915.3		12.943729	0.834	-0.26
1275633.5		6.9349177	6.545	2.71
691746.1		40.72366	0.801	-0.32
1074166.3		9.3556045	0.677	-0.56
925114.8		45.531095	2.128	1.09
840972.18		47.192164	1.062	0.09
681042.97		52.69428	1.747	0.8
1034735.2		36.393959	0.661	-0.6
919792.54		24.494847	0.687	-0.54
446878.96		70.623661	0.494	-1.02
472776.3		85.116891	1.987	0.99
833043.61		26.272563	1.033	0.05
918637.97		23.763092	5.554	2.47
437898.67		66.957905	2.15	1.1

Only

Supplementary Table 2 Primers used for qPCR

Name	Forward Sequence	Reverse Sequence
**GAPDH**	GCATCCACTGGTGCTGCC	TCATCATACTTGGCAGGTTTC
**ACC1**	GAGTGACTGCCGAAACATCTCT[illegible]	GCAAGGAGGACAGAGTTTATCGTG
**SCD1**	CGGAAATGAACGAGAGAAGG	CCGAAGAGGCAGGTGTAGAG
**C/EBPα**	AGACATCAGCGCCTACATCG	TGCAGGTGCATGGTGGTC
**Fas**	GCTGCGGAAACTTCAGGAAAT	AGAGACGTGTCACTCCTGGACTT
**SREBP-1c**	GATGTGCGAACTGGACACAG	CATAGGGGGCGTCAAACAG
**PPAR-γ**	GCAGCTACTGCATGTGATCAAG.	GTCAGCGGGTGGGACTTTC

For Review Only

邮箱使用情况说明

万江红高级工程师是成都大学药食同源植物资源开发高校重点实验室客座研究员，供职于四川姜氏茶业有限公司，她主要从事藏茶营养和发酵工艺改进研究。她所在公司没有专用邮箱，她也没有成都大学邮箱，她使用腾讯商业邮箱：8491490@qq.com。

特此证明

四川姜氏茶业有限公司
年 月 日

成都大学药食同源植物资源开发
高校重点实验室
年 月 日

Dear Editor

Senior Engineer, Jianghong Wan is a visiting researcher at the key laboratory of Medicinal and Edible Plants Resources Development of Sichuan Education Department of Chengdu University, working for Sichuan Jiang's Tibetan Tea Co., LTD., where she is mainly engaged in the research of Tibetan tea nutrition and fermentation process improvement. Her company does not have a special email address, nor does she have an email address for Chengdu University. she uses Tencent Business Email: 8491490@qq.com.

Sichuan Jiang's Tibetan Tea Co., LTD

Key Laboratory of Medicinal and Edible Plants Resources Development of Sichuan Education Department

2023-3-28

563x796mm (72 x 72 DPI)
